# The pharmacological and non-pharmacological treatment of attention deficit hyperactivity disorder in children and adolescents: A systematic review with network meta-analyses of randomised trials

**DOI:** 10.1371/journal.pone.0180355

**Published:** 2017-07-12

**Authors:** Ferrán Catalá-López, Brian Hutton, Amparo Núñez-Beltrán, Matthew J. Page, Manuel Ridao, Diego Macías Saint-Gerons, Miguel A. Catalá, Rafael Tabarés-Seisdedos, David Moher

**Affiliations:** 1 Clinical Epidemiology Program, Ottawa Hospital Research Institute, Ottawa, Ontario, Canada; 2 Fundación Instituto de Investigación en Servicios de Salud, Valencia, Spain; 3 Department of Medicine, University of Valencia/INCLIVA Health Research Institute and CIBERSAM, Valencia, Spain; 4 School of Epidemiology, Public Health and Preventive Medicine, Faculty of Medicine, University of Ottawa, Ottawa, Ontario, Canada; 5 Centro de Atención Integral a Drogodependientes (CAID) Norte, Regional Health Council, Madrid, Spain; 6 School of Public Health and Preventive Medicine, Monash University, Melbourne, Australia; 7 School of Social and Community Medicine, University of Bristol, Bristol, United Kingdom; 8 Instituto Aragonés de Ciencias de la Salud (IACS), Red de Investigación en Servicios de Salud en Enfermedades Crónicas (REDISSEC), Zaragoza, Spain; 9 Division of Pharmacoepidemiology and Pharmacovigilance, Spanish Medicines and Healthcare Products Agency (AEMPS), Madrid, Spain; 10 Faculty of Medicine, University of Valencia, Valencia, Spain; Copenhagen University Hospital, DENMARK

## Abstract

**Background:**

Attention deficit hyperactivity disorder (ADHD) is one of the most commonly diagnosed psychiatric disorders in childhood. A wide variety of treatments have been used for the management of ADHD. We aimed to compare the efficacy and safety of pharmacological, psychological and complementary and alternative medicine interventions for the treatment of ADHD in children and adolescents.

**Methods and findings:**

We performed a systematic review with network meta-analyses. Randomised controlled trials (≥ 3 weeks follow-up) were identified from published and unpublished sources through searches in PubMed and the Cochrane Library (up to April 7, 2016). Interventions of interest were pharmacological (stimulants, non-stimulants, antidepressants, antipsychotics, and other unlicensed drugs), psychological (behavioural, cognitive training and neurofeedback) and complementary and alternative medicine (dietary therapy, fatty acids, amino acids, minerals, herbal therapy, homeopathy, and physical activity). The primary outcomes were efficacy (treatment response) and acceptability (all-cause discontinuation). Secondary outcomes included discontinuation due to adverse events (tolerability), as well as serious adverse events and specific adverse events. Random-effects Bayesian network meta-analyses were conducted to obtain estimates as odds ratios (ORs) with 95% credibility intervals. We analysed interventions by class and individually. 190 randomised trials (52 different interventions grouped in 32 therapeutic classes) that enrolled 26114 participants with ADHD were included in complex networks. At the class level, behavioural therapy (alone or in combination with stimulants), stimulants, and non-stimulant seemed significantly more efficacious than placebo. Behavioural therapy in combination with stimulants seemed superior to stimulants or non-stimulants. Stimulants seemed superior to behavioural therapy, cognitive training and non-stimulants. Behavioural therapy, stimulants and their combination showed the best profile of acceptability. Stimulants and non-stimulants seemed well tolerated. Among medications, methylphenidate, amphetamine, atomoxetine, guanfacine and clonidine seemed significantly more efficacious than placebo. Methylphenidate and amphetamine seemed more efficacious than atomoxetine and guanfacine. Methylphenidate and clonidine seemed better accepted than placebo and atomoxetine. Most of the efficacious pharmacological treatments were associated with harms (anorexia, weight loss and insomnia), but an increased risk of serious adverse events was not observed. There is lack of evidence for cognitive training, neurofeedback, antidepressants, antipsychotics, dietary therapy, fatty acids, and other complementary and alternative medicine. Overall findings were limited by the clinical and methodological heterogeneity, small sample sizes of trials, short-term follow-up, and the absence of high-quality evidence; consequently, results should be interpreted with caution.

**Conclusions:**

Clinical differences may exist between the pharmacological and non-pharmacological treatment used for the management of ADHD. Uncertainties about therapies and the balance between benefits, costs and potential harms should be considered before starting treatment. There is an urgent need for high-quality randomised trials of the multiple treatments for ADHD in children and adolescents. PROSPERO, number CRD42014015008.

## Introduction

Attention deficit hyperactivity disorder (ADHD) is one of the most commonly diagnosed psychiatric disorders in children and adolescents. Recent estimates suggest that ADHD affects about 3–7% of young people worldwide [1–2], producing considerable impact on health services and the community [3–5]. ADHD is a childhood-onset disorder characterized by a persistent pattern of symptoms of developmentally inappropriate and impaired inattention and/or hyperactivity/impulsivity, with difficulties often continuing into adulthood. The diagnosis of the disorder also requires the presence of symptoms across more than one setting (e.g., home and school) and requires that the symptoms needed for diagnosis result in academic, social, or occupational impairment [6–8].

In recent years, the management of ADHD has become increasingly complex as new therapies are introduced in clinical practice. The comparative efficacy and safety of pharmacological and non-pharmacological treatments are largely unknown, mainly because of the paucity of head-to-head trials. A wide variety of interventions have been used for the treatment of ADHD, including pharmacological and psychological interventions, herbal and homeopathic remedies, and dietary management. Despite the extensive body of research into the epidemiology and pathophysiology of ADHD, less emphasis has been placed on methodologically sound comparative research questions evaluating and comparing different ADHD treatment options. For example, which ADHD treatment modality works best in children and adolescents, psychological interventions, pharmacological therapy or both? Among broad groups of treatment interventions, is there any particular treatment which is clinically superior (or inferior) to others? Is there a unique role of complementary and alternative medicine used in the treatment of children and adolescents with ADHD? Although the evidence is not strong, pharmacological treatment using stimulant medication is generally recommended for school-age children and adolescents with ADHD, along with implementation of psychological interventions based on behavioural approaches [9–11]. Very frequently, however, parents consider using complementary and alternative medicine as a therapeutic option for controlling the core ADHD symptoms. Randomised trials and systematic reviews have evaluated the effects of pharmacological and non-pharmacological interventions in children and adolescents with ADHD [12–19], but most research has been limited to a particular treatment approach only without considering all available treatment alternatives, comparators and outcome measures of clinical importance.

Knowledge gaps in the current evidence base present opportunities for the application of novel evidence synthesis methods to complex networks of trials and interventions, as well as rigorous comparative evaluations of multiple treatments for ADHD. Relatively new methods have been developed to analyse such complex trial data, including network meta-analysis, which helps to integrate and synthesise diverse results to determine the relative merits of multiple treatments. Network meta-analysis has been recommended as a next generation tool for evidence synthesis when a heterogeneous set of treatments and trials is included in the same review [20–24]. Network meta-analysis may also be of particular use when included interventions are not only heterogeneous, but complex in nature (e.g., treatments that may operate through a variety of causal pathways) [23, 25–28]. To our knowledge, none of the previous research has attempted to establish evidence-based comparative effects of all pharmacological and non-pharmacological ADHD treatments in a comprehensive systematic review using network meta-analyses.

Accordingly, we aimed to compare the efficacy and safety of pharmacological, psychological and complementary and alternative medicine interventions for the treatment of ADHD in children and adolescents. We used network meta-analyses comparing multiple interventions to integrate direct and indirect evidence into unified networks of all available randomised trials.

## Methods

This systematic review was conducted and reported in accordance with the reporting guidance provided in the PRISMA statement extension for systematic reviews incorporating network meta-analysis [29] (S1 Checklist). We developed a systematic review protocol and registered with PROSPERO (CRD42014015008) [30]. Our methods are briefly described here (and explained in more detail in S1 Text and in the published protocol [30]).

### Eligibility criteria

We included randomised controlled trials which: (1) had at least three weeks follow-up (three weeks per treatment arm in parallel-group trials and three weeks in the first randomisation period for crossover studies) as this is the minimum length of treatment chosen in trials designed to measure dose responses or efficacy; (2) compared pharmacological or non-pharmacological interventions against each other or against placebo/control in the treatment of children and adolescents (under 18 years of age) with a diagnosis of ADHD (e.g., following the Diagnostic and Statistical Manual of Mental Disorders [DSM] criteria or the International Classification of Diseases [ICD]). All ADHD subtypes (e.g., combined type, predominantly inattentive and predominantly hyperactive/impulsive) were considered for inclusion. We included trials that enrolled patients with comorbid conditions (such as oppositional defiant, conduct disorder, anxiety, depression, epilepsy or other ADHD associated medical conditions). Eligible interventions were pharmacological, psychological, complementary and alternative medicine, and combined interventions. Pharmacological interventions were not required to be licensed for ADHD. Control conditions were placebo or no treatment, waitlist (in psychological studies), and usual care or control (however defined in the trials) (see Box 1 for definitions of interventions). We assumed that patients who fulfilled the inclusion criteria were equally eligible to be randomised to any of the interventions of interest.

**Box 1. Brief description of interventions being evaluated**

**Pharmacological interventions**. Pharmacological interventions refer to the treatment of ADHD using medication, under the supervision of a medical professional. Studies evaluating any of the following drugs at any therapeutic dose were considered: *Stimulants* (e.g., methylphenidate, amphetamine); *Non-stimulants* (e.g., atomoxetine, guanfacine, clonidine, antidepressants (e.g., bupropion, venlafaxine, reboxetine, desipramine, imipramine); *Antipsychotics* (e.g., risperidone, aripiprazole, thioridazine); *Other unlicensed drugs* (e.g. modafinil, carbamazepine).

**Psychological interventions**. A diverse range of psychological therapies is available for the treatment of ADHD in children and adolescents. Psychological interventions were: *Behavioural therapy*: an intervention directed at changing behaviours (increasing desired behaviours and decreasing undesired behaviours), based on social learning principles and other cognitive theories. These include classical contingency management, behaviour therapy (mainly through mediators such as parents or teachers) and cognitive behaviour therapy (such as verbal self-instruction, problem solving strategies or social skills training). These treatments are usually offered in several sessions over time, either through training the parents (teachers) or the child or both. *Cognitive training*: working memory training incorporating adaptive schedules that are hypothesized to strengthen ADHD-deficient neuropsychological processes. We retained studies including training interventions that aim to directly train a cognitive function, or working memory, or attention (e.g. working memory training, attention training). *Neurofeedback* using the visualization of brain activity to teach children to increase attention and impulse control. Neurofeedback is commonly based on electroencephalography; sensors are placed on the scalp to measure activity, and measurements displayed using video displays or sound. By learning to control their brain activity based on behavioural principles of operant conditioning, it is hypothesized that ADHD patients will learn to regulate the associated attentional states and processes.

**Complementary and alternative medicine interventions**. Complementary and alternative medicine is a group of diverse medical and health care systems, practices and products that are not presently considered to be part of conventional medicine. A diverse range of interventions are being used for the treatment of ADHD in children and adolescents: *Dietary therapy*, such as restricted elimination diet or ‘few foods approach’ (exclusion of items associated with food hypersensitivity, sometimes referred to as an oligoantigenic diet) and artificial food colour elimination from child’s diet (e.g. removing food colours such as azo dyes, tartrazine, carmoisine, sunset yellow, brilliant blue, indigotine, allura red, quinoline yellow or ponceau 4R); *Polyunsaturated fatty acids* (PUFA e.g. omega-3 and omega-6 fatty acids); *Vitamins* (e.g., vitamin B6, vitamin B9, vitamin B12, vitamin C); *Minerals* (e.g., magnesium, zinc, iron, calcium); *Amino acids* (e.g., acetyl-L-carnitine, gamma-aminobutyric acid, glycine, L-tyrosine). *Herbal therapy* (e.g., Ginkgo Biloba, Ginseng, St John’s Wort/*Hypericum perforatum*, Valerian); *Homeopathic treatment* and any other supplementary interventions; *Mind- and body-based interventions* (such as physical activity or exercise).

**Control comparators**. Eligible control conditions were: *Placebo* (psychological or pill), *waiting list* (in psychological studies), and *control* (usual care, conventional therapy or control).

The pre-specified primary outcomes were treatment response and all-cause treatment discontinuation rates, chosen because they are among the most clinically meaningful and consistently reported estimates of treatment efficacy and acceptability in mental health research [30,31]. We defined response as the proportion of patients who displayed improvements in the symptoms of ADHD or global functioning on standardized rating scales–e.g., pre-defined cut-off points for this specific age group such as ‘much improved’ or ‘very much improved’ on the clinical global impression (CGI), or a reduction of at least 25% from the baseline score on the ADHD Rating Scale (ADHD-RS). When trials reported results from multiple rating scales, we used CGI results as the preferred scale. Clinicians, teachers or parents, and patients rated symptoms and global functioning. As per our review protocol [30], clinician rated measures were given preference in the primary analyses of treatment response. We defined all-cause discontinuation (acceptability) as the proportion of patients who left the study early for any reason, measured at the longest available follow-up. Secondary outcomes were treatment tolerability, serious adverse events and specific adverse events, respectively. We defined tolerability as the proportion of patients who have left the study early due to adverse events, measured at the longest available follow-up. Serious adverse events were defined as the occurrence of any untoward medical event that resulted in death, was life-threatening, required inpatient hospitalization or prolonged existing hospitalization, resulted in persistent or significant disability/incapacity, or was an important medical event. Specific adverse events of interest included the occurrence of anorexia (or loss of appetite), weight loss (or decreased weight gain), insomnia, sleep disturbances (unspecified), anxiety, syncope, and any cardiovascular adverse event.

### Electronic literature search

We used a staged approach to study identification, beginning with a systematic search of relevant trials included in systematic reviews available in PubMed/MEDLINE (January 1, 2005 –April 7, 2016), the Cochrane Database of Systematic Reviews (The Cochrane Library, issue 4 of 12, April 2016) and/or existing meta-analyses of which we were aware (S2 Text for details of search terms and S3 Text for references of previous reviews) with no language restrictions. PubMed/MEDLINE was next searched to identify other additional relevant trials published outside the time frames of previous reviews (up to May 6, 2016). We compiled a list of the unique PubMed/MEDLINE identification numbers of all relevant articles, and performed a related articles search. This technique has been shown to be highly effective in identifying relevant studies [32], increases efficiency in study identification in the presence of an already large evidence base and is being used as part of an ongoing network meta-analysis research program [33,34]. Searches were supplemented by consulting alternative databases (PsycINFO and AMED), public clinical trial registers (e.g., www.clinicaltrials.gov) and manufacturer clinical trial registers (e.g., www.lillytrials.com and www.shiretrials.com), regulatory agencies websites (e.g., the European Medicines Agency and the U.S. Foods and Drug Administration), and review of references of relevant papers, health technology assessment reports and clinical practice guidelines. We contacted authors for missing outcome data or unclear information. Furthermore, we contacted the European Medicines Agency for unpublished data through its recent transparency policy [35].

### Screening, data extraction and risk of bias assessment

Eligible trials identified from our searching efforts were independently screened by two reviewers, first at the abstract level and then at the full text level for those considered potentially eligible. Using a predesigned form that was piloted on a small sample (n = 30; 16%) of studies, two reviewers were responsible for independent data extraction on general characteristics (e.g. average age, gender, subtype and duration of ADHD, initial severity, patient comorbidity history, past medication use with stimulants, mean or median follow-up) and outcome data. The Cochrane Risk of Bias tool [36] that considers sequence generation, allocation concealment, and masking and other aspects of bias, was used to assess each included study’s risk of bias. The overall rating of risk of bias for each study was the worst rating for any of the criteria (e.g., if any domain is scored high risk of bias, the study was considered high risk of bias). Risk of bias assessments were independently performed by two of four reviewers. We used the Grading of Recommendations Assessment, Development, and Evaluation (GRADE) methodology [37,38] to evaluate the quality of evidence for each outcome. Quality of evidence was adjudicated as high (further research is very unlikely to change our confidence in the estimate of effect), moderate (further research is likely to have an important impact on our confidence in the estimate of effect and may change the estimate), low (further research is very likely to have an important impact on our confidence in the estimate of effect and is likely to change the estimate), or very low (very uncertain about the estimate of effect). This approach for grading the strength of the body of evidence was consistent with previous reporting of network meta-analyses of healthcare interventions [33,39]. Any discrepancies between reviewers for any of the above steps were discussed until consensus was achieved.

### Geometry of the evidence networks assessed

We analysed interventions by class (e.g., behavioural therapy, stimulants, non-stimulants, etc.) and individually (e.g., parent training, methylphenidate, etc.). In general, treatments with similar mechanisms of action were classified in classes in which treatment effects were considered to be similar. Our primary class-level analyses classified therapeutic class interventions used in monotherapy and/or combination as separate treatment nodes irrespective of their doses: controls (placebo, usual care/control and waiting list), psychological interventions (behavioural therapy such as parent training, child training and teacher training; cognitive training including attention training and working memory training; neurofeedback; other psychotherapies), pharmacological interventions (stimulants including methylphenidate and amphetamines; non-stimulants including atomoxetine, guanfacine and clonidine; antidepressants; antipsychotics and other unlicensed drugs) and complementary and alternative medicine interventions (dietary therapy such as elimination diet; polyunsaturated fatty acids; amino acids; minerals; herbal therapy; homeopathy; and physical activity). For the primary outcomes, we expressed comparative effects of therapeutic class interventions using placebo as the reference comparator. Additionally, we also expressed individual effects of commonly prescribed pharmacological interventions [40] using methylphenidate as the reference treatment, because methylphenidate has been used for the treatment of ADHD for over 50 years and is now the most frequently prescribed drug for ADHD worldwide [13].

### Methods for evidence synthesis

Whenever possible we used results from intention-to-treat analyses in which the original random participant assignment was maintained in the data analyses. For each outcome, we presented graphically the geometry of the treatment network of all comparisons [41]. Using a Bayesian framework, we performed network meta-analyses for each pre-specified outcome and treatment. Network meta-analyses [42,43] allow the integration of direct and indirect evidence to increase precision while randomisation is preserved, but can also be used to estimate comparisons between pairs of treatments that have not been compared in individual trials. Network meta-analyses rely on the assumptions of transitivity (that is, one can learn about treatment A versus treatment B via a common comparator C) and consistency (equivalency of treatment effects from direct and indirect evidence) [29,44]. We used random effects network meta-analysis models as recommended elsewhere assuming a common heterogeneity parameter across all comparisons, accounting for correlations in multi-arm studies [45] and using vague (non-informative) prior distributions for all treatment effects. We reported the results as posterior median odds ratios (ORs) with corresponding 95% credibility intervals (CrIs), which are the Bayesian analogue of 95% CIs. Network meta-analyses were generally based on a total of 40,000 iterations or more with a burn-in of 20,000 iterations. We assessed convergence on the basis of Brooks–Gelman–Rubin plots [46]. To calculate direct estimates of treatment effect, we conducted pairwise random-effects meta-analyses for primary outcomes. We reported the results as odds ratios (ORs) and corresponding 95% confidence intervals (CIs). Statistical heterogeneity was assessed by the I^2^ index [47] and the Cochran’s Q chi-square test [48]. Small study effect bias (sometimes referred to publication bias) was examined with the use of funnel plots and the Begg’s adjusted rank correlation test [49,50]. Consistency was examined by fitting both consistency and inconsistency models [51] for network meta-analysis and comparing the deviance information criteria (DIC) between models, with smaller values indicative of a better fit and a difference of five or more being considered as important. In general, when both models had a similar fit to the data as indicated by their DIC values, we concluded that there was no evidence of inconsistency. League table structure has been used to present the findings from network meta-analyses.

### Sensitivity analyses performed

Sensitivity analyses were conducted to explore the impact of potentially important effect modifiers on findings from network meta-analysis. These included separate analyses that involved exclusion of the following: studies at overall high risk of bias, studies where both the assessor and the patient (or caregiver) were unmasked (commonly called ‘unblinded’ trials), and small studies (with less than 100 participants). Other pre-planned analyses were the extension of the primary unadjusted network meta-analysis model to include covariates in meta-regression models [52] that considered the following: year of publication, study duration (follow-up), mean age of trial participants, % of male participants, and baseline risk (e.g., response rate in placebo as a proxy of medical history, severity, comorbidities or other unmeasured underlying factors). Several subgroup analyses for treatment response effects included: type of scale (ADHD symptoms-based compared to global functioning-based rating scale) and type of rater (clinicians compared to other). Because of the chronic course of ADHD, we also analysed studies of different durations separately in relation to response outcome measured during initial short-term treatment (the first 6 weeks of treatment with a range of 3 to 12 weeks), mid-term treatment (24 weeks of treatment with a range of 13 to 48 weeks) and long-term treatment (more than 48 weeks). In addition, sensitivity analyses of the geometry of treatment networks were conducted including the dosage of stimulants (amphetamine low to moderate dose ≤ 20 mg/day compared to high dose >20 mg/day; methylphenidate low to moderate dose ≤ 30 mg/day compared to high dose >30 mg/day) and type of formulations (short-acting compared to long-acting preparations).

### Software considerations

All Bayesian network meta-analyses were conducted using WinBUGS 1.4.3 (MRC Biostatistics Unit, Cambridge, UK), while pairwise meta-analyses and network diagrams were generated using Stata 13 (StataCorp LP, College Station, Texas, USA).

## Results

### Extent of relevant literature identified

The systematic search identified 5,606 citations (from 116 systematic reviews and updated searches), of which 804 were examined in full-text review and 540 were excluded. Accordingly, we included 190 studies (described in 264 publications). A PRISMA flowchart documenting the process of study selection is shown in S1 Fig, while the full reference listing of included studies is provided in S4 Text. Studies included a total of 26,114 participants randomly assigned to one of the study treatments or control conditions; details of the included studies (including general characteristics of patients such as average age, gender distribution, study follow-up, treatment comparisons, etc.) are shown in S1–S5 Tables. Key characteristics are described next.

### Overview of study and patient characteristics

The mean duration of follow-up of included studies was 12 weeks (range 3–96) and the mean sample size was 137 (range 9–1323), with 91 trials (47.9%) having at least 100 participants and 7 trials (3.8%) having more than 400. Studies assessed 52 interventions or control conditions, which were grouped in 32 therapeutic classes (125 trials evaluated pharmacological interventions, 48 trials evaluated psychological interventions and 39 trials their combinations). Most studies (86%) included two groups; while smaller totals included three groups (11%) or more (3%). Most studies were conducted in North America and Western Europe (75%). At baseline, the mean and median age was 10 years (range 3–16) and the mean percentage of male participants was 77%. Fifty-three trials excluded children and adolescents with a comorbidity. Oppositional defiant disorder was the most common comorbidity (mean 44% of participants at baseline among studies reporting data), followed by conduct disorder (21%). In terms of methodological quality (S3 Table), 100 trials (52.6%) had a high risk of bias for at least one criterion, 85 trials (44.7%) had an unclear risk of bias and 5 trials (2.6%) had a low risk of bias. Ninety-six trials (50.5% of all trials; 76.8% of trials evaluating pharmacological interventions) were funded by drug companies. We obtained unpublished supplementary information for 42 of the included trials (19 from manufacturer documents, 18 from trial registry/regulatory documents, 3 from investigators, and 2 other sources).

**Fig 1:**
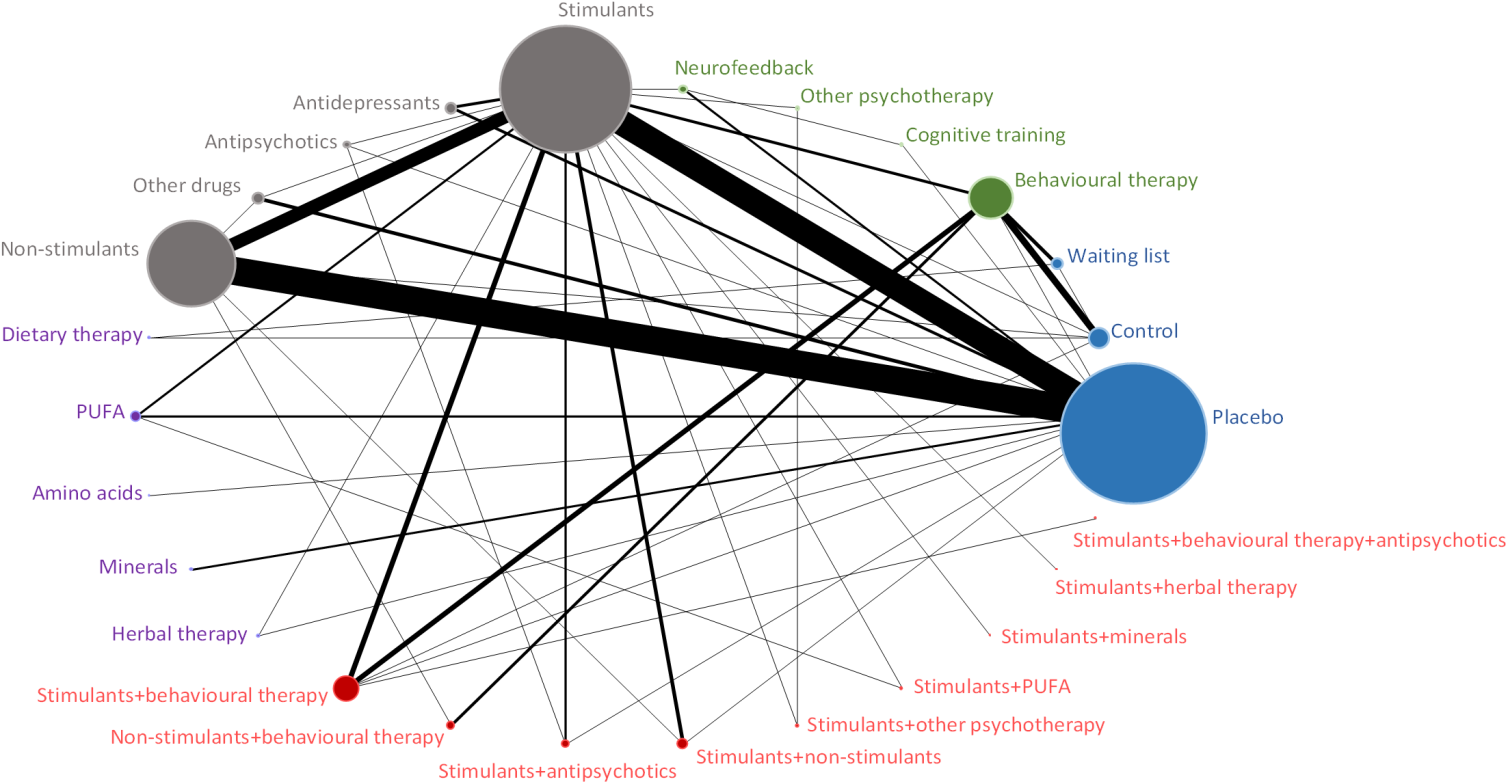
Evidence network diagram for primary outcome of efficacy among therapeutic classes. Solid lines represent direct comparisons within randomised trials. Nodes in blue represent controls. Nodes in green represent psychological interventions. Nodes in grey represent pharmacological interventions. Nodes in purple represent complementary and alternative medicine interventions. Nodes in red represent combined interventions. Size of node is proportional to number of randomised trials, and thickness of line connecting nodes is proportional to number of randomised trials directly comparing the two treatments. PUFA = polyunsaturated fatty acids.

**Fig 2:**
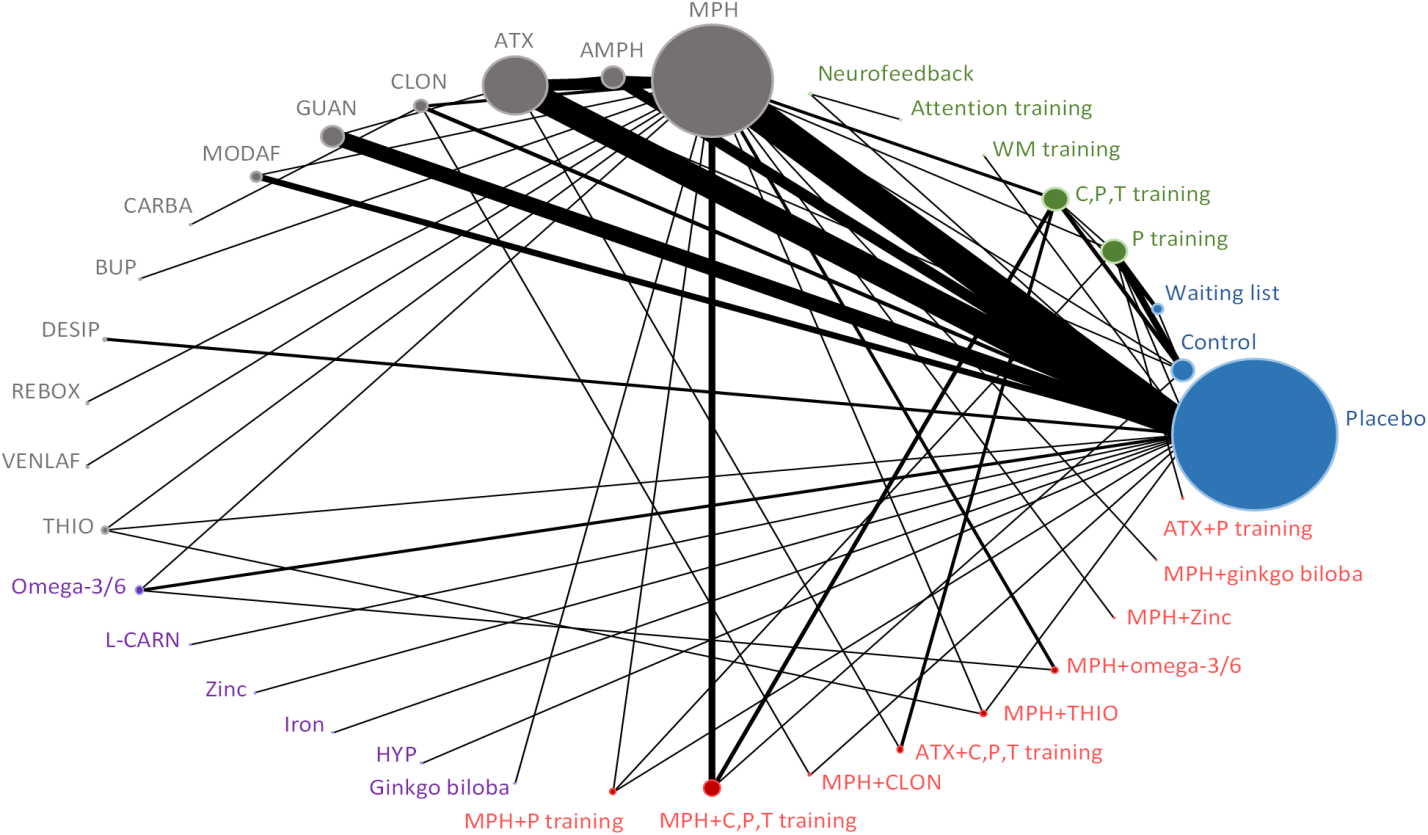
Evidence network diagram for primary outcome of efficacy among individual therapies. Solid lines represent direct comparisons within randomised trials. Nodes in blue represent controls. Nodes in green represent psychological interventions. Nodes in grey represent pharmacological interventions. Nodes in purple represent complementary and alternative medicine interventions. Nodes in red represent combined interventions. Size of node is proportional to number of randomised trials, and thickness of line connecting nodes is proportional to number of randomised trials directly comparing the two treatments. AMPH = amphetamine. ATX = atomoxetine. CLON = clonidine. GUAN = guanfacine. MODAF = modafinil. CARBA = carbamazepine. BUP = bupropion. DESIP = desipramine. REBOX = reboxetine. VENLAF = venlafaxine. RISP = risperidone. THIO = thioridazine. L-CARN = L-carnitine. HYP = hypericum. C, P, T training = child, parent and/or teacher training. P training = parent training. C training = child training. T training = teacher training. WM training = working memory training.

**Fig 3:**
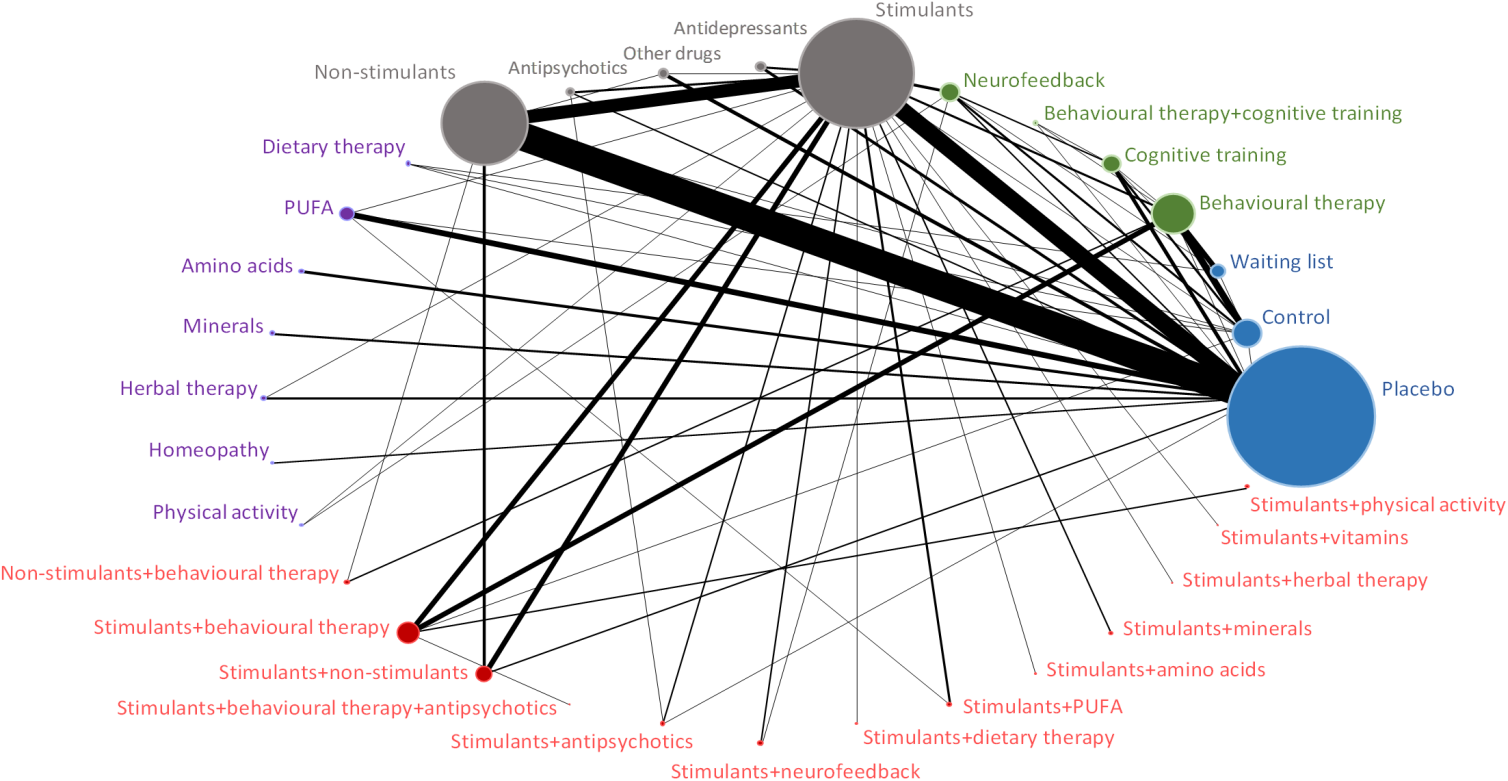
Evidence network diagram for primary outcome of acceptability among therapeutic classes. Solid lines represent direct comparisons within randomised trials. Nodes in blue represent controls. Nodes in green represent psychological interventions. Nodes in grey represent pharmacological interventions. Nodes in purple represent complementary and alternative medicine interventions. Nodes in red represent combined interventions. Size of node is proportional to number of randomised trials, and thickness of line connecting nodes is proportional to number of randomised trials directly comparing the two treatments. PUFA = polyunsaturated fatty acids.

**Fig 4:**
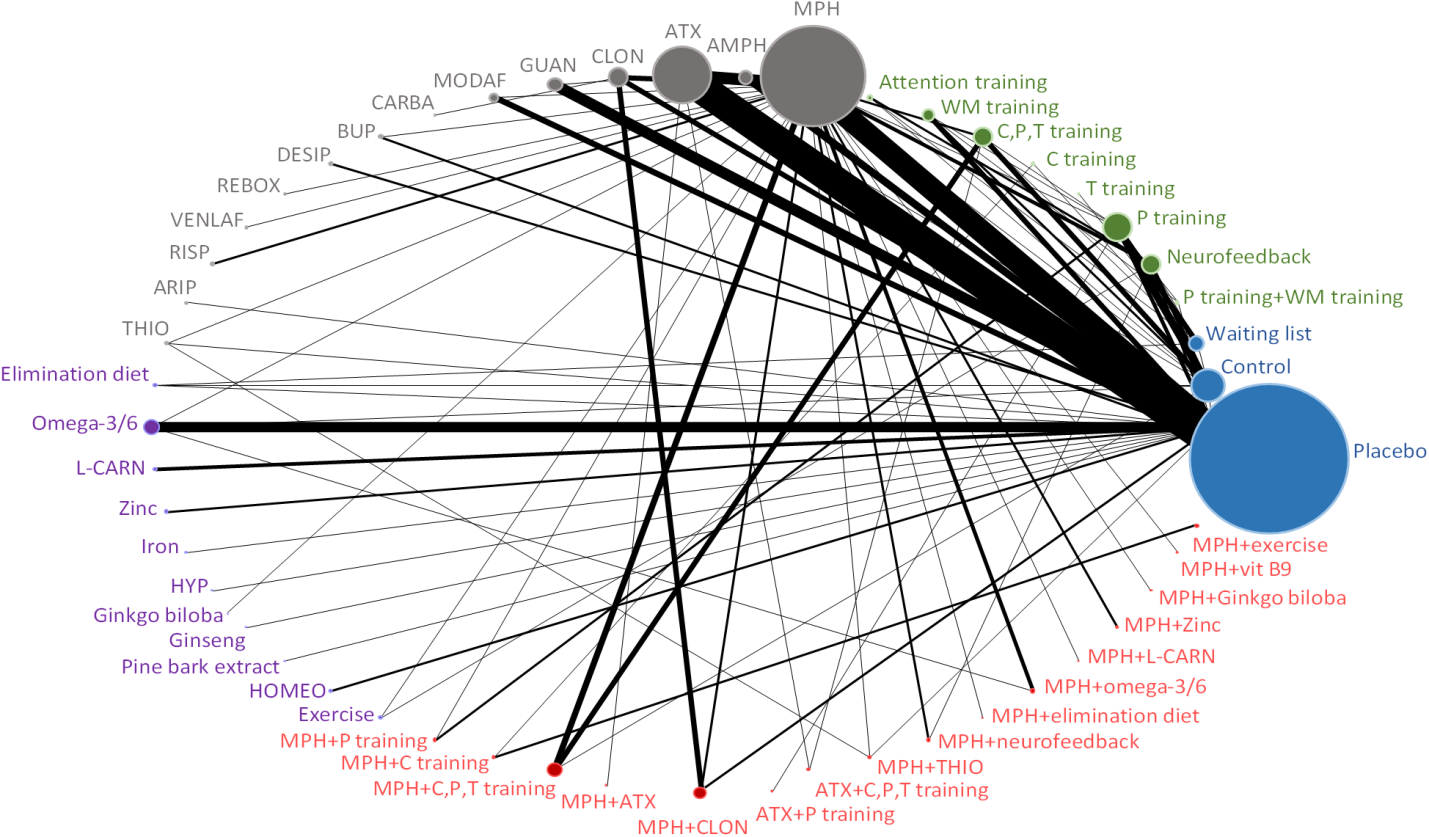
Evidence network diagram for primary outcome of acceptability among individual therapies. Solid lines represent direct comparisons within randomised trials. Nodes in blue represent controls. Nodes in green represent psychological interventions. Nodes in grey represent pharmacological interventions. Nodes in purple represent complementary and alternative medicine interventions. Nodes in red represent combined interventions. Size of node is proportional to number of randomised trials, and thickness of line connecting nodes is proportional to number of randomised trials directly comparing the two treatments. AMPH = amphetamine. ARIP = aripiprazole. ATX = atomoxetine. CLON = clonidine. GUAN = guanfacine. MODAF = modafinil. CARBA = carbamazepine. BUP = bupropion. DESIP = desipramine. REBOX = reboxetine. VENLAF = venlafaxine. RISP = risperidone. THIO = thioridazine. L-CARN = L-carnitine. HYP = hypericum. HOMEO = homeopathy. C, P, T training = child, parent and/or teacher training. P training = parent training. C training = child training. T training = teacher training. WM training = working memory training.

Figs 1–4 (and S2 Fig) presents the evidence networks of eligible treatment comparisons for the network meta-analyses. Placebo, stimulants (such as methylphenidate), non-stimulants (such as atomoxetine) and behavioural therapy were most often investigated (103, 77, 55, and 28 trials, respectively). The networks also included psychological interventions such as cognitive training and neurofeedback (20 trials), other pharmacological interventions (19 trials), and complementary and alternative medicine (26 trials in monotherapy and 12 trials in combination with stimulants).

### Findings from network meta-analysis

Summaries from all network meta-analyses are provided below for each outcome.

#### Findings for treatment response

**Fig 5:**
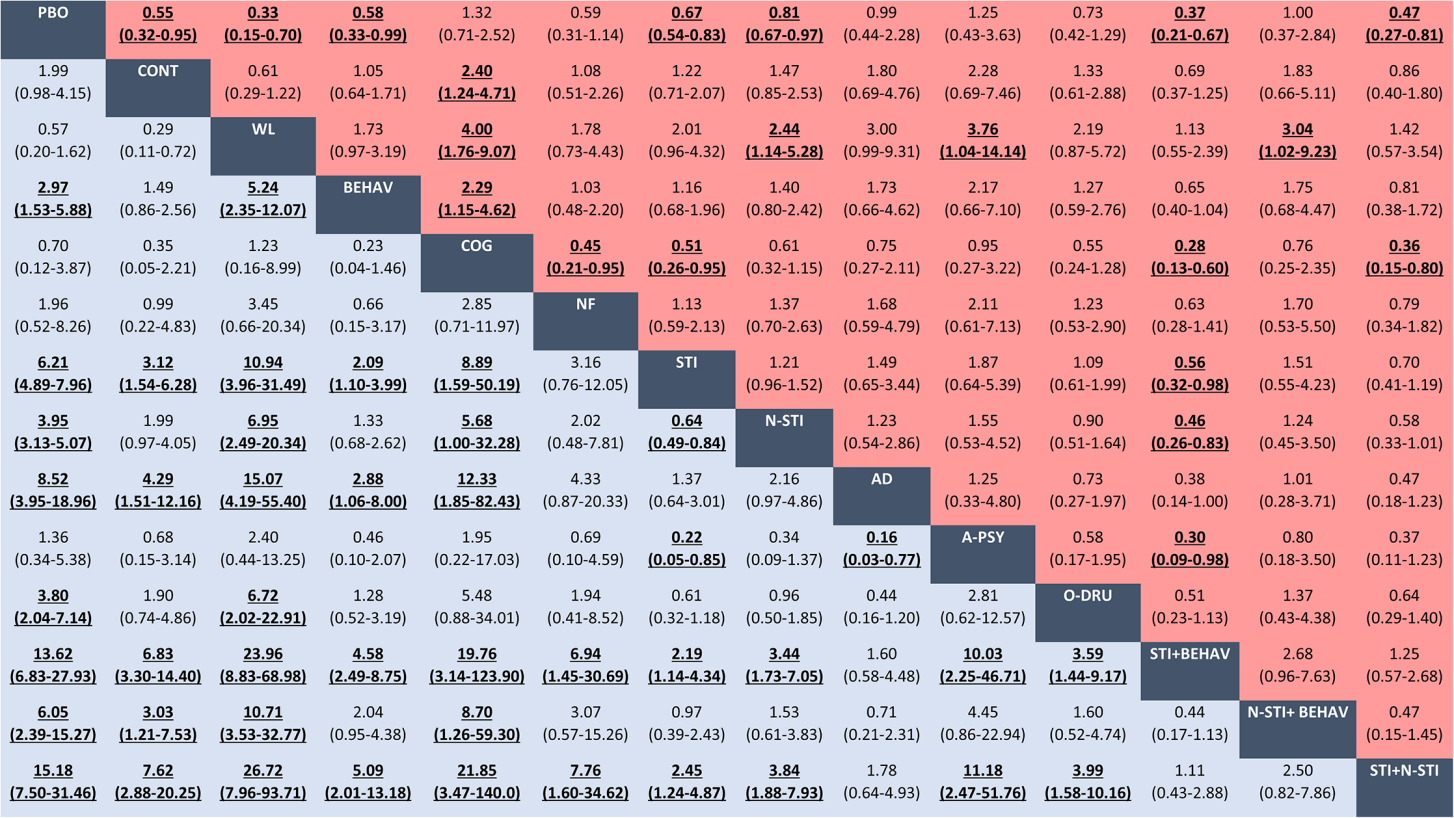
Network meta-analyses for efficacy and acceptability of classes of pharmacological and psychological interventions. Data in blue represents efficacy (treatment response). Data in red represents acceptability (all-cause discontinuation). Results are the ORs in the column-defining treatment compared with the ORs in the row-defining treatment. For efficacy (acceptability), ORs higher than 1 favour the row-defining treatment. For acceptability, ORs lower than 1 favour the row-defining treatment. Significant results are in bold and underscored. PBO = placebo. CONT = control. WL = waiting list. BEHAV = behavioural therapy. COGN = cognitive training. NF = neurofeedback. STI = stimulants. N-STI = non-stimulants. AD = antidepressants. A-PSY = antipsychotics. O-DRU = other unlicensed drugs. STI+BEHAV = stimulants+behavioural therapy. N-STI+BEHAV = non-stimulants+behavioural therapy. STI+N-STI = stimulants+non-stimulants. OR = Odds ratio. CI = credibility interval.

Treatment response (efficacy) was reported in 8,916 of 19,398 patients from a total of 113 trials evaluating 26 classes of interventions or control conditions. Low quality evidence suggests that behavioural therapy alone, stimulant monotherapy, and non-stimulant monotherapy seemed significantly more efficacious than placebo (Table 1 and Fig 5). Antidepressants seemed also associated with greater response compared with placebo, but the evidence was very limited; only 128 participants received antidepressants (Table 1). Stimulant monotherapy seemed superior to behavioural therapy alone, cognitive training and non-stimulant monotherapy. Behavioural therapy in combination with stimulants seemed also superior to placebo, cognitive training, neurofeedback, stimulant or non-stimulant monotherapy (Fig 5). The combination of stimulants and non-stimulants seemed significantly better than placebo, behavioural therapy alone, cognitive training, neurofeedback and monotherapy with stimulants or non-stimulants; however, only 480 participants received combined therapy with stimulants and non-stimulants across 4 trials and the results might be overestimated (very low quality of evidence).

**Table 1:** Network meta-analysis for efficacy. Summary of treatment effects compared with placebo.

	Trials	Events and participants	Class effect, OR (95% CrI)	Individual effect, OR (95% CrI)
**Controls**				
**Placebo**	65	929/4070	Reference	Reference
**Control (e.g., usual care)**	8	148/545	1.99 (0.98–4.15); Very low-quality	1.29 (0.65–2.59); Very low-quality
**Waiting list**	5	25/121	0.57 (0.20–1.62); Very low-quality	0.29 (0.10–0.85)^a^; Very low-quality
**Psychological interventions**				
**Behavioural therapy**	15	396/1016	2.97 (1.53–5.88)^a^; Low-quality	-
Parent training	8	128/357	-	1.19 (0.50–2.77); Very low-quality
Child, parent and/or teacher training	8	254/535	-	2.73 (1.41–5.39)^a^; Low-quality
**Cognitive training**	2	7/65	0.70 (0.12–3.87); Very low-quality	-
Working memory training	1	1/27	-	0.34 (0.01–5.82); Very low-quality
Attention training	1	6/38	-	4.17x10^12^ (1.63–12.44x10^28^)^b^; Very low-quality
**Other psychotherapies**	1	10/32	7.02 (1.56–32.59)^a^; Very low-quality	-
**Neurofeedback**	4	26/110	1.96 (0.52–8.26); Very low-quality	-
Neurofeedback/theta-beta training	2	23/86	-	1.61x10^13^ (4.57–23.91x10^28^)^b^; Very low-quality
**Pharmacological interventions**				
**Stimulants**	53	3464/5831	6.21 (4.89–7.96)^a^; Low-quality	-
Methylphenidate	40	2279/3836	-	5.26 (4.09–6.82)^a^; Low-quality
Amphetamine	9	1017/1628	-	7.45 (5.10–11.09)^a^; Low-quality
**Non-stimulants**	40	2447/4741	3.95 (3.13–5.07)^a^; Low-quality	-
Atomoxetine	27	1547/3162	-	3.63 (2.81–4.73)^a^; Low-quality
Guanfacine	10	837/1461	-	3.29 (2.27–4.82)^a^; Low-quality
Clonidine	4	63/118	-	3.96 (1.89–8.41)^a^; Very low-quality
**Antidepressants**	6	69/128	8.52 (3.95–18.96)^a^; Very low-quality	-
Bupropion	1	8/22	-	2.41 (0.48–11.63); Very-low quality
Desipramine	2	36/58	-	36.76 (9.17–214.0)^a^; Very-low quality
Venlafaxine	1	12/19	-	4.07 (0.73–22.36); Very-low quality
Reboxetine	1	5/17	-	3.58 (0.57–22.11); Very-low quality
**Antipsychotics**	1	8/41	1.36 (0.34–5.38); Very low-quality	-
Thioridazine	1	8/41	-	1.04 (0.28–3.78); Very-low quality
**Other unlicensed drugs**	6	248/572	3.80 (2.04–7.14)^a^; Very low-quality	-
Modafinil	5	245/547	-	5.51 (3.04–10.32)^a^; Very-low quality
Carbamazepine	1	3/25	-	0.18 (0.02–1.20); Very-low quality
**Complementary and alternative medicine interventions**				
**Dietary therapy**	2	43/65	2.07x10^7^ (0.46x10^3^-13.01x10^21^)^b^; Very low-quality	-
**Polyunsaturated fatty acids (or PUFAs)**	3	41/124	2.14 (0.83–5.57); Very low-quality	-
Omega-3 and -3/6 fatty acids	3	41/124	-	1.99 (0.85–4.82); Very-low quality
**Amino acids**	1	9/58	1.19 (0.25–5.71); Very low-quality	-
L-carnitine	1	9/58	-	1.20 (0.29–5.22); Very-low quality
**Minerals**	2	49/220	2.93 (0.90–10.15); Very low-quality	-
Zinc	1	45/202	-	2.42 (0.80–7.67); Very-low quality
Iron	1	4/18	-	2.71x10^10^ (3.34–9.19x10^23^)^b^; Very low-quality
**Herbal therapy**	2	13/52	0.59 (0.17–1.99); Very low-quality	-
St. John’s wort (*Hypericum perforatum*)	1	11/27	-	1.00 (0.23–4.26); Very-low quality
Ginkgo biloba	1	2/25	-	0.21 (0.02–1.35); Very low-quality
**Combined interventions**				
**Stimulants plus behavioural therapy**	8	283/521	13.62 (6.83–27.93)^a^; Very low-quality	-
Methylphenidate plus parent training	1	1/4	-	55.63 (3.18–29.52x10^2^)^a^; Very low-quality
Methylphenidate plus child, parent and/or teacher training	5	187/282	-	15.82 (8.06–32.80)^a^; Very-low quality
**Stimulants plus non-stimulants**	4	313/480	15.18 (7.50–31.46)^a^; Very low-quality	-
Methylphenidate plus clonidine	1	29/33	-	21.91 (5.52–105.4)^a^^,^^b^; Very-low quality
**Non-stimulants plus behavioural therapy**	4	86/163	6.05 (2.39–15.27)^a^; Very low-quality	-
Atomoxetine plus parent training	1	18/50	-	2.48 (0.51–11.79); Very-low quality
Atomoxetine plus child, parent and/or teacher training	3	68/113	-	5.53 (2.19–14.06)^a^; Very-low quality
**Stimulants plus antipsychotics plus behavioural therapy**	1	63/84	18.19 (4.10–83.33)^a^^,^^b^; Very low-quality	-
**Stimulants plus antipsychotics**	2	33/54	10.32 (3.49–32.11)^a^^,^^b^; Very low-quality	-
Methylphenidate plus thioridazine	1	8/41	-	5.97 (1.90–19.59)^a^^,^^b^; Very-low quality
**Stimulants plus other psychotherapies**	1	31/59	15.42 (3.38–70.31) ^a^^,^^b^; Very low-quality	-
**Stimulants plus polyunsaturated fatty acids (or PUFAs)**	2	47/50	16.81 (3.45–101.6) ^a^^,^^b^; Very low-quality	-
Methylphenidate plus omega-3/6 fatty acids	2	47/50	-	15.68 (3.43–87.0)^a^^,^^b^; Very-low quality
**Stimulants plus minerals**	1	11/20	18.67 (3.15–116.0)^a^^,^^b^; Very low-quality	-
Methylphenidate plus zinc	1	11/20	-	15.73 (2.94–85.29)^a^^,^^b^; Very low-quality
**Stimulants plus herbal therapy**	1	26/33	10.15 (1.97–54.8)^a^^,^^b^; Very low-quality	-
Methylphenidate plus ginkgo biloba	1	26/33	-	8.65 (1.94–40.79)^a^^,^^b^; Very low-quality

Results are odds ratios (OR) with 95% credible Intervals and quality of evidence. An OR < 1 favours placebo (that is, more response events occur with placebo that with other intervention).

^a^p < 0.05.

^b^Extremely wide credible intervals owing to small patient and trial numbers and frequent events among treatment arm.

#### Findings for treatment acceptability

The acceptability outcome (all-cause discontinuation) was observed in a total of 4,822 of 22,961 patients from a total of 171 trials evaluating 32 classes of interventions or control conditions. Low to very low quality evidence suggests that behavioural therapy alone and stimulant monotherapy showed the best profile of acceptability, leading to significantly reduced chance of discontinuation compared to placebo (Table 2 and Fig 5). Non-stimulant monotherapy or in combination with stimulants seemed better accepted than placebo (very low quality of evidence). Behavioural therapy in combination with stimulants seemed also superior to placebo, cognitive training, stimulant or non-stimulant monotherapy (very low quality of evidence) (Fig 5).

**Table 2:** Network meta-analysis for acceptability. Summary of treatment effects compared with placebo.

	Trials	Events and participants	Class effect, OR (95% CrI)	Individual effect, OR (95% CrI)
**Controls**				
**Placebo**	92	1406/5202	Reference	Reference
**Control (e.g., usual care)**	17	79/819	0.55 (0.32–0.95)^a^; Very low-quality	0.55 (0.32–0.98)^a^; Very low-quality
**Waiting list**	10	28/290	0.33 (0.15–0.70)^a^; Very low-quality	0.30 (0.13–0.69)^a^; Very low-quality
**Psychological interventions**				
**Behavioural therapy**	25	224/1385	0.58 (0.33–0.99)^a^; Very low-quality	-
Parent training	16	124/742	-	0.69 (0.35–1.36); Very low-quality
Child, parent and/or teacher training	9	54/569	-	0.37 (0.17–0.80)^a^; Low-quality
Child training	2	48/205	-	0.25 (0.04–1.98); Very low-quality
Teacher training	1	4/28	-	0.76 (0.08–8.49); Very low-quality
**Cognitive training**	10	49/339	1.32 (0.71–2.52); Very low-quality	-
Working memory training	6	27/198	-	1.75 (0.75–4.14); Very low-quality
Attention training	3	19/126	-	0.65 (0.22–1.92); Very low-quality
**Behavioural therapy with cognitive training**	1	3/26	3.39 (0.60–19.58); Very low-quality	4.08 (0.69–24.66); Very low-quality
**Neurofeedback**	10	38/271	0.59 (0.31–1.14); Very low-quality	-
Neurofeedback/theta-beta training	9	34/225	-	0.40 (0.19–0.82)^a^; Very low-quality
**Pharmacological interventions**				
**Stimulants**	65	847/4778	0.67 (0.54–0.83)^a^; Low-quality	-
Methylphenidate	55	585/3196	-	0.59 (0.46–0.75)^a^; Low-quality
Amphetamine	8	255/1433	-	0.78 (0.52–1.18); Very low-quality
**Non-stimulants**	52	1420/5817	0.81 (0.67–0.97)^a^; Very low-quality	-
Atomoxetine	37	857/3844	-	0.85 (0.68–1.07); Very low-quality
Guanfacine	9	439/1449	-	0.79 (0.54–1.14); Very low-quality
Clonidine	6	71/298	-	0.40 (0.20–0.78)^a^; Very low-quality
**Antidepressants**	7	23/208	0.99 (0.44–2.28); Very low-quality	-
Bupropion	3	11/114	-	1.54 (0.39–6.76); Very low-quality
Desipramine	2	6/58	-	0.70 (0.17–2.89); Very low -quality
Venlafaxine	1	1/19	-	0.64 (0.02–19.78); Very low-quality
Reboxetine	1	5/17	-	0.73 (0.12–4.44); Very low-quality
**Antipsychotics**	4	11/108	1.25 (0.43–3.63); Very low-quality	-
Risperidone	2	5/42	-	0.56 (0.13–2.77); Very low-quality
Thioridazine	1	5/41	-	3.01 (0.57–17.81); Very low-quality
Aripiprazole	1	1/25	-	0.61 (0.02–25.34); Very low-quality
**Other unlicensed drugs**	7	133/585	0.73 (0.42–1.29); Very low-quality	-
Modafinil	6	127/560	-	0.67 (0.37–1.24); Very low-quality
Carbamazepine	1	6/25	-	0.69 (0.11–4.27); Very low-quality
**Complementary and alternative medicine interventions**				
**Dietary therapy**	3	13/85	0.79 (0.23–2.76); Very low-quality	-
Elimination diet	3	13/85	-	0.77 (0.23–2.64); Very low-quality
**Polyunsaturated fatty acids (or PUFAs)**	9	92/442	1.09 (0.68–1.74); Very low-quality	-
Omega-3 and -3/6 fatty acids	9	92/442	-	1.06 (0.66–1.71); Very low-quality
**Amino acids**	4	20/121	0.59 (0.24–1.44); Very low-quality	-
L-carnitine	3	19/101	-	0.66 (0.25–1.75); Very low-quality
**Minerals**	3	111/248	1.14 (0.49–2.72); Very low-quality	-
Zinc	2	109/230	-	1.05 (0.42–2.62); Very low-quality
Iron	1	2/18	-	26.79 (0.19–1.03x10^6^)^b^; Very low-quality
**Herbal therapy**	4	6/131	0.53 (0.15–1.88); Very low-quality	-
St. John's wort (*Hypericum perforatum*)	1	1/27	-	0.41 (0.01–6.11); Very low-quality
Ginkgo biloba	1	2/25	-	0.57 (0.05–6.67); Very low-quality
Ginseng	1	0/35	-	5.31x10^-4^ (5.70x10^-12^–0.69)^b^; Very low-quality
Pine bark (extract)	1	3/44	-	1.33 (0.12–44.24); Very low-quality
**Homeopathy**	2	10/52	0.56 (0.18–1.73); Very low-quality	0.56 (0.17–1.77); Very low-quality
**Physical activity (exercise)**	1	3/37	0.44 (0.07–2.19); Very low-quality	0.48 (0.07–2.75); Very low-quality
**Combined interventions**				
**Stimulants plus behavioural therapy**	13	109/699	0.37 (0.21–0.67)^a^; Low-quality	-
Methylphenidate plus parent training	2	19/68	-	0.50 (0.18–1.44); Very low-quality
Methylphenidate plus child training	3	37/185	-	0.18 (0.02–1.74); Very low -quality
Methylphenidate plus child, parent and/or teacher training	7	38/362	-	0.24 (0.12–0.50)^a^; Vey low-quality
**Stimulants plus non-stimulants**	7	85/529	0.47 (0.27–0.81)^a^; Low-quality	-
Methylphenidate plus atomoxetine	1	1/9	-	1.00 (0.03–48.87); Very low-quality
Methylphenidate plus clonidine	3	12/73	-	0.32 (0.13–0.77)^a^; Very low-quality
**Non-stimulants plus behavioural therapy**	3	22/114	1.00 (0.37–2.84); Very low-quality	-
Atomoxetine plus parent training	1	14/50	-	0.87 (0.21–3.74); Very low-quality
Atomoxetine plus child, parent and/or teacher training	2	8/64	-	1.23 (0.29–6.03); Very low-quality
**Stimulants plus antipsychotics plus behavioural therapy**	1	23/84	0.65 (0.19–2.37); Very low-quality	-
**Stimulants plus antipsychotics**	2	4/54	0.99 (0.21–4.19); Very low-quality	-
Methylphenidate plus thioridazine	1	3/42	-	1.61 (0.25–10.33); Very low-quality
**Stimulants plus neurofeedback**	2	12/76	0.37 (0.13–1.05); Very low-quality	-
Methylphenidate plus neurofeedback	2	12/76	-	0.30 (0.10–0.89)^a^; Very low-quality
**Stimulants plus dietary therapy**	1	10/53	0.59 (0.16–2.21); Very low-quality	-
Methylphenidate plus elimination diet	1	10/53	-	0.52 (0.13–2.00); Very low-quality
**Stimulants plus polyunsaturated fatty acids (or PUFAs)**	3	9/111	0.34 (0.12–0.94)^a^; Very low-quality	-
Methylphenidate plus omega-3/6 fatty acids	3	9/111	-	0.31 (0.11–0.84)^a^; Very low-quality
**Stimulants plus amino acids**	1	1/20	0.65 (0.02–34.56); Very low-quality	-
Methylphenidate plus L-carnitine	1	1/20	-	0.50 (0.01–17.26); Very low-quality
**Stimulants plus minerals**	2	3/42	0.36 (0.05–1.97); Very low-quality	-
Methylphenidate plus zinc	2	3/42	-	0.31 (0.05–1.68); Very low-quality
**Stimulants plus herbal therapy**	1	2/33	0.28 (0.03–2.08); Very low-quality	-
Methylphenidate plus ginkgo biloba	1	2/33	-	0.23 (0.02–1.75); Very low-quality
**Stimulants plus vitamins**	1	15/23	1.49 (0.35–6.67); Very low-quality	-
Methylphenidate plus vitamin B9	1	15/23	-	1.32 (0.30–5.94); Very low-quality
**Stimulants plus physical activity**	2	5/33	0.50 (0.09–2.76); Very low-quality	-
Methylphenidate plus exercise	2	5/33	-	0.25 (0.02–3.93); Very low-quality

Results are odds ratios (OR) with 95% credible Intervals and quality of evidence. An OR > 1 favours placebo (that is, fewer events occur with placebo that with other intervention).

^a^p < 0.05.

^b^Extremely wide credible intervals owing to small patient and trial numbers and rare events among treatment arm.

Cognitive training, neurofeedback, antipsychotics and complementary and alternative medicine (such as dietary therapy, polyunsaturated fatty acids, amino acids, minerals, herbal therapy, homeopathy, and physical activity) did not have greater effect on the primary outcomes of efficacy and acceptability compared with placebo; the effects of these interventions were not significant or were very imprecise (Table 2 and Table 3 and S6 Table, S7 Table).

**Table 3:** Network meta-analysis for efficacy and safety of commonly prescribed medications compared with methylphenidate.

Pharmacological treatments	Efficacy (response)	Acceptability (all-cause discontinuation)	Tolerability (discontinuation due to adverse events)	Serious adverse events	Decreased weight gain	Anorexia	Insomnia	Sleep disturbances (unspecified)	Anxiety
**Methylphenidate**	Reference	Reference	Reference	Reference	Reference	Reference	Reference	Reference	Reference
**Amphetamine**	1.42 (0.92–2.20); Very low-quality	1.33 (0.85–2.08); Very low-quality	1.76 (0.70–4.39); Very low-quality	1.15 (0.20–6.72); Very low-quality	3.37 (1.62–7.02)^a^; Very low-quality	1.75 (0.95–3.31); Very low-quality	1.79 (1.17–2.87)^a^; Low-quality	0.41 (0.01–21.06); Very low-quality	0.42 (0.02–5.72); Very low-quality
**Atomoxetine**	0.69 (0.52–0.92)^a^; Low-quality	1.45 (1.09–1.91)^a^; Low-quality	1.33 (0.73–2.40); Very low-quality	1.15 (0.40–3.50); Very low-quality	0.71 (0.42–1.24); Very low-quality	0.60 (0.42–0.86)^a^; Low-quality	0.41 (0.30–0.58)^a^; Low-quality	0.54 (0.03–2.19); Very low-quality	0.52 (0.18–2.00); Very low-quality
**Clonidine**	0.75 (0.36–1.58); Very low-quality	0.68 (0.33–1.35); Very low-quality	3.77 (0.52–32.98); Very-low quality	-	-	0.90 (0.28–2.82); Very low-quality	1.20 (0.43–3.95); Very low-quality	0.21 (0.02–1.05); Very low-quality	-
**Guanfacine**	0.62 (0.40–0.98)^a^; Low-quality	1.34 (0.86–2.07); Very low-quality	2.30 (0.87–6.16); Very low-quality	1.45 (0.35–7.20); Very low-quality	-	0.26 (0.12–0.57)^a^; Low-quality	0.68 (0.37–1.31); Very low-quality	-	0.70 (0.14–4.36); Very low-quality
**Modafinil**	1.05 (0.56–2.00); Very low-quality	1.14 (0.61–2.19); Very low-quality	0.86 (0.21–3.75); Very low-quality	-	0.36 (0.10–1.44); Very low-quality	0.48 (0.20–1.14); Very low-quality	2.47 (1.16–5.90)^a^; Low-quality	0.20 (0.03–1.12); Very low-quality	0.46 (0.08–2.38); Very low-quality
**Bupropion**	0.46 (0.09–2.21); Very low-quality	2.60 (0.66–11.64); Very low-quality	-	-	-	0.68 (0.12–3.68); Very low-quality	0.53 (0.13–1.94); Very-low quality	-	0.50 (0.05–4.01); Very low-quality

Results are odds ratios (OR) with 95% credible Intervals and quality of evidence. For efficacy, an OR < 1 favours methylphenidate (that is, more response events occur with methylphenidate). For acceptability, tolerability and specific adverse events, an OR > 1 favours methylphenidate (that is, fewer events occur with methylphenidate).

^a^p < 0.05.

#### Findings for comparisons amongst commonly prescribed medications

**Fig 6:**
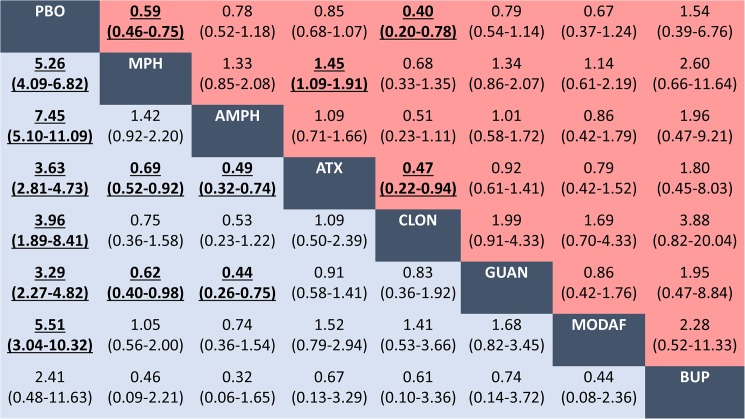
Network meta-analyses for efficacy and acceptability of commonly prescribed medications for ADHD. Data in blue represents efficacy (treatment response). Data in red represents acceptability (all-cause discontinuation). Results are the ORs in the column-defining treatment compared with the ORs in the row-defining treatment. For efficacy (acceptability), ORs higher than 1 favour the row-defining treatment. For acceptability, ORs lower than 1 favour the row-defining treatment. Significant results are in bold and underscored. PBO = placebo. MPH = methylphenidate. AMPH = amphetamine. ATX = atomoxetine. CLON = clonidine. GUAN = guanfacine. MODAF = modafinil. BUP = bupropion. OR = Odds ratio. CI = credibility interval.

Results from network meta-analyses performed at the agent level for commonly prescribed medications are presented in Table 3 and Fig 6. Methylphenidate, amphetamine, atomoxetine, guanfacine, clonidine and modafinil seemed significantly more efficacious than placebo (though we have less confidence in the effects of clonidine and modafinil based upon GRADE ratings). Methylphenidate and amphetamine seemed more efficacious than atomoxetine and guanfacine. In terms of acceptability, methylphenidate and clonidine seemed superior to placebo, whereas methylphenidate and clonidine seemed better accepted than atomoxetine (even though limited evidence for clonidine based on numbers of studies and patients available).

#### Findings for secondary outcomes

Estimated effects for the secondary outcomes of tolerability (discontinuations due to adverse events), serious adverse events and specific adverse events are presented in S8 Table.

Discontinuations due to adverse events were reported in 740 of 18,863 patients from a total of 105 trials. Very low quality evidence suggests that stimulants, non-stimulants and the combination of stimulants and non-stimulants were each well tolerated compared to placebo (OR 2.38, 95% CrI 1.45–3.99; 3.11, 1.99–5.08; and 4.54, 1.10–20.41, respectively).

Stimulants (OR 8.01, 95% CrI 5.75–11.34), non-stimulants (OR 4.70, 3.41–6.62), combinations of stimulants and non-stimulants (OR 6.25, 1.97–19.11), antidepressants (OR 4.01, 1.63–10.17) and other unlicensed drugs (OR 3.64, 1.59–8.67) produced anorexia (2,440 events in 15,856 patients from 81 trials); whereas stimulants (OR 21.64, 11.92–42.28), non-stimulants (OR 10.31, 6.13–18.31) and other unlicensed drugs (OR 7.08, 1.71–31.97) led to weight loss (710 events in 6,453 patients from 34 trials).

Stimulants led to unspecified sleep disturbances (OR 6.02, 95% CrI 2.81–14.45; 371 events in 2,125 patients from 22 trials), whereas regimens containing either stimulants (OR 3.99, 2.90–5.55), non-stimulants (OR 1.55, 1.12–2.19) or both (OR 3.81, 1.70–8.23), and other unlicensed drugs (OR 7.18, 3.20–18.27) caused insomnia (1,068 events in 11,722 patients from 49 trials).

Treatment estimates for serious adverse events (96 events in 9,212 patients from 32 trials) and anxiety (256 events in 2,961 patients from 21 trials) were non-significant or were highly imprecise. Given the limited number of trials reporting data on syncope, this outcome was not evaluated in the network meta-analyses.

Table 3 presents safety of commonly prescribed medications compared with methylphenidate. Amphetamine was associated with an increased the risk of weight loss compared with methylphenidate. In terms of anorexia, methylphenidate was worse than atomoxetine and guanfacine. For insomnia, methylphenidate was better than amphetamine but worse than atomoxetine.

In terms of cardiovascular events, a limited number of trials reported similar outcome definitions on vital signs for different pharmacological interventions. For example, some studies reported the following: an increase in blood pressure (183 events, 3034 patients and 13 trials); a decrease in blood pressure (77 events, 1675 patients and 7 trials); an increase in heart rate (242 events, 5258 patients and 25 trials); a decrease in heart rate (87 events, 1972 patients and 9 trials); palpitations (23 events, 1366 patients, and 8 trials); and QT prolongation (64 events, 2234 patients, and 8 trials). Given this wide variability of reporting of cardiovascular events, network meta-analysis for these specific events was considered not feasible.

#### Additional analyses

The full details of the additional analyses including sensitivity analyses, subgroup analyses, model fit evaluations and small study effect bias assessments are reported in the report S8 Table. Overall, it was noted that the results were not substantially different when we examined the effects of the distribution of potential effect modifiers by including covariates in network meta-regression models. We excluded studies with high risk of bias and small studies, but analyses may have led to insufficient statistical power for different treatment comparisons. Primary analyses were influenced by short-term trials, with very limited information on long-term effects in a subgroup analysis examining duration of treatment. No differences in conclusions were observed in sensitivity analyses including the dosage and type of formulations of stimulant medications. There was generally a better trade-off between model fit and complexity when consistency was assumed. Measure of model fit suggested the presence of potential significant inconsistency in a small number of cases: for acceptability (consistency model DIC = 1855.73 versus inconsistency model DIC = 1881.88) and for tolerability (842.87 versus 851.00, respectively) (S9 Table), limiting the conclusiveness of findings for these endpoints. Data were double-checked, and in both cases we could not identify any important covariate that differed across comparisons. Small study effect bias was quantitatively assessed, but inadequate numbers of included trials with direct comparisons prevented us from drawing firm conclusions from funnel plots and statistical tests (S10 Table, S3 Fig). Results on the primary outcomes for the frequentist pairwise meta-analysis comparisons (for both the class effects as well as the individual interventions) are provided in online supplement (see S10 Table). Overall, these analyses suggested no conclusive evidence in direct estimates of treatment effects when comparing therapies using placebo as the reference comparator.

## Discussion

ADHD is one of the most commonly diagnosed and treated psychiatric disorders in childhood. In many Western countries, the prescribing rates have increased dramatically, with wide variations in medical practice [53–56] that have led to remarkable controversies and public debate around the clinical (and social) management of ADHD [57–60]. Our analysis of 190 studies that enrolled 26,114 participants randomly assigned to 52 different interventions (grouped in 32 therapeutic classes) has evaluated the comparative effects of multiple pharmacological and non-pharmacological treatments used in children and adolescents with ADHD.

Our main findings have potential implications for informing evidence-based treatment selection, but also establishing recommendations in clinical practice guidelines as well as the future design of new studies. Some treatments for the management of ADHD differed in both clinical and statistical aspects. In terms of response, behavioural therapy (alone or in combination with stimulants), stimulants and non-stimulants seemed significantly more efficacious than placebo; stimulants seemed superior to behavioural therapy, cognitive training and non-stimulants; and behavioural therapy in combination with stimulants seemed superior to monotherapy with stimulants or non-stimulants. Among single drug agents, methylphenidate, amphetamine, atomoxetine, guanfacine, clonidine and modafinil seemed significantly more efficacious than placebo (even though less clear confidence with clonidine and modafinil). Methylphenidate and amphetamine seemed more efficacious than atomoxetine and guanfacine. In terms of acceptability, behavioural therapy and stimulants (and their combination), showed the best profile of acceptability leading to significantly fewer discontinuations that did placebo. Methylphenidate and clonidine seemed superior to placebo, whereas methylphenidate and clonidine seemed better accepted than atomoxetine (even though limited evidence for clonidine). Most of the efficacious pharmacological treatments (particularly, stimulants) were associated with anorexia, weight loss and insomnia.

One of the most important clinical implications is that behavioural therapy, particularly given by parents and with active child and teacher involvement, is the only non-pharmacological intervention that was found to be associated with statistically significant benefits in our analyses. Cognitive training, neurofeedback, dietary therapy (such as restricted elimination diet), polyunsaturated fatty acids, amino acids, minerals, herbal therapy, homeopathy, and physical activity cannot be recommended as evidence-based interventions for global functioning and core ADHD symptoms until better evidence of their comparative efficacy is reported in well-designed and conducted clinical trials. However, a healthy, balanced diet and regular exercise should be emphasised for all children and adolescents, including those with ADHD [7]. Stimulants such as methylphenidate and amphetamine may represent the best options amongst the pharmacological interventions for ADHD, and non-stimulants such as atomoxetine, guanfacine and (less studied) clonidine may be considered secondary treatment options, respectively. Other unlicensed medications have gained popularity in the mental health treatment of young people (such as modafinil, antidepressants such as bupropion and antipsychotic agents), but the evidence for these therapies (not indicated in ADHD) is currently very limited. Similarly, concerns have been raised about the benefit-risk profile of the use in ADHD of these agents outside of approved indications [40,61–64].

Our systematic review suggests that treatment effects are larger when behavioural therapy is combined with stimulants (with very low quality of evidence). The clinical decision to combine these interventions should be driven by the symptoms presented, the needs of the children and their family and the availability of healthcare services. The value of combined treatment for ADHD is recognized as an area of interest because it might lead to potential beneficial effects in different domains [65]. Combining behavioural therapy with stimulants may enhance attentional processes and reduce impulsive responding, and may be a way of reducing the dosage and duration of pharmacological treatment, and thus addressing concerns about the potential harms associated with the use of pharmacotherapy [9,65]. The Multimodal Treatment for children with ADHD (MTA) study, a randomised trial that compared stimulant methylphenidate, intensive behavioural therapy, and combination of both methylphenidate with intensive behavioural therapy with a community control, is considered the largest (n = 579 participants) and perhaps the most methodologically sound investigation of combination treatment for ADHD [19,66]. In line with our findings, results of the MTA study suggested a benefit from combination treatment over stimulant monotherapy when categorical measures of excellent response were used [67], but the effects were considered small in magnitude and subsequent trials of smaller sample size reported conflicting results [68–71]. Our finding that combination of stimulants and non-stimulants treatment (such as methylphenidate plus clonidine, atomoxetine, or guanfacine) appears to be effective for ADHD may challenge some guidance on the management of ADHD, as commonly no clear recommendations are made for (or against) combining these two pharmacological treatments. In our opinion, concerns about widespread adoption of this therapeutic strategy could be justified despite the potential benefits of combination drug regimens, because available information on the balance between potential benefits and safety are incompletely characterized in the biomedical literature [72–77].

There are multiple and diverse clinical practice guidelines worldwide for the management of ADHD [9–11,40,78–80], such as those developed by the National Institute for Health and Care Excellence (NICE) [9] and by the Scottish Intercollegiate Guidelines Network (SIGN) in the UK [78], and by the American Academy of Pediatrics (AAP) [79] and the American Academy of Child and Adolescent Psychiatry (AACAP) [80] in the US. Some of the most important differences between them are that US guidelines recommend the use of pharmacological treatment for children with mild ADHD or pre-school children; conversely, the UK guidelines state that pharmacological treatment should be reserved for those ADHD children with severe symptoms and impairment, or moderate levels of impairment who have refused non-pharmacological interventions, or whose symptoms have not responded sufficiently to behavioural interventions. Generally, some of these guidelines recognise that if pharmacological treatment is prescribed, it should be provided in conjunction with behavioural therapy. Thus, considering the overall benefit-risk balance of the multiple interventions, the interpretation of our findings may support a stepwise approach following UK recommendations, which suggests that increased access to behavioural therapy and pharmacological treatment (as indicated) would improve symptomatic and functional outcomes for children and adolescents with ADHD.

Along with broad recognition that data from some clinical trials and treatment comparisons were sparse, and that more and higher-quality data could improve estimations and recommendations, aspects of the general approach of previous systematic reviews and meta-analyses [12–17,81–83] were challenged. For example, two recent Cochrane systematic reviews have suggested that stimulants such as amphetamines [12] and methylphenidate [13] may improve ADHD core symptoms compared to placebo (or no intervention) in children and adolescents; but overall quality of the evidence ranged from low to very low on most outcomes. However, no network meta-analyses to compare the relative effects of all existing interventions have been performed to address this knowledge gap. In contrast to the traditional meta-analysis methods which focus on reducing the evaluation to a single pair-wise comparison, network meta-analyses allow multiple intervention comparisons to be made. The strengths of this review also include the completeness of the search including multiple databases and trial registries, forward searching of unpublished data and hand searching of the grey literature, risk of bias assessment and reporting of confidence in treatment effects when interpreting each therapy comparison. We also used an a priori published protocol and followed established reporting guidelines [29,30].

There are some limitations to be noted regarding our study. We used established methods [32–34] to leverage a large number of existing systematic reviews and meta-analyses, to identify relevant studies. Although we believe this technique has performed well, our review may have missed studies due to differences in the eligibility criteria and coverage of the retrieved reviews. Similar to other reviews and meta-analyses [12–17,81–83], there is methodological and clinical heterogeneity in the included trials in terms of trial design, patient populations, and outcome measurement. Caution should be used when interpreting findings, considering the fact that not all treatments (particularly, non-pharmacological treatments) are represented in the trials that formed the evidence base. The primary outcomes of efficacy and acceptability were the most commonly and consistently reported outcomes across the network of trials. However, as we did not control the risks of type I errors in meta-analyses of primary outcomes, the presence of potential false positive findings cannot be excluded. As with individual clinical trials and pairwise meta-analyses, secondary outcomes and additional analyses (e.g., sensitivity analyses and subgroups analyses) should be regarded as exploratory and multiple testing [84,85] issues need to be considered when results are interpreted. Our review does not provide evidence that supports any stimulant over another, and does not reveal any significant differences between short-acting and long-acting formulations. Insufficient patient numbers and events to form well connected networks limited the analyses that could be conducted to account for dosage and intensity of non-stimulants and non-pharmacological treatments in the absence of more studies.

Although we adopted reproducible definitions, the heterogeneous reporting of events in studies posed a major challenge. None of the trials examined and reported all the outcomes of interest. The overall quality of the network meta-analyses evidence ranged from low to very low as assessed by the GRADE approach (the quality or confidence of evidence ratings were rated down by serious concerns of risk of bias in the randomised trials, imprecision of treatment effect estimates, and potential inconsistency) (see S7 Table). In fact, results on the primary outcomes for both the network meta-analyses and pairwise meta-analyses include small number of studies relative to the number of comparisons considered, resulting in low confidence in estimates for many key analyses and comparisons. Therefore, further high-quality trials are likely to have an important impact on the confidence of treatment effects estimates and may change the interpretation of our findings. At the study level, many trials appear to have several reporting or methodological issues, and an additional limitation was the very small sample sizes. Many trials had a high risk of bias (53%) or unclear risk of bias (45%) for at least one criterion, and most trials were funded by industry (50% of all trials; 77% of trials evaluating pharmacological treatments). Inclusion of funding source as a standard item for rating sponsorship bias and conflicts of interest [13,86] could make the risk of bias even more apparent than that reported in our review. In our opinion, these issues require further pragmatic action at the levels of planning, funding, conduct, and reporting of randomised trials [85,87–90]. Preexisting therapies, the settings in which psychological interventions were undertaken, how they were carried out, and potential factors responsible for response or failure are not always clearly reported in many ADHD trials and presents a challenge to treatment classification and knowledge translation. To the extent possible, we pursued sensitivity analyses including study-level covariates in network meta-regression models to infer about transitivity/consistency and to establish the robustness of our findings. Overall, we show that our results are robust to a variety of estimation procedures, but these analyses may be underpowered. We used study-level data instead of individual patient data, so the small number of studies limited the additional analyses that could be conducted to account for effect modifiers in the absence of patient-level data. There is also a limited ability of studies with a mean duration of about 12 weeks to inform long-term (chronic) management and treatment decisions. We selected for our primary efficacy outcome of response rate to use a dichotomous rather than continuous measure (e.g. standardized mean difference), because from a clinical perspective, syntheses of continuous outcomes measured on different scales can be difficult to interpret [30,31]. However, a limitation of this method is that there can be a substantial information loss when continuous outcome variables are dichotomised. Dichotomisation may also increase the risk of a positive result being a false positive [91–93]. Furthermore, we were unable to investigate other important outcomes such as remission and recovery (e.g. no longer meeting the criteria for the diagnosis of ADHD), potential serious adverse effects (e.g. ischaemic heart disease, hepatic damage and suicidal ideation/behaviour), or other aspects such as drugs abuse/misuse, resource allocation and costs [6–8,94–99]. Finally, our results might have limited generalizability (social, economic, educational and cultural context), because studies were mostly conducted in children and adolescents from Western countries. Consequently, our results should be interpreted with caution given the caveats mentioned above.

There is still room for improvement in the design, conduct and reporting of trials in the field. The clinical research agenda of ADHD is incomplete and needs better designed and reported randomised trials to assess the benefits and harms of the multiple interventions available for the management of ADHD in the young people. In this respect, ADHD complex pathogenesis implicates multiple factors of a diverse nature that should be considered in clinical research [6–8]. A pertinent research agenda [100,101] which views ADHD dimensionally, but also integrating mental health services and interventions into priority care strategies, should be established in order to appreciate which treatments are best for ADHD children of different ages and in different communities. There is an urgent need for long-term randomised trials at low-risk of bias and with sufficient numbers of participants. It is important that future clinical trials report completely the methods and results for all outcomes of interest (including adverse events) using standardised outcome measures at similar time points to ensure include in future evidence syntheses. Reporting guidelines such as SPIRIT (Standard Protocol Items: Recommendations for Intervention Trials) [102] and CONSORT (Consolidated Standards of Reporting Trials) [103–106] should be rigorously adopted and implemented for study protocols and study reports, respectively.

In conclusion, although the quality of evidence is not strong, clinical differences may exist between the pharmacological and non-pharmacological treatments commonly used for the management of ADHD. Behavioural therapy and pharmacological treatment may improve the symptoms of ADHD and global functioning in the short-term. Our findings can help clinicians, healthcare providers, parents, and caregivers make informed decisions regarding treatment selection for the management of ADHD. An open and honest discussion with parents and older children about uncertainties of available treatments and the balance between benefits, costs, and potential harms should be established before starting treatment. In addition, there is an urgent need for high-quality randomised trials of the multiple treatments for ADHD in children and adolescents.

## Supporting information

S1 Checklist Completed PRISMA NMA checklist for the present systematic review. (DOCX)

S1 Text Methods clarifications/modifications from the protocol. (DOCX)

S2 Text PubMed search terms. (DOCX)

S3 Text List of screened systematic reviews and/or meta-analyses. (DOCX)

S4 Text Reference list of all included studies. (DOCX)

S1 Table Baseline characteristics of included studies. (DOCX)

S2 Table Definitions of study participants, interventions and outcomes. (DOCX)

S3 Table Risk of bias and sponsorship of included studies. (DOCX)

S4 Table Number of events per trial and treatment comparison for response, all-cause discontinuation, tolerability and serious AEs. (DOCX)

S5 Table Number of events per trial and treatment comparison for specific adverse effects. (DOCX)

S6 Table Network meta-analyses for efficacy and acceptability of classes of interventions. (DOCX)

S7 Table Quality of evidence (GRADE) for primary outcomes. (DOCX)

S8 Table Additional analyses. (DOCX)

S9 Table Summary of model fit statistics from network meta-analysis by outcome. (DOCX)

S10 Table Pairwise meta-analysis and heterogeneity for the primary outcomes. (DOCX)

S1 Fig PRISMA flow diagram for study selection process. (DOCX)

S2 Fig Network geometry. (DOCX)

S3 Fig Publication bias for primary outcomes. (DOCX)
