# Supplementary material for: The pharmacological and non-pharmacological treatment of attention deficit hyperactivity disorder in children and adolescents: A systematic review with network meta-analyses of randomised trials
Source: PLoS One. 2017 Jul 12;12(7):e0180355. doi: 10.1371/journal.pone.0180355 (PMC5507500; doi:10.1371/journal.pone.0180355)
Supplement: S1 Text — (DOCX) [file pone.0180355.s002.docx]

**S1 Text. Methods clarifications/modifications from the protocol**

The protocol has been registered in PROSPERO (No. CRD42014015008), and published in the open-access journal *Systematic Reviews*, available at: <https://systematicreviewsjournal.biomedcentral.com/articles/10.1186/s13643-015-0005-7>

**Clarification 2.1: Type of interventions**

During the screening process, we were contacted by decision makers and scientists regarding the inclusion of ‘other approved or unapproved drugs used in ADHD’ not described in our published protocol. In particular, we were asked for the inclusion of studies examining the effects of antipsychotics for treating ADHD in children and adolescents. After careful consideration by the review team and before data extraction, other pharmacological treatments including antipsychotics (such as risperidone and aripiprazole) available in the ADHD literature were considered eligible for inclusion in our network meta-analysis. These studies were included in the evidence networks in order to incorporate additional indirect evidence for the analyses while preserving randomization of existing trials. Although very limited evidence was found, the results for these other pharmacological treatments have been presented in our review.

**Clarification 2.2: Data synthesis**

Page 4 of published protocol (Types of outcome measures): (…) “*When dichotomous outcomes are not reported in studies, but baseline scores, endpoint means and standard deviations (SD) of the rating scales are provided, we will attempt to estimate the number of patients responding to treatment with the validated imputation method previously employed by Furukawa et al. (2005) [56].”*

Clarification for treatment response: When dichotomous outcomes were not reported in included studies, we originally mentioned in our published review protocol the possibility of applying imputation methods for estimating the number of patients responding to treatment (based on continuous measures of the ratings). However, during the data extraction process we observed that baseline scores, endpoint means and standard deviations were incompletely and inconsistently reported among studies not reporting dichotomous outcome data. Considering the large number of studies included in the review, we therefore decided not to apply the imputation procedures proposed by Furukawa and colleagues for trials in other mental disorders (such as depression and schizophrenia in adults). This decision was made during the data extraction and before the data analysis.

**Clarification 2.3: Description and classification of interventions**

The description of the interventions was extracted from each eligible report. A small multidisciplinary group of the review team (including 4 members: 1 clinical psychologist, 1 psychiatrist, 1 clinical epidemiologist/pharmacist and 1 health economist) assessed the treatment interventions and grouped similar (homogeneous) but not necessarily identical interventions under the same therapeutic class, considering mechanism of action and/or licensing status (in the case of drugs). Regarding controls, we distinguished 3 classes: placebo (pill and psychological), control (usual care, conventional therapy or control) and waiting list (in psychological trials). Regarding psychological interventions, we distinguished 4 classes of treatment: behavioural therapy, cognitive training, neurofeedback, and other psychotherapies. Regarding pharmacological therapy, we distinguished 5 classes of treatment: stimulants (including amphetamines and methylphenidate), non-stimulants (including atomoxetine, clonidine and guanfacine), antidepressants (including bupropion, venlafaxine, reboxetine, desipramine, imipramine, nortriptyline, clomipramine, amitriptyline), antipsychotics (including risperidone, aripiprazole, thioridazine), and other unlicensed drugs (modafinil and carbamazepine). Regarding complementary and alternative medine interventions, we distinguished 7 classes of treatment: diet therapy (such as restricted elimination diet), polyunsaturated fatty acids (PUFA), amino acids, minerals, herbal therapy, homeopathy, and physical activity. Finally, we also considered any possible combination among the above treatment alternatives (for example: stimulants plus behavioural therapy, non-stimulants plus behavioural therapy, stimulants plus non-stimulants, etc.)

**Clarification 2.4: Geometry of networks of trials**

For a given network of trials, each treatment node represented an intervention and each edge a randomised comparison of 2 (or more) interventions. We produced graphs for 2 levels of treatment networks of randomised trials. The first treatment network included all trials and treatment nodes corresponding to interventions at the therapeutic class level as labeled by the review team (as described above). The second treatment network included trials contributing to the outcomes at the single (specific) intervention level. For psychological interventions, we distinguished behavioural therapies such as parent training, child/parent/teacher training, and cognitive training such as attention training and working memory training. For pharmacological interventions, we distinguished commonly prescribed medications such as methylphenidate, amphetamine, atomoxetine, clonidine, guanfacine, modafinil and bupropion, and other investigated drugs such as desipramine, reboxetine, venlafaxine, thioridazine, etc. In sensitivity analyses, more complex treatment networks included trials contributing to the outcome at the single level for both psychological and pharmacological interventions (as described in section 2.3 for second network), but for pharmacological interventions nodes included dosage for single stimulants (low/moderate vs high) and type of formulations (long acting- vs short acting).

**Clarification 2.5: Treatment rankings**

Since the amount of evidence for a number a treatment comparisons was very limited and data in networks sparse, we did not feel it was appropriate to conduct and report analyses of treatment rankings in our review. As described elsewhere, an intervention may be ranked highly even though it was assessed in only a few trials and with few patients, which may result in a misleading strong endorsement for the intervention despite large uncertainty. Treatment rankings and probabilities can be fragile when the network is sparse. For that reason, it is generally recommended that authors should place less emphasis on the probabilities and treatment rankings output and greater emphasis on the treatment effects and their uncertainty. Discussion is ongoing as to whether rankings must be reported and are appropriate to derive inference [Mills et al., 2013; Trinquart et al., 2016]. See the following references:

Trinquart L, Attiche N, Bafeta A, Porcher R, Ravaud P. Uncertainty in Treatment Rankings: Reanalysis of Network Meta-analyses of Randomized Trials. Ann Intern Med. 2016;164(10):666-73. [Epub ahead of print] PubMed PMID: 27089537

Mills EJ, Thorlund K, Ioannidis JP. Demystifying trial networks and network meta-analysis. BMJ. 2013 May 14;346:f2914. PubMed PMID: 23674332

**Clarification 2.6: Quality of evidence and GRADE approach**

We used the Grading of Recommendations Assessment, Development, and Evaluation (GRADE) methodology to evaluate the quality of evidence for each outcome. For purposes of systematic reviews, the GRADE approach defines the quality of a body of evidence as the extent to which one can be confident that an estimate of effect or association is close to the quantity of specific interest. Quality of a body of evidence involves consideration of within-study risk of bias (methodological quality), inconsistency (directness of evidence)heterogeneity, precision of effect estimates and risk of publication bias. In this review, the following factors/reasons were used for downgrading the quality of evidence for a specific outcome: 1) Risk of bias: Our confidence in an estimate of effect decreased if studies suffered from major limitations that were likely to result in a biased assessment of the intervention effect (e.g. lack of allocation concealment, lack of blinding, a large loss to follow-up, randomised trials stopped early for benefit or selective reporting of outcomes). 2) Imprecision of results: When studies include few participants and few events and thus have wide confidence intervals, we lowered their rating of the quality of the evidence. 3) Unexplained inconsistency of results: When inconsistency potentially existed and affected the interpretation of results, but we failed to identify a plausible explanation, the quality of evidence was downgraded. Publication bias was quantitatively assessed, but inadequate numbers of included trials with direct comparisons prevented us from drawing firm conclusions from funnel plots and statistical tests. Therefore, publication bias was not considered a reason for downgrading the quality of the body of evidence for a specific outcome.

**Clarification 2.7: Example of WinBUGS code for main analyses (network meta-analysis)**

model{

for(i in 1:ns){

# Loop through studies

# Adjustment for multi-arm trials is zero for control arm

w[i,1] <- 0

# Treatment effect is zero for control arm

delta[i,1] <- 0

# Vague priors for trial baselines

mu[i] ~ dnorm(0,.0001)

# Binomial likelihood

# Loop through arms

for (k in 1:na[i]) {

r[i,k] ~ dbin(p[i,k],n[i,k])

#parametization of the model for NMA

logit(p[i,k]) <- mu[i] + delta[i,k] # Model for linear predictor

rhat[i,k] <- p[i,k] * n[i,k] # Expected value of the numerators

dev[i,k] <- 2 * (r[i,k] * (log(r[i,k])-log(rhat[i,k])) # Deviance contribution

+ (n[i,k]-r[i,k]) * (log(n[i,k]-r[i,k])-log(n[i,k]-rhat[i,k])))

}

# Summed residual deviance

resdev[i] <- sum(dev[i, 1:na[i]]) # contribution for this trial

# Loop through arms

for (k in 2:na[i]) {

delta[i,k] ~ dnorm(md[i,k],taud[i,k]) # Trial-specific LOR distributions

md[i,k] <- d[t[i,k]] - d[t[i,1]] + sw[i,k] # Mean of LOR distributions

# (with multi-arm trial correction)

taud[i,k] <- tau *2*(k-1)/k # Precision of LOR distributions

# (with multi-arm trial correction)

w[i,k] <- (delta[i,k] - d[t[i,k]] + d[t[i,1]]) # Adjustment for multi-arm RCTs

sw[i,k] <- sum(w[i,1:k-1])/(k-1) # Cumulative adjustment for multi-arm trials

}

}

# Total Residual Deviance

totresdev <- sum(resdev[])

# treatment effect is zero for reference treatment

d[1]<-0

#priors#

for (k in 2:nt){ d[k] ~ dnorm(0,.0001) } # vague priors for treatment effects

sd ~ dunif(0,5) # vague prior for between-trial SD

tau <- pow(sd,-2) # between-trial precision = (1/between-trial variance)

# Pairwise ORs and LORs for all possible pair-wise comparisons, if nt>2

for (c in 1:(nt-1)) {

for (k in (c+1):nt) {

lor[c,k] <- (d[k]-d[c])

or[c,k] <- exp(lor[c,k])

}

}

# Predictive distribution for future trial is multivariate normal

delta.new[1] <- 0 # Treatment effect is zero for reference treatment

w.new[1] <- 0 # Adjustment for conditional mean is zero for reference treatment

# Loop through treatments

for (k in 2:nt) {

delta.new[k] ~ dnorm(m.new[k],tau.new[k]) # Conditional distribution of each delta.new

m.new[k] <- d[k] + sw.new[k] # Conditional mean of delta.new

tau.new[k] <- tau *2*(k-1)/k # Conditional precision of delta.new

w.new[k] <- delta.new[k] - d[k] # Adjustment for conditional mean

sw.new[k] <- sum(w.new[1:k-1])/(k-1) # Cumulative adjustment for conditional mean

}

p.base ~ dbeta(a,b) # Draw baseline (control group) effect

a <- r[ns,1] # No. of events in control group

b <- n[ns,1] - r[ns,1] # No. of non-events in control group

# Loop through treatments or studies?

for (k in 2:na[ns]) {

# Predictive probability of event for each treatment arm of the new trial

logit(p.new[k]) <- logit(p.base) + (delta.new[t[ns,k]]- delta.new[t[ns,1]])

r.new[k] ~ dbin(p.new[k], n[ns,k]) # draw predicted number of events for each arm of the new trial

# Bayesian p-value: probability of obtaining a value as extreme as the

# value observed (r[ns,2]), given the model and the remaining data

p.cross[k] <- step(r[ns,2] - r.new[k]) - 0.5*equals(r.new[k],r[ns,2]) # extreme value “smaller”

}

# pairwise ORs and LORs for all possible pair-wise comparisons, if nt>2

for (c in 1:(nt-1)) {

for (k in (c+1):nt) {

lor.new[c,k] <- delta.new[k]- delta.new[c]

or.new[c,k] <- exp(lor.new[c,k])

}

}

} # End of model
