# Supplementary material for: The pharmacological and non-pharmacological treatment of attention deficit hyperactivity disorder in children and adolescents: A systematic review with network meta-analyses of randomised trials
Source: PLoS One. 2017 Jul 12;12(7):e0180355. doi: 10.1371/journal.pone.0180355 (PMC5507500; doi:10.1371/journal.pone.0180355)
Supplement: S2 Text — (DOCX) [file pone.0180355.s003.docx]

**S2 Text. PubMed search terms and history**

| **Search** | **Query** | **Items found** |
| --- | --- | --- |
| #1 | **Search**attention deficit disorder with hyperactivity OR adhd OR hyperkinetic* OR inattent* OR impulsivity OR hyperkinesis OR tdah | 55495 |
| #2 | **Search**child OR children OR adolescent* OR pediatric* OR paediatric* | 3197879 |
| #3 | **Search**meta-analy* OR metanaly* OR metaanaly* OR metanaly* OR integrative research OR research integration OR research overview* OR collaborative review* OR systematic review* OR systematic overview* OR evidence based review* OR evidence-based OR overview* OR meta-review* OR review of reviews OR technology assessment* OR HTA | 2421298 |
| #4 | **Search** methylphenidate OR equasym OR ritalin OR concerta OR rubifen OR tranquilyn OR phenidylate OR methyl phenidate OR dexmethylphenidate OR focalin OR metilfenidato OR dextroamphetamine OR dexamphetamine OR dexamfetamine OR elvanse OR vyvanse OR venvanse OR tyvense OR d amphetamine OR amphetamine OR Adderall OR dexedrine OR lisdexamfetamine OR atomoxetine OR tomoxetine OR strattera OR guanfacine OR guanfacina OR tenex OR intuniv OR clonidine OR kapvay OR nexiclon OR catapres OR modafinil OR bupropion OR venlafaxine OR reboxetine OR desipramine OR imipramine OR nortriptyline OR clomipramine OR amitriptyline OR antidepressant* OR contingency management OR management techniques OR contingency techniques OR psychosocial interventions OR psychosocial treatment OR psychosocial therapy OR social skills training OR social skills intervention OR social skills treatment OR problem solving intervention OR problem solving treatment OR problem solving training OR problem solving therapy OR behavior modification OR cognitive behavior treatment OR cognitive behavior therapy OR cognitive behavior training OR parent training OR parent counselling OR parent support OR school-based OR classroombased OR school intervention OR classroom intervention OR teacher training OR after-school or remedial teaching OR peer tutoring OR computer assistance learning OR task modification OR curriculum modification OR classroom management OR education intervention OR multimodal intervention OR multimodal treatment OR multimodal therapy OR multimodal intervention OR multimodal treatment OR multimodal therapy OR educational intervention OR and verbal self-instruction training OR cognitive training OR attention training OR working memory training OR cognitive remediation OR executive function training OR and cognitive control OR neurofeedback OR EEG biofeedback OR neurotherapy OR slow cortical potentials OR few foods diet OR elimination diet OR oligoantigenic diet OR restriction diet OR food intolerance OR food allergy OR and food hypersensitivity OR food color OR food dye OR Feingold diet OR Kaiser Permanente diet OR K-P diet OR tartrazine OR azo dye OR carmoisine OR sunset yellow OR brilliant blue OR indigotine OR allura red OR quinoline yellow OR ponceau 4R OR essential fatty acid OR long-chain polyunsaturated fatty acids OR PUFA OR omega-3 OR omega-6 OR docosahexaenoic acid OR eicosapentaenoic acid OR arachidonic acid OR vitamin B6 OR pyridoxine OR vitamin B9 OR folate OR vitamin B12 OR cobalamin OR magnesium OR zinc OR iron OR calcium OR aminoacid OR amino acid OR carnitine OR L-carnitine OR L-tyrosine OR tyrosine OR tryptophan OR glycine OR melatonin OR taurine OR 5-HTP OR phenilalananine OR aspartame OR ginkgo OR ginkgo biloba OR ginseng OR St John's Wort OR hypericum perforatum OR rhodiola OR chamomile OR valerian OR bacopa OR pinus marinus OR massage OR chiropractic OR osteopathic manipulation OR acupuncture OR yoga OR meditation OR Tai chi OR complementary medicine OR alternative medicine OR complementary and alternative medicine | 4323828 |
| **#5** | **Search #1 AND #2 AND #3 AND #4**  **Filters: Publication date from 2005/01/01 to 2016/04/07** | **1772** |
