# Supplementary material for: The pharmacological and non-pharmacological treatment of attention deficit hyperactivity disorder in children and adolescents: A systematic review with network meta-analyses of randomised trials
Source: PLoS One. 2017 Jul 12;12(7):e0180355. doi: 10.1371/journal.pone.0180355 (PMC5507500; doi:10.1371/journal.pone.0180355)
Supplement: S3 Text — (DOCX) [file pone.0180355.s004.docx]

**S3 Text. List of screened systematic reviews and/or meta-analyses**

1. Richardson M, Moore DA, Gwernan-Jones R, Thompson-Coon J, Ukoumunne O, Rogers M, Whear R, Newlove-Delgado TV, Logan S, Morris C, Taylor E, Cooper P, Stein K, Garside R, Ford TJ. Non-pharmacological interventions for attention-deficit/hyperactivity disorder (ADHD) delivered in school settings: systematic reviews of quantitative and qualitative research. Health Technol Assess. 2015 Jun;19(45):1-470. doi: 10.3310/hta19450. PubMed PMID: 26129788
2. Stuhec M, Munda B, Svab V, Locatelli I. Comparative efficacy and acceptability of atomoxetine, lisdexamfetamine, bupropion and methylphenidate in treatment of attention deficit hyperactivity disorder in children and adolescents: a meta-analysis with focus on bupropion. J Affect Disord. 2015 Jun 1;178:149-59. doi: 10.1016/j.jad.2015.03.006. Epub 2015 Mar 13. Review. PubMed PMID: 25813457.
3. Cerrillo-Urbina AJ, García-Hermoso A, Sánchez-López M, Pardo-Guijarro MJ, Santos Gómez JL, Martínez-Vizcaíno V. The effects of physical exercise in children with attention deficit hyperactivity disorder: a systematic review and meta-analysis of randomized control trials. Child Care Health Dev. 2015 May 18. doi: 10.1111/cch.12255. [Epub ahead of print] PubMed PMID: 25988743.
4. Ghanizadeh A. A systematic review of reboxetine for treating patients with attention deficit hyperactivity disorder. Nord J Psychiatry. 2015 May;69(4):241-8. doi: 10.3109/08039488.2014.972975. Epub 2014 Nov 21. PubMed PMID: 25415763.
5. Fabiano GA, Schatz NK, Aloe AM, Chacko A, Chronis-Tuscano A. A systematic review of meta-analyses of psychosocial treatment for attention-deficit/hyperactivity disorder. Clin Child Fam Psychol Rev. 2015 Mar;18(1):77-97. doi: 10.1007/s10567-015-0178-6. PubMed PMID: 25691358; PubMed Central PMCID: PMC4346344.
6. Cortese S, Ferrin M, Brandeis D, Buitelaar J, Daley D, Dittmann RW, Holtmann M, Santosh P, Stevenson J, Stringaris A, Zuddas A, Sonuga-Barke EJ; European ADHD Guidelines Group (EAGG). Cognitive training for attention-deficit/hyperactivity disorder: meta-analysis of clinical and neuropsychological outcomes from randomized controlled trials. J Am Acad Child Adolesc Psychiatry. 2015 Mar;54(3):164-74. doi: 10.1016/j.jaac.2014.12.010. Epub 2014 Dec 29. Review. PubMed PMID: 25721181
7. Mulqueen JM, Bartley CA, Bloch MH. Meta-analysis: parental interventions for preschool ADHD. J Atten Disord. 2015 Feb;19(2):118-24. doi:10.1177/1087054713504135. Epub 2013 Sep 26. PubMed PMID: 24071773.
8. Maneeton B, Maneeton N, Likhitsathian S, Suttajit S, Narkpongphun A, Srisurapanont M, Woottiluk P. Comparative efficacy, acceptability, and tolerability of lisdexamfetamine in child and adolescent ADHD: a meta-analysis of randomized, controlled trials. Drug Des Devel Ther. 2015 Apr 1;9:1927-36. doi:10.2147/DDDT.S79071. eCollection 2015. PubMed PMID: 25897203
9. Watson SM, Richels C, Michalek AP, Raymer A. Psychosocial treatments for ADHD: a systematic appraisal of the evidence. J Atten Disord. 2015 Jan;19(1):3-10. doi:10.1177/1087054712447857. Epub 2012 May 30. Review. PubMed PMID: 22647286
10. Heilskov Rytter MJ, Andersen LB, Houmann T, Bilenberg N, Hvolby A, Mølgaard C, Michaelsen KF, Lauritzen L. Diet in the treatment of ADHD in children – a systematic review of the literature. Nord J Psychiatry. 2015 Jan;69(1):1-18. doi:10.3109/08039488.2014.921933. Epub 2014 Jun 16. Review. PubMed PMID: 24934907
11. Rubia K, Alegria AA, Cubillo AI, Smith AB, Brammer MJ, Radua J. Effects of stimulants on brain function in attention-deficit/hyperactivity disorder: a systematic review and meta-analysis. Biol Psychiatry. 2014 Oct 15;76(8):616-28. doi:10.1016/j.biopsych.2013.10.016. Epub 2013 Oct 24. Review. PubMed PMID: 24314347; PubMed Central PMCID: PMC4183380.
12. Coghill DR, Seth S, Pedroso S, Usala T, Currie J, Gagliano A. Effects of methylphenidate on cognitive functions in children and adolescents with attention-deficit/hyperactivity disorder: evidence from a systematic review and a meta-analysis. Biol Psychiatry. 2014 Oct 15;76(8):603-15. doi:10.1016/j.biopsych.2013.10.005. Epub 2013 Oct 12. Review. PubMed PMID: 24231201
13. Bloch MH, Mulqueen J. Nutritional supplements for the treatment of ADHD. Child Adolesc Psychiatr Clin N Am. 2014 Oct;23(4):883-97. doi:10.1016/j.chc.2014.05.002. Epub 2014 Aug 12. Review. PubMed PMID: 25220092; PubMed Central PMCID: PMC4170184.
14. Ruggiero S, Clavenna A, Reale L, Capuano A, Rossi F, Bonati M. Guanfacine for attention deficit and hyperactivity disorder in pediatrics: a systematic review and meta-analysis. Eur Neuropsychopharmacol. 2014 Oct;24(10):1578-90. doi:10.1016/j.euroneuro.2014.08.001. Epub 2014 Aug 11. PubMed PMID: 25156577
15. Otasowie J, Castells X, Ehimare UP, Smith CH. Tricyclic antidepressants for attention deficit hyperactivity disorder (ADHD) in children and adolescents. Cochrane Database Syst Rev. 2014 Sep 19;9:CD006997. doi:10.1002/14651858.CD006997.pub2. Review. PubMed PMID: 25238582
16. Clavenna A, Bonati M. Safety of medicines used for ADHD in children: a review of published prospective clinical trials. Arch Dis Child. 2014 Sep;99(9):866-72. doi: 10.1136/archdischild-2013-304170. Epub 2014 Apr 19. Review. PubMed PMID: 24748641
17. Roskell NS, Setyawan J, Zimovetz EA, Hodgkins P. Systematic evidence synthesis of treatments for ADHD in children and adolescents: indirect treatment comparisons of lisdexamfetamine with methylphenidate and atomoxetine. Curr Med Res Opin. 2014 Aug;30(8):1673-85. doi: 10.1185/03007995.2014.904772. Epub 2014 Apr 15. Review. PubMed PMID: 24627974
18. Connor DF, Arnsten AF, Pearson GS, Greco GF. Guanfacine extended release for the treatment of attention-deficit/hyperactivity disorder in children and adolescents. Expert Opin Pharmacother. 2014 Aug;15(11):1601-10. doi:10.1517/14656566.2014.930437. Epub 2014 Jul 3. Review. PubMed PMID: 24992513
19. Daley D, van der Oord S, Ferrin M, Danckaerts M, Doepfner M, Cortese S, Sonuga-Barke EJ; European ADHD Guidelines Group. Behavioral interventions in attention-deficit/hyperactivity disorder: a meta-analysis of randomized controlled trials across multiple outcome domains. J Am Acad Child Adolesc Psychiatry. 2014 Aug;53(8):835-47, 847.e1-5. doi: 10.1016/j.jaac.2014.05.013. Epub 2014 Jun 26. Review. PubMed PMID: 25062591
20. Kamp CF, Sperlich B, Holmberg HC. Exercise reduces the symptoms of attention-deficit/hyperactivity disorder and improves social behaviour, motor skills, strength and neuropsychological parameters. Acta Paediatr. 2014 Jul;103(7):709-14. doi: 10.1111/apa.12628. Epub 2014 Apr 29. Review. PubMed PMID: 24612421
21. Coates J, Taylor JA, Sayal K. Parenting Interventions for ADHD: A Systematic Literature Review and Meta-Analysis. J Atten Disord. 2014 Jun 10. pii: 1087054714535952. [Epub ahead of print] PubMed PMID: 24915737
22. Coghill DR, Caballero B, Sorooshian S, Civil R. A systematic review of the safety of lisdexamfetamine dimesylate. CNS Drugs. 2014 Jun;28(6):497-511. doi:10.1007/s40263-014-0166-2. Review. PubMed PMID: 24788672; PubMed Central PMCID: PMC4057639
23. Hodgson K, Hutchinson AD, Denson L. Nonpharmacological treatments for ADHD: a meta-analytic review. J Atten Disord. 2014 May;18(4):275-82. doi:10.1177/1087054712444732. Epub 2012 May 29. Review. PubMed PMID: 22647288
24. Stevenson J, Buitelaar J, Cortese S, Ferrin M, Konofal E, Lecendreux M, Simonoff E, Wong IC, Sonuga-Barke E. Research review: the role of diet in the treatment of attention-deficit/hyperactivity disorder--an appraisal of the evidence on efficacy and recommendations on the design of future studies. J Child Psychol Psychiatry. 2014 May;55(5):416-27. doi: 10.1111/jcpp.12215. Epub 2014 Feb 19. Review. PubMed PMID: 24552603
25. Vollebregt MA, van Dongen-Boomsma M, Buitelaar JK, Slaats-Willemse D. Does EEG-neurofeedback improve neurocognitive functioning in children with attention-deficit/hyperactivity disorder? A systematic review and a double-blind placebo-controlled study. J Child Psychol Psychiatry. 2014 May;55(5):460-72. doi:10.1111/jcpp.12143. Epub 2013 Oct 30. Erratum in: J Child Psychol Psychiatry. 2014 Aug;55(8):954-5. PubMed PMID: 24168522
26. Puri BK, Martins JG. Which polyunsaturated fatty acids are active in children with attention-deficit hyperactivity disorder receiving PUFA supplementation? A fatty acid validated meta-regression analysis of randomized controlled trials. Prostaglandins Leukot Essent Fatty Acids. 2014 May;90(5):179-89. doi:10.1016/j.plefa.2014.01.004. Epub 2014 Feb 3. Review. PubMed PMID: 24560325
27. Sibley MH, Kuriyan AB, Evans SW, Waxmonsky JG, Smith BH. Pharmacological and psychosocial treatments for adolescents with ADHD: an updated systematic review of the literature. Clin Psychol Rev. 2014 Apr;34(3):218-32. doi:10.1016/j.cpr.2014.02.001. Epub 2014 Feb 27. Review. PubMed PMID: 24632046
28. Schwartz S, Correll CU. Efficacy and safety of atomoxetine in children and adolescents with attention-deficit/hyperactivity disorder: results from a comprehensive meta-analysis and metaregression. J Am Acad Child Adolesc Psychiatry. 2014 Feb;53(2):174-87. doi: 10.1016/j.jaac.2013.11.005. Epub 2013 Nov 26. PubMed PMID: 24472252
29. Hirota T, Schwartz S, Correll CU. Alpha-2 agonists for attention-deficit/hyperactivity disorder in youth: a systematic review and meta-analysis of monotherapy and add-on trials to stimulant therapy. J Am Acad Child Adolesc Psychiatry. 2014 Feb;53(2):153-73. doi: 10.1016/j.jaac.2013.11.009. Epub 2013 Dec 6. Review. PubMed PMID: 24472251
30. Park P, Caballero J, Omidian H. Use of serotonin norepinephrine reuptake inhibitors in the treatment of attention-deficit hyperactivity disorder in pediatrics. Ann Pharmacother. 2014 Jan;48(1):86-92. doi:10.1177/1060028013506561. Epub 2013 Oct 9. Review. PubMed PMID: 24259607
31. Micoulaud-Franchi JA, Geoffroy PA, Fond G, Lopez R, Bioulac S, Philip P. EEG neurofeedback treatments in children with ADHD: an updated meta-analysis of randomized controlled trials. Front Hum Neurosci. 2014 Nov 13;8:906. doi:10.3389/fnhum.2014.00906. eCollection 2014. Review. PubMed PMID: 25431555
32. Evans SW, Owens JS, Bunford N. Evidence-based psychosocial treatments for children and adolescents with attention-deficit/hyperactivity disorder. J Clin Child Adolesc Psychol. 2014;43(4):527-51. doi: 10.1080/15374416.2013.850700. Epub 2013 Nov 18. Review. PubMed PMID: 24245813
33. Gajria K, Lu M, Sikirica V, Greven P, Zhong Y, Qin P, Xie J. Adherence, persistence, and medication discontinuation in patients with attention-deficit/hyperactivity disorder - a systematic literature review. Neuropsychiatr Dis Treat. 2014 Aug 22;10:1543-69. doi: 10.2147/NDT.S65721. eCollection 2014. Review. PubMed PMID: 25187718; PubMed Central PMCID: PMC4149449
34. Maneeton N, Maneeton B, Intaprasert S, Woottiluk P. A systematic review of randomized controlled trials of bupropion versus methylphenidate in the treatment of attention-deficit/hyperactivity disorder. Neuropsychiatr Dis Treat. 2014 Aug 1;10:1439-49. doi: 10.2147/NDT.S62714. eCollection 2014. PubMed PMID: 25120365; PubMed Central PMCID: PMC4128852
35. Coghill D, Banaschewski T, Zuddas A, Pelaz A, Gagliano A, Doepfner M. Long-acting methylphenidate formulations in the treatment of attention-deficit/hyperactivity disorder: a systematic review of head-to-head studies. BMC Psychiatry. 2013 Sep 27;13:237. doi: 10.1186/1471-244X-13-237. Review. PubMed PMID: 24074240
36. Parker J, Wales G, Chalhoub N, Harpin V. The long-term outcomes of interventions for the management of attention-deficit hyperactivity disorder in children and adolescents: a systematic review of randomized controlled trials. Psychol Res Behav Manag. 2013 Sep 17;6:87-99. doi: 10.2147/PRBM.S49114. Review. PubMed PMID: 24082796
37. Ghanizadeh A. A systematic review of the efficacy and safety of desipramine for treating ADHD. Curr Drug Saf. 2013 Jul;8(3):169-74. Review. PubMed PMID: 23914752
38. Sallee F, Connor DF, Newcorn JH. A review of the rationale and clinical utilization of α2-adrenoceptor agonists for the treatment of attention-deficit/hyperactivity and related disorders. J Child Adolesc Psychopharmacol. 2013 Jun;23(5):308-19. doi: 10.1089/cap.2013.0028. Review. PubMed PMID: 23782125
39. Linton D, Barr AM, Honer WG, Procyshyn RM. Antipsychotic and psychostimulant drug combination therapy in attention deficit/hyperactivity and disruptive behavior disorders: a systematic review of efficacy and tolerability. Curr Psychiatry Rep. 2013 May;15(5):355. doi: 10.1007/s11920-013-0355-6. Review. PubMed PMID: 23539465
40. Tanaka Y, Rohde LA, Jin L, Feldman PD, Upadhyaya HP. A meta-analysis of the consistency of atomoxetine treatment effects in pediatric patients with attention-deficit/hyperactivity disorder from 15 clinical trials across four geographic regions. J Child Adolesc Psychopharmacol. 2013 May;23(4):262-70. doi: 10.1089/cap.2012.0049. PubMed PMID: 23683141
41. Charach A, Carson P, Fox S, Ali MU, Beckett J, Lim CG. Interventions for preschool children at high risk for ADHD: a comparative effectiveness review. Pediatrics. 2013 May;131(5):e1584-604. doi: 10.1542/peds.2012-0974. Epub 2013 Apr 1. Review. PubMed PMID: 23545375
42. Prasad V, Brogan E, Mulvaney C, Grainge M, Stanton W, Sayal K. How effective are drug treatments for children with ADHD at improving on-task behaviour and academic achievement in the school classroom? A systematic review and meta-analysis. Eur Child Adolesc Psychiatry. 2013 Apr;22(4):203-16. doi:10.1007/s00787-012-0346-x. Epub 2012 Nov 21. Review. PubMed PMID: 23179416
43. Treuer T, Gau SS, Méndez L, Montgomery W, Monk JA, Altin M, Wu S, Lin CC, Dueñas HJ. A systematic review of combination therapy with stimulants and atomoxetine for attention-deficit/hyperactivity disorder, including patient characteristics, treatment strategies, effectiveness, and tolerability. J Child Adolesc Psychopharmacol. 2013 Apr;23(3):179-93. doi: 10.1089/cap.2012.0093. Epub 2013 Apr 6. Review. PubMed PMID: 23560600; PubMed Central PMCID: PMC3696926
44. Punja S, Zorzela L, Hartling L, Urichuk L, Vohra S. Long-acting versus short-acting methylphenidate for paediatric ADHD: a systematic review and meta-analysis of comparative efficacy. BMJ Open. 2013 Mar 15;3(3). pii: e002312. doi: 10.1136/bmjopen-2012-002312. PubMed PMID: 23503579
45. Sonuga-Barke EJ, Brandeis D, Cortese S, Daley D, Ferrin M, Holtmann M, Stevenson J, Danckaerts M, van der Oord S, Döpfner M, Dittmann RW, Simonoff E, Zuddas A, Banaschewski T, Buitelaar J, Coghill D, Hollis C, Konofal E, Lecendreux M, Wong IC, Sergeant J; European ADHD Guidelines Group. Nonpharmacological interventions for ADHD: systematic review and meta-analyses of randomized controlled trials of dietary and psychological treatments. Am J Psychiatry. 2013 Mar;170(3):275-89. doi: 10.1176/appi.ajp.2012.12070991. Review. PubMed PMID: 23360949
46. Grassmann V, Santos-Galduróz RF, Galduróz JC. Effects of low doses of polyunsaturated Fatty acids on the attention deficit/hyperactivity disorder of children: a systematic review. Curr Neuropharmacol. 2013 Mar;11(2):186-96. doi:10.2174/1570159X11311020005. PubMed PMID: 23997753; PubMed Central PMCID: PMC3637672.
47. Cortese S, Holtmann M, Banaschewski T, Buitelaar J, Coghill D, Danckaerts M, Dittmann RW, Graham J, Taylor E, Sergeant J; European ADHD Guidelines Group. Practitioner review: current best practice in the management of adverse events during treatment with ADHD medications in children and adolescents. J Child Psychol Psychiatry. 2013 Mar;54(3):227-46. doi: 10.1111/jcpp.12036. Epub 2013 Jan 7. Review. PubMed PMID: 23294014
48. Ghanizadeh A, Freeman RD, Berk M. Efficacy and adverse effects of venlafaxine in children and adolescents with ADHD: a systematic review of non-controlled and controlled trials. Rev Recent Clin Trials. 2013 Mar;8(1):2-8. Review. PubMed PMID: 23157376
49. Serrano-Troncoso E, Guidi M, Alda-Díez JÁ. Is psychological treatment efficacious for attention deficit hyperactivity disorder (ADHD)? Review of non-pharmacological treatments in children and adolescents with ADHD. Actas Esp Psiquiatr. 2013 Jan-Feb;41(1):44-51. Epub 2013 Jan 1. Review. PubMed PMID: 23440535
50. Ghanizadeh A, Berk M. Zinc for treating of children and adolescents with attention-deficit hyperactivity disorder: a systematic review of randomized controlled clinical trials. Eur J Clin Nutr. 2013 Jan;67(1):122-4. doi:10.1038/ejcn.2012.177. Epub 2012 Nov 21. Review. PubMed PMID: 23169472
51. Ghanizadeh A. A systematic review of magnesium therapy for treating attention deficit hyperactivity disorder. Arch Iran Med. 2013 Jul;16(7):412-7. doi: 013167/AIM.0010. Review. PubMed PMID: 23808779.
52. Ghanizadeh A. Systematic review of clinical trials of aripiprazole for treating attention deficit hyperactivity disorder. Neurosciences (Riyadh). 2013 Oct;18(4):323-9. Review. PubMed PMID: 24141455.
53. Searight HR, Robertson K, Smith T, Perkins S, Searight BK. Complementary and alternative therapies for pediatric attention deficit hyperactivity disorder: a descriptive review. ISRN Psychiatry. 2012 Dec 26;2012:804127. doi:10.5402/2012/804127. Print 2012. PubMed PMID: 23762770; PubMed Central PMCID: PMC3671686
54. Lee PC, Niew WI, Yang HJ, Chen VC, Lin KC. A meta-analysis of behavioral parent training for children with attention deficit hyperactivity disorder. Res Dev Disabil. 2012 Nov-Dec;33(6):2040-9. doi: 10.1016/j.ridd.2012.05.011. Epub2012 Jun 29. PubMed PMID: 22750360
55. Bukstein OG, Head J. Guanfacine ER for the treatment of adolescent attention-deficit/hyperactivity disorder. Expert Opin Pharmacother. 2012 Oct;13(15):2207-13. doi: 10.1517/14656566.2012.721778. Epub 2012 Sep 7. Review. PubMed PMID: 22957772
56. Cortese S, Angriman M, Lecendreux M, Konofal E. Iron and attention deficit/hyperactivity disorder: What is the empirical evidence so far? A systematic review of the literature. Expert Rev Neurother. 2012 Oct;12(10):1227-40. doi: 10.1586/ern.12.116. Review. PubMed PMID: 23082739
57. Shaw M, Hodgkins P, Caci H, Young S, Kahle J, Woods AG, Arnold LE. A systematic review and analysis of long-term outcomes in attention deficit hyperactivity disorder: effects of treatment and non-treatment. BMC Med. 2012 Sep 4;10:99. doi: 10.1186/1741-7015-10-99. Review. PubMed PMID: 22947230; PubMed Central PMCID: PMC3520745
58. Hodgkins P, Shaw M, Coghill D, Hechtman L. Amfetamine and methylphenidate medications for attention-deficit/hyperactivity disorder: complementary treatment options. Eur Child Adolesc Psychiatry. 2012 Sep;21(9):477-92. doi:10.1007/s00787-012-0286-5. Epub 2012 Jul 5. Review. PubMed PMID: 22763750; PubMed Central PMCID: PMC3432777
59. Conway F. Psychodynamic psychotherapy of ADHD: a review of the literature. Psychotherapy (Chic). 2012 Sep;49(3):404-17. doi: 10.1037/a0027344. Epub 2012 Mar 26. Review. PubMed PMID: 22448924
60. Gillies D, Sinn JKh, Lad SS, Leach MJ, Ross MJ. Polyunsaturated fatty acids (PUFA) for attention deficit hyperactivity disorder (ADHD) in children and adolescents. Cochrane Database Syst Rev. 2012 Jul 11;7:CD007986. doi:10.1002/14651858.CD007986.pub2. Review. PubMed PMID: 22786509
61. Lofthouse N, Arnold LE, Hersch S, Hurt E, DeBeus R. A review of neurofeedback treatment for pediatric ADHD. J Atten Disord. 2012 Jul;16(5):351-72. doi:10.1177/1087054711427530. Epub 2011 Nov 16. Review. PubMed PMID: 22090396
62. Moriyama TS, Polanczyk G, Caye A, Banaschewski T, Brandeis D, Rohde LA. Evidence-based information on the clinical use of neurofeedback for ADHD. Neurotherapeutics. 2012 Jul;9(3):588-98. doi: 10.1007/s13311-012-0136-7. Review. PubMed PMID: 22930416; PubMed Central PMCID: PMC3441929
63. van Wyk GW, Hazell PL, Kohn MR, Granger RE, Walton RJ. How oppositionality, inattention, and hyperactivity affect response to atomoxetine versus methylphenidate: a pooled meta-analysis. J Atten Disord. 2012 May;16(4):314-24. doi: 10.1177/1087054710389989. Epub 2011 Feb 2. PubMed PMID: 21289234
64. Wong YW, Kim DG, Lee JY. Traditional Oriental Herbal Medicine for Children and Adolescents with ADHD: A Systematic Review. Evid Based Complement Alternat Med. 2012;2012:520198. doi: 10.1155/2012/520198. Epub 2012 Dec 17. PubMed PMID: 23346205; PubMed Central PMCID: PMC3538416
65. Nigg JT, Lewis K, Edinger T, Falk M. Meta-analysis of attention-deficit/hyperactivity disorder or attention-deficit/hyperactivity disorder symptoms, restriction diet, and synthetic food color additives. J Am Acad Child Adolesc Psychiatry. 2012 Jan;51(1):86-97.e8. doi:10.1016/j.jaac.2011.10.015. PubMed PMID: 22176942; PubMed Central PMCID: PMC4321798
66. Storebø OJ, Skoog M, Damm D, Thomsen PH, Simonsen E, Gluud C. Social skills training for Attention Deficit Hyperactivity Disorder (ADHD) in children aged 5 to 18 years. Cochrane Database Syst Rev. 2011 Dec 7;(12):CD008223. doi:10.1002/14651858.CD008223.pub2. Review. PubMed PMID: 22161422
67. Zwi M, Jones H, Thorgaard C, York A, Dennis JA. Parent training interventions for Attention Deficit Hyperactivity Disorder (ADHD) in children aged 5 to 18 years. Cochrane Database Syst Rev. 2011 Dec 7;(12):CD003018. doi:10.1002/14651858.CD003018.pub3. Review. PubMed PMID: 22161373
68. McDonagh MS, Peterson K, Thakurta S, Low A. Drug Class Review: Pharmacologic Treatments for Attention Deficit Hyperactivity Disorder: Final Update 4 Report [Internet]. Portland (OR): Oregon Health & Science University; 2011 Dec. Available from http://www.ncbi.nlm.nih.gov/books/NBK84419/ PubMed PMID: 22420008
69. Hanwella R, Senanayake M, de Silva V. Comparative efficacy and acceptability of methylphenidate and atomoxetine in treatment of attention deficit hyperactivity disorder in children and adolescents: a meta-analysis. BMC Psychiatry. 2011 Nov 10;11:176. doi: 10.1186/1471-244X-11-176. PubMed PMID: 22074258; PubMed Central PMCID: PMC3229459
70. Hazell PL, Kohn MR, Dickson R, Walton RJ, Granger RE, Wyk GW. Core ADHD symptom improvement with atomoxetine versus methylphenidate: a direct comparison meta-analysis. J Atten Disord. 2011 Nov;15(8):674-83. doi:10.1177/1087054710379737. Epub 2010 Sep 13. PubMed PMID: 20837981
71. Charach A, Dashti B, Carson P, Booker L, Lim CG, Lillie E, Yeung E, Ma J, Raina P, Schachar R. Attention Deficit Hyperactivity Disorder: Effectiveness of Treatment in At-Risk Preschoolers; Long-Term Effectiveness in All Ages; and Variability in Prevalence, Diagnosis, and Treatment [Internet]. Rockville (MD): Agency for Healthcare Research and Quality (US); 2011 Oct. Available from http://www.ncbi.nlm.nih.gov/books/NBK82368/ PubMed PMID: 22191110
72. Bloch MH, Qawasmi A. Omega-3 fatty acid supplementation for the treatment of children with attention-deficit/hyperactivity disorder symptomatology: systematic review and meta-analysis. J Am Acad Child Adolesc Psychiatry. 2011 Oct;50(10):991-1000. doi:10.1016/j.jaac.2011.06.008. Epub 2011 Aug 12. Review. PubMed PMID: 21961774; PubMed Central PMCID: PMC3625948
73. Sarris J, Kean J, Schweitzer I, Lake J. Complementary medicines (herbal and nutritional products) in the treatment of Attention Deficit Hyperactivity Disorder (ADHD): a systematic review of the evidence. Complement Ther Med. 2011 Aug;19(4):216-27. doi: 10.1016/j.ctim.2011.06.007. Epub 2011 Jul 26. Review. PubMed PMID: 21827936
74. Pringsheim T, Steeves T. Pharmacological treatment for Attention Deficit Hyperactivity Disorder (ADHD) in children with comorbid tic disorders. Cochrane Database Syst Rev. 2011 Apr 13;(4):CD007990. doi: 10.1002/14651858.CD007990.pub2. Review. PubMed PMID: 21491404
75. Li S, Yu B, Zhou D, He C, Kang L, Wang X, Jiang S, Chen X. Acupuncture for Attention Deficit Hyperactivity Disorder (ADHD) in children and adolescents. Cochrane Database Syst Rev. 2011 Apr 13;(4):CD007839. doi: 10.1002/14651858.CD007839.pub2. Review. PubMed PMID: 21491402
76. Montoya A, Colom F, Ferrin M. Is psychoeducation for parents and teachers of children and adolescents with ADHD efficacious? A systematic literature review. Eur Psychiatry. 2011 Apr;26(3):166-75. doi: 10.1016/j.eurpsy.2010.10.005. Epub 2011 Feb 2. Review. PubMed PMID: 21292454
77. Keen D, Hadijikoumi I. ADHD in children and adolescents. BMJ Clin Evid. 2011 Feb 4;2011. pii: 0312. PubMed PMID: 21718557; PubMed Central PMCID: PMC3217800
78. Ming X, Mulvey M, Mohanty S, Patel V. Safety and efficacy of clonidine and clonidine extended-release in the treatment of children and adolescents with attention deficit and hyperactivity disorders. Adolesc Health Med Ther. 2011 Sep 30;2:105-12. doi: 10.2147/AHMT.S15672. eCollection 2011. Review. PubMed PMID: 24600280; PubMed Central PMCID: PMC3926778
79. Transler C, Eilander A, Mitchell S, van de Meer N. The impact of polyunsaturated fatty acids in reducing child attention deficit and hyperactivity disorders. J Atten Disord. 2010 Nov;14(3):232-46. doi: 10.1177/1087054709347250. Epub 2010 Apr 27. Review. PubMed PMID: 20424008
80. Krisanaprakornkit T, Ngamjarus C, Witoonchart C, Piyavhatkul N. Meditation therapies for attention-deficit/hyperactivity disorder (ADHD). Cochrane Database Syst Rev. 2010 Jun 16;(6):CD006507. doi: 10.1002/14651858.CD006507.pub2. Review. PubMed PMID: 20556767
81. Karpouzis F, Bonello R, Pollard H. Chiropractic care for paediatric and adolescent Attention-Deficit/Hyperactivity Disorder: A systematic review. Chiropr Osteopat. 2010 Jun 2;18:13. doi: 10.1186/1746-1340-18-13. PubMed PMID: 20525195; PubMed Central PMCID: PMC2891800
82. Faraone SV, Buitelaar J. Comparing the efficacy of stimulants for ADHD in children and adolescents using meta-analysis. Eur Child Adolesc Psychiatry. 2010 Apr;19(4):353-64. doi: 10.1007/s00787-009-0054-3. Epub 2009 Sep 10. PubMed PMID: 19763664
83. Cornforth C, Sonuga-Barke E, Coghill D. Stimulant drug effects on attention deficit/hyperactivity disorder: a review of the effects of age and sex of patients. Curr Pharm Des. 2010;16(22):2424-33. Review. PubMed PMID: 20513225
84. Bloch MH, Panza KE, Landeros-Weisenberger A, Leckman JF. Meta-analysis: treatment of attention-deficit/hyperactivity disorder in children with comorbid tic disorders. J Am Acad Child Adolesc Psychiatry. 2009 Sep;48(9):884-93. doi: 10.1097/CHI.0b013e3181b26e9f. PubMed PMID: 19625978; PubMed Central PMCID: PMC3943246
85. Raz R, Gabis L. Essential fatty acids and attention-deficit-hyperactivity disorder: a systematic review. Dev Med Child Neurol. 2009 Aug;51(8):580-92. doi: 10.1111/j.1469-8749.2009.03351.x. Epub 2009 Jun 22. Review. PubMed PMID:19549202
86. Lan Y, Zhang LL, Luo R. Attention deficit hyperactivity disorder in children: comparative efficacy of traditional Chinese medicine and methylphenidate. J Int Med Res. 2009 May-Jun;37(3):939-48. PubMed PMID: 19589280
87. Fabiano GA, Pelham WE Jr, Coles EK, Gnagy EM, Chronis-Tuscano A, O'Connor BC. A meta-analysis of behavioral treatments for attention-deficit/hyperactivity disorder. Clin Psychol Rev. 2009 Mar;29(2):129-40. doi:10.1016/j.cpr.2008.11.001. Epub 2008 Nov 11. PubMed PMID: 19131150
88. Faraone SV. Using Meta-analysis to Compare the Efficacy of Medications for Attention-Deficit/Hyperactivity Disorder in Youths. P T. 2009 Dec;34(12):678-94. PubMed PMID: 20140141; PubMed Central PMCID: PMC2810184
89. Kratochvil CJ, Milton DR, Vaughan BS, Greenhill LL. Acute atomoxetine treatment of younger and older children with ADHD: a meta-analysis of tolerability and efficacy. Child Adolesc Psychiatry Ment Health. 2008 Sep 15;2(1):25. doi: 10.1186/1753-2000-2-25. PubMed PMID: 18793405; PubMed Central PMCID: PMC2556311
90. Faraone SV, Biederman J, Morley CP, Spencer TJ. Effect of stimulants on height and weight: a review of the literature. J Am Acad Child Adolesc Psychiatry. 2008 Sep;47(9):994-1009. doi: 10.1097/CHI.ObO13e31817eOea7. Review. PubMed PMID: 18580502
91. Van der Oord S, Prins PJ, Oosterlaan J, Emmelkamp PM. Efficacy of methylphenidate, psychosocial treatments and their combination in school-aged children with ADHD: a meta-analysis. Clin Psychol Rev. 2008 Jun;28(5):783-800. Epub 2007 Nov 6. PubMed PMID: 18068284
92. Toplak ME, Connors L, Shuster J, Knezevic B, Parks S. Review of cognitive, cognitive-behavioral, and neural-based interventions for Attention-Deficit/Hyperactivity Disorder (ADHD). Clin Psychol Rev. 2008 Jun;28(5):801-23. Epub 2007 Nov 5. Review. PubMed PMID: 18061324
93. Pelham WE Jr, Fabiano GA. Evidence-based psychosocial treatments for attention-deficit/hyperactivity disorder. J Clin Child Adolesc Psychol. 2008 Jan;37(1):184-214. doi: 10.1080/15374410701818681. Review. PubMed PMID: 18444058
94. Heirs M, Dean ME. Homeopathy for attention deficit/hyperactivity disorder or hyperkinetic disorder. Cochrane Database Syst Rev. 2009;1. Review. PubMed PMID: 17943868
95. Cheng JY, Chen RY, Ko JS, Ng EM. Efficacy and safety of atomoxetine for attention-deficit/hyperactivity disorder in children and adolescents-meta-analysis and meta-regression analysis. Psychopharmacology (Berl). 2007 Oct;194(2):197-209. Epub 2007 Jun 16. PubMed PMID: 17572882
96. Faraone SV, Biederman J, Spencer TJ, Aleardi M. Comparing the efficacy of medications for ADHD using meta-analysis. MedGenMed. 2006 Oct 5;8(4):4. PubMed PMID: 17415287; PubMed Central PMCID: PMC1868385
97. King S, Griffin S, Hodges Z, Weatherly H, Asseburg C, Richardson G, Golder S, Taylor E, Drummond M, Riemsma R. A systematic review and economic model of the effectiveness and cost-effectiveness of methylphenidate, dexamfetamine and atomoxetine for the treatment of attention deficit hyperactivity disorder in children and adolescents. Health Technol Assess. 2006 Jul;10(23):iii-iv,xiii-146. Review. PubMed PMID: 16796929
98. Gibson AP, Bettinger TL, Patel NC, Crismon ML. Atomoxetine versus stimulants for treatment of attention deficit/hyperactivity disorder. Ann Pharmacother. 2006 Jun;40(6):1134-42. Epub 2006 May 30. Review. PubMed PMID: 16735655
99. Turner D. A review of the use of modafinil for attention-deficit hyperactivity disorder. Expert Rev Neurother. 2006 Apr;6(4):455-68. Review. PubMed PMID: 16623645
100. Bjornstad G, Montgomery P. Family therapy for attention-deficit disorder or attention-deficit/hyperactivity disorder in children and adolescents. Cochrane Database Syst Rev. 2005 Apr 18;(2):CD005042. Review. PubMed PMID: 15846741
101. Maia CR, Cortese S, Caye A, Deakin TK, Polanczyk GV, Polanczyk CA, Rohde LA. Long-Term Efficacy of Methylphenidate Immediate-Release for the Treatment of Childhood ADHD: A Systematic Review and Meta-Analysis. J Atten Disord. 2014 Dec 10. pii: 1087054714559643. [Epub ahead of print] PubMed PMID: 25501355
102. Zaso MJ, Park A, Antshel KM. Treatments for Adolescents With Comorbid ADHD and Substance Use Disorder: A Systematic Review. J Atten Disord. 2015 Feb 5. pii: 1087054715569280. [Epub ahead of print] PubMed PMID: 25655767
103. Aagaard L, Hansen EH. The occurrence of adverse drug reactions reported for attention deficit hyperactivity disorder (ADHD) medications in the pediatric population: a qualitative review of empirical studies. Neuropsychiatr Dis Treat. 2011;7:729-44. doi: 10.2147/NDT.S26403. Epub 2011 Dec 16. PubMed PMID: 22247615; PubMed Central PMCID: PMC3256000
104. Cunill R, Castells X, Tobias A, Capellà D. Pharmacological treatment of attention deficit hyperactivity disorder with co-morbid drug dependence. J Psychopharmacol. 2015 Jan;29(1):15-23. doi: 10.1177/0269881114544777. Epub 2014 Aug 20. PubMed PMID: 25142405
105. Willis WG, Weyandt LL, Lubiner AG, Schubart CD. Neurofeedback as a treatment for attention-deficit/hyperactivity disorder: A systematic review of evidence for practice. J Appl Sch Psychol. 2011;27:201-27
106. Banaschewski T, Coghill D, Santosh P, Zuddas A, Asherson P, Buitelaar J, Danckaerts M, Döpfner M, Faraone SV, Rothenberger A, Sergeant J, Steinhausen HC, Sonuga-Barke EJ, Taylor E. Long-acting medications for the hyperkinetic disorders. A systematic review and European treatment guideline. Eur Child Adolesc Psychiatry. 2006 Dec;15(8):476-95. Epub 2006 May 5. Review. PubMed PMID: 16680409
107. Cooper RE, Tye C, Kuntsi J, Vassos E, Asherson P. The effect of omega-3 polyunsaturated fatty acid supplementation on emotional dysregulation, oppositional behaviour and conduct problems in ADHD: A systematic review and meta-analysis. J Affect Disord. 2015 Oct 29;190:474-482. doi:10.1016/j.jad.2015.09.053. [Epub ahead of print] Review. PubMed PMID: 26551407.
108. Hariri M, Azadbakht L. Magnesium, Iron, and Zinc Supplementation for the Treatment of Attention Deficit Hyperactivity Disorder: A Systematic Review on the Recent Literature. Int J Prev Med. 2015 Sep 2;6:83. doi:10.4103/2008-7802.164313. eCollection 2015. Review. PubMed PMID: 26445630; PubMed Central PMCID: PMC4587068.
109. Maneeton N, Maneeton B, Woottiluk P, Suttajit S, Likhitsathian S, Charnsil C, Srisurapanont M. Comparative efficacy, acceptability, and tolerability of dexmethylphenidate versus placebo in child and adolescent ADHD: a meta-analysis of randomized controlled trials. Neuropsychiatr Dis Treat. 2015 Nov 25;11:2943-52. doi: 10.2147/NDT.S91765. eCollection 2015. PubMed PMID: 26648726; PubMed Central PMCID: PMC4664521
110. Storebø OJ, Krogh HB, Ramstad E, Moreira-Maia CR, Holmskov M, Skoog M, Nilausen TD, Magnusson FL, Zwi M, Gillies D, Rosendal S, Groth C, Rasmussen KB, Gauci D, Kirubakaran R, Forsbøl B, Simonsen E, Gluud C. Methylphenidate for attention-deficit/hyperactivity disorder in children and adolescents: Cochrane systematic review with meta-analyses and trial sequential analyses of randomised clinical trials. BMJ. 2015 Nov 25;351:h5203. doi: 10.1136/bmj.h5203. PubMed PMID: 26608309; PubMed Central PMCID: PMC4659414
111. Storebø OJ, Ramstad E, Krogh HB, Nilausen TD, Skoog M, Holmskov M, Rosendal S, Groth C, Magnusson FL, Moreira-Maia CR, Gillies D, Buch Rasmussen K, Gauci D, Zwi M, Kirubakaran R, Forsbøl B, Simonsen E, Gluud C. Methylphenidate for children and adolescents with attention deficit hyperactivity disorder (ADHD). Cochrane Database Syst Rev. 2015 Nov 25;11:CD009885. Review. PubMed PMID: 26599576.
112. Kidwell KM, Van Dyk TR, Lundahl A, Nelson TD. Stimulant Medications and Sleep for Youth With ADHD: A Meta-analysis. Pediatrics. 2015 Dec;136(6):1144-53. doi:10.1542/peds.2015-1708. Review. PubMed PMID: 26598454.
113. Punja S, Shamseer L, Hartling L, Urichuk L, Vandermeer B, Nikles J, Vohra S. Amphetamines for attention deficit hyperactivity disorder (ADHD) in children and adolescents. Cochrane Database Syst Rev. 2016 Feb 4;2:CD009996. [Epub ahead of print] Review. PubMed PMID: 26844979
114. Vysniauske R, Verburgh L, Oosterlaan J, Molendijk ML. The Effects of Physical Exercise on Functional Outcomes in the Treatment of ADHD: A Meta-Analysis. J Atten Disord. 2016 Feb 9. pii: 1087054715627489. [Epub ahead of print] PubMed PMID: 26861158
115. Battagliese G, Caccetta M, Luppino OI, Baglioni C, Cardi V, Mancini F, Buonanno C. Cognitive-behavioral therapy for externalizing disorders: A meta-analysis of treatment effectiveness. Behav Res Ther. 2015 Dec;75:60-71. doi: 10.1016/j.brat.2015.10.008. Epub 2015 Nov 3. PubMed PMID: 26575979
116. Cairncross M, Miller CJ. The Effectiveness of Mindfulness-Based Therapies for ADHD: A Meta-Analytic Review. J Atten Disord. 2016 Feb 2. pii: 1087054715625301. [Epub ahead of print] PubMed PMID: 26838555
