# Supplementary material for: The pharmacological and non-pharmacological treatment of attention deficit hyperactivity disorder in children and adolescents: A systematic review with network meta-analyses of randomised trials
Source: PLoS One. 2017 Jul 12;12(7):e0180355. doi: 10.1371/journal.pone.0180355 (PMC5507500; doi:10.1371/journal.pone.0180355)
Supplement: S4 Text — (DOCX) [file pone.0180355.s005.docx]

**S4 Text. Reference list of all included studies (190 studies described in 264 publications)**

1. Gittelman-Klein R, Klein DF, Katz S, Saraf K, Pollack E. Comparative effects of methylphenidate and thioridazine in hyperkinetic children. I. Clinical results. Arch Gen Psychiatry. 1976 Oct;33(10):1217-31. PubMed PMID: 971031
2. Firestone P, Crowe D, Goodman JT, McGrath P. Vicissitudes of follow-up studies: differential effects of parent training and stimulant medication with hyperactives. Am J Orthopsychiatry. 1986 Apr;56(2):184-94. PubMed PMID: 3706498
3. Casat CD, Pleasants DZ, Van Wyck Fleet J. A double-blind trial of bupropion in children with attention deficit disorder. Psychopharmacol Bull. 1987;23(1):120-2. PubMed PMID: 3110853
4. Casat CD, Pleasants DZ, Schroeder DH, Parler DW. Bupropion in children with attention deficit disorder. Psychopharmacol Bull. 1989;25(2):198-201. PubMed PMID: 2513592
5. Kupietz SS, Winsberg BG, Richardson E, Maitinsky S, Mendell N. Effects of methylphenidate dosage in hyperactive reading-disabled children: I. Behavior and cognitive performance effects. J Am Acad Child Adolesc Psychiatry. 1988 Jan;27(1):70-7. PubMed PMID: 3343209.
6. Biederman J, Baldessarini RJ, Wright V, Knee D, Harmatz JS. A double-blind placebo controlled study of desipramine in the treatment of ADD: I. Efficacy. J Am Acad Child Adolesc Psychiatry. 1989 Sep;28(5):777-84. PubMed PMID: 2676967
7. Biederman J, Baldessarini RJ, Wright V, Knee D, Harmatz JS, Goldblatt A. A double-blind placebo controlled study of desipramine in the treatment ADD: II. Serum drug levels and cardiovascular findings. J Am Acad Child Adolesc Psychiatry. 1989 Nov;28(6):903-11. PubMed PMID: 2808261
8. Egger J, Stolla A, McEwen LM. Controlled trial of hyposensitisation in children with food-induced hyperkinetic syndrome. Lancet. 1992 May 9;339(8802):1150-3. PubMed PMID: 1349376
9. Gunning WB. A Controlled Trial of Clonidine in Hyperkinetic Children. Erasmus University Rotterdam; 1992. Retrieved from <http://hdl.handle.net/1765/8339> [date last consultation 26 November 2015]
10. Pisterman S, Firestone P, McGrath P, Goodman JT, Webster I, Mallory R, Goffin B. The role of parent training in treatment of preschoolers with ADDH. Am J Orthopsychiatry. 1992 Jul;62(3):397-408. PubMed PMID: 1497105
11. Buitelaar JK, Van der Gaag R, Swaab-Barneveld H, Kuiper M. Pindolol and methylphenidate in children with attention-deficit hyperactivity disorder: clinical efficacy and side-effects. J Child Psychol Psychiatry 1996;37(5):587-95
12. Conners CK, Casat CD, Gualtieri CT, Weller E, Reader M, Reiss A, Weller RA, Khayrallah M, Ascher J. Bupropion hydrochloride in attention deficit disorder with hyperactivity. J Am Acad Child Adolesc Psychiatry. 1996 Oct;35(10):1314-21. PubMed PMID: 8885585
13. Schachar RJ, Tannock R, Cunningham C, Corkum PV. Behavioral, situational, and temporal effects of treatment of ADHD with methylphenidate. J Am Acad Child Adolesc Psychiatry. 1997 Jun;36(6):754-63. PubMed PMID: 9183129
14. Klein RG, Abikoff H. Behavior therapy and methylphenidate in the treatment of children with ADHD. J Atten Disord. 1997;2:89-114
15. van der Meere J, Gunning B, Stemerdink N. The effect of methylphenidate and clonidine on response inhibition and state regulation in children with ADHD. J Child Psychol Psychiatry. 1999 Feb;40(2):291-8. PubMed PMID: 10188712
16. MTA Cooperative Group. A 14-month randomized clinical trial of treatment strategies for attention-deficit/hyperactivity disorder. The MTA Cooperative Group. Multimodal Treatment Study of Children with ADHD. Arch Gen Psychiatry. 1999 Dec;56(12):1073-86. PubMed PMID: 10591283
17. Conners CK, Epstein JN, March JS, Angold A, Wells KC, Klaric J, Swanson JM, Arnold LE, Abikoff HB, Elliott GR, Greenhill LL, Hechtman L, Hinshaw SP, Hoza B, Jensen PS, Kraemer HC, Newcorn JH, Pelham WE, Severe JB, Vitiello B, Wigal T. Multimodal treatment of ADHD in the MTA: an alternative outcome analysis. J Am Acad Child Adolesc Psychiatry. 2001 Feb;40(2):159-67. PubMed PMID: 11211364
18. Swanson JM, Kraemer HC, Hinshaw SP, Arnold LE, Conners CK, Abikoff HB, Clevenger W, Davies M, Elliott GR, Greenhill LL, Hechtman L, Hoza B, Jensen PS, March JS, Newcorn JH, Owens EB, Pelham WE, Schiller E, Severe JB, Simpson S, Vitiello B, Wells K, Wigal T, Wu M. Clinical relevance of the primary findings of the MTA: success rates based on severity of ADHD and ODD symptoms at the end of treatment. J Am Acad Child Adolesc Psychiatry. 2001 Feb;40(2):168-79. PubMed PMID: 11211365
19. Arnold LE, Chuang S, Davies M, Abikoff HB, Conners CK, Elliott GR, Greenhill LL, Hechtman L, Hinshaw SP, Hoza B, Jensen PS, Kraemer HC, Langworthy-Lam KS, March JS, Newcorn JH, Pelham WE, Severe JB, Swanson JM, Vitiello B, Wells KC, Wigal T. Nine months of multicomponent behavioral treatment for ADHD and effectiveness of MTA fading procedures. J Abnorm Child Psychol. 2004 Feb;32(1):39-51. PubMed PMID: 14998110
20. Connor DF, Barkley RA, Davis HT. A pilot study of methylphenidate, clonidine, or the combination in ADHD comorbid with aggressive oppositional defiant or conduct disorder. Clin Pediatr (Phila). 2000 Jan;39(1):15-25. PubMed PMID: 10660814
21. Pliszka SR, Browne RG, Olvera RL, Wynne SK. A double-blind, placebo-controlled study of Adderall and methylphenidate in the treatment of attention-deficit/hyperactivity disorder. J Am Acad Child Adolesc Psychiatry. 2000 May;39(5):619-26. PubMed PMID: 10802980
22. Prince JB, Wilens TE, Biederman J, Spencer TJ, Millstein R, Polisner DA, Bostic JQ. A controlled study of nortriptyline in children and adolescents with attention deficit hyperactivity disorder. J Child Adolesc Psychopharmacol. 2000 Fall;10(3):193-204. PubMed PMID: 11052409
23. Michelson D, Faries D, Wernicke J, Kelsey D, Kendrick K, Sallee FR, Spencer T; Atomoxetine ADHD Study Group. Atomoxetine in the treatment of children and adolescents with attention-deficit/hyperactivity disorder: a randomized, placebo controlled, dose-response study. Pediatrics. 2001 Nov;108(5):E83. PubMed PMID: 11694667
24. Clinical Study Summary ID#3468 - Study B4Z-MC-LYAC: A Phase 3 Randomized, Double-blind, Placebo-Controlled Efficacy and Safety Comparison of Fixed-Dose Ranges (mg/kg/day) of Tomoxetine with Placebo in Child and Adolescent Outpatients with ADHD, Aged 8 to 18 Years. Eli Lilly and Company, 2004 (pp. 267-289). Available at: <http://www.lillytrials.com/results/Strattera.pdf> (date last access: December 1^st^, 2015)
25. FDA Drug Approval Package. Medical Review for Strattera (Atomoxetine Hydrochloride) Capsules. Company:  Eli Lilly & Co. Application No.:  21-411. Available at: <http://www.accessdata.fda.gov/drugsatfda_docs/nda/2002/21-411_Strattera.cfm> (date last access: December 1^st^, 2015)
26. Scahill L, Chappell PB, Kim YS, Schultz RT, Katsovich L, Shepherd E, Arnsten AF, Cohen DJ, Leckman JF. A placebo-controlled study of guanfacine in the treatment of children with tic disorders and attention deficit hyperactivity disorder. Am J Psychiatry. 2001 Jul;158(7):1067-74. PubMed PMID: 11431228
27. Sonuga-Barke EJ, Daley D, Thompson M, Laver-Bradbury C, Weeks A. Parent-based therapies for preschool attention-deficit/hyperactivity disorder: a randomized, controlled trial with a community sample. J Am Acad Child Adolesc Psychiatry. 2001 Apr;40(4):402-8. PubMed PMID: 11314565
28. Voigt RG, Llorente AM, Jensen CL, Fraley JK, Berretta MC, Heird WC. A randomized, double-blind, placebo-controlled trial of docosahexaenoic acid supplementation in children with attention-deficit/hyperactivity disorder. J Pediatr. 2001 Aug;139(2):189-96. PubMed PMID: 11487742
29. Wolraich ML, Greenhill LL, Pelham W, Swanson J, Wilens T, Palumbo D, Atkins M, McBurnett K, Bukstein O, August G. Randomized, controlled trial of oros methylphenidate once a day in children with attention-deficit/hyperactivity disorder. Pediatrics. 2001 Oct;108(4):883-92. PubMed PMID: 11581440
30. Biederman J, Heiligenstein JH, Faries DE, Galil N, Dittmann R, Emslie GJ, Kratochvil CJ, Laws HF, Schuh KJ; Atomoxetine ADHD Study Group. Efficacy of atomoxetine versus placebo in school-age girls with attention-deficit/hyperactivity disorder. Pediatrics. 2002 Dec;110(6):e75. PubMed PMID: 12456942
31. Biederman J, Lopez FA, Boellner SW, Chandler MC. A randomized, double-blind, placebo-controlled, parallel-group study of SLI381 (Adderall XR) in children with attention-deficit/hyperactivity disorder. Pediatrics. 2002 Aug;110(2 Pt 1):258-66. PubMed PMID: 12165576
32. Findling RL, Biederman J, Wilens TE, Spencer TJ, McGough JJ, Lopez FA, Tulloch SJ; SLI381.301 and .302 Study Groups. Short- and long-term cardiovascular effects of mixed amphetamine salts extended release in children. J Pediatr. 2005 Sep;147(3):348-54. PubMed PMID: 16182674
33. Bor W, Sanders MR, Markie-Dadds C. The effects of the Triple P-Positive Parenting Program on preschool children with co-occurring disruptive behavior and attentional/hyperactive difficulties. J Abnorm Child Psychol. 2002 Dec;30(6):571-87. PubMed PMID: 12481972
34. Greenhill LL, Findling RL, Swanson JM; ADHD Study Group. A double-blind, placebo-controlled study of modified-release methylphenidate in children with attention-deficit/hyperactivity disorder. Pediatrics. 2002 Mar;109(3):E39. PubMed PMID: 11875167
35. Lehmkuhl G. Placebo-controlled, double-blind multicenter trial on the efficacy of sustained-release methylphenidate in children suffering from attention deficit hyperactivity disorder (ADHD), Phase III. Integrated Final Report. Institut für medizinische Informatik, Biometrie und Epidemiologie, Universitätsklinikum Essen, Trial no 6520-9979-02, 1-9 2002
36. Döpfner M, Bonaschewski T, Schmidt J, Uebel H, Schmeck K, Gerber WD, et al. Long-acting methylphenidate preparation in children with ADHD - a multicenter study. Nervenheilkd 2003;22:85-92
37. Sinzig J, Döpfner M, Lehmkuhl G; German Methylphenidate Study Group, Uebel H, Schmeck K, Poustka F, Gerber WD, Günter M, Knölker U, Gehrke M, Hässler F, Resch F, Brünger M, Ose C, Fischer R. Long-acting methylphenidate has an effect on aggressive behavior in children with attention-deficit/hyperactivity disorder. J Child Adolesc Psychopharmacol. 2007 Aug;17(4):421-32. PubMed PMID: 17822338
38. Kratochvil CJ, Heiligenstein JH, Dittmann R, Spencer TJ, Biederman J, Wernicke J, Newcorn JH, Casat C, Milton D, Michelson D. Atomoxetine and methylphenidate treatment in children with ADHD: a prospective, randomized, open-label trial. J Am Acad Child Adolesc Psychiatry. 2002 Jul;41(7):776-84. PubMed PMID: 12108801
39. Clinical Study Summary: Summary ID#1855 - Study B4Z-MC-HFBE: Randomized, Double-Blind, Placebo-Controlled, Variable Discontinuation Study of Tomoxetine Hydrochloride in Children with Attention-Deficit/Hyperactivity Disorder. Eli Lilly and Company, 2004 (pp. 51-70). Available at: <http://www.lillytrials.com/results/Strattera.pdf> (date last access: December 12th, 2015)
40. Michelson D, Allen AJ, Busner J, Casat C, Dunn D, Kratochvil C, Newcorn J, Sallee FR, Sangal RB, Saylor K, West S, Kelsey D, Wernicke J, Trapp NJ, Harder D. Once-daily atomoxetine treatment for children and adolescents with attention deficit hyperactivity disorder: a randomized, placebo-controlled study. Am J Psychiatry. 2002 Nov;159(11):1896-901. PubMed PMID: 12411225
41. Clinical Study Summary: Summary ID#2746 - Study B4Z-MC-LYAT: Efficacy, Tolerability, and Safety of Once-Daily Atomoxetine Hydrochloride versus Placebo in Children with Attention-Deficit/Hyperactivity Disorder. Eli Lilly and Company, 2007 (pp. 251-66). Available at: <http://www.lillytrials.com/results/Strattera.pdf> (date last access: December 14th, 2015)
42. Spencer T, Heiligenstein JH, Biederman J, Faries DE, Kratochvil CJ, Conners CK, Potter WZ. Results from 2 proof-of-concept, placebo-controlled studies of atomoxetine in children with attention-deficit/hyperactivity disorder. J Clin Psychiatry. 2002 Dec;63(12):1140-7. PubMed PMID: 12523874
43. Spencer T, Biederman J, Coffey B, Geller D, Crawford M, Bearman SK, Tarazi R, Faraone SV. A double-blind comparison of desipramine and placebo in children and adolescents with chronic tic disorder and comorbid attention-deficit/hyperactivity disorder. Arch Gen Psychiatry. 2002 Jul;59(7):649-56. PubMed PMID: 12090818
44. Tourette's Syndrome Study Group. Treatment of ADHD in children with tics: a randomized controlled trial. Neurology. 2002 Feb 26;58(4):527-36. PubMed PMID: 11865128
45. Van Oudheusden LJ, Scholte HR. Efficacy of carnitine in the treatment of children with attention-deficit hyperactivity disorder. Prostaglandins Leukot Essent Fatty Acids. 2002 Jul;67(1):33-8. PubMed PMID: 12213433
46. Hazell PL, Stuart JE. A randomized controlled trial of clonidine added to psychostimulant medication for hyperactive and aggressive children. J Am Acad Child Adolesc Psychiatry. 2003 Aug;42(8):886-94. PubMed PMID: 12874489
47. Rugino TA, Samsock TC. Modafinil in children with attention-deficit hyperactivity disorder. Pediatr Neurol. 2003 Aug;29(2):136-42. PubMed PMID: 14580657
48. Abikoff H, Hechtman L, Klein RG, Weiss G, Fleiss K, Etcovitch J, Cousins L, Greenfield B, Martin D, Pollack S. Symptomatic improvement in children with ADHD treated with long-term methylphenidate and multimodal psychosocial treatment. J Am Acad Child Adolesc Psychiatry. 2004 Jul;43(7):802-11. PubMed PMID: 15213581
49. Abikoff H, Hechtman L, Klein RG, Gallagher R, Fleiss K, Etcovitch J, Cousins L, Greenfield B, Martin D, Pollack S. Social functioning in children with ADHD treated with long-term methylphenidate and multimodal psychosocial treatment. J Am Acad Child Adolesc Psychiatry. 2004 Jul;43(7):820-9. PubMed PMID: 15213583
50. Klein RG, Abikoff H, Hechtman L, Weiss G. Design and rationale of controlled study of long-term methylphenidate and multimodal psychosocial treatment in children with ADHD. J Am Acad Child Adolesc Psychiatry. 2004 Jul;43(7):792-801
51. Akhondzadeh S, Mohammadi MR, Khademi M. Zinc sulfate as an adjunct to methylphenidate for the treatment of attention deficit hyperactivity disorder in children: a double blind and randomized trial [ISRCTN64132371]. BMC Psychiatry. 2004 Apr 8;4:9. PubMed PMID: 15070418; PubMed Central PMCID: PMC400741
52. Bilici M, Yildirim F, Kandil S, Bekaroğlu M, Yildirmiş S, Değer O, Ulgen M, Yildiran A, Aksu H. Double-blind, placebo-controlled study of zinc sulfate in the treatment of attention deficit hyperactivity disorder. Prog Neuropsychopharmacol Biol Psychiatry. 2004 Jan;28(1):181-90. PubMed PMID: 14687872
53. Döpfner M, Breuer D, Schürmann S, Metternich TW, Rademacher C, Lehmkuhl G. Effectiveness of an adaptive multimodal treatment in children with Attention-Deficit Hyperactivity Disorder -- global outcome. Eur Child Adolesc Psychiatry. 2004;13 Suppl 1:I117-29. PubMed PMID: 15322962
54. Kaplan S, Heiligenstein J, West S, Busner J, Harder D, Dittmann R, Casat C, Wernicke JF. Efficacy and safety of atomoxetine in childhood attention-deficit/hyperactivity disorder with comorbid oppositional defiant disorder. J Atten Disord. 2004 Oct;8(2):45-52. PubMed PMID: 15801334
55. Kelsey DK, Sumner CR, Casat CD, Coury DL, Quintana H, Saylor KE, Sutton VK, Gonzales J, Malcolm SK, Schuh KJ, Allen AJ. Once-daily atomoxetine treatment for children with attention-deficit/hyperactivity disorder, including an assessment of evening and morning behavior: a double-blind, placebo-controlled trial. Pediatrics. 2004 Jul;114(1):e1-8. PubMed PMID: 15231966
56. Clinical Study Summary: Summary ID#5670 – Study B4Z-US-LYBG: A Randomized, Double-Blind, Placebo-Controlled Trial of Once Daily Atomoxetine Hydrochloride to Evaluate Efficacy in the Treatment of Attention-Deficit/Hyperactivity Disorder in Children Ages 6 through 12 with an Assessment of Evening Behavior. Eli Lilly and Company, 2004 (pp. 630-658). Available at: <http://www.lillytrials.com/results/Strattera.pdf> (date last access: December 29th, 2015)
57. Michelson D, Buitelaar JK, Danckaerts M, Gillberg C, Spencer TJ, Zuddas A, Faries DE, Zhang S, Biederman J. Relapse prevention in pediatric patients with ADHD treated with atomoxetine: a randomized, double-blind, placebo-controlled study. J Am Acad Child Adolesc Psychiatry. 2004 Jul;43(7):896-904. PubMed PMID: 15213591
58. Clinical Study Summary: Summary ID#2552 – Study B4Z-MC-LYAF: Relapse Prevention after 10-Week and 52-Week Treatment with Tomoxetine Hydrochloride in Children with Attention-Deficit/Hyperactivity Disorder. Eli Lilly and Company, 2007 (pp. 82-202). Available at: <http://www.lillytrials.com/results/Strattera.pdf> (date last access: December 29th, 2015)
59. Wigal S, Swanson JM, Feifel D, Sangal RB, Elia J, Casat CD, Zeldis JB, Conners CK. A double-blind, placebo-controlled trial of dexmethylphenidate hydrochloride and d,l-threo-methylphenidate hydrochloride in children with attention-deficit/hyperactivity disorder. J Am Acad Child Adolesc Psychiatry. 2004 Nov;43(11):1406-14. PubMed PMID: 15502600
60. Allen AJ, Kurlan RM, Gilbert DL, Coffey BJ, Linder SL, Lewis DW, Winner PK, Dunn DW, Dure LS, Sallee FR, Milton DR, Mintz MI, Ricardi RK, Erenberg G, Layton LL, Feldman PD, Kelsey DK, Spencer TJ. Atomoxetine treatment in children and adolescents with ADHD and comorbid tic disorders. Neurology. 2005 Dec 27;65(12):1941-9. PubMed PMID: 16380617
61. Clinical Study Summary: Summary ID#4908 – Study B4Z-MC-LYAS: A Randomized, Double-Blind Study of Tomoxetine Hydrochloride and Placebo in Pediatric Outpatients with Attention-Deficit/Hyperactivity Disorder and Comorbid Tic Disorders. Eli Lilly and Company, 2005 (pp. 358-384). Available at: <http://www.lillytrials.com/results/Strattera.pdf> (date last access: January 5th, 2016)
62. Biederman J, Swanson JM, Wigal SB, Kratochvil CJ, Boellner SW, Earl CQ, Jiang J, Greenhill L. Efficacy and safety of modafinil film-coated tablets in children and adolescents with attention-deficit/hyperactivity disorder: results of a randomized, double-blind, placebo-controlled, flexible-dose study. Pediatrics. 2005 Dec;116(6):e777-84. PubMed PMID: 16322134
63. Jacobs J, Williams AL, Girard C, Njike VY, Katz D. Homeopathy for attention-deficit/hyperactivity disorder: a pilot randomized-controlled trial. J Altern Complement Med. 2005 Oct;11(5):799-806. PubMed PMID: 16296913
64. Kemner JE, Starr HL, Ciccone PE, Hooper-Wood CG, Crockett RS. Outcomes of OROS methylphenidate compared with atomoxetine in children with ADHD: a multicenter, randomized prospective study. Adv Ther. 2005 Sep-Oct;22(5):498-512. PubMed PMID: 16418159
65. Klingberg T, Fernell E, Olesen PJ, Johnson M, Gustafsson P, Dahlström K, Gillberg CG, Forssberg H, Westerberg H. Computerized training of working memory in children with ADHD--a randomized, controlled trial. J Am Acad Child Adolesc Psychiatry. 2005 Feb;44(2):177-86. PubMed PMID: 15689731
66. So YC. Effectiveness of methylphenidate and combined treatment (methylphenidate and psychosocial treatment) for chinese children with Attention-Deficit/Hyperactivity Disorder in a Community Mental Health Center (PHD theses). Hong Kong: The Chinese University of Hong Kong, 2005
67. So CY, Leung PW, Hung SF. Treatment effectiveness of combined medication/behavioural treatment with chinese ADHD children in routine practice. Behav Res Ther. 2008 Sep;46(9):983-92. doi: 10.1016/j.brat.2008.06.007. Epub 2008 Jun 27. PubMed PMID: 18692170
68. Starr HL, Kemner J. Multicenter, randomized, open-label study of OROS methylphenidate versus atomoxetine: treatment outcomes in African-American children with ADHD. J Natl Med Assoc. 2005 Oct;97(10 Suppl):11S-16S. PubMed PMID: 16350601
69. Weiss M, Tannock R, Kratochvil C, Dunn D, Velez-Borras J, Thomason C, Tamura R, Kelsey D, Stevens L, Allen AJ. A randomized, placebo-controlled study of once-daily atomoxetine in the school setting in children with ADHD. J Am Acad Child Adolesc Psychiatry. 2005 Jul;44(7):647-55. PubMed PMID: 15968233
70. Clinical Study Summary: Summary ID#5003 – Study B4Z-MC-LYAW: Double-Blind, Placebo-Controlled Trial of Atomoxetine Hydrochloride to Evaluate Efficacy in the School Setting in Children Ages 8 to 12 Years with Attention-Deficit/Hyperactivity Disorder – Acute Treatment Period. Eli Lilly and Company, 2005 (pp. 469-501). Available at: <http://www.lillytrials.com/results/Strattera.pdf> (date last access: January 11th, 2016)
71. Brown RT, Perwien A, Faries DE, Kratochvil CJ, Vaughan BS. Atomoxetine in the management of children with ADHD: effects on quality of life and school functioning. Clin Pediatr (Phila). 2006 Nov;45(9):819-27. PubMed PMID: 17041169
72. Wigal SB, McGough JJ, McCracken JT, Biederman J, Spencer TJ, Posner KL, Wigal TL, Kollins SH, Clark TM, Mays DA, Zhang Y, Tulloch SJ. A laboratory school comparison of mixed amphetamine salts extended release (Adderall XR) and atomoxetine (Strattera) in school-aged children with attention deficit/hyperactivity disorder. J Atten Disord. 2005 Aug;9(1):275-89. PubMed PMID: 16371674
73. Biederman J, Swanson JM, Wigal SB, Boellner SW, Earl CQ, Lopez FA; Modafinil ADHD Study Group. A comparison of once-daily and divided doses of modafinil in children with attention-deficit/hyperactivity disorder: a randomized, double-blind, and placebo-controlled study. J Clin Psychiatry. 2006 May;67(5):727-35. PubMed PMID: 16841622
74. Findling RL, Quinn D, Hatch SJ, Cameron SJ, DeCory HH, McDowell M. Comparison of the clinical efficacy of twice-daily Ritalin and once-daily Equasym XL with placebo in children with Attention Deficit/Hyperactivity Disorder. Eur Child Adolesc Psychiatry. 2006 Dec;15(8):450-9. Epub 2006 Jun 21. PubMed PMID: 16791541
75. Gau SS, Shen HY, Soong WT, Gau CS. An open-label, randomized, active-controlled equivalent trial of osmotic release oral system methylphenidate in children with attention-deficit/hyperactivity disorder in Taiwan. J Child Adolesc Psychopharmacol. 2006 Aug;16(4):441-55. PubMed PMID: 16958569
76. Greenhill LL, Muniz R, Ball RR, Levine A, Pestreich L, Jiang H. Efficacy and safety of dexmethylphenidate extended-release capsules in children with attention-deficit/hyperactivity disorder. J Am Acad Child Adolesc Psychiatry. 2006 Jul;45(7):817-23. PubMed PMID: 16832318
77. Greenhill LL, Biederman J, Boellner SW, Rugino TA, Sangal RB, Earl CQ, Jiang JG, Swanson JM. A randomized, double-blind, placebo-controlled study of modafinil film-coated tablets in children and adolescents with attention-deficit/hyperactivity disorder. J Am Acad Child Adolesc Psychiatry. 2006 May;45(5):503-11. PubMed PMID: 16601402
78. Greenhill L, Kollins S, Abikoff H, McCracken J, Riddle M, Swanson J, McGough J, Wigal S, Wigal T, Vitiello B, Skrobala A, Posner K, Ghuman J, Cunningham C, Davies M, Chuang S, Cooper T. Efficacy and safety of immediate-release methylphenidate treatment for preschoolers with ADHD. J Am Acad Child Adolesc Psychiatry. 2006 Nov;45(11):1284-93. Erratum in: J Am Acad Child Adolesc Psychiatry. 2007 Jan;46(1):141. PubMed PMID: 17023867
79. Wigal T, Greenhill L, Chuang S, McGough J, Vitiello B, Skrobala A, Swanson J, Wigal S, Abikoff H, Kollins S, McCracken J, Riddle M, Posner K, Ghuman J, Davies M, Thorp B, Stehli A. Safety and tolerability of methylphenidate in preschool children with ADHD. J Am Acad Child Adolesc Psychiatry. 2006 Nov;45(11):1294-303. PubMed PMID: 17028508
80. Abikoff HB, Vitiello B, Riddle MA, Cunningham C, Greenhill LL, Swanson JM, Chuang SZ, Davies M, Kastelic E, Wigal SB, Evans L, Ghuman JK, Kollins SH, McCracken JT, McGough JJ, Murray DW, Posner K, Skrobala AM, Wigal T. Methylphenidate effects on functional outcomes in the Preschoolers with Attention-Deficit/Hyperactivity Disorder Treatment Study (PATS). J Child Adolesc Psychopharmacol. 2007 Oct;17(5):581-92. PubMed PMID: 17979579
81. Sangal RB, Owens J, Allen AJ, Sutton V, Schuh K, Kelsey D. Effects of atomoxetine and methylphenidate on sleep in children with ADHD. Sleep. 2006 Dec;29(12):1573-85. PubMed PMID: 17252888
82. Spencer TJ, Wilens TE, Biederman J, Weisler RH, Read SC, Pratt R. Efficacy and safety of mixed amphetamine salts extended release (Adderall XR) in the management of attention-deficit/hyperactivity disorder in adolescent patients: a 4-week, randomized, double-blind, placebo-controlled, parallel-group study. Clin Ther. 2006 Feb;28(2):266-79. PubMed PMID: 16678648
83. Spencer TJ, Abikoff HB, Connor DF, Biederman J, Pliszka SR, Boellner S, Read SC, Pratt R. Efficacy and safety of mixed amphetamine salts extended release (adderall XR) in the management of oppositional defiant disorder with or without comorbid attention-deficit/hyperactivity disorder in school-aged children and adolescents: A 4-week, multicenter, randomized, double-blind, parallel-group, placebo-controlled, forced-dose-escalation study. Clin Ther. 2006 Mar;28(3):402-18. PubMed PMID: 16750455
84. Steele M, Weiss M, Swanson J, Wang J, Prinzo RS, Binder CE. A randomized, controlled effectiveness trial of OROS-methylphenidate compared to usual care with immediate-release methylphenidate in attention deficit-hyperactivity disorder. Can J Clin Pharmacol. 2006 Winter;13(1):e50-62. Epub 2006 Jan 23. PubMed PMID: 16456216
85. Trebatická J, Kopasová S, Hradecná Z, Cinovský K, Skodácek I, Suba J, Muchová J, Zitnanová I, Waczulíková I, Rohdewald P, Duracková Z. Treatment of ADHD with French maritime pine bark extract, Pycnogenol. Eur Child Adolesc Psychiatry. 2006 Sep;15(6):329-35. Epub 2006 May 13. PubMed PMID: 16699814
86. Dvoráková M, Jezová D, Blazícek P, Trebatická J, Skodácek I, Suba J, Iveta W, Rohdewald P, Duracková Z. Urinary catecholamines in children with attention deficit hyperactivity disorder (ADHD): modulation by a polyphenolic extract from pine bark (pycnogenol). Nutr Neurosci. 2007 Jun-Aug;10(3-4):151-7. PubMed PMID: 18019397
87. Armenteros JL, Lewis JE, Davalos M. Risperidone augmentation for treatment-resistant aggression in attention-deficit/hyperactivity disorder: a placebo-controlled pilot study. J Am Acad Child Adolesc Psychiatry. 2007 May;46(5):558-65. PubMed PMID: 17450046
88. Arnold LE, Amato A, Bozzolo H, Hollway J, Cook A, Ramadan Y, Crowl L, Zhang D, Thompson S, Testa G, Kliewer V, Wigal T, McBurnett K, Manos M. Acetyl-L-carnitine (ALC) in attention-deficit/hyperactivity disorder: a multi-site, placebo-controlled pilot trial. J Child Adolesc Psychopharmacol. 2007 Dec;17(6):791-802. doi: 10.1089/cap.2007.018. PubMed PMID: 18315451
89. Atomoxetine ADHD and Comorbid MDD Study Group: Bangs ME, Emslie GJ, Spencer TJ, Ramsey JL, Carlson C, Bartky EJ, Busner J, Duesenberg DA, Harshawat P, Kaplan SL, Quintana H, Allen AJ, Sumner CR. Efficacy and safety of atomoxetine in adolescents with attention-deficit/hyperactivity disorder and major depression. J Child Adolesc Psychopharmacol. 2007 Aug;17(4):407-20. PubMed PMID: 17822337
90. Clinical Study Summary: Summary ID#5004 – Study B4Z-MC-LYAX: A Randomized, Double-Blind, Placebo-Controlled Study of Atomoxetine Hydrochloride in Adolescents with Attention-Deficit/Hyperactivity Disorder and Comorbid Depressive Disorder. Eli Lilly and Company, 2006 (pp. 521-547). Available at: <http://www.lillytrials.com/results/Strattera.pdf> (date last access: January 13th, 2016)
91. Biederman J, Krishnan S, Zhang Y, McGough JJ, Findling RL. Efficacy and tolerability of lisdexamfetamine dimesylate (NRP-104) in children with attention-deficit/hyperactivity disorder: a phase III, multicenter, randomized, double-blind, forced-dose, parallel-group study. Clin Ther. 2007 Mar;29(3):450-63. PubMed PMID: 17577466
92. Lopez FA, Ginsberg LD, Arnold V. Effect of lisdexamfetamine dimesylate on parent-rated measures in children aged 6 to 12 years with attention-deficit/hyperactivity disorder: a secondary analysis. Postgrad Med. 2008 Sep;120(3):89-102. doi: 10.3810/pgm.2008.09.1910. PubMed PMID: 18824828
93. Buitelaar JK, Michelson D, Danckaerts M, Gillberg C, Spencer TJ, Zuddas A, Faries DE, Zhang S, Biederman J. A randomized, double-blind study of continuation treatment for attention-deficit/hyperactivity disorder after 1 year. Biol Psychiatry. 2007 Mar 1;61(5):694-9. Epub 2006 Aug 7. PubMed PMID: 16893523
94. Carlson GA, Dunn D, Kelsey D, Ruff D, Ball S, Ahrbecker L, Allen AJ. A pilot study for augmenting atomoxetine with methylphenidate: safety of concomitant therapy in children with attention-deficit/hyperactivity disorder. Child Adolesc Psychiatry Ment Health. 2007 Sep 27;1(1):10. PubMed PMID: 17897473
95. Gau SS, Huang YS, Soong WT, Chou MC, Chou WJ, Shang CY, Tseng WL, Allen AJ, Lee P. A randomized, double-blind, placebo-controlled clinical trial on once-daily atomoxetine in Taiwanese children and adolescents with attention-deficit/hyperactivity disorder. J Child Adolesc Psychopharmacol. 2007;17(4):447-60. PubMed PMID: 17822340
96. Clinical Study Summary: Summary ID#8160 – Study B4Z-TW-S010: Efficacy, Tolerability, and Safety of Once-Daily Atomoxetine Hydrochloride versus Placebo in Taiwanese Children and Adolescents with Attention-Deficit/Hyperactivity Disorder. Eli Lilly and Company, 2005 (pp. 1328-1375). Available at: <http://www.lillytrials.com/results/Strattera.pdf> (date last access: January 14th, 2016)
97. Geller D, Donnelly C, Lopez F, Rubin R, Newcorn J, Sutton V, Bakken R, Paczkowski M, Kelsey D, Sumner C. Atomoxetine treatment for pediatric patients with attention-deficit/hyperactivity disorder with comorbid anxiety disorder. J Am Acad Child Adolesc Psychiatry. 2007 Sep;46(9):1119-27. PubMed PMID: 17712235
98. Clinical Study Summary: Summary ID#6477 – Study B4Z-US-LYBP: A Randomized, Double-Blind, Placebo-Controlled Study of Atomoxetine Hydrochloride in Children and Adolescents with Attention-Deficit/Hyperactivity Disorder and Comorbid Anxiety. Eli Lilly and Company, 2006 (pp. 872-898). Available at: <http://www.lillytrials.com/results/Strattera.pdf> (date last access: January 14th, 2016)
99. Prasad S, Harpin V, Poole L, Zeitlin H, Jamdar S, Puvanendran K; SUNBEAM Study Group. A multi-centre, randomised, open-label study of atomoxetine compared with standard current therapy in UK children and adolescents with attention-deficit/hyperactivity disorder (ADHD). Curr Med Res Opin. 2007 Feb;23(2):379-94. PubMed PMID: 17288692
100. Clinical Study Summary: Summary ID#6962 – Study B4Z-BP-LYBS: A Randomized, Controlled, Open-Label Study of the Broader Efficacy of Atomoxetine Hydrochloride in the Treatment of Attention-Deficit/Hyperactivity Disorder (ADHD) in Children and Adolescents. Eli Lilly and Company, 2007 (pp. 983-1008). Available at: <http://www.lillytrials.com/results/Strattera.pdf> (date last access: January 15th, 2016)
101. van den Hoofdakker BJ, van der Veen-Mulders L, Sytema S, Emmelkamp PM, Minderaa RB, Nauta MH. Effectiveness of behavioral parent training for children with ADHD in routine clinical practice: a randomized controlled study. J Am Acad Child Adolesc Psychiatry. 2007 Oct;46(10):1263-71. PubMed PMID: 17885567
102. van der Oord S, Prins PJ, Oosterlaan J, Emmelkamp PM. Does brief, clinically based, intensive multimodal behavior therapy enhance the effects of methylphenidate in children with ADHD? Eur Child Adolesc Psychiatry. 2007 Feb;16(1):48-57. Epub 2006 Sep 13. PubMed PMID: 16972117
103. Wang Y, Zheng Y, Du Y, Song DH, Shin YJ, Cho SC, Kim BN, Ahn DH, Marquez-Caraveo ME, Gao H, Williams DW, Levine LR. Atomoxetine versus methylphenidate in paediatric outpatients with attention deficit hyperactivity disorder: a randomized, double-blind comparison trial. Aust N Z J Psychiatry. 2007 Mar;41(3):222-30. PubMed PMID: 17464703
104. Clinical Study Summary: Summary ID#6934 – Study B4Z-MC-LYBR: A Randomized, Double-Blind Comparison, Safety and Efficacy Trial of Atomoxetine Hydrochloride and Methylphenidate Hydrochloride in Pediatric Outpatients with DSM-IV Attention-Deficit/Hyperactivity Disorder. Eli Lilly and Company, 2005 (pp. 952-982). Available at: <http://www.lillytrials.com/results/Strattera.pdf> (date last access: January 16th, 2016)
105. Amiri S, Mohammadi MR, Mohammadi M, Nouroozinejad GH, Kahbazi M, Akhondzadeh S. Modafinil as a treatment for Attention-Deficit/Hyperactivity Disorder in children and adolescents: a double blind, randomized clinical trial. Prog Neuropsychopharmacol Biol Psychiatry. 2008 Jan 1;32(1):145-9. Epub 2007 Aug 8. PubMed PMID: 17765380
106. Bangs ME, Hazell P, Danckaerts M, Hoare P, Coghill DR, Wehmeier PM, Williams DW, Moore RJ, Levine L; Atomoxetine ADHD/ODD Study Group. Atomoxetine for the treatment of attention-deficit/hyperactivity disorder and oppositional defiant disorder. Pediatrics. 2008 Feb;121(2):e314-20. doi: 10.1542/peds.2006-1880. Erratum in: Pediatrics.2008 Jul;122(1): 227. PubMed PMID: 18245404
107. Clinical Study Summary: Summary ID#7068 – Study B4Z-MC-LYBX: A Randomized, Double-Blind Comparison of Atomoxetine Hydrochloride and Placebo in Child and Adolescent Outpatients with Attention-Deficit/Hyperactivity Disorder and Comorbid Oppositional Defiant Disorder. Eli Lilly and Company, 2008 (pp. 1057-1068). Available at: <http://www.lillytrials.com/results/Strattera.pdf> (date last access: January 16th, 2016)
108. Biederman J, Melmed RD, Patel A, McBurnett K, Konow J, Lyne A, Scherer N; SPD503 Study Group. A randomized, double-blind, placebo-controlled study of guanfacine extended release in children and adolescents with attention-deficit/hyperactivity disorder. Pediatrics. 2008 Jan;121(1):e73-84. doi: 10.1542/peds.2006-3695. PubMed PMID: 18166547
109. Study Record Detail in ClinicalTrials.gov: NCT00152009 – Safety and Efficacy of SPD503 in Treating Attention-Deficit/Hyperactivity Disorder (ADHD) in Children Aged 6-17. U.S. National Institutes of Health, 2009. Available at: <https://clinicaltrials.gov/ct2/show/NCT00152009?term=NCT00152009&rank=1> (date last access: January 18th, 2016)
110. Clinical Study Report. Study No: SPD503-301. A phase III, randomized, multi-center, double-blind, parallel-group, placebo-controlled safety and efficacy study of SPD503 in children and adolescents aged 6-17 with attention deficit hyperactivity disorder (ADHD). Shire Plc, 16 March 2006. Document provided on Feb 15^th^ 2016 by the European Medicines Agency (under the Agency policy on access to documents related to medicinal products for human and veterinary use).
111. Palumbo DR, Sallee FR, Pelham WE Jr, Bukstein OG, Daviss WB, McDermott MP. Clonidine for attention-deficit/hyperactivity disorder: I. Efficacy and tolerability outcomes. J Am Acad Child Adolesc Psychiatry. 2008 Feb;47(2):180-8. doi: 10.1097/chi.0b013e31815d9af7. PubMed PMID: 18182963
112. Daviss WB, Patel NC, Robb AS, McDermott MP, Bukstein OG, Pelham WE Jr, Palumbo D, Harris P, Sallee FR. Clonidine for attention-deficit/hyperactivity disorder: II. ECG changes and adverse events analysis. J Am Acad Child Adolesc Psychiatry. 2008 Feb;47(2):189-98. doi: 10.1097/chi.0b013e31815d9ae4. PubMed PMID: 18182964
113. Cannon M, Pelham WH, Sallee FR, Palumbo DR, Bukstein O, Daviss WB. Effects of clonidine and methylphenidate on family quality of life in attention-deficit/hyperactivity disorder. J Child Adolesc Psychopharmacol. 2009 Oct;19(5):511-7. doi: 10.1089/cap.2009.0008. PubMed PMID: 19877975
114. Findling RL, Bukstein OG, Melmed RD, López FA, Sallee FR, Arnold LE, Pratt RD. A randomized, double-blind, placebo-controlled, parallel-group study of methylphenidate transdermal system in pediatric patients with attention-deficit/hyperactivity disorder. J Clin Psychiatry. 2008 Jan;69(1):149-59. Erratum in: J Clin Psychiatry. 2008 Feb;69(2):329. PubMed PMID: 18312050
115. Heriot SA, Evans IM, Foster TM. Critical influences affecting response to various treatments in young children with ADHD: a case series. Child Care Health Dev. 2008 Jan;34(1):121-33. doi: 10.1111/j.1365-2214.2007.00745.x. PubMed PMID: 18171453
116. Konofal E, Lecendreux M, Deron J, Marchand M, Cortese S, Zaïm M, Mouren MC, Arnulf I. Effects of iron supplementation on attention deficit hyperactivity disorder in children. Pediatr Neurol. 2008 Jan;38(1):20-6. PubMed PMID: 18054688
117. Newcorn JH, Kratochvil CJ, Allen AJ, Casat CD, Ruff DD, Moore RJ, Michelson D; Atomoxetine/Methylphenidate Comparative Study Group. Atomoxetine and osmotically released methylphenidate for the treatment of attention deficit hyperactivity disorder: acute comparison and differential response. Am J Psychiatry. 2008 Jun;165(6):721-30. doi: 10.1176/appi.ajp.2007.05091676. Epub 2008 Feb 15. PubMed PMID: 18281409
118. Clinical Study Summary: Summary ID#5831 – Study B4Z-MC-LYBI: A Randomized, Double-Blind Comparison of Atomoxetine Hydrochloride, Extended-Release Methylphenidate Hydrochloride (Concerta), and Placebo in Pediatric Outpatients with DSM-IV Attention-Deficit/Hyperactivity Disorder. Eli Lilly and Company, 2006 (pp. 708-770). Available at: <http://www.lillytrials.com/results/Strattera.pdf> (date last access: January 20th, 2016)
119. Torrioli MG, Vernacotola S, Peruzzi L, Tabolacci E, Mila M, Militerni R, Musumeci S, Ramos FJ, Frontera M, Sorge G, Marzullo E, Romeo G, Vallee L, Veneselli E, Cocchi E, Garbarino E, Moscato U, Chiurazzi P, D'Iddio S, Calvani M, Neri G. A double-blind, parallel, multicenter comparison of L-acetylcarnitine with placebo on the attention deficit hyperactivity disorder in fragile X syndrome boys. Am J Med Genet A. 2008 Apr 1;146A(7):803-12. doi: 10.1002/ajmg.a.32268. PubMed PMID: 18286595
120. Vaisman N, Kaysar N, Zaruk-Adasha Y, Pelled D, Brichon G, Zwingelstein G, Bodennec J. Correlation between changes in blood fatty acid composition and visual sustained attention performance in children with inattention: effect of dietary n-3 fatty acids containing phospholipids. Am J Clin Nutr. 2008 May;87(5):1170-80. PubMed PMID: 18469236
121. Weber W, Vander Stoep A, McCarty RL, Weiss NS, Biederman J, McClellan J. Hypericum perforatum (St John's wort) for attention-deficit/hyperactivity disorder in children and adolescents: a randomized controlled trial. JAMA. 2008 Jun 11;299(22):2633-41. doi: 10.1001/jama.299.22.2633. PubMed PMID: 18544723
122. Arabgol F, Panaghi L, Hebrani P. Reboxetine versus methylphenidate in treatment of children and adolescents with attention deficit-hyperactivity disorder. Eur Child Adolesc Psychiatry. 2009 Jan;18(1):53-9. doi: 10.1007/s00787-008-0705-9. Epub 2008 Jun 18. PubMed PMID: 18563471
123. Block SL, Kelsey D, Coury D, Lewis D, Quintana H, Sutton V, Schuh K, Allen AJ, Sumner C. Once-daily atomoxetine for treating pediatric attention-deficit/hyperactivity disorder: comparison of morning and evening dosing. Clin Pediatr (Phila). 2009 Sep;48(7):723-33. doi: 10.1177/0009922809335321. Epub 2009 May 6. PubMed PMID: 19420182
124. Clinical Study Summary: Summary ID#7972 – Study B4Z-US-LYCC: Evaluation of Continuous Symptom Treatment of ADHD: A Placebo-Controlled Double-Blind Assessment of Morning-Dosed or Evening-Dosed Strattera. Eli Lilly and Company, 2006 (pp. 1198-1234). Available at: <http://www.lillytrials.com/results/Strattera.pdf> (date last access: January 20th, 2016)
125. Childress AC, Spencer T, Lopez F, Gerstner O, Thulasiraman A, Muniz R, Post A. Efficacy and safety of dexmethylphenidate extended-release capsules administered once daily to children with attention-deficit/hyperactivity disorder. J Child Adolesc Psychopharmacol. 2009 Aug;19(4):351-61. doi: 10.1089/cap.2009.0007. PubMed PMID: 19702487
126. Dell'Agnello G, Maschietto D, Bravaccio C, Calamoneri F, Masi G, Curatolo P, Besana D, Mancini F, Rossi A, Poole L, Escobar R, Zuddas A; LYCY Study Group. Atomoxetine hydrochloride in the treatment of children and adolescents with attention-deficit/hyperactivity disorder and comorbid oppositional defiant disorder: A placebo-controlled Italian study. Eur Neuropsychopharmacol. 2009 Nov;19(11):822-34. doi: 10.1016/j.euroneuro.2009.07.008. Epub 2009 Aug 28. PubMed PMID: 19716683
127. Johnson M, Ostlund S, Fransson G, Kadesjö B, Gillberg C. Omega-3/omega-6 fatty acids for attention deficit hyperactivity disorder: a randomized placebo-controlled trial in children and adolescents. J Atten Disord. 2009 Mar;12(5):394-401. doi: 10.1177/1087054708316261. Epub 2008 Apr 30. PubMed PMID: 18448859
128. Kahbazi M, Ghoreishi A, Rahiminejad F, Mohammadi MR, Kamalipour A, Akhondzadeh S. A randomized, double-blind and placebo-controlled trial of modafinil in children and adolescents with attention deficit and hyperactivity disorder. Psychiatry Res. 2009 Aug 15;168(3):234-7. doi: 10.1016/j.psychres.2008.06.024. Epub 2009 May 12. PubMed PMID: 19439364
129. Montoya A, Hervas A, Cardo E, Artigas J, Mardomingo MJ, Alda JA, Gastaminza X, García-Polavieja MJ, Gilaberte I, Escobar R. Evaluation of atomoxetine for first-line treatment of newly diagnosed, treatment-naïve children and adolescents with attention deficit/hyperactivity disorder. Curr Med Res Opin. 2009 Nov;25(11):2745-54. doi: 10.1185/03007990903316152. PubMed PMID: 1978551
130. Study Record Detail in ClinicalTrials.gov: NCT00191945 – Efficacy and Safety of Atomoxetine in Children with Recent Diagnosis of Attention-Deficit/Hyperactivity Disorder (ADHD). U.S. National Institutes of Health, 2010. Available at: <https://clinicaltrials.gov/ct2/show/results/NCT00191945> (date last access: January 20th, 2016)
131. Nair V, Mahadevan S. Randomised controlled study-efficacy of clonidine versus carbamazepine in children with ADHD. J Trop Pediatr. 2009 Apr;55(2):116-21. doi: 10.1093/tropej/fmn117. Epub 2009 Feb 8. PubMed PMID: 19203986
132. Pelsser LM, Frankena K, Toorman J, Savelkoul HF, Pereira RR, Buitelaar JK. A randomised controlled trial into the effects of food on ADHD. Eur Child Adolesc Psychiatry. 2009 Jan;18(1):12-9. doi: 10.1007/s00787-008-0695-7. Epub 2008 Apr 21. PubMed PMID: 18431534
133. Raz R, Carasso RL, Yehuda S. The influence of short-chain essential fatty acids on children with attention-deficit/hyperactivity disorder: a double-blind placebo-controlled study. J Child Adolesc Psychopharmacol. 2009 Apr;19(2):167-77. doi: 10.1089/cap.2008.070. PubMed PMID: 19364294
134. Sallee FR, McGough J, Wigal T, Donahue J, Lyne A, Biederman J; SPD503 STUDY GROUP. Guanfacine extended release in children and adolescents with attention-deficit/hyperactivity disorder: a placebo-controlled trial. J Am Acad Child Adolesc Psychiatry. 2009 Feb;48(2):155-65. doi: 10.1097/CHI.0b013e318191769e. PubMed PMID: 19106767
135. Study Record Detail in ClinicalTrials.gov: NCT00150618 –Safety and Efficacy of SDP503 in Treating ADHD in Children and Adolescents Aged 6-17. U.S. National Institutes of Health, 2009. Available at: <https://clinicaltrials.gov/ct2/show/results/NCT00150618?term=NCT00150618&rank=1> (date last access: January 22nd, 2016)
136. Clinical Study Report. Study No: SPD503-304. A phase II study to assess the safety, tolerability and efficacy of SPD503 administered to children and adolescents aged 6-17 with attention deficit hyperactivity disorder (ADHD). Shire Plc, 2 May 2006. Document provided on Mar 1^st^ 2016 by the European Medicines Agency (under the Agency policy on access to documents related to medicinal products for human and veterinary use).
137. Svanborg P, Thernlund G, Gustafsson PA, Hägglöf B, Poole L, Kadesjö B. Efficacy and safety of atomoxetine as add-on to psychoeducation in the treatment of attention deficit/hyperactivity disorder: a randomized, double-blind, placebo-controlled study in stimulant-naïve Swedish children and adolescents. Eur Child Adolesc Psychiatry. 2009 Apr;18(4):240-9. doi: 10.1007/s00787-008-0725-5. Epub 2009 Jan 20. PubMed PMID: 19156355
138. Clinical Study Summary: Summary ID#6671 – Study B4Z-SO-LY15: A Randomized, Double Blind Placebo Controlled Study of the Broader Efficacy of Atomoxetine Hydrochloride in the Treatment of Attention-Deficit/Hyperactivity Disorder in Swedish Children and Adolescents. Eli Lilly and Company, 2007 (pp. 924-951). Available at: <http://www.lillytrials.com/results/Strattera.pdf> (date last access: January 22nd, 2016)
139. Takahashi M, Takita Y, Yamazaki K, Hayashi T, Ichikawa H, Kambayashi Y, Koeda T, Oki J, Saito K, Takeshita K, Allen AJ. A randomized, double-blind, placebo-controlled study of atomoxetine in Japanese children and adolescents with attention-deficit/hyperactivity disorder. J Child Adolesc Psychopharmacol. 2009 Aug;19(4):341-50. doi: 10.1089/cap.2008.0154. PubMed PMID: 19702486
140. Clinical Study Summary: Summary ID#5285 – Study B4Z-JE-LYBC: A Randomized, Double-Blind Placebo-Controlled Efficacy and Safety Comparison of Fixed-Dose Rages of Atomoxetine Hydrochloride in Child Outpatients with Attention-Deficit/Hyperactivity Disorder. Eli Lilly and Company, 2007 (pp. 560-602). Available at: <http://www.lillytrials.com/results/Strattera.pdf> (date last access: January 22nd, 2016)
141. Perez-Alvarez F, Serra-Amaya C, Timoneda-Gallart CA. Cognitive versus behavioral ADHD phenotype: what is it all about? Neuropediatrics. 2009 Feb;40(1):32-8. doi: 10.1055/s-0029-1231055. Epub 2009 Jul 28. PubMed PMID:19639526
142. Thompson MJ, Laver-Bradbury C, Ayres M, Le Poidevin E, Mead S, Dodds C, Psychogiou L, Bitsakou P, Daley D, Weeks A, Brotman LM, Abikoff H, Thompson P, Sonuga-Barke EJ. A small-scale randomized controlled trial of the revised new forest parenting programme for preschoolers with attention deficit hyperactivity disorder. Eur Child Adolesc Psychiatry. 2009 Oct;18(10):605-16. doi: 10.1007/s00787-009-0020-0. Epub 2009 Apr 30. PubMed PMID: 19404717
143. Tramontina S, Zeni CP, Ketzer CR, Pheula GF, Narvaez J, Rohde LA. Aripiprazole in children and adolescents with bipolar disorder comorbid with attention-deficit/hyperactivity disorder: a pilot randomized clinical trial. J Clin Psychiatry. 2009 Apr 21;70(5):756-64. doi: 10.4088/JCP.08m04726. PubMed PMID: 19389329
144. Tucker JD, Suter W, Petibone DM, Thomas RA, Bailey NL, Zhou Y, Zhao Y, Muniz R, Kumar V. Cytogenetic assessment of methylphenidate treatment in pediatric patients treated for attention deficit hyperactivity disorder. Mutat Res. 2009 Jun-Jul;677(1-2):53-8. doi: 10.1016/j.mrgentox.2009.05.005. Epub 2009 May 22. PubMed PMID: 19465145
145. Gevensleben H, Holl B, Albrecht B, Vogel C, Schlamp D, Kratz O, Studer P, Rothenberger A, Moll GH, Heinrich H. Is neurofeedback an efficacious treatment for ADHD? A randomised controlled clinical trial. J Child Psychol Psychiatry. 2009 Jul;50(7):780-9. doi: 10.1111/j.1469-7610.2008.02033.x. Epub 2009 Jan 12. PubMed PMID: 19207632
146. Gevensleben H, Holl B, Albrecht B, Schlamp D, Kratz O, Studer P, Rothenberger A, Moll GH, Heinrich H. Neurofeedback training in children with ADHD: 6-month follow-up of a randomised controlled trial. Eur Child Adolesc Psychiatry. 2010;19(9):715-24
147. Wangler S, Gevensleben H, Albrecht B, Studer P, Rothenberger A, Moll GH, Heinrich H. Neurofeedback in children with ADHD: specific event-related potential findings of a randomized controlled trial. Clin Neurophysiol. 2011 May;122(5):942-50. doi: 10.1016/j.clinph.2010.06.036. Epub 2010 Sep 16. PubMed PMID: 20843737
148. Connor DF, Findling RL, Kollins SH, Sallee F, López FA, Lyne A, Tremblay G. Effects of guanfacine extended release on oppositional symptoms in children aged 6-12 years with attention-deficit hyperactivity disorder and oppositional symptoms: a randomized, double-blind, placebo-controlled trial. CNS Drugs. 2010 Sep;24(9):755-68. doi: 10.2165/11537790-000000000-00000. PubMed PMID: 20806988
149. Study Record Detail in ClinicalTrials.gov: NCT00367835 – SDP503 (Guanfacine Hydrochloride) in ADHD Plus Oppositional Symptoms. U.S. National Institutes of Health, 2010. Available at: <https://clinicaltrials.gov/ct2/show/results/NCT00367835?term=NCT00367835&rank=1&sect=X1056#base> (date last access: January 27th, 2016)
150. Fabiano GA, Vujnovic RK, Pelham WE, Waschbusch DA, Massetti GM, Pariseau ME, et al. Enhancing the effectiveness of special education programming for children with attention deficit hyperactivity disorder using a daily report card. Sch Psychol Rev. 2010;39:219-39
151. Findling RL, Turnbow J, Burnside J, Melmed R, Civil R, Li Y. A randomized, double-blind, multicenter, parallel-group, placebo-controlled, dose-optimization study of the methylphenidate transdermal system for the treatment of ADHD in adolescents. CNS Spectr. 2010 Jul;15(7):419-30. PubMed PMID: 20625364
152. Gustafsson PA, Birberg-Thornberg U, Duchén K, Landgren M, Malmberg K, Pelling H, Strandvik B, Karlsson T. EPA supplementation improves teacher-rated behaviour and oppositional symptoms in children with ADHD. Acta Paediatr. 2010 Oct;99(10):1540-9. doi: 10.1111/j.1651-2227.2010.01871.x. Epub 2010 Jun 8. PubMed PMID: 20491709
153. Martenyi F, Zavadenko NN, Jarkova NB, Yarosh AA, Soldatenkova VO, Bardenstein LM, Kozlova IA, Neznanov NG, Maslova OI, Petrukhin AS, Sukchotina NK, Zykov VP. Atomoxetine in children and adolescents with attention-deficit/hyperactivity disorder: a 6-week, randomized, placebo-controlled, double-blind trial in Russia. Eur Child Adolesc Psychiatry. 2010 Jan;19(1):57-66. doi: 10.1007/s00787-009-0042-7. Epub 2009 Jul 1. PubMed PMID: 19568826
154. Clinical Study Summary: Summary ID#9081 – Study B4Z-MW-LYCZ: Efficacy, Tolerability, and Safety of Once-Daily Atomoxetine Hydrochloride versus Placebo in Russian Children and Adolescents with Attention-Deficit/Hyperactivity Disorder. Eli Lilly and Company, 2006 (pp. 1461-1472). Available at: <http://www.lillytrials.com/results/Strattera.pdf> (date last access: January 27th, 2016)
155. Perreau-Linck E, Lessard N, Lévesque J, Beauregard M. Effects of neurofeedback training on inhibitory capacities in ADHD children: a single-blind, randomized, placebo-controlled study. J Neurotherapy. 2010;14:229-42
156. Salehi B, Imani R, Mohammadi MR, Fallah J, Mohammadi M, Ghanizadeh A, Tasviechi AA, Vossoughi A, Rezazadeh SA, Akhondzadeh S. Ginkgo biloba for attention-deficit/hyperactivity disorder in children and adolescents: a double blind, randomized controlled trial. Prog Neuropsychopharmacol Biol Psychiatry. 2010 Feb 1;34(1):76-80. doi: 10.1016/j.pnpbp.2009.09.026. Epub 2009 Oct 5. PubMed PMID: 19815048
157. Thurstone C, Riggs PD, Salomonsen-Sautel S, Mikulich-Gilbertson SK. Randomized, controlled trial of atomoxetine for attention-deficit/hyperactivity disorder in adolescents with substance use disorder. J Am Acad Child Adolesc Psychiatry. 2010 Jun;49(6):573-82. doi: 10.1016/j.jaac.2010.02.013. PubMed PMID: 20494267
158. Study Record Detail in ClinicalTrials.gov: NCT00399763 – Atomoxetine for Attention Deficit Hyperactivity Disorder in Adolescents with Substance Use Disorder. U.S. National Institutes of Health, 2015. Available at: <https://clinicaltrials.gov/ct2/show/results/NCT00399763?term=NCT00399763&rank=1&sect=X4390156#othr> (date last access: January 29th, 2016)
159. Waxmonsky JG, Waschbusch DA, Pelham WE, Draganac-Cardona L, Rotella B, Ryan L. Effects of atomoxetine with and without behavior therapy on the school and home functioning of children with attention-deficit/hyperactivity disorder. J Clin Psychiatry. 2010 Nov;71(11):1535 51. doi: 10.4088/JCP.09m05496pur. Epub 2010 Jun 29. PubMed PMID: 20673557
160. Zarinara AR, Mohammadi MR, Hazrati N, Tabrizi M, Rezazadeh SA, Rezaie F, Akhondzadeh S. Venlafaxine versus methylphenidate in pediatric outpatients with attention deficit hyperactivity disorder: a randomized, double-blind comparison trial. Hum Psychopharmacol. 2010 Nov;25(7-8):530-5. doi: 10.1002/hup.1148. Epub 2010 Sep 22. PubMed PMID: 20860068
161. Abbasi SH, Heidari S, Mohammadi MR, Tabrizi M, Ghaleiha A, Akhondzadeh S. Acetyl-L-carnitine as an adjunctive therapy in the treatment of attention-deficit/hyperactivity disorder in children and adolescents: a placebo-controlled trial. Child Psychiatry Hum Dev. 2011 Jun;42(3):367-75. doi: 10.1007/s10578-011-0220-y. PubMed PMID: 21336630
162. Arnold LE, Disilvestro RA, Bozzolo D, Bozzolo H, Crowl L, Fernandez S, Ramadan Y, Thompson S, Mo X, Abdel-Rasoul M, Joseph E. Zinc for attention-deficit/hyperactivity disorder: placebo-controlled double-blind pilot trial alone and combined with amphetamine. J Child Adolesc Psychopharmacol. 2011 Feb;21(1):1-19. doi: 10.1089/cap.2010.0073. PubMed PMID: 21309695; PubMed Central PMCID: PMC3037197
163. Bakhshayesh AR, Hänsch S, Wyschkon A, Rezai MJ, Esser G. Neurofeedback in ADHD: a single-blind randomized controlled trial. Eur Child Adolesc Psychiatry. 2011 Sep;20(9):481-91. doi: 10.1007/s00787-011-0208-y. Epub 2011 Aug 13. PubMed PMID: 21842168
164. Dittmann RW, Schacht A, Helsberg K, Schneider-Fresenius C, Lehmann M, Lehmkuhl G, Wehmeier PM. Atomoxetine versus placebo in children and adolescents with attention-deficit/hyperactivity disorder and comorbid oppositional defiant disorder: a double-blind, randomized, multicenter trial in Germany. J Child Adolesc Psychopharmacol. 2011 Apr;21(2):97-110. doi: 10.1089/cap.2009.0111. Epub 2011 Apr 13. PubMed PMID: 21488751
165. Findling RL, Childress AC, Cutler AJ, Gasior M, Hamdani M, Ferreira-Cornwell MC, Squires L. Efficacy and safety of lisdexamfetamine dimesylate in adolescents with attention-deficit/hyperactivity disorder. J Am Acad Child Adolesc Psychiatry. 2011 Apr;50(4):395-405. doi: 10.1016/j.jaac.2011.01.007. Epub 2011 Mar 3. PubMed PMID: 21421179
166. Study Record Detail in ClinicalTrials.gov: NCT00735371 – Efficacy and Safety of Lisdexamfetamine Dimesylate (LDX) in Adolescents With Attention-Deficit/Hyperactivity Disorder (ADHD). U.S. National Institutes of Health, 2011. Available at: <https://clinicaltrials.gov/ct2/show/results/NCT00735371?term=NCT00735371&rank=1> (date last access: February 1st, 2016)
167. Jain R, Segal S, Kollins SH, Khayrallah M. Clonidine extended-release tablets for pediatric patients with attention-deficit/hyperactivity disorder. J Am Acad Child Adolesc Psychiatry. 2011 Feb;50(2):171-9. doi: 10.1016/j.jaac.2010.11.005. PubMed PMID: 21241954
168. Giblin JM, Strobel AL. Effect of lisdexamfetamine dimesylate on sleep in children with ADHD. J Atten Disord. 2011;15(6):491–498
169. Kang KD, Choi JW, Kang SG, Han DH. Sports therapy for attention, cognitions and sociality. Int J Sports Med. 2011 Dec;32(12):953-9. doi: 10.1055/s-0031-1283175. Epub 2011 Nov 8. PubMed PMID: 22068930
170. Kollins SH, Jain R, Brams M, Segal S, Findling RL, Wigal SB, Khayrallah M. Clonidine extended-release tablets as add-on therapy to psychostimulants in children and adolescents with ADHD. Pediatrics. 2011 Jun;127(6):e1406-13. doi: 10.1542/peds.2010-1260. Epub 2011 May 9. PubMed PMID: 21555501
171. Kollins SH, López FA, Vince BD, Turnbow JM, Farrand K, Lyne A, Wigal SB, Roth T. Psychomotor functioning and alertness with guanfacine extended release in subjects with attention-deficit/hyperactivity disorder. J Child Adolesc Psychopharmacol. 2011 Apr;21(2):111-20. doi: 10.1089/cap.2010.0064. Epub 2011 Apr 10. PubMed PMID: 21476931
172. Kratochvil CJ, Vaughan BS, Stoner JA, Daughton JM, Lubberstedt BD, Murray DW, Chrisman AK, Faircloth MA, Itchon-Ramos NB, Kollins SH, Maayan LA, Greenhill LL, Kotler LA, Fried J, March JS. A double-blind, placebo-controlled study of atomoxetine in young children with ADHD. Pediatrics. 2011 Apr;127(4):e862-8. doi: 10.1542/peds.2010-0825. Epub 2011 Mar 21. PubMed PMID: 21422081; PubMed Central PMCID: PMC3387889
173. Study Record Detail in ClinicalTrials.gov: NCT00254462 – Atomoxetine for Treating Attention Deficit Hyperactivity Disorder in Young Children. U.S. National Institutes of Health, 2015. Available at: <https://clinicaltrials.gov/ct2/show/NCT00254462?term=NCT00254462&rank=1> (date last access: February 3rd, 2016)
174. Lansbergen MM, van Dongen-Boomsma M, Buitelaar JK, Slaats-Willemse D. ADHD and EEG-neurofeedback: a double-blind randomized placebo-controlled feasibility study. J Neural Transm. 2011 Feb;118(2):275-84. doi: 10.1007/s00702-010-0524-2. Epub 2010 Dec 17. PubMed PMID: 21165661
175. Pelsser LM, Frankena K, Toorman J, Savelkoul HF, Dubois AE, Pereira RR, Haagen TA, Rommelse NN, Buitelaar JK. Effects of a restricted elimination diet on the behaviour of children with attention-deficit hyperactivity disorder (INCA study): a randomised controlled trial. Lancet. 2011 Feb 5;377(9764):494-503. doi: 10.1016/S0140-6736(10)62227-1. PubMed PMID: 21296237
176. Riggs PD, Winhusen T, Davies RD, Leimberger JD, Mikulich-Gilbertson S, Klein C, Macdonald M, Lohman M, Bailey GL, Haynes L, Jaffee WB, Haminton N, Hodgkins C, Whitmore E, Trello-Rishel K, Tamm L, Acosta MC, Royer-Malvestuto C, Subramaniam G, Fishman M, Holmes BW, Kaye ME, Vargo MA, Woody GE, Nunes EV, Liu D. Randomized controlled trial of osmotic-release methylphenidate with cognitive-behavioral therapy in adolescents with attention-deficit/hyperactivity disorder and substance use disorders. J Am Acad Child Adolesc Psychiatry. 2011 Sep;50(9):903-14. doi: 10.1016/j.jaac.2011.06.010. Epub 2011 Aug 4. PubMed PMID: 21871372
177. Steiner NJ, Sheldrick RC, Gotthelf D, Perrin EC. Computer-based attention training in the schools for children with attention deficit/hyperactivity disorder: a preliminary trial. Clin Pediatr. 2011;50:615–22
178. Wehmeier PM, Schacht A, Wolff C, Otto WR, Dittmann RW, Banaschewski T. Neuropsychological outcomes across the day in children with attention-deficit/hyperactivity disorder treated with atomoxetine: results from a placebo-controlled study using a computer-based continuous performance test combined with an infra-red motion-tracking device. J Child Adolesc Psychopharmacol. 2011 Oct;21(5):433-44. doi: 10.1089/cap.2010.0142. PubMed PMID: 22040189
179. Study Record Detail in ClinicalTrials.gov: NCT00546910 – Comparison of Atomoxetine versus Placebo in Children with Attention-Deficit/Hyperactivity Disorder (ADHD). U.S. National Institutes of Health, 2010. Available at: <https://clinicaltrials.gov/ct2/show/results/NCT00546910?term=NCT00546910&rank=1&sect=X43cb0156#othr> (date last access: February 4th, 2016)
180. Wilens TE, Gault LM, Childress A, Kratochvil CJ, Bensman L, Hall CM, Olson E, Robieson WZ, Garimella TS, Abi-Saab WM, Apostol G, Saltarelli MD. Safety and efficacy of ABT-089 in pediatric attention-deficit/hyperactivity disorder: results from two randomized placebo-controlled clinical trials. J Am Acad Child Adolesc Psychiatry. 2011 Jan;50(1):73-84.e1. doi: 10.1016/j.jaac.2010.10.001. Epub 2010 Nov 25. PubMed PMID: 21156272
181. Yildiz O, Sismanlar SG, Memik NC, Karakaya I, Agaoglu B. Atomoxetine and methylphenidate treatment in children with ADHD: the efficacy, tolerability and effects on executive functions. Child Psychiatry Hum Dev. 2011 Jun;42(3):257-69. doi: 10.1007/s10578-010-0212-3. PubMed PMID: 21165694
182. Zamora J, Velásquez A, Troncoso L, Barra P, Guajardo K, Castillo-Duran C. [Zinc in the therapy of the attention-deficit/hyperactivity disorder in children. A preliminar randomized controlled trial]. Arch Latinoam Nutr. 2011 Sep;61(3):242-6. Spanish. PubMed PMID: 22696891
183. Assareh M, Davari Ashtiani R, Khademi M, Jazayeri S, Rai A, Nikoo M. Efficacy of Polyunsaturated Fatty Acids (PUFA) in the Treatment of Attention Deficit Hyperactivity Disorder: A Randomized, Double-Blind, Placebo-Controlled Clinical Trial. J Atten Disord. 2012 Nov 15. [Epub ahead of print] PubMed PMID: 23160488
184. Duric NS, Assmus J, Gundersen D, Elgen IB. Neurofeedback for the treatment of children and adolescents with ADHD: a randomized and controlled clinical trial using parental reports. BMC Psychiatry. 2012 Aug 10;12:107. doi:10.1186/1471-244X-12-107. PubMed PMID: 22877086
185. Fabiano GA, Pelham WE, Cunningham CE, Yu J, Gangloff B, Buck M, Linke S, Gormley M, Gera S. A waitlist-controlled trial of behavioral parent training for fathers of children with ADHD. J Clin Child Adolesc Psychol. 2012;41(3):337-45. doi: 10.1080/15374416.2012.654464. Epub 2012 Mar 7. PubMed PMID: 22397639
186. Green CT, Long DL, Green D, Iosif AM, Dixon JF, Miller MR, Fassbender C, Schweitzer JB. Will working memory training generalize to improve off-task behavior in children with attention deficit/hyperactivity disorder? Neurotherapeutics. 2012 Jul;9(3):639-48. doi: 10.1007/s13311-012-0124-y. PubMed PMID: 22752960
187. Jafarinia M, Mohammadi MR, Modabbernia A, Ashrafi M, Khajavi D, Tabrizi M, Yadegari N, Akhondzadeh S. Bupropion versus methylphenidate in the treatment of children with attention-deficit/hyperactivity disorder: randomized double-blind study. Hum Psychopharmacol. 2012 Jul;27(4):411-8. doi: 10.1002/hup.2242. PubMed PMID: 22806822
188. Manor I, Magen A, Keidar D, Rosen S, Tasker H, Cohen T, Richter Y, Zaaroor-Regev D, Manor Y, Weizman A. The effect of phosphatidylserine containing Omega3 fatty-acids on attention-deficit hyperactivity disorder symptoms in children: a double-blind placebo-controlled trial, followed by an open-label extension. Eur Psychiatry. 2012 Jul;27(5):335-42. doi: 10.1016/j.eurpsy.2011.05.004. Epub 2011 Jul 31. PubMed PMID: 21807480
189. Manor I, Magen A, Keidar D, Rosen S, Tasker H, Cohen T, Richter Y, Zaaroor-Regev D, Manor Y, Weizman A. Safety of phosphatidylserine containing omega3 fatty acids in ADHD children: a double-blind placebo-controlled trial followed by an open-label extension. Eur Psychiatry. 2013 Aug;28(6):386-91. doi: 10.1016/j.eurpsy.2012.11.001. Epub 2013 Jan 9. PubMed PMID: 23312676
190. Study Record Detail in ClinicalTrials.gov: NCT00418184 – The Efficacy and Safety of Phosphatidylserine-Omega3 in Children with Attention-Deficit/Hyperactivity Disorder. U.S. National Institutes of Health, 2014. Available at: <https://clinicaltrials.gov/ct2/show/results/NCT00418184?term=NCT00418184&rank=1> (date last access: February 8th, 2016)
191. Perera H, Jeewandara KC, Seneviratne S, Guruge C. Combined ω3 and ω6 supplementation in children with attention-deficit hyperactivity disorder (ADHD) refractory to methylphenidate treatment: a double-blind, placebo-controlled study. J Child Neurol. 2012 Jun;27(6):747-53. doi: 10.1177/0883073811435243. PubMed PMID: 22596014
192. Wilens TE, Bukstein O, Brams M, Cutler AJ, Childress A, Rugino T, Lyne A, Grannis K, Youcha S. A controlled trial of extended-release guanfacine and psychostimulants for attention-deficit/hyperactivity disorder. J Am Acad Child Adolesc Psychiatry. 2012 Jan;51(1):74-85.e2. doi: 10.1016/j.jaac.2011.10.012. Epub 2011 Nov 25. PubMed PMID: 22176941
193. Cutler AJ, Brams M, Bukstein O, Mattingly G, McBurnett K, White C, Rubin J. Response/remission with guanfacine extended-release and psychostimulants in children and adolescents with attention-deficit/hyperactivity disorder. J Am Acad Child Adolesc Psychiatry. 2014 Oct;53(10):1092-101. doi:10.1016/j.jaac.2014.08.001. Epub 2014 Aug 15. PubMed PMID: 25245353
194. Study Record Detail in ClinicalTrials.gov: NCT00734578 – Efficacy and Safety of SPD503 in Combination with Psychostimulants. U.S. National Institutes of Health, 2012. Available at: <https://clinicaltrials.gov/ct2/show/results/NCT00734578?term=NCT00734578&rank=1&sect=X4c80156#othr> (date last access: February 8th, 2016)
195. Abikoff H, Gallagher R, Wells KC, Murray DW, Huang L, Lu F, Petkova E. Remediating organizational functioning in children with ADHD: immediate and long term effects from a randomized controlled trial. J Consult Clin Psychol. 2013 Feb;81(1):113-28. doi: 10.1037/a0029648. Epub 2012 Aug 13. PubMed PMID: 22889336
196. Arnold LE, Lofthouse N, Hersch S, Pan X, Hurt E, Bates B, Kassouf K, Moone S, Grantier C. EEG neurofeedback for ADHD: double-blind sham-controlled randomized pilot feasibility trial. J Atten Disord. 2013 Jul;17(5):410-9. doi: 10.1177/1087054712446173. Epub 2012 May 22. PubMed PMID: 22617866
197. Coghill D, Banaschewski T, Lecendreux M, Soutullo C, Johnson M, Zuddas A, Anderson C, Civil R, Higgins N, Lyne A, Squires L. European, randomized, phase 3 study of lisdexamfetamine dimesylate in children and adolescents with attention-deficit/hyperactivity disorder. Eur Neuropsychopharmacol. 2013 Oct;23(10):1208-18. doi: 10.1016/j.euroneuro.2012.11.012. Epub 2013 Jan 15. PubMed PMID: 23332456
198. Soutullo C, Banaschewski T, Lecendreux M, Johnson M, Zuddas A, Anderson C, Civil R, Higgins N, Bloomfield R, Squires LA, Coghill DR. A post hoc comparison of the effects of lisdexamfetamine dimesylate and osmotic-release oral system methylphenidate on symptoms of attention-deficit hyperactivity disorder in children and adolescents. CNS Drugs. 2013 Sep;27(9):743-51. doi:10.1007/s40263-013-0086-6. PubMed PMID: 23801529
199. Coghill DR, Banaschewski T, Lecendreux M, Zuddas A, Dittmann RW, Otero IH, Civil R, Bloomfield R, Squires LA. Efficacy of lisdexamfetamine dimesylate throughout the day in children and adolescents with attention-deficit/hyperactivity disorder: results from a randomized, controlled trial. Eur Child Adolesc Psychiatry. 2014 Feb;23(2):61-8. doi: 10.1007/s00787-013-0421-y. Epub 2013 May 25. PubMed PMID: 23708466; PubMed Central PMCID: PMC3918120
200. Study Record Detail in ClinicalTrials.gov: NCT00763971 – Randomized, Double-blind Safety and Efficacy Study of Lisdexamfetamine Dimesylate (LDX) in Children and Adolescents Aged 6-17. U.S. National Institutes of Health, 2014. Available at: <https://clinicaltrials.gov/ct2/show/results/NCT00763971?term=NCT00763971&rank=1> (date last access: February 9th, 2016)
201. Dittmann RW, Cardo E, Nagy P, Anderson CS, Bloomfield R, Caballero B, Higgins N, Hodgkins P, Lyne A, Civil R, Coghill D. Efficacy and safety of lisdexamfetamine dimesylate and atomoxetine in the treatment of attention-deficit/hyperactivity disorder: a head-to-head, randomized, double-blind, phase IIIb study. CNS Drugs. 2013 Dec;27(12):1081-92. PubMed PMID: 23959815
202. Dittmann RW, Cardo E, Nagy P, Anderson CS, Adeyi B, Caballero B, Hodgkins P, Civil R, Coghill DR. Treatment response and remission in a double-blind, randomized, head-to-head study of lisdexamfetamine dimesylate and atomoxetine in children and adolescents with attention-deficit hyperactivity disorder. CNS Drugs. 2014 Nov;28(11):1059-69. doi: 10.1007/s40263-014-0188-9. PubMed PMID: 25038977
203. Study Record Detail in ClinicalTrials.gov: NCT01106430 – Comparison of Lisdexamfetamine Dimesylate With Atomoxetine HCl in Attention-Deficit/Hyperactivity Disorder (ADHD) Subjects With an Inadequate Response to Methylphenidate. U.S. National Institutes of Health, 2014. Available at: <https://clinicaltrials.gov/ct2/show/NCT01106430?term=NCT01106430&rank=1> (date last access: February 9th, 2016)
204. Nagy P, Häge A, Coghill DR, Caballero B, Adeyi B, Anderson CS, Sikirica V, Cardo E. Functional outcomes from a head-to-head, randomized, double-blind trial of lisdexamfetamine dimesylate and atomoxetine in children and adolescents with attention-deficit/hyperactivity disorder and an inadequate response to methylphenidate. Eur Child Adolesc Psychiatry. 2016 Feb;25(2):141-9. doi: 10.1007/s00787-015-0718-0. Epub 2015 May 22. PubMed PMID: 25999292
205. Hovik KT, Saunes BK, Aarlien AK, Egeland J. RCT of working memory training in ADHD: long-term near-transfer effects. PLoS One. 2013 Dec 9;8(12):e80561. doi: 10.1371/journal.pone.0080561. eCollection 2013. PubMed PMID: 24352414
206. Egeland J, Aarlien AK, Saunes BK. Few effects of far transfer of working memory training in ADHD: a randomized controlled trial. PLoS One. 2013 Oct 4;8(10):e75660. doi: 10.1371/journal.pone.0075660. eCollection 2013. PubMed PMID: 24124503
207. Li L, Yang L, Zhuo CJ, Wang YF. A randomised controlled trial of combined EEG feedback and methylphenidate therapy for the treatment of ADHD. Swiss Med Wkly. 2013 Aug 22;143:w13838. doi: 10.4414/smw.2013.13838. PubMed PMID: 23986461
208. Newcorn JH, Stein MA, Childress AC, Youcha S, White C, Enright G, Rubin J. Randomized, double-blind trial of guanfacine extended release in children with attention-deficit/hyperactivity disorder: morning or evening administration. J Am Acad Child Adolesc Psychiatry. 2013 Sep;52(9):921-30. doi: 10.1016/j.jaac.2013.06.006. Epub 2013 Aug 1. PubMed PMID: 23972694
209. Young J, Rugino T, Dammerman R, Lyne A, Newcorn JH. Efficacy of guanfacine extended release assessed during the morning, afternoon, and evening using a modified Conners' Parent Rating Scale-revised: Short Form. J Child Adolesc Psychopharmacol. 2014 Oct;24(8):435-41. doi: 10.1089/cap.2013.0134. Epub 2014 Oct 6. PubMed PMID: 25286026; PubMed Central PMCID: PMC4203148
210. Stein MA, Sikirica V, Weiss MD, Robertson B, Lyne A, Newcorn JH. Does Guanfacine Extended Release Impact Functional Impairment in Children with Attention-Deficit/Hyperactivity Disorder? Results from a Randomized Controlled Trial. CNS Drugs. 2015 Nov;29(11):953-62. doi: 10.1007/s40263-015-0291-6. PubMed PMID: 26547425
211. Study Record Detail in ClinicalTrials.gov: NCT00997984 – Tolerability and Efficacy of AM and PM Once Daily Dosing With Extended-release Guanfacine Hydrochloride in Children 6-12 With Attention-Deficit/Hyperactivity Disorder (ADHD) (The ADHD Tempo Study). U.S. National Institutes of Health, 2014. Available at: <https://clinicaltrials.gov/ct2/show/NCT00997984?term=NCT00997984&rank=1> (date last access: February 10th, 2016)
212. Ghanizadeh A, Sayyari Z, Mohammadi MR. Effect of methylphenidate and folic Acid on ADHD symptoms and quality of life and aggression: a randomized double blind placebo controlled clinical trial. Iran J Psychiatry. 2013 Aug;8(3):108-12. PubMed PMID: 24454418
213. Oberai P, Gopinadhan S, Varanasi R, et al. Homoeopathic management of attention deficit hyperactivity disorder: A randomised placebo-controlled pilot trial. Indian J Homeopathic Med 2013;7(4):158-167
214. Ogrim G, Hestad KA. Effects of neurofeedback versus stimulant medication in attention-deficit/hyperactivity disorder: a randomized pilot study. J Child Adolesc Psychopharmacol. 2013 Sep;23(7):448-57. doi: 10.1089/cap.2012.0090. Epub 2013 Jun 29. PubMed PMID: 23808786
215. Simonoff E, Taylor E, Baird G, Bernard S, Chadwick O, Liang H, Whitwell S, Riemer K, Sharma K, Sharma SP, Wood N, Kelly J, Golaszewski A, Kennedy J, Rodney L, West N, Walwyn R, Jichi F. Randomized controlled double-blind trial of optimal dose methylphenidate in children and adolescents with severe attention deficit hyperactivity disorder and intellectual disability. J Child Psychol Psychiatry. 2013 May;54(5):527-35. doi: 10.1111/j.1469-7610.2012.02569.x. Epub 2012 Jun 7. PubMed PMID: 22676856
216. Tamm L, Epstein JN, Peugh JL, Nakonezny PA, Hughes CW. Preliminary data suggesting the efficacy of attention training for school-aged children with ADHD. Dev Cogn Neurosci. 2013 Apr;4:16-28. doi: 10.1016/j.dcn.2012.11.004. Epub 2012 Nov 15. PubMed PMID: 23219490
217. van Dongen-Boomsma M, Vollebregt MA, Slaats-Willemse D, Buitelaar JK. A randomized placebo-controlled trial of electroencephalographic (EEG) neurofeedback in children with attention-deficit/hyperactivity disorder. J Clin Psychiatry. 2013 Aug;74(8):821-7. doi: 10.4088/JCP.12m08321. PubMed PMID: 24021501
218. Aman MG, Bukstein OG, Gadow KD, Arnold LE, Molina BS, McNamara NK, Rundberg-Rivera EV, Li X, Kipp H, Schneider J, Butter EM, Baker J, Sprafkin J, Rice RR Jr, Bangalore SS, Farmer CA, Austin AB, Buchan-Page KA, Brown NV, Hurt EA, Grondhuis SN, Findling RL. What does risperidone add to parent training and stimulant for severe aggression in child attention-deficit/hyperactivity disorder? J Am Acad Child Adolesc Psychiatry. 2014 Jan;53(1):47-60.e1. doi:10.1016/j.jaac.2013.09.022. Epub 2013 Nov 18. PubMed PMID: 24342385; PubMedCentral PMCID: PMC3984501
219. Gadow KD, Arnold LE, Molina BS, Findling RL, Bukstein OG, Brown NV, McNamara NK, Rundberg-Rivera EV, Li X, Kipp HL, Schneider J, Farmer CA, Baker JL, Sprafkin J, Rice RR Jr, Bangalore SS, Butter EM, Buchan-Page KA, Hurt EA, Austin AB, Grondhuis SN, Aman MG. Risperidone added to parent training and stimulant medication: effects on attention-deficit/hyperactivity disorder, oppositional defiant disorder, conduct disorder, and peer aggression. J Am Acad Child Adolesc Psychiatry. 2014 Sep;53(9):948-959.e1. doi: 10.1016/j.jaac.2014.05.008. Epub 2014 Jun 12. PubMed PMID: 25151418
220. Farmer CA, Brown NV, Gadow KD, Arnold LE, Kolko DG, Findling RL, Molina BS, Buchan-Page KA, Rice RR Jr, Bangalore SS, Bukstein O, Rundberg-Rivera EV, McNamara N, Aman MG. Comorbid symptomatology moderates response to risperidone, stimulant, and parent training in children with severe aggression, disruptive behavior disorder, and attention-deficit/hyperactivity disorder. J Child Adolesc Psychopharmacol. 2015 Apr;25(3):213-24. doi: 10.1089/cap.2014.0109. PubMed PMID: 25885011
221. Barragán E, Breuer D, Döpfner M. Efficacy and Safety of Omega-3/6 Fatty Acids, Methylphenidate, and a Combined Treatment in Children With ADHD. J Atten Disord. 2014 Jan 24. [Epub ahead of print] PubMed PMID: 24464327
222. Chacko A, Bedard AC, Marks DJ, Feirsen N, Uderman JZ, Chimiklis A, Rajwan E, Cornwell M, Anderson L, Zwilling A, Ramon M. A randomized clinical trial of Cogmed Working Memory Training in school-age children with ADHD: a replication in a diverse sample using a control condition. J Child Psychol Psychiatry. 2014 Mar;55(3):247-55. doi: 10.1111/jcpp.12146. Epub 2013 Oct 7. PubMed PMID: 24117656
223. Ferrin M, Moreno-Granados JM, Salcedo-Marin MD, Ruiz-Veguilla M, Perez-Ayala V, Taylor E. Evaluation of a psychoeducation programme for parents of children and adolescents with ADHD: immediate and long-term effects using a blind randomized controlled trial. Eur Child Adolesc Psychiatry. 2014 Aug;23(8):637-47. doi: 10.1007/s00787-013-0494-7. Epub 2013 Dec 1. PubMed PMID: 24292412
224. Garg J, Arun P, Chavan BS. Comparative short term efficacy and tolerability of methylphenidate and atomoxetine in attention deficit hyperactivity disorder. Indian Pediatr. 2014 Jul;51(7):550-4. PubMed PMID: 25031133
225. Hervas A, Huss M, Johnson M, McNicholas F, van Stralen J, Sreckovic S, Lyne A, Bloomfield R, Sikirica V, Robertson B. Efficacy and safety of extended-release guanfacine hydrochloride in children and adolescents with attention-deficit/hyperactivity disorder: a randomized, controlled, phase III trial. Eur Neuropsychopharmacol. 2014 Dec;24(12):1861-72. doi:10.1016/j.euroneuro.2014.09.014. Epub 2014 Oct 23. PubMed PMID: 25453486
226. Study Record Detail in ClinicalTrials.gov: NCT01244490 – Efficacy and Safety of Extended-release Guanfacine Hydrochloride in Children and Adolescents Aged 6-17 Years With Attention-Deficit/Hyperactivity Disorder (ADHD). U.S. National Institutes of Health, 2015. Available at: <https://clinicaltrials.gov/ct2/show/results/NCT01244490?term=NCT01244490&rank=1>(date last access: February 16th, 2016)
227. Clinical Study Report. Study No: SPD503-316. A phase 3, randomized, double-blind, multicenter, parallel-group, placebo- and active-reference, dose-optimisation efficacy and safety study of extended-release guanfacine hydrochloride in children and adolescents aged 6-17 with attention deficit hyperactivity disorder (ADHD). Shire Plc, 15 October 2013 (version 3.0 Amendment 2 13 January 2015). Document provided on Apr 14^th^ 2016 by the European Medicines Agency (under the Agency policy on access to documents related to medicinal products for human and veterinary use).
228. Hirayama S, Terasawa K, Rabeler R, Hirayama T, Inoue T, Tatsumi Y, Purpura M, Jäger R. The effect of phosphatidylserine administration on memory and symptoms of attention-deficit hyperactivity disorder: a randomised, double-blind, placebo-controlled clinical trial. J Hum Nutr Diet. 2014 Apr;27 Suppl 2:284-91. doi: 10.1111/jhn.12090. Epub 2013 Mar 17. PubMed PMID: 23495677
229. Ko HJ, Kim I, Kim JB, Moon Y, Whang MC, Lee KM, Jung SP. Effects of Korean red ginseng extract on behavior in children with symptoms of inattention and hyperactivity/impulsivity: a double-blind randomized placebo-controlled trial. J Child Adolesc Psychopharmacol. 2014 Nov;24(9):501-8. doi: 10.1089/cap.2014.0013. Epub 2014 Nov 4. PubMed PMID: 25369174
230. Lin DY, Kratochvil CJ, Xu W, Jin L, D'Souza DN, Kielbasa W, Allen AJ. A randomized trial of edivoxetine in pediatric patients with attention-deficit/hyperactivity disorder. J Child Adolesc Psychopharmacol. 2014 May;24(4):190-200. doi: 10.1089/cap.2013.0043. PubMed PMID: 24840045
231. Study Record Detail in ClinicalTrials.gov: NCT00922636 – A Study of Pediatric Patients With Attention-Deficit/Hyperactivity Disorder. U.S. National Institutes of Health, 2014. Available at: <https://clinicaltrials.gov/ct2/show/results/NCT00922636?term=NCT00922636&rank=1>(date last access: February 17th, 2016)
232. Meisel V, Servera M, Garcia-Banda G, Cardo E, Moreno I. Reprint of "Neurofeedback and standard pharmacological intervention in ADHD: a randomized controlled trial with six-month follow-up". Biol Psychol. 2014 Jan;95:116-25. doi: 10.1016/j.biopsycho.2013.09.009. Epub 2013 Sep 18. PubMed PMID: 24055220
233. Pfiffner LJ, Hinshaw SP, Owens E, Zalecki C, Kaiser NM, Villodas M, McBurnett K. A two-site randomized clinical trial of integrated psychosocial treatment for ADHD-inattentive type. J Consult Clin Psychol. 2014 Dec;82(6):1115-27. doi:10.1037/a0036887. Epub 2014 May 26. PubMed PMID: 24865871; PubMed Central PMCID: PMC4244306
234. Steiner NJ, Frenette EC, Rene KM, Brennan RT, Perrin EC. In-school neurofeedback training for ADHD: sustained improvements from a randomized control trial. Pediatrics. 2014 Mar;133(3):483-92. doi: 10.1542/peds.2013-2059. Epub 2014 Feb 17. PubMed PMID: 24534402
235. Steiner NJ, Frenette EC, Rene KM, Brennan RT, Perrin EC. Neurofeedback and cognitive attention training for children with attention-deficit hyperactivity disorder in schools. J Dev Behav Pediatr. 2014 Jan;35(1):18-27. doi: 10.1097/DBP.0000000000000009. PubMed PMID: 24399101
236. van Dongen-Boomsma M, Vollebregt MA, Buitelaar JK, Slaats-Willemse D. Working memory training in young children with ADHD: a randomized placebo controlled trial. J Child Psychol Psychiatry. 2014 Aug;55(8):886-96. doi:10.1111/jcpp.12218. Epub 2014 Mar 15. PubMed PMID: 24628438
237. Widenhorn-Müller K, Schwanda S, Scholz E, Spitzer M, Bode H. Effect of supplementation with long-chain ω-3 polyunsaturated fatty acids on behavior and cognition in children with attention deficit/hyperactivity disorder (ADHD): a randomized placebo-controlled intervention trial. Prostaglandins Leukot Essent Fatty Acids. 2014 Jul-Aug;91(1-2):49-60. doi: 10.1016/j.plefa.2014.04.004. Epub 2014 May 28. PubMed PMID: 24958525
238. Abikoff HB, Thompson M, Laver-Bradbury C, Long N, Forehand RL, Miller Brotman L, Klein RG, Reiss P, Huo L, Sonuga-Barke E. Parent training for preschool ADHD: a randomized controlled trial of specialized and generic programs. J Child Psychol Psychiatry. 2015 Jun;56(6):618-31. doi: 10.1111/jcpp.12346. Epub 2014 Oct 16. PubMed PMID: 25318650
239. Bigorra A, Garolera M, Guijarro S, Hervás A. Long-term far-transfer effects of working memory training in children with ADHD: a randomized controlled trial. Eur Child Adolesc Psychiatry. 2015 Dec 15. [Epub ahead of print] PubMed PMID:26669692
240. Bédard AC, Schulz KP, Krone B, Pedraza J, Duhoux S, Halperin JM, Newcorn JH. Neural mechanisms underlying the therapeutic actions of guanfacine treatment in youth with ADHD: a pilot fMRI study. Psychiatry Res. 2015 Mar 30;231(3):353-6. doi: 10.1016/j.pscychresns.2015.01.012. Epub 2015 Jan 19. PubMed PMID: 25659477
241. Bos DJ, Oranje B, Veerhoek ES, Van Diepen RM, Weusten JM, Demmelmair H, Koletzko B, de Sain-van der Velden MG, Eilander A, Hoeksma M, Durston S. Reduced Symptoms of Inattention after Dietary Omega-3 Fatty Acid Supplementation in Boys with and without Attention Deficit/Hyperactivity Disorder. Neuropsychopharmacology. 2015 Sep;40(10):2298-306. doi: 10.1038/npp.2015.73. Epub 2015 Mar 19. PubMed PMID: 25790022
242. Choi JW, Han DH, Kang KD, Jung HY, Renshaw PF. Aerobic exercise and attention deficit hyperactivity disorder: brain research. Med Sci Sports Exerc. 2015 Jan;47(1):33-9. doi: 10.1249/MSS.0000000000000373. PubMed PMID: 24824770
243. Choi ES, Lee WK. Comparative effects of emotion management training and social skills training in Korean children with ADHD. J Atten Disord. 2015 Feb;19(2):138-46. doi: 10.1177/1087054713496460. Epub 2013 Aug 8. PubMed PMID: 23929521
244. Chou TL, Chia S, Shang CY, Gau SS. Differential therapeutic effects of 12-week treatment of atomoxetine and methylphenidate on drug-naïve children with attention deficit/hyperactivity disorder: A counting Stroop functional MRI study. Eur Neuropsychopharmacol. 2015 Dec;25(12):2300-10. doi: 10.1016/j.euroneuro.2015.08.024. Epub 2015 Sep 8. PubMed PMID: 26409297
245. Corkum P, Elik N, Blotnicky-Gallant PA, McGonnell M, McGrath P. Web-Based Intervention for Teachers of Elementary Students With ADHD: Randomized Controlled Trial. J Atten Disord. 2015 Sep 11. pii: 1087054715603198. [Epub ahead of print] PubMed PMID: 26362259
246. Ghanizadeh A, Haddad B. The effect of dietary education on ADHD, a randomized controlled clinical trial. Ann Gen Psychiatry. 2015 Mar 1;14:12. doi:10.1186/s12991-015-0050-6. eCollection 2015. PubMed PMID: 25767556
247. Hiscock H, Sciberras E, Mensah F, Gerner B, Efron D, Khano S, Oberklaid F. Impact of a behavioural sleep intervention on symptoms and sleep in children with attention deficit hyperactivity disorder, and parental mental health: randomised controlled trial. BMJ. 2015 Jan 20;350:h68. doi: 10.1136/bmj.h68. PubMed PMID: 25646809; PubMed Central PMCID: PMC4299655
248. Matsudaira T, Gow RV, Kelly J, Murphy C, Potts L, Sumich A, Ghebremeskel K, Crawford MA, Taylor E. Biochemical and Psychological Effects of Omega-3/6 Supplements in Male Adolescents with Attention-Deficit/Hyperactivity Disorder: A Randomized, Placebo-Controlled, Clinical Trial. J Child Adolesc Psychopharmacol. 2015 Dec;25(10):775-82. doi: 10.1089/cap.2015.0052. PubMed PMID: 26682998
249. Shakibaei F, Radmanesh M, Salari E, Mahaki B. Ginkgo biloba in the treatment of attention-deficit/hyperactivity disorder in children and adolescents. A randomized, placebo-controlled, trial. Complement Ther Clin Pract. 2015 May;21(2):61-7. doi: 10.1016/j.ctcp.2015.04.001. Epub 2015 Apr 18. PubMed PMID: 25925875
250. Shang CY, Pan YL, Lin HY, Huang LW, Gau SS. An Open-Label, Randomized Trial of Methylphenidate and Atomoxetine Treatment in Children with Attention-Deficit/Hyperactivity Disorder. J Child Adolesc Psychopharmacol. 2015 Sep;25(7):566-73. doi: 10.1089/cap.2015.0035. Epub 2015 Jul 29. PubMed PMID: 26222447
251. Storebø OJ, Skoog M, Rasmussen PD, Winkel P, Gluud C, Pedersen J, Thomsen PH, Simonsen E. Attachment Competences in Children With ADHD During the Social-Skill Training and Attachment (SOSTRA) Randomized Clinical Trial. J Atten Disord. 2015 Oct;19(10):865-71. doi: 10.1177/1087054713520220. Epub 2014 Feb 14. PubMed PMID: 24532801
252. Storebø OJ, Gluud C, Winkel P, Simonsen E. Social-skills and parental training plus standard treatment versus standard treatment for children with ADHD—the randomised SOSTRA trial. PLoS One. 2012;7(6):e37280. doi:10.1371/journal.pone.0037280. Epub 2012 Jun 20. PubMed PMID: 22745657; PubMed Central PMCID: PMC3380035
253. Wilens TE, Robertson B, Sikirica V, Harper L, Young JL, Bloomfield R, Lyne A, Rynkowski G, Cutler AJ. A Randomized, Placebo-Controlled Trial of Guanfacine Extended Release in Adolescents With Attention-Deficit/Hyperactivity Disorder. J Am Acad Child Adolesc Psychiatry. 2015 Nov;54(11):916-25.e2. doi: 10.1016/j.jaac.2015.08.016. Epub 2015 Sep 15. PubMed PMID: 26506582
254. Study Record Detail in ClinicalTrials.gov: NCT01081132 – Dose-optimization in Adolescentes Aged 13-17 Diagnosed With Attention-Deficit/Hyperactivity Disorder (ADHD) Using Extended-release Guanfacine HCl. U.S. National Institutes of Health, 2014. Available at: <https://clinicaltrials.gov/ct2/show/results/NCT01081132?term=NCT01081132&rank=1> (date last access: February 24th, 2016)
255. Clinical Study Report. Study No: SPD503-312. A phase 3, double-blind, randomized, multi-center, placebo-controlled, dose-optimization study evaluating the safety, efficacy, and tolerability of once-daily dosing with extended-release guanfacine hydrochloride in adolescents aged 13-17 diagnosed with attention-deficit/hyperactivity disorder (ADHD). Shire Plc, 3 October 2013. Document provided on Mar 16^th^ 2016 by the European Medicines Agency (under the Agency policy on access to documents related to medicinal products for human and veterinary use).
256. Arabgol F, Panaghi L, Nikzad V. Risperidone Versus Methylphenidate in Treatment of Preschool Children With Attention-Deficit Hyperactivity Disorder. Iran J Pediatr. 2015 Feb;25(1):e265. doi: 10.5812/ijp.265. Epub 2015 Feb 21. PubMed PMID: 26199694
257. Correia Filho AG, Bodanese R, Silva TL, Alvares JP, Aman M, Rohde LA. Comparison of risperidone and methylphenidate for reducing ADHD symptoms in children and adolescents with moderate mental retardation. J Am Acad Child Adolesc Psychiatry. 2005 Aug;44(8):748-55. PubMed PMID: 16034276
258. Ferrin M, Perez-Ayala V, El-Abd S, Lax-Pericall T, Jacobs B, Bilbow A, Taylor E. A Randomized Controlled Trial Evaluating the Efficacy of a Psychoeducation Program for Families of Children and Adolescents With ADHD in the United Kingdom: Results After a 6-Month Follow-Up. J Atten Disord. 2016 Feb 2. pii:1087054715626509. [Epub ahead of print] PubMed PMID: 26838557
259. Janssen TW, Bink M, Geladé K, van Mourik R, Maras A, Oosterlaan J. A randomized controlled trial into the effects of neurofeedback, methylphenidate, and physical activity on EEG power spectra in children with ADHD. J Child Psychol Psychiatry. 2016 Jan 8. doi: 10.1111/jcpp.12517. [Epub ahead of print] PubMed PMID: 26748531
260. Janssen TW, Bink M, Geladé K, van Mourik R, Maras A, Oosterlaan J. A Randomized Controlled Trial Investigating the Effects of Neurofeedback, Methylphenidate, and Physical Activity on Event-Related Potentials in Children with Attention-Deficit/Hyperactivity Disorder. J Child Adolesc Psychopharmacol. 2016 Jan 15. [Epub ahead of print] PubMed PMID: 26771913
261. Steeger CM, Gondoli DM, Gibson BS, Morrissey RA. Combined cognitive and parent training interventions for adolescents with ADHD and their mothers: A randomized controlled trial. Child Neuropsychol. 2016;22(4):394 419. doi: 10.1080/09297049.2014.994485. Epub 2015 Mar 3. PubMed PMID: 25731907; PubMed Central PMCID: PMC4621267
262. Su Y, Yang L, Stein MA, Cao Q, Wang Y. Osmotic Release Oral System Methylphenidate versus Atomoxetine for the Treatment of Attention-Deficit/Hyperactivity Disorder in Chinese Youth: 8-Week Comparative Efficacy and 1-Year Follow-up. J Child Adolesc Psychopharmacol. 2016 Jan 18. [Epub ahead of print] PubMed PMID: 26779845
263. Newcorn JH, Harpin V, Huss M, Lyne A, Sikirica V, Johnson M, Ramos-Quiroga JA, van Stralen J, Dutray B, Sreckovic S, Bloomfield R, Robertson B. Extended-release guanfacine hydrochloride in 6-17-year olds with ADHD: a randomised-withdrawal maintenance of efficacy study. J Child Psychol Psychiatry. 2016 Feb 12. doi: 10.1111/jcpp.12492. [Epub ahead of print] PubMed PMID: 26871297
264. Study Record Detail in ClinicalTrials.gov: NCT01081145 – Maintenance of Efficacy of Extended-Release Guanfacine HCl in Children and Adolescents With Attention-Deficit/Hyperactivity Disorder (ADHD). U.S. National Institutes of Health, 2014. Available at: <https://clinicaltrials.gov/ct2/show/results/NCT01081145?term=NCT01081145&rank=1> (date last access: February 24th, 2016)
