# Supplementary material for: The pharmacological and non-pharmacological treatment of attention deficit hyperactivity disorder in children and adolescents: A systematic review with network meta-analyses of randomised trials
Source: PLoS One. 2017 Jul 12;12(7):e0180355. doi: 10.1371/journal.pone.0180355 (PMC5507500; doi:10.1371/journal.pone.0180355)
Supplement: S1 Table — (DOCX) [file pone.0180355.s006.docx]

**S1 Table. Baseline characteristics of included studies.**

| **Trial name, year**  **Country** | **Study design** | **Sample size** | **Age (years), mean or range** | **Male %** | **Follow-up, evaluation time points (weeks)** | **Mean ADHD duration (years)** | **ADHD severity (e.g. % or ADHD-RS total)** | **Comorbities %**  **Prior stimulant treatment** | **Treatment comparisons** | **Source of data** |
| --- | --- | --- | --- | --- | --- | --- | --- | --- | --- | --- |
| Gittelman-Klein et al., 1976^1^  USA | RCT, parallel, double-blind | 166 | 8.5 | 90.3 | 4 | NR | NR | None –  No (naïve) | Stimulant (MPH-SA); Antipsychotic (THIO); Stimulant+antipsychotic (MPH-SA+THIO); Placebo | Journal article |
| Firestone et al., 1986^2^  Canada | RCT, parallel, triple-blind | 73 | 5-9 | NR | 96 | NR | NR | None –  NR | Stimulant (MPH-SA); BT (parent training); Stimulant+BT (MPH-SA+parent training) | Journal article |
| Casat et al., 1987^3,4^  USA | RCT, parallel, double-blind | 30 | 8.7 | 80.0 | 6 | NR | NR | None –  No (87% treatment naïve) | Antidepressant (BUP); Placebo | Journal article |
| Kupietz et al., 1988^5^  USA | RCT, parallel, double-blind | 58 | 9.7 | NR | 28 | NR | NR | Yes, development reading disorder (100%) –  NR | Stimulant (MPH-SA); Placebo | Journal article |
| Biederman et al., 1989^6,7^  USA | RCT, parallel, double-blind | 73 | 6-17 | 93.5 | 6 | NR | NR | Yes, learning disability (76%), oppositional disorder (48%), conduct disorder (37%) –  Yes (69% previously treatment refractory) | Antidepressant (DESIP); Placebo | Journal article |
| Egger et al., 1992^8^  Germany | RCT, parallel, double-blind | 40 | 9.3 | 90.0 | 24 | 6.8 | 42.5 | NR –  No (15%) | Restricted elimination diet (oligoantigenic diet); Placebo | Journal article |
| Gunning 1992^9^  The Netherlands | RCT, parallel, double-blind | 109 | 8.9 | 95.3 | 7 | NR | NR | Yes, oppositional disorder (30%), Tourette syndrome (19%), conduct disorder (9%) –  No (naïve) | Stimulant (MPH-SA); Non-stimulant α-2 agonist (CLON-SA); Placebo | Unpublished (thesis dissertation) |
| Pisterman et al., 1992^10^  Canada | RCT, parallel, blinding unclear | 57 | 4.1 | 84.2 | 24 | NR | NR | NR –  No (93% medication for ADHD off) | BT (parent training); Waiting list | Journal article |
| Buitelaar et al., 1996^11^  The Netherlands | RCT, crossover, double-blind | 21 | 9.2 | 93.8 | 4 | NR | NR | Yes, depression (15%), anxiety (22%) – No (naïve) | Stimulant (MPH-SA); Placebo | Journal article |
| Conners et al., 1996^12^  USA | RCT, parallel, double-blind | 109 | 8.5 | 90.0 | 6 | ADHD symptoms > 2 years | Moderate-to-severe | NR – No (naïve) | Antidepressant (BUP); Placebo | Journal article |
| Schachar et al., 1997^13^  Canada | RCT, parallel, double-blind | 91 | 8.4 | 81.3 | 24 | ADHD symptoms ≥ 6 months | NR | Yes, oppositional disorder (51%), anxiety (23%), conduct disorders (13%) - NR | Stimulant+BT (MPH-SA+parent training); BT (parent training) + placebo | Journal article |
| Klein and Abikoff, 1997^14^  USA | RCT, crossover, unblinded | 89 | 7.8 | 94.2 | 8 | NR | Severe | None - NR | Stimulant (MPH-SA); BT (parent and teacher training); Stimulant+BT (MPH-SA+parent and teacher training) | Journal article |
| Van der Meere et al., 1999^15^  The Netherlands | RCT, parallel, double-blind | 53 | 9.2 | 86.8 | 7 | NR | NR | Yes, oppositional disorder (26%), conduct disorder (11%), depressive/anxiety disorder (4%)–  No (naïve) | Stimulant (MPH-SA); Non-stimulant α-2 agonist (CLON-SA); Placebo | Journal article |
| MTA Cooperative group, 1999^16-19^  USA | RCT, parallel, unblinded | 579 | 8.5 | 80.0 | 36  56 | NR | CPRS: 0.83 (0.30) | Yes, oppositional disorder (40%), conduct disorder (14%), anxiety disorder (34%), tics (11%)–  No (31% receiving medication prior to study) | Stimulant (MPH-SA); BT (child, parent and teacher training); Stimulant+BT (MPH-SA+child, parent and teacher training); Standard care | Journal article |
| Connor et al., 2000^20^  USA | RCT, parallel, triple-blind | 24 | 9.4 | 100 | 12 | NR | CBCL: 74.0 (4.1) | Yes, oppositional disorder or conduct disorder (100%)–  No (46% history of stimulants, but all subjects were free of medication at baseline) | Stimulant (MPH-SA); Non-stimulant α-2 agonist (CLON-SA); Stimulant (MPH-SA) + non-stimulant α-2 agonist (CLON-SA) | Journal article |
| Pliszka et al., 2000^21^  USA | RCT, parallel, double-blind | 58 | 8.1 | NR | 2 | NR | NR | Yes, oppositional disorder (62%), conduct disorder (10%), anxiety disorder (12%)– No (21% history of stimulants) | Stimulant (MPH-SA); Stimulant (MIX-AMPH); Placebo | Journal article |
| Prince et al., 2000^22^  USA | RCT, parallel, double-blind | 23 | 6-17 | 78.3 | 3 | NR | NR | Yes, oppositional disorder (54%), conduct disorder (11%), anxiety disorder (31%), depression (31%), learning disorders (20%)– Yes (all previous responders to antidepressant during open phase, and 57% had previous ADHD medication) | Antidepressant (NT); Placebo | Journal article |
| Michelson et al., 2001^23-25^  USA | RCT, parallel, double-blind | 297 | 11.2 | 71.4 | 8 | NR | ADHD-RS: 39.4 (9.1)  CGI-S: 4.8 (0.8) | Yes, oppositional disorder (38%), anxiety disorder (0.3%), depression (0.3%) – Yes (70% had previously treated with stimulants) | Non-stimulant (ATX); Placebo | Journal article, unpublished data (sponsor registry and FDA) |
| Scahill et al., 2001^26^  USA | RCT, parallel, double-blind | 34 | 10.4 | 91.2 | 8 | NR | ADHD-RS: 35.8 (8.8) | Yes, tic disorders (100%) –  NR | Non-stimulant α-2 agonist (GUAN-SA); Placebo | Journal article |
| Sonuga-Barke et al., 2001^27^  UK | RCT, parallel,  blinded | 78 | 3.0 | 61.5 | 23 | NR | NR | Yes, conduct disorders (29%) - NR | BT (parent training); Control (parent counseling with support); Waiting list | Journal article |
| Voigt et al., 2001^28^  USA | RCT, parallel, double-blind | 63 | 9.3 | 78.0 | 16 | NR | NR | Yes, oppositional disorder (89%), conduct disorder (15%) – Yes (all patients were being treated successfully with stimulants) | Stimulant (MPH-SA)+PUFA (omega-3 fatty acid); Stimulant (MPH-SA)+Placebo | Journal article |
| Wolraich et al., 2001^29^  USA | RCT, parallel, double-blind | 282 | 9.0 | 82.6 | 4 | NR | NR | Yes, oppositional disorder (42%), conduct disorder (11%), tic disorders (5%), anxiety disorder (1%), depression (1%) – Yes (68% had previously treated with methylphenidate, and 20% naïve) | Stimulant (MPH-SA); Stimulant (MPH-LA); Placebo | Journal article |
| Biederman et al., 2002^31,32^  USA | RCT, parallel, double-blind | 584 | 8.6 | 77.9 | 3 | 2.5-2.9 | CGI-S: 12.2 (6.9) | Yes, comorbid conditions (30%) – Yes, previous prescriptions (62%) | Stimulant (MIX-AMPH-LA); Placebo | Journal article |
| Bor et al., 2002^33^  Australia | RCT, parallel, unclear blinding | 87 | 3.6 | 68.0 | 16 | NR | NR | Yes, all comorbid disruptive behavior – No | BT (parent training); Waiting list | Journal article |
| Greenhill et al., 2002^34^  USA | RCT, parallel, double-blind | 321 | 9.0 | 82.5 | 3 | NR | CGI-S: 4.5 (0.9) | None – Yes, previously treated (64%) | Stimulant (MPH-INT); Placebo | Journal article |
| Lehmkuhl et al., 2002^35-37^  Germany | RCT, parallel, double-blind | 85 | 9.8 | 88.2 | 4 | NR | NR | Yes, oppositional disorder (52%), conduct disorder (9%), unspecified conduct disorder (4%), dysthymia (1%) – NR | Stimulant (MPH-LA); Placebo | Journal article, unpublished data (Cochrane review 2015) |
| Kratochvil et al., 2002^38,39^  USA and Canada | RCT, parallel, open-label | 228 | 10.4 | 73.3 | 10 | NR | ADHD-RS T score: 76.2 (10.3) | Yes, oppositional disorder (55%), depression (9%), elimination disorder (15%) – NR | Stimulant (MPH-SA); Non-stimulant (ATX) | Journal article, unpublished data (sponsor registry) |
| Michelson et al., 2002^40,41^  USA | RCT, parallel, double-blind | 171 | 10.3 | 70.6 | 6 | NR | ADHD-RS T score: 37.2 (9.1) | Yes, oppositional disorder (20%), depression (2%), anxiety (1%), phobia (3%) – Yes, previous stimulant treatment (55%) | Non-stimulant (ATX); Placebo | Journal article, unpublished data (sponsor registry) |
| Spencer et al., 2002a^42,30^  USA | RCT, parallel, double-blind | 147 | 9.8 | 81.1 | 9 | NR | ADHD-RS: 37.7 (8.0) | Yes, oppositional disorder (39%), depression (3%), anxiety (3%), phobia (12%) – No (all stimulant-naïve) | Stimulant (MPH-SA); Non-stimulant (ATX); Placebo | Journal article |
| Spencer et al., 2002b^42,30^  USA | RCT, parallel, double-blind | 144 | 9.8 | 81.1 | 9 | NR | ADHD-RS: 41.3 (8.4) | Yes, oppositional disorder (39%), depression (3%), anxiety (3%), phobia (12%) – Yes (all prior stimulant exposure) | Non-stimulant (ATX); Placebo | Journal article |
| Spencer et al., 2002c^43^  USA | RCT, parallel, double-blind | 41 | 11.0 | 82.9 | 6 | NR | ADHD-RS: 44.0 (6.3) | Yes, all chronic tic disorders (100%), oppositional disorder (52%), conduct disorders (32%), depression (41%), anxiety (51%) – Yes (46% received stimulants) | Antidepressant (DESIP); Placebo | Journal article |
| TSSG, 2002^44^  USA | RCT, parallel, double-blind | 136 | 10.2 | 85.3 | 16 | NR | CTRS: 14.7 (6.5) | Yes, all tic disorders/Tourette’s syndrome (100%), oppositional disorder (38%), conduct disorders (9%), depression (5%), anxiety (9%) – Yes (58% prior stimulants) | Stimulant (MPH-SA); Non-stimulant α-2 agonist (CLON-SA); Stimulant (MPH-SA) + non-stimulant α-2 agonist (CLON-SA); Placebo | Journal article |
| van Oudheusden et al., 2002^45^  The Netherlands | RCT, crossover, double-blind | 26 | 6-13 | 100 | 8 | NR | NR | NR - No | Aminoacids (L-carnitine); Placebo | Journal article |
| Hazell et al., 2003^46^  Australia | RCT, parallel, double-blind | 67 | 9.9 | 91.0 | 6 | NR | NR | Yes, all with oppositional disorder or conduct disorders, anxiety (6%), pervasive disorders (2%) – Yes (100% prior stimulants) | Stimulant (MPH-SA or DEXAM); Stimulant (MPH-SA or DEXAM) + non-stimulant α-2 agonist (CLON-SA) | Journal article |
| Rugino et al., 2003^47^  USA | RCT, parallel, double-blind | 24 | 7.9 | 62.5 | 6 | NR | ADHD-RS T score: 77.7 (8.7) | Yes, oppositional disorder or conduct disorders (25%) - NR | Other unlicensed drug (MODAF); Placebo | Journal article |
| Abikoff et al., 2004^48-50^  USA and Canada | RCT, parallel, single-blind | 103 | 8.2 | 93.0 | 24-96 | NR | NR | Yes, oppositional disorder (53%), conduct disorder (30%), anxiety (17%), depression (4%) – Yes (all with stimulants, and 20% prior stimulant exposure) | Stimulant (MPH-SA); Stimulant+BT (MPH-SA+child and parent training); Stimulant+control (MPH-SA+attential control psychosocial therapy) | Journal article |
| Akhondzadeh et al., 2004^51^  Iran | RCT, parallel, double-blind | 44 | 7.9 | 59.1 | 6 | NR | NR | NR – No (naïve) | Stimulant (MPH-SA); Stimulant+minerals (MPH-SA+Zinc sulfate) | Journal article |
| Bilici et al., 2004^52^  Turkey | RCT, parallel, double-blind | 400 | 9.6 | 82.0 | 12 | 6.5 | ADHD-RS T score: 75.2 (12.0) | NR - NR | Minerals (Zinc sulfate); Placebo | Journal article, unpublished data (trialists’ communication) |
| Döpfner et al., 2004^53^  Germany | RCT, adaptive, blinding unclear | 73 | 8.3 | 93.3 | 16 | NR | NR | Yes, oppositional disorder or conduct disorder (61%), learning problems (5%), tics (3%), dysthymia (3%) - NR | Stimulant+BT (MPH-SA+ MPH-SA+child, parent and teacher training); BT (child, parent and teacher training) | Journal article |
| Kaplan et al., 2004^54^  USA | RCT, parallel, double-blind | 98 | 10.0 | 79.6 | 9 | NR | ADHD-RS: 42.1 (8.1) | Yes, oppositional disorder (100%) - NR | Non-stimulant (ATX); Placebo | Journal article |
| Kelsey et al., 2004^55,56^  USA | RCT, parallel, double-blind | 197 | 9.5 | 70.6 | 8 | NR | ADHD-RS: 42.2 (8.2) | Yes, oppositional disorder (35%), conduct disorder (4%) – Yes (52% prior stimulant exposure) | Non-stimulant (ATX); Placebo | Journal article, unpublished data (sponsor registry) |
| Michelson et al., 2004^57,58^  Europe, Israel, South Africa, and Australia | RCT, parallel, double-blind | 416 | 10.4 | 89.7 | 36 | NR | ADHD-RS: 41.3 (7.8) | Yes, oppositional disorder (43%), depression (2%) – Yes (53% prior stimulant exposure) | Non-stimulant (ATX); Placebo | Journal article, unpublished data (sponsor registry) |
| Wigal et al., 2004^59^  USA | RCT, parallel, double-blind | 132 | 9.8 | 87.8 | 4 | NR | SNAP-ADHD: 43.2 (1.6) | None – No (95% naïve) | Stimulant (MPH-SA); Placebo | Journal article |
| Allen et al., 2005^60,61^  USA | RCT, parallel, double-blind | 148 | 11.2 | 88.5 | 18 | NR | ADHD-RS: 37.0 (9.3) | Yes, all with tic disorders (Tourette syndrome: 79%; chronic motor tics: 30%); oppositional disorders (22%), anxiety (3%), obsessive-compulsive disorder (3%), depression (1%) – Yes (68% prior stimulant exposure) | Non-stimulant (ATX); Placebo | Journal article, unpublished data (sponsor registry) |
| Biederman et al., 2005^62^  USA | RCT, parallel, double-blind | 248 | 10.3 | 71.5 | 9 | NR | ADHD-RS: 36.4 (9.2) | None – Yes (46% previous ADHD treatment) | Other unlicensed drug (MODAF); Placebo | Journal article |
| Jacobs et al., 2005^63^  USA | RCT, parallel, double-blind | 43 | 9.3 | 76.7 | 18 | NR | CPRS: 70.4 (9.0) | None – Yes (21% taking stimulants) | Homeopathy; Placebo | Journal article |
| Kemner et al., 2005^64^  USA | RCT, parallel, open-label | 1323 | 8.9 | 74.0 | 3 | 2.3 | ADHD-RS: 39.9 (8.0) | None – Yes (57% previous ADHD treatment) | Stimulant (MPH-LA); Non-stimulant (ATX) | Journal article |
| Klingberg et al., 2005^65^  Sweden | RCT, parallel, double-blind | 53 | 9.8 | 82.0 | 6 | NR | NR | None – No (naïve) | Cognitive training (WM training); Control | Journal article |
| So, 2005^66,67^  China | RCT,  parallel,  Double-blind | 86 | 8.0 | 89.5 | 24 | NR | NR | Yes, oppositional disorders (50%), anxiety (29%), learning disabilities (19%), conduct disorders (6%), depression (6%) – No (naïve) | Stimulant (MPH-SA); Stimulant+BT (MPH-SA+child and parent training) | Journal article, unpublished data (thesis dissertation) |
| Starr et al., 2005^68^  USA | RCT, parallel, open-label (sub-analysis) | 183 | 8.8 | 82.0 | 3 | 2.2 | ADHD-RS: 40.6 (8.4) | None – Yes (52% previous ADHD treatment) | Stimulant (MPH-LA); Non-stimulant (ATX) | Journal article |
| Weiss et al., 2005^69-71^  USA, Canada and Puerto Rico | RCT, parallel, double-blind | 153 | 9.9 | 80.4 | 7 | NR | ADHD-RS: 37.8 (8.5) | Yes, oppositional disorders (33%), anxiety (3%), learning disability (30%), motor skilss disorder (7%), communications disorder (8%) – Yes (60% prior stimulant exposure) | Non-stimulant (ATX); Placebo | Journal article, unpublished data (sponsor registry) |
| Wigal et al., 2005^72^  USA | RCT, parallel, double-blind | 215 | 8.7 | 67.9 | 3 | NR | CGI-S: 89.8% moderately or severily impaired | NR – NR | Stimulant (MIX-AMPH-LA); Placebo | Journal article |
| Biederman et al., 2006^73^  USA | RCT, parallel, double-blind | 248 | 9.2 | 74.6 | 4 | NR | CPRS: 74.6 (10.4) | None – Yes (31% prior stimulant exposure) | Other unlicensed drug (MODAF); Placebo | Journal article |
| Findling et al., 2006^74^  USA, Canada and Australia | RCT, parallel, double-blind | 318 | 9.5 | 79.2 | 3 | NR | NR | None – Yes (NR) | Stimulant (MPH-SA); Stimulant (MPH-INT); Placebo | Journal article |
| Gau et al., 2006^75^  China | RCT, parallel, open-label | 64 | 10.5 | 90.6 | 4 | NR | CPRS: 77.6 (9.7) | None – Yes (NR) | Stimulant (MPH-SA); Stimulant (MPH-LA) | Journal article |
| Greenhill et al., 2006^76^  USA | RCT, parallel, double-blind | 103 | 10.0 | 64.1 | 7 | 5.0 | CADS-P: 39.5 (8.9) | None – Yes (39% prior stimulant exposure) | Stimulant (MPH-LA); Placebo | Journal article |
| Greenhill et al., 2006b^77^  USA | RCT, parallel, double-blind | 200 | 9.9 | 73.0 | 9 | NR | ADHD-RS: 38.2 (8.9) | None – Yes (53% prior stimulant exposure) | Other unlicensed drug (MODAF); Placebo | Journal article |
| Greenhill et al., 2006c^78-80^  USA | RCT, parallel, double-blind | 114 | 4.8 | 75.0 | 4 | NR | CPRS: 35.5 (8.9) | Yes, oppositional disorders (53%), anxiety (11%), communication disability (22%), conduct disorders (3%) – Yes (all prior stimulant exposure in early phases of the PATS trial) | Stimulant (MPH-LA); Placebo | Journal article |
| Sangal et al., 2006^81^  USA | RCT, crossover, double-blind | 85 | 10.1 | 75.3 | 7 | NR | NR | Yes, oppositional disorders (48%), conduct disorders (4%) – Yes (57% prior stimulant exposure) | Stimulant (MPH-SA); Non-stimulant (ATX) | Journal article |
| Spencer et al., 2006^82^  USA | RCT, parallel, double-blind | 287 | 14.2 | 65.5 | 4 | 5.1 | NR | None – Yes (21% prior stimulant exposure) | Stimulant (MIX-AMPH-LA); Placebo | Journal article |
| Spencer et al., 2006b^83^  USA | RCT, parallel, double-blind | 244 | 10.6 | 69.2 | 4 | 2.5 | NR | Yes, all oppositional disorders with ADHD (subgroup) – NR | Stimulant (MIX-AMPH-LA); Placebo | Journal article |
| Steele et al., 2006^84^  Canada | RCT,  parallel, open-label | 147 | 9.0 | 83.5 | 8 | NR | SNAP: 38.0 (9.6) | Yes, oppositional disorders (41%), anxiety (4%), conduct disorders (1%) – NR | Stimulant (MPH-SA); Stimulant (MPH-LA) | Journal article |
| Trebatická et al., 2006^85,86^  Slovakia | RCT, parallel, double-blind | 61 | 9.5 | 82.0 | 4 | NR | NR | Yes, learning disabilities (30%) – Yes (31% prior ADHD treatment) | Herbal therapy (pine bark extract); Placebo | Journal article |
| Armenteros et al., 2007^87^  USA | RCT,  parallel, double-blind | 25 | 8.1 | 88.0 | 4 | NR | NR | Yes, oppositional disorders (52%), conduct disorders (24%), anxiety (16%) – Yes (all received concomitant psychoestimulants) | Stimulant+antipsychotic (MPH-SA or MIX-AMPH+RISP); Stimulant (MPH-SA or MIX-AMPH) | Journal article |
| Arnold et al., 2007^88^  USA | RCT, parallel, double-blind | 118 | 8.4 | 74.0 | 16 | NR | CGI-S: mean 4.5 (4 = moderate; 5 = marked) | Yes, oppositional disorders (11%), other (18%) – Yes (25% prior ADHD treatment) | Aminoacids (L-carnitine); Placebo | Journal article |
| Bangs et al., 2007^89,90^  USA | RCT, parallel, double-blind | 142 | 14.4 | 73.2 | 9 | NR | ADHD-RS: 34.1 (8.3) | Yes, all co-morbid major depression (100%) – Yes (81% prior stimulant exposure) | Non-stimulant (ATX); Placebo | Journal article, unpublished data (sponsor registry) |
| Biederman et al., 2007^91,92^  USA | RCT, parallel, double-blind | 290 | 9.0 | 69.0 | 4 | 2.0 | ADHD-RS: 43.9 (-)  CGI-S: all patients > moderate or marked ill | NR – Yes (36% prior stimulant exposure) | Stimulant (LDX-LA); Placebo | Journal article |
| Buitelaar et al., 2007^93,58^  Europe, Israel, South Africa, and Australia | RCT, parallel, double-blind, relapse | 163 | 10.8 | 89.6 | 24 | NR | ADHD-RS: 40.9 (7.8) | Yes, oppositional disorders (44%), depression (3%) – Yes (52% prior stimulant exposure) | Non-stimulant (ATX); Placebo | Journal article, unpublished data (sponsor registry) |
| Carlson et al., 2007^94^  USA | RCT, parallel, double-blind | 21 | 9.6 | 83.0 | 6 | NR | NR | Yes, oppositional disorders (50%) – Yes (all prior stimulant exposure) | Stimulant+non-stimulant (MPH-LA+ATX); Non-stimulant (ATX) | Journal article |
| Gau et al., 2007^95,96^  China | RCT, parallel, double-blind | 106 | 9.3 | 88.7 | 6 | NR | ADHD-RS: 36.9 (6.6) | Yes, oppositional disorders (16%), conduct disorders (8%) – Yes (58% prior stimulant exposure) | Non-stimulant (ATX); Placebo | Journal article, unpublished data (sponsor registry) |
| Geller et al., 2007^97,98^  USA | RCT, parallel, double-blind | 176 | 12.0 | 64.8 | 12 | NR | ADHD-RS: 38.7 (8.4) | Yes, all co-morbid anxiety disorder (100%), oppositional disorders (43%), conduct disorders (2%) – Yes (62% prior stimulant exposure) | Non-stimulant (ATX); Placebo | Journal article, unpublished data (sponsor registry) |
| Prasad et al., 2007^99,100^  UK | RCT, parallel, open-label | 201 | 10.9 | 88.6 | 10 | NR | NR | Yes, oppositional disorders (62%), conduct disorders (7%) – NR | Non-stimulant (ATX); Control (standard scare) | Journal article, unpublished data (sponsor registry) |
| van den Hoofdakker et al., 2007^101^  The Netherlands | RCT, parallel, open-label | 96 | 7.4 | 80.9 | 20 | NR | CPRS: 21.4 (6.8) | Yes, oppositional disorders (76%), conduct disorders (16%), anxiety (44%), depression (9%), tic disorders (18%), elimination disorder (22%) – Yes (40% prior ADHD treatment) | BT (parent training); Control (standard care) | Journal article |
| van der Oord et al., 2007^102^  The Netherlands | RCT, parallel, open-label | 50 | 9.9 | 88.0 | 10 | NR | NR | Yes, oppositional disorders/conduct disorders (52%) – No | Stimulant (MPH-SA); Stimulant+BT (MPH-SA+child, parent and teacher training) | Journal article |
| Wang et al., 2007^103,104^  China, Korea, Mexico | RCT, parallel, double-blind | 330 | 9.7 | 81.8 | 8 | NR | ADHD-RS: 38.0 (7.6) | Yes, oppositional disorders (24%) – Yes (24% prior stimulant exposure) | Stimulant (MPH-SA); Non-stimulant (ATX) | Journal article, unpublished data (sponsor registry) |
| Amiri et al., 2008^105^  Iran | RCT, parallel, double-blind | 60 | 9.1 | 78.3 | 6 | NR | NR | NR – NR | Stimulant (MPH-SA); Other unlicensed drug (MODAF) | Journal article |
| Bangs et al., 2008^106,107^  Europe and Australia | RCT, parallel, double-blind | 226 | 9.6 | 93.4 | 8 | NR | NR | Yes, all co-morbid oppositional disorders (100%), conduct disorders (12%), anxiety (2%) – Yes (69% prior stimulant exposure) | Non-stimulant (ATX); Placebo | Journal article, unpublished data (sponsor registry) |
| Bierdeman et al., 2008^108-110^  USA | RCT, parallel, double-blind | 345 | 10.5 | 74.5 | 8 | 2.6 | NR | NR – NR | Non-stimulant α-2 agonist (GUAN-LA); Placebo | Journal article, unpublished data (ClinicalTrials.gov and study report provided by EMA, Feb 2016) |
| Palumbo et al., 2008^111-113^  USA | RCT, parallel, double-blind | 122 | 9.5 | 80.4 | 16 | NR | NR | Yes, oppositional disorders (47%), conduct disorders (9%) – Yes (47% prior stimulant exposure) | Stimulant (MPH-SA); Non-stimulant α-2 agonist (CLON-SA); Stimulant (MPH-SA) + non-stimulant α-2 agonist (CLON-SA); Placebo | Journal article |
| Findling et al., 2008^114^  USA | RCT, parallel, double-blind | 282 | 8.8 | 66.3 | 7 | 1.6 | ADHD-RS: 42.0 (-) | Not reported – No (86% naïve) | Stimulant (MPH-LA); Stimulant (MPH-TS); Placebo | Journal article |
| Heriot et al., 2008^115^  New Zealand | RCT, parallel, double-blind | 16 | 4.8 | 81.3 | 12 | >1 | NR | Yes, oppositional disorders (31%) – NR | Stimulant (MPH-SA); BT (parent training); Stimulant+BT (MPH-SA+parent training); Placebo | Journal article |
| Konofal et al., 2008^116^  France | RCT, parallel, double-blind | 23 | 6.1 | 78.3 | 12 | NR | ADHD-RS: 36.6 (7.3) | Yes, all iron-deficient non-anemia (100%), restless legs syndrome (48%) – NR | Minerals (Iron); Placebo | Journal article |
| Newcorn et al., 2008^117,118^  USA | RCT, parallel, double-blind | 516 | 10.3 | 74.2 | 6 | NR | ADHD-RS: 40.5 (8.8) | Yes, oppositional disorders (37%) – Yes (60% prior stimulant exposure) | Stimulant (MPH-LA); Non-stimulant (ATX); Placebo | Journal article, unpublished data (sponsor registry) |
| Torrioli et al., 2008^119^  France, Italy and Spain | RCT, parallel, double-blind | 63 | 9.2 | 100 | 48 | NR | CPRS: 69.6 (-) | Yes, all comorbid X fragile syndrome (100%), seizure disorders/epilepsy (5%) – NR | Aminoacids (L-carnitine); Placebo | Journal article |
| Vaisman et al., 2008^120^  Israel | RCT, parallel, double-blind | 83 | 9.3 | 54.2 | 12 | NR | NR | NR – No | PUFA (omega-3 fatty acid); Control (fish oil); Placebo | Journal article |
| Weber et al., 2008^121^  USA | RCT, parallel, double-blind | 54 | 9.8 | 63.0 | 8 | 6.6 | ADHD-RS: 26.8 (-) | Yes, oppositional disorders (44%) – NR | Herbal therapy (St John’s Wort); Placebo | Journal article |
| Arabgol et al., 2009^122^  Iran | RCT, parallel, double-blind | 33 | 8.9 | 75.8 | 6 | NR | ADHD-RS: 27.6 (6.0) | None – NR | Stimulant (MPH-SA); Antidepressant (REBOX) | Journal article |
| Block et al., 2009^123,124^  USA | RCT, parallel, double-blind | 288 | 8.9 | 72.6 | 6 | NR | ADHD-RS: 30.7 (9.9) | Yes, oppositional disorders (32%) – Yes (32% prior stimulant exposure) | Non-stimulant (ATX); Placebo | Journal article, unpublished data (sponsor registry) |
| Childress et al., 2009^125^  USA | RCT, parallel, double-blind | 253 | 8.7 | 64.4 | 5 | 3.9 | NR | Yes, respiratory, thoracic and mediastinal disorders (MPH 27%,), immune system disorders (23%), nervous system disorders (19%), surgical and medical  procedures (15%), infections and infestations  (14%) and skin and subcutaneous tissue disorders (MPH 11%) – Yes (31% prior stimulant exposure) | Stimulant (MPH-LA); Placebo | Journal article |
| Dell'Agnello et al., 2009^126^  Italy | RCT, parallel, double-blind | 139 | 9.9 | 92.9 | 8 | NR | CPRS: 12.0 (2.4) | Yes, all comorbid oppositional disorders (100%), anxiety (11%), depression/dysthymia (8%), specific phobias (8%), obsessive-compulsive disorder (2%) – Yes (18% prior ADHD therapy) | Non-stimulant (ATX); Placebo | Journal article |
| Johnson et al., 2009^127^  Sweden | RCT, crossover, double-blind | 75 | 12.0 | 85.3 | 12 | NR | NR | Yes, reading/writing disorders (43%), oppositional disorders (24%), Austisme-like condition or Asperger (15%), learning disability (11%), depression or anxiety (8%), specific phobias (8%), obsessive-compulsive disorder (2%) – NR | PUFA (omega-3/6 fatty acid); Placebo | Journal article |
| Kahbazi et al., 2009^128^  Iran | RCT, parallel, double-blind | 46 | 9.1 | 76.1 | 6 | NR | NR | NR – NR | Other unlicensed drug (MODAF); Placebo | Journal article |
| Montoya et al., 2009^129,130^  Spain | RCT, parallel, double-blind | 151 | 10.3 | 79.5 | 12 | NR | ADHD-RS: 39.2 (9.0) | Yes, oppositional disorders (26%), anxiety (13%), tic disorders (17%), affective disorders (3%) – No (naïve) | Non-stimulant (ATX); Placebo | Journal article, unpublished data (ClinicalTrials.gov) |
| Nair et al., 2009^131^  India | RCT, parallel, double-blind | 50 | 7.1 | 80.0 | 4 | NR | NR | Yes, oppositional disorders (10%), conduct disorders (15%), seizures (13%) – NR | Non-stimulant α-2 agonist (CLON-SA); Other unlicensed drug (CARBA) | Journal article |
| Pelsser et al., 2009^132^  The Netherlands | RCT, parallel, open-label | 27 | 6.2 | 81.5 | 9 | NR | NR | Yes, oppositional disorders (82%) – NR | Restricted elimination diet (elimination diet); Waiting list | Journal article |
| Raz et al., 2009^133^  Israel | RCT, parallel, double-blind | 78 | 10.5 | 60.3 | 7 | NR | NR | Yes, learning disability (35%), sleep disorders (8%), dyspraxia (8%), oppositional disorders (5%), anxiety (5%), tic disorders (3%), obsessive-compulsive disorder (3%), conduct disorders (2%) – Yes (16% prior stimulant exposure) | PUFA (omega-3/6 fatty acid); Placebo | Journal article |
| Sallee et al., 2009^134-136^  USA | RCT, parallel, double-blind | 324 | 10.5 | 71.9 | 9 | NR | ADHD-RS: 40.1 (8.7) | Yes, oppositional disorders (6%) – NR | Non-stimulant α-2 agonist (GUAN-LA); Placebo | Journal article, unpublished data (ClinicalTrials.gov and study report provided by EMA, Mar 2016) |
| Svanborg et al., 2009^137,138^  Sweden | RCT, parallel, double-blind | 99 | 11.5 | 80.8 | 10 | NR | ADHD-RS: 39.5 (6.7) | Yes, oppositional disorders (20%), tic disorders (14%), depression (5%) – No (naïve) | Non-stimulant+BT (ATX+child and parent training); BT (child and parent training) + placebo | Journal article, unpublished data (sponsor registry) |
| Takahashi et al., 2009^139,140^  Japan | RCT, parallel, double-blind | 245 | 10.5 | 85.3 | 8 | NR | ADHD-RS: 32.2 (8.7) | Yes, oppositional disorders (14%), conduct disorders (1%) – Yes (54% prior stimulant exposure) | Non-stimulant (ATX); Placebo | Journal article, unpublished data (sponsor registry) |
| Perez-Alvarez et al., 2009^141^  Spain | RCT, parallel, open-label | 150 | 9.0 | 80.0 | 48 | NR | SNAP-IV ≥ 2.5: 64% | None – No (naïve) | Stimulant (MPH-LA); HPT (humanistic psychotherapy); Stimulant+HPT (MPH-LA+humanistic psychotherapy) | Journal article |
| Thompson et al., 2009^142^  UK | RCT, parallel, double-blind | 41 | 4.2 | 73.0 | 17 | NR | NR | NR – NR | BT (parent training); Control (standard care) | Journal article |
| Tramontina et al., 2009^143^  Brazil | RCT, parallel, double-blind | 43 | 11.9 | 46.5 | 6 | NR | SNAP-IV: 2.1 (0.5) | Yes, all comorbid bipolar disorder (100%), disruptive behavioral disorders (81%), anxiety (49%), psychosis (37%) – NR | Antipsychotic (ARIP); Placebo | Journal article |
| Tucker et al., 2009^144^  USA | RCT, parallel, open-label | 109 | 8.4 | 63.4 | 12 | NR | NR | NR – No (naïve) | Stimulant+BT (MPH-LA+child and parent training); BT (child and parent training) | Journal article |
| Gevensleben et al., 2009^145-147^  Germany | RCT, parallel, open-label | 102 | 9.6 | 81.9 | 8, 24 | NR | NR | Yes, dyslexia (23%), conduct disorders (18%), emotional disorders (6%), tic disorders (3%) – No (93% naïve) | Neurofeedback (theta-beta and slow cortical potential training); Cognitive training (attention training) | Journal article |
| Connor et al., 2010^148,149^  USA | RCT, parallel, double-blind | 217 | 9.4 | 68.2 | 9 | NR | ADHD-RS: 42.3 (7.9) | Yes, all comorbid oppositional disorders (100%) – NR | Non-stimulant α-2 agonist (GUAN-LA); Placebo | Journal article, unpublished data (ClinicalTrials.gov) |
| Fabiano et al., 2010^150^  USA | RCT, parallel, open-label | 63 | 8.2 | 86.0 | 28 | NR | NR | Yes, all comorbid learning disabilities (in special education 100%), oppositional disorders or conduct disorders (88%) | BT (child, parent and teacher training); Control (standard of care) | Journal article |
| Findling et al., 2010^151^  USA | RCT, parallel, double-blind | 217 | 14.6 | 74.7 | 7 | NR | ADHD-RS: 36.5 (7.3) | None – Yes (44% prior stimulant exposure) | Stimulant (MPH-TS); Placebo | Journal article |
| Gustafsson et al., 2010^152^  Sweden | RCT, parallel, double-blind | 109 | 7-12 | NR | 15 | NR | CPRS: 48.5 (16.0) | Yes, oppositional disorders (61%), neuromotor problems (48%) – NR | PUFA (omega-3 fatty acid); Placebo | Journal article, unpublished data (trialists’ communication) |
| Martenyi et al., 2010^153,154^  Russia | RCT, parallel, double-blind | 105 | 9.8 | 85.7 | 6 | NR | ADHD-RS: 37.6 (7.4) | Yes, conduct disorders (5%), oppositional disorders (2%) – No (40% prior treatment, but no stimulants) | Non-stimulant (ATX); Placebo | Journal article, unpublished data (sponsor registry) |
| Perreau-Linck et al., 2010^155^  Canada | RCT, parallel, single-blind | 9 | 10.3 | 88.9 | 9 | NR | NR | None – Yes (78% prior stimulant exposure) | Neurofeedback (theta-beta training); Placebo | Journal article |
| Salehi et al., 2010^156^  Iran | RCT, parallel, double-blind | 50 | 9.4 | 78.0 | 6 | NR | NR | NR – NR | Stimulant (MPH-SA); Herbal therapy (*Ginkgo biloba*) | Journal article |
| Thurstone et al., 2010^157,158^  USA | RCT, parallel, double-blind | 70 | 16.1 | 78.6 | 12 | NR | ADHD-RS: 42.2 (9.4) | Yes, all comorbid substance use disorders (100%), conduct disorders (53%), depression (29%) – NR | Non-stimulant+BT (ATX+child and parent training); BT (child and parent training) + placebo | Journal article, unpublished data (ClinicalTrials.gov) |
| Waxmonsky et al., 2010^159^  USA | RCT, parallel, open-label | 56 | 8.6 | 80.4 | 8 | NR | CGI-S: 4.3 (-) | Yes, oppositional disorders (43%), conduct disorders (39%) – Yes (63% prior stimulant exposure) | Non-stimulant+BT (ATX+child, parent and teacher training); Non-stimulant (ATX) | Journal article |
| Zarinara et al., 2010^160^  Iran | RCT, parallel, double-blind | 38 | 9.5 | 71.1 | 6 | NR | NR | NR – NR | Stimulant (MPH-SA); Antidepressant (VENLAF) | Journal article |
| Abbasi et al., 2011^161^  Iran | RCT, parallel, double-blind | 40 | 8.6 | 70.0 | 6 | NR | NR | NR – NR | Stimulant (MPH-SA); Stimulant + aminoacids (MPH-SA+L-carnitine) | Journal article |
| Arnold et al., 2011^162^  USA | RCT, parallel, double-blind | 52 | 9.6 | 82.7 | 13 | NR | NR | Yes, oppositional disorders or conduct disorders (65%), learning disorders (19%), depression (17%) – Yes (23% prior stimulant exposure) | Minerals (Zinc supplementation); Placebo | Journal article |
| Bakhshayesh et al., 2011^163^  Germany | RCT, parallel, single-blind | 38 | 9.3 | 74.3 | 15 | NR | NR | Yes, motor skills disorder (14%), enuresis (6%), emotional disorder (6%), conduct disorder (3%) – Yes (20% concomitant stimulants) | Neurofeedback (theta-beta training); Control | Journal article |
| Dittmann et al., 2011^164^  Germany | RCT, parallel, double-blind | 180 | 11.0 | 84.4 | 9 | NR | SNAP-IV: 37.0 (9.5) | Yes, oppositional disorders, conduct disorders (24%) –  Yes (44% prior stimulant exposure) | Non-stimulant (ATX); Placebo | Journal article |
| Findling et al., 2011^165,166^  USA | RCT, parallel, double-blind | 314 | 14.6 | 70.3 | 4 | NR | ADHD-RS: 37.8 (6.9) | None – NR | Stimulant (LDX-LA); Placebo | Journal article, unpublished data (ClinicalTrials.gov) |
| Jain et al., 2011^167^  USA | RCT, parallel, double-blind | 236 | 9.5 | 72.4 | 8 | NR | ADHD-RS: 44.5 (-) | None – NR | Non-stimulant α-2 agonist (CLON-LA); Placebo | Journal article |
| Giblin et al., 2011^168^  USA | RCT, parallel, double-blind | 24 | 9.7 | 41.7 | 10 | NR | ADHD-RS: 47.7 (5.5) | NR – Yes (83% prior stimulant exposure) | Stimulant (LDX-LA); Placebo | Journal article |
| Kang et al., 2011^169^  Korea | RCT, parallel, double-blind | 32 | 8.5 | 100 | 6 | NR | NR | NR – NR | Stimulant+BT (MPH-SA+child training); Stimulant+Exercise (MPH-SA+physical activity/sports) | Journal article |
| Kollins et al., 2011^170^  USA | RCT, parallel, double-blind | 198 | 10.4 | 73.6 | 8 | NR | ADHD-RS: 39.0 (7.3) | None – Yes (all prior stimulant exposure) | Stimulant+non-stimulant α-2 agonist (MPH-LA or LDX-LA+CLON-LA); Stimulant (MPH-LA or LDX) | Journal article |
| Kollins et al., 2011^171^  USA | RCT, parallel, double-blind | 178 | 12.6 | 69.7 | 6 | 4.6 | NR | NR – Yes (72% prior stimulant exposure) | Non-stimulant α-2 agonist (GUAN-LA); Placebo | Journal article |
| Kratochvil et al., 2011^172,173^  USA | RCT, parallel, double-blind | 101 | 6.1 | 67.7 | 8 | NR | ADHD-RS: 38.3 (1.0) | Yes, oppositional disorders (35%), enuresis (17%) – NR | Non-stimulant+BT (ATX+parent training); BT (parent training) | Journal article, unpublished data (ClinicalTrials.gov) |
| Lansbergen et al., 2011^174^  The Netherlands | RCT, parallel, double-blind | 14 | 10.2 | 92.9 | 16 | NR | Moderatelly ill (79%), marked ill (21%) | NR – NR | Neurofeedback (IFBT); Placebo | Journal article |
| Pelsser et al., 2011^175^  Belgium and The Netherlands | RCT, parallel phase (cross-over trial), open-label | 100 | 6.9 | 86.0 | 5 | NR | ADHD-RS: 46.4 (4.4) | Yes, oppositional disorders (47%), conduct disorders (8%) – NR | Restricted elimination diet (elimination diet); Control (healthy diet) | Journal article |
| Riggs et al., 2011^176^  USA | RCT, parallel, double-blind | 303 | 16.5 | 78.9 | 16 | NR | ADHD-RS: 38.7 (8.9) | Yes, all comorbid substance use disorders (100%), conduct disorders (32%), depression (13%) – NR | Simulant+BT (MPH-LA+child training); BT (child training) + placebo | Journal article |
| Steiner et al., 2011^177^  USA | RCT, parallel, blinding unclear | 41 | 12.4 | 52.2 | 16 | NR | NR | NR – NR | Neurofeedback (theta-beta training); Control; Waiting list | Journal article |
| Wehmeier et al., 2011^178,179^  Germany | RCT, parallel, double-blind | 125 | 9.0 | 77.6 | 8 | NR | ADHD-RS: 36.9 (11.6) | Yes, oppositional disorders (31%), conduct disorders (17%), tics (1%) – Yes (25% prior stimulant exposure) | Non-stimulant (ATX); Placebo | Journal article, unpublished data (ClinicalTrials.gov) |
| Wilens et al., 2011^180^  USA | RCT, parallel, doube-blind | 97 | 8.6 | 65.7 | 8 | NR | ADHD-RS: 42.8 (1.1) | None– Yes (NR) | Non-stimulant (ATX); Placebo | Journal article |
| Yildiz et al., 2011^181^  Turkey | RCT, parallel, open-label | 29 | 9.9 | 88.0 | 12 | NR | ADHD-RS: 45.7 (12.1) | Yes, oppositional disorders (28%), conduct disorders (24%) – NR | Stimulant (MPH-LA); Non-stimulant (ATX) | Journal article |
| Zamora et al., 2011^182^  Chile | RCT, parallel, double-blind | 40 | 9.6 | 72.5 | 6 | NR | NR | NR – NR | Stimulant (MPH-SA); Stimulant+minerals (MPH-SA+Zinc sulfate) | Journal article |
| Assareh et al., 2012^183^  Iran | RCT, parallel, double-blind | 40 | 9.1 | 75.0 | 10 | NR | ADHD-RS: 35.5 (6.0) | Yes, oppositional disorders (53%) – NR | Stimulant+PUFA (MPH-SA+omega-3/6 fatty acid); Stimulant (MPH-SA)+Placebo | Journal article |
| Duric et al., 2012^184^  Norway | RCT, parallel, blinding unclear | 130 | 11.2 | 79.1 | 10 | NR | ADHD-RS: 34.1 (8.9) | NR – NR | Stimulant (MPH-SA); Neurofeedback (theta-beta training); Stimulant+neurofeedback (MPH-SA+theta-beta training) | Journal article |
| Fabiano et al., 2012^185^  USA | RCT, parallel, blinding unclear | 55 | 8.5 | 87.3 | 8 | NR | NR | Yes, oppositional disorders (70%), conduct disorders (6%) – NR | BT (parent training); Waiting list | Journal article |
| Green et al., 2012^186^  USA | RCT, parallel, double-blind | 30 | 9.7 | 65.4 | 4 | NR | CPRS: 75.3 (7.9) | Yes, learning disability (4%) – NR | Cognitive training (WM training); Placebo | Journal article |
| Jafarinia et al., 2012^187^  Iran | RCT, parallel, double-blind | 44 | 9.6 | 67.5 | 6 | NR | ADHD-RS: 32.8 (11.2) | None – No (naïve) | Stimulant (MPH-SA); Antidepressant (BUP) | Journal article |
| Manor et al., 2012^188-189^  Israel | RCT, parallel, double-blind | 200 | 9.2 | 66.5 | 15 | NR | CPRS: 68.7 (15.3) | Yes, oppositional disorders (9%), anxiety (3%), tic disorders (3%) – Yes (17% prior ADHD treatment) | PUFA (omega-3 fatty acid); Placebo | Journal article, unpublished data (ClinicalTrials.gov) |
| Perera et al., 2012^190^  Sri Lanka | RCT, parallel, double-blind | 98 | 9.3 | 73.4 | 24 | NR | NR | Yes, concomitant physical illness (19%) – Yes (all) | Stimulant+PUFA (MPH-SA+omega-3/6 fatty acid); Stimulant (MPH-SA)+Placebo | Journal article |
| Wilens et al., 2012^191-194^  USA | RCT, parallel, double-blind | 461 | 10.8 | 71.6 | 9 | NR | ADHD-RS: 37.4 (7.8) | None – Yes (all with stimulants) | Stimulant+non-stimulant α-2 agonist (MPH-LA or LDX-LA+GUAN-LA); Stimulant (MPH-LA or LDX) | Journal article, unpublished data (ClinicalTrials.gov) |
| Abikoff et al., 2013^195^  USA | RCT, parallel, single-blind | 158 | 9.1 | 64.6 | 12 | NR | CPRS: 75.0 (9.2) | Yes, enuresis (5%), tic disorders (4%), learning/reading disability (3%) – NR | BT (child, parent and teacher training); Waiting list | Journal article |
| Arnold et al., 2013^196^  USA | RCT, parallel, double-blind | 39 | 8.9 | 79.5 | 8 | NR | Moderatelly ill (74%), marked ill (10%) | NR – Yes (56% prior ADHD treatment) | Neurofeedback (theta-beta training); Placebo | Journal article |
| Coghill et al., 2013^197-200^  Europe (Belgium, France, Germany, Hungary, Italy, The Netherlands, Poland, Spain, Sweden, UK) | RCT, parallel, double-blind | 336 | 10.9 | 81.0 | 7 | 2.3 | NR | Yes, any comorbid psychiatric diagnosis (20%), oppositional disorders (8%) – Yes (55% prior ADHD treatment) | Stimulant (LDX-LA); Stimulant (MPH-LA); Placebo | Journal article, unpublished data (ClinicalTrials.gov) |
| Dittmann et al., 2013^201-2034^  North America (Canada, USA) and Europe (Belgium, Germany, Hungary, Italy, Poland, Spain, Sweden) | RCT, parallel, double-blind | 267 | 10.7 | 75.2 | 9 | 2.5 | ADHD-RS: 42.3 (6.4) | Yes, any comorbid psychiatric diagnosis (19%), oppositional disorders (10%) – Yes (all prior ADHD treatment and non-responders to MPH) | Stimulant (LDX-LA); Non-stimulant (ATX) | Journal article, unpublished data (ClinicalTrials.gov) |
| Hovik et al., 2013^205,206^  Norway | RCT, parallel, open-label | 75 | 10.4 | 73.1 | 16 | NR | ADHD-RS: 31.6 (10.3) | None -Yes (NR) | Cognitive training (WM training); Control | Journal article |
| Li et al., 2013^207^  China | RCT, parallel, double-blind | 64 | 10.6 | 84.4 | 24 | NR | ADHD-RS: 41.7 (8.7) | None – Yes (all) | Stimulant+neurofeedback (MPH-SA+theta-beta training); Stimulant (MPH-SA) | Journal article |
| Newcorn et al., 2013^208-211^  Canada and USA | RCT, parallel, double-blind | 340 | 9.1 | 70.6 | 8 | 1.7 | NR | None – NR | Non-stimulant α-2 agonist (GUAN-LA); Placebo | Journal article, unpublished data (ClinicalTrials.gov) |
| Ghanizadeh et al., 2013^212^  Iran | RCT, parallel, double-blind | 49 | 9.6 | 85.7 | 8 | NR | NR | None – NR | Stimulant+vitamins (MPH-SA+vitamin B9/folic acid); Stimulant (MPH-SA) | Journal article |
| Oberai et al., 2013^213^  India | RCT, parallel, single-blind | 61 | 9.3 | 79.6 | 48 | NR | Moderatelly ill (69%), marked ill (6%) | None – NR | Homeopathy; Placebo | Journal article |
| Ogrim et al., 2013^214^  Norway | RCT, parallel, open-label | 32 | 10.9 | 62.1 | 34 | NR | NR | Yes, learning disability (59%), oppositional disorders or conduct disorders (45%), depression or anxiety (21%) – No (naïve) | Stimulant (MPH-LA or DEXAM); Neurofeedback (theta-beta training) | Journal article |
| Simonoff et al., 2013^215^  UK | RCT, parallel, double-blind | 122 | 11.2 | 69.8 | 16 | NR | CPRS: 27.6 (5.9) | Yes, all comorbid intellectual disability (100%) – NR | Stimulant (MPH-SA); Placebo | Journal article |
| Tamm et al., 2013^216^  USA | RCT, parallel, open-label | 105 | 9.3 | 67.6 | 12 | NR | NR | Yes, elimination disorders (8%), anxiety (5%), oppositional disorders (3%), learning disability (1%), depression (1%) – NR | Cognitive training (attention training); Waiting list | Journal article |
| van Dongen-Boomsma et al., 2013^217^  The Netherlands | RCT, parallel, double-blind | 41 | 10.6 | 82.9 | 10 | NR | ADHD-RS: 31.3 (8.6) | Yes, oppositional disorders (15%), anxiety (12%), dislexia (5%) – Yes (61% prior ADHD treatment) | Neurofeedback (theta-beta training); Placebo | Journal article |
| Aman et al., 2014^218-220^  USA | RCT, parallel, double-blind | 168 | 8.9 | 76.8 | 9 | NR | NR | Yes, all severe physical aggression (100%), oppositional disorders (74%), conduct disorders (26%) – Yes (NR) | Stimulant+antipsychotic+BT (MPH-LA or MIX-AMPH+RISP+parent training); Stimulant+BT (MPH-LA or MIX-AMPH+parent training) | Journal article |
| Barragán et al., 2014^221^  Mexico | RCT, parallel, open-label | 90 | 8.3 | 66.7 | 48 | NR | ADHD-RS: 41.4 (4.4) | None – No (naïve) | Stimulant (MPH-SA)+PUFA (omega-3/6 fatty acid); Stimulant (MPH-SA); PUFA (omega-3/6 fatty acid) | Journal article |
| Chacko et al., 2014^222^  USA | RCT, parallel, double-blind | 85 | 8.4 | 77.6 | 3 | NR | NR | Yes, oppositional disorders (45%), conduct disorder (12%) – NR | Cognitive training (WM training); Placebo | Journal article |
| Ferrin et al., 2014^223^  Spain | RCT, parallel, double-blind | 81 | 10.6 | 80.2 | 48 | NR | NR | Yes, oppostional disorders or conduct disorders (30%), depression or anxiety (2%), autistic spectrum disorder (2%), other (19%) – NR | BT (parent training); Control (parent counseling with support) | Journal article |
| Garg et al., 2014^224^  India | RCT, parallel, open-label | 84 | 8.6 | 81.2 | 8 | NR | ADHD-RS: 43.6 (14.4) | Yes, oppositional disorders (54%), conduct disorders (10%) – NR | Stimulant (MPH-SA); Non-stimulant (ATX) | Journal article |
| Hervas et al., 2014^225-227^  North America (Canada, USA) and Europe (Austria, France, Germany, Ireland, Italy, Poland, Romania, Spain, Sweden, Ukraine, UK) | RCT, parallel, double-blind | 338 | 10.8 | 73.7 | 10-13 | 2.2 | ADHD-RS: 43.3 (5.7) | Yes, oppositional disorders (12%) – NR | Non-stimulant (ATX); Non-stimulant α-2 agonist (GUAN-LA); Placebo | Journal article, unpublished data (ClinicalTrials.gov and study report provided by EMA, Apr 2016) |
| Hirayama et al., 2014^228^  Japan | RCT, parallel, double-blind | 40 | 8.9 | 94.4 | 8 | NR | NR | NR – No (naïve) | Aminoacids (phosphatidyl-serine); Placebo | Journal article |
| Ko et al., 2014^229^  Korea | RCT, parallel, double-blind | 72 | 10.9 | 62.9 | 8 | NR | NR | None – NR | Herbal therapy (*Ginseng*); Placebo | Journal article |
| Lin et al., 2014^230,231^  North America (Canada, USA), Mexico, Puerto Rico, Taiwan | RCT, parallel, double-blind | 340 | 11.6 | 70.6 | 8 | 4.7 | ADHD-RS: 38.0 (-) | Yes, conduct disorders (19%), oppositional disorders (2%), anxiety (2%) – Yes (42% prior stimulant exposure) | Stimulant (MPH-LA); Non-stimulant (EDIVOX); Placebo | Journal article, unpublished data (ClinicalTrials.gov) |
| Meisel et al., 2014^232^  Spain | RCT, parallel, unblinded | 27 | 9.2 | 52.2 | 8 | NR | ADHD-RS: 31.6 (7.4) | None – NR | Stimulant (MPH-SA); Neurofeedback (theta-beta training) | Journal article |
| Pfiffner et al., 2014^233^  USA | RCT, parallel, unblinded | 199 | 8.6 | 58.4 | 12 | NR | NR | NR – NR | BT (child, parent and teacher training); BT (parent training); Control (usual care) | Journal article |
| Steiner et al., 2014^234,235^  USA | RCT, parallel, single-blind | 104 | 8.6 | 67.3 | 24 | NR | NR | NR – NR | Cognitive training (attention training); Neurofeedback (theta-beta training); Control | Journal article |
| van Dongen-Boomsma et al., 2014^236^  The Netherlands | RCT, parallel, double (triple)-blind | 51 | 6.6 | 72.3 | 5 | NR | ADHD-RS: 34.1 (5.1) | Yes, with comorbidities (19%), oppositional disorders (6%) – No (naïve) | Cognitive training (WM training); Placebo | Journal article |
| Widenhorn-Müller et al., 2014^237^  Germany | RCT, parallel, double-blind | 110 | 8.9 | 77.9 | 16 | NR | NR | NR – No (97% naïve) | PUFA (omega-3 fatty acid); Placebo | Journal article |
| Abikoff et al., 2015^238^  USA | RCT, parallel, single-blind | 164 | 4.0 | 43.8 | 8 | NR | NR | NR – NR | BT (parent training); Waiting list | Journal article |
| Bigorra et al., 2015^239^  Spain | RCT, parallel, double-blind | 66 | 8.9 | 45.5 | 7 | NR | NR | Yes, oppositional disorders (27%), elimination disorders (5%) – No (naïve) | Cognitive training (WM training); Placebo | Journal article |
| Bédard et al., 2015^240^  USA | RCT, parallel, double-blind | 25 | 11.1 | 72.0 | 8 | NR | CGI-S: mean 5.2 (0.9); (4 = moderate; 5 = marked) | Yes, oppositional disorders (44%) – Yes (20% prior stimulant exposure) | Non-stimulant α-2 agonist (GUAN-LA); Placebo | Journal article |
| Bos et al., 2015^241^  The Netherlands | RCT, parallel, double-blind | 40 | 10.3 | 100.0 | 16 | NR | NR | NR – Yes (95% prior stimulant exposure) | PUFA (omega-3 fatty acid); Placebo | Journal article |
| Choi et al., 2015^242^  Korea | RCT, parallel, blinding unclear | 35 | 15.9 | 100.0 | 6 | NR | NR | NR – NR | Stimulant+BT (MPH-SA+child training); Stimulant+Exercise (MPH-SA+physical activity/sports) | Journal article |
| Choi et al., 2015^243^  Korea | RCT, parallel, blinding unclear | 80 | 11.2 | 44.4 | 16 | NR | NR | NR – NR | BT (child training); Waiting list | Journal article |
| Chou et al., 2015^244^  Taiwan | RCT, parallel, open-label | 50 | 10.5 | 81.0 | 12 | NR | NR | Yes, oppositional disorders (33%) – No (naïve) | Stimulant (MPH-LA); Non-stimulant (ATX) | Journal article |
| Corkum et al., 2015^245^  Canada | RCT, parallel, single-blind | 58 | 8.8 | 87.9 | 12 | NR | NR | None – NR | BT (teacher training); Waiting list | Journal article |
| Ghanizadeh et al., 2015^246^  Iran | RCT, parallel, blinding unclear | 106 | 8.4 | NR | 4 | NR | NR | NR – Yes (94% prior stimulant exposure) | Stimulant+restricted elimination diet (MPH-SA+elimination diet); Stimulant (MPH-SA) | Journal article |
| Hiscock et al., 2015^247^  Australia | RCT, parallel, single-blind | 244 | 10.1 | 85.2 | 24 | NR | ADHD-RS: 36.4 (9.7) | Yes, all comorbid moderate to severe sleep disorders (100%, rated by parents), learning disability (34%), autism spectrum or Asperger disorder (25%) – Yes (NR) | BT (parent training); Control (usual care) | Journal article, unpublished data (trialists’ communication) |
| Matsudaira et al., 2015^248^  UK | RCT, parallel, double-blind | 76 | 13.7 | 100.0 | 12 | NR | NR | None – Yes (NR) | PUFA (omega-3/6 fatty acid); Placebo | Journal article |
| Shakibaei et al., 2015^249^  Iran | RCT, parallel, double-blind | 66 | 8.1 | 65.0 | 6 | NR | ADHD-RS: 37.1 (7.5) | None – NR | Stimulant+herbal therapy (MPH-SA+*Ginkgo biloba*); Stimulant (MPH-SA) | Journal article |
| Shang et al., 2015^250^  Taiwan | RCT, parallel, open-label | 160 | 9.8 | 87.5 | 24 | NR | CGI-S: mean 5.8 (0.6); (4 = moderate; 5 = marked) | None – No (naïve) | Stimulant (MPH-LA); Non-stimulant (ATX) | Journal article |
| Storebø et al., 2015^251,252^  Denmark | RCT, parallel, single-blind | 56 | 10.4 | 70.9 | 24 | NR | NR | Yes, oppositional disorders (15%), anxiety (11%), enuresis (7%), depression (4%) – No (naïve) | BT (child and parent training); Control (usual care) | Journal article |
| Wilens et al., 2015^253-255^  USA | RCT, parallel, double-blind | 314 | 14.5 | 64.7 | 13 | 5.1 | NR | Yes, oppositional disorders – Yes (74% prior stimulant exposure) | Non-stimulant α-2 agonist (GUAN-LA); Placebo | Journal article, unpublished data (ClinicalTrials.gov and study report provided by EMA, Mar 2016) |
| Arabgol et al., 2015^256^  Iran | RCT, parallel, double-blind | 38 | 4.4 | 69.7 | 6 | NR | NR | NR – NR | Antipsychotic (RISP); Stimulant (MPH-SA) | Journal article |
| Correia-Filho et al., 2005^257^  Brazil | RCT, parallel, single-blind | 46 | 11.8 | 75.6 | 4 | NR | NR | Yes, all mental retardation – No | Antipsychotic (RISP); Stimulant (MPH-SA) | Journal article |
| Ferrin et al., 2016^258^  UK | RCT, parallel, open-label | 69 | 10.7 | 81.1 | 24 | 1.2 | NR | Yes, oppostional disorders or conduct disorders (91%), dyslexia (32%), anxiety (18%), obsessive compulsive disorders (15%), self-harm (11%), conduct disorders (9%)– NR | BT (parent training); Control (usual care) | Journal article |
| Janssen et al., 2016^259,260^  The Netherlands | RCT, parallel, double-blind | 112 | 9.6 | 75.3 | 10 | NR | NR | None – NR | Stimulant (MPH-SA); Neurofeedback (theta-beta training); Exercise (physical activity/sports) | Journal article |
| Steeger et al., 2016^261^  USA | RCT, parallel, double-blind | 104 | 12.5 | 69.0 | 5 | NR | NR | Yes, oppositional disorders (40%), conduct disorders (2%), depression (2%), anxiety (2%), tic disorders (3%), Tourette’s disorder (2%) – NR | BT (parent training); Cognitive training (WM training); BT+cognitive training (parent training+WM training); Placebo | Journal article |
| Su et al., 2016^262^  China | RCT, parallel, open-label | 262 | 9.5 | 83.1 | 8 | NR | ADHD-RS: 32.5 (8.8) | Yes, oppositional disorders (30%) – No (97% naïve) | Stimulant (MPH-LA); Non-stimulant (ATX) | Journal article |
| Newcorn et al., 2016^263,264^  North America (Canada, USA) and Europe (Belgium, France, Germany, Italy, The Netherlands, Spain, Sweden, UK) | RCT, parallel, double-blind | 316 | 10.8 | 74.3 | 26 | 2.6 | ADHD-RS: 43.5 (6.3) | Yes, oppositional disorders (27%) – Yes (71% prior psychoactive drugs) | Non-stimulant α-2 agonist (GUAN-LA); Placebo | Journal article, unpublished data (ClinicalTrials.gov) |

ARIP: aripiprazole (antipsychotic); BT: behavioral therapy; BUP: bupropion; CARBA: carbamazepine (anticonvulsant/antiepileptic); CLON-SA: clonidine short acting; CLON-LA: clonidine long acting; DESIP: desipramina (tricyclic antidepressant); DEXAM: dexamphetamine; EDIVOX: edivoxetine; DISC: Diagnostic Interview Schedule for Children; GUAN-SA: guanfacine short acting; GUAN-LA: guanfacine long acting; IFBT: individualized frequency band training; LDX-LA: lisdexamphetamine long acting; MIX-AMPH: mixed amphetamine salts; MIX-AMPH-LA: mixed amphetamine salts long acting; MODAF: modafinil; MPH-LA: methylphenidate long acting; MPH-SA: methylphenidate short acting; MPH-INT: methylphenidate intermediate release/methylphenidate modified-release; MPH-TS: methylphenidate transdermal system (patch); MTA: Multimodal Treatment study of children with ADHD; NR: Not reported; NT: nortriptyline (tricyclic antidepressant); PUFA: polyunsaturated fatty acid; REBOX: reboxetine; RISP: risperidone; THIO: Thioridazine (antipsychotic); TSSG: Tourette’s Syndrome Study Group; WM: working memory; VENLAF: venlafaxine (antidepressant SNRI)
