# Supplementary material for: The pharmacological and non-pharmacological treatment of attention deficit hyperactivity disorder in children and adolescents: A systematic review with network meta-analyses of randomised trials
Source: PLoS One. 2017 Jul 12;12(7):e0180355. doi: 10.1371/journal.pone.0180355 (PMC5507500; doi:10.1371/journal.pone.0180355)
Supplement: S2 Table — (DOCX) [file pone.0180355.s007.docx]

**S2 Table. Definitions of study participants, interventions and outcomes**

| **Trial name, year** | **Participants** | **Interventions** | **Outcomes** |
| --- | --- | --- | --- |
| Gittelman-Klein et al., 1976^1^ | **Eligibility criteria**  Age between 6-12 years, attending school, free of neurological disease (such as epilepsy, hemiparesis, cerebral palsy, microcephaly), nonpsychotic, had to obtain a Weschsler Intelligence Scale for children intelligence quotient of at least 80, one sub-IQ of 85 or more; have a parent or responsible adult willing to come for weekly visits; have a telephone at home; and come from a family fluent in English  **Diagnostic criteria**  DSM-II  **Number randomized**  166  **Age**  6-12 years (range)  **Co-morbidities/Co-medications**  None/No  **ADHD subtypes**  Not reported | **Arm 1**  MPH-SA high dose: methylphenidate 1.8 mg/kg/day (50 mg/day)  **Arm 2**  THI: thioridazine 6 mg/kg/day (193 mg/day)  **Arm 3**  MPH-SA high dose+THIO: combination of both methylphenidate 1.8 mg/kg/day (57 mg/day) and thioridazine 7 mg/kg/day (128 mg/day)  **Arm 4**  Placebo: placebo pill | **Treatment response** (as a dichotomous outcome)  Global improvement based on Global Improvement Scale, 8-point rating scale scale (from completely well to much worse). Ratings of “improved”, “much improved” and “completely well” were combined in the category of “responders” (rater: psychiatrist/psychologist, parents and teachers)  **All-cause treatment discontinuation** (as a dichotomous outcome)  Yes, reported as study discontinuations  **Tolerability** (as a dichotomous outcome)  Yes, reported as study discontinuations due to AEs  **Serious adverse events** (as dichotomous outcome)  None reported  **Specific adverse events** (as dichotomous outcome)  Loss of appetite/anorexia (as an adverse event)  Sleep disturbances (as an dverse event)  Any cardiovascular event (as adverse events) |
| Firestone et al., 1986^2^ | **Eligibility criteria**  Age between 5-9 years, referred to the learning, psychiatry or psychology outpatient services of a children hospital; ratings of 1.5 or higher on the Teachers Hyperactivity Index; Peabody Picture Vocabulary IQ of 85 or higher; free of definite signs of brain damage, epilepsy, or psychosis.  **Diagnostic criteria**  DSM-III  **Number randomized**  73  **Age**  5-9 years (range)  **Co-morbidities/Co-medications**  None/No  **ADHD subtypes**  Not reported | **Arm 1**  MPH-SA low/medium dose: methylphenidate 10-30 mg/day (mean: 22 mg/day)  **Arm 2**  BT (parent training): parent training plus placebo pill. Parent training was carried out by senior doctoral-level interns in clinical psychology (supervised by registered psychologists). Parents of children were provided with initial consultations averaging 3 sessions. During this time, they were asked to read a book on child management and demonstrate a reasonable understanding of behavioral principles. Having completed this, parents were asked to join a parents’ group in which more specific child reading beahavior management programs were discussed for 6 sessions. Finally, the parents were taugh how to cooperate efficiently with school personnel  **Arm 3**  MPH-SA+BT: combination of both methylphenidate (10-30 mg/day) and parent training (as described before) | **Treatment response** (as a dichotomous outcome)  Not reported/data not abstractable  **All-cause treatment discontinuation** (as a dichotomous outcome)  Yes, reported as study discontinuations  **Tolerability** (as a dichotomous outcome)  Not reported  **Serious adverse events** (as dichotomous outcome)  Not reported  **Specific adverse events** (as dichotomous outcome)  Not reported |
| Casat et al., 1987^3,4^ | **Eligibility criteria**  Age between 6-12 years, children were either treatment naïve or unresponsive to conventional stimulant therapy; concurrence of parent and teacher scores on the Conners parent and teacher’s questionnaires (> 1.5); less than 20 kg, to be in good physical health, and to have normal hematological and clinical lab values as well as normal electroencephalogram (EEG) and electrocardiogram (EKG). All children were free of all medications for a minimum of 14 days prior to study entry. Girls were premenarchal. Exclusions: IQ < 70 on the WISC-R, history of seizure, tic disorder, any unstable medical condition, and known hypersensitivity to psychomedications.  **Diagnostic criteria**  DSM-III  **Number randomized**  30  **Age**  6-12 years (range)  **Co-morbidities/Co-medications**  Yes, conduct disorders (3%)/No  **ADHD subtypes**  Not reported | **Arm 1**  BUP: bupropion 50-75 mg/day  **Arm 2**  Placebo: placebo pill | **Treatment response** (as a dichotomous outcome)  Not reported/data not abstractable  **All-cause treatment discontinuation** (as a dichotomous outcome)  Yes, reported as study discontinuations  **Tolerability** (as a dichotomous outcome)  Yes, reported as study discontinuations due to AEs  **Serious adverse events** (as dichotomous outcome)  Not reported  **Specific adverse events** (as dichotomous outcome)  Not reported |
| Kupietz et al., 1988^5^ | **Eligibility criteria**  Children had to be: 7-13 years, IQ ≥ 80 and dual diagnosis of ADHD and developmental reading disorder. Exclusion: an additional Axis I psychiatric diagnosis or uncorrected hearing or visual deficits.  **Diagnostic criteria**  DSM-III  **Number randomized**  58  **Age**  7-13 years (range)  **Co-morbidities/Co-medications**  Yes, all with developmental reading disorder/Not reported  **ADHD subtypes**  Not reported | **Arm 1**  MPH-SA low/medium dose: one of three methyphenidate dosages (0.6, 1.0 or 1.4 mg/kg/day)  **Arm 2**  Placebo: placebo pill | **Treatment response** (as a dichotomous outcome)  Not reported/data not abstractable  **All-cause treatment discontinuation** (as a dichotomous outcome)  Yes, reported as study discontinuations  **Tolerability** (as a dichotomous outcome)  Not reported  **Serious adverse events** (as dichotomous outcome)  Not reported  **Specific adverse events** (as dichotomous outcome)  Not reported |
| Biederman et al., 1989^6,7^ | **Eligibility criteria**  No patient needed to be excluded by having mental retardation (full scale IQ < 70), autism, psychosis, ir another medical or neurological disorder or by abnormal results of psychiatric and medical evaluations, including routine laboratory tests and initial ECG.  **Diagnostic criteria**  DSM-III  **Number randomized**  73  **Age**  6-17 years (range)  **Co-morbidities/Co-medications**  Yes, learning disability (76%), oppositional disorder (48%) and conduct disorder (37%)/No  **ADHD subtypes**  Not reported | **Arm 1**  DESIP: Desipramine 2-5 mg/kg/day  **Arm 2**  Placebo: placebo pill | **Treatment response** (as a dichotomous outcome)  Physician-rated Clinical Global Impression (CGI-S) Scale which includes scales rating of Global Severity (1=not ill, to 7=extremely ill) and Global Improvement (CGI-I) wich includes scales rating of improvement (1=very much improved, to 7=very much worse – e.g. responder considered very much [1] or much improved [2]). Response was also evaluated using the Conners Abbreviated Parent and Teacher Questionnaires (10 items, maximum score=30) completed by parent and teachers.  **All-cause treatment discontinuation** (as a dichotomous outcome)  Yes, reported as study discontinuations  **Tolerability** (as a dichotomous outcome)  Yes, reported as study discontinuations  **Serious adverse events** (as dichotomous outcome)  None reported  **Specific adverse events** (as dichotomous outcome)  Loss of appetite/anorexia (as an adverse event)  Sleep disturbances (as an adverse event)  Decreased weight (adverse event as defined by the authors e.g. sustained weight loss > 5% of initial weight) |
| Egger et al., 1992^8^ | **Eligibility criteria**  Children had a more than 1-year history of short attention span, distractibility, impulsivity, and poorly organized overactivity. In addition, they had to have a score of more than 15 on the short form of the Conners’ Rating Scale  **Diagnostic criteria**  DSM-III-R and ICD-9  **Number randomized**  40  **Age**  3-15 years (range)  **Co-morbidities/Co-medications**  Not reported/No  **ADHD subtypes**  Not reported | **Arm 1**  Dietary restriction diet (oligoantigenic diet): three doses of enzyme desensitisation (including beta-glucuronidase and small quantities of food antigens) intradermally at two monthly intervals  **Arm 2**  Placebo: placebo solution | **Treatment response** (as a dichotomous outcome)  Not reported/data not abstractable  **All-cause treatment discontinuation** (as a dichotomous outcome)  Yes, reported as study discontinuatios  **Tolerability** (as a dichotomous outcome)  Not reported  **Serious adverse events** (as dichotomous outcome)  Not reported  **Specific adverse events** (as dichotomous outcome)  Not reported |
| Gunning 1992^9^ | **Eligibility criteria**  Boys and girls aged 6-15 years, IQ > 70, living in a family home, and attending school; ADHD symptoms impeding development, and psychological/educational treatments insufficient effect; no earlier use of stimulant drugs or clonidine, and no psychoactive medications of any kind in the last 6 months; no medical contraindications; no important changes expected for the course of the trial  **Diagnostic criteria**  DSM-III-R  **Number randomized**  109  **Age**  6-15 years (range)  **Co-morbidities/Co-medications**  Yes, oppositional disorder (30%), Tourette syndrome (19%), conduct disorder (9%)/No  **ADHD subtypes**  Not reported | **Arm 1**  MPH-SA low/medium dose: methylphenidate 0.6 mg/kg/day  **Arm 2**  CLON-SA: clonidine 4 µg/kg/day  **Arm 3**  Placebo: placebo pill | **Treatment response** (as a dichotomous outcome)  Global improvement based on rating of the extent change in clinical condition of the subject compared to baseline. For the ratings informant had to make a choice out of the following categories: “not clinically significant”, “clinically significant improvement”, or “clinically significant deterioration” (rating evaluations reported by 2 child psychiatrists)  **All-cause treatment discontinuation** (as a dichotomous outcome)  Yes, reported as study withdrawals  **Tolerability** (as a dichotomous outcome)  Not reported  **Serious adverse events** (as dichotomous outcome)  Not reported  **Specific adverse events** (as dichotomous outcome)  Decreased apetite (adverse event)  Sleep disturbances (adverse event) |
| Pisterman 1992^10^ | **Eligibility criteria**  Children scored at least one rating of 1.5 SD above the age and gender mean on the Conners Hyperactivity Index; standard score was ≥ 80 on the Peabody Picture Vocabulary Test if children spoke only English or ≥ if the clildren was bilingual (English/French); 3-6 years and not yet attending first grade; medication status was contestant (if any).  **Diagnostic criteria**  DSM-III  **Number randomized**  57  **Age**  3-6 years (range)  **Co-morbidities/Co-medications**  Not reported/No  **ADHD subtypes**  Not reported | **Arm 1**  BT (parent training): Parent training was carried out by a therapist with a master’s degree and took place in a room equipped with one-way mirror, age-appropiate toys, and living room furniture. Session 1 involved a comprehensive diagnostic interview. Session 2, the parents completed questionnaires and consent forms. Sesion 3 stareted BT consisted of 20 minutes each of free play and compliance task ant 10 minutes each of parent-supervised and unsupervised attention tasks. Target parent was instructed to read a magazine and let the child play with toys in the room (free-play period) which was followed by the compliance task (in which the parent was instructed to issue 15 predetermined simple commandas). This was followed by a short break. In this task, the parent the parents were instructed to provide assistance as desired to their children in performing a paper-and-pencil task. After that task, and for the next 10 minutes, the child engaged in a different attention task with the therapist (not parent supervised). The BT concluded with a second 10-min period of free play.  All behavioral instruction was provided through modeling, role-playing, and 2 individual in-clinic instruction sessions in which the parent and child were videotaped. Parents reviewed the tapes and received individual feedback  **Arm 2**  Waiting list (WL): control-group families were seen in the clinic at comparable points in time. They were given the same standardize tasks to perform, followed by a brief interview about their children’s behavior. No videotape feedback was received | **Treatment response** (as a dichotomous outcome)  Clinical improvement on percentage compliance minimum of 50% increase (ratings evaluations reported by parents)  **All-cause treatment discontinuation** (as a dichotomous outcome)  Yes, reported as study withdrawals  **Tolerability** (as a dichotomous outcome)  Not reported  **Serious adverse events** (as dichotomous outcome)  Not reported  **Specific adverse events** (as dichotomous outcome)  Not reported |
| Buitelaar et al., 1996^11^ | **Eligibility criteria**  Children had scores in the clinical range on both CBCL and CTRS hyperactivity factors, deficits in attention performance task in the neuropschological testing; no previous treatment with psychotropic medication; and a clinical indication for drug treatment. Children were excluded if a diagnosis of tic disorder or pervasive developmental disorder, a family history of tic disorder, and the usual contra-indications for treatment with β-blocker (e.g. cardiac diseases, hypotension, obstructive pulmonary diseases, and insulin-dependent diabetes)  **Diagnostic criteria**  DSM-III-R  **Number randomized**  32  **Age**  6-13 years (range)  **Co-morbidities/Co-medications**  Yes, depressive disorder including dysthymia or major depressive disorder (15%) and anxiety diroder (42%)/No  **ADHD subtypes**  Not reported | **Arm 1**  MPH-SA low/medium dose: methylphenidate 10-20 mg/day  **Arm 2**  PINDO: pindolol 20-40 mg/day (excluded – betablockers are not eligible)  **Arm 3**  Placebo: placebo pill | **Treatment response** (as a dichotomous outcome)  Not reported/data not abstractable (e.g. for first 4 weeks)  **All-cause treatment discontinuation** (as a dichotomous outcome)  Yes, reported as study withdrawals  **Tolerability** (as a dichotomous outcome)  Not reported  **Serious adverse events** (as dichotomous outcome)  Not reported  **Specific adverse events** (as dichotomous outcome)  Loss of appetite/anorexia (as an adverse event)  Insomnia (as an adverse event)  Sleep disturbances (adverse event including insomnia + nightmares)  Anxiety (adverse event) |
| Conners et al., 1996^12^ | **Eligibility criteria**  Children had to be: scored a moderate illness severity (on the Child Diagnostic Scale), occurrence of mean parent and teacher scores of at least 1.5 (on the Conners Parent/Teacher Questionnaires), good physical health and without evidence of laboratory, EEG, ECG abnormalities. Exclusion criteria were: WISC-R IQ < 70; body weight < 20 kg; girls who had passed menarche; history or presence of seizure or tic disorders. All subjects had to be free of psychotic medications for a minimum of 14 days prior to study entry; heart disease, impaired renal function, impaired liver function, diabetes mellitus;  treatment with b3-sympatico-mimetics and/or parasympaticolytics for chronic obstructive lung disease and bronchial asthma; important changes in school or home situation expected during the course of the study period; psychosis or severe brain damage or other serious psychiatric disease as assessed by the child psychiatrist or psychologist; any concomitant disease that might interfere with drug evaluation; participation  in another pharmacological study or treatment with carnitine within 1 month prior to the screening visit. Before this trial, no boy received any carnitine treatment  **Diagnostic criteria**  DSM-III  **Number randomized**  109  **Age**  6-12 years (range)  **Co-morbidities/Co-medications**  Not reported (but possible a secondary diagnosis of conduct disorder)/No  **ADHD subtypes**  Not reported | **Arm 1**  BUP: bupropion 50-75 mg/day (3-6 mg/kg/day)  **Arm 2**  Placebo: placebo pill | **Treatment response** (as a dichotomous outcome)  Not reported/data not abstractable  **All-cause treatment discontinuation** (as a dichotomous outcome)  Yes, reported as study discontinuations  **Tolerability** (as a dichotomous outcome)  Yes, reported as study discontinuations due to AEs  **Serious adverse events** (as dichotomous outcome)  Not reported  **Specific adverse events** (as dichotomous outcome)  Not reported |
| Schachar et al., 1997^13^ | **Eligibility criteria**  Children had to exhibit pervasive ADHD (defined as 8 or more of the 14 DSM-III-R criteria for ADHD) in one setting and at least 5 ADHD criteria in the other setting; have a history of ADHD symptoms of at least 6 months’ durations before the age of 7 years; have an estimated Full Scale IQ > than 80; not have a primary anxiety or affective disorder  **Diagnostic criteria**  DSM-III-R  **Number randomized**  91  **Age**  6-12 years (range)  **Co-morbidities/Co-medications**  Yes, oppositional disorder (51%), anxiety (23%), conduct disorders (13%) /Not reported  **ADHD subtypes**  Not reported | **Arm 1**  MPH-SA high dose+BT: combination of methylphenidate 20-40 mg/day (mean: 31 mg/day or 1 mg/kg/day) plus behavioral therapy with parent training and support. Full details of the parent training strategy were not provided in the original paper  **Arm 2**  BT (parent training): parent training plus placebo pill | **Treatment response** (as a dichotomous outcome)  Not reported/data not abstractable  **All-cause treatment discontinuation** (as a dichotomous outcome)  Yes, reported as study discontinuations  **Tolerability** (as a dichotomous outcome)  Yes, reported as study dicontinuations due to AEs  **Serious adverse events** (as dichotomous outcome)  Not reported  **Specific adverse events** (as dichotomous outcome)  Not reported |
| Klein and Abikoff, 1997^14^ | **Eligibility criteria**  Children had to be attending elementary school; had to be free of neurological, tic, and psychotic disorders; had to obtain a verbal IQ or PIQ of at least 85 on the WISC-R; had o have parents willing to participate in the study after it was explained fully, and provide signed consent; could not be receiving psychostimulant treatment; and could not have had appropriate stimulant treatment in the past (for at least 4 weeks); had to meet severity criteria for hyperactivity  **Diagnostic criteria**  DSM-IV/III - As per the authors, study was initiated prior to the publication of the DSM-III, and was ongoing at the time of its publication. Diagnostic criteria could not be applied, but each child received a clinical psychiatric examination and the presence of pervasive symptoms of ADHD had to be confirmed utilizating standardized clinical evaluations with the child and parents. The study requirement for cross-situational hyperactivity brings the sample’s clinical features in line with the DSM-IV)  **Number randomized**  89  **Age**  6-12 years (range)  **Co-morbidities/Co-medications**  None /Not reported  **ADHD subtypes**  Not reported | **Arm 1**  MPH-SA high dose: methylphenidate 41 mg/day (mean: 1.6 mg/kg/day)  **Arm 2**  BT (parent and teacher training): parent and teacher training plus placebo pill. Operant behavior therapy both in the home (described by O’Leary 1972) and school individualized for each child. The behavior therapist met with both parents for at least 2 extended sessions prior to the formal treatment. General principles of learning theory were introduced and related to specific parental practices. The BT was recognized as a special program that would help both parents and the child learn new ways of responding. Parents were trained to provide concrete descriptions of actual behavior. Concomitant with parents sessions, interviews were held with teacher in school. The behavioral program was explained, and texts previously mentioned were offered. Therapists elicited concrete behaviors that interfered with the child’s functioning (e.g. not listening, calling out, leaving one’s seat). Parents and teachers were asked to keep frequency counts of specific target behaviors. Children were included in the weekly sessions held with parents and teachers. Initially, the child’s behavior was monitored every 30 minutes in both settings (and increased when possible). Parents reported reinforcements at the end of the day. Along with positive reinforcement for good behavior, punishments were used to decrease problematic behaviors (e.g. loss of privileges or time out). Other behavioral techinques were applied such as modeling and role-playing alternative coping in difficult social situations, and cognitive restructuring to alter parental attitudes. In most instanes, weekly sessions were augmented by phone contact between parents and therapist  **Arm 3**  MPH-SA high dose+BT: combination of methylphenidate 40 mg/day (mean: 1.5 mg/kg/day) plus behavioral therapy with parent and teacher training | **Treatment response** (as a dichotomous outcome)  Clinical Global Improvement (CGI-I) – e.g. responder considered “improved”: improved, much improved, completely well  **All-cause treatment discontinuation** (as a dichotomous outcome)  Yes, reported as study discontinuations  **Tolerability** (as a dichotomous outcome)  Yes, reported as study discontinuations due to AEs  **Serious adverse events** (as dichotomous outcome)  Not reported  **Specific adverse events** (as dichotomous outcome)  Cardiovascular event – tachycardia defined as adverse event |
| Van der Meere et al., 1999^15^ | **Eligibility criteria**  Not explicit  **Diagnostic criteria**  DSM-III-R  **Number randomized**  53  **Age**  7-12 years (range)  **Co-morbidities/Co-medications**  Yes, oppositional disorder (26%), conduct disorder (11%), depressive/anxiety disorders (4%)/No  **ADHD subtypes**  Not reported | **Arm 1**  MPH-SA low/medium dose: methylphenidate 0.6 mg/kg/day  **Arm 2**  CLON-SA: clonidine 4 µg/kg/day  **Arm 3**  Placebo: placebo pill | **Treatment response** (as a dichotomous outcome)  Clinical responders defined on the composite of a global judgment of both parent(s) and the teacher (independent of one another). Children were considered to be clinical responders when their behavior improved at home and/or at school. These data were collected in the seventh week of the trial  **All-cause treatment discontinuation** (as a dichotomous outcome)  Not reported  **Tolerability** (as a dichotomous outcome)  Not reported  **Serious adverse events** (as dichotomous outcome)  Not reported  **Specific adverse events** (as dichotomous outcome)  Not reported |
| MTA Cooperative group, 1999^16-19^ | **Eligibility criteria**  Children of either sex were between ages 7-9.9 years, in grades 1 through 4, and in residence with the same primary caretaker(s) for the last 6 months or longer. Exclusion criteria were limited to situations that would prevent families’ full participation in assessments or treatment, or that might require additional treatments incompatible with study treatments. The presence of comorbid conditions did not lead to exclusions per se  **Diagnostic criteria**  DSM-IV (using DISC criteria)  **Number randomized**  579  **Age**  7-9.9 years (range)  **Co-morbidities/Co-medications**  Yes, oppositional disorder (40%), conduct disorder (14%), anxiety disorders (34%), tics (11%)/No  **ADHD subtypes**  Combined (100%) | **Arm 1**  MPH-SA high dose: methylphenidate 38 mg/day (mean dose)  **Arm 2**  BT (child, parent and teacher training): Behavioral therapy included parent training, child-focused treatment, and school-based intervention organized and integrated with the school year. The parent training involved 27 groups (6 families per group) and 8 individual sessions pre family. It began weekly on randomization, concurrent with biweekly teacher consultation; both were tapered over time. The same therapist-consultant conducted parent training and teacher consultation, with each therapist-consultant having a case-load of 12 famlies. The child-focused treatment was a summer treatment program developed as a therapeutic summer camp. The 8-week, 5-days per week, 9-hours per day employed intensive behavioral interventions administered by counselors/aides, supervised by the same teacher-consultants who performed parent training and teacher consultation. Behavioral interventions were delivered in group-based recreational settings, and included a point system tied to specific rewards, time out, social reinforcement, modeling, group-problem solving, sports skills, and social skills training.  The school-based treatment had 2 components: 10 to 16 sessions of biweekly teacher consultation focused on class-room behavior management strategies and 12 weeks (60 school days) of a part-time, behaviorally trained, paraprofessional aide working directly with the child  **Arm 3**  MPH-SA high dose+BT: combination of both methylphenidate (31 mg/day) and BT with child, parent and teacher training (as described before)  **Arm 4**  Standard care (control): community care participants received none of the study treatments, but were provided a report of their initial study assessments, along with a list of community mental health resources. Most community care patients (67%) received ADHD medications from their own provider | **Treatment response** (as a dichotomous outcome)  Response defined as “successful treatment” by averaging the parent and teacher ratings of total symptoms on the Swanson, Nolan and Pelham version IV (SNAP-IV) scale and low symptom severity (“just a little"). Response defined as an excellent response (and thus “success”) defined by cutoff of ≤ 1.0 on the SNAP-IV score obtained at the end of treatment.  **All-cause treatment discontinuation** (as a dichotomous outcome)  Yes, reported as study withdrawals/drop-outs (“attrition rates”)  **Tolerability** (as a dichotomous outcome)  Not reported  **Serious adverse events** (as dichotomous outcome)  Not reported  **Specific adverse events** (as dichotomous outcome)  Not reported |
| Connor et al., 2000^20^ | **Eligibility criteria**  Children required to meet comorbid ADHD with either aggressive oppositional defiant disorder or conduct disorder; have a score of 1.5 SD above the mean of age and gender on the Parent CBCL Attention scale (T score ≥ 65) and a score on the Teacher Child Attention Problem Rating scale (CAPS) of at least the 93^rd^ percentile. They were also required to have a score 1.5 SD above the mean of age and gender on the Teacher CBCL Delinquency or Aggresion Problems scale  **Diagnostic criteria**  DSM-III-R  **Number randomized**  24  **Age**  6-16 years (range)  **Co-morbidities/Co-medications**  Yes, either oppositional disorder or conduct disorder (100%)/ No (46% history of stimulants, but all subjects were free of medication at baseline)  **ADHD subtypes**  Not reported | **Arm 1**  MPH-SA high dose: methylphenidate 32.5 mg/day  **Arm 2**  CLON-SA: clonidine 0.2 mg/day  **Arm 3**  MPH-SA high dose+CLON-SA: combination of both methylphenidate (35 mg/day) and clonidine (0.2 mg/day) | **Treatment response** (as a dichotomous outcome)  Not reported/data not abstractable  **All-cause treatment discontinuation** (as a dichotomous outcome)  Yes, defined as study drop-outs  **Tolerability** (as a dichotomous outcome)  Not reported  **Serious adverse events** (as dichotomous outcome)  Not reported  **Specific adverse events** (as dichotomous outcome)  Cardiovascular event – new-onset bradycardia defined as pulse < 60 bpm (as assessed by cardiology) |
| Pliszka et al., 2000^21^ | **Eligibility criteria**  Children with ADHD in grades 1 through 5 who had no other medical illnesses and were not currently being treated with any other nonstimulant psychotropic medication. Not diagnostic criteria for major depression episode, manic episode, or tic disorder. In addition, the child could not have any history of psychosis or have signs of psychosis or significantly depressed mood on the mental status examination  **Diagnostic criteria**  DSM-III-R/based DISC criteria for ADHD (confirmed by the psychiatrist)  **Number randomized**  58  **Age**  6-11 years (range according to grade level in the USA e.g. grade 1 to 5)  **Co-morbidities/Co-medications**  Yes, oppositional disorder (62%), conduct disorder (10%), anxiety disorder (12%)/No  **ADHD subtypes**  Not reported | **Arm 1**  MPH-SA low/medium dose: methylphenidate 25-50 mg/day (mean dose: 25 mg/day)  **Arm 2**  MIX-AMPH: mixed amphetamine salts (Adderall) 15-30 mg/day (mean dose: 12.5 mg/day)  **Arm 3**  Placebo: placebo pill | **Treatment response** (as a dichotomous outcome)  Global improvement based on Clinical Global Impression Improvement (CGI-I) scale. Subjects with improvement of 1 or 2 were considered responders  (rating evaluations reported by the psychiatrist)  **All-cause treatment discontinuation** (as a dichotomous outcome)  Yes, reported as study drop-outs  **Tolerability** (as a dichotomous outcome)  Yes, reported as study drop-outs due to AEs  **Serious adverse events** (as dichotomous outcome)  Not reported  **Specific adverse events** (as dichotomous outcome)  Loss of appetite/anorexia (as an adverse event e.g. “appetite loss”)  Anxiety (as an adverse event e.g. “anxious”) |
| Prince et al., 2000^22^ | **Eligibility criteria**  Outpatient children and adolescents with ADHD between 6-17 years ascertained from clinical referrals to a pediatric psychopharmacology clinic. Exclusions: any clinically significant chronic medical condition, including a personal history of cardiovascular disease, a family history of nongeriatric cardiac disease, mental retardation (IQ < 70), organic brain disorders, seizures, pregnant or nursing females, psychotic disorder, bipolar disorder, current abuse or dependence on drugs and/or alcohol within the past 6 months, and current treatment with psychotropics (including anticonvulsants for behavioral control)  **Diagnostic criteria**  DSM-IV  **Number randomized**  23  **Age**  6-17 years (range)  **Co-morbidities/Co-medications**  Yes, oppositional disorder (54%), conduct disorder (11%), anxiety disorder (31%), depression (31%), learning disorders (20%)– Yes (all patients were previous responders to antidepressant during open phase, and 57% had previous ADHD medication)  **ADHD subtypes**  Not reported | **Arm 1**  NT: nortriptyline 80 mg/day (1.8 mg/kg/day)  **Arm 2**  Placebo: placebo pill | **Treatment response** (as a dichotomous outcome)  Global improvement based on Clinical Global Impression Improvement (CGI-I) scale. Subjects with improvement of “very much” or “much” improved were considered responders (rating evaluations reported by the psychiatrist)  **All-cause treatment discontinuation** (as a dichotomous outcome)  Not reported  **Tolerability** (as a dichotomous outcome)  Not reported  **Serious adverse events** (as dichotomous outcome)  Not reported  **Specific adverse events** (as dichotomous outcome)  Not reported |
| Michelson et al., 2001^23-25^ | **Eligibility criteria**  Age 8-18 years of age with ADHD, symptom severity score at least 1.5 SD above age and gender norms on the ADHD-RS for the total score or either the inattentive or the hyperactive/impulsive subscales. Important exclusion criteria included IQ < 80, serious medical illness, comorbid psychosis or bipolar disorder, history of seizure disorder, orr ongoing use of psychoactive medications other than the study drug  **Diagnostic criteria**  DSM-IV  **Number randomized**  297  **Age**  8-18 years (range)  **Co-morbidities/Co-medications**  Yes, oppositional disorder (38%), anxiety disorder (0.3%), depression (0.3%) – Yes (70% had previous ADHD medication, but ongoing use of psychoactive medications was an exclusion criteria)  **ADHD subtypes**  Combined (67%), Inattentive (31%), Hyperactive/impulsive (2%) | **Arm 1**  ATX: 3 doses of atomoxetine (0.5, 1.2 or 1.8 mg/kg/day)  **Arm 2**  Placebo: placebo pill | **Treatment response** (as a dichotomous outcome)  Not reported/data not abstractable  **All-cause treatment discontinuation** (as a dichotomous outcome)  Yes, reported as study discontinuations  **Tolerability** (as a dichotomous outcome)  Yes, reported as study discontinuations due to AEs  **Serious adverse events** (as dichotomous outcome)  Yes, reported (unpublished data)  **Specific adverse events** (as dichotomous outcome)  Loss of appetite/anorexia (as an adverse event)  Insomnia (as an adverse event) |
| Scahill et al., 2001^26^ | **Eligibility criteria**  Age 7-15 with ADHD (any type), tic disorder (any type), and a score ≥ 1.5 SD unit for age and gender on the 10-item Conners hyperactivity index rated by the teacher or a parent. Exclusion criteria included evidence of current major depression, generalized anxiety disorder, separation anxiety disorder, or psychotic symptoms (based on all available information); WISC-R IQ < 70; and a prior adequate trial of guanfacine (dose of ≥ 1.5 mg/day for at least 2 weeks). Subjects had to be free of all psychotropic medication for at least 2 weeks and free of any significant medical problem. Children with moderate or more severe tic symptoms were also excluded because of their likely need for pharmacological treatment  **Diagnostic criteria**  DSM-IV  **Number randomized**  34  **Age**  8-14 years (range)  **Co-morbidities/Co-medications**  Yes, tic disorders (100%)/No  **ADHD subtypes**  Combined (100%) | **Arm 1**  GUAN-SA: guanfacine short acting 3 mg/day  **Arm 2**  Placebo: placebo pill | **Treatment response** (as a dichotomous outcome)  Global improvement based on Clinical Global Impression Improvement (CGI-I) scale. Subjects with improvement of “very much” or “much” improved were considered responders (rated by clinicians); ADHD-RS improvement from baseline in total score (completed by teacher), and Parent Conners Hyperactivity index improvement from baseline (rated by parents)  **All-cause treatment discontinuation** (as a dichotomous outcome)  Yes, reported as study withdrawals  **Tolerability** (as a dichotomous outcome)  Yes, reported as study withdrawals due to AEs  **Serious adverse events** (as dichotomous outcome)  None  **Specific adverse events** (as dichotomous outcome)  Sleep disturbances (as an adverse event e.g. “mid-sleep awakening” event)  Cardiovascular event – blood pressure decrease (as an adverse event e.g. decrease in blood pressure, with clinically significant changes = drop of 1 SD in systolic or diastolic blood pressure) |
| Sonuga-Barke et al., 2001^27^ | **Eligibility criteria**  Only children who met clinically validated cutoffs on the Parental Account of Childhood (PACS) ADHD/Hyperkinesis scale and whose parents reported that their condition was associated with impairment siginificant enough to warrant clinical intervention were included. Children were excluded from the trial if their parents had a serious mental illness, they had a serious learning disability, or they had a previous diagnosis for an unrelated mental health condition  **Diagnostic criteria**  DSM-IV (modified for preschool age)  **Number randomized**  78  **Age**  2-4 years (range)  **Co-morbidities/Co-medications**  Yes, conduct disorders (29%)/Not reported  **ADHD subtypes**  Not reported | **Arm 1**  BT (parent training): A structured 8-week program involving 1-hour weekly visits by one of two specially trained health visitor therapists. Mothers were the recipients of the training in all cases, and were educated about ADHD and introduced to a range of behavioral strategies for increasing attention and behavioral organization and reducing defiant and difficult behavior. Progress was monitored on a weekly bass, and there were regular reviews of previously coveted issues and strategies. Parents completed a behavior diary  **Arm 2**  Parent counseling with support (control): A structured 8-week program involving 1-hour weekly visits by one of two specially trained health visitor therapists. Parents received no training in behavioral strategies. They were, however, given the opportunity to explore issues of concern to them and to discuss their feelings about their child and the impact that the child had on family in a nondirective, nonthreatening environment. To maintain dialogue, the behavior diary was used to structure discussion  **Arm 3**  Waiting list: Wating list children received no contact with clinical services during the 23 weeks of the trial | **Treatment response** (as a dichotomous outcome)  The authors assessed clinical significance of treatment response in terms of “recovery” – based on the formula developed by Jacobson and Truax (1991) for PACS ADHD core symptoms – when scores cross the clinical threshold at the midpoint between the means of the clinical and the normal populations (e.g. PACS score < 15.65)  **All-cause treatment discontinuation** (as a dichotomous outcome)  Not reported  **Tolerability** (as a dichotomous outcome)  Not reported  **Serious adverse events** (as dichotomous outcome)  Not reported  **Specific adverse events** (as dichotomous outcome)  Not reported |
| Voigt et al., 2001^28^ | **Eligibility criteria**  Children treated successfully with stimulant medication and with clear historical evidence of clinically significant impoairment in social or academic functioning. Children who met criteria for oppositional defiant disorder or conduct disorder were not excluded  **Diagnostic criteria**  DSM-IV  **Number randomized**  63  **Age**  6-12 years (range)  **Co-morbidities/Co-medications**  Yes, oppositional disorder (52%), conduct disorder (15%)/Yes (all received stimulants)  **ADHD subtypes**  Combined (89%) | **Arm 1**  MPH-SA low/medium dose: methylphenidate 29 mg/day (median dose: 25 mg/day)+placebo  **Arm 2**  MPH-SA low/medium dose+PUFA: methylphenidate 29 mg/day (median dose: 20 mg/day) + polyunsaturated fatty acid (omega-3 fatty acid: docosahexaenoic acid 345 mg/day) | **Treatment response** (as a dichotomous outcome)  Not reported/data not abstractable  **All-cause treatment discontinuation** (as a dichotomous outcome)  Yes, reported as study drop-outs  **Tolerability** (as a dichotomous outcome)  None  **Serious adverse events** (as dichotomous outcome)  Not reported  **Specific adverse events** (as dichotomous outcome)  Not reported |
| Wolraich et al., 2001^29^ | **Eligibility criteria**  Boys and girls, ages 6-12 years, with any subtype of ADHD; patients who were taking MPH or had taken it in the past had to have been on a total daily MPH dose of at least 10 mg but no more than 60 mg; patients had to agree to take the supplied study drug as the only medication for  ADHD during the 4-week study period; IQ > 70.  Patients were excluded if: acute or serious chronic disease; hypersensitivity to methylphenidate; significant adverse experiences from methylphenidate; taking a medication that would interfere with safe administration of  Methylphenidate; glaucoma, Tourette’s syndrome, ongoing seizure disorder or psychotic disorder; girls who had reached menarche  **Diagnostic criteria**  DSM-IV  **Number randomized**  282  **Age**  6-12 years (range)  **Co-morbidities/Co-medications**  Yes, oppositional disorder (42%), conduct disorder (11%), depression (1%), anxiety (1%), tics (5%)/Yes (68% previous treatment with methylphenidate, 20% treatment naïve)  **ADHD subtypes**  Combined (73%), innatentive (19%) and hyperactive/impulsive (7%) | **Arm 1**  MPH-SA low/medium dose: methylphenidate 29.5 mg/day (mean dose: 0.9 mg/kg/day)  **Arm 2**  MPH-LA low/medium dose: methylphenidate long acting (OROS, Concerta®) 34.3 mg/day (mean dose: 1.1 mg/kg/day)  **Arm 3**  Placebo | **Treatment response** (as a dichotomous outcome)  Global improvement based on Clinical Global Impression Improvement (CGI-I) scale. Subjects with improvement ratings of “very much” or “much” improved were considered responders (rated by clinicians); Global assessment of efficacy at the end of study was also evaluated by means of rating the patient’s behavior and attention on a 4-point scale (0=poor, 1=fair; 2=good; 3=excellent) (rated by parents and teachers)  **All-cause treatment discontinuation** (as a dichotomous outcome)  Yes, reported as study drop-outs  **Tolerability** (as a dichotomous outcome)  Yes, reported as drop-outs due to AEs  **Serious adverse events** (as dichotomous outcome)  Not reported  **Specific adverse events** (as dichotomous outcome)  Decreased apetite/anorexia (as an adverse event “eating less”) |
| Biederman et al., 2002^31,32^ | **Eligibility criteria**  Children 6-12 years, were required to be in school setting in which the same teacher was able to make assessments of both morning and afternoon behaviors; participants who were home-schooled were not permitted to enroll. Children were either to be responsive to stimulants or naïve to stimulant treatment. Participants incaple of understanding or following the instructions given in the study, known nonresponders to stimulant medication, and those with comorbid psychiatric diagnois (psychosis, bipolar illness, pervasive developmentl disorder, severe obsessive compulsive disorder, severe depressive or severe anxiety disorder) were excluded from the study. Participants with a history of seizure, tic disorder, or a family of Tourette’s disorder, those with a documented allergy or intolerance to Adderall, and participants taking clonidine, anticonvulsivant drugs, pemoline (within 30 days), medications that affect blood pressure or heart rate, steroids, or other medications that have central nervous system effects or affect performance ( such as sedating antihistamines and decongestant sympathomimetics) also were excluded Other exclusion criteria were: concurrent chronic or acute illness or condition that might confound results or increase risk to the participant, history of suspected substance abuse disorder, or living with someone with a current diagnosed substance abuse disorder  **Diagnostic criteria**  DSM-IV  **Number randomized**  584  **Age**  6-12 (range)  **Co-morbidities/Co-medications**  Yes, with comorbid conditions unspecified (30%)/No  **ADHD subtypes**  Combined (93%), hyperactive/impulsive (5%), inattentive (2%) | **Arm 1**  MIX-AMPH-LA: mixed amphetamine salts long acting (Adderall XR) 10, 20 or 30 mg/day  **Arm 2**  Placebo: placebo pill | **Treatment response** (as a dichotomous outcome)  Global assessment of overall improvement by the investigator (the Clinical Global Impressions Scale improvement, GGI-I) and by parents (the Parent Global Assessment for improvement, PGA). Extracted from figure 6  **All-cause treatment discontinuation** (as a dichotomous outcome)  Yes, reported as study discontinuations  **Tolerability** (as a dichotomous outcome)  Yes, reported as study discontinuations due to AEs  **Serious adverse events** (as dichotomous outcome)  Not reported  **Specific adverse events** (as dichotomous outcome)  Loss of appetite/anorexia (as an adverse event)  Insomnia (adverse effect as defined by the authors)  Cardiovascular event – blood pressure increase (e.g. SBP > 130 mm Hg)  Mild ECG abnormalities (as an adverse event) |
| Bor et al., 2002^33^ | **Eligibility criteria**  The target child was aged 36-48 months; mothers rated their child’s behavior as being in the elevated range on the Eyberg Child Behavior Inventory (ECBI; intensity score ≥ 127 or problem score ≥ 11); the child was not currently having regular contact with another professional or agency or taking medication for behavioral problems; no evidence of developmental disorder (e.g. language disorder, autism) or significant health impairment; the parents were not currently reeiving therapy for psychological problems, were not intellectually disabled, and reported they were able to read the newspaper without assistance; all families had at least one family adversity factor  **Diagnostic criteria**  DSM-IV  **Number randomized**  87  **Age**  3 years  **Co-morbidities/Co-medications**  Yes, ADHD with co-ocurring disruptive behavior/No  **ADHD subtypes**  Not reported | **Arm 1**  BT (parent training): Two groups were lumped in arm 1, standard parent training program (SBFI) and the enhanced parent training program (EBFI). SBFI consisted of an intensive, well-validated, parent training intervention known as Standard Triple P – Positive Parenting Program – during 15 weeks. EBFI comprised 3 elements: parent training (Standard Triple P), partner support training and coping skills training during 17 weeks  **Arm 2**  Waiting list: Wating list children received no contact with clinical services during 15 weeks | **Treatment response** (as a dichotomous outcome)  Not reported/data not abstractable  **All-cause treatment discontinuation** (as a dichotomous outcome)  Yes, reported as study discontinuations  **Tolerability** (as a dichotomous outcome)  Not reported  **Serious adverse events** (as dichotomous outcome)  Not reported  **Specific adverse events** (as dichotomous outcome)  Not reported |
| Greenhill et al., 2002^34^ | **Eligibility criteria**  Children 6-16 years of age; primary diagnosis of ADHD, combined subtype or predominantly hyperactive-impulsive subtype; did not respond to placebo with a reduction of ADHD symptoms during washout period; first-grade or higher school setting in which a single teacher could assess  Behavior; blood pressure, heart rate and oral temperature had to be within normal range; did not respond to placebo with a reduction of ADHD symptoms during washout period; first-grade or higher school setting in which a single teacher could assess behavior; blood pressure, heart rate and oral temperature had to be within normal range. Exclusion criteria were: comorbid psychiatric diagnosis; history of seizure or tic disorder; family history of Tourette’s syndrome; IQ < 80; inability to follow or understand study instructions; female who had undergone menarche; use of amphetamines, pemoline or an investigational drug within 30 days of study entry; concomitant use of clonidine, anticonvulsant drugs or medications known to affect blood pressure, heart rate or CNS function; hyperthyroidism or glaucoma; concurrent chronic or acute illness (e.g. allergic rhinitis, severe cold) or disability that could confound study results; failed a previous trial of stimulants for ADHD; requiring a third daily dose in the afternoon or evening; documented allergy or intolerance to methylphenidate; living with anyone who currently had substance abuse disorder (excluding dependency)  **Diagnostic criteria**  DSM-IV  **Number randomized**  321  **Age**  6-16 years (range)  **Co-morbidities/Co-medications**  None/Yes (64% previously treated; concomitant use of clonidine, anticonvulsant drugs and medications known to affect blood pressure and heart rate was not allowed)  **ADHD subtypes**  Combined subtype or predominantly hyperactive-impulsive | **Arm 1**  MPH-INT high dose: methylphenidate modified-release with mean dose of 40.7 mg/d (1.28 mg/kg/day)  **Arm 2**  Placebo: placebo pill | **Treatment response** (as a dichotomous outcome)  Clinician-rated Clinical Global Impression (CGI-S) Scale which includes scales rating of Global Severity (1=not ill, to 7=extremely ill) and Global Improvement (CGI-I) wich includes scales rating of improvement (1=very much improved, to 7=very much worse – e.g. responder considered very much [1] or much improved [2]).  **All-cause treatment discontinuation** (as a dichotomous outcome)  Yes, reported as study withdrawls  **Tolerability** (as a dichotomous outcome)  Yes, reported as withdrawals due to AEs  **Serious adverse events** (as dichotomous outcome)  None  **Specific adverse events** (as dichotomous outcome)  Loss of appetite/anorexia (as an adverse event)  Insomnia (as an adverse event) |
| Lehmkuhl et al., 2002^35-37^ | **Eligibility criteria**  Children 6-16 years of age; attendance at an elementary or secondary school; IQ > 85; body weight > 20 kg; according to the class teacher, occurrence of considerable ADHD symptoms over  the previous 3 school days (provided the mean score on the aggregate Fremdbeurteilungsbogen für Hyper kinetische Störunge rating scale was > 1.0). Exclusions: diagnosis of depression or anxiety; tics or Tourette’s syndrome or family occurrence of tic disorder; pervasive developmental disorder; psychosis; history of seizures or evidence on the EEG of risk of seizures; pre-treatment of patients with methylphenidate or other psychostimulants up to 3 weeks before the study; lack of knowledge of the German language of the patient or legal guardian  **Diagnostic criteria**  DSM-IV  **Number randomized**  85  **Age**  6-15 years (range)  **Co-morbidities/Co-medications**  Yes, oppositional disorder (52%), conduct disorder (9%), depression (1%)/No (5% co-medication)  **ADHD subtypes**  Combined (74%), innatentive (25%) | **Arm 1**  MPH-LA high dose: methylphenidate sustained-release 20 to 60 mg/day  **Arm 2**  Placebo | **Treatment response** (as a dichotomous outcome)  ADHD-RS by teacher (in German, FBB-HKS)/responders in counts and percentages  **All-cause treatment discontinuation** (as a dichotomous outcome)  Yes, reported as study withdrawals  **Tolerability** (as a dichotomous outcome)  Yes, reported as withdrawals due to AEs  **Serious adverse events** (as dichotomous outcome)  Yes, reported  **Specific adverse events** (as dichotomous outcome)  Not reported |
| Kratochvil et al., 2002^38,39^ | **Eligibility criteria**  Boys aged 7-15 years and girls aged 7-9 years; severity score of at least 1.5 SD above age and gender norms on the ADHD-IV Ratins Scale-Parent version:investigator administered. Exclusion criteria were: history of bipolar or psychotic disorders, motor tics or family history of Tourette syndrome, substance abuse, non-response to a previous trial of methylphenidate, and serious medical illness. Other concurrent psychiatric diagnoses did not exclude patients from the trial  **Diagnostic criteria**  DSM-IV  **Number randomized**  228  **Age**  7-15 years (range)  **Co-morbidities/Co-medications**  Yes, oppositional disorder (55%), depression (9%), elimination disorder (15%)/Not reported  **ADHD subtypes**  Combined (76%), innatentive (23%) | **Arm 1**  MPH-SA low/medium dose: methylphenidate 0.85 mg/kg/day  **Arm 2**  ATX: atomoxetine 1.40 mg/kg/day | **Treatment response** (as a dichotomous outcome)  Clinician-rated Clinical Global Impression Severity (CGI-S) scale and Global Improvement (CGI-I) wich includes scales rating of improvement (1=very much improved, to 7=very much worse – e.g. responder considered very much [1] or much improved [2]).  **All-cause treatment discontinuation** (as a dichotomous outcome)  Yes, reported as study discontinuations  **Tolerability** (as a dichotomous outcome)  Yes, reported as discontinuations due to AEs  **Serious adverse events** (as dichotomous outcome)  None  **Specific adverse events** (as dichotomous outcome)  Decreased weight (as an adverse event e.g. “weight loss”)  Loss of appetite/anorexia (as an adverse event)  Insomnia (as an adverse event)  Cardiovascular event – Tachycardia (as an adverse event)  Palpitations (as an adverse event) |
| Michelson et al., 2002^40,41^ | **Eligibility criteria**  Children and adolescents with a symptom severity threshold (score at least 1.5 SD above age and gender norms as assessed by the investigator-administered and –scored parent version of the ADHD RS-IV). Important exclusion criteria: serious medical illness, a history of psychosis or bipolar disorder, alcohol or drug abuse within the past 3 months, and ongoing use of psychoactive medications other than the study drug)  **Diagnostic criteria**  DSM-IV  **Number randomized**  171  **Age**  6-16 years (range)  **Co-morbidities/Co-medications**  Yes, oppositional disorder (20%), depression (2%), phobia (3%), anxiety (0.6%)/Yes  **ADHD subtypes**  Combined (58%), innatentive (41%), hyperactive/impulsive (2%) | **Arm 1**  ATX: atomoxetine 1.40 mg/kg/day  **Arm 2**  Placebo: placebo pill | **Treatment response** (as a dichotomous outcome)  ADHD-RS ≥ 25% reduction from baseline in total score (rater: parents)  Clinician-rated Clinical Global Impression Severity (CGI-S) scale (score 1 or 2)  **All-cause treatment discontinuation** (as a dichotomous outcome)  Yes, reported as study discontinuations  **Tolerability** (as a dichotomous outcome)  Yes, reported as discontinuations due to AEs  **Serious adverse events** (as dichotomous outcome)  None  **Specific adverse events** (as dichotomous outcome)  Loss of appetite/anorexia (as an adverse event) |
| Spencer et al., 2002a^42,30^ | **Eligibility criteria**  Children had an ADHD-RS at least 1.5 SD above the age and gender norms for their diagnostic subtype or the total score for the combined subtype. Patients were excluded if based on their genotype were characterized as poor metabolizers of CYP2D6; had documented history of bipolar I or II disorder or any history of psychosis; had any organic brain disease or a history of any seizure disorder; were taking any psychotropic medication; had any history of alcohol or drug abuse within the past 3 months; or had significant (prior or current) medical conditions  **Diagnostic criteria**  DSM-IV  **Number randomized**  147  **Age**  7-12 years (range)  **Co-morbidities/Co-medications**  Yes, oppositional disorder (39%), depression (3%), phobia (12%), anxiety (3%)/No  **ADHD subtypes**  Combined (80%), innatentive (19%), hyperactive/impulsive (1%) | **Arm 1**  MPH-SA high dose: methylphenidate up to 60 mg/day (or 1.5 mg/kg/day)  **Arm 2**  ATX: atomoxetine up to 2.0 mg/kg/day  **Arm 3**  Placebo: placebo pill | **Treatment response** (as a dichotomous outcome)  ADHD-RS ≥ 25% reduction from baseline in total score (rater: parents) – not reported for MPH-SA  **All-cause treatment discontinuation** (as a dichotomous outcome)  Yes, reported as study discontinuations  **Tolerability** (as a dichotomous outcome)  Yes, reported as discontinuations due to AEs – pooled data for Spencer 2002a and 2002b (not reported for MPH)  **Serious adverse events** (as dichotomous outcome)  Not reported for MPH-SA nor ATX  **Specific adverse events** (as dichotomous outcome) - pooled data for Spencer 2002a and 2002b (not reported for MPH)  Decreased weight (as an adverse event)  Loss of appetite/anorexia (as an adverse event)  Insomnia (as an adverse event) |
| Spencer et al., 2002b^42,30^ | **Eligibility criteria**  Children had an ADHD-RS at least 1.5 SD above the age and gender norms for their diagnostic subtype or the total score for the combined subtype. Patients were excluded if based on their genotype were characterized as poor metabolizers of CYP2D6; had documented history of bipolar I or II disorder or any history of psychosis; had any organic brain disease or a history of any seizure disorder; were taking any psychotropic medication; had any history of alcohol or drug abuse within the past 3 months; or had (significant prior or current) medical conditions  **Diagnostic criteria**  DSM-IV  **Number randomized**  144  **Age**  7-12 years (range)  **Co-morbidities/Co-medications**  Yes, oppositional disorder (39%), depression (3%), phobia (12%), anxiety (3%)/Yes (all prior-stimulant exposure)  **ADHD subtypes**  Combined (80%), innatentive (19%), hyperactive/impulsive (1%) | **Arm 1**  ATX: atomoxetine up to 2.0 mg/kg/day  **Arm 2**  Placebo: placebo pill | **Treatment response** (as a dichotomous outcome)  ADHD-RS ≥ 25% reduction from baseline in total score (rater: parents)  **All-cause treatment discontinuation** (as a dichotomous outcome)  Yes, reported as study discontinuations  **Tolerability** (as a dichotomous outcome)  Yes, reported as discontinuations due to AEs – pooled data for Spencer 2002a and 2002b  **Serious adverse events** (as dichotomous outcome)  Not reported for ATX  **Specific adverse events** (as dichotomous outcome) - pooled data for Spencer 2002a and 2002b  Decreased weight (as an adverse event)  Loss of appetite/anorexia (as an adverse event)  Insomnia (as an adverse event) |
| Spencer et al., 2002c^43^ | **Eligibility criteria**  Children with combined type ADHD and chronic motor tic disorder, chonic vocal tic disorder, or Tourette disorder. Exclusions: significant chronic medical conditions or abnormal baseline laboratory values, low IQ (IQ<75), clinically unstable psychiatric conditions (ie, suicidality), current bipolar disorder, psychosis, drug or alcohol abuse or dependence, or current use of other psychotropic drugs. We also excluded pregnant or nursing females. Although patients with a personal history of cardiac disease or a family history of nongeriatric cardiac disease were specifically excluded by the protocol, no patient had to be excluded because of this limitation  **Diagnostic criteria**  DSM-IV  **Number randomized**  41  **Age**  5-17 years (range)  **Co-morbidities/Co-medications**  Yes, all chronic tic disorders (100%), oppositional disorder (52%), conduct disorders (32%), depression (41%), anxiety (51%) – Yes (46% received stimulants)  **ADHD subtypes**  Combined (100%) | **Arm 1**  DESIP: Desipramine up to 3.5 mg/kg/day  **Arm 2**  Placebo: placebo pill | **Treatment response** (as a dichotomous outcome)  ADHD-RS ≥ 30% reduction from baseline in total score (rater: parents); Clinical Global Impression Improvement (CGI-I) scale (score 1 or 2, much or very much improved)  **All-cause treatment discontinuation** (as a dichotomous outcome)  Yes, reported as study discontinuations  **Tolerability** (as a dichotomous outcome)  Yes, reported as study discontinuations due to AEs  **Serious adverse events** (as dichotomous outcome)  None  **Specific adverse events** (as dichotomous outcome)  Loss of appetite/anorexia (as an adverse event)  Sleeping disturbances (as an adverse event eg. “difficulty sleeping”) |
| TSSG, 2002^44^ | **Eligibility criteria**  Children 7-14 years in school; designated teacher in daily direct contact with the participant had to indicate the presence of a sufficient number of ADHD symptoms (rated as “pretty much” or “very  much”) in the classroom setting using the Disruptive Behavior Disorders Rating Scale (updated to DSM-IV); and had to rate the severity of ADHD symptoms above specified cutoff scores (boys: grade 2 to 3 = 10, grade ≥ 4 = 9; girls: grade 2 to 3 = 7, grade ≥ 4 = 6) on the Iowa Conners’ Teacher Rating Scale; Investigator’s rating of global functioning on the C-GAS had to be 70 (indicating difficulty in ≥ 1 area, such as school); DSM-IV for Tourette’s disorder, chronic motor tic disorder or chronic vocal tic disorder. Exclusions: secondary tic disorder (tardive tics, neuroacanthocytosis, Huntington disease); major depression, pervasive developmental disorder, autism, psychosis, anorexia nervosa, bulimia, serious cardiovascular or other medical disorder that would preclude  the safe use of methylphenidate or clonidine, impaired renal function, pregnancy; mental retardation; the following cardiac conditions: prolonged QTc interval (> 440 milliseconds),  high-grade ventricular ectopy, AV block > 1 degree, bundle branch block, intraventricular conduction block (100 milliseconds), pacemaker rhythm or heart rate< 60 on the electrocardiogram, cardiomyopathy, complex heart disease, aortic or pulmonary stenosis, family history of long QT syndrome, cardiomyopathy or premature sudden death (age 45 years), history of syncope and blood pressure < 2 SD from age- and sex-adjusted mean; other medications for treatment of ADHD, tics or other associated behavioural symptoms. Any such treatment had to be discontinued ≥ 6 weeks (2 weeks for MPH) before enrolment  **Diagnostic criteria**  DSM-IV  **Number randomized**  136  **Age**  7-14 years (range)  **Co-morbidities/Co-medications**  Yes, all tic disorders/Tourette’s syndrome (100%), oppositional disorder (38%), conduct disorders (9%), depression (5%), anxiety (9%) – Yes (58% received stimulants)  **ADHD subtypes**  Combined (26%), inattentive (72%), hyperactive/impulsive (2%) | **Arm 1**  MPH-SA low/medium dose: methylphenidate 26 mg/day  **Arm 2**  CLON-SA: clonidine 0.25 mg/day  **Arm 3**  MPH-SA low/medium dose+CLON-SA: combination of both methylphenidate (26 mg/day) and clonidine (0.25 mg/day)  **Arm 4**  Placebo: placebo pill | **Treatment response** (as a dichotomous outcome)  Rating of clinical change/improvement using Clinical Global Impression (CGI) scale for ADHD (rater: clinician, parents, teachers; independently)  **All-cause treatment discontinuation** (as a dichotomous outcome)  Yes, reported as study withdrawals  **Tolerability** (as a dichotomous outcome)  Yes, reported as study withdrawals due to AEs (e.g. ECG change)  **Serious adverse events** (as dichotomous outcome)  Not reported  **Specific adverse events** (as dichotomous outcome)  Not reported |
| van Oudheusden et al., 2002^45^ | **Eligibility criteria**  Boys aged between 6-13 years, outpatients living in a family home; a diagnosis of ADHD according to DSM IV for the combined group with  attention deficit/hyperactivity/impulsiveness based on the combined use of clinical interview of child psychiatrist, child psychologist and pediatrician according to their routine procedures and a CBCL score in the clinical area on the total score or at least one the following sub-scores: attention problems, delinquency and aggressive behavior; not receiving any stimulant medication; attending normal elementary school; (5) healthy on physical examination and laboratory tests  **Diagnostic criteria**  DSM-IV  **Number randomized**  23  **Age**  6-13 years (range)  **Co-morbidities/Co-medications**  Not reported/Not receiving stimulants  **ADHD subtypes**  Combined (100%) | **Arm 1**  L-CARN: l-carnitine 100 mg/kg/day  **Arm 2**  Placebo: placebo pill | **Treatment response** (as a dichotomous outcome)  Not reported for the first period (before crossover)  **All-cause treatment discontinuation** (as a dichotomous outcome)  Yes, reported as study withdrawals  **Tolerability** (as a dichotomous outcome)  Yes, reported as study withdrawals due to AEs  **Serious adverse events** (as dichotomous outcome)  Not reported  **Specific adverse events** (as dichotomous outcome)  Not reported |
| Hazell et al., 2003^46^ | **Eligibility criteria**  Children aged 6-14 years of age with ADHD and comorbid ODD or CD who had been treated for a minimum of 3 months with either MPH or DEXAM and attended psychiatric or pediatric clinics. Comorbid anxiety or depression was permitted, but patients with obsessional symptoms, movement disorders, or psychosis were excluded because these are contraindications for persisting  with psychostimulant medication. Borderline intellectual functioning and mild pervasive developmental disorder were permitted because they represent reasonably common comorbidities with ADHD, but children were excluded if they met criteria for mental retardation (IQ < 70). Children were also excluded if they had a history as determined by a physician of cardiac anomalies or other medical contraindications to the prescription of clonidine  **Diagnostic criteria**  DSM-IV  **Number randomized**  67  **Age**  6-14 years (range)  **Co-morbidities/Co-medications**  Yes, all with oppositional disorder or conduct disorders, anxiety (6%), pervasive disorders (2%) – Yes (100% prior stimulants)  **ADHD subtypes**  Not reported | **Arm 1**  MPH-SA or DEXAM low/medium dose: methylphenidate or dexamphetamine (0.67 mg/kg/day of stimulants in methylphenidate equivalents)  **Arm 2**  MPH-SA or DEXAM low/medium dose+CLON-SA: combination of both methylphenidate or dexamphetamine (0.61 mg/kg/day) and clonidine (0.10-0.20 mg/day) | **Treatment response** (as a dichotomous outcome)  Conners’ Parent Rating Scale (CPRS) ≥ 25% reduction from baseline in conduct symptoms (rater: parents)  **All-cause treatment discontinuation** (as a dichotomous outcome)  Yes, reported as study withdrawals  **Tolerability** (as a dichotomous outcome)  Not reported  **Serious adverse events** (as dichotomous outcome)  Not reported  **Specific adverse events** (as dichotomous outcome)  Not reported |
| Rugino et al., 2003^47^ | **Eligibility criteria**  Children were Caucasian aged 5-15 years. The inclusion criteria were: reliable transportation to and from the development center; regular school  attendance; an average Conners Teacher Rating Scale ADHD index *t* score of 70 or higher; an average percentile score for the ADHD Rating Scale IV of 70 or higher; and (5) a verbal intelligence quotient of 80 or higher. Exclusion criteria: acute medical or uncontrolled psychiatric illness; allergy to modafinil or any of the components of the tablet; mitral valve prolapse, left ventricular hypertrophy, cardiac ischemia, clinically significant cardiac arrhythmia, or history of syncope; use of the following medications within 30 days before the study: psychoactive medications other than stimulants prescribed to manage ADHD, antiepileptics, or medications  metabolized primarily through the hepatic cytochrome P450 system; more than three migraine headaches within 3 months before the study; female with potential of becoming pregnant during the study; uncontrolled seizure disorder; sleep disorder with insomnia; and history of manic episodes or psychosis  **Diagnostic criteria**  DSM-IV  **Number randomized**  24  **Age**  5-15 years (range)  **Co-morbidities/Co-medications**  Yes, oppositional disorder or conduct disorders (25%) – Not reported  **ADHD subtypes**  Combined (67%), inattentive (4%) | **Arm 1**  MODAF: modafinil (mean dose: 264 mg/day)  **Arm 2**  Placebo: placebo pill | **Treatment response** (as a dichotomous outcome)  Not reported/data not abstractable  **All-cause treatment discontinuation** (as a dichotomous outcome)  Yes, reported as study withdrawals  **Tolerability** (as a dichotomous outcome)  Yes, reported as study withdrawals due to AEs  **Serious adverse events** (as dichotomous outcome)  None  **Specific adverse events** (as dichotomous outcome)  Loss of appetite/anorexia (as an adverse event)  Sleep disturbances (as an adverse event e.g. “delayed sleep onset”) |
| Abikoff et al., 2004^48-50^ | **Eligibility criteria**  Boys and girls, ages 7.0 to 9.9 years in grades 1 through 4, who met *DSM-III-R* criteria for ADHD; children had to be medication free for at least 2 weeks before evaluation, have normal IQs (WISC-R ≥85), be living with at least one parent, and have telephone access; a positive response to methylphenidate was a study criterion. Exclusions:  diagnosable neurological disorders; psychosis; significant medical illness; current physical or sexual abuse; chronic tic disorder or Tourette’s  disorder; a *DSM-III-R* developmental reading or arithmetic disorder; diagnosable learning disorders were exclusionary (significant learning problems  and poor academic performance were not). Comorbid anxiety and depressive disorders were not exclusionary  **Diagnostic criteria**  DSM-III-R  **Number randomized**  103  **Age**  7-9.9 years (range)  **Co-morbidities/Co-medications**  Yes, oppositional disorder (53%), conduct disorder (30%), anxiety (17%), depression (4%) – Yes (all with stimulants, and 20% prior stimulant exposure)  **ADHD subtypes**  Not reported | **Arm 1**  MPH-SA high dose: methylphenidate (36-38 mg/day)  **Arm 2**  MPH-SA high dose+BT (child and parent training): combination of both methylphenidate (36-38 mg/day) and behavioral therapy including parent and child training. Patients received a multimodal psychosocial treatment. The components included parent training/family therapy, academic organizational skills training, individualized academic assistance, academic remediation (when necessary), social skills training, and individual psychotherapy. The treatment modules were fully manual based before study initiation. Each component was delivered once weekly during the first year (requiring two clinic visits per week) and once monthly during the second year (requiring two clinic visits per month). A 75% attendance rate was required, and make-up sessions were provided. Interventions  focused on parental child-rearing behavior, children’s behavior at home and school, and children’s academic  performance, emotional functioning, and social skills  Interventions were provided by Ph.D. clinical psychologists, and special education teachers with master’s degrees  **Arm 3**  MPH-SA high dose+control: combination of both methylphenidate (36-38 mg/day) and an attention control psychosocial treatment | **Treatment response** (as a dichotomous outcome)  Rate of patients without a diagnosis of ADHD according to DSM-III-R criteria (remission) at 6, 12, 18 and 24 months postbaseline  **All-cause treatment discontinuation** (as a dichotomous outcome)  Yes, reported as withdrawals/drop-outs  **Tolerability** (as a dichotomous outcome)  Not reported  **Serious adverse events** (as dichotomous outcome)  Not reported  **Specific adverse events** (as dichotomous outcome)  Not reported |
| Akhondzadeh et al., 2004^51^ | **Eligibility criteria**  Children 5-11 years with; all patients had combined subtype of ADHD and were newly diagnosed and had not received another stimulant medication prior to enrollment; parents were carefully interviewed and asked to rate the severity of the DSM IV inattention symptoms their children displayed. Exclusions: previous diagnosis of a psychiatric disorder or mental retardation (IQ< 70); clinically significant chronic medical condition,  including a past history of cardiovascular disease,  organic brain disorder, seizures, current abuse or dependence on drugs within 6 months and current treatment with psychotropic medications  **Diagnostic criteria**  DSM-IV  **Number randomized**  44  **Age**  5-11 years (range)  **Co-morbidities/Co-medications**  Not reported – None  **ADHD subtypes**  Combined subtype (100%) | **Arm 1**  MPH-SA low/medium dose: methylphenidate (mean dose: 1 mg/kg/day)  **Arm 2**  MPH-SA low/medium dose+Zinc: combination of methylphenidate (mean dose: 1 mg/kg/day) and zinc sulfate (mean dose: 55 mg/day) | **Treatment response** (as a dichotomous outcome)  Not reported/data not abstractable  **All-cause treatment discontinuation** (as a dichotomous outcome)  Yes, reported as study withdrawals  **Tolerability** (as a dichotomous outcome)  Not reported  **Serious adverse events** (as dichotomous outcome)  None  **Specific adverse events** (as dichotomous outcome)  Loss of appetite/anorexia (as an adverse event)  Sleep disturbances (as an adverse event e.g. “difficulty falling sleep”)  Anxiety (as an adverse event) |
| Bilici et al., 2004^52^ | **Eligibility criteria**  Children with ADHD in outpatient setting. A patient was excluded if he or she had another axis I disorder and had any medical condition. Patients including any comorbid illnesses were excluded.  Other exclusion criteria were that taking any psychotropic and other medication, having intolerance to zinc, having pathological findings in routine biochemical measurements, and taking medication that would affect the study results. Patients with history of clinically significant and  currently relevant hematologic, renal, hepatic, gastrointestinal, endocrine, pulmonary, dermatologic, oncologic, or neurologic (including seizures or epilepsy) disease were excluded. Anyone with a history of chronic hepatitis and elevated liver enzymes as well as those known to be positive rheumatoid factor, was also excluded. Other reasons for exclusion included a history of hypersensitivity to any drug, use of psychostimulants, antipsychotic compounds and antidepressants at any time  **Diagnostic criteria**  DSM-IV  **Number randomized**  400  **Age**  6-14 years (range)  **Co-morbidities/Co-medications**  No – Not reported  **ADHD subtypes**  Not reported | **Arm 1**  Zinc: Zinc sulfate (mean dose: 150 mg/day)  **Arm 2**  Placebo: placebo pill | **Treatment response** (as a dichotomous outcome)  Full therapeutic response defined as an ADHD-RS score decrease ≥ 25% from baseline (unpublished outcome data provided by Prof. Bilici, correspondence of January 9^th^ 2016)  **All-cause treatment discontinuation** (as a dichotomous outcome)  Yes, reported as study discontinuation  **Tolerability** (as a dichotomous outcome)  Yes, reported as study discontinuation due to AEs  **Serious adverse events** (as dichotomous outcome)  Not reported  **Specific adverse events** (as dichotomous outcome)  Not reported |
| Döpfner et al., 2004^53^ | **Eligibility criteria**  Children aged 6-10 years; attendance at school between the first and fourth grade; nonverbal IQ of 80 or higher; ADHD diagnosis according to DSM-III-R/ICD-10-PRDC  **Diagnostic criteria**  DSM-III-R  **Number randomized**  73  **Age**  6-10 years (range)  **Co-morbidities/Co-medications**  Yes, oppositional disorder or conduct disorder (61%), learning problems (5%), tics (3%), dysthymia (3%) – Not reported  **ADHD subtypes**  Not reported | **Arm 1**  MPH-SA medium/low dose+BT (child, parent and teacher training): combination of both methylphenidate (0.3-0.8 mg/kg/day) and BT with child, parent and teacher training (as described below)  **Arm 2**  BT (child, parent and teacher training): Behavioral therapy included parent training, child-focused treatment, and school-based intervention. BT was individually tailored according to a treatment  manual which integrates family based and school  based interventions with cognitive behaviour therapy of the child. The family interventions are based on the parent training manuals of Forehand, McMahon [26] and Barkley; the school interventions are based on manuals of DuPaul and Stoner and Swanson. Self  instructional procedures are based on Camp and Bash. Basic aims and principles for interventions in the  family and school were (1) the reduction of specific  problem behaviour in specific family or school situations as defined by the Individual Problem Checklist; (2) the enhancement attending skills of parents and teachers; in addition for parents during supervised playtime sessions; (3) development of effective methods of communicating commands and setting rules in specific problem situations (parents and teachers); (4) token economies, response cost for systems and daily home report cards in order to reinforce appropriate behavior and to reduce problematic behaviour in specific situations (parents and teachers); (5) development of appropriate negative consequences for problem behavior and use of time out in order to reduce problem behaviour  in specific situations (parents and teachers). In this  therapeutic process the child was integrated as an active member. Self instructional training was added as indicated. The child was taught steps of self instruction which were trained with different materials especially school-related materials. Training generalization was enhanced by using self monitoring and self evaluation of whether procedures have been followed. Parents and teachers were taught to help the child to apply self-instruction procedures at home or at school | **Treatment response** (as a dichotomous outcome)  For comparison purposes with MTA trial, the parent and teacher ratings of ADHD and ODD/CD were combined for a global outcome measure by averaging the parent and teacher ratings in terms of “success rates” (reponders)  **All-cause treatment discontinuation** (as a dichotomous outcome)  Yes, reported as drop-outs/terminated early  **Tolerability** (as a dichotomous outcome)  Not reported  **Serious adverse events** (as dichotomous outcome)  Not reported  **Specific adverse events** (as dichotomous outcome)  Not reported |
| Kaplan et al., 2004^54^ | **Eligibility criteria**  Patients in this subset analysis were 7 to 13 years of age at their initial visit and met diagnostic criteria for ADHD and also met criteria for ODD, as  characterized by the computerized Diagnostic Interview for Children and Adolescents-IV (DICA-IV) completed by the parent and confirmed by clinical assessment. As a participation requirement, patients scored at least 1.5 standard deviations above the age and gender norms for their ADHD diagnostic subtype on the  ADHD-RS. All children had an IQ in the normal  range, as measured by four subtests of the WISC–III. Patients were excluded if they had significant prior or current medical conditions, psychosis, seizure disorder, history of alcohol or drug abuse within the past 3 months or positive screening for abuse of drugs, or were identified as poor metabolizers of the cytochrome P4502D6 isoenzyme  **Diagnostic criteria**  DSM-IV  **Number randomized**  98  **Age**  7-13 years (range)  **Co-morbidities/Co-medications**  Yes, oppositional disorder (100%) – Not reported  **ADHD subtypes**  Not reported | **Arm 1**  ATX: atomoxetine total dose 55.3 mg/day (1.6 mg/kg/day)  **Arm 2**  Placebo: placebo pill | **Treatment response** (as a dichotomous outcome)  Responders based on the achievement og a 25% or greater decrease from baseline to endpoint in the ADHD-RS-IV-Parent:Inv total score (rater: parent)  **All-cause treatment discontinuation** (as a dichotomous outcome)  Yes, reported as study discontinuations  **Tolerability** (as a dichotomous outcome)  Yes, reported as study discontinuations due to AEs  **Serious adverse events** (as dichotomous outcome)  Not reported  **Specific adverse events** (as dichotomous outcome)  Loss of appetite/anorexia (as an adverse event) |
| Kelsey et al., 2004^55,56^ | **Eligibility criteria**  Children 6 to 12 years of age; symptom severity score at least 1.5 SDs above age and gender normative values, as assessed with the ADHD-RS  for the total score or either of the inattentive or hyperactive/impulsive subscales. Important exclusion criteria included: serious medical illness, a history of psychosis or bipolar disorder, alcohol or drug abuse within the past 3 months, and ongoing use of psychoactive medications other than the study drug  **Diagnostic criteria**  DSM-IV  **Number randomized**  197  **Age**  6-12 years (range)  **Co-morbidities/Co-medications**  Yes, oppositional disorder (35%) and conduct disorder (4%)– No  **ADHD subtypes**  Combined (69%), inattentive (27%), hyperactive/impulsive (4%) | **Arm 1**  ATX: atomoxetine total dose 44.5 mg/day (1.3 mg/kg/day)  **Arm 2**  Placebo: placebo pill | **Treatment response** (as a dichotomous outcome)  Responders based on the achievement of a 25% or greater decrease from baseline to endpoint in the ADHD-RS-IV total score (rater: clinician) or an endpoint CGI-I score of 1 or 2 (rater: clinician)  **All-cause treatment discontinuation** (as a dichotomous outcome)  Yes, reported as study withdrawals  **Tolerability** (as a dichotomous outcome)  Yes, reported as study withdrawals due to AEs  **Serious adverse events** (as dichotomous outcome)  None  **Specific adverse events** (as dichotomous outcome)  Loss of appetite/anorexia (as an adverse event)  Syncope (as an adverse event) |
| Michelson et al., 2004^57,58^ | **Eligibility criteria**  Children aged 6 to 15 years; symptom severity was at least 1.5 SD above US age and gender norms. Exclusions: patients with bipolar disorder or a psychotic illness; patients with unstable medical illness or patients with a condition that would require ongoing administration of a psychoactive medication (other than ATX)  **Diagnostic criteria**  DSM-IV  **Number randomized**  416  **Age**  6-15 years (range)  **Co-morbidities/Co-medications**  Yes, oppositional disorder (35%) and depression (2%)– Not reported  **ADHD subtypes**  Combined (73%), inattentive (27%), hyperactive/impulsive (5%) | **Arm 1**  ATX: atomoxetine 1.6 mg/kg/day  **Arm 2**  Placebo: placebo pill | **Treatment response** (as a dichotomous outcome)  Not reported/data not abstractable  **All-cause treatment discontinuation** (as a dichotomous outcome)  Yes, reported as study discontinuations  **Tolerability** (as a dichotomous outcome)  Yes, reported as study discontinuations due to AEs  **Serious adverse events** (as dichotomous outcome)  Yes, reported  **Specific adverse events** (as dichotomous outcome)  Not reported |
| Wigal et al., 2004^59^ | **Eligibility criteria**  Children 6-17 years of age enrolled in elementary school; within 30% of normal body weight; anticipated as available for the entire length of the study; female participants were required to be pre-menarche. Exclusions were: history or evidence of cardiovascular, renal, respiratory (other than asthma/allergy), endocrine or immune system disease; history of substance abuse; hypersensitivity to dex,l-methylphenidate or other stimulants  **Diagnostic criteria**  DSM-IV  **Number randomized**  132  **Age**  6-17 years (range)  **Co-morbidities/Co-medications**  None – No  **ADHD subtypes**  Combined (64%), inattentive (35%), hyperactive/impulsive (1%) | **Arm 1**  MPH-SA low/medium dose: methylphenidate (d-isomer, dexmethylphenidate) 18.3 mg/day  **Arm 2**  MPH-SA high dose: methylpenidate (racemic mixture of both d- and l- enantiomers, d-l-methylphenidate) 32.1 mg/day  **Arm 3**  Placebo: placebo pill | **Treatment response** (as a dichotomous outcome)  A therapeutic response was defined by a rating of “much improved” or “very much improved” on the CGI-I (reported by clinician)  **All-cause treatment discontinuation** (as a dichotomous outcome)  Yes, reported as study discontinuation  **Tolerability** (as a dichotomous outcome)  Yes, reported as study discontinuatios due to AES  **Serious adverse events** (as dichotomous outcome)  None  **Specific adverse events** (as dichotomous outcome)  Loss of appetite/anorexia (as an adverse event)  Decreased weight (as anadverse event of clinically significant weight loss e.g. ranging from 5% to 18% of baseline values)  Anxiety (adas an averse event leading to discontinuation e.g. symptoms of anxiety)  Cardiovascular event – Increased heart rate (as an adverse event e.g. mildly increased heart rate)  Decreased blood pressure (as an adverse event e.g. ranges SBP from 20 to 49 mm Hg; DBP from 16 to 22 mm Hg)  Increased pulse values (as an adverse event e.g. range from 15 to 44 beats per minute above baseline) |
| Allen et al., 2005^60,61^ | **Eligibility criteria**  Children or adolescents at least 7 years of age but less than 17 years and 6 months and weighing between 20 and 80 kg; concurrent Tourette syndrome or chronic motor tic disorder, as diagnosed by clinical interview and examination by the investigator and confirmed by the K-SADSPL;  Subjects’ scores on the ADHDRS-IV-Parent:Inv had to be at least 1.5 standard deviations above the age and sex norm for diagnostic subtype (predominantly inattentive or predominantly hyperactive–impulsive), or for the total score for the combined subtype; subjects’ Yale Global Tic Severity Scale (YGTSS) total scores had to be at least 5 at both Visits 1 and 2. Exclusion criteria included: a Children’s Yale–Brown Obsessive–  Compulsive Scale19 (C-YBOCS) total score >15 or diagnosis of obsessive-compulsive disorder severe enough, in the investigator’s opinion, to require pharmacotherapy; a Children’s Depression  Rating Scale–Revised (CDRS-R) total score >40 or diagnosis of depression severe enough to require pharmacotherapy; a history of bipolar disorder or psychosis; seizure disorder; or current use of any psychotropic medication other than study drug  **Diagnostic criteria**  DSM-IV  **Number randomized**  148  **Age**  7-17 years (range)  **Co-morbidities/Co-medications**  Yes, all with tic disorders (Tourette syndrome: 79%; chronic motor tics: 30%); oppositional disorders (22%), anxiety (3%), obsessive-compulsive disorder (3%), depression (1%) – No  **ADHD subtypes**  Combined (61%), inattentive (36%), hyperactive/impulsive (3%) | **Arm 1**  ATX: atomoxetine 1.3 mg/kg/day  **Arm 2**  Placebo: placebo pill | **Treatment response** (as a dichotomous outcome)  Not reported/data not abstractable  **All-cause treatment discontinuation** (as a dichotomous outcome)  Yes, reported as study discontinuation  **Tolerability** (as a dichotomous outcome)  Yes, reported as study discontinuatios due to AEs  **Serious adverse events** (as dichotomous outcome)  Yes, reported  **Specific adverse events** (as dichotomous outcome)  Loss of appetite/anorexia (as an adverse event)  Decreased weight (significant weight loss e.g. ≥ 3.5% of body weight loss)  Insomnia (as an adverse event)  Cardiovascular event – Increased heart rate (e.g. ≥ 25 bpm to value of at least 110 bpm) |
| Biederman et al., 2005^62^ | **Eligibility criteria**  Children 6 to 17 years of age and had a diagnosis of ADHD with a Clinical Global Impression Severity of Illness (CGI-S) rating of 4 or higher (“moderately ill” or worse). In addition, patients were attending full-time school (ie, they were not being homeschooled); had a teacher-/investigator-rated  ADHD-RS-IV School Version total and/or subscale score at least 1.5 SDs above normal values for age and gender, were between the 5th and 95^th^ percentile for weight and height on the basis of National Center for Health Statistics guidelines, had an IQ of at least 80 as estimated by the Wechsler Intelligence Scale for Children–Third Edition, and had a score of at least 80 on the Wechsler Individual Achievement Test–Second Edition–Abbreviated. Patients were excluded when they had a history or current diagnosis of pervasive developmental disorder, schizophrenia, or other psychotic disorders (DSM-IV Axis I); evidence of suicide risk; current psychiatric comorbidity that required pharmacotherapy; or other active clinically significant disease. Patients whose ADHD was well controlled and who were satisfied with current ADHD therapy were also excluded, as were those who had failed to respond to 2 or more adequate courses (dose and duration) of stimulant therapy for ADHD. Other exclusion criteria  included a clinically significant drug sensitivity to stimulants, a history of alcohol or substance abuse as defined by DSM-IV criteria, 21 consumption of >250 mg/day caffeine, absolute neutrophil  count <1*10^9^/L, hypertension, and resting heart rate outside the range of 60 to 115 beats per minute. Concomitant use of prescription or nonprescription agents with psychotropic properties, including ADHD treatments and dietary supplements, was prohibited within 1 week of the baseline visit (within 2 weeks for monoamine oxidase inhibitors and selective serotonin reuptake inhibitors) and during the study  **Diagnostic criteria**  DSM-IV  **Number randomized**  248  **Age**  6-17 years (range)  **Co-morbidities/Co-medications**  None – Not reporte  **ADHD subtypes**  Combined (59%), inattentive (38%), hyperactive/impulsive (3%) | **Arm 1**  MODAF: modafinil 170-425 mg/day (mean dose: 368.5 mg/day)  **Arm 2**  Placebo: placebo pill | **Treatment response** (as a dichotomous outcome)  A therapeutic response was defined by a rating of “much improved” or “very much improved” on the CGI-I (reported by clinician)  **All-cause treatment discontinuation** (as a dichotomous outcome)  Yes, reported as study discontinuation  **Tolerability** (as a dichotomous outcome)  Yes, reported as study discontinuatios due to AEs  **Serious adverse events** (as dichotomous outcome)  Yes, reported  **Specific adverse events** (as dichotomous outcome)  Loss of appetite/anorexia (as an adverse event)  Insomnia (as an adverse event)  Cardiovascular event – Tachycardia (adverse event leading to study discontinuation) |
| Jacobs et al., 2005^63^ | **Eligibility criteria**  Children aged 6 to 12 years taking stimulant medication if their dosage had been stable for 6 months prior to enrollment. Exclusions included: comorbid medical or psychological conditions that influenced behavior or the ability to complete the study protocol or required the use of medications thought to interfere with homeopathic treatment (such as corticosteroids); home-schoold children  **Diagnostic criteria**  DSM-IV  **Number randomized**  43  **Age**  6-12 years (range)  **Co-morbidities/Co-medications**  None – Yes (21% taking stimulants)  **ADHD subtypes**  Not reported | **Arm 1**  HOMEO: homeopathy including 41 different remedies such as *Medorrhinum*, *Saccharum officinalis*, *Calcarea carbonica*, *Calcarea phosphorica*, *China officinalis* and *Stramonium*. The homeopathic medicines were prepared by impregnating pellets made of 85% sucrose and 15% lactose with a liquid homeopathic dilutions  **Arm 2**  Placebo: placebo pill | **Treatment response** (as a dichotomous outcome)  Not reported/data not abstractable  **All-cause treatment discontinuation** (as a dichotomous outcome)  Yes, reported as study drop-outs/lost to follow-up  **Tolerability** (as a dichotomous outcome)  Not reported  **Serious adverse events** (as dichotomous outcome)  Not reported  **Specific adverse events** (as dichotomous outcome)  Not reported |
| Kemner et al., 2005^64^ | **Eligibility criteria**  Children 6-12 with an investigator-rated ADHD-RS score of at least 24 and a Clinical Global  Impression–Severity of Illness scale (CGI-S) score of at least 4 (“moderately ill” or worse). The study population included both newly diagnosed patients who had not previously received ADHD medication treatment (ie, treatment naïve) and patients who were previously taking some type of ADHD medication but may not have been receiving optimal treatment, as judged by the clinician in conjunction with the parents. Exclusion criteria included: eating disorders; substance use disorders; comorbid psychiatric conditions other than oppositional defiant disorder; history of seizure, tic disorder, mental retardation, or severe developmental disorder; personal or family history of Tourette’s syndrome; previous diagnosis of hyperthyroidism or glaucoma; use of medications contraindicated for coadministration with OROS MPH or ATX; known nonresponse to treatments indicated for ADHD; and occurrence of menarche in girls  **Diagnostic criteria**  DSM-IV-TR  **Number randomized**  1323  **Age**  6-12 years (range)  **Co-morbidities/Co-medications**  None – Yes (57% previous medications for ADHD)  **ADHD subtypes**  Combined (72%), inattentive (15%), hyperactive/impulsive (13%) | **Arm 1**  MPH-LA low/medium dose: methylphenidate long-acting 32.7 mg/day (1 mg/kg/day)  **Arm 2**  ATX: atomoxetine 36.7 mg/day (1 mg/kg/day) | **Treatment response** (as a dichotomous outcome)  ADHD-RS ≥ 30% reduction from baseline in total score (rater: clinician); Clinical Global Impression Improvement (CGI-I) scale (score 1 or 2, much or very much improved reported by the clinician/investigator)  **All-cause treatment discontinuation** (as a dichotomous outcome)  Not reported  **Tolerability** (as a dichotomous outcome)  Yes, reported as study discontinuatios due to AEs  **Serious adverse events** (as dichotomous outcome)  Yes, reported  **Specific adverse events** (as dichotomous outcome)  Loss of appetite/anorexia (as an adverse event)  Insomnia (as an adverse event) |
| Jacobs et al., 2005^63^ | **Eligibility criteria**  Children 7-12 years, nonmedicated, diagnosis of ADHD of either combined or predominantly inattentive subtype; access to a personal computer with an Internet connection at home or in school. Exclusion criteria were: being treated with stimulants, atomoxetine, neuroleptic, or any other psychoactive drugs; fulfilling criteria for diagnosis of clinically significant oppositional defiant disorder, autistic syndrome, Asperger’s syndrome or depression; history of seizures during the past 2 years; IQ <80 (based on an IQ test or the physician’s clinical impression and school history); motor or perceptual handicap that would prevent using the computer program; educational level and socioeconomic situation that made it unlikely that the family would be able to follow the treatment procedure and study requirements (the educational level of the parents was not specified in terms of academic degree); and medical illness  requiring immediate treatment  **Diagnostic criteria**  DSM-IV  **Number randomized**  53  **Age**  7-12 years (range)  **Co-morbidities/Co-medications**  None – No  **ADHD subtypes**  Combined (72%), inattentive (28%) | **Arm 1**  WM training: Cognitive training – working memory training. The treatment consisted of performing WM tasks implemented in a computer program developed for this study (RoboMemo, Cogmed Cognitive Medical Systems AB, Stockholm, Sweden). The program was provided on a CD and used by the child on a personal  computer either at home or in school. The program included visuospatial WM tasks (remembering the position of objects in a 4 3 4 grid as well as verbal tasks (remembering phonemes, letters, or digits). Responses were made by clicking on displays with the computer  mouse. The children performed 90 WM trials on each day of training. Total time depended on the level and time between trials. Medium total training time (excluding breaks) was about 40 minutes. The difficulty level was automatically adjusted, on a trial by-trial basis, to match the WM span of the child on each task  **Arm 2**  Control: easy training. The comparison condition was identical to the treatment except that the difficulty of the 90 WM trials remained on the initial low level (two to three items) instead of being increased to match the  WM span of the child | **Treatment response** (as a dichotomous outcome)  Not reported/data not abstractable  **All-cause treatment discontinuation** (as a dichotomous outcome)  Yes, reported as study withdrawals and/or noncompliance  **Tolerability** (as a dichotomous outcome)  Not reported  **Serious adverse events** (as dichotomous outcome)  Not reported  **Specific adverse events** (as dichotomous outcome)  Not reported |
| So, 2005^66,67^ | **Eligibility criteria**  Children between 7 and 9.9 years; ethnic Chinese;  studying in Grades 1–4; living with parents, one of them being the main caretaker; a diagnosis of ADHD (combined type); normal intelligence (IQ above 80); no significant physical disability; no past exposure to stimulant medication (MPH) for more than 2 weeks; willingness to accept random allocation into either one treatment  condition (MPH-only or combined MPH+BT); and parents having no intellectual impairment or  current psychosis  **Diagnostic criteria**  DSM-IV  **Number randomized**  579  **Age**  7-9.9 years (range)  **Co-morbidities/Co-medications**  Yes, oppositional disorder (50%), conduct disorder (6%), anxiety disorders (29%), depression (6%), learning disabilities (19%)/No (naïve)  **ADHD subtypes**  All children met criteria for ADHD Combined Type | **Arm 1**  MPH-SA low/medium dose: methylphenidate 14.7 mg/day (mean dose)  **Arm 2**  MPH-SA low/medium dose+BT (child and parent training): combination of both methylphenidate (13.7mg/day) and behavioral therapy with child and parent training (as described below). BT consisted of 24 weekly sessions for 6 months in a group format. The programme contained three components: (1) direct contingency management in the laboratory classroom; (2) skills training; and (3) parent training. The first two components were conducted by paraprofessional trainers (psychiatric nurses, clinic teachers, and occupational therapists) and supervised by a clinical psychologist. Each child training session lasted for about 100 min. Therewas one trainer in a group of 8–9 ADHD children and 2–3 assistants to implement the behavioural intervention. Parent training was conducted by the first author herself. Each parent training session lasted for about 90 min.  In the laboratory classroom, a system of token economy was established, based on a continuous schedule of social reinforcement paired with continuous application of response cost to the following rules which were prominently displayed in the classroom: (1) following directions; (2) working quietly; (3) raising hand to speak or ask for help; (4) remaining in the assigned seat or area; (5) showing good sportsmanship; and (6) helping or sharing  with a peer. Children started each group session with 180 tokens. They were magnetic stickers adhering onto a board, providing children with visual aids to keep track of their progress. A tokenwas removed contingent upon each occurrence of behaviour that violated the rules. Concurrently, individualized target behaviours (e.g. completing assignments at 80% accuracy, ignoring provocations, recognizing and dealing with feelings) were identified for each child. Trainers regularly reviewed token totals with the children to keep them apprised of the accumulative consequences of their behaviour and gave them liberal praise and reminders on the behaviours to be improved. At later sessions, children performed their own self-reviews in order to practise self-monitoring and self management in finding solutions to their problematic behaviours. Visual cues of the rules and the tokens retained were also removed. The initial token system was replaced by a ‘‘Match Game’’ (Hinshaw, Henker, & Whalen, 1984), in which at  the beginning of the session, each ADHD child estimated how well he/she could perform. Extra tokens would be given for an accurate self-evaluation (e.g. rating matching to that of the trainer), regardless  of the actual eventual rating | **Treatment response** (as a dichotomous outcome)  Response defined by a symptom composite score across 3 domains (inattention, hyperactivity/impulsivity and defiance) and two sources (parents and teachers) on the SWAN (Strenghts and Weaknesses of ADHD symptoms and Normal behaviors) rating scale, which is equivalent to SNAP. In particular, the numer of patients within normal functioning range of SWAN rating scale where considered responders in our analyses.  **All-cause treatment discontinuation** (as a dichotomous outcome)  Yes, reported as withdrawals/drop-outs (“attrition rates”)  **Tolerability** (as a dichotomous outcome)  Not reported  **Serious adverse events** (as dichotomous outcome)  Not reported  **Specific adverse events** (as dichotomous outcome)  Not reported |
| Starr et al., 2005^68^ | **Eligibility criteria**  Children 6-12 with an investigator-rated ADHD-RS score of at least 24 and a Clinical Global  Impression–Severity of Illness scale (CGI-S) score of at least 4 (“moderately ill” or worse). The study population included both newly diagnosed patients who had not previously received ADHD medication treatment (ie, treatment naïve) and patients who were previously taking some type of ADHD medication but may not have been receiving optimal treatment, as judged by the clinician in conjunction with the parents. Exclusion criteria included: eating disorders; substance use disorders; comorbid psychiatric conditions other than oppositional defiant disorder; history of seizure, tic disorder, mental retardation, or severe developmental disorder; personal or family history of Tourette’s syndrome; previous diagnosis of hyperthyroidism or glaucoma; use of medications contraindicated for coadministration with OROS MPH or ATX; known nonresponse to treatments indicated for ADHD; and occurrence of menarche in girls. This sub-analysis reports methods and results in African-American children with ADHD  **Diagnostic criteria**  DSM-IV-TR  **Number randomized**  183  **Age**  6-12 years (range)  **Co-morbidities/Co-medications**  None – No  **ADHD subtypes**  Not reported | **Arm 1**  MPH-LA low/medium dose: methylphenidate long-acting 32.8 mg/day (1 mg/kg/day)  **Arm 2**  ATX: atomoxetine 1.1 mg/kg/day | **Treatment response** (as a dichotomous outcome)  ADHD-RS ≥ 30% reduction from baseline in total score (rater: clinician) ; Clinical Global Impression Improvement (CGI-I) scale (score 1 or 2, much or very much improved reported by the clinician/investigator)  **All-cause treatment discontinuation** (as a dichotomous outcome)  Not reported  **Tolerability** (as a dichotomous outcome)  Yes, reported as study discontinuatios due to AEs  **Serious adverse events** (as dichotomous outcome)  Yes, reported  **Specific adverse events** (as dichotomous outcome)  Loss of appetite/anorexia (as an adverse event)  Insomnia (as an adverse event) |
| Weiss et al., 2005^69-71^ | **Eligibility criteria**  Children 8 to 12 years of age with ADHD (any subtype); symptom severity had to be at  least 1.0 SD above age and sex norms on the ADHD Rating Scale-IV-Teacher Version; a mean Conners  Parent Rating Scale (CPRS-R:S) ADHD Index score at least 1.5 SDs above age and sex norms. Important exclusion criteria included: unavailability of a primary teacher willing to keep telephone appointments and to provide ratings  and reports as part of the study, evidence of a significant intellectual deficit, serious medical illness, or use of other psychotropic medication. Children with concurrent learning disorders were  included  **Diagnostic criteria**  DSM-IV  **Number randomized**  153  **Age**  8-12 years (range)  **Co-morbidities/Co-medications**  Yes, oppositional disorder (33%), anxiety (3%), learning disabilities (30%), motor skills disorder (7%) and communications disorder (8%) – Yes, 60% prior stimulant exposure  **ADHD subtypes**  Combined (73%), inattentive (27%), hyperactive/impulsive (1%) | **Arm 1**  ATX: atomoxetine total dose 1.3 mg/kg/day  **Arm 2**  Placebo: placebo pill | **Treatment response** (as a dichotomous outcome)  Responders based on the achievement of a 20% or greater decrease from baseline to endpoint in the ADHD-RS-IV-Teacher:Inv total score (rater: teacher) or an endpoint CGI-S score of 1 or 2 (no symptomatology)  **All-cause treatment discontinuation** (as a dichotomous outcome)  Yes, reported as study withdrawals  **Tolerability** (as a dichotomous outcome)  Yes, reported as study withdrawals due to AEs  **Serious adverse events** (as dichotomous outcome)  None  **Specific adverse events** (as dichotomous outcome)  Loss of appetite/anorexia (as an adverse event)  Decreased weight (as an adverse event e.g. at least 3.5% reduction)  Insomnia (as an adverse event)  Anxiety (as an adverse event)  Cardiovascular event – Palpitations (as an adverse event) |
| Wigal et al., 2005^72^ | **Eligibility criteria**  Children 6-12 years with ADHD combined  subtype or predominantly hyperactive/impulsive  subtype; weight between 40 lb (18.18 kg) and 120 lb (54.54 kg) at enrollment; and capable of understanding and following classroom instruction and generally functioning academically at age-appropriate levels. Subjects were excluded if any of the following criteria were met: (a) *DSM-IV-TR* diagnosis of ADHD, predominantly inattentive subtype; current controlled or uncontrolled comorbid psychiatric diagnosis (except oppositional defiant disorder) with significant symptoms such as pervasive developmental disorder, post-traumatic stress disorder, psychosis, bipolar illness, severe obsessive-compulsive disorder, severe depression, or severe anxiety disorder; documented history of aggressive behavior serious enough to preclude participation in regular classroom activities, or a *DSM-IV-TR* diagnosis of conduct disorder; documented allergies, adverse reactions, or intolerance of stimulants, including MAS XR, atomoxetine, or tricyclic antidepressants, or a history of failure to respond clinically to adequate doses of these medications; history of suspected substance abuse or drug abuse (excluding nicotine) or living with someone with such history or suspicion; taking any prohibited medication including antidepressants, antipsychotics, neuroleptics, anxiolytics, and anticonvulsants; and history of seizure during  the past 2 years, a tic disorder, or a family history of Tourette’s disorder  **Diagnostic criteria**  DSM-IV-TR  **Number randomized**  215  **Age**  6-12 (range)  **Co-morbidities/Co-medications**  Not reported  **ADHD subtypes**  Combined (99%), hyperactive/impulsive (1%) | **Arm 1**  MIX-AMPH-LA: mixed amphetamine salts long acting (Adderall XR) 10, 20 or 30 mg/day  **Arm 2**  ATX: atomoxetine total dose 1.2 mg/kg/day | **Treatment response** (as a dichotomous outcome)  Global assessment of overall improvement by the investigator (the Clinical Global Impressions - Improvement, GGI-I scale) and SKAMP rating scale with at least 25% improvement (rater: teacher)  **All-cause treatment discontinuation** (as a dichotomous outcome)  Yes, reported as study discontinuations  **Tolerability** (as a dichotomous outcome)  Yes, reported as study discontibuations due to AEs  **Serious adverse events** (as dichotomous outcome)  Yes, reported  **Specific adverse events** (as dichotomous outcome)  Loss of appetite/anorexia (as an adverse event)  Decreased weight (as an adverse event)  Insomnia (as an adverse event) |
| Biederman et al., 2006^73^ | **Eligibility criteria**  Children 6 to 13 years whose height and weight corresponded to greater than the 5^th^ percentile in standardized growth charts and who were attending ful-day kidenrgarten, elementary school, or middle school; children who were stimulant-naïve or who had manifested an unsatisfactory response to stimulant therapy; IQ of at least 80 and a score of 80 or higher on the screener version of th WIAT were used to rule out low IQ or learning disabilities; CGI-S score of 4 or more (moderately ill or worse). Main exclusion criteria: clinically significant gastrointestinal, cardiovascular, renal, hematologic, neoplastic, endocrine, neurologic, immunodeficiency, pulmonary, or other major clinically significant disorder or disease; any current psychiatric comorbidity, including but not limited to depression or other mood disorder, anxiety disorder, or pervasive mental disorder that required pharmacotherapy; use of any prescription (e.g. clonidine, guanfacine) or nonprescription medication with psychoactive properties within 1 week of the start of washout period; and a history or evidence of substance abuse  **Diagnostic criteria**  DSM-IV  **Number randomized**  248  **Age**  6-13 years (range)  **Co-morbidities/Co-medications**  None – No  **ADHD subtypes**  Combined (77%), inattentive (21%), hyperactive/impulsive (2%) | **Arm 1**  MODAF: modafinil 300-400 mg/day  **Arm 2**  Placebo: placebo pill | **Treatment response** (as a dichotomous outcome)  A therapeutic response was defined by a rating of “much improved” or “very much improved” on the CGI-I (reported by clinician)  **All-cause treatment discontinuation** (as a dichotomous outcome)  Yes, reported as study discontinuations  **Tolerability** (as a dichotomous outcome)  Yes, reported as study discontinuatios due to AEs  **Serious adverse events** (as dichotomous outcome)  Yes, reported  **Specific adverse events** (as dichotomous outcome)  Loss of appetite/anorexia (as an adverse event)  Insomnia (as an adverse event) |
| Findling et al., 2006^74^ | **Eligibility criteria**  Children aged 6–12 years (inclusive); on a stable dose of methylphenidate ‡3 weeks prior to screening; diagnosed with ADHD based on DSM-IV criteria for any subtype; attending a school setting in which a single teacher could make morning and afternoon assessments of the child’s behavior. Exclusion criteria were: female who had experienced menarche; co-morbid psychiatric disorder requiring medication; history of seizure, tic disorder, or a family history of Tourette’s disorder; IQ test score below 80, or functioning at a level of intelligence indicative of an IQ below 80; the use of unapproved medication(s); use of an investigational product within 30 days prior to study entry; concurrent chronic or acute illness, disability, or medication, that might confound the results of rating tests; diagnosed with hyperthyroidism, glaucoma, or eating disorder; current substance abuse disorder or living with someone with a current substance abuse disorder; demonstrated lack of response to MPH  **Diagnostic criteria**  DSM-IV  **Number randomized**  318  **Age**  6-12 years (range)  **Co-morbidities/Co-medications**  None/Not reported  **ADHD subtypes**  Combined (71%), inattentive (23%), hyperactive/impulsive (6%) | **Arm 1**  MPH-SA any dose: methylphenidate short-acting (inmediate release) 20, 40 or 60 mg/day  **Arm 2**  MPH-INT any dose: methylphenidate modified-release 20, 40 or 60 mg/day  **Arm 3**  Placebo: placebo pill | **Treatment response** (as a dichotomous outcome)  Clinician-rated Clinical Global Impression Improvement (CGI-I) scale – e.g. responder considered very much improved or much improved; Parent’s Global Assessment (PGA) scale  **All-cause treatment discontinuation** (as a dichotomous outcome)  Not reported  **Tolerability** (as a dichotomous outcome)  Yes, reported as withdrawals due to AEs  **Serious adverse events** (as dichotomous outcome)  Yes, reported  **Specific adverse events** (as dichotomous outcome)  Loss of appetite/anorexia (as an adverse event)  Insomnia (as an adverse event) |
| Gau et al., 2006^75^ | **Eligibility criteria**  Children aged 6–15 years, with a clinical diagnosis  of any subtype of ADHD taking MPH on a total daily dose of MPH of 10 mg but not more than 40 mg for past 3 months. Patients were excluded  from participation if they had significant gastrointestinal problems, a history of hypertension, known hypersensitivity to MPH, or a co-existing medical condition or concurrent medication (such as monoamine oxidase inhibitors, and medicines used to treat depression, prevent seizure, or prevent blood clots) likely to interfere with the safe administration of MPH. Patients with glaucoma, Tourette’s syndrome, an active seizure disorder, or a psychotic disorder were excluded, as were girls who had reached menarche  **Diagnostic criteria**  DSM-IV  **Number randomized**  64  **Age**  6-15 years (range)  **Co-morbidities/Co-medications**  None/Not reported  **ADHD subtypes**  Combined (78%), inattentive (19%), hyperactive/impulsive (3%) | **Arm 1**  MPH-SA low/medium dose: methylphenidate short-acting (inmediate release) 26.7 mg/day  **Arm 2**  MPH-LA low/medium dose: methylphenidate long-acting (OROS formulation) 27.7 mg/day | **Treatment response** (as a dichotomous outcome)  Clinician-rated Clinical Global Impression Improvement (CGI-I) scale – e.g. responder considered very much improved or much improved  **All-cause treatment discontinuation** (as a dichotomous outcome)  None  **Tolerability** (as a dichotomous outcome)  None  **Serious adverse events** (as dichotomous outcome)  Not reported  **Specific adverse events** (as dichotomous outcome)  Loss of appetite/anorexia (as an adverse event)  Insomnia (as an adverse event)  Anxiety (as an adverse event e.g. ‘anxious’) |
| Greenhill et al., 2006^76^ | **Eligibility criteria**  Children aged 6–17 years; Conners’ ADHD/DSM-IV Scales - Teacher, for boys 6 to 8 years ≥ 27, 9 to 11  years ≥ 24, 12 to 14 years ≥ 19, 15 to 17 years ≥ 14; Conners’ ADHD/DSM-IV Scales - Teacher, for girls 6 to 8 years ≥ 16, 9 to 11 years ≥ 13, 12 to 14 years ≥ 12; 15 to 17 years ≥ 6; age-appropriate functioning levels academically; negative pregnancy test and adequate contraception. Exclusion criteria were: clinically significant abnormalities in vital signs, physical examination findings or laboratory test results history of seizures or use of anticonvulsant medication; comorbid psychiatric conditions (obtained by clinical interview); any medical condition that could interfere with study participation or assessments, or that may pose danger with administration of MPH; psychotropic medications; initiation of psychotherapy within the past 3 months; positive urine drug screen; history of poor response or intolerance to MPH; pregnant or nursing; any other investigational drug within 30 days of study entry  **Diagnostic criteria**  DSM-IV  **Number randomized**  103  **Age**  6-17 years (range)  **Co-morbidities/Co-medications**  None/Not reported (39% prior MPH exposure)  **ADHD subtypes**  Combined (77%), inattentive (21%), hyperactive/impulsive (2%) | **Arm 1**  MPH-LA low/medium dose: dexmethylphenidate long-acting (extended-release) 24.0 mg/day  **Arm 2**  Placebo: placebo pill | **Treatment response** (as a dichotomous outcome)  Clinician-rated Clinical Global Impression Improvement (CGI-I) scale – e.g. responder considered very much improved or much improved; Clinician-rated Clinical Global Impression Severy (CGI-S) scale – e.g. responder with ratings of 1 (not at all ill), 2 (borderline ill) or 3 (mildly ill)  **All-cause treatment discontinuation** (as a dichotomous outcome)  Yes, reported as study discontinuations  **Tolerability** (as a dichotomous outcome)  Yes, reported as study discontinuations due to AEs  **Serious adverse events** (as dichotomous outcome)  None  **Specific adverse events** (as dichotomous outcome)  Loss of appetite/anorexia (as an adverse event)  Decreased weight (as an adverse event e.g. ≥ 7% decreases from baseline)  Insomnia (as an adverse event e.g. insomnia and initial insomnia)  Cardiovascular event – blood pressure increased (e.g. notable rise in SBP and/or DBP)  Heart rate decreased (as an adverse event) |
| Greenhill et al., 2006b^77^ | **Eligibility criteria**  Children aged 7–17 years; Clinical Global Impression of Severity of Illness (CGI-S) rating of 4 or higher (moderately ill or worse); weight and height between the 5th and 95th percentile based  on the National Center for Health Statistics; intelligence quotient of at least 80; absence of learning disabilities, with a score of at least 80; attending a full-time school (not home school), with a teacher and parent or legal guardian willing to participate; and total and/or factor scores on the teacher-/investigator-rated ADHD-RS-IV School Version at least 1.5 SD above the norm for the patient’s age and gender. Patients were excluded if they had a history or current diagnosis of pervasive developmental disorder, schizophrenia, or other psychotic disorders (DSM-IV axis I); any current psychiatric comorbidity that required pharmacotherapy; any evidence of suicide risk; or ADHD symptoms well controlled on current therapy with tolerable side effects. Patients who had failed to respond to two or more adequate courses (dose and duration) of stimulant therapy for ADHD were also excluded. Additional  exclusion criteria were absolute neutrophil count (ANC) < 1x10^9^/L; hypertension; hypotension; resting heart rate outside the range of 60 to 115 beats per minute; a history of alcohol or substance abuse; and consumption of >250 mg/day of caffeine. Concomitant use of prescription or nonprescription agents with psychotropic properties, including ADHD treatments and dietary supplements, was prohibited within 1 week of the baseline visit and during the study. Monoamine oxidase inhibitors and SSRIs were prohibited within 2 weeks of baseline testing and throughout the study  **Diagnostic criteria**  DSM-IV  **Number randomized**  200  **Age**  7-17 years (range)  **Co-morbidities/Co-medications**  None/Yes (nonopioid analgesics/NSAIDs 33%; respiratory agents 17%; antihistamines 14%; antiinfectives 12%)  **ADHD subtypes**  Combined (70%), inattentive (24%), hyperactive/impulsive (1%) | **Arm 1**  MODAF: modafinil 170-425 mg/day (mean dose: 361.4 mg/day)  **Arm 2**  Placebo: placebo pill | **Treatment response** (as a dichotomous outcome)  Clinician-rated Clinical Global Impression Improvement (CGI-I) scale – e.g. responder considered very much improved or much improved  **All-cause treatment discontinuation** (as a dichotomous outcome)  Yes, reported as study discontinuation  **Tolerability** (as a dichotomous outcome)  Yes, reported as study discontinuation due to AEs  **Serious adverse events** (as dichotomous outcome)  None  **Specific adverse events** (as dichotomous outcome)  Loss of appetite/anorexia (as an adverse event)  Decreased weight (as an adverse event e.g. weight loss)  Insomnia (as an adverse event)  Anxiety (as an adverse event leading to discontinuation)  Cardiovascular event – Tachycardia (as an adverse event leading to discontinuation) |
| Greenhill et al., 2006c^78-80^ | **Eligibility criteria**  Children aged 3.5-5 years; stimulant na*ï*ve, children of both sexes; ADHD combined or predominantly  hyperactive subtype; an impairment scale score <55 on the Children`s Global Assessment Scale;  hyperactive-impulsive subscale T score of 65 (1.5 SDs above the age- and sex-adjusted means) on both the Revised Conners Parent and Teacher Rating Scales; Full Scale IQ equivalent of >70 on the Differential Ability Scales; participation in a preschool, day care group setting, or other school program at least 2 half-days per week with at least eight same-age peers; and the same primary caretaker for at least 6 months before screening.  Children were excluded if there was current evidence of adjustment disorder, pervasive developmental disorders, psychosis, significant suicidality, or other psychiatric disorder in addition to ADHD that required treatment with additional medication; current stimulant or cocaine abuse in a relative living in the home; a confounding medical condition; inability of the parent to understand or follow study instructions, or history of bipolar disorder in both biological parents  **Diagnostic criteria**  DSM-IV  **Number randomized**  114  **Age**  3.5-5 years (range)  **Co-morbidities/Co-medications**  Yes, oppositional disorders (53%), anxiety (11%), communication disability (22%), conduct disorders (3%)/Yes (all prior stimulant exposure and parent training in early phases of the PATS trial)  **ADHD subtypes**  Combined (75%), hyperactive/impulsive (25%) | **Arm 1**  MPH-SA low/medium dose: methylphenidate short-acting (inmediate release) 14.2 mg/day  **Arm 2**  Placebo: placebo pill | **Treatment response** (as a dichotomous outcome)  Response was defined by a cutoff of ≤ 1.0 on the parent-teacher SNAP composite score at the end of treatment  **All-cause treatment discontinuation** (as a dichotomous outcome)  Yes, reported as study discontinuation  **Tolerability** (as a dichotomous outcome)  Yes, reported as study discontinuation due to AEs  **Serious adverse events** (as dichotomous outcome)  Not reported  **Specific adverse events** (as dichotomous outcome)  Not reported |
| Sangal et al., 2006^81^ | **Eligibility criteria**  Children aged 6-14 years; ADHD RS score at least 1.0 SD above normative values for age and sex for either the inattentive or hyperactive/impulsive  subscore, or for the combined score; all patients scored at least 80 on the Wechsler Intelligence Scale for Children. Exclusion criteria included serious medical illness, a history of symptoms suggestive of a primary sleep disorder—such as obstructive sleep apnea, periodic limb movement disorder, or insufficient sleep syndrome (e.g., voluntary sleep restriction resulting in sleep duration habitually significantly shorter than expected age norms)—that could potentially result in a daytime symptom constellation similar to ADHD, and abnormal laboratory values or electrocardiogram (ECG) readings. Patients agreed not to use caffeinated beverages during the duration of the study  **Diagnostic criteria**  DSM-IV  **Number randomized**  85  **Age**  6-14 years (range)  **Co-morbidities/Co-medications**  Yes, oppositional disorders (48%), conduct disorders (4%)/Yes (57% prior stimulant exposure)  **ADHD subtypes**  Combined (67%), inattentive (30%), hyperactive/impulsive (2%) | **Arm 1**  MPH-SA high dose: methylphenidate 42.3 mg/day (or 1.1 mg/kg/day)  **Arm 2**  ATX: atomoxetine 58.3 mg/day (or 1.6 mg/kg/day) | **Treatment response** (as a dichotomous outcome)  Not reported/data not abstractable (for first study period before crossover)  **All-cause treatment discontinuation** (as a dichotomous outcome)  Yes, reported as study discontinuation  **Tolerability** (as a dichotomous outcome)  Yes, reported as study discontinuation due to AEs  **Serious adverse events** (as dichotomous outcome)  Not reported  **Specific adverse events** (as dichotomous outcome)  Not reported/data not abstractable |
| Spencer et al., 2006^82^ | **Eligibility criteria**  Adolescents aged 13 to 17 years, weighing ≤ 75 kg  (≤165 lb), *DSM-IV-TR* criteria for primary diagnosis of ADHD combined subtype (predominantly inattentive subtype or hyperactive-impulsive subtype); an intelligence quotient score ≥80, normal blood pressure, 2s ECG findings within the normal range, and a willingness and ability to comply with protocol requirements in conjunction with a parent or caregiver. Adolescents who were known to be nonresponsive to stimulants (defined as no clinical improvement after trials of 2 stimulant medications, taken for at least 3 weeks each) or naive to stimulant treatment were eligible  for enrollment. Exclusion criteria included comorbid illness that could interfere with study participation or impact the efficacy and tolerability of MAS XR; a history of nonresponse to stimulant medication; a documented allergy or intolerance to MAS, MAS XR, or amphetamines; and medication use (not including ADHD medication) that could affect blood pressure or heart rate. Other exclusion criteria included a current comorbid psychiatric diagnosis except oppositional defiant disorder, hypertension, history of seizure disorder within the last 2 years, tic disorder, Tourette's syndrome, abnormal thyroid function, cardiac disorder, and significant laboratory abnormalities. In addition, patients with a history of drug abuse or who were current abusers of drugs or other substances or who had a parent or guardian who abused drugs were excluded  **Diagnostic criteria**  DSM-IV-TR  **Number randomized**  287  **Age**  13-17 (range)  **Co-morbidities/Co-medications**  Not reported  **ADHD subtypes**  Combined (57%), inattentive (3%), hyperactive/impulsive (3%) | **Arm 1**  MIX-AMPH-LA: mixed amphetamine salts long acting (Adderall XR) 10, 20, 30 or 40 mg/day  **Arm 2**  Placebo: placebo pill | **Treatment response** (as a dichotomous outcome)  Clinician-rated Clinical Global Impression Improvement (CGI-I) scale – e.g. responder considered very much improved or much improved  **All-cause treatment discontinuation** (as a dichotomous outcome)  Yes, reported as study discontinuations  **Tolerability** (as a dichotomous outcome)  Yes, reported as study discontinuations due to AEs  **Serious adverse events** (as dichotomous outcome)  None  **Specific adverse events** (as dichotomous outcome)  Loss of appetite/anorexia (adverse event)  Decreased weight (adverse event)  Insomnia (adverse event) |
| Spencer et al., 2006b^83^ | **Eligibility criteria**  Children and adolescents aged 6 to 17 years with  ODD; normal blood pressure (eg, within the 95th  percentile for their age, height, and sex), an ECG finding within normal range, and no comorbid illness that could affect the efficacy or tolerability of MAS XR. Patients were excluded if they had another psychiatric diagnosis (except ADHD); a diagnosis of conduct disorder; or a medical history of nonresponse to stimulant medication, seizures, tic disorder, or Tourette's syndrome  **Diagnostic criteria**  DSM-IV  **Number randomized**  244  **Age**  6-17 (range)  **Co-morbidities/Co-medications**  All children with oppositional defiant disorder and comorbid ADHD (subanalysis)/Not reported  **ADHD subtypes**  Combined (60%), inattentive (16%), hyperactive/impulsive (6%) | **Arm 1**  MIX-AMPH-LA: mixed amphetamine salts long acting (Adderall XR) 10, 20, 30 or 40 mg/day  **Arm 2**  Placebo: placebo pill | **Treatment response** (as a dichotomous outcome)  Clinician-rated Clinical Global Impression Improvement (CGI-I) scale – e.g. responder considered very much improved or much improved  **All-cause treatment discontinuation** (as a dichotomous outcome)  Not reported/data not abstractable for ADHD  **Tolerability** (as a dichotomous outcome)  Yes, reported as study discontinuations due to AEs  **Serious adverse events** (as dichotomous outcome)  None  **Specific adverse events** (as dichotomous outcome)  Loss of appetite/anorexia (adverse event – review authors applied same % as for overall study population)  Decreased weight (adverse event – review authors applied same % as for overall study population)  Insomnia (adverse event –  review authors applied same % as for overall study population) |
| Steele et al., 2006^84^ | **Eligibility criteria**  Children 6-12 years; medication naïve or currently on ADHD medication therapy; had a baseline Clinical Global Impression-Severity (CGI-S) score of 4 or greater (at least “moderate” severity); and had to demonstrate significant after-school/evening behavioural difficulties as assessed by the clinician via parent/child interviews; psychotropic medications to treat non ADHD disorders and psychological interventions were permitted as long as the treatment/intervention had been stable for a minimum of 4 weeks prior to entry and did not change nor newly commence during the trial. Exclusion criteria included: known MPH non-responders, hypersensitivity, or adversely affected by MPH; concomitant use of contraindicated medication likely to interfere with the safe administration of study medication; marked anxiety, tension, aggression/agitation; glaucoma; ongoing seizure disorder; psychotic disorder; diagnosis or family history of Tourette’s disorder; bipolar disorder; suspected mental retardation or significant learning disorder; medication/alcohol abuse/dependence by either the child or parent; history of, or current eating disorder; severe gastrointestinal narrowing; inability to swallow study medications; and any serious/unstable medical illness  **Diagnostic criteria**  DSM-IV  **Number randomized**  147  **Age**  6-12 years (range)  **Co-morbidities/Co-medications**  Yes, oppositional disorders (41%), conduct disorders (1%)/Not reported  **ADHD subtypes**  Combined (79%), inattentive (18%), hyperactive/impulsive (2%) | **Arm 1**  MPH-SA high dose: methylphenidate short-acting (inmediate release) 33.3 mg/day  **Arm 2**  MPH-LA low/medium dose: methylphenidate long-acting (OROS formulation) 37.8 mg/day | **Treatment response** (as a dichotomous outcome)  Clinician-rated Clinical Global Impression Improvement (CGI-I) scale – e.g. responder considered very much improved or much improved; Clinician-rated Clinical Global Impression Severy (CGI-S) scale – e.g. responder with ratings of 1 (not at all ill), 2 (borderline ill) or 3 (mildly ill); Improvement in the SNAP rating scale  **All-cause treatment discontinuation** (as a dichotomous outcome)  Yes, reported as study discontinuations  **Tolerability** (as a dichotomous outcome)  Yes, resportes as study discontinuations due to AEs  **Serious adverse events** (as dichotomous outcome)  Not reported  **Specific adverse events** (as dichotomous outcome)  Loss of appetite/anorexia (as an adverse event)  Insomnia (as an adverse event)  Sleep disturbances (as an adverse event) |
| Trebatická et al., 2006^85,86^ | **Eligibility criteria**  Children aged 6-14 years; early onset of ADHD—by 6 to 7 years, chronicity—at least 6 months of symptoms, general disposition as restless, inattentive, distractible and disorganized; disorders of cognitive function: inattention, distractibility, difficulty to persist with any task, difficulty in selective process to information, disturbance of the executive functions (production, sequention and realization of plans), disturbance of motivation, effort and fortitude, visuospacial and memory disturbance; disorders in control of activity: child’s inability to suppress activity, abnormality in control of activity, disorganisation and discontinuation of motoric activity; impulsiveness: acting without due reflection,  engaging in rash and sometimes dangerous behaviours, disturbances of emotions and affectivity. Exclusion criteria: situational hyperactivity, pervasive developmental disorders, schizophrenia, other psychotic disorders as mood, anxiety, personality disorder as unsocial behaviour, personality change due to a general medical  condition, mental retardation, understimulating environments, conduct disorder, tics, chorea and other dyskinesias; patients with acute inflammatory diseases, renal and cardiovascular disorders and diabetics  **Diagnostic criteria**  ICD-10  **Number randomized**  61  **Age**  6-14 (range)  **Co-morbidities/Co-medications**  Yes, learning disabilities (30%)/Not reported  **ADHD subtypes**  ICD-10 hiperkinetic disorder (72%), hyperkinetic conduct disorder (18%), attention deficit without hyperactivity (10%) | **Arm 1**  Herbal therapy: Polyphenolic extract from maritime pine bark (*Pinus pinaster*) 1 mg/kg/day  **Arm 2**  Placebo: placebo pill | **Treatment response** (as a dichotomous outcome)  Not reported/data not abstractable  **All-cause treatment discontinuation** (as a dichotomous outcome)  Yes, reported as study discontinuations  **Tolerability** (as a dichotomous outcome)  Not reported  **Serious adverse events** (as dichotomous outcome)  None  **Specific adverse events** (as dichotomous outcome)  Not reported |
| Armenteros et al., 2007^87^ | **Eligibility criteria**  Patients were required to have been treated with a constant dose of stimulant medication during the 3 weeks before entering the study; an Aggression Questionnaire Predatory-Affective index score of 0 or below, indicating primarily an affective or impulsive subtype of aggression. Additional inclusion criteria were a minimum Clinical Global Impressions-Severity (CGI-S) scale rating of 4 (moderately ill), a Full Scale IQ Q75, and  normal results at screening from physical examination and laboratory tests. Exclusions: a  substance use disorder, an unstable medical or neurological illness, a history of intolerance or failure to respond to an adequate trial of risperidone (defined as 2 mg/day for at least 4 weeks), or the patient was suicidal or homicidal. Subjects were allowed to continue receiving any psychosocial treatment that was in place before entering the study. However, subjects were not allowed to seek psychosocial interventions during the study  **Diagnostic criteria**  DSM-IV  **Number randomized**  25  **Age**  7-12 (range)  **Co-morbidities/Co-medications**  Yes, oppositional disorders (52%), conduct disorders (24%), anxiety (16%) – Yes (all received concomitant psychoestimulants)  **ADHD subtypes**  Not reported | **Arm 1**  MPH-SA or MIX-AMPH + RISP: combination of risperidone (1.08 mg/day) and methylphenidate (18-54 mg/day) or mixed amphetamine salts (10-40 mg/day)  **Arm 2**  MPH-SA or MIX-AMPH: methylphenidate (18-54 mg/day) or mixed amphetamine salts (10-40 mg/day) | **Treatment response** (as a dichotomous outcome)  Clinician-rated Clinical Global Impression Improvement (CGI-I) scale – e.g. responder considered completely recovered, very much improved or much improved (CGI ≤ 3)  **All-cause treatment discontinuation** (as a dichotomous outcome)  Yes, reported as study discontinuations  **Tolerability** (as a dichotomous outcome)  Not reported  **Serious adverse events** (as dichotomous outcome)  None  **Specific adverse events** (as dichotomous outcome)  Not reported |
| Arnold et al., 2007^88^ | **Eligibility criteria**  Children aged 5–12 with ADHD (any  of the 3 subtypes); an item mean on either the 18 DSM-IV ADHD symptoms or 9 inattentive symptoms of 1.5 or more (on a 0 to 3 scale) averaging both informants (parent and teacher); reasonably good health during the 4 weeks immediately prior to initial screening as demonstrated by medical history, physical examination, and laboratory testing; failure of FDA-approved therapy or stopped therapy due to adverse events or declined or stopped approved therapy because of parental/guardian  concern about the risks of approved drugs. Exclusion criteria were: severe medical, surgical, or neurological problems; anything that would interfere with treatment or assessment; co-morbid diagnoses requiring treatment with psychoactive medication; a prior history of carnitine therapy in the past three months prior to baseline; use of any investigational drug in the 30 days prior to baseline; use of any medication or supplement to treat ADHD; a frank deficiency of magnesium or zinc, increased levels of lead above 10mcg/dL; impaired renal or liver function; pathologically abnormal peripheral blood count; neuroleptic medication in the past six months; body weight below 13.5 kg, missing one-quarter of school days in the previous two months; another child in the same household or classroom already in the study; no telephone; a non-English speaking primary caretaker; important changes expected in school or home situation, (divorce, relocating) during the course of the trial period; or actively suicidal or homicidal  **Diagnostic criteria**  DSM-IV  **Number randomized**  118  **Age**  5-12 years (range)  **Co-morbidities/Co-medications**  Yes, oppositional disorders (11%), other (18%)/No use of any medication or supplement to treat ADHD  **ADHD subtypes**  Combined (60%), inattentive (35%), hyperactive/impulsive (5%) | **Arm 1**  L-CARN: l-carnitine 1-3 g/day  **Arm 2**  Placebo: placebo pill | **Treatment response** (as a dichotomous outcome)  Clinician-rated Clinical Global Impression Improvement (CGI-I) scale – e.g. responder considered very much improved or much improved  **All-cause treatment discontinuation** (as a dichotomous outcome)  Yes, reported as study withdrawals  **Tolerability** (as a dichotomous outcome)  Not reported  **Serious adverse events** (as dichotomous outcome)  Not reported  **Specific adverse events** (as dichotomous outcome)  Not reported |
| Bangs et al., 2007^89,90^ | **Eligibility criteria**  Adolescents 12–18 years who met the criteria for both ADHD and MDD DSM-IV, both diagnoses  were confirmed by the Kiddie Schedule for Affective Disorders and Schizophrenia for School  Aged Children–Present and Lifetime Version. For inclusion, patients were required to have a score on the ADHDRS-IV-Parent:Inv at least 1.5 SD above age and sex norms and a Children’s Depression  Rating Scale–Revised total score of ≥40 at every visit prior to randomization (visit 4). Patients beginning structured psychotherapy for ADHD and/or depression less than 1 month before trial entry were excluded  **Diagnostic criteria**  DSM-IV  **Number randomized**  142  **Age**  12-17.9 years (range)  **Co-morbidities/Co-medications**  Yes, all co-morbid major depression – Yes, 81% prior stimulant exposure  **ADHD subtypes**  Combined (43%), inattentive (57%) | **Arm 1**  ATX: atomoxetine total dose 1.5 mg/kg/day  **Arm 2**  Placebo: placebo pill | **Treatment response** (as a dichotomous outcome)  Clinician-rated Clinical Global Impression Improvement (CGI-I) scale – e.g. responder considered very much improved or much improved; Clinician-rated Clinical Global Impression Severy (CGI-S) scale  **All-cause treatment discontinuation** (as a dichotomous outcome)  Yes, reported as study discontinuations  **Tolerability** (as a dichotomous outcome)  Yes, reported as study discontinuations due to AEs  **Serious adverse events** (as dichotomous outcome)  Yes, reported  **Specific adverse events** (as dichotomous outcome)  Loss of appetite/anorexia (as an adverse event)  Decreased weight (adverse event as defined by the authors e.g. at least 3.5% reduction – unpublished data)  Cardiovascular event – blood pressure increased (e.g. increase of SBP or DBP at least 5 mm to above 95% percentile – unpublished data)  Decreased pulse values (e.g. decrease of at least 20 to a  value of at most 65) |
| Biederman et al., 2007^91,92^ | **Eligibility criteria**  Children aged 6-12 years with ADHD combined  or hyperactive-impulsive subtypes; ADHD-RS-IV >28 (indicating severity of ADHD symptoms) at study baseline; academic functioning at age-appropriate levels, normal blood pressure and ECG measurements, absence of a history of or current  medical conditions or use of medications that  might confound the results of the study or increase  risk to the patient, and ability to swallow a capsule  were also determined. Exclusion criteria were: comorbid psychiatric diagnosis (e.g. psychosis, bipolar disorder), history of seizures or current diagnosis or family history of Tourette's disorder, obesity based on the investigator's opinion, weight <25 kg (55 lb), positive screening for illicit drug use, and/or current health conditions or use of medications that might confound the results of the  study or increase risk to the patient. Female patients of childbearing potential were required to have a negative result on urine pregnancy testing and were given specific instructions on avoiding pregnancy throughout the period of study drug exposure  **Diagnostic criteria**  DSM-IV  **Number randomized**  290  **Age**  6-12 years (range)  **Co-morbidities/Co-medications**  Not reported – Yes, 36% prior stimulant exposure  **ADHD subtypes**  Combined (96%), hyperactive/impulsive (4%) | **Arm 1**  LDX-LA: lisdexamphetamine dimesylate 30, 50 or 70 mg/day  **Arm 2**  Placebo: placebo pill | **Treatment response** (as a dichotomous outcome)  Clinician-rated Clinical Global Impression Improvement (CGI-I) scale – e.g. responder considered very much improved or much improved; ADHD-RS ≥ 30% reduction from baseline in total score (rater: clinician)  **All-cause treatment discontinuation** (as a dichotomous outcome)  Yes, reported as study discontinuations  **Tolerability** (as a dichotomous outcome)  Yes, reported as study discontinuations due to AEs  **Serious adverse events** (as dichotomous outcome)  Yes, reported  **Specific adverse events** (as dichotomous outcome)  Loss of appetite/anorexia (as an adverse event)  Decreased weight (as an adverse event)  Insomnia (as an adverse event) |
| Buitelaar et al., 2007^93,58^ | **Eligibility criteria**  Children aged 6 to 15 years; symptom severity was at least 1.5 SD above US age and gender norms. Exclusions: patients with bipolar disorder or a psychotic illness; patients with unstable medical illness or patients with a condition that would require ongoing administration of a psychoactive medication (other than ATX).  Note: randomization occurred after approximately 1 year of treatment, with subjects initially randomly assigned to ATX being re-randomized to an additional 6 months (24w) of ATX or placebo substitution  **Diagnostic criteria**  DSM-IV  **Number randomized**  163  **Age**  6-15 years (range)  **Co-morbidities/Co-medications**  Yes, oppositional disorder (44%) and depression (3%)– Yes, 52% prior stimulant exposure  **ADHD subtypes**  Combined (74%), inattentive (21%), hyperactive/impulsive (5%) | **Arm 1**  ATX: atomoxetine 1.6 mg/kg/day  **Arm 2**  Placebo: placebo pill | **Treatment response** (as a dichotomous outcome)  Not reported/data not abstractable  **All-cause treatment discontinuation** (as a dichotomous outcome)  Yes, reported as study discontinuation  **Tolerability** (as a dichotomous outcome)  Yes, reported as study discontinuations due to AEs  **Serious adverse events** (as dichotomous outcome)  Not reported  **Specific adverse events** (as dichotomous outcome)  Not reported |
| Carlson et al., 2007^94^ | **Eligibility criteria**  Children aged 6 to 12 years; ADHD, any type; rating on ADHD-RS of ≥ 1.5 SD above age and sex norms; severity rating of at least moderate on the Clinical Global Impressions Severity Scale; history (preceding 12 months) of insufficient response to an adequate stimulant trial, which was defined as gradual titration of stimulant medication for ≥ 2 weeks at specified doses for each medication. Inadequate response was determined by the child’s prescribing physician, who also documented his or her opinion that a change in treatment was needed; patients must be of normal intelligence, as assessed by the investigator (e.g. without a general impairment of intelligence, and likely, in the investigator’s judgement, to achieve a score ≥ 70 on an IQ test); patients must be able to swallow capsules. Exclusion criteria were: weighed < 22 kg or > 60 kg at study entry; had any other Axis I diagnosis, including pervasive developmental disorder, mood or anxiety disorder (presence of comorbid oppositional defiant disorder was not  an exclusion criterion); nipolar disorder, autism; any medical conditions that would contraindicate the use of ATX or MPH-LA  **Diagnostic criteria**  DSM-IV  **Number randomized**  21  **Age**  6-12 years (range)  **Co-morbidities/Co-medications**  Yes, oppositional disorder (50%) – Yes, all prior stimulant exposure  **ADHD subtypes**  Combined (79%) | **Arm 1**  MPH-LA low/medium dose+ATX: combination of methylphenidate long-acting (1.08 mg/kg/day) and atomoxetine (1.2 mg/kg/day)  **Arm 2**  ATX: atomoxetine 1.2 mg/kg/day | **Treatment response** (as a dichotomous outcome)  Not reported/data not abstractable  **All-cause treatment discontinuation** (as a dichotomous outcome)  Yes, reported as study discontinuation  **Tolerability** (as a dichotomous outcome)  Yes, reported as study discontinuations due to AEs  **Serious adverse events** (as dichotomous outcome)  Not reported  **Specific adverse events** (as dichotomous outcome)  Insomnia (as an adverse event)  Cardiovascular event – blood pressure increased (as an adverse event)  Supraventricular extrasystole (as an adverse event) |
| Gau et al., 2007^95,96^ | **Eligibility criteria**  Children aged 6 to 16 years; a total score  on the ADHD RS-IV–Parent version:Investigator-Administered and scored of at least 25 for boys or 22 for girls, or greater than 12 for their diagnostic subtype at both visit 1 and visit 2; a Clinical Global  Impression–Severity (CGI-S) score ≥4 at both visit 1 and visit 2; normal intelligence as judged by investigators; and no ADHD treatment medication, or completion of the washout procedures before entering this study. Exclusion criteria were: subjects weighed less than 20 kg or more than 60 kg; had a serious medical illness, such as a cardiovascular disease; had a history of bipolar I or II disorder, psychosis, or pervasive developmental disorder; had anxiety disorder; had a history of any  seizure disorder or prior EEG abnormalities related to epilepsy, or had taken (or were taking) anticonvulsants for seizure control; had a history of alcohol or drug abuse within the past 3 months; or if they might have to use psychoactive medications other than the study drug during the study period  **Diagnostic criteria**  DSM-IV  **Number randomized**  106  **Age**  6-16 years (range)  **Co-morbidities/Co-medications**  Yes, oppositional disorder (44%) and depression (3%)– Yes, 58% prior stimulant exposure  **ADHD subtypes**  Combined (73%), inattentive (27%) | **Arm 1**  ATX: atomoxetine 1.4 mg/kg/day (or 43.1 mg/kg)  **Arm 2**  Placebo: placebo pill | **Treatment response** (as a dichotomous outcome)  Responders based on the achievement of a 25% or greater decrease from baseline to endpoint in the ADHD-RS-IV-Parent:Inv total score (rater: parents)  **All-cause treatment discontinuation** (as a dichotomous outcome)  Yes, reported as study discontinuation  **Tolerability** (as a dichotomous outcome)  Yes, reported as study discontinuations due to AEs  **Serious adverse events** (as dichotomous outcome)  Yes, reported  **Specific adverse events** (as dichotomous outcome)  Loss of appetite/anorexia (as an adverse event)  Decreased weight (as an adverse event e.g. weight loss)  Insomnia (as an adverse event) |
| Geller et al., 2007^97,98^ | **Eligibility criteria**  Children aged 8-17 years; who met the DSM-IV  criteria for ADHD and for at least one of the  following anxiety disorders: separation anxiety disorder, generalized anxiety disorder, or social phobia; had a total or subscale score  on the ADHD-RSIV-Parent version of at least 1.5 SDs above age and sex norms for ADHD subtype, and a total score on the Pediatric Anxiety Rating Scale (PARS) of at least 15 (maximum score 25). Exclusion criteria were: significant abnormalities in  baseline laboratory or ECG results; met diagnostic criteria for current posttraumatic stress disorder, panic disorder, specific phobias, or obsessive-compulsive disorder; scored Q15 on the Children`s Yale-Brown Obsessive-Compulsive Scale; or had a history of hypertension or bipolar, psychotic, pervasive developmental, or seizure disorders;  pregnant and lactating females, users of monoamine oxidase inhibitors within 2 weeks of visit 2, recent substance abusers, and individuals at serious suicidal risk or with medical or personal conditions likely to affect the trial or health outcomes. Concomitant use of drugs that inhibit the CYP2D6 enzyme pathway were not allowed due to potential interactions  **Diagnostic criteria**  DSM-IV  **Number randomized**  176  **Age**  8-17 years (range)  **Co-morbidities/Co-medications**  Yes, all co-morbid anxiey disorder (100%), and oppositional disorder (43%) and conduct disorders (2%)– Yes, 58% prior stimulant exposure  **ADHD subtypes**  Combined (75%), inattentive (24%) | **Arm 1**  ATX: atomoxetine 1.3 mg/kg/day  **Arm 2**  Placebo: placebo pill | **Treatment response** (as a dichotomous outcome)  Responders based on the achievement of a 25% or greater decrease from baseline to endpoint in the ADHD-RS-IV-Parent:Inv total score (rater: parents)  **All-cause treatment discontinuation** (as a dichotomous outcome)  Yes, reported as study discontinuation  **Tolerability** (as a dichotomous outcome)  Yes, reported as study discontinuations due to AEs  **Serious adverse events** (as dichotomous outcome)  Yes, reported  **Specific adverse events** (as dichotomous outcome)  Loss of appetite/anorexia (as an adverse event) |
| Prasad et al., 2007^99,100^ | **Eligibility criteria**  Children were 7–15 years; not intellectually impaired in the viewpoints of the investigators;  symptom severity score ≥ 1.5 SD above the investigator-rated ADHD‑RS age norm for their ADHD subtype to be eligible. Patients were excluded if they weighed < 20 kg; had a history of bipolar disorder, psychotic disorders, pervasive development disorder (autistic spectrum disorder), any seizure disorder or alcohol/drug abuse; were with significant prior/current medical conditions or at serious suicidal risk; or were taking medication that could potentially interfere with study outcomes. Females who were pregnant/breastfeeding or sexually active and not using contraception were also excluded  **Diagnostic criteria**  DSM-IV  **Number randomized**  201  **Age**  7-15 years (range)  **Co-morbidities/Co-medications**  Yes, oppositional disorder (62%) and conduct disorders (7%)– Not reported  **ADHD subtypes**  Combined (91%), inattentive (8%), hyperactive/impulsive (2%) | **Arm 1**  ATX: atomoxetine 1.5 mg/kg/day  **Arm 2**  Standard care (control) | **Treatment response** (as a dichotomous outcome)  Responders based on the achievement of a 25% decrease from baseline to endpoint in the ADHD-RS total score (rater: investigator/clinician); Clinician-rated Clinical Global Impression Improvement (CGI-I) scale – e.g. responder considered very much improved or much improved (CGI-I ≤ 2); Clinician-rated Clinical Global Impression Severy (CGI-S) scale – e.g. GGI-S ≤ 3  **All-cause treatment discontinuation** (as a dichotomous outcome)  Yes, reported as study discontinuation  **Tolerability** (as a dichotomous outcome)  Yes, reported as study discontinuations due to AEs  **Serious adverse events** (as dichotomous outcome)  None  **Specific adverse events** (as dichotomous outcome)  Loss of appetite/anorexia (as an adverse event)  Decreased weight (as an adverse event) |
| van den Hoofdakker et al., 2007^101^ | **Eligibility criteria**  Children aged 4-12 years; IQ >80 (Full Scale IQ of the WISC-III-R, for children under the age of 6 years; the Full Scale IQ of the WPPSI-R); and both parents (if present) were willing to participate in the BPT program  **Diagnostic criteria**  DSM-IV  **Number randomized**  96  **Age**  4-12 years (range)  **Co-morbidities/Co-medications**  Yes, oppositional disorders (76%), conduct disorders (16%), anxiety (44%), depression (9%), tic disorders (18%), elimination disorder (22%) – Not reported  **ADHD subtypes**  Not reported | **Arm 1**  BT (parent training): The manual-based behavioral parent traning (BP) consisted of twelve 120-minute  sessions of group parent training led by two psychologists. Six children’s parents could participate in each group. Specific target behaviors were established for each child. The BPT program drew  most of its techniques from the programs of Barkley and Forehand and McMahon. The parenting skills dealt with in the program were structuring the environment, setting rules, giving instructions, anticipating misbehaviors, communicating, reinforcing positive behavior, ignoring, employing punishment, and implementing token systems. Psychoeducation and cognitive restructuring of parental cognitions were also important elements. Compared with other typical ADHD parent training programs, the first phase of the training focused strongly on teaching parents to anticipate misbehaviors and to manipulate the antecedents. Homework assignments played a central role in the program. For each session, parents read a chapter of a book especially written for this purpose. In addition, parents practiced each week the parenting skill that was introduced in the preceding session. All of the exercises were tailored to the specific target behaviors of each child. The parents wrote reports after the exercises. Each session started with a discussion of the homework assignments and the parental reports. Then a new topic was introduced. The sessions ended with the preparations for new  homework assignments.  **Arm 2**  Standard care (control): The psychiatrists were instructed to provide care as usual, including supportive counseling, psychoeducation, pharmacotherapy, and crisis management whenever necessary | **Treatment response** (as a dichotomous outcome)  Not reported/data not abstractable  **All-cause treatment discontinuation** (as a dichotomous outcome)  Yes, reported as study disontinuations  **Tolerability** (as a dichotomous outcome)  Not reported  **Serious adverse events** (as dichotomous outcome)  Not reported  **Specific adverse events** (as dichotomous outcome)  Not reported |
| van der Oord et al., 2007^102^ | **Eligibility criteria**  Children aged 8-12 years; an estimated full scale IQ  of 75 or above based on a short version of the  Wechsler Intelligence Scale for Children-Revised  (WISC-R). Exclusion criteria were inadequate mastering of the Dutch language by the child or both parents, and a history of MPH use  **Diagnostic criteria**  DSM-IV  **Number randomized**  50  **Age**  8-12 years (range)  **Co-morbidities/Co-medications**  Yes, oppositional disorders and conduct disorders (52%) /Not reported  **ADHD subtypes**  Not reported | **Arm 1**  MPH-SA low/medium dose: methylphenidate 20.8 mg/day (mean dose)  **Arm 2**  BT (child, parent and teacher training): Behavioral therapy included parent training, child-focused treatment, and school-based intervention. The parent BT consisted of 10 weekly sessions of 90 min group therapy for four or five parent couples, provided by  two therapists. The parent training was based on  Barkley’s training: ‘‘Defiant children: A clinicians  manual for parent training’’. Components included  psycho-education on ADHD, structuring the environment, practicing positive attending skills,  giving effective behavioral commands to the child, contingency management skills, and knowledge of parenting techniques such as time-out. The teacher training was based on the teachers training manual by Pelham: ‘‘Attention deficit hyperactivity disorder, diagnosis, nature, etiology and treatment’’. The teacher training consisted of a two-hour workshop, in which psycho-education on ADHD, structuring the classroom environment, implementing contingency management in the classroom, and a daily report card  (DRC) system were explained to the teacher.  The DRC is a classroom contingency management  technique where parents provide rewards based on the teacher’s ratings of the child’s classroom behavior for that day. Teachers received an extensive handout of the training and weekly additional contacts by phone, during which the implementation of behavioral techniques was monitored, the use of the DRC was evaluated, and possible problems were discussed. The child BT consisted of 10 weekly 75-min group sessions for four or five children, provided by two therapists. The program used was adapted from Kendall and Braswell. Cognitive-behavioral techniques consisted of the children acquiring problem- solving techniques. Relaxation and contingency management techniques were also used. Training comprised modeling by the therapists, role-playing, and guided practice. Academic and interpersonal problems were extensively covered, to ensure generalization across the wide range of problem behaviors. In addition, a token reinforcement system was used during the group sessions | **Treatment response** (as a dichotomous outcome)  Not reported/data not abstractable  **All-cause treatment discontinuation** (as a dichotomous outcome)  Yes, reported as study disontinuations  **Tolerability** (as a dichotomous outcome)  Not reported  **Serious adverse events** (as dichotomous outcome)  Not reported  **Specific adverse events** (as dichotomous outcome)  Not reported |
| Wang et al., 2007^103,104^ | **Eligibility criteria**  Children and adolescents aged 6-16 years; weighing between 20 and 60 kg; all patients were required to meet the following symptom severity thresholds: a score of ≥25 for boys or ≥22 for girls, or >12 for a specific subtype, on the ADHDRS-IVParent:Inv), as well as a CGI-ADHD-S score of ≥4.  Exclusion criteria included: any history of bipolar, psychotic or pervasive developmental disorders; suicidal risk; or ongoing use of psychoactive medications other than the study drug. Patients with motor tics, a diagnosis or family history of Tourette’s syndrome or those who met DSM-IV criteria for anxiety disorder as assessed by the investigator and confirmed by the K-SADS-PL were also excluded from participating  **Diagnostic criteria**  DSM-IV  **Number randomized**  330  **Age**  6-16 years (range)  **Co-morbidities/Co-medications**  Yes, oppositional disorders (24%)/Yes (24% prior stimulant exposure)  **ADHD subtypes**  Combined (59%), inattentive (38%), hyperactive/impulsive (3%) | **Arm 1**  MPH-SA low/medium dose: methylphenidate 0.2-0.6 mg/kg/day  **Arm 2**  ATX: atomoxetine 0.8-1.8 mg/kg/day | **Treatment response** (as a dichotomous outcome)  Responders based on the achievement of a 40% or greater decrease from baseline to endpoint in the ADHD-RS-IV-Parent:Inv total score (rater: parents)  **All-cause treatment discontinuation** (as a dichotomous outcome)  Yes, reported as study discontinuation  **Tolerability** (as a dichotomous outcome)  Yes, reported as study discontinuation due to AEs  **Serious adverse events** (as dichotomous outcome)  Yes, reported  **Specific adverse events** (as dichotomous outcome)  Loss of appetite/anorexia (as an adverse event)  Insomnia (as an adverse event)  Cardiovascular event – palpitations (as an adverse event) |
| Amiri et al., 2008^105^ | **Eligibility criteria**  Children aged 6–15 years; total and/or subscale scores on ADHD-RS-IV School Version at least 1.5 SD above norms for patient's age and gender. All patients had combined subtype of ADHD and were newly diagnosed. Children were excluded if  they had a history or current diagnosis of pervasive developmental disorders, schizophrenia or other psychiatric disorders (DSM-IV axis I); any current psychiatric comorbidity that required pharmacotherapy; any evidence of suicide risk and mental retardation (IQ<70 based on clinical judgment). In addition, patients were excluded if they had a clinically significant chronic medical condition, including organic brain disorder, seizures and, current abuse or dependence on drugs within 6 months. Additional exclusion criteria were hypertension, hypotension and habitual consumption of more than 250 mg/day of caffeine  **Diagnostic criteria**  DSM-IV-TR  **Number randomized**  60  **Age**  6-15 years (range)  **Co-morbidities/Co-medications**  Not reported/Not reported  **ADHD subtypes**  Combined (100%) | **Arm 1**  MPH-SA low/medium dose: methylphenidate 20-30 mg/day  **Arm 2**  MODAF: modafinil 200-300 mg/day | **Treatment response** (as a dichotomous outcome)  Responders based on the achievement of a 40% or greater decrease from baseline to endpoint in the ADHD-RS total score (rater: parents and teachers)  **All-cause treatment discontinuation** (as a dichotomous outcome)  Yes, reported as study discontinuation  **Tolerability** (as a dichotomous outcome)  Not reported  **Serious adverse events** (as dichotomous outcome)  Not reported  **Specific adverse events** (as dichotomous outcome)  Loss of appetite/anorexia (as an adverse event)  Decreased weight (as an adverse event e.g. weight loss)  Anxiety (as an advserse event e.g. anxiety/nervousness)  Sleep disturbances (as an adverse event e.g. difficulty falling asleep) |
| Bangs et al., 2008^106,107^ | **Eligibility criteria**  Children aged 6 to 12 years with ADHD (any subtype) and comorbid ODD; a structured interview (Kiddie Schedule for Affective Disorders and Schizophrenia for School Aged Children-Present and Lifetime Version); SNAP-IV ADHD subscale score above age and gender norms; Clinical Global Impressions-Severity Scale score ≥4 at visits 1 and 2; and SNAP-IV ODD subscale score of ≥15 at both visits 1 and 2. If other comorbid conditions were present, either ADHD or ODD was the primary diagnosis. Patients who had a history of bipolar I or II disorder, psychosis, or pervasive developmental disorder were excluded. Patients also were excluded if they had a current diagnosis of major depressive disorder, posttraumatic stress disorder, a Children’s Depression Rating Scale-  Revised total raw score >40 at visit 1, or if they were determined to be at serious suicidal risk. Patients with a history of any seizure disorder (other than febrile seizures), a history of alcohol or drug abuse within the past 3 months, current cardiovascular disease or other conditions that could be aggravated by an increased heart  rate or increased blood pressure, a medical condition that would markedly increase sympathetic nervous system activity, or severe gastrointestinal narrowing were excluded. Finally, patients who, in the investigator’s judgment, were likely to need psychotropic medications apart from the drug under study or who at any time during the study were likely to begin structured psychotherapy were excluded  **Diagnostic criteria**  DSM-IV  **Number randomized**  226  **Age**  6-12 years (range)  **Co-morbidities/Co-medications**  Yes, all co-morbid oppositional disorders (100%), conduct disorders (12%), anxiety (2%) – Yes, 69% prior stimulant exposure  **ADHD subtypes**  Combined (85%), inattentive (9%), hyperactive/impulsive (6%) | **Arm 1**  ATX: atomoxetine 1.2 mg/kg/day  **Arm 2**  Placebo: placebo pill | **Treatment response** (as a dichotomous outcome)  Not reported/data not abstractable  **All-cause treatment discontinuation** (as a dichotomous outcome)  Yes, reported as study discontinuation  **Tolerability** (as a dichotomous outcome)  Yes, reported as study discontinuations due to AEs  **Serious adverse events** (as dichotomous outcome)  Yes, reported  **Specific adverse events** (as dichotomous outcome)  Loss of appetite/anorexia (as an adverse event)  Decreased weight (as an adverse event e.g. weight loss defined as a decrease of 3.5% from baseline weight)  Cardiovascular event – blood pressure increase (as an adverse event e.g. increased DBP of ≥ 5 mm to above the 95^th^ percentile) |
| Bierdeman et al., 2008^108-110^ | **Eligibility criteria**  Children and adolescent aged 6-17 years; with  function intellectually at age-appropriate levels; ECG results within the reference range; and have blood pressure measurements within the 95th percentile for their age, gender, and height.  Patients were excluded from the study when they had a current, uncontrolled, comorbid psychiatric diagnosis (except oppositional defiant disorder) with significant symptoms, such as any severe comorbid Axis II disorder or severe Axis I disorder, or when other symptomatic manifestations would, in the opinion of the examining physician, contraindicate GXR treatment or confound efficacy or safety assessments. Patients who weighed  <55 lb or were morbidly overweight or obese, pregnant, lactating, or hypertensive were also excluded. In addition, patients were not enrolled when they had any of the following: a QTc interval of >440 milliseconds; a history of seizure during the past 2 years (exclusive of febrile seizures); a tic disorder; family history of Tourette’s disorder; a positive urine drug screen; any abnormal thyroid function that was not adequately treated; or any cardiac condition or family history of cardiac condition that, in the opinion of the physician investigator, would require exclusion. Patients who had taken an investigational drug within 28 days, were taking medications that affect BP or heart rate, or were taking other medications that have central nervous system effects or affect performance were also not eligible to participate  **Diagnostic criteria**  DSM-IV  **Number randomized**  345  **Age**  6-17 years (range)  **Co-morbidities/Co-medications**  Not reported/Not reported  **ADHD subtypes**  Combined (72%), inattentive (26%), hyperactive/impulsive (2%) | **Arm 1**  GUAN-LA: guanfacine long acting 2, 3 or 4 mg/day  **Arm 2**  Placebo: placebo pill | **Treatment response** (as a dichotomous outcome)  Global improvement based on Clinical Global Impression Improvement (CGI-I) scale. Subjects with improvement of “very much” or “much” improved were considered responders (rating evaluations reported by clinician);  Global improvement in Parent Global Assessment (PGA). Subjects with improvement of “very much” or “much” improved were considered responders (rating evaluations reported by parents)  **All-cause treatment discontinuation** (as a dichotomous outcome)  Yes, reported as study withdrawals  **Tolerability** (as a dichotomous outcome)  Yes, reported as study withdrawals due tu adverse effects  **Serious adverse events** (as dichotomous outcome)  Yes, reported  **Specific adverse events** (as dichotomous outcome)  Loss of appetite/anorexia (as an adverse event)  Insomnia (as an adverse event)  Cardiovascular event – QT prolongation (as an adverse event)  Bradycardia (as an adverse event) |
| Palumbo et al., 2008^111-113^ | **Eligibility criteria**  Children 7-12 years in school; presence of a sufficient number of ADHD symptoms (rated as pretty much or very much) in the classroom setting using the Disruptive Behavior Disorders Rating Scale7 (updated to DSM-IV) to meet DSM-IV criteria and rate the severity of ADHD symptoms above specified cutoff scores (boys: grades 2-3 = 10; grade 4 and above = 9; girls: grades 2-3 = 7, grade 4 and above = 6) on the Iowa Conners Teacher Rating Scale. A designated parent in daily contact with the subject  also had to indicate the presence of sufficient ADHD symptoms at home on the Iowa Conners Parent Rating Scale. The investigator’s rating of global functioning on the Child Global Assessment Scale (CGAS) had to be ≤70 with difficulty evident in at least two areas, such as school and home. Exclusions were: evidence of a tic disorder, major depression, pervasive developmental disorder, autism, psychosis, mental retardation, anorexia nervosa, bulimia, a serious cardiovascular (e.g., significant hypotension, congenital heart disease) or other medical disorder that would preclude the safe use of MPH or CLON, impaired renal function (a routine urinalysis was performed), or pregnancy (a urine pregnancy test was performed for all adolescent girls). Family history of long QT syndrome, cardiomyopathy, or premature sudden  death were also exclusions. Subjects could not receive any other medications for the treatment of ADHD or other associated psychiatric symptoms  **Diagnostic criteria**  DSM-IV  **Number randomized**  122  **Age**  7-12 years (range)  **Co-morbidities/Co-medications**  Yes, oppositional disorder (47%), conduct disorders (9%) – Yes (47% prior stimulants exposure)  **ADHD subtypes**  Combined (76%), inattentive (20%), hyperactive/impulsive (4%) | **Arm 1**  MPH-SA low/medium dose: methylphenidate 30 mg/day  **Arm 2**  CLON-SA: clonidine 0.24 mg/day  **Arm 3**  MPH-SA low/medium dose+CLON-SA: combination of both methylphenidate (25 mg/day) and clonidine (0.23 mg/day)  **Arm 4**  Placebo: placebo pill | **Treatment response** (as a dichotomous outcome)  Not reported/data not abstractable  **All-cause treatment discontinuation** (as a dichotomous outcome)  Yes, reported as study withdrawals  **Tolerability** (as a dichotomous outcome)  Yes, reported as study withdrawals due to AEs  **Serious adverse events** (as dichotomous outcome)  Yes, reported  **Specific adverse events** (as dichotomous outcome)  Loss of appetite/anorexia (as a moderate or severe adverse event on Pittsburg Side Effect Rating Scale rated by parents e.g. loss of appetite)  Insomnia (as an adverse event)  Sleep distubances (as a moderate or severe adverse event on Pittsburg Side Effect Rating Scale rated by parents e.g. trouble sleeping)  Cardiovascular event – ECG abnormal (as an adverse event leading to withdrawal)  Tachycardia (as an adverse event leading to withdrawal)  Palpitations (as a moderate or severe adverse event on Pittsburg Side Effect Rating Scale rated by parents) |
| Findling et al., 2008^114^ | **Eligibility criteria**  Children 6-12 years; stimulant-naive or known to be stimulant-responsive; IQ ≥ 80; total score of 26 on ADHD Rating Scale, Fourth Edition, while unmedicated; normal laboratory parameters and vital signs, including ECG; females of childbearing potential must have a negative serum beta human  chorionic gonadotropin (HCG) pregnancy test at screening and a negative urine  pregnancy test at baseline. Exclusion criteria were:  comorbid psychiatric diagnosis (except oppositional defiant disorder); history of seizures during the past 2 years; tic disorder; any concurrent illness or skin disorder that might compromise safety or study assessments; ingestion of CLON, ATX, antidepressants, antihypertensives, investigational medications, hepatic or cytochrome (p 450) enzyme-altering agents, medications with central nervous system effects, sedatives, antipsychotics or anxiolytics within the 30 days before study entry; Overweight (body mass index (BMI)-for-age > 90th percentile)  **Diagnostic criteria**  DSM-IV  **Number randomized**  282  **Age**  6-12 years (range)  **Co-morbidities/Co-medications**  Not reported/Not reported  **ADHD subtypes**  Combined (81%), inattentive (17%), hyperactive/impulsive (1%) | **Arm 1**  MPH-LA low/medium dose: methylphenidate 18, 30, 54 mg/day (56% with ≤ 30 mg/day)  **Arm 2**  MPH-TS: methylphenidate transdermal system (patch) 10-30 mg over 9-h periods  **Arm 3**  Placebo: placebo pill | **Treatment response** (as a dichotomous outcome)  Global improvement based on Clinical Global Impression Improvement (CGI-I) scale. Subjects with improvement of “very much” or “much” improved were considered responders (rating evaluations reported by clinician);  Global improvement in Parent Global Assessment (PGA). Subjects with improvement of “very much” or “much” improved were considered responders (rating evaluations reported by parents)  **All-cause treatment discontinuation** (as a dichotomous outcome)  Yes, reported as study withdrawals  **Tolerability** (as a dichotomous outcome)  Yes, reported as study withdrawals due to AEs  **Serious adverse events** (as dichotomous outcome)  Not reported  **Specific adverse events** (as dichotomous outcome)  Loss of appetite/anorexia (as an adverse event)  Decreased weight (as an adverse event)  Insomnia (as an adverse event) |
| Heriot et al., 2008^115^ | **Eligibility criteria**  Children 3-6 years; resident with a primary caregiver for ≥ 6 months; features of ADHD had to be present for ≥ 12 months and to a degree that was considered to be developmentally inappropriate and functionally inappropriate and  functionally impairing across settings; above the 93rd percentile on the Global Index subscale of the Conners’ Rating Scales. Exclusion criteria were: currently in hospital; currently in another treatment study; currently receiving treatment; full scale IQ < 80; pervasive developmental disorder or psychosis; major neurological or medical illness that would interfere with participation or require medications incompatible with MPH; chronic serious tics or Tourette’s disorder; history of child abuse; inability of parent to understand English  **Diagnostic criteria**  DSM-IV  **Number randomized**  16  **Age**  3-6 years (range)  **Co-morbidities/Co-medications**  Yes, oppositional disorders (31%)/No  **ADHD subtypes**  Combined (25%), inattentive (19%), hyperactive/impulsive (56%) | **Arm 1**  MPH-LA low/medium dose: methylphenidate 0.3 mg/kg/day  **Arm 2**  BT (parent training): Behavioral therapy included parent training programme. The parent training involved Step 1: Programme orientation and review of ADHD including knowledge of ‘normal’development, aswell as issues pertaining to ADHD. Step 2: Parent–child relations and principles of behaviour management. Step 3: Enhancing parental attending skills (in the context of special play time exercise). Step 4: Paying positive attention to appropriate independent play and compliance and giving commands more effectively. Step 5: Establishing home token system. Step 6: Refinement of token system. Step 7: Using time out from reinforcement. Step 8: Extending time out to other behaviours and managing the child’s behaviour in public places. Step 9: Handling future behaviour problems. Step 10: Individual booster session (within 1 month later). In the final session, the parent completed several rating scales including the parent versions of the ADHD Rating Scale and Rating Scale IV (A), Conners’ Rating Scale, Family Environment  Scale (Real form), Parental Acceptance-Rejection Questionnaire and the evaluation form. The parent also was given forms for the teacher to complete and post back.  **Arm 3**  MPH-SA low/medium dose+BT: combination of both methylphenidate (0.3 mg/kg/day) and BT parent training (as described above)  **Arm 4**  Placebo: placebo pill | **Treatment response** (as a dichotomous outcome)  ADHD Rating Scales-Parent and Teacher Versions calculating RCI scores. The RCI procedure was used to assess clinical significance of change (treatment response) because of intervention. The RCI is equal to the difference between a child’s pre-treatment and posttreatment  score divided by the standard error of difference between the two test scores. When this exceeds 1.96, it is assumed that change is due to the effects of treatment rather than chance (*P* < 0.05)  **All-cause treatment discontinuation** (as a dichotomous outcome)  Not reported  **Tolerability** (as a dichotomous outcome)  Not reported  **Serious adverse events** (as dichotomous outcome)  Not reported  **Specific adverse events** (as dichotomous outcome)  Not reported |
| Konofal et al., 2008^116^ | **Eligibility criteria**  Children aged 5-8 years; had serum ferritin levels <30 ng/mL (retaining the definition of iron deficiency from a previous study) with normal hemoglobin levels at the screening. Exclusion criteria were: IQ <80 by the French version of the Wechsler Intelligence Scale, third edition, for children, relevant psychiatric comorbidities (depressive, anxiety, and sleep disorders according to DSM-IV criteria), or chronic medical conditions (including malnutrition), children who had received  iron supplementation in the past 3 months or previous treatment with psychotropic agents or psychostimulants  **Diagnostic criteria**  DSM-IV  **Number randomized**  23  **Age**  5-8 years (range)  **Co-morbidities/Co-medications**  No – Not reported  **ADHD subtypes**  Not reported | **Arm 1**  Iron: oral iron (Ferrous sulfate 80 mg/day)  **Arm 2**  Placebo: placebo pill | **Treatment response** (as a dichotomous outcome)  Global improvement based on Clinical Global Impression Severy(CGI-S) scale. Subjects with improvement of “very much” or “much” improved were considered responders (rating evaluations reported by clinician)  **All-cause treatment discontinuation** (as a dichotomous outcome)  Yes, reported as study discontinuation  **Tolerability** (as a dichotomous outcome)  Yes, reported as study discontinuation due to AEs  **Serious adverse events** (as dichotomous outcome)  Not reported  **Specific adverse events** (as dichotomous outcome)  Not reported |
| Newcorn et al., 2008^117,118^ | **Eligibility criteria**  Children aged 6–16 years; Symptom severity at entry was required to be ≥ 1.5 SD above US age and sex norms, as assessed by the ADHD-RS- Parent Version; ADHD as the primary diagnosis. Exclusion criteria were: Seizures, bipolar disorder, psychotic illness or pervasive developmental disorder; taking concomitant psychoactive medications; anxiety or tic disorders or both; previously treated with an adequate trial of MPH or AMPH and either did not experience at least some improvement in ADHD signs and symptoms (non-responders) or did experience intolerable adverse events  **Diagnostic criteria**  DSM-IV  **Number randomized**  516  **Age**  6-16 years (range)  **Co-morbidities/Co-medications**  Not reported/Not reported  **ADHD subtypes**  Combined (70%), inattentive (28%), hyperactive/impulsive (2%) | **Arm 1**  MPH-LA low/medium dose: methylphenidate OROS 39.9 mg/day  **Arm 2**  ATX: atomoxetine 1.45 mg/kg/day  **Arm 3**  Placebo: placebo pill | **Treatment response** (as a dichotomous outcome)  Responders based on the achievement of a 40% or greater decrease from baseline to endpoint in the ADHD-RS total score (rater: parents)  **All-cause treatment discontinuation** (as a dichotomous outcome)  Yes, reported as study discontinuation  **Tolerability** (as a dichotomous outcome)  Yes, reported as study discontinuation due to AEs  **Serious adverse events** (as dichotomous outcome)  Yes, reported  **Specific adverse events** (as dichotomous outcome)  Loss of appetite/anorexia (as an adverse event)  Decreased weight (as an adverse event e.g. ≥ 3.5% of body weight loss)  Insomnia (as an adverse event)  Cardiovascular event – blood pressure increase (as an adverse event e.g. increased DBP of ≥ 5 mm to above the 95^th^ percentile)  Increased pulse values (as an adverse event e.g. range from 25 to 110) |
| Torrioli et al., 2008^119^ | **Eligibility criteria**  Boys aged between 6-13 years; comorbid fragile X syndrome with ADHD. Exclusion criteria: patients had disorders of the central nervous system other than fragile x syndrome, excluding epilepsy (in fact, three had a seizure disorder and received antiepileptic medication); a malignancy or suffered from cardiovascular, renal, or other medical conditions that would compromise their safety or ability to comply with the protocol; head trauma within the previous 12 months; malabsorption; physical or mental disability was so severe to compromise the administration and evaluation of the tests; known hypersensitivity to or had been treated with carnitine or its derivatives during the last 3 months; treated with drugs active on the central nervous system (stimulants, tranquillizers, hypnotics), excluding antiepileptic drugs  **Diagnostic criteria**  DSM-IV  **Number randomized**  63  **Age**  6-13 years (range)  **Co-morbidities/Co-medications**  Yes, all comorbid X fragile syndrome (100%), seizure disorders/epilepsy (5%) – Not receiving CNS drugs (stimulants, tranquillizers, hypnotics)  **ADHD subtypes**  Not reported | **Arm 1**  L-CARN: l-carnitine 500 mg (twice)/day  **Arm 2**  Placebo: placebo pill | **Treatment response** (as a dichotomous outcome)  Not reported/data not abstractable  **All-cause treatment discontinuation** (as a dichotomous outcome)  Yes, reported as study dropp-outs  **Tolerability** (as a dichotomous outcome)  Not reported  **Serious adverse events** (as dichotomous outcome)  Not reported  **Specific adverse events** (as dichotomous outcome)  Not reported |
| Vaisman et al., 2008^120^ | **Eligibility criteria**  Children aged 8-13; had received a previous diagnosis of ADHD by a clinical psychiatrist,  neurologist, or pediatrician. Children with significant sensory or neurological limitations, epilepsy, mental retardation, psychosis, or pervasive developmental disorder were excluded.  Also excluded were children taking medications with known central nervous system effects, including stimulants or dietary supplements other than vitamins  **Diagnostic criteria**  DSM-IV (diagnosis of ADHD by a clinical psychiatrist, neurologist or pediatrician)  **Number randomized**  83  **Age**  8-13 years (range)  **Co-morbidities/Co-medications**  Not reported/No  **ADHD subtypes**  Not reported | **Arm 1**  PUFA: polyunsaturated fatty acid (omega-3 fatty acid: 250 mg/day eicosapentaenoic acid and docosahexaenoic acid)  **Arm 2**  Control: fish oil  **Arm 3**  Placebo: placebo pill | **Treatment response** (as a dichotomous outcome)  Not reported/data not abstractable  **All-cause treatment discontinuation** (as a dichotomous outcome)  Yes, reported as study withdrawls  **Tolerability** (as a dichotomous outcome)  Yes, reported as study withdrawals due to AEs  **Serious adverse events** (as dichotomous outcome)  Not reported  **Specific adverse events** (as dichotomous outcome)  Not reported |
| Weber et al., 2008^121^ | **Eligibility criteria**  Children and adolescents aged 6-17; participants  scored more than 1.5 SDs above age and sex norms on the ADHD RS-IV; parents and participants could read the consent and assent forms in written English; parents and participants were able to attend all study visits; and participants were capable of swallowing pills. Children with severe depression or an active suicide plan, a history or current diagnosis of bipolar disorder or severe conduct disorder, or psychotic symptoms were excluded from the trial  **Diagnostic criteria**  DSM-IV  **Number randomized**  83  **Age**  6-17 years (range)  **Co-morbidities/Co-medications**  Yes, oppositional disorders (44%)/No  **ADHD subtypes**  Not reported | **Arm 1**  Herbal therapy (HYP): St John’s Wort *(Hypericum Perforatum* standardized to 0.3% hypericin) 600 mg/day  **Arm 2**  Placebo: placebo pill | **Treatment response** (as a dichotomous outcome)  Global improvement based on Clinical Global Impression Improvement (CGI-I) scale. Subjects with improvement of “very much” or “much” improved were considered responders (rating evaluations reported by clinician)  **All-cause treatment discontinuation** (as a dichotomous outcome)  Yes, reported as study withdrawls  **Tolerability** (as a dichotomous outcome)  Yes, reported as study withdrawals due to AEs  **Serious adverse events** (as dichotomous outcome)  Not reported  **Specific adverse events** (as dichotomous outcome)  Not reported |
| Arabgol et al., 2009^122^ | **Eligibility criteria**  Children aged 7–16 years. Exclusion criteria included the presence of any physical disease, any comorbid psychiatric disorders (for example: mood disorders, anxiety  disorders, tic disorders, conduct disorder, alcohol  or drug abuses), and mental retardation (IQ < 70). All subjects were drug free for at least 2 weeks prior to the study and did not receive any other pharmacotherapy or psychotherapy simultaneously  **Diagnostic criteria**  DSM-IV-TR  **Number randomized**  33  **Age**  7-16 years (range)  **Co-morbidities/Co-medications**  None/Not reported  **ADHD subtypes**  Combined (88%), inattentive (8%), hyperactive/impulsive (4%) | **Arm 1**  MPH-SA high dose: methylphenidate 20-50 mg/day (mean dose: 32.3 mg/day)  **Arm 2**  REBOX: reboxetine 4-6 mg/day | **Treatment response** (as a dichotomous outcome)  Responders based on the achievement of a 40% or greater decrease from baseline to endpoint in the ADHD-RS total score (rater: parents)  **All-cause treatment discontinuation** (as a dichotomous outcome)  Yes, reported as study discontinuation  **Tolerability** (as a dichotomous outcome)  Yes, reported as study discontinuation due to AEs  **Serious adverse events** (as dichotomous outcome)  Yes, reported  **Specific adverse events** (as dichotomous outcome)  Loss of appetite/anorexia (as an adverse event)  Insomnia (as an adverse event) |
| Block et al., 2009^123,124^ | **Eligibility criteria**  Children aged 6 to 12 years; a symptom severity threshold with scores at least 1.5 SD  above age and gender norms on the ADHD-RS IV–Parent Version. Exclusion criteria included serious  medical illness, a history of psychosis or bipolar  disorder, weight <20 kg or >65 kg at visit 1, uncontrolled hypertension, previous nonresponse to an adequate trial of ATX, intolerable side effects  while receiving ATX, alcohol or drug abuse within the past 3 months, and ongoing use of psychoactive medications other than the study drug  **Diagnostic criteria**  DSM-IV  **Number randomized**  288  **Age**  6-12 years (range)  **Co-morbidities/Co-medications**  Yes, oppositional disorder (32%) and depression (3%)– Not reported  **ADHD subtypes**  Combined (75%), inattentive (22%), hyperactive/impulsive (2%) | **Arm 1**  ATX: atomoxetine 1.25 mg/kg/day  **Arm 2**  Placebo: placebo pill | **Treatment response** (as a dichotomous outcome)  Responders based on the achievement of a 25% or greater decrease from baseline to endpoint in the ADHD-RS-IV-Parent:Inv total score (rater: parents); Global improvement based on Clinical Global Impression Severy(CGI-S) scale. Subjects with improvement of “very much” or “much” improved were considered responders (rating evaluations reported by clinician)  **All-cause treatment discontinuation** (as a dichotomous outcome)  Yes, reported as study discontinuation  **Tolerability** (as a dichotomous outcome)  Yes, reported as study discontinuations due to AEs  **Serious adverse events** (as dichotomous outcome)  None  **Specific adverse events** (as dichotomous outcome)  Loss of appetite/anorexia (as an adverse event)  Cardiovascular event – Increased heart rate (e.g. ≥ 20 bpm to value of at least 100 bpm)  QT prolongation (e.g.. ≥ 30 to a value of at least 435) |
| Childress et al., 2009^125^ | **Eligibility criteria**  Children aged 6–12 years; patients attending school had to have the same teacher (English or Math) for the entire duration of the study, who was willing and able to spend sufficient time with the patient to make valid weekly assessments; drug naive or not treated with any MPH-related medication during the month before the study; patients receiving psychological or behavioural therapies before the screening visit were considered eligible to participate, provided that therapy had been ongoing for ≥ 3 months with the same therapist; patients had to have academic competence appropriate to their age and the  following subscale total scores on Conners’ ADHD/DSM-IV Scales - Teacher Version: (for boys, baseline scores on Conners’ ADHD/DSM-IV Scales - Teacher Version, Total subscale were required to be 27 for those 6 to 8 years old, 24 for those 9 to 11 years old and 19 for those 12 years old; for girls, respective baseline cutoff scores for the same age groups were 16, 13 and 12). Exclusion criteria were: home-schooled children; any medical condition that interfered with study assessments or that was not stable for ≥ 3 months before screening; clinically significant abnormalities detected during screening; family history of long-QT syndrome, current diagnosis or history of cardiac abnormalities, seizures, psychiatric disorders such as schizophrenia, schizoaffective  disorder, severe obsessive-compulsive disorder, conduct disorder, autism, chronic tic  disorder, Tourette’s disorder or any mood or anxiety disorder; antidepressants, antipsychotics, herbal preparations with psychotropic effects,  amphetamine-based medications, benzodiazepines, barbiturates, sedatives or hypnotics, monoamine oxidase inhibitors and atomoxetine had to be stopped 1 to 4 weeks before randomisation according to their half-lives. All concomitant medications that could interfere with absorption, metabolism and distribution of study drug were excluded from the start of screening until the end of all evaluations. Over the-counter analgesics, short-term antibiotic treatment for minor infections and any medication needed to treat adverse events were allowed. Additionally, patients who were judged by the investigator as likely to be noncompliant with study procedures, including those with a suspected history of substance abuse, or those living with a person diagnosed with a substance abuse disorder or whose parent or guardian was unable or unwilling to complete the Conners’ ADHD DSM-IV Scales for parents (CADS-P) were also excluded  **Diagnostic criteria**  DSM-IV  **Number randomized**  253  **Age**  6-12 years (range)  **Co-morbidities/Co-medications**  Yes, respiratory, thoracic and mediastinal disorders (MPH 27%,), immune system disorders (23%), nervous system disorders (19%), surgical and medical procedures (15%), infections and infestations (14%) and skin and subcutaneous tissue disorders (MPH 11%) – Yes (31% prior stimulant exposure)/Yes, ≥ 1 concomitant medication or non-drug therapy after start of study (MPH 39%, placebo 44%).The most common concomitant medications were analgesics, antihistamines and allergy medications  **ADHD subtypes**  Combined (74%), inattentive (28%), hyperactive/impulsive (2%) | **Arm 1**  MPH-LA low/medium dose: dexmethylphenidate extended-release 10-30 mg/day  **Arm 2**  Placebo: placebo pill | **Treatment response** (as a dichotomous outcome)  Clinician-rated Clinical Global Impression Improvement (CGI-I) scale – e.g. responder considered very much improved or much improved (CGI-I ≤ 2); Clinician-rated Clinical Global Impression Severy (CGI-S) scale – e.g. GGI-S ≤ 3  **All-cause treatment discontinuation** (as a dichotomous outcome)  Yes, reported as study discontinuation  **Tolerability** (as a dichotomous outcome)  Yes, reported as study discontinuation due to AEs  **Serious adverse events** (as dichotomous outcome)  None  **Specific adverse events** (as dichotomous outcome)  Loss of appetite/anorexia (as an adverse event)  Decreased weight (as an adverse event)  Insomnia (as an adverse event)  Anxiety (as an advser event)  Cardiovascular event – blood pressure increase (as an adverse event e.g. increased DBP)  Increased pulse values (as an adverse event)  ECG abnormal (as an adverse event)  QT prolongation (as an advserse event) |
| Dell'Agnello et al., 2009^126^ | **Eligibility criteria**  Children aged 6 to 15 years; with ADHD and ODD diagnosed according to the DSM-IV criteri; a score  of at least 1.5 SD above the age norm for the ADHD subscale of the SNAP-IV, a CGI-S ≥4 at both screening and baseline, a SNAP-IV ODD subscale score of at least 15, and a normal intelligence, i.e. a score of IQ ≥70. Exclusion criteria were: body weight <20 kg; history of bipolar I or II disorder, or history of psychosis or pervasive development disorder; history of any seizure disorder (other than febrile seizures) or past/concomitant intake of anticonvulsants for seizure control; serious risk of suicide; history of severe drug allergies; current or past (within 3 months) alcohol or drug abuse; clinically significant cardiovascular disease (including hypertension) or other conditions that could be worsened by an increased heart rate or increased blood pressure; clinically significant  laboratory or ECG abnormalities; medical conditions likely to increase sympathetic nervous system activity or regular intake of sympathomimetic drugs; narrow-angle glaucoma; uncontrolled thyroid dysfunction; likelihood of start of structured psychotherapy at any time during the study; pregnant or breastfeeding females, or females at risk of pregnancy  **Diagnostic criteria**  DSM-IV  **Number randomized**  139  **Age**  6-15 years (range)  **Co-morbidities/Co-medications**  Yes, all comorbid oppositional disorders (100%), anxiety (11%), depression/dysthymia (8%), specific phobias (8%), obsessive-compulsive disorder (2%) – Yes (18% prior ADHD therapy)  **ADHD subtypes**  Combined (89%), inattentive (6%), hyperactive/impulsive (5%) | **Arm 1**  ATX: atomoxetine up to 1.2 mg/kg/day  **Arm 2**  Placebo: placebo pill | **Treatment response** (as a dichotomous outcome)  Responders based on the achievement of a 30% or greater decrease from baseline to endpoint in the SNAP-IV scale (rater: parents); Global improvement based on Clinical Global Impression Severy(CGI-S) scale. Subjects with improvement of “very much” or “much” improved were considered responders (rating evaluations reported by clinician)  **All-cause treatment discontinuation** (as a dichotomous outcome)  Yes, reported as study discontinuation  **Tolerability** (as a dichotomous outcome)  Yes, reported as study discontinuations due to AEs  **Serious adverse events** (as dichotomous outcome)  None  **Specific adverse events** (as dichotomous outcome)  Loss of appetite/anorexia (as an adverse event)  Weight decreased (as an adverse event)  Insomnia (as an adverse event) |
| Johnson et al., 2009^127^ | **Eligibility criteria**  Children and adolescents aged 8-18 years; scoring at least 1.5 *SD* above the age norm for their diagnostic subtype using norms for ADHD-RS-IV. Exclusion criteria were: autism (however, autistic symptoms [AS] diagnosed in cases meeting three or more but not full symptom criteria for a diagnosis of autistic disorder, Asperger syndrome, or any of the other autism spectrum disorders was not an exclusion criterion), psychosis, bipolar disorder, mental retardation, uncontrolled seizure disorder, hyper- or hypothyroidism, significant other medical conditions, weight below 20 kg, alcohol or drug abuse, or the use of any psychoactive drugs or omega 3 preparations in the past 3 months  **Diagnostic criteria**  DSM-IV  **Number randomized**  75  **Age**  8-18 years (range)  **Co-morbidities/Co-medications**  Yes, reading/writing disorders (43%), oppositional disorders (24%), Austisme-like condition or Asperger (15%), learning disability (11%), depression or anxiety (8%), specific phobias (8%), obsessive-compulsive disorder (2%) – Not reported  **ADHD subtypes**  Combined (47%), inattentive (40%), hyperactive/impulsive (-%) | **Arm 1**  PUFA: polyunsaturated fatty acid (omega-3/6 fatty acid: 558 mg/day eicosapentaenoic acid and 174 mg/day docosahexaenoic acid; 60 mg gama linoleic acid; 10.8 mg vitamin E)  **Arm 2**  Placebo: olive oil | **Treatment response** (as a dichotomous outcome)  Responders based on the achievement of a 30% greater decrease from baseline to endpoint in the ADHD-RS (rater: clinician)  **All-cause treatment discontinuation** (as a dichotomous outcome)  Yes, reported as study discontinuation  **Tolerability** (as a dichotomous outcome)  Yes, reported as study discontinuations due to AEs  **Serious adverse events** (as dichotomous outcome)  None  **Specific adverse events** (as dichotomous outcome)  Not reported |
| Kahbazi et al., 2009^128^ | **Eligibility criteria**  Children aged 6–15 years; total and/or subscale scores on ADHD-RS-IV School Version at least 1.5 SD above norms for patient's age and gender. All patients had combined subtype of ADHD and were newly diagnosed. Children were excluded if  they had a history or current diagnosis of pervasive developmental disorders, schizophrenia or other psychiatric disorders (DSM-IV axis I); any current psychiatric comorbidity that required pharmacotherapy; any evidence of suicide risk and mental retardation (IQ<70 based on clinical judgment). In addition, patients were excluded if they had a clinically significant chronic medical condition, including organic brain disorder, seizures and, current abuse or dependence on drugs within 6 months. Additional exclusion criteria were hypertension, hypotension and habitual consumption of more than 250 mg/day of caffeine  **Diagnostic criteria**  DSM-IV-TR  **Number randomized**  46  **Age**  6-15 years (range)  **Co-morbidities/Co-medications**  Not reported/Not reported  **ADHD subtypes**  Combined (100%) | **Arm 1**  MODAF: modafinil 200-300 mg/day  **Arm 2**  Placebo: placebo pill | **Treatment response** (as a dichotomous outcome)  Responders based on the achievement of a 40% or greater decrease from baseline to endpoint in the ADHD-RS total score (rater: parents and teachers)  **All-cause treatment discontinuation** (as a dichotomous outcome)  Yes, reported as study discontinuation  **Tolerability** (as a dichotomous outcome)  Not reported  **Serious adverse events** (as dichotomous outcome)  Not reported  **Specific adverse events** (as dichotomous outcome)  Loss of appetite/anorexia (as an adverse event)  Decreased weight (as an adverse event e.g. weight loss)  Anxiety (as an adverse event e.g. anxiety/nervousness)  Sleep disturbances (as an adverse event e.g. difficulty falling asleep) |
| Montoya et al., 2009^129,130^ | **Eligibility criteria**  Children aged 6 to 15 years; newly diagnosed (time since diagnosis ≤ 3 months), treatment-naïve cases of ADHD defined according to the criteria of the revised ADHDRS-IVParent:Inv total score ≥1.5 SD above the age norm for their diagnostic subtype. Exclusion criteria were: bipolar disorder, psychosis, pervasive developmental disorder or seizure disorder, glaucoma or hypertension, IQ < 70, any pervasive developmental disorder, alcohol or drug abuse within the past 3 months, planned start of structured psychotherapy at any time during the study, and taking any regular psychoactive or sympathomimetic medication  **Diagnostic criteria**  DSM-IV-TR  **Number randomized**  151  **Age**  6-15 years (range)  **Co-morbidities/Co-medications**  Yes, oppositional disorder (26%), anxiety (13%), tic disorders (17%), affective disorders (3%) – No  **ADHD subtypes**  Combined (63%), inattentive (33%), hyperactive/impulsive (4%) | **Arm 1**  ATX: atomoxetine 1.2 mg/kg/day  **Arm 2**  Placebo: placebo pill | **Treatment response** (as a dichotomous outcome)  Responders based on the achievement of a 25% or greater decrease from baseline to endpoint in the ADHD-RS-IV-Parent:Inv total score (rater: parents); Global improvement based on Clinical Global Impression Severy (CGI-S) scale. Subjects with improvement of “very much” or “much” improved were considered responders (rating evaluations reported by clinician) e.g. CGI-S ≤ 3  **All-cause treatment discontinuation** (as a dichotomous outcome)  Yes, reported as study discontinuation  **Tolerability** (as a dichotomous outcome)  Not reported  **Serious adverse events** (as dichotomous outcome)  Yes, reported  **Specific adverse events** (as dichotomous outcome)  Loss of appetite/anorexia (as an adverse event)  Cardiovascular event – tachycardia (as an adverse event) |
| Nair et al., 2009^131^ | **Eligibility criteria**  Children aged 4-12 years. Children with history of  sensitivity to drug testing and those children suffering from other organic disorders were excluded from the study. All the parents of the affected children were informed about the availability of the psychostimulant drugs and their efficacy. Those parents who could not afford pychostimulant drugs were explained about the side effects of clonidine and carbamazepine and informed consent were obtained from them  **Diagnostic criteria**  DSM-IV-TR  **Number randomized**  50  **Age**  4-12 years (range)  **Co-morbidities/Co-medications**  Yes, oppositional disorders (10%), conduct disorders (15%), seizures (13%)/Not reported  **ADHD subtypes**  Combined (55%) | **Arm 1**  CLON-SA: clonidine 8 µg/kg/day  **Arm 2**  CARBA: carbamazepine (dose unknown) | **Treatment response** (as a dichotomous outcome)  Responders based on the achievement of a 25% or greater decrease from baseline to endpoint in the ADHD-RS total score (rater: unclear, but assumed the clinicians/invesigators)  **All-cause treatment discontinuation** (as a dichotomous outcome)  Yes, reported as study discontinuation  **Tolerability** (as a dichotomous outcome)  Not reported  **Serious adverse events** (as dichotomous outcome)  Not reported  **Specific adverse events** (as dichotomous outcome)  Not reported |
| Pelsser et al., 2009^132^ | **Eligibility criteria**  Children 3.8 and 8.5 years old and they all  met the criteria for ADHD Combined Type or Predominantly Hyperactive-Impulsive Type. Exclusion criteria were: adopted or foster children, co-existing neurological diseases, an IQ<70, prematurity or dysmaturity, use of alcohol, or smoking by mother during pregnancy, and co-existence of other psychiatric disorders, except for oppositional defiant disorder and conduct disorder (CD). The screening involved a systematic  and complete review of the symptoms and diagnostic criteria of all DSM-IV axis I disorders occurring in childhood. None of the children used psychotropic medication  **Diagnostic criteria**  DSM-IV  **Number randomized**  27  **Age**  3.8-8.5 years (range)  **Co-morbidities/Co-medications**  Yes, oppositional disorders (82%)/On dietary restriction (8% in waiting list group)  **ADHD subtypes**  Combined (67%), inattentive (-%), hyperactive/impulsive (33%) | **Arm 1**  Restricted elimination diet (elimination diet): during the baseline diet the parents kept an extended diary in order to enable an assessment of the child’s normal diet, its behaviour and activities. After the baseline diet and the second assessment, the intervention group started with an individually composed elimination diet, which had to be followed for a period of 5 weeks. The elimination diet consisted of rice, turkey, lamb, vegetables, fruits, margarine, vegetable oil, tea, pear juice and water  **Arm 2**  Waiting list (WL): the control group was placed on a waiting list and continued their own, freely chosen diet. At the start of the trial the parents of the control group were informed that they could start with the elimination diet immediately after the last assessment if they so wished | **Treatment response** (as a dichotomous outcome)  Responders based on the achievement of a 50% or greater decrease from baseline to endpoint in the ADHD-RS total score (rater: parent); the achievement of a 50% or greater decrease from baseline to endpoint in the abbreviated Conners’ Rating Scale (rater: parents and teachers)  **All-cause treatment discontinuation** (as a dichotomous outcome)  Yes, reported as study dropp-outs  **Tolerability** (as a dichotomous outcome)  Yes, reported as study dropp-outs due to illness  **Serious adverse events** (as dichotomous outcome)  Not reported  **Specific adverse events** (as dichotomous outcome)  Not reported |
| Raz et al., 2009^133^ | **Eligibility criteria**  Children and adolescents aged 7-13 years; a written ADHD diagnosis from a child psychiatrist, a neurologist, a pediatrician, or a clinical psychologist. (This diagnosis was made using the standard clinical practice, and not as a part of the trial or by our staff; it was not based on the assessment in the trial.) Exclusion criteria were: use of medications for ADHD during the past month, use of PUFA supplements during the past 3 months, or the presence of at least one of the following co-morbidities—pervasive developmental disorder, seizure disorder, schizophrenia, major depression, or bipolar disorder  **Diagnostic criteria**  DSM-IV  **Number randomized**  78  **Age**  7-13 years (range)  **Co-morbidities/Co-medications**  Yes, learning disability (35%), sleep disorders (8%), dyspraxia (8%), oppositional disorders (5%), anxiety (5%), tic disorders (3%), obsessive-compulsive disorder (3%), conduct disorders (2%)/ Not reported  **ADHD subtypes**  Inattentive ‘factor’ (93%), hyperactive/impulsive ‘factor’ (93%) | **Arm 1**  PUFA: polyunsaturated fatty acid (omega-3/6 fatty acid: 240 mg linoleic acid, 60 mg alpha-linoleic acid, 95 mg mineral oil and 5 mg alpha-tocopherol twice/daily)  **Arm 2**  Placebo: placebo pill containing 500 mg ascorbic acid, twice daily | **Treatment response** (as a dichotomous outcome)  Not reported/data not abstractable  **All-cause treatment discontinuation** (as a dichotomous outcome)  Yes, reported as study discontinuation  **Tolerability** (as a dichotomous outcome)  Yes, reported as study discontinuations due to AEs  **Serious adverse events** (as dichotomous outcome)  None  **Specific adverse events** (as dichotomous outcome)  Sleep disturbances (as an adverse event) |
| Sallee et al., 2009^134-136^ | **Eligibility criteria**  Children and adolescent aged 6-17 years; a minimum baseline score of 24 on the ADHD-RS. Exclusion criteria were: any current severe Axis I or  Axis II disorders or any other current uncontrolled comorbid psychiatric diagnosis (excluding oppositional defiant disorder), weight of less than 55 lb (25 kg), morbid obesity (body mass index  ≥35), current use of medications that affect blood pressure (BP) or heart rate (except for ADHD therapies, which were discontinued during the washout period), hypertension or orthostatic hypotension, abnormal electrocardiogram or vital signs, previous treatment of ADHD with GUAN-LA, or intolerance of GUAN  **Diagnostic criteria**  DSM-IV  **Number randomized**  324  **Age**  6-17 years (range)  **Co-morbidities/Co-medications**  Yes, oppositional disorders (6%)/Not reported  **ADHD subtypes**  Combined (73%), inattentive (26%), hyperactive/impulsive (2%) | **Arm 1**  GUAN-LA: guanfacine long acting 1, 2, 3 or 4 mg/day  **Arm 2**  Placebo: placebo pill | **Treatment response** (as a dichotomous outcome)  Global improvement based on Clinical Global Impression Improvement (CGI-I) scale. Subjects with improvement of “very much” or “much” improved were considered responders (rating evaluations reported by clinician);  Global improvement in Parent Global Assessment (PGA). Subjects with improvement of “very much” or “much” improved were considered responders (rating evaluations reported by parents)  **All-cause treatment discontinuation** (as a dichotomous outcome)  Yes, reported as study withdrawals  **Tolerability** (as a dichotomous outcome)  Yes, reported as study withdrawals due tu AEs  **Serious adverse events** (as dichotomous outcome)  Yes, reported  **Specific adverse events** (as dichotomous outcome)  Loss of appetite/anorexia (as an adverse event leading to discontinuation)  Anxiety (as an adverse event leading to discontinuation)  Cardiovascular event – hypotension (as an adverse event)  Increased pulse values (as an adverse event e.g. 100 bpm or greater)  Decreased pulse values (as an adverse event e.g. 50 bpm or lesser)  ECG abnormal (as an adverse event)  QT prolongation (as an advserse event) |
| Svanborg et al., 2009^137,138^ | **Eligibility criteria**  Children aged 7-15; had a severity threshold of 1.5 SD above the US age and gender norms for their diagnostic subtype on the ADHD RS parent  version: Investigator Administered and Scored. Eligible patients had to be stimulant-naïve.  Exclusion criteria were: general impairment of intelligence, as clinically assessed by the investigator, serious medical illness, a history of psychosis or bipolar disorder, alcohol or drug abuse within the previous 3 months, or ongoing use of psychoactive medication other than the study drug. Patients who required immediate pharmacotherapy or structured psychotherapy were also excluded  **Diagnostic criteria**  DSM-IV  **Number randomized**  99  **Age**  7-15 years (range)  **Co-morbidities/Co-medications**  Yes, oppositional disorder (20%), tic disorders (14%), depression (5%) – Not reported  **ADHD subtypes**  Combined (78%), inattentive (4%), hyperactive/impulsive (18%) | **Arm 1**  ATX+BT (child and parent training): combination of both atomoxetine (1.2 mg/day) and behavioral therapy including parent and child training (described below)  **Arm 2**  BT (child and parent training): BT included a psychoeducational program for the patients’ caregivers of both treatment groups. This program, developed by a professional educational manager, consisted of four 3-h sessions and was specifically adapted to the conditions associated with this clinical study in children and adolescents. It aimed to improve the caregivers’ knowledge and understanding of ADHD and the implications of the disorder on the child’s everyday functioning, to increase the caregivers’ awareness of how they can adjust the environment to the child’s functional level, to provide guidance on how to facilitate and promote positive parent-child interaction and positive behavioral patterns, to give an introduction on how behavior modification principles can be applied in handling the child’s problem behavior, and to guide and inform about available social and educational support and health care. Methods included introductory lectures, group discussions, problem solving, modeling, and role plays based on real life situations. Each session was conducted by one or two group leaders with various professional backgrounds (psychologists, social workers, special educators, and psychiatric nurses), who had broad experience with ADHD patients and were familiar with psychosocial treatment based on behavioral principles. All group leaders had to participate in a specific 2-day training supervised by the educational manager before the start of the trial. All information provided in slides and printed material was standardized, and all sites used the same educational material. After the last session, the caregivers received an evaluation form for assessment of the psychoeducational program | **Treatment response** (as a dichotomous outcome)  Responders based on the achievement of a 25% or greater decrease from baseline to endpoint in the ADHD-RS total score (rater: clinician);  **All-cause treatment discontinuation** (as a dichotomous outcome)  Not reported  **Tolerability** (as a dichotomous outcome)  Not reported  **Serious adverse events** (as dichotomous outcome)  Not reported  **Specific adverse events** (as dichotomous outcome)  Loss of appetite/anorexia (as an adverse event) |
| Takahashi et al., 2009^139,140^ | **Eligibility criteria**  Children and adolescents aged 6-17 years; Clinical Global Impressions–ADHD-Severity (CGI-ADHD-S)  assessment score ≤3 and a symptom severity score at least 1.5 SD above Japanese pediatric age and gender norms on the ADHD-RS-IV–Parent Version:Investigator Administered and Scored=Translated and Validated in Japanese; normal intelligence (IQ≥80). For patients younger than 17 years of age, this was assessed by the Wechsler Intelligence Scale for Children–Third Edition (WISC-III). Important exclusion criteria included patients who took any antipsychotic medication within 26 weeks of study visit 1, had a history of bipolar disorder or psychosis, or were determined by the investigator to be at suicidal risk  **Diagnostic criteria**  DSM-IV  **Number randomized**  245  **Age**  6-17 years (range)  **Co-morbidities/Co-medications**  Yes, oppositional disorder (14%), conduct disorder (1%)/Not reported  **ADHD subtypes**  Combined (34%), Inattentive (61%), Hyperactive/impulsive (5%) | **Arm 1**  ATX: 3 doses of atomoxetine (0.5, 1.2 or 1.8 mg/kg/day)  **Arm 2**  Placebo: placebo pill | **Treatment response** (as a dichotomous outcome)  Responders based on the achievement of a 25% or greater decrease from baseline to endpoint in the ADHD-RS-IV total score (rater: clinician)  **All-cause treatment discontinuation** (as a dichotomous outcome)  Yes, reported as study discontinuation  **Tolerability** (as a dichotomous outcome)  Yes, reported as study discontinuation due to AEs  **Serious adverse events** (as dichotomous outcome)  Yes, reported  **Specific adverse events** (as dichotomous outcome)  Loss of appetite/anorexia (as an adverse event)  Decreased weight (significant weight loss e.g. ≥ 3.5% of body weight loss)  Cardiovascular event – blood pressure increase (as an adverse event e.g. increased DBP)  Decreased pulse or heart rate values (as an adverse event e.g. 50 bpm or lesser)  QT prolongation (as an advserse event) |
| Perez-Alvarez et al., 2009^141^ | **Eligibility criteria**  Children aged 7–15 years; ADHD combined or inattentive subtypes; IQ > 70. Exclusion criteria were: previous medication or therapy; comborbidity  **Diagnostic criteria**  DSM-IV-TR  **Number randomized**  150  **Age**  7-14 years (range)  **Co-morbidities/Co-medications**  Not reported/Not reported  **ADHD subtypes**  Combined (67%), inattentive (33%), hyperactive/impulsive (0%) | **Arm 1**  MPH-LA any dose: methylphenidate extended-release (OROS®) dose unspecified  **Arm 2**  HPT: humanistic psychotherapy. The humanistic psychotherapy has to do with Rogerian psychotherapy  (person-centered therapy), Ericksonian hypnosis, and  family-systemic psychotherapy. Trained psychotherapists (qualified in psychology) performed the procedure consisting of 24 sessions, 1 every 15 days  **Arm 3**  MPH-LA any dose+HPT: combination of methylphenidate extended-release and humanistic psychotherapy | **Treatment response** (as a dichotomous outcome)  Improvement in the SNAP rating scale ≤ 1, by parents/teachers  **All-cause treatment discontinuation** (as a dichotomous outcome)  None  **Tolerability** (as a dichotomous outcome)  None  **Serious adverse events** (as dichotomous outcome)  Not reported  **Specific adverse events** (as dichotomous outcome)  Not reported |
| Thompson et al., 2009^142^ | **Eligibility criteria**  Preschool children aged 2-6; parents who were concerned about their children’s ADHD behavior; no child entering the trial had been on medication; no child received medication during the trial or the follow-up period; families had to be fluent in English; able to commit to the length of the trial including the follow-up period, and were willing to be seen at home. Families were excluded from the trial if they had previously or were currently attending the local child and adolescent services, if the mother was known to have a severe mental illness or if the child had a pervasive developmental disorder, severe receptive language impairment, neurological disorder or was on the social services register for a current history of child sexual or physical abuse. The information about exclusion criteria was obtained directly from the mother and/or the referral source (e.g., health visitor)  **Diagnostic criteria**  DSM-IV/PACS (but a formal diagnosis was  not made in the current trial as the PACS diagnosis has not been validated for preschool children)  **Number randomized**  41  **Age**  2-6 years (range)  **Co-morbidities/Co-medications**  Not reported/Not reported  **ADHD subtypes**  Not reported | **Arm 1**  BT (parent training): Two part-time nurses were employed to deliver the intervention and were trained in the revised new forest parenting programme (NFPP) program by two core members of the program development team. Weekly telephone and email supervision was supported with monthly visits to supervise the therapists on a face-to-face basis for the first 6 months and then every 2 months for the last 7 months during the intervention phase. All therapy sessions were audio-taped and these tapes were used for supervision sessions to ensure that the intervention was delivered as planned. The integrity of ongoing treatment delivery was reviewed using checklists completed by the therapists and the clinical supervisors independently. The therapists also kept reflective diaries. The reflective diaries were used by the therapists to review the sessions and give a view on whether the families were responding to the program and to help plan the delivery of the following  week  **Arm 2**  Standard care (control): Treatment as usual (TAU) was intended to control for the effects of time in treatment and to compare NFPP treatment effects with the potential impact of interventions typically provided by community-based practitioners on children’s and parents’ functioning during the course of treatment and follow-up. TAU participants received no treatment  from study staff, nor were they referred onto services, but were given contact information for Health Visitors, general practitioners or school nurses which they could use as they wished | **Treatment response** (as a dichotomous outcome)  Improvement in the PACS rating scale (rated by parents), treatment success was judged against a threshold for clinical change of a decrease in PACS ADHD symptoms of five points  **All-cause treatment discontinuation** (as a dichotomous outcome)  Yes, reported as study drop-outs  **Tolerability** (as a dichotomous outcome)  Not reported  **Serious adverse events** (as dichotomous outcome)  Not reported  **Specific adverse events** (as dichotomous outcome)  Not reported |
| Tramontina et al., 2009^143^ | **Eligibility criteria**  Children aged 8-17 years; bipolar I or II disorder comorbid with ADHD; clear reports of ADHD symptom onset preceding any mood symptomatology; acute manic or mixed state. Exclusion criteria were: IQ < 70; use of any medication 4 weeks prior to entering the study; pervasive developmental disorder, schizophrenia or substance abuse or dependence; severe suicide/homicide risk contraindicating outpatient treatment; previous use of aripiprazole; any other acute or chronic disease that might interfere in the study; or pregnancy  **Diagnostic criteria**  DSM-IV  **Number randomized**  43  **Age**  8-17 years (range)  **Co-morbidities/Co-medications**  Yes, all comorbid bipolar disorder (100%), disruptive behavioral disorders (81%), anxiety (49%), psychosis (37%) – No  **ADHD subtypes**  Combined (79%) | **Arm 1**  ARIP: aripiprazole 5-20 mg/day (mean dose: 13.6 mg/day)  **Arm 2**  Placebo: placebo pill | **Treatment response** (as a dichotomous outcome)  Not reported/data not abstractable  **All-cause treatment discontinuation** (as a dichotomous outcome)  Yes, reported as study withdrawals  **Tolerability** (as a dichotomous outcome)  Yes, reported as study withdrawals due to AEs  **Serious adverse events** (as dichotomous outcome)  Not reported  **Specific adverse events** (as dichotomous outcome)  Not reported |
| Tucker et al., 2009^144^ | **Eligibility criteria**  Children aged 6-12; had age-appropriate cognitive functioning. Study participants were excluded if they had: previous exposure to MPH or any amphetamine-basedmedication, a positive urine drug screen, an abnormality in the screening assessment (physical exam, vital signs, laboratory tests) thatwas judged to be clinically significant, a cardiac abnormality, a history of seizures or schizophrenia, or a current diagnosis of mood disorder or anxiety disorder  **Diagnostic criteria**  DSM-IV  **Number randomized**  109  **Age**  6-12 years (range)  **Co-morbidities/Co-medications**  Not reported – Not reported  **ADHD subtypes**  Not reported | **Arm 1**  MPH-LA high dose+BT (child and parent training): combination of methylphenidate extended release (up to 60 mg/day) and behavioral therapy including parent and child training (described below)  **Arm 2**  BT (child and parent training): all patients received BT based on the program of Disruptive Behavior Intervention that was designed both for the children and the parents. Clinicians trained in BT for ADHD implemented this program. In general, the therapy consisted of two parts: Phase I was designed to target key symptoms of the disease (non-compliance, off-tasks, impulse behavior) and Phase II sessions focused on organizational skills, family communication, and discipline. A maximum of 17 sessions were scheduled individually and were evenly spread over the course of the study | **Treatment response** (as a dichotomous outcome)  Not reported/data not abstractable  **All-cause treatment discontinuation** (as a dichotomous outcome)  Yes, reported as study discontinuations  **Tolerability** (as a dichotomous outcome)  Not reported  **Serious adverse events** (as dichotomous outcome)  Not reported  **Specific adverse events** (as dichotomous outcome)  Not reported |
| Gevensleben et al., 2009^145-147^ | **Eligibility criteria**  Children aged 8-12. Children with comorbid disorders other than conduct disorder, emotional disorders, tic disorder and dyslexia were excluded from the study. All children lacked gross neurological or other organic disorders. All children were drug-free and without concurring psychotherapy for at least 6 weeks before starting the training  **Diagnostic criteria**  DSM-IV  **Number randomized**  102  **Age**  8-12 years (range)  **Co-morbidities/Co-medications**  Not reported – Not reported  **ADHD subtypes**  Combined (70%), inattentive (30%) | **Arm 1**  Neurofeedback (theta-beta and slow cortical potential training): the neurofeedback (NF) system SAM (Self-regulation and Attention Management) was used for neurofeedback training. It contains several feedback animations to keep the training diversified and appropriate for children. During training, children sat in front of a monitor and controlled a kind of computer game by modulating their brain electrical activity. In the course of the SCP training the task was to find appropriate strategies to direct a ball upwards (negativity trials) or downwards (positivity trials). In the theta/beta-protocol a bar on the left of the screen (representing theta activity) had to be reduced while simultaneously a bar on the right (representing beta activity) had to be increased. In each SCP training session approximately 120 trials were performed. Negativity (50%) and positivity trials (50%) were presented in random order. A trial lasted for 8 seconds (baseline period: 2 s, feedback period: 6 s). Intertrial interval was set to 5 ± 1 s. Trials of the theta/beta training lasted for 5 minutes at the start of training and were extended to 10 minutes as the training proceeded. Feedback was calculated from Cz (reference: mastoids, bandwidth: 1–30 Hz for theta/beta training and .01–30 Hz for SCP training, respectively, sampling rate: 250 Hz). Baseline values  were determined at the beginning of each session (3 minutes). An adjustment within a session was not  scheduled. Vertical eye movements, which were recorded with electrodes above and below the left eye, were corrected online using slightly different regression- based algorithms for theta/beta training  and SCP training. For segments containing artefacts  exceeding ±100 lV in the EEG channel and ±200 lV in the EOG channel, no feedback was calculated. Transfer trials, i.e., trials without contingent feedback, were also conducted (about 40% at the beginning of a training block and about 60% at the end of a training block). The children of the NF group were required to practice their focused state (which was practised in the  sessions) at home, in different situations (one situation  per day, e.g., ‘try to be very focused while reading’, ‘try to stay focused on the ball while playing football this afternoon’)  **Arm 2**  Cognitive training (attention training): the attention skills training was based on ‘Skillies’, a German learning software, which primarily exercises visual and auditory perception, vigilance, sustained attention, and reactivity. In ‘Skillies’, the children had to sail to several islands. On each island, a defined task – each requiring different attention-based skills – has to be solved; e.g., on an island named ‘Coloured Reef’, fish of different colours swim from one side of the screen to the other and back. All fish must be the same colour. The colour can be modified by clicking on a fish. With every change of direction, the fish change their colour (fixed order). Thus, the main aim of this  task is to improve vigilance and reactivity. The training was complemented by some self-directed interventions from cognitive therapy to assure comparability to NF, i.e., the children were to compile  (meta-)cognitive strategies such as focusing attention,  careful processing of tasks and impulse control.  Corresponding to the NF group, children of the cognitive training group should practise one of the strategies needed to solve a task of the computer-game (‘watch like a hawk’), in daily-life situations (as described in the NF section above) | **Treatment response** (as a dichotomous outcome)  Responders based on the achievement of a > 25% decrease from baseline to endpoint in the German Parent ADHD-RS-IV total score : P-FBB-HKS (rater: parents)  **All-cause treatment discontinuation** (as a dichotomous outcome)  Yes, reported as study discontinuations  **Tolerability** (as a dichotomous outcome)  Not reported  **Serious adverse events** (as dichotomous outcome)  Not reported  **Specific adverse events** (as dichotomous outcome)  Not reported |
| Connor et al., 2010^148,149^ | **Eligibility criteria**  Children and adolescent aged 6-12 years; a baseline score ≥24 on the ADHD_RS-IV and a baseline score ≥14 (males) or ≥12 (females) on the oppositional subscale of the Conners’ Parent Rating Scale- Revised: Long Form (CPRS-R:L). Subjects were excluded for any current co-morbid  psychiatric diagnosis (except oppositional defiant  disorder [ODD], dysthymia or simple phobias),  weight <55 lb (<25 kg), pre-existing cardiovascular complications, or current use of medications that affect the CNS, blood pressure or heart rate (except for ADHD therapies, which were discontinued during the washout period)  **Diagnostic criteria**  DSM-IV-TR  **Number randomized**  217  **Age**  6-12 years (range)  **Co-morbidities/Co-medications**  Yes, all comorbid oppositional disorders (100%)/No  **ADHD subtypes**  Combined (84%), inattentive (13%), hyperactive/impulsive (3%) | **Arm 1**  GUAN-LA: guanfacine long acting 1-4 mg/day  **Arm 2**  Placebo: placebo pill | **Treatment response** (as a dichotomous outcome)  Global improvement based on Clinical Global Impression Improvement (CGI-I) scale. Subjects with improvement of “very much” or “much” improved were considered responders e.g. GGI-I ≤ 2 (rating evaluations reported by clinician; Global improvement based on Clinical Global Impression Severy (CGI-S) scale e.g. CGI-S ≤ 3  **All-cause treatment discontinuation** (as a dichotomous outcome)  Yes, reported as early discontinuations  **Tolerability** (as a dichotomous outcome)  Yes, reported as early discontinuations due tu AEs  **Serious adverse events** (as dichotomous outcome)  None  **Specific adverse events** (as dichotomous outcome)  Cardiovascular event – decreased blood pressure (as an adverse event)  Decreased heart rate (as an adverse event e.g. 50 bpm or lesser)  Synus bradycardia (as an adverse event)  Exctopic supraventricular rhythm (as an adverse event)  QT prolongation (as an advserse event) |
| Fabiano et al., 2010^150^ | **Eligibility criteria**  Children aged 6-12 years; IQ > 79. Not reported exclusion criteria.  **Diagnostic criteria**  DSM-IV  **Number randomized**  63  **Age**  6-12 years (range)  **Co-morbidities/Co-medications**  Yes, oppositional disorder or conduct disorder (88%), learning problems (in special education 100%)– Yes, 53%  **ADHD subtypes**  Combined (87%), inattentive (11%), hyperactive/impulsive (2%) | **Arm 1**  BT (child, parent and teacher training): Behavioral therapy using a daily report card with child, parent and teacher training  **Arm 2**  Standard care (control): usual care | **Treatment response** (as a dichotomous outcome)  Improvement of ADHD symptoms (DBD-ADHD-RS) endpoint teachers rating were within 1 SD of the normative mean  **All-cause treatment discontinuation** (as a dichotomous outcome)  Yes, reported as drop-outs/terminated early  **Tolerability** (as a dichotomous outcome)  Not reported  **Serious adverse events** (as dichotomous outcome)  Not reported  **Specific adverse events** (as dichotomous outcome)  Not reported |
| Findling et al., 2010^151^ | **Eligibility criteria**  Adolescents 13-17 years; ADHD-RS total score ≥ 26 at baseline; ECG results within normal range or variants that were not clinically significant, as judged by investigators in conjunction with the central laboratory; blood pressure measurements within the 95th percentile for age, sex and height; no current or past skin disease or other skin problems, including sensitive skin or  signs of skin irritation; females must have a negative urine pregnancy test at entry and must agree to use acceptable contraceptives throughout the study period and for 30 days the last dose of IP. Exclusion criteria were: conduct disorder or comorbid psychiatric illness (such as clinically significant obsessive-compulsive disorder, depressive or anxiety disorder; post-traumatic stress disorder; psychosis; bipolar illness; or pervasive developmental disorder); history of structural cardiac abnormality, cardiomyopathy, cardiac rhythm abnormalities or other serious cardiac problems; suicidal ideation; alcohol or other substance abuse (except caffeine or nicotine) within the past 6 months; seizures during the previous 2 years and a history of being non-responsive to psychostimulant treatment; use of clonidine, atomoxetine, antidepressants, sedatives, antipsychotics, anxiolytics, P450 enzyme-altering agents or other investigational medications within 30 days before screening; female participant who is pregnant or lactating  **Diagnostic criteria**  DSM-IV-TR  **Number randomized**  217  **Age**  13-17 years (range)  **Co-morbidities/Co-medications**  None/Not reported  **ADHD subtypes**  Not reported | **Arm 1**  MPH-TS: methylphenidate transdermal system (patch) 10-30 mg over 9-h periods  **Arm 2**  Placebo: placebo transdermal system | **Treatment response** (as a dichotomous outcome)  Global improvement based on Clinical Global Impression Improvement (CGI-I) scale. Subjects with improvement of “very much” or “much” improved were considered responders (rating evaluations reported by clinician);  Global improvement in Parent Global Assessment (PGA). Subjects with improvement of “very much” or “much” improved were considered responders (rating evaluations reported by parents)  **All-cause treatment discontinuation** (as a dichotomous outcome)  Yes, reported as study withdrawals  **Tolerability** (as a dichotomous outcome)  Yes, reported as study withdrawals due to AEs  **Serious adverse events** (as dichotomous outcome)  Yes, reported  **Specific adverse events** (as dichotomous outcome)  Loss of appetite/anorexia (as an adverse event)  Decreased weight (as an adverse event)  Insomnia (as an adverse event)  Syncope (as an adverse event)  Cardiovascular event – blood pressure decreased (as an adverse event)  Tachycardia (as an adverse event)  Palpitations (as an adverse event)  Increased heart rate (as an adverse event) |
| Gustafsson et al., 2010^152^ | **Eligibility criteria**  Children and adolescents aged 7-12 years; combined ADHD subtype; with any neuropsychiatric comorbidity. Exclusion criteria were: mental retardation (IQ < 70 on formal psychological testing), autism, major depression, epileptic seizure (including Petit Mal) during the proceeding 2 years, other neurological disorder, other endocrinological disorders (e.g. diabetes mellitus, thyroid disorder, etc.), fish allergy, severely impaired hearing and vision, severe sleeping disorder, psychotic symptoms or other ongoing medication (e.g. psychoactive compounds, anti-convulsants, stimulants). No child had been on stimulants. If the child had taken PUFA, a wash-out period of 10 weeks was required  **Diagnostic criteria**  DSM-IV  **Number randomized**  109  **Age**  7-12 years (range)  **Co-morbidities/Co-medications**  Yes, oppositional disorders (61%), neuromotor problems (48%) – No  **ADHD subtypes**  Combined (100%) | **Arm 1**  PUFA: polyunsaturated fatty acid (omega-3 fatty acid: 500 mg/day eicosapentaenoic acid and 2.7 mg/day docosahexaenoic acid; 10 mg vitamin E)  **Arm 2**  Placebo: placebo pill (rape seed oil and medium-chain triglycerides) | **Treatment response** (as a dichotomous outcome)  Response defined as Conners’ Rating Scale (raters: parents and teachers) total score decrease ≥ 25% from baseline (unpublished outcome data provided by Prof. Gustafsson, correspondence of February 7^th^ 2016)  **All-cause treatment discontinuation** (as a dichotomous outcome)  Yes, reported as study discontinuation  **Tolerability** (as a dichotomous outcome)  Not reported  **Serious adverse events** (as dichotomous outcome)  Not reported  **Specific adverse events** (as dichotomous outcome)  Not reported |
| Martenyi et al., 2010^153,154^ | **Eligibility criteria**  Children aged 6 to 16 years; had a minimum score of 25 for boys and 22 for girls, or >12 for their diagnostic subtype on the ADHD-RS Parent Version  as well as a score of ≥4 on the CGI-ADHD-S scale; had not taken any medication for the treatment of ADHD or completed washout procedures; had no significant abnormalities in laboratory results and baseline ECG; and were able to communicate suitably with the investigator and study coordinator. Exclusion criteria: weight <20 kg or  >60 kg at study entry; experienced no clinical benefit after an adequate trial with MPH or AMPH  (all patients were psychostimulant naïve, but it was not required by the protocol); had been treated, within the previous 30 days, with a drug (not including study drug) that had not received a regulatory approval for any indication at the time of study entry; had a history of bipolar I or II disorder, psychosis, or pervasive developmental disorder; met DSM-IV criteria for an anxiety disorder; had a history of any seizure disorder (other than febrile seizures) or prior electroencephalogram abnormalities related to epilepsy; had taken (or were taking) anticonvulsants for seizure control; were at serious suicidal risk or had a serious medical illness; or were pregnant or breast-feeding. Sexually active female patients had to use a medically acceptable method of contraception. Female patients of child-bearing potential, who were abstinent, were allowed to enter the study, provided they agreed that if they became sexually active, they would use a medically acceptable method of contraception  **Diagnostic criteria**  DSM-IV  **Number randomized**  105  **Age**  6-16 years (range)  **Co-morbidities/Co-medications**  Yes, oppositional disorder (2%) and conduct disorders (5%)– Yes, 10%  **ADHD subtypes**  Combined (72%), inattentive (23%), hyperactive/impulsive (5%) | **Arm 1**  ATX: atomoxetine 1.4 mg/kg/day  **Arm 2**  Placebo: placebo pill | **Treatment response** (as a dichotomous outcome)  Responders based on the achievement of a > 25% from baseline to endpoint in the ADHD-RS-IV-Parent:Inv total score (rater: parents)  **All-cause treatment discontinuation** (as a dichotomous outcome)  Yes, reported as study discontinuation  **Tolerability** (as a dichotomous outcome)  Yes, reported as study discontinuations due to AEs  **Serious adverse events** (as dichotomous outcome)  None  **Specific adverse events** (as dichotomous outcome)  Loss of appetite/anorexia (as an adverse event)  Decreased weight (as an adverse event e.g. loss ≥ 7% from baseline)  Insomnia (as an adverse event)  Sleep disturbances (as an adverse event) |
| Perreau-Linck et al., 2010^155^ | **Eligibility criteria**  Children aged 8-13; no comorbid disorder; IQ of 90 or higher; an abnormal EEG pattern with an increased anterior theta nd decreased posterior beta activity as measured by qEEG. Exclusion critera were: DSM-IV type I disorders; neurological conditions (e.g. epilepsy, dyslexia or learning disorders), and previous NF training  **Diagnostic criteria**  DSM-IV  **Number randomized**  39  **Age**  8-13 years (range)  **Co-morbidities/Co-medications**  None – Yes, 11% stimulants  **ADHD subtypes**  Not reported | **Arm 1**  Neurofeedback (theta-beta training): the NF training protocols comprised forty 1-hr sessions (20 meetings of 2 sessions each, with a short break in between 2 consecutive sessions). Parents were not allowed access to the raining room and did not get feedback on their child’s progress. NF training was provided using the Biograph Infiniti software with ProComp2 Legacy Suite (Thought Technology Ltd., Montreal, Canada). Training sessions took place on an individual basis. Each session comprised about 30 min of NF. The remaining time concerned installing the sensors and coaching. EEG was recorded from C4, with reference placed on the left earlobe and ground on the right earlobe. A sampling rate of 128 Hz with 2 epochs was used. Skin impedance was less than 5 kΩ. Theta band was set at 4-8 Hz and SMR at 12-15 Hz. Trainers were volunteer undergraduate students. Each participant was trained by a maximum of two trainers  **Arm 2**  Placebo: placebo (sham-control) neurofeedback | **Treatment response** (as a dichotomous outcome)  Not reported/data not abstractable  **All-cause treatment discontinuation** (as a dichotomous outcome)  Yes, reported as study drop-outs  **Tolerability** (as a dichotomous outcome)  Yes, reported as study drop-outs due to AEs  **Serious adverse events** (as dichotomous outcome)  Not reported  **Specific adverse events** (as dichotomous outcome)  Not reported |
| Salehi et al., 2010^156^ | **Eligibility criteria**  Children and adolescents aged 6-14; total and/or subscale scores on ADHD-RS-IV School Version at least 1.5 SD above norms for patient's age and gender. All patients had combined subtype of ADHD and were newly diagnosed. Children were excluded if they had a history or current diagnosis of pervasive developmental disorders, schizophrenia or other psychiatric disorders (DSM-IV axis I); any current psychiatric comorbidity that required pharmacotherapy; any evidence of suicide risk and mental retardation (IQ<70 based on clinical judgment). In addition, patients were excluded if they had a clinically significant chronic medical condition, including organic brain disorder, seizures and, current abuse or dependence on drugs within 6 months. Additional exclusion criteria were hypertension and hypotension  **Diagnostic criteria**  DSM-IV  **Number randomized**  50  **Age**  6-14 years (range)  **Co-morbidities/Co-medications**  No/Not reported  **ADHD subtypes**  Combined (100%) | **Arm 1**  MPH-SA low/medium dose: methylphenidate 20-30 mg/day  **Arm 2**  Herbal therapy: *Ginkgo biloba* 80-120 mg/day | **Treatment response** (as a dichotomous outcome)  Responders based on the achievement of a 40% or greater decrease from baseline to endpoint in the ADHD-RS-IV (rater: parents and teachers)  **All-cause treatment discontinuation** (as a dichotomous outcome)  Yes, reported as study drop-outs  **Tolerability** (as a dichotomous outcome)  Not reported  **Serious adverse events** (as dichotomous outcome)  Not reported  **Specific adverse events** (as dichotomous outcome)  Loss of appetite/anorexia (as an adverse event)  Decreased weight (as an adverse event)  Insomnia (as an adverse event)  Anxiety (as an adverse event) |
| Thurstone et al., 2010^157,158^ | **Eligibility criteria**  Adolescents aged 13-19; DSM-IV ADHD checklist  score greater than or equal to 22; DSM-IV  diagnosis of at least one nonnicotine SUD; plans  to live locally for at least 4 months; and willingness  to participate in motivational interviewing/ cognitive behavioral therapy (MI/CBT) for SUD during the medication trial. Exclusion criteria were: mental illness that could not be managed as an outpatient (e.g., serious suicidal ideation), or without concurrent psychotropic medication; history of bipolar disorder or psychosis; medical contraindication to taking ATX; pregnancy, breast feeding, or unwillingness to use an effective form of birth control while in the study; and SUD that could not be managed as an outpatient or without concurrent psychotropic medications (e.g., alcohol withdrawal, opioid withdrawal)  **Diagnostic criteria**  DSM-IV  **Number randomized**  70  **Age**  13-19 years (range)  **Co-morbidities/Co-medications**  Yes, all comorbid substance use disorders (100%), conduct disorders (53%), depression (29%) – Not reported  **ADHD subtypes**  Combined (76%), inattentive (17%), hyperactive/impulsive (7%) | **Arm 1**  ATX+BT (child and parent training): combination of both atomoxetine (1.2 mg/day) and cognitive-behavioral therapy including parent and child training (described below)  **Arm 2**  BT (child and parent training): All participants received weekly individual MI/ CBT. The manual that was used was chosen because of its feasibility and empirical support as a psychosocial treatment for SUD in adolescents with co-occurring psychiatric and substance use disorders. The MI/CBT consisted of hour-long, weekly individual sessions and could include up to three family sessions. Cognitive, behavioral, and motivational techniques were used to help adolescents reduce their drug use and improve coping skills. Core modules included goal setting, a functional analysis of drug use, and coping with cravings. Subsequent modules included anger management, communication skills, mood management, drug refusal skills, and problem solving. The principal investigator and one of the research therapists were trained by the manual’s developers. The principal investigator then trained the other five research therapists. Each therapist was audiotaped at  least once during the study and chose a convenient  session for the taping. A total of 12 sessions from a  variety of different modules in the MI/CBT manual  were reviewed by the principal investigator for fidelity  and adherence, and no session fell below standards developed before the study began. The clinical program offered the MI/CBT sessions at no cost to research participants. There was also no cost to participants for the study procedures and medication | **Treatment response** (as a dichotomous outcome)  Clinician-rated Clinical Global Impression Improvement (CGI-I) scale – e.g. responder considered very much improved or much improved (CGI-I ≤ 2)  **All-cause treatment discontinuation** (as a dichotomous outcome)  Yes, reported as study discontinuations  **Tolerability** (as a dichotomous outcome)  Yes, reported as study discontinuations due to AEs (e.g. one case of depression with ATX)  **Serious adverse events** (as dichotomous outcome)  Yes, reported  **Specific adverse events** (as dichotomous outcome)  Loss of appetite/anorexia (as an adverse event)  Sleep disturbances (as an adverse event)  Cardiovascular event – tachycardia (as an adverse event) |
| Waxmonsky et al., 2010^159^ | **Eligibility criteria**  Children aged 6-12. Exclusion criteria were: current or past history of seizures; other physical conditions that recluded administration of ATX (e.g. marked cardiac conduction delay); documented failed trial of ATX (3 weeks or more on treatment with at least 0.8 mg/kg/day or a documented inability to tolerate this dose); serious forms of psychopathology other than ADHD, such as autism, bipolar disorders, schizophrenia, or any other psychopathology requiring urgent treatment with psychotropic medication; any history of major depression requiring treatment, or any past history of self-harm or serious suicidal ideation; an IQ < 75; no evidence of ADHD-related impairment at school  **Diagnostic criteria**  DSM-IV-TR  **Number randomized**  56  **Age**  6-12 years (range)  **Co-morbidities/Co-medications**  Yes, oppositional disorder (43%), conduct disorders (39%)– Not reported  **ADHD subtypes**  Combined (86%), inattentive (13%), hyperactive/impulsive (2%) | **Arm 1**  ATX+BT (child, parent and teacher training): combination of both atomoxetine (1.2 mg/day) and behavioral therapy including child, parent and teacher training. There were 3 components in the BT: a parenting program (e.g. 8 sessions of the Community Oriented Parent Education [COPE] program), a social skills program and a scholl-based daily report card  **Arm 2**  ATX: atomoxetine 1.2 mg/kg/day | **Treatment response** (as a dichotomous outcome)  Clinician-rated Clinical Global Impression Improvement (CGI-I) scale – e.g. responder considered very much improved or much improved (CGI-I ≤ 2)  **All-cause treatment discontinuation** (as a dichotomous outcome)  Yes, reported as study discontinuations  **Tolerability** (as a dichotomous outcome)  Yes, reported as study discontinuations due to AEs  **Serious adverse events** (as dichotomous outcome)  Not reported  **Specific adverse events** (as dichotomous outcome)  Not reported |
| Zarinara et al., 2010^160^ | **Eligibility criteria**  Children and adolescents aged 6-13; total and/or subscale scores on ADHD-RS-IV School Version at least 1.5 SD above norms for patient's age and gender. All patients had combined subtype of ADHD and were newly diagnosed. Children were excluded if they had a history or current diagnosis of pervasive developmental disorders, schizophrenia or other psychiatric disorders (DSM-IV axis I); any current psychiatric comorbidity that required pharmacotherapy; any evidence of suicide risk and mental retardation (IQ<70 based on clinical judgment). In addition, patients were excluded if they had a clinically significant chronic medical condition, including organic brain disorder, seizures and, current abuse or dependence on drugs within 6 months. Additional exclusion criteria were hypertension and hypotension  **Diagnostic criteria**  DSM-IV-TR  **Number randomized**  38  **Age**  6-13 years (range)  **Co-morbidities/Co-medications**  No/Not reported  **ADHD subtypes**  Combined (100%) | **Arm 1**  MPH-SA low/medium dose: methylphenidate 20-30 mg/day  **Arm 2**  VENLAF: venlafaxine 50-75 mg/day | **Treatment response** (as a dichotomous outcome)  Responders based on the achievement of a 40% or greater decrease from baseline to endpoint in the ADHD-RS-IV (rater: parents and teachers)  **All-cause treatment discontinuation** (as a dichotomous outcome)  Yes, reported as study drop-outs  **Tolerability** (as a dichotomous outcome)  Not reported  **Serious adverse events** (as dichotomous outcome)  Not reported  **Specific adverse events** (as dichotomous outcome)  Loss of appetite/anorexia (as an adverse event)  Insomnia (as an adverse event) |
| Abbasi et al., 2011^161^ | **Eligibility criteria**  Children and adolescents aged 7-13; total and/or subscale scores on ADHD-RS-IV School Version at least 1.5 SD above norms for patient's age and gender. All patients had combined subtype of ADHD and were newly diagnosed. Children were excluded if they had a history or current diagnosis of pervasive developmental disorders, schizophrenia or other psychiatric disorders (DSM-IV axis I); any current psychiatric comorbidity that required pharmacotherapy; any evidence of suicide risk and mental retardation (IQ<70 based on clinical judgment). In addition, patients were excluded if they had a clinically significant chronic medical condition, including organic brain disorder, seizures and, current abuse or dependence on drugs within 6 months. Additional exclusion criteria were hypertension and hypotension  **Diagnostic criteria**  DSM-IV-TR  **Number randomized**  40  **Age**  7-13 years (range)  **Co-morbidities/Co-medications**  No/Not reported  **ADHD subtypes**  Combined (100%) | **Arm 1**  MPH-SA low/medium dose: methylphenidate 20-30 mg/day  **Arm 2**  MPH-SA low/medium dose + L-CARN: l-carnitine 500-1500 mg/day and adjunctive methylphenidate 20-30 mg/day | **Treatment response** (as a dichotomous outcome)  Not reported/data not abstractable  **All-cause treatment discontinuation** (as a dichotomous outcome)  Yes, reported as study drop-outs  **Tolerability** (as a dichotomous outcome)  Not reported  **Serious adverse events** (as dichotomous outcome)  Not reported  **Specific adverse events** (as dichotomous outcome)  Loss of appetite/anorexia (as an adverse event)  Decreased weight (as an adverse event)  Sleep disturbances (as an adverse event)  Anxiety (as an adverse event) |
| Arnold et al., 2011^162^ | **Eligibility criteria**  Children aged 6-14 years. Any co-morbid diagnosis that required psychopharmacotherapy other than catecholaminergic stimulant was excluded. The only other exclusions were current zinc supplementation or anything that would interfere with assessments or study drug or contraindicate study drug (including other diagnoses that contraindicate study drug). States of infection or other inflammation required postponement of entry until the inflammation cleared because of the interaction of inflammation with zinc levels  **Diagnostic criteria**  DSM-IV  **Number randomized**  52  **Age**  6-14 years (range)  **Co-morbidities/Co-medications**  Yes, oppositional disorders or conduct disorders (65%), learning disorders (19%), depression (17%) – Yes, 15% concomitant amphetamine with zinc  **ADHD subtypes**  Combined (73%), inattentive (27%) | **Arm 1**  Zinc: Zinc supplementation (15-30 mg/day) as glycinate  **Arm 2**  Placebo: placebo pill | **Treatment response** (as a dichotomous outcome)  Not reported/data not abstractable  **All-cause treatment discontinuation** (as a dichotomous outcome)  Yes, reported as study drop-outs  **Tolerability** (as a dichotomous outcome)  Yes, reported as study drop-outs due to AEs  **Serious adverse events** (as dichotomous outcome)  Not reported  **Specific adverse events** (as dichotomous outcome)  Loss of appetite/anorexia (as an adverse event)  Sleep disturbances (as an adverse event)  Anxiety (as an adverse event) |
| Bakhshayesh et al., 2011^163^ | **Eligibility criteria**  Children aged 6-14; a primary diagnosis of hyperkinetic disorder (disturbance of activity and attention; ICD-10: F90.0) or attention deficit without hyperactivity (ICD-10: F98.8); an IQ>80 (CPM, SPM); no known neurological or gross organic diseases and no hyperkinetic conduct disorders (ICD-10: F90.1) or pervasive developmental disorders. Children currently taking stimulant medication were not excluded from the study, but their parents were asked to keep medication levels constant throughout the training period in order to avoid interference effects  **Diagnostic criteria**  ICD-10  **Number randomized**  38  **Age**  6-14 years (range)  **Co-morbidities/Co-medications**  Yes, motor skills disorder (14%), enuresis (6%), emotional disorder (6%), conduct disorders (3%) – Yes, 20% stimulants  **ADHD subtypes**  Not reported | **Arm 1**  Neurofeedback (theta-beta training): A Nexus amplifier was used for neurofeedback (NF) training. The connection between the electrodes and skin was continuously monitored throughout the session. Nexus uses DC offset checking which is done online and does not interfere with the signals, instead of using an impedance check which interferes with the EEG signals. In order to reduce skin impedance, an opaque adhesive paste (Ten20) was applied. Artefacts were controlled automatically. The thresholds were fixed (theta: 4–8 Hz, beta: 16–20 Hz). The present study employed a theta/beta protocol; thus, active electrodes were located on CPz and FCz, based on the international 10/20 system. The reference electrode was installed on the mastoid. Children in the NF group were instructed to use their concentration when  playing the different computer games  **Arm 2**  Control: In the control group, electrodes were placed on the frontalis musculature to measure EMG amplitudes. The children were instructed to use relaxation in order to play the games | **Treatment response** (as a dichotomous outcome)  Not reported/data not abstractable  **All-cause treatment discontinuation** (as a dichotomous outcome)  Yes, reported as study discontinuations  **Tolerability** (as a dichotomous outcome)  Not reported  **Serious adverse events** (as dichotomous outcome)  Not reported  **Specific adverse events** (as dichotomous outcome)  Not reported |
| Dittmann et al., 2011^164^ | **Eligibility criteria**  Children aged 6-17 with comorbid ODD/CD. Patients who had a history of bipolar I or II disorder, psychosis, pervasive developmental disorder, or seizure disorder (other than febrile seizures) were excluded. Patients were excluded if they were at serious suicidal risk, as determined by the investigator, or if they were likely to require psychotropic medications other than study drug or a structured psychotherapy. Psychotherapy initiated before study participation was acceptable  **Diagnostic criteria**  DSM-IV-TR  **Number randomized**  180  **Age**  6-17 years (range)  **Co-morbidities/Co-medications**  Yes, oppositional disorders (74%), conduct disorders (24%), anxiety (2%) – Not reported  **ADHD subtypes**  Combined (76%), inattentive (19%), hyperactive/impulsive (5%) | **Arm 1**  ATX: atomoxetine 1.2 mg/kg/day  **Arm 2**  Placebo: placebo pill | **Treatment response** (as a dichotomous outcome)  Not reported/data not abstractable  **All-cause treatment discontinuation** (as a dichotomous outcome)  Yes, reported as study discontinuation  **Tolerability** (as a dichotomous outcome)  Yes, reported as study discontinuations due to AEs  **Serious adverse events** (as dichotomous outcome)  Yes, reported  **Specific adverse events** (as dichotomous outcome)  Loss of appetite/anorexia (as an adverse event)  Cardiovascular event – tachycardia (as an adverse event) |
| Findling et al., 2011^165,166^ | **Eligibility criteria**  Adolescents aged 13-17 years; moderate to severe ADHD symptoms at baseline (score of ≥28 on the ADHD Rating Scale IV: Clinician Version [ADHDRS-  IV] assessment); age-appropriate intellectual function and blood pressure (BP) measurements ≤95th percentile for age, gender, and height. Participants with conduct disorder or a comorbid  psychiatric diagnosis (oppositional defiant disorder  was not exclusionary) requiring medication were excluded. Those participants with a concurrent chronic/acute medical condition that might confound efficacy/safety assessments or pose a safety risk, a history of seizures, tic disorder or family history of Tourette disorder, family history of sudden cardiac death or arrhythmia, abnormal thyroid function (a stable dose of thyroid  medication for at least 3 months was permitted), glaucoma, or those considered a suicide risk were  excluded. Body mass index could not be <5th or >97th percentile for age and gender. Participants who tested positive on urine drug screen (except current stimulant therapy), or had a recent history of suspected substance abuse (excluding nicotine) were not enrolled. Pregnant/lactating females were not included. Participants with clinically significant electrocardiogram (ECG) findings, who required medications with central nervous system effects, with failure to  respond to and/or intolerance of amphetamine therapy, and/or who were well controlled on current ADHD medication with acceptable safety and efficacy were disqualified  **Diagnostic criteria**  DSM-IV-TR  **Number randomized**  314  **Age**  13-17 years (range)  **Co-morbidities/Co-medications**  None – Not reported  **ADHD subtypes**  Combined (86%) | **Arm 1**  LDX-LA: lisdexamphetamine dimesylate 30, 50 or 70 mg/day  **Arm 2**  Placebo: placebo pill | **Treatment response** (as a dichotomous outcome)  Clinician-rated Clinical Global Impression Improvement (CGI-I) scale – e.g. responder considered very much improved or much improved; ADHD-RS ≥ 30% reduction from baseline in total score (rater: clinician)  **All-cause treatment discontinuation** (as a dichotomous outcome)  Yes, reported as study discontinuations  **Tolerability** (as a dichotomous outcome)  Yes, reported as study discontinuations due to AEs  **Serious adverse events** (as dichotomous outcome)  None  **Specific adverse events** (as dichotomous outcome)  Loss of appetite/anorexia (as an adverse event)  Decreased weight (as an adverse event)  Insomnia (as an adverse event)  Cardiovascular event - blood pressure increase (as adverse event e.g. increased SBP≥ 130 mm Hg at 2 consecutive weeks)  Increased pulse values (as an adverse event e.g. 100 bpm or greater + increase from baseline of ≥ 15 bpm)  Incresed heart rate (as an adverse event)  QT prolongation (as an advserse event) |
| Jain et al., 2011^167^ | **Eligibility criteria**  Children and adolescents aged 6-17 years; patient’s clinical research physician and a minimum score of 26 on the ADHD-RS-IV; good  health, be able to swallow tablets, be mentally competent, and have a body mass index of at least the fifth percentile for the patients’ age group. Patients with a concomitant diagnosis of tics or oppositional defiant disorder were eligible for study inclusion. Female patients of childbearing age who were pregnant or lactating or who refused to use birth control were excluded from the study. Patients were also excluded if they had a clinically significant illness or abnormality that would increase the safety risk of clonidine or if they had a clinically significant abnormality on electrocardiographic readings that were interpreted by a single entity. Patients with a concomitant diagnosis or history of a psychiatric disorder that required psychotropic medication  and patients with a severe concomitant Axis I or II  disorder that could interfere with assessment of  clonidine safety and efficacy were also excluded. In  addition, patients with a history of conduct disorders, syncopal episodes, or seizures (except for febrile seizure before 2 years of age) were not enrolled. Patients with known drug abuse, a history of drug abuse, or a history of CLON intolerance, including dermatologic reaction  to transdermal CLON, were excluded. Patients were also not enrolled if they had used any investigational drug within 30 days of the study initiation or had a positive drug test result for any medications other than those used for the treatment of ADHD  **Diagnostic criteria**  DSM-IV  **Number randomized**  236  **Age**  6-17 years (range)  **Co-morbidities/Co-medications**  None/Yes, the most commonly  used class of concomitant medications was cough  and cold preparations (11%), systemic antibacterial agents, anti-inflammatory products, and antirheumatic products  **ADHD subtypes**  Not reported | **Arm 1**  CLON-LA: clonidine long acting 0.2 or 0.4 mg/day  **Arm 2**  Placebo: placebo pill | **Treatment response** (as a dichotomous outcome)  Not reported/data not abstractable  **All-cause treatment discontinuation** (as a dichotomous outcome)  Yes, reported as study discontinuation  **Tolerability** (as a dichotomous outcome)  Yes, reported as study discontinuation due to AEs  **Serious adverse events** (as dichotomous outcome)  Not reported  **Specific adverse events** (as dichotomous outcome)  Insomnia (as an adverse event)  Cardiovascular event – bradycardia (as an adverse event)  QT prolongation (as an advserse event) |
| Giblin et al., 2011^168^ | **Eligibility criteria**  Children aged 6-12 years; a minimum baseline  score of 28 on the ADHD-RS-IV. Exclusion criteria were: any current severe Axis I or Axis II disorders or any other uncontrolled comorbid psychiatric diagnosis, a history of symptoms suggestive of a primary sleep disorder or an identified sleep disorder (e.g., sleep apnea, restless legs syndrome, or sleep disordered breathing) and abnormal laboratory values or ECG reading  **Diagnostic criteria**  DSM-IV-TR  **Number randomized**  24  **Age**  6-12 years (range)  **Co-morbidities/Co-medications**  None – Not reported  **ADHD subtypes**  Not reported | **Arm 1**  LDX-LA: lisdexamphetamine dimesylate 30, 50 or 70 mg/day  **Arm 2**  Placebo: placebo pill | **Treatment response** (as a dichotomous outcome)  Not reported/data not abstractable  **All-cause treatment discontinuation** (as a dichotomous outcome)  Not reported  **Tolerability** (as a dichotomous outcome)  Not reported  **Serious adverse events** (as dichotomous outcome)  Not reported  **Specific adverse events** (as dichotomous outcome)  Cardiovascular event - blood pressure increase (as an adverse event)  Increased pulse values (as an adverse event) |
| Kang et al., 2011^169^ | **Eligibility criteria**  All participants were screened for substance use history and psychiatric disorders such as Tourette’s Syndrome, mood disorders, anxiety disorders, and pervasive developmental disorders, as determined by a semi-structured clinical interview (K-SADSPL-  K). Exclusion criteria were: patients with a history or current episode of Axis I psychiatric disease; patients with an IQ < 80; and patients with neurological or medical disorders  **Diagnostic criteria**  DSM-IV (all patients were diagnosed in the Department of Psychiatry)  **Number randomized**  24  **Age**  8.5 years (range not reported)  **Co-morbidities/Co-medications**  None – Not reported  **ADHD subtypes**  Not reported | **Arm 1**  MPH-SA low/medium dose+BT (child training): methylphenidate (30 mg/day) in combination with behavioral therapy (education for behavior control). The BT intervention consisted of 12 sessions (S); S1: a  time for self-introduction, S2: a comparison of good behavior and bad behavior, S3: a review of self behavior, S4: a comparison of self and others, S5: doing well with family, S6: making friends I, S7: making friends II, S8: treatment methods for attention defi cit, S9: treatment methods for hyperactivity, S10: anger control, S11: guest speakers who have recovered from ADHD. S12: review of ADHD education  **Arm 2**  MPH-SA low/medium dose+physical activity: methylphenidate (26.7 mg/day) in combination with physical activity or sports therapy. The research team for sports therapy consisted of 1 psychiatrist, 1 sports psychologist, and 2 teaching assistants majoring in sports psychology. From 2:00 pm to 3:30 pm every Tuesday and Friday, 90 min sessions of sports therapy were scheduled as follows: 5 min for greeting, 15 min for aerobic exercise, 10 min for seated rest, 20 min for goal-directed exercise, 10 min for another seated rest, 20 min for rope jumping, and 10 min for feedback. Aerobic exercise consisted of a 200-m shuttle run and a 200-m zigzag run. Goal-direct exercises included throwing tennis balls and magnetic darts at a target score board. Jump roping involved 2 instructors swinging a 10-m rope, while another instructor and 3 or 4 children jumped. In every therapy, children with good performance and a nice attitude were rewarded with candy or a small toy. For safety purposes, the heart rate was checked every 30 min during the sports therapy. In addition, if a child perceived excess exertion and raised his hand, he was allowed to rest until recovered | **Treatment response** (as a dichotomous outcome)  Not reported/data not abstractable  **All-cause treatment discontinuation** (as a dichotomous outcome)  Yes, reported as study discontinuations  **Tolerability** (as a dichotomous outcome)  Yes, reported as study discontinuations due to AEs  **Serious adverse events** (as dichotomous outcome)  Not reported  **Specific adverse events** (as dichotomous outcome)  Not reported |
| Kollins et al., 2011^170^ | **Eligibility criteria**  Children and adolescents aged 6-17; had a stable  regimen of stimulant treatment (e.g. MPH or AMPH) during the previous 4 weeks; inadequate stimulant medication response, defined as a total  score ≥26 on the ADHD-RS-IV questionnaire  after a minimum of 4 weeks on a stable stimulant regimen (ie, ≤10% variation in dose). Additional inclusion criteria were intelligence quotient  estimated to be ≥80 by the investigator and a BMI in the ≥5th percentile for the patient’s gender and age. Exclusion criteria were: current diagnosis or history of a psychiatric disorder that required psychotropic medication or severe comorbid  Axis I or Axis II disorder that could interfere with assessment of clonidine efficacy and safety; a history of conduct disorder; a history of syncopal episodes or seizures (except for febrile seizures); current or past drug abuse; a history of clonidine  Intolerance; or used any investigational drug within 30 days of the study initiation or had a positive drug test (except for ADHD medication). Patients were also excluded if they had a clinically significant illness or abnormality that would increase the safety risk of clonidine or if they had clinically significant electrocardiogram readings. Females of childbearing age who were pregnant or lactating or who refused to use birth control were not allowed to participate. Concomitant use of antihypertensive medications, psychotropic drugs, oral corticosteroids, sedating antihistamines, antidiabetic medications,  diet aids, and bronchodilators >3 days per week was not allowed  **Diagnostic criteria**  DSM-IV-TR  **Number randomized**  198  **Age**  6-17 (range)  **Co-morbidities/Co-medications**  None/All with stimulants  **ADHD subtypes**  Not reported | **Arm 1**  MPH-LA or LDX-LA + CLON-LA: combination of stimulants (Concerta® 39 mg/day or Vyvanse 49 mg/day were the most frequent among other stimulants) and clonidine long acting (0.1-0.4 mg/day)  **Arm 2**  MPH-LA or LDX-LA: stimulants (Concerta® 42 mg/day or Vyvanse 52 mg/day were the most frequent among other stimulants) | **Treatment response** (as a dichotomous outcome)  Responders were defined posthoc as patients with a ≥ 30% reduction in ADHD-RS total score from baseline (rater: clinician/investigator) who were considered to be “very much” or “much improved” on the CGI-I (rater: clinician/investigator)  **All-cause treatment discontinuation** (as a dichotomous outcome)  Yes, reported as study discontinuations  **Tolerability** (as a dichotomous outcome)  Yes, reported as study discontinuations due to AEs  **Serious adverse events** (as dichotomous outcome)  Not reported  **Specific adverse events** (as dichotomous outcome)  Insomnia (as an adverse event)  Cardiovascular events – increased heart rate (as an adverse event) |
| Kollins et al., 2011^171^ | **Eligibility criteria**  Children and adolescent aged 6-17 years; a baseline score ≥24 on the ADHD-RS-IV and a baseline score ≥4 on the Clinical Global Impressions-Severity (CGI-S) scale. Exclusion criteria were any current comorbid psychiatric diagnosis (except oppositional defiant disorder),  weight <25 kg (55 lb), cardiac conditions that might have increased the safety risk to the subject, or a Pediatric Daytime Sleepiness Scale (PDSS) score ≥22 at screening and/or baseline  **Diagnostic criteria**  DSM-IV-TR  **Number randomized**  178  **Age**  6-17 years (range)  **Co-morbidities/Co-medications**  Not reported/No  **ADHD subtypes**  Combined (75%), inattentive (24%), hyperactive/impulsive (2%) | **Arm 1**  GUAN-LA: guanfacine long acting 1-3 mg/day  **Arm 2**  Placebo: placebo pill | **Treatment response** (as a dichotomous outcome)  Global improvement based on Clinical Global Impression Improvement (CGI-I) scale. Subjects with improvement of “very much” or “much” improved were considered responders e.g. GGI-I ≤ 2 (rating evaluations reported by clinician  **All-cause treatment discontinuation** (as a dichotomous outcome)  Yes, reported as early discontinuations  **Tolerability** (as a dichotomous outcome)  Yes, reported as early discontinuations due tu AEs  **Serious adverse events** (as dichotomous outcome)  Yes, reported  **Specific adverse events** (as dichotomous outcome)  Not reported |
| Kratochvil et al., 2011^172,173^ | **Eligibility criteria**  Children aged 5-6 years; arent and child must be English speaking; child has been living with parent/guardian for at least six months; meets criteria for ADHD on the DISC, on clinical interview, and on clinical consensus conference; ADHD is primary disorder with symptoms present for at least 9 months; ADHD-IV-Rating Scale (ADHD-IV-RS) score that is at least 1.5 standard deviations above age and sex norms; score of 55 or below on the Children's Global Assessment Scale; score of 4 or greater on the Clinical Global Impression Scale; estimated IQ of 70 or greater; currently participating in school at least 2 half-days per week; able to identify a teacher who can make valid assessments; patient and parent are able to attend regular study visits. Exclusion criteria were: currently taking other psychotropic medications or other medications with effects on the central nervous system; currently being treated effectively with ATX; major medical conditions that might interfere with study medications; history of or current clinically significant kidney illness; wvidence of adjustment disorder, autism, psychosis, bipolar disorder, suicide ideations, or any other psychiatric disorder requiring treatment with additional psychotropic medication; history of physical, sexual, or emotional abuse impacting clinical presentation; prior failure to respond to an adequate trial of ATX  **Diagnostic criteria**  DSM-IV (DISC)  **Number randomized**  101  **Age**  5-6 years (range)  **Co-morbidities/Co-medications**  Yes, oppositional disorder (35%), enuresis (17%) – No  **ADHD subtypes**  Combined (82%), inattentive (9%), hyperactive/impulsive (10%) | **Arm 1**  ATX+BT (parent training): combination of both atomoxetine (1.4 mg/day) and behavioral therapy including parent training (described below)  **Arm 2**  BT (parent training): BT included parent training. Each study visit lasted 30 to 40 minutes; half of that time was spent providing psychoeducation about ADHD  and behavioral management strategies using handouts adapted from McMahon and Forehand’s *Helping* *the Noncompliant Child: Family-Based* *Treatment for Oppositional Behavior*. Although parents were encouraged to implement the strategies, and pharmacotherapists inquired about their success in doing so, no skills training was provided | **Treatment response** (as a dichotomous outcome)  Global improvement based on Clinical Global Impression Improvement (CGI-I) scale. Subjects with improvement of “very much” or “much” improved were considered responders e.g. GGI-I ≤ 2 (rating evaluations reported by clinician); Global improvement based on Clinical Global Impression Severy (CGI-S) scale e.g. CGI-S ≤ 3  **All-cause treatment discontinuation** (as a dichotomous outcome)  Yes, reported as discontinuations  **Tolerability** (as a dichotomous outcome)  Yes, reported as discontinuations due to AEs  **Serious adverse events** (as dichotomous outcome)  None  **Specific adverse events** (as dichotomous outcome)  Loss of appetite/anorexia (as an adverse event)  Decreased weight (as an adverse event)  Insomnia (as an adverse event)  Anxiety (as an adverse event) |
| Lansbergen et al., 2011^174^ | **Eligibility criteria**  Children aged 6-15; IQ of at least 80; QEEG deviated at least 1.5 SD from a normative database; they were psychopharmaca- naïve or -free, or used a stable dosage of psychostimulants or ATX with room for improvement. Room for improvement was defined as an average score of more than 1 SD above the mean on ADHD-DSM-IV rating scale. Children on a stable dosage of psychostimulants or ATX were included. Exclusion criteria were: intensive (i.e., weekly) individual or group psychotherapy during the experiment; used medication other than psycho-stimulants or ATX; had a comorbid disorder, other than oppositional defiant disorder (ODD) or an anxiety disorder; had a neurological disorder and/or a cardiovascular disease; participated in another clinical trial; received neurofeedback training in the past;  used alcohol or drugs  **Diagnostic criteria**  DSM-IV-TR  **Number randomized**  14  **Age**  6-15 years (range)  **Co-morbidities/Co-medications**  Not reported – Yes, 64% stimulants  **ADHD subtypes**  Not reported | **Arm 1**  Neurofeedback (IFBT): For all children in the present study, eyes open and eyes closed raw EEG data were visually inspected and quantitative EEG (QEEG) data from each participant before EEG-neurofeedback training was analyzed using Deymed Truescan software. EEG data with eye movements and artifacts were removed. Subsequently, individual QEEGs were compared to the NeuroGuide database and deviations from the normative database were identified (FFT maximal z scores). The aim of the EEG-neurofeedback training was to normalize power within specific frequency bands and at specific electrode sites. Children in the EEG-neurofeedback group received feedback on their real-time EEG-signal. During training, all children watched a movie for 20 min. They were asked to sit as quiet as possible in a comfortable arm chair in front of a 17–19 in TFT computer screen showing a part of the movie. EEG data were obtained from the active electrode(s) placed on the scalp at  the location(s) of interest. The reference was  linked ears or left ear if the active electrode was placed on the middle of the scalp. All electrode impedances were kept under 6 kX. Sampling rate was 256 Hz. EEG data were filtered (DC-120 Hz) and de-artefacted online (peakto-peak amplitude criterion of 120 lV). Positive feedback was provided by both brightening the computer screen and presenting an auditory tone when the production of SMR (estimated from the filtered and de-artefacted EEG signal) remained above threshold, and/or theta and beta activity (estimated from the filtered and de-artefacted EEG signal) remained below threshold. Reward threshold levels were automatically adjusted based on the digitally filtered realtime EEG signal every 30 s so that the child was rewarded about 80% of the time (i.e., received positive feedback). Consequently, the amount of reward remained at about the same level across sessions and across groups. During training, children were instructed to try to self-regulate their brain activity by receiving positive feedback based on the real-time EEG signal. The children were trained over a period of approximately 4 months with 2 sessions per week, in total 30 sessions. The duration of each session was 45 min and included approximately 20 min of uninterrupted EEG-neurofeedback or placebo feedback training. Training was conducted in an ‘active focusing state’ with eyes open  **Arm 2**  Placebo: placebo feedback | **Treatment response** (as a dichotomous outcome)  Global improvement based on Clinical Global Impression Improvement (CGI-I) scale. Subjects with improvement of “very much” or “much” improved were considered responders e.g. GGI-I ≤ 2 (rating evaluations reported by clinician)  **All-cause treatment discontinuation** (as a dichotomous outcome)  Not reported  **Tolerability** (as a dichotomous outcome)  Not reported  **Serious adverse events** (as dichotomous outcome)  Not reported  **Specific adverse events** (as dichotomous outcome)  Not reported |
| Pelsser et al., 2011^175^ | **Eligibility criteria**  Children aged 4-8 years (sufficiently young to  maximise dietary compliance); parents with adequate knowledge of Dutch and who were motivated to follow a 5-week restricted elimination diet. Exclusion criteria were: children receiving drugs or behavioural therapy for ADHD, children already following a diet, or family circumstances that were likely to prevent completion of the study. The presence of comorbid psychiatric disorders was not a reason for exclusion  **Diagnostic criteria**  DSM-IV  **Number randomized**  100  **Age**  4-8 years (range)  **Co-morbidities/Co-medications**  Yes, oppositional disorders (47%), conduct disorders (8%)/None  **ADHD subtypes**  Combined (85%), inattentive (6%), hyperactive/impulsive (9%) | **Arm 1**  Restricted elimination diet (elimination diet): the diet group started a 5-week individually designed restricted  elimination diet. Briefly, the diet consisted of the fewfoods diet (e.g. rice, meat, vegetables, pears, and water) complemented with specifi c foods such as potatoes, fruits, and wheat. The aim was to create an elimination diet as comprehensive as possible for each individual child, to make the intervention easy for children and their parents to follow. If the parents reported no behavioural changes by the end of the second diet week, the diet was gradually restricted to the few-foods diet only  **Arm 2**  Control (healthy diet): instructions for a healthy diet | **Treatment response** (as a dichotomous outcome)  Responders based on the achievement of a 40% or greater decrease from baseline to endpoint in the ADHD-RS total score (rater: clinician)  **All-cause treatment discontinuation** (as a dichotomous outcome)  Yes, reported as study dropp-outs  **Tolerability** (as a dichotomous outcome)  Yes, reported as study dropp-outs due to illness  **Serious adverse events** (as dichotomous outcome)  Not reported  **Specific adverse events** (as dichotomous outcome)  Not reported |
| Riggs et al., 2011^176^ | **Eligibility criteria**  Adolescents aged 13-18; DSM-IV diagnostic criteria for ≥ 1 non-nicotine substance use disorder; DSM-IV ADHD Symptom Checklist score ≥ 22 derived from the adolescentcompleted. Exclusion criteria: serious medical illness or cardiac illness; history of tic disorder; pregnant or breastfeeding; meets DSM-IV criteria for current or lifetime psychotic disorder; meets DSM-IV criteria for current or lifetime bipolar disorder; requires/or prescribed other concurrent psychotropic medication; taking any medications that may produce interactions with OROS MPH; opiate dependence; methamphetamine abuse, dependence or past month use; suicidal risk; enrolled in an in-patient, residential, day treatment or out-patient substance abuse programme within 28 days before signing consent; participation in other substance or mental health treatment  **Diagnostic criteria**  DSM-IV  **Number randomized**  303  **Age**  13-18 years (range)  **Co-morbidities/Co-medications**  Yes, all comorbid substance use disorders (100%), conduct disorders (32%), depression (13%) – Not reported  **ADHD subtypes**  Combined (69%), inattentive (28%), hyperactive/impulsive (3%) | **Arm 1**  MPH-LA high dose+BT (child training): combination of both methylphenidate long acting (72 mg/day) and cognitive-behavioral therapy including adolescent training (described below)  **Arm 2**  BT (child training): participants in both medication groups received manual-standardized, individual cognitive behavior therapy using motivational enhancement approaches throughout the 16 week medication trial. The efficacy and feasibility of training and implementation of the manual-driven BT used in this study has been demonstrated in previous studies  Master’s level CBT therapists were trained and certified by the study’s national trainer (Ms. Klein), who was herself trained and certified as both therapist and trainer by the developer of the manual. All sessions were audio-taped and approximately 5% were selected and independently rated for fidelity and adherence by Ms. Klein. Of 147 sessions rated, 138 (94%) were rated as adherent | **Treatment response** (as a dichotomous outcome)  Clinician-rated Clinical Global Impression Improvement (CGI-I) scale – e.g. responder considered very much improved or much improved (CGI-I ≤ 2)  **All-cause treatment discontinuation** (as a dichotomous outcome)  Yes, reported as study discontinuations  **Tolerability** (as a dichotomous outcome)  Not reported  **Serious adverse events** (as dichotomous outcome)  Yes, reported  **Specific adverse events** (as dichotomous outcome)  Anxiety (as an adverse event e.g. panic/anxiety) |
| Steiner et al., 2011^177^ | **Eligibility criteria**  Children in grades 6, 7, or 8 from 2 middle schools; had a diagnosis of ADHD confirmed by their physician and sufficient English ability to complete assessments and intervention protocols. Both boys and girls were eligible, regardless of their subtype of ADHD or medication use. Exclusion criteria were: coexisting diagnosis of conduct  disorder, pervasive developmental disorder, or other serious mental illness (eg, psychosis).  **Diagnostic criteria**  (ADHD diagnosis confirmed by a physician)  **Number randomized**  41  **Age**  12.4 years (range not reported)  **Co-morbidities/Co-medications**  Not reported – Yes, 66% comedications  **ADHD subtypes**  Not reported | **Arm 1**  Neurofeedback (theta-beta training): this system detects 2 frequency ranges, one in the low-frequency theta range that has been associated with drowsiness (4 to 8 Hz), and another in the high-frequency beta range that has been associated with alert attention (12 to 16 Hz). Three EEG sensors are embedded in a bike helmet, 1 located at the top of the  head, and 2 behind the ears on the straps. During training sessions, children play a simple computer game that involves flying an airplane. Children are told that if they concentrate, the airplane will go up, and if not, the plane will go down. An individual baseline is set at the beginning of each session, and as the children progress they reach higher (more challenging) levels. The computer interface provides children with immediate auditory and visual feedback about the degree to which they are successful in paying attention  **Arm 2**  Control: standard computer format  **Arm 3**  Waiting list (WL): children in the WLC condition were  provided no intervention until after the final postintervention assessment, after which they were invited to complete a course of interventions | **Treatment response** (as a dichotomous outcome)  Not reported/data not abstractable  **All-cause treatment discontinuation** (as a dichotomous outcome)  Yes, reported as study discontinuations  **Tolerability** (as a dichotomous outcome)  Not reported  **Serious adverse events** (as dichotomous outcome)  Not reported  **Specific adverse events** (as dichotomous outcome)  Not reported |
| Wehmeier et al., 2011^178,179^ | **Eligibility criteria**  Children aged 6-12. Exclusion criteria were: previous treatment with ATX, treatment with psychotropic medication other than the study drug, clinically relevant over- and underweight, a  history of bipolar disorder, psychosis, pervasive developmental disorder, seizure disorder (other than febrile seizures), serious suicidal risk, and other relevant acute or unstable medical conditions. Psychotherapy initiated before the study was acceptable  **Diagnostic criteria**  DSM-IV-TR  **Number randomized**  125  **Age**  6-12 years (range)  **Co-morbidities/Co-medications**  Yes, oppositional disorders (31%), conduct disorders (17%), tics (1%) – Not reported  **ADHD subtypes**  Combined (70%), inattentive (22%), hyperactive/impulsive (7%) | **Arm 1**  ATX: atomoxetine 1.2 mg/kg/day  **Arm 2**  Placebo: placebo pill | **Treatment response** (as a dichotomous outcome)  Not reported/data not abstractable  **All-cause treatment discontinuation** (as a dichotomous outcome)  Yes, reported as study discontinuation  **Tolerability** (as a dichotomous outcome)  Yes, reported as study discontinuations due to AEs  **Serious adverse events** (as dichotomous outcome)  Not reported  **Specific adverse events** (as dichotomous outcome)  Loss of appetite/anorexia (as an adverse event) |
| Wilens et al., 2011^180^ | **Eligibility criteria**  Children aged 6-12; rating of 4 or higher on the Clinical Global Impression-ADHD-Severity Scale (CGI-ADHD-S) were enrolled at 10 sites. Exclusion criteria were: current or past diagnosis of bipolar I, II, or NOS (Not Otherwise Specified) disorder; psychotic disorder; autism, Asperger’s syndrome or pervasive developmental disorder; tics or Tourette syndrome; seizure disorder; traumatic brain injury; current diagnosis of obsessive-compulsive disorder, eating disorder, anxiety disorder, or depressive disorder requiring treatment of any kind; psychotropic medications within 14 days or 5 half-lives (7 days for stimulants), whichever was longer, prior to the Day −1 ADHD-RS assessment; in Study 1, ATX within 3 months of randomization or not a suitable candidate to receive ATX. Failure to respond to two or more adequate trials of U.S. Food and Drug  Administration-approved ADHD medication was also exclusionary  **Diagnostic criteria**  DSM-IV  **Number randomized**  278  **Age**  6-12 years (range)  **Co-morbidities/Co-medications**  None – Not reported  **ADHD subtypes**  Combined (80%), inattentive (18%), hyperactive/impulsive (2%) | **Arm 1**  ATX: atomoxetine 1.2 mg/kg/day  **Arm 2**  POZ: pozamicline 0.1-0.7 mg/kg day (excluded)  **Arm 3**  Placebo: placebo pill | **Treatment response** (as a dichotomous outcome)  Not reported/data not abstractable  **All-cause treatment discontinuation** (as a dichotomous outcome)  Yes, reported as study discontinuation  **Tolerability** (as a dichotomous outcome)  Yes, reported as study discontinuations due to AEs  **Serious adverse events** (as dichotomous outcome)  None  **Specific adverse events** (as dichotomous outcome)  None |
| Yildiz et al., 2011^181^ | **Eligibility criteria**  Children aged 8-14 years. Symptom severity at entry was required to be at least 4 points or above as assessed by the Clinical Global Impression-  Severity Scale (CGI-S). Patients who had seizures, bipolar disorder, psychotic illness, mental retardation, pervasive developmental disorder or who were taking concomitant psychoactive medications were excluded from the study. Because anxiety and tic disorders are relative contraindications for the use of OROS-MPH, patients with these conditions were also excluded  **Diagnostic criteria**  DSM-IV-TR  **Number randomized**  29  **Age**  8-14 years (range)  **Co-morbidities/Co-medications**  Yes, oppositional disorders (28%), conduct disorders (24%)/Not reported  **ADHD subtypes**  Combined (84%), inattentive (16%), hyperactive/impulsive (-%) | **Arm 1**  MPH-LA low/medium dose: methylphenidate OROS 1.07 mg/kg/day  **Arm 2**  ATX: atomoxetine 1.3 mg/kg/day | **Treatment response** (as a dichotomous outcome)  Responders based on the achievement of a 40% or greater decrease from baseline to endpoint in the ADHD-RS total score (rater: parents); Clinician-rated Clinical Global Impression Improvement (CGI-I) scale – e.g. responder considered very much improved or much improved (CGI-I ≤ 2)  **All-cause treatment discontinuation** (as a dichotomous outcome)  Yes, reported as study discontinuation  **Tolerability** (as a dichotomous outcome)  Yes, reported as study discontinuation due to AEs  **Serious adverse events** (as dichotomous outcome)  Not reported  **Specific adverse events** (as dichotomous outcome)  Loss of appetite/anorexia (as an adverse event)  Decreased weight (as an adverse event e.g. weight loss)  Insomnia (as an adverse event) |
| Zamora et al., 2011^182^ | **Eligibility criteria**  Children 7-14 years; born at term (≥ 37 weeks of gestation); birth weight > 2500 g. Exclusions: previous diagnosis of a psychiatric disorder or clinically significant chronic medical condition, twins  **Diagnostic criteria**  DSM-IV  **Number randomized**  40  **Age**  7-14 years (range)  **Co-morbidities/Co-medications**  Not reported – Not reported  **ADHD subtypes**  Not reported | **Arm 1**  MPH-SA low/medium dose: methylphenidate (mean dose: 0.3 mg/kg/day)  **Arm 2**  MPH-SA low/medium dose+Zinc: combination of methylphenidate (mean dose: 0.3 mg/kg/day) and zinc sulfate (mean dose: 10 mg/day) | **Treatment response** (as a dichotomous outcome)  Responders based on Conners’ Rating Scale (rater: teachers) with less than 14 points (cut-off)  **All-cause treatment discontinuation** (as a dichotomous outcome)  Yes, reported as study withdrawals  **Tolerability** (as a dichotomous outcome)  Not reported  **Serious adverse events** (as dichotomous outcome)  Not reported  **Specific adverse events** (as dichotomous outcome)  Not reported |
| Assareh et al., 2012^183^ | **Eligibility criteria**  Children aged 6-12 years; ADHD-RS > 20. Exclusion criteria: any psychiatric disorder, except for oppositional defiant disorder (ODD) and learning disability (LD); IQ less than 70; use of any psychotropic substance, opioid, or other drugs affecting central nervous system in two previous weeks; any significant neurologic disease; and use of any combination containing PUFAs more than  once weekly were excluded from the study  **Diagnostic criteria**  DSM-IV  **Number randomized**  40  **Age**  6-12 years (range)  **Co-morbidities/Co-medications**  Yes, oppositional disorder (53%)/Yes (all received stimulants)  **ADHD subtypes**  Not reported | **Arm 1**  MPH-SA low/medium dose: methylphenidate 0.3-1.0 mg/kg/day + placebo  **Arm 2**  MPH-SA low/medium dose+PUFA: methylphenidate 0.3-1.0 mg/kg/day + polyunsaturated fatty acid (430 mg/day omega-3/6 fatty acid: docosahexaenoic acid 241 mg/day, 33 mg/day EPA and 180 mg/day omega-6) | **Treatment response** (as a dichotomous outcome)  Responders based on the achievement of a > 25% decrease from baseline to endpoint in the ADHD-RS-IV-Parent:Inv total score (rater: parents)  **All-cause treatment discontinuation** (as a dichotomous outcome)  Not reported  **Tolerability** (as a dichotomous outcome)  Not reported  **Serious adverse events** (as dichotomous outcome)  Not reported  **Specific adverse events** (as dichotomous outcome)  Not reported |
| Duric et al., 2012^184^ | **Eligibility criteria**  Children and adolescents aged 6-18 years; IQ>70.  **Diagnostic criteria**  ICD-10  **Number randomized**  130  **Age**  6-18 years (range)  **Co-morbidities/Co-medications**  Not reported – Not reported  **ADHD subtypes**  Not reported | **Arm 1**  MPH-SA low/medium dose: methylphenidate 1 mg/kg/day  **Arm 2**  Neurofeedback (theta-beta training): a beta/theta neurofeedback (NF) treatment protocol for ADHD was  used, including the appropriate frequency ranges for the EEGs and electrode placements. Beta enhancement (16–20 HZ) and Theta suppression (4–7 Hz) were assessed. The treatment site used was in the central area and unipolar electrodes was placed on the Cz, whereas the ground electrode was placed on the earlobe. NF was conducted three times a week with 30 treatments for each child and adolescent. Each treatment lasted 40 minutes and was separated into pre and post 5-minute baseline periods (alpha training) and 30 minutes of NF (beta/theta training). Theta activity was defined as 4–7 Hz, alpha activity as 8–12 Hz, SMR activity as 13–15 Hz, beta activity as 16–20 Hz, and electromyography (EMG) activity as 80–150 Hz activity. The events above these threshold levels were monitored. The standards used in the treatment were to decrease theta activity by inhibiting high amplitude theta activity or by rewarding high amplitude beta activity. The treatment effect was defined as increased beta/SMR activity of 13–20 Hz, decreased theta activity of 4–7 Hz, and decreased EMG activity  **Arm 3**  MPH-SA low/medium dose+neurofeedback: combination of methylphenidate 1 mg/kg/day and theta-beta training neurofeedback as described above | **Treatment response** (as a dichotomous outcome)  Not reported/data not abstractable  **All-cause treatment discontinuation** (as a dichotomous outcome)  Yes, reported as study drop-outs  **Tolerability** (as a dichotomous outcome)  Not reported  **Serious adverse events** (as dichotomous outcome)  Not reported  **Specific adverse events** (as dichotomous outcome)  Not reported |
| Fabiano et al., 2012^185^ | **Eligibility criteria**  Children aged 6-12 years. Participants were excluded from the study if the child had an estimated IQ below 80, psychosis, or pervasive developmental disorder. The child and parent also had to be able to speak and understand English.  **Diagnostic criteria**  DSM-III-R  **Number randomized**  55  **Age**  6-12 years (range)  **Co-morbidities/Co-medications**  Yes, oppositional disorder (70%), conduct disorders (6%) – Yes, 54%  **ADHD subtypes**  Not reported | **Arm 1**  BT (parent training): BT included parent training. The COACHES program is an eight-week, two-hour behavioral parent training program. During the first hour, fathers learned how to implement effective parenting strategies in a group setting through homework review, viewing videotapes of parenting errors, discussing and identifing the errors, and generating solutions. Further, the group facilitator (a clinical psychologist) modeled the use of the parenting  strategy consistent with the COPE manual. Parent training topics included: (1) Constructing a home-based daily report card and reward system; (2)  Attending to positive behavior; (3) Ignoring minor inappropriate behavior; (4) Issuing effective commands; (5) Using “When-Then” contingencies and transitional warnings; (6) Using time out; (7) Problem-solving; and (8) Planning for maintenance. Concurrently, children practiced soccer skill drills with undergraduate counselors using procedures for teaching sport skill competencies combined with a contingency management approach for  appropriate behavior.  Then, during the second hour, the parent and child groups joined together for a soccer game. The soccer game provided a context for the fathers to interact with their children and practice the parenting strategies taught in the classroom (e.g., praise, using effective commands), and for clinicians to provide feedback to the fathers. For example, during the week emphasizing attending to positive behavior, a father was asked to issue at least five, labeled praise statements to his child each quarter of the game. Following each quarter, fathers and the group facilitator met to review progress. Fathers were assigned weekly homework assignments to review the week’s material with their partner (if present) and practice the techniques with their child during the week. During week 8, the structure of the second hour was modified to include a father versus child soccer game followed by a trophy presentation.  **Arm 2**  Waiting list (WL): Fathers assigned to the waitlist group were evaluated eight weeks later and again for a one-month follow-up. Following the one-month follow-up evaluation, families assigned to the waitlist condition enrolled in the COACHES program | **Treatment response** (as a dichotomous outcome)  Responders rated by parents – Eyberg Child Behavior Inventory (ECBI) < 60  **All-cause treatment discontinuation** (as a dichotomous outcome)  Yes, reported as discontinuations  **Tolerability** (as a dichotomous outcome)  Not reported  **Serious adverse events** (as dichotomous outcome)  Not reported  **Specific adverse events** (as dichotomous outcome)  Not reported |
| Green et al., 2012^186^ | **Eligibility criteria**  Children 7-14 years; T-score of at least 65 on any of the ADHD CPRS-R; no history of mental retardation (i.e., IQ > 70). Exclusion criteria were: severe mental illness (i.e., psychotic, bipolar, or major depressive disorder, by history or clinical interview) or autism spectrum disorders  **Diagnostic criteria**  DSM-IV-TR  **Number randomized**  50  **Age**  7-14 years (range)  **Co-morbidities/Co-medications**  Yes, learning disability (4%) – Yes , 38%  **ADHD subtypes**  Combined (42%), inattentive (50%), hyperactive/impulsive (8%) | **Arm 1**  WM training: Cognitive training – working memory training. 90 trials of WM woth Cogmed tasks a day for 3-4 weeks (25 days). The Cogmed training includes 10 verbal and visuospatial WM span tasks. Some tasks are both auditory and visual in nature, requiring cross-modal processing. During each session, participants choose 6 of 8 presented tasks. During the active training condition, for each task, the difficulty level automatically adjusts to match the WM span of the child. The difficulty level gradually increased when the child answered correctly on consecutive trials and decreased when the child answered incorrectly on consecutive trials. The tasks require good attention and tracking ability, as some stimuli move on the screen. In the placebo control-nonadaptive condition,  the same tasks were used, but the difficulty level  remained low (2 or 3 numbers/items at maximum) throughout all of the training sessions. These similar versions controlled for nonspecific effects of the training procedure and enabled us to attribute any improvement in ADHD-related behaviors and WM exclusively, due to the effect of training WM  Coaching was used to enhance compliance with completing the sessions for both the placebo and training condition. The same licensed clinical psychologist coached all participants during each week of training on the telephone at least once a week. Coaching involved answering questions regarding the use of the computer program and troubleshooting software issues, general feedback for the use of the  program, and addressing parental concerns of how to engage their child in the training protocol. Coaching was kept to a minimum so as to reduce any possible differences between groups in amount or type of feedback. The coach had access via the internet to the frequency of use and performance on the computer tasks  **Arm 2**  Placebo: placebo condition (nonadaptive) | **Treatment response** (as a dichotomous outcome)  Not reported/data not abstractable  **All-cause treatment discontinuation** (as a dichotomous outcome)  Yes, reported as study withdrawals  **Tolerability** (as a dichotomous outcome)  Not reported  **Serious adverse events** (as dichotomous outcome)  Not reported  **Specific adverse events** (as dichotomous outcome)  Not reported |
| Jafarinia et al., 2012^187^ | **Eligibility criteria**  Children aged 6–17 years; total and/or subscale scores on ADHD-RS-IV School Version of at least 1.5 SD above norms for patient’s age and gender. Exclusion criteria were: psychiatric comorbidities (excluding oppositional defiant disorder), high risk of suicide, mental retardation (IQ 70), clinically important chronic medical condition (such as epilepsy and organic brain disorders), drug abuse or dependence in the last 6months, hypertension  or hypotension, history of allergy to BUP or  MPH, abnormal electrocardiogram, and psychotropic medication use in the last 14 days  **Diagnostic criteria**  DSM-IV-TR  **Number randomized**  44  **Age**  6-17 years (range)  **Co-morbidities/Co-medications**  None/Not reported  **ADHD subtypes**  Not reported | **Arm 1**  MPH-SA low/medium dose: methylphenidate 20-30 mg/day  **Arm 2**  BUP: bupropion 100-150 mg/day | **Treatment response** (as a dichotomous outcome)  Responders based on the achievement of a 50% or greater decrease from baseline to endpoint in the ADHD-RS total score (rater: parents and teachers)  **All-cause treatment discontinuation** (as a dichotomous outcome)  Yes, reported as study discontinuation  **Tolerability** (as a dichotomous outcome)  Not reported  **Serious adverse events** (as dichotomous outcome)  Not reported  **Specific adverse events** (as dichotomous outcome)  Loss of appetite/anorexia (as an adverse event)  Anxiety (as an advserse event e.g. anxiety/nervousness)  Cardiovascular event – tachycardia (as an adverse event) |
| Manor et al., 2012^188,189^ | **Eligibility criteria**  Children aged 6-13; score of at least 1.5 SD above the normal for the patient’s age and gender in the Teacher-rated ADHD Rating  Scale-IV School Version; a score of 4 or higher (moderately ill or worse) in the Clinical  Global Impression of Severity of Illness (CGI-S) test. Exclusion criteria were: girls who reached menarche and presented with three previous  regular menstrual cycles; history or current diagnosis of any serious systemic (e.g.,  diabetes, hyper/hypothyroidism) or neurological condition (e.g., epilepsy, brain tumor); failure to respond to two or more adequate courses of stimulant therapy (among those previously treated children); pervasive developmental disorder or nonverbal learning disability; diagnosed with psychotic disorders (e.g., schizophrenia)  according to the DSM-IV axis; any evidence of suicidal risk or any current psychiatric comorbidity that required psychiatric pharmacotherapy; concomitant use of prescription or nonprescription agents with potent psychotropic properties, including ADHD treatments and dietary supplements, 4-week prior to the study entry; history of alcohol or substance abuse as defined by DSM-IV criteria; consumption of > 250 mg/day of caffeine; history of allergic reactions or sensitivity to marine products, soy, or corn as well as any illness that could jeopardize the participant’s health or limit their successful completion of the trial  **Diagnostic criteria**  DSM-IV  **Number randomized**  200  **Age**  6-13 years (range)  **Co-morbidities/Co-medications**  Yes, oppositional disorders (9%), anxiety (3%), tic disorders (3%)/No  **ADHD subtypes**  Not reported | **Arm 1**  PUFA: polyunsaturated fatty acid (omega-3 fatty acid: 120 mg eicosapentaenoic acid and docosahexaenoic acid + 30 mg phosphatidylserine)  **Arm 2**  Placebo: placebo pill | **Treatment response** (as a dichotomous outcome)  Not reported/data not abstractable  **All-cause treatment discontinuation** (as a dichotomous outcome)  Yes, reported as study drop-outs  **Tolerability** (as a dichotomous outcome)  Yes, reported as study drop-outsdue to AEs  **Serious adverse events** (as dichotomous outcome)  None  **Specific adverse events** (as dichotomous outcome)  Loss of appetite/anorexia (as an adverse event)  Insomnia (as an adverse event)  Anxiety (as an adverse event)  Cardiovascular event – tachycardia (as an adverse event leading to discontinuation) |
| Perera et al., 2012^190^ | **Eligibility criteria**  Children aged 6-12 years  **Diagnostic criteria**  DSM-IV  **Number randomized**  98  **Age**  6-12 years (range)  **Co-morbidities/Co-medications**  Yes, concomitant physical illness (19%)/Yes (all received stimulants)  **ADHD subtypes**  Not reported | **Arm 1**  MPH-SA low/medium dose: methylphenidate 0.7-1.0 mg/kg/day + placebo  **Arm 2**  MPH-SA low/medium dose+PUFA: methylphenidate 0.7-1.0 mg/kg/day + polyunsaturated fatty acid (omega-3/6 fatty acid: 296.4 mg omega-3 and 180.8 mg omega-6) | **Treatment response** (as a dichotomous outcome)  Not reported/data not abstractable  **All-cause treatment discontinuation** (as a dichotomous outcome)  Yes, reported as study discontinuations  **Tolerability** (as a dichotomous outcome)  Not reported  **Serious adverse events** (as dichotomous outcome)  Not reported  **Specific adverse events** (as dichotomous outcome)  Not reported |
| Wilens et al., 2012^191-194^ | **Eligibility criteria**  Children and adolescents aged 6-17; ADHD with a suboptimal response to an extended-release oral preparation of MPH or AMPH. Suboptimal response was defined as follows: ≥4 weeks of a stable dose of treatment with an extended-release psychostimulant with improvement but continued mild to moderate symptoms of ADHD; ADHD-RS-IV total score of ≥24 and a Clinical Global Impressions of Severity of Illness (CGI-S) indicative of at least mild impairment (≥3); and investigator assessment of inadequate response to current psychostimulant. Exclusion criteria were: lack of response to current psychostimulant medication, the presence of cardiovascular abnormalities, body weight of <55 or >176 lb, or any current, controlled or uncontrolled, comorbid psychiatric diagnosis (except oppositional defiant disorder), including any severe comorbid Axis II disorders or severe Axis I disorders  **Diagnostic criteria**  DSM-IV-TR  **Number randomized**  461  **Age**  6-17 (range)  **Co-morbidities/Co-medications**  None/All with stimulants  **ADHD subtypes**  Not reported | **Arm 1**  MPH-LA or LDX-LA + GUAN-LA: combination of stimulants (MPH OROS and LDX were the most frequent among other stimulants) and guanfacine long acting (3.2 mg/day)  **Arm 2**  MPH-LA or LDX-LA: stimulants (Concerta® 42 mg/day or Vyvanse 52 mg/day were the most frequent among other stimulants) | **Treatment response** (as a dichotomous outcome)  Responders were defined posthoc as patients with a ≥ 40% reduction in ADHD-RS total score from baseline (rater: clinician/investigator); Clinician-rated Clinical Global Impression Improvement (CGI-I) scale – e.g. responder considered very much improved or much improved (CGI-I ≤ 2); Clinician-rated Clinical Global Impression Severy (CGI-S) scale – e.g. GGI-S ≤ 3; the Parent Global Assessment for improvement (PGA)  **All-cause treatment discontinuation** (as a dichotomous outcome)  Yes, reported as study early terminations  **Tolerability** (as a dichotomous outcome)  Yes, reported as study early terminations due to AEs  **Serious adverse events** (as dichotomous outcome)  Yes, reported  **Specific adverse events** (as dichotomous outcome)  Loss of appetite/anorexia (as an adverse event)  Insomnia (as an adverse event)  Syncope (as an adverse event) |
| Abikoff et al., 2013^195^ | **Eligibility criteria**  Children aged 8-11 years; in 3rd–5th grade; IQ ≥ 85 on the WASI; elevated ADHD ratings on the Revised Conners Parent and Teacher Rating Scales; OTMP entry criteria required factor or Total scores > 1 SD above age and sex norms on the teacher-completed Children’s Organizational Skills Scale (COSS-T), or on the parent COSS (COSS-P) Total score, and the teacher or parent had to indicate that OTMP problems were functionally  interfering. Exclusion criteria were: attending an all-day special education classroom; having an Individualized Educational Plan or paraprofessional aide to help specifically with organization; diagnosis of PDD, psychosis, bipolar disorder, PTSD, or any other condition judged to contra-indicate participation; or currently receiving behavioral treatment for ADHD. Children with diagnoses of conduct disorder, oppositional defiant disorder, anxiety or mood disorders were not excluded  **Diagnostic criteria**  DSM-IV  **Number randomized**  158  **Age**  8-11 years (range)  **Co-morbidities/Co-medications**  Yes, enuresis (5%), tic disorders (4%), learning disability (3%) – Yes, 35%  **ADHD subtypes**  Combined (44%), inattentive (56%), hyperactive/impulsive (14%) | **Arm 1**  BT (child, parent and teacher training): Behavioral therapy using a daily report card with child, parent and teacher training with OST or PATHKO. Both treatments are individual and target organizational functioning, but rely on different procedures consonant with their underlying theoretical models. Treatments total 20 hour long in clinic sessions held twice-a-week after school. (Initial OST treatment development  efforts using once-weekly sessions yielded limited benefits. Two sessions a week provided  more opportunities for practice and feedback, increased children’s engagement and  improved their OTMP functioning. PATHKO was designed to match the frequency of OST  sessions.) The treatments are fully manualized with detailed instructions to therapists  regarding session goals, scripts and procedures used to address these goals, and betweensession  “homework” assignments for participants. For example, PATHKO motivates children by training teachers and parents to establish specific, individualized goals for children on written charts completed daily and to prompt, monitor, and praise/reward children for achieving these goals. Sessions primarily involve parents, with children coming in briefly at the end of every session. The three core components of PATHKO are: 1) Daily Report Cards (DRC) targeting end-point OTMP behaviors e.g., “assignments completed on time”, “desk/cubby is neat and organized”, where teachers monitor the behaviors at school and parents provide points at home, 2) Token Economy System, in which children receive points for achieving goals at home (e.g., “brings home all materials needed to do homework, backpack packed by bedtime”) and on their DRC, and exchange the points for privileges and rewards on a daily and weekly basis, and 3) Homework Rules and Structures, in which parents establish and reward children’s adherence to rules regarding completing homework. Underlying each of these components is an emphasis on Parent-Teacher Collaboration and procedures to facilitate sustainability  **Arm 2**  Waiting list (WL): waitlist control | **Treatment response** (as a dichotomous outcome)  Parent or teacher-rated Clinical Global Impression Improvement (CGI-I) scale – e.g. responder considered very much improved or much improved (CGI-I ≤ 2)  **All-cause treatment discontinuation** (as a dichotomous outcome)  Yes, reported as drop-outs/withdrawals  **Tolerability** (as a dichotomous outcome)  Yes, reported as drop-outs/withdrawals due to AEs  **Serious adverse events** (as dichotomous outcome)  Not reported  **Specific adverse events** (as dichotomous outcome)  Anxiety (as an adverse event leading to discontinuation) |
| Arnold et al., 2013^196^ | **Eligibility criteria**  Children aged 6-12; an item-mean score of ≥1.5 on a 0-3 metric was required on the parent/teacher SNAP Rating Scales-IV (30). Exclusion criteria were: IQ <80, mental age < 6, comorbid disorder requiring psychoactive medication, medical disorder requiring medication that had psychoactive effects, >5 previous NF treatments, antipsychotic medication within 6 months pre baseline, FLUOX/ATX 4-weeks pre-baseline, stimulant 1-week pre-baseline, or any other psychotropic medication 2-weeks pre-baseline  **Diagnostic criteria**  DSM-IV  **Number randomized**  39  **Age**  6-12 years (range)  **Co-morbidities/Co-medications**  Not reported – Not reported  **ADHD subtypes**  Combined (67%), inattentive (33%) | **Arm 1**  Neurofeedback (theta-beta training): the neurofeedback (NF) system was administered via a single channel CZ electrode placement and a reference electrode on each ear, with feedback to decrease theta/alpha and increase beta,including sensorimotor rhythm (SMR) EEG activity. Elements of NF were provided via a commercially available training device, Smartbrain by Cyberlearning Technology, LLC (www.smartbraintech.com. This device utilized off theshelf videogames (Sony PlayStation & MS Xbox) via use of an interface which modulated input to the videogame hand-controller based on EEG activity. It was selected because: it was the most frequently used technology in RCTs of NF for ADHD at the time of the grant application in 2007, and was the only device used in a sham-controlled small RCT; it was developed from NASA technology with some supporting science behind it; and it was interactive, engaging, and, unlike many other devices, did not require a “NF coach” to guide trainees. In the active condition, children used a game controller for normal gaming functions but its responsiveness (speed, control & vibration) was contingent on the child’s real-time EEG activity. Reinforcement was provided for EEG theta-beta power ratio below a threshold that was set minute-to-minute by fuzzy logic based on the immediately preceding EEG  **Arm 2**  Placebo: placebo (sham-control) neurofeedback appeared identical to the active condition in all aspects (equipment, duration, frequency, & videogame choices) except the interface module was pre-programmed to give random feedback not contingent on the child’s EEG. To blind staff to treatment condition, The SmartBox interface devices were independently pre-programmed by an off-site consultant who had no interaction with participants or data (analogous to prepackaged randomized medication) | **Treatment response** (as a dichotomous outcome)  Not reported/data not abstractable  **All-cause treatment discontinuation** (as a dichotomous outcome)  Yes, reported as study drop-outs  **Tolerability** (as a dichotomous outcome)  Not reported  **Serious adverse events** (as dichotomous outcome)  Not reported  **Specific adverse events** (as dichotomous outcome)  Not reported |
| Coghill et al., 2013^197-200^ | **Eligibility criteria**  Children and adolescents aged 6-17; had at least moderate severity as defined by a baseline ADHD-RS total score of 28 or higher; age-appropriate intellectual functioning; blood pressure measurements within the 95^th^ percentile for age, sex, and height; and ability to swallow a capsule; girls of childbearing potential had to have a negative urine pregnancy test at baseline and to comply with any contraceptive requirements of the protocol. Exclusion criteria were: failure to respond to previous OROS-MPH therapy; presence of a comorbid psychiatric disagnsosi with significant symptoms; conduct disorder (exclusing oppositional defiant disorder); pregnancy or lactation; weight below 22.7 kg; body mass index greater than the 97^th^ percentile for age and sex; positive urine drug test; clinically significant electrocardiogram or laboratory abnormalies; suspected substance abuse or dependence disorder (excluding nicotine) within the previous 6 months; history of seizures; tics or Tourette’s disorder; known structural cardiac abnormality; or any other condition that might increase vulnerability to the sympathomimetic effects of a stimulant drug. Patients whose current ADHD medications provided effective control of symptoms with acceptable tolerability were also excluded  **Diagnostic criteria**  DSM-IV-TR  **Number randomized**  336  **Age**  6-17 years (range)  **Co-morbidities/Co-medications**  Yes, any comorbid psychiatric diagnosis (20%), oppositional disorders (8%) – Not reported  **ADHD subtypes**  Combined (0.3%), inattentive (81%), hyperactive/impulsive (3%) | **Arm 1**  LDX-LA: lisdexamphetamine dimesylate 30, 50 or 70 mg/day (mean dose: 53.8 mg/day)  **Arm 2**  MPH-LA high dose: methylphenidate OROS® 18, 36 or 59 mg/day (mean dose: 45.4 mg/day)  **Arm 3**  Placebo: placebo pill | **Treatment response** (as a dichotomous outcome)  Responders based on the achievement of a 25% decrease from baseline to endpoint in the ADHD-RS total score (rater: investigator/clinician); Clinician-rated Clinical Global Impression Improvement (CGI-I) scale – e.g. responder considered very much improved or much improved (CGI-I ≤ 2)  **All-cause treatment discontinuation** (as a dichotomous outcome)  Yes, reported as study discontinuations  **Tolerability** (as a dichotomous outcome)  Yes, reported as study discontinuations due to AEs  **Serious adverse events** (as dichotomous outcome)  Yes, reported  **Specific adverse events** (as dichotomous outcome)  Loss of appetite/anorexia (as an adverse event)  Decreased weight (as an adverse event e.g. loss ≥ 7% from baseline)  Insomnia (as an adverse event)  Sleep disturbances (as an adverse event)  Syncope (as an adverse event)  Cardiovascular event – QT prolongation (as an adverse event) |
| Dittmann et al., 2013^201-204^ | **Eligibility criteria**  Children and adolescents aged 6-17; an inadequate response to previous MPH treatment; had at least moderate severity as defined by a baseline ADHD-RS total score of 28 or higher; age-appropriate intellectual functioning; blood pressure measurements within the 95^th^ percentile for age, sex, and height; and ability to swallow a capsule; girls of childbearing potential had to have a negative urine pregnancy test at baseline and to comply with any contraceptive requirements of the protocol. Exclusion criteria were: presence of a comorbid psychiatric disagnosis with significant symptoms; conduct disorder (exclusing oppositional defiant disorder); pregnancy or lactation; weight below 22.7 kg; body mass index greater than the 97^th^ percentile for age and sex; positive urine drug test; clinically significant electrocardiogram or laboratory abnormalies; known CYP2D6 poor-metabolizer genotype; suspected substance abuse or dependence disorder (excluding nicotine) within the previous 6 months; history of seizures; tics or Tourette’s disorder; pre-existing liver disease or laboratory evidence of liver disease; known structural cardiac abnormality; or any other condition that might increase vulnerability to the sympathomimetic effects of a stimulant drug  **Diagnostic criteria**  DSM-IV-TR  **Number randomized**  267  **Age**  6-17 years (range)  **Co-morbidities/Co-medications**  Yes, any comorbid psychiatric diagnosis (19%), oppositional disorders (10%) – Not reported  **ADHD subtypes**  Combined (80%), inattentive (17%), hyperactive/impulsive (3%) | **Arm 1**  LDX-LA: lisdexamphetamine dimesylate 30, 50 or 70 mg/day (mean dose: 52.5 mg/day)  **Arm 2**  ATX: atomoxetine 0.5-1.2 mg/kg/day (mean dose: 40.2 mg/day) | **Treatment response** (as a dichotomous outcome)  Responders based on the achievement of a 25% decrease from baseline to endpoint in the ADHD-RS total score (rater: investigator/clinician); Clinician-rated Clinical Global Impression Improvement (CGI-I) scale – e.g. responder considered very much improved or much improved (CGI-I ≤ 2); Clinician-rated Clinical Global Impression Severity (CGI-S) scale – e.g. responder considered normal, not at all ill or borderline mentally ill  **All-cause treatment discontinuation** (as a dichotomous outcome)  Yes, reported as study discontinuations  **Tolerability** (as a dichotomous outcome)  Yes, reported as study discontinuations due to AEs  **Serious adverse events** (as dichotomous outcome)  None  **Specific adverse events** (as dichotomous outcome)  Loss of appetite/anorexia (as an adverse event)  Decreased weight (as an adverse event e.g. loss ≥ 7% from baseline)  Insomnia (as an adverse event)  Cardiovascular event – blood pressure increased (e.g. increase of SBP)  Decreased pulse values (e.g. decrease of at least 50 bpm)  Increased pulse values (e.g. increase of at least 100 bpm) |
| Hovik et al., 2013^205,206^ | **Eligibility criteria**  Children aged 10-12 years;  **Diagnostic criteria**  ICD-10  **Number randomized**  75  **Age**  10-12 years (range)  **Co-morbidities/Co-medications**  None – Yes, 70%  **ADHD subtypes**  Not reported | **Arm 1**  WM training: Cognitive training – working memory training. The WM training program for the treatment group consisted of 13 different PC-based exercises (CogMed). The WM training program includes three letter span tasks (all forward condition), three digit span tasks (one forward condition, two backward conditions), and seven visuospatial tasks (all forward  sequenced), including static visuospatial tasks (one 2D visuospatial task, one 3D visuospatial task), and two dynamic visuospatial tasks, in which students recall the positions of rotated or moving objects. Nine of the tasks are presented purely in visual format, and four  are delivered with an auditory input. Eleven of the tasks are forward sequenced, while only two are reverse-order tasks. A critical feature of the program is adaptivity, i.e. the level of difficulty is adjusted continuously to the individual student’s skill level throughout the training program. The training group completed 10–15 trials of eight exercises each day for a total of 115 WM trials per day. Training time averaged about 30–40 minutes per day, depending on the exercise set and the student’s performance level. Training participants agreed in advance with the person administering the training program on a reward schedule for achieving training targets. They received daily verbal and visual feedback about increases in performance and were rewarded after training by being allowed to play the RoboRacing-computer game. Every fifth day participants received an additional individualized reward agreed to by the school administration. Parents were instructed not to provide any rewards. The training program has a built-in compliance measure, which is calculated by subtracting the Start Index (results of day 2 and 3)  from the Max Index (results from the two best training days)  **Arm 2**  Control: treatment as usual | **Treatment response** (as a dichotomous outcome)  Not reported/data not abstractable  **All-cause treatment discontinuation** (as a dichotomous outcome)  Yes, reported as study discontinuations  **Tolerability** (as a dichotomous outcome)  Not reported  **Serious adverse events** (as dichotomous outcome)  Not reported  **Specific adverse events** (as dichotomous outcome)  Not reported |
| Li et al., 2013^207^ | **Eligibility criteria**  Children and adolescents aged 7-16 years. Exclusion criteria were: younger than 7 years old; afflicted by severe mental illness; showed predominantly fast β waves upon EEG examination; or IQ <80  **Diagnostic criteria**  DSM-IV  **Number randomized**  64  **Age**  7-16 years (range)  **Co-morbidities/Co-medications**  Not reported – Not reported  **ADHD subtypes**  Combined (30%), inattentive (66%), hyperactive/impulsive (5%) | **Arm 1**  MPH-SA low/medium dose+neurofeedback: combination of methylphenidate 5-10 mg initial dose and theta-beta training neurofeedback. All training was performed on an Autogenic A620 EEG feedback therapeutic apparatus (Rayfield Technology Inc.,  USA). For EEG feedback training, the 4-8 Hz θ wave was suppressed while 12-15 Hz SMR was strengthened. For non-feedback attention training, the threshold was set to non-feedback status. Instructions and game sequences were unified. Patients received the training 2 to 5 times per week and each training session lasted 25 to 35 minutes  **Arm 2**  MPH-SA low/medium dose: methylphenidate 5-10 mg/day initial dose (up to 60 mg) | **Treatment response** (as a dichotomous outcome)  Not reported/data not abstractable  **All-cause treatment discontinuation** (as a dichotomous outcome)  Yes, reported as study drop-outs  **Tolerability** (as a dichotomous outcome)  Not reported  **Serious adverse events** (as dichotomous outcome)  Not reported  **Specific adverse events** (as dichotomous outcome)  Not reported |
| Newcorn et al., 2013^208-211^ | **Eligibility criteria**  Children aged 6-12 years; a baseline ADHD-RS-IV total score ≥28 and a Clinical Global Impressions–Severity of Illness Scale score ≥4. Exclusion criteria were: any current controlled or uncontrolled comorbid psychiatric diagnosis (except oppositional defiant disorder), including any severe comorbid Axis II disorders or Axis I disorders (e.g., posttraumatic stress disorder, bipolar illness, psychosis, pervasive developmental disorder, obsessive compulsive disorder, substance abuse disorder, or other symptomatic manifestations) that could confound efficacy or safety assessments, or for which GUAN-LA treatment might be contraindicated; at risk for suicide currently or in the past; history or presence of cardiac abnormalities or a primary sleep disorder; body weight <55 lbs or body mass index >95th percentile; and use of another investigational product within 30 days of baseline  **Diagnostic criteria**  DSM-IV-TR  **Number randomized**  340  **Age**  6-12 years (range)  **Co-morbidities/Co-medications**  No/No  **ADHD subtypes**  Combined (96%), inattentive (2%), hyperactive/impulsive (2%) | **Arm 1**  GUAN-LA: guanfacine long acting 1-4 mg/day  **Arm 2**  Placebo: placebo pill | **Treatment response** (as a dichotomous outcome)  Response was defined as a ≥30 % reduction  in ADHD-RS-IV Total score from baseline and a CGI-I  score of 1 or 2 at final on-treatment assessment; Global improvement based on Clinical Global Impression Improvement (CGI-I) scale. Subjects with improvement of “very much” or “much” improved were considered responders e.g. GGI-I ≤ 2 (rating evaluations reported by clinician; Global improvement based on Clinical Global Impression Severy (CGI-S) scale e.g. CGI-S ≤ 3  **All-cause treatment discontinuation** (as a dichotomous outcome)  Yes, reported as early termination  **Tolerability** (as a dichotomous outcome)  Yes, reported as early termination due tu AEs  **Serious adverse events** (as dichotomous outcome)  Yes, reported  **Specific adverse events** (as dichotomous outcome)  Loss of appetite/anorexia (as an adverse event)  Insomnia (as an adverse event)  Syncope (as an adverse event) |
| Ghanizadeh et al., 2013^212^ | **Eligibility criteria**  Children and adolescents aged 5-16 years. Exclusion criteria were: self-reported allergic reaction to folic acid; kidney disease; estimated mental retardation and mild pervasive developmental disorder; being on dialysis, infection; anemia; being alcoholic; epilepsy; taking medications such as phenytoin, methotrexate, nitrofurantoin, tetracycline; barbiturates such as phenobarbital; and antieplielptic medication such as phenytoin or primidone; a diagnosed psychotic disorder and mood disorder; other conditions that preclude participation (or increase risk) in the clinical trial (Type 1 diabetes mellitus; metabolic diseases, gastro-intestinal disorders affecting nutrient absorption, cancer); extensive use of nutritional folic acid supplements within the previous 3 months; and behavior therapy or any other psychotherapy in the last three months or during this study  **Diagnostic criteria**  DSM-IV-TR  **Number randomized**  49  **Age**  5-16 years (range)  **Co-morbidities/Co-medications**  None – Not reported  **ADHD subtypes**  Not reported | **Arm 1**  MPH-SA low/medium dose+vitamins: methylphenidate 10-20 mg/day plus vitamin B9 (folic acid) 5 mg/day  **Arm 2**  MPH-SA low/medium dose: methylphenidate 10-20 mg/day | **Treatment response** (as a dichotomous outcome)  Not reported/data not abstractable  **All-cause treatment discontinuation** (as a dichotomous outcome)  Yes, reported as study discontinuations/lost follow-up  **Tolerability** (as a dichotomous outcome)  Yes, reported as study discontinuatios due to AEs  **Serious adverse events** (as dichotomous outcome)  Yes, reported  **Specific adverse events** (as dichotomous outcome)  Loss of appetite/anorexia (as adverse events leading to discontinuation) |
| Oberai et al., 2013^213^ | **Eligibility criteria**  Children aged 6 to 15 years. Exclusion criteria were: chronic physical or neurological disorder, history of drug abuse, seizure, Tic disorder, Tourette syndrome, severely ill patient requiring  hospitalisation and patients who were on anti -ADHD or psychoactive medications in the previous two weeks  **Diagnostic criteria**  DSM-IV  **Number randomized**  61  **Age**  6-15 years (range)  **Co-morbidities/Co-medications**  None – Not reported  **ADHD subtypes**  Not reported | **Arm 1**  HOMEO: homeopathy including 9 different remedies including *Calcarea carbonicum* (*n* = 8), *Lycopodium*  (*n* = 6), *Phosphorus* (*n* = 5), *Hyoscyamus* (*n* = 2), *Sulphur* (*n* = 2), *Belladonna* (*n* = 1), *Argentum nitricum* (*n* = 1), *Natrum muriaticum* (*n* = 1) and *Pulsatilla* (*n* = 1). Individualised homoeopathic medicine was customised to each patient (started with 0/1 potency).  **Arm 2**  Placebo: placebo pill | **Treatment response** (as a dichotomous outcome)  Not reported/data not abstractable  **All-cause treatment discontinuation** (as a dichotomous outcome)  Yes, reported as study drop-outs  **Tolerability** (as a dichotomous outcome)  Not reported  **Serious adverse events** (as dichotomous outcome)  Not reported  **Specific adverse events** (as dichotomous outcome)  Not reported |
| Ogrim et al., 2013^213^ | **Eligibility criteria**  Children and adolescents aged 7-16 years. Patients with IQ and adaptive levels <70 were excluded. Subjects with learning disabilities and behavioral and emotional comorbidities were included, as was one patient with both ADHD and Asperger’s syndrome. None of the patients were on or had been treated with stimulants when evaluated for diagnostic assessment, and none of them had received psychotherapy  **Diagnostic criteria**  DSM-IV  **Number randomized**  32  **Age**  7-16 years (range)  **Co-morbidities/Co-medications**  Yes, learning disability (59%), oppositional disorder or conduct disorders (45%), depression or anxiety (21%) – Not reported  **ADHD subtypes**  Combined (76%), inattentive (24%) | **Arm 1**  MPH-LA or DEXAM any dose: methylphenidate long acting 30-90 mg/day or dexamphetamine 5-10 mg/day  **Arm 2**  Neurofeedback (theta-beta training): 30 sessions of neurofeedback (NF). The NF training was performed using Brain Tuner Version 1.4 from Mitsar. Training protocols were individually selected, combining behavioral information and data from the QEEG. Excess theta, compared with norms, led to a theta/beta protocol, rewarding 13–20 Hz and inhibiting 4–8 Hz, with the active electrodes at parietal, central, or frontal midline (Pz, Cz, or Fz, respectively), depending upon the individual QEEG. In some cases, minor changes in band ranges were made; inhibiting 3–8 Hz, for example. Authors found significant deviances in QEEG, such as excess occipital alpha, which were probably unrelated to ADHD. In accordance with principles of QEEG-based neurofeedback, authors reasoned that training deviances toward normality might produce improvements in behavior. In these patients, a maximum of 30% of the training time addressed these deviances. No patients had more than two protocols.  The following ADHD protocols were used: theta/beta (7), SMR (5), and beta inhibiting (2). Additional protocols were rewarding occipital alpha (3), inhibition of 4–13 Hz occipitally (1), and inhibition of 11–13 Hz at motor strip (1). The training, which was conducted in the clinic, was completed by the first author, who has worked in the area for 30 years and with NF for 14 years; together with a licensed psychologist, receiving in-clinic training and working under close supervision | **Treatment response** (as a dichotomous outcome)  Relevant changes as improvement of ≥ 1 SD on two or more of the inattention and hyperactivity/impulsivity scales of Conners’ Parent/Teacher Rating Scales  **All-cause treatment discontinuation** (as a dichotomous outcome)  Yes, reported as study drop-outs  **Tolerability** (as a dichotomous outcome)  Not reported  **Serious adverse events** (as dichotomous outcome)  Not reported  **Specific adverse events** (as dichotomous outcome)  Not reported |
| Simonoff et al., 2013^214^ | **Eligibility criteria**  Children 7-15 years; a full-scale IQ of 30–69. Exclusion criteria were: living in a stable situation  and regular school attendance; current stimulant use; use of neuroleptic medication in the last 6 months; history of a sensitivity reaction to stimulant medication; a diagnosis of a dementing disorder; epilepsy with daily seizures; presence of a psychotic, bipolar, severe obsessive-compulsive disorder or severe Tourette syndrome; or a household resident with a current substance abuse disorder  **Diagnostic criteria**  ICD-10  **Number randomized**  147  **Age**  7-15 years (range)  **Co-morbidities/Co-medications**  Yes, all comorbid intellectual disability (100%)/Not reported  **ADHD subtypes**  Not reported | **Arm 1**  MPH-SA high dose: methylphenidate short-acting (inmediate release) up to 1.5 mg/kg/day  **Arm 2**  Placebo: placebo pill | **Treatment response** (as a dichotomous outcome)  Clinician-rated Clinical Global Impression Improvement (CGI-I) scale – e.g. responder considered very much improved or much improved  **All-cause treatment discontinuation** (as a dichotomous outcome)  Yes, reported as study discontinuations/lost to follow-up  **Tolerability** (as a dichotomous outcome)  Yes, resportes as study discontinuations due to AEs  **Serious adverse events** (as dichotomous outcome)  Not reported  **Specific adverse events** (as dichotomous outcome)  Loss of appetite/anorexia (as an adverse event e.g. poor appetite)  Sleep disturbances (as an adverse event e.g. troubling getting to sleep)  Cardiovascular events – increased blood pressure (e.g. increased SBP ≥ 5 points)  Increased pulse values (as an adverse event e.g. above the 95% centile at week 16) |
| Tamm et al., 2013^215^ | **Eligibility criteria**  Children and adolescents aged 7-15 years. Exclusion criteria were: estimated full scale IQ < 85, history of head injury, history of prenatal drug exposure, diagnosis with other congenital or acquired neurological conditions, and participating in other nonpharmacological treatment interventions for ADHD (e.g., neurofeedback, cognitivebehavioral therapy, etc.)  **Diagnostic criteria**  DSM-IV  **Number randomized**  105  **Age**  7-15 years (range)  **Co-morbidities/Co-medications**  Yes, elimination disorders (8%), anxiety (5%), oppositional disorders (3%), learning disability (1%), depression (1%) – Yes, 69%  **ADHD subtypes**  Combined (59%), inattentive (39%) | **Arm 1**  Cognitive training (attention training): the Pay Attention! materials are designed to train sustained, selective, alternating, and divided attention using visual and auditory stimuli. The visual stimuli include a set of cards depicting drawings of children and adults that can be distinguished by various features including age, gender, hair color, and other physical qualities; and a set of home layouts that include several rooms with objects that can be sorted by color, shape, and other characteristics. The auditory stimuli include lists of words played on a CD with the participant required to press a buzzer whenever a specific word is heard. The tasks become progressively more difficult (e.g., a distracting overlay is placed over the visual stimuli, a  distracting sound is played during task completion, or participants are asked to complete two tasks simultaneously). All participants completed an initial session to establish performance levels and orient them to the materials. Participants then progressed through the four modules, beginning with the simplest sustained attention tasks. After criterion was reached (e.g., gains in speed while maintaining overall accuracy), the next module was started. Not all participants completed all four modules since they progressed at different rates, although the majority completed at least the sustained and selective attention modules. Participants were given immediate feedback regarding their performance and interventionists spent time each session discussing how the targeted attentional skill could be applied in a home or school setting. Pay Attention! is designed to be flexibly delivered so that the interventionist can tailor the treatment to the specific needs of the participant. Parents were also provided with reading materials about the attention skills being trained and met with the child and interventionist for a few minutes after each session to discuss the activities practiced at each session, which skill was being trained, and how parents could support the child's implementation of the skill in home and school activities  **Arm 2**  Waiting list (WL): waitlist control | **Treatment response** (as a dichotomous outcome)  Not reported/data not abstractable  **All-cause treatment discontinuation** (as a dichotomous outcome)  Yes, reported as study lost to follow-up  **Tolerability** (as a dichotomous outcome)  Not reported  **Serious adverse events** (as dichotomous outcome)  Not reported  **Specific adverse events** (as dichotomous outcome)  Not reported |
| van Dongen-Boomsma et al., 2013^217^ | **Eligibility criteria**  Children aged 8-15; a full-scale IQ of at least 80; qEEG deviated at least 1.5 SD from normative data; did not use psychoactive drugs, or they used a stable dose of psychostimulants or ATX; there was room for improvement, defined as a minimum score of 2 on a 4-point Likert scale dor at least 6 items of the ADHD-RS-IV. Exclusion criteria were: if involved in individual or group psychotherapy; used medication other than psychostimulants or ATX; had a comorbid disorder other that oppositional defiant disorder or any anxiety; had a neurologic disorder and/or a cardiovascular disease; participated in another clinical trial at the same time; had received EEG neurofeedback in the past; or used alcohol or drugs  **Diagnostic criteria**  DSM-IV-TR  **Number randomized**  41  **Age**  8-15 years (range)  **Co-morbidities/Co-medications**  Yes, oppositional disorders (15%), anxiety (12%), dislexia (5%) – Not reported  **ADHD subtypes**  Combined (73%), inattentive (22%), hyperactive/impulsive (5%) | **Arm 1**  Neurofeedback (theta-beta training): individualized EEG neurofeedback (NF) protocols based on visual inspection of the raw EEG and qEEG were used for EEG NF training. NF training for 30 sessions given as 2 sessions per week  **Arm 2**  Placebo: placebo neurofeedback | **Treatment response** (as a dichotomous outcome)  Clinician-rated Clinical Global Impression Improvement (CGI-I) scale – e.g. responder considered very much improved or much improved  **All-cause treatment discontinuation** (as a dichotomous outcome)  Not reported  **Tolerability** (as a dichotomous outcome)  Not reported  **Serious adverse events** (as dichotomous outcome)  Not reported  **Specific adverse events** (as dichotomous outcome)  Not reported |
| Aman et al., 2014^218-220^ | **Eligibility criteria**  Children aged 6-12 years; DBD diagnosis (CD or ODD); evidence of serious physical aggression as rated on the Overt Aggression Scale–M (score ≥ 3 on assaults against other people, objects, or self); and evidence of seriously disruptive behavior as determined by a parent or guardian rating of at least 27 (90th percentile) on the NCBRF D-Total. In addition, a CGI–Severity (CGI-S) score of at least 4 (“moderately ill” or higher) for aggression was required by blinded clinicians. Participants needed to be free of psychotropic medicines for 2 weeks for most drugs (such as most antidepressants, a-agonists, b-blockers, anxiolytics, mood stabilizers, oral antipsychotics, and antihistamines) and 4  weeks for depot antipsychotics or fluoxetine. This rule was occasionally relaxed (to as few as 3–7 days) for extreme cases who could not tolerate being unmedicated the full time, as approved by the cross-site steering committee.  Exclusion criteria were: Full-Scale IQ below 71;  pregnancy or a history of seizure disorder or other neurologic or medical disorder for which medication might present a considerable risk; abnormal liver function; pervasive developmental disorder, schizophrenia, other psychotic disorders, or eating disorders; hypomanic/biphasic score of at least 36 as rated by a child’s parent on the General Behavior Inventory (see below) and, if positive, confirmed by clinician as an indication of mood disorder; current or previous major depressive disorder or diagnosis of bipolar disorder; current use of psychotropic medications from which discontinuation would present a significant risk; active substance use disorder; evidence of current child abuse or neglect; history of suicide attempt in the past year or current suicidal ideation; and family history of type 2 diabetes in at least 2 first-degree relatives (owing to potential weight gain with RISP)  **Diagnostic criteria**  DSM-IV  **Number randomized**  168  **Age**  6-12 (range)  **Co-morbidities/Co-medications**  Yes, all severe physical aggression (100%), oppositional disorders (74%), conduct disorders (26%) – Yes (all received concomitant psychoestimulants)  **ADHD subtypes**  Not reported | **Arm 1**  MPH-LA or MIX-AMPH parent training + RISP: combination of risperidone (0.5-3.5 mg/day) and methylphenidate long-acting (18-72 mg/day) or mixed amphetamine salts and behavioral therapy/parent training (COPE program)  **Arm 2**  MPH-LA or MIX-AMPH + parent training: combination of methylphenidate long-acting (18-72 mg/day) or mixed amphetamine salts and behavioral therapy/parent training (COPE program) | **Treatment response** (as a dichotomous outcome)  Clinician-rated Clinical Global Impression Improvement (CGI-I) scale – e.g. responder considered completely recovered, very much improved or much improved  **All-cause treatment discontinuation** (as a dichotomous outcome)  Yes, reported as study drop-outs  **Tolerability** (as a dichotomous outcome)  Not reported  **Serious adverse events** (as dichotomous outcome)  None  **Specific adverse events** (as dichotomous outcome)  Loss of appetite/anorexia (as an adverse event)  Sleep disturbances (as an adverse event e.g. troubling falling asleep) |
| Barragán et al., 2014^221^ | **Eligibility criteria**  Children aged 6-12 years. Exclusion criteria were: neurologic disorders (epilepsy, brain damage, mental retardation), autism or pervasive developmental disorders, known hypersensitivity to components of omega-3/6, previous pharmacological treatment for ADHD, ongoing chronic conditions (e.g., asthma), or medication for chronic conditions. Children not receiving school assistance were also excluded from the study  **Diagnostic criteria**  DSM-IV-TR  **Number randomized**  90  **Age**  6-12 years (range)  **Co-morbidities/Co-medications**  None/Not reported  **ADHD subtypes**  Combined (57%), inattentive (36%), hyperactive/impulsive (8%) | **Arm 1**  MPH-LA low/medium dose: methylphenidate long acting 0.5-1.0 mg/kg/day  **Arm 2**  PUFA: polyunsaturated fatty acid (omega-3/6 fatty acid: 558 mg EPA, 174 mg DHA, 60 mg gamma linoleic acid [GLA])  **Arm 3**  MPH-LA low/medium dose+PUFA: methylphenidate long acting 0.5-1.0 mg/kg/day + polyunsaturated fatty acid (omega-3/6 fatty acid: 558 mg EPA, 174 mg DHA, 60 mg GLA) | **Treatment response** (as a dichotomous outcome)  ADHD-RS ≥ 30% reduction from baseline in total score (rater: parents)  **All-cause treatment discontinuation** (as a dichotomous outcome)  Yes, reported as study withdrawals  **Tolerability** (as a dichotomous outcome)  Yes, reported as study withdrawals due to AEs  **Serious adverse events** (as dichotomous outcome)  Not reported  **Specific adverse events** (as dichotomous outcome)  Insomnia (as an advserse event)  Cardiovascular event – Palpitations (as an adverse event) |
| Chacko et al., 2014^222^ | **Eligibility criteria**  Children aged 7-11 years; fluency in English (parent and child) and; internet access at home. Exclusion criteria were: evidence of a pervasive developmental disorder based on previous diagnosis and/ or elevated sores on the Child Autism Rating Scale, or psychosis; the child or parent presented with emergency psychiatric needs that required immediate services (e.g., suicidal or homicidal intent) and; if the child had an estimated Full Scale IQ below 80  **Diagnostic criteria**  DSM-III-R  **Number randomized**  85  **Age**  7-11 years (range)  **Co-morbidities/Co-medications**  Yes, oppositional disorders (45%), conduct disorders (12%) – Yes, 29%  **ADHD subtypes**  Combined (62%), inattentive (38%), hyperactive/impulsive (-%) | **Arm 1**  WM training: Cognitive training – working memory training. The Cogmed WM training program is a computerized training program that targets both the  storage and storage plus processing/manipulation components of verbal and nonverbal working memory through training which takes place in approximately 30–45 minute increments over five days per week (25 training-days total). WM trials are titrated to the capacity of the individual using an adaptive staircase design that adjusts the difficulty of the program on a trial-by-trial basis. Each individual’s training is supervised by a training aide (typically a parent or guardian) and a certified WM coach, who is able to track closely (via online access) each individual’s performance and provide support to the family through weekly coaching interactions (by phone)  **Arm2**  Placebo: WM placebo | **Treatment response** (as a dichotomous outcome)  Not reported/data not abstractable  **All-cause treatment discontinuation** (as a dichotomous outcome)  Yes, reported as lost to follow-up  **Tolerability** (as a dichotomous outcome)  Not reported  **Serious adverse events** (as dichotomous outcome)  Not reported  **Specific adverse events** (as dichotomous outcome)  Not reported |
| Ferrin et al., 2014^223^ | **Eligibility criteria**  Children and adolescents aged 5-18; parents’ age greater than or equal to 18 years; responsibility and legal capacity in parents; participant on clinical ADHD symptoms stabilization for at least 1 month before entering the study, with most of their comorbidity represented (except for the exclusion criteria and including autistic spectrum disorders with mild severity), and any treatment prescribed. In those receiving medication, doses had been previously adjusted to a maximum of 1.5 mg/kg/day, according to their clinical response defined by the ADHD-RS. Exclusion criteria were: severe intellective disabilities (IQ<70); severe autistic spectrum disorders; subjects with any clinically significant or unstable medical or psychiatric condition; and children whose families had received any school-based individual and/or group psychosocial treatments at any point in time  **Diagnostic criteria**  DSM-IV  **Number randomized**  81  **Age**  5-18 years (range)  **Co-morbidities/Co-medications**  Yes, oppositional disorders and conduct disorders (30%), depression and anxiety (2%), autistic spectrum disorder (2%), other (19%)/Yes, 84%  **ADHD subtypes**  Combined (75%) | **Arm 1**  BT (parent training): The BT/psychoeducation programme was developed according to the basic principles and requirements for an educational programme; it was adapted and implemented from a previous evidence-based programme developed for patients with Bipolar Disorder.The BT group was composed of five successive groups of 8–10 families who received 12-week 90 min weekly sessions; families were educated on the disorder during the first nine sessions and finally very briefly introduced to a range of behavioural strategies for managing ADHD symptoms and reducing defiant behaviour during the last three. The integrity of the  BT sessions was guaranteed by a manual that explicitly outlined all the procedures to be used in the  intervention. Sessions were audiotaped and an independent person reviewed through a checklist that the different groups received an equivalent set of information. Parents received no further parental training or behavioural strategies as the aim of the programme was purely educational; they were given the opportunity to express their own experiences and feelings about their child and the impact that the child’s condition had had on them. At the end of each session a hand-out was delivered  **Arm 2**  Parent counseling with support (control): The parent-support group consisted of another five successive groups of 8–10 families who received 12-week 90 min weekly sessions; these families were reunited and encouraged to comment on their thoughts and share their experiences in a nondirective, nonthreatening environment. In this case, the therapist was not allowed to provide formal psychotherapy or specific psychoeducation and families did not receive any specific educational material. The therapist was not allowed to give any feedback or additional information, but to guide the groups and allow everyone to express and to give their personal point of view | **Treatment response** (as a dichotomous outcome)  Not reported/data not abstractable  **All-cause treatment discontinuation** (as a dichotomous outcome)  Yes, reported as lost to follow-up  **Tolerability** (as a dichotomous outcome)  Not reported  **Serious adverse events** (as dichotomous outcome)  Not reported  **Specific adverse events** (as dichotomous outcome)  Not reported |
| Garg et al., 2014^224^ | **Eligibility criteria**  Children aged 6–14 years; moderate to severe illness as assessed by Clinical Global Impressions Severity Scale (CGI-S). Exclusion criteria were: patients with history of non-response or adverse drug reactions to MPH or ATX in the past, those who had taken any medication for ADHD in past one month, or those with history of heart disease, seizures, pervasive developmental disorder, substance abuse, mental retardation or tic disorder  **Diagnostic criteria**  DSM-IV-TR  **Number randomized**  84  **Age**  6-14 years (range)  **Co-morbidities/Co-medications**  Yes, oppositional disorders (54%), conduct disorders (10%)/Not reported  **ADHD subtypes**  Combined (70%); inattentive (22%), hyperactive/impulsive (9%) | **Arm 1**  MPH-SA low/medium dose: methylphenidate 0.2-1 mg/kg/dat  **Arm 2**  ATX: atomoxetine 0.5-1.2 mg/kg/dat | **Treatment response** (as a dichotomous outcome)  Responders based on the achievement of a > 25% or decrease from baseline to endpoint in the Vanderbilt ADHD-RS total score (rater: parents)  **All-cause treatment discontinuation** (as a dichotomous outcome)  Yes, reported as study drop-outs  **Tolerability** (as a dichotomous outcome)  Yes, reported as study drop-outs due to AES  **Serious adverse events** (as dichotomous outcome)  Not reported  **Specific adverse events** (as dichotomous outcome)  Loss of appetite/anorexia (as an adverse event)  Insomnia (as an adverse event) |
| Hervas et al., 2014^225-227^ | **Eligibility criteria**  Children aged 6-17 years; baseline ADHD-RS-IV with a total score of 32 or higher and a minimum Clinical Global Impression-Severity (CGI-S) score of 4, were enrolled in the study. Those with age appropriate intellectual functioning; blood pressure measurements within the 95th percentile for age, sex and height; and the ability to swallow tablets or capsules were included. Girls of childbearing potential had to have a negative urine pregnancy test at screening and baseline and to comply with any protocol contraceptive requirements. Exclusion criteria were: clinically significant illness, including ac linically significant abnormal screening visit; current, comorbid psychiatric diagnosis (except oppositional defiant disorder; history/presence of cardiac abnormalities, cardiovascular or cerebrovascular disease, serious heart rhythm abnormalities, syncope, tachycardia, cardiac conduction problems, exercise-related cardiac events or clinically significant bradycardia; orthostatic hypotension and/or aknown history of hypertension; seizures; and glaucoma; those with a family history of sudden cardiac death, ventricular arrhythmia or QT prolongation, a patient history of alcohol or substance abuse and those patients with serious tic disorder, including Tourette's syndrome  **Diagnostic criteria**  DSM-IV-TR  **Number randomized**  338  **Age**  6-17 years (range)  **Co-morbidities/Co-medications**  Yes, oppositional disorders (12%)/Not reported  **ADHD subtypes**  Combined (85%), inattentive (11%), hyperactive/impulsive (4%) | **Arm 1**  ATX: atomoxetine 0.5-1.4 mg/kg/day  **Arm 2**  GUAN-LA: guanfacine long acting 1-7 mg/day  **Arm 3**  Placebo: placebo pill | **Treatment response** (as a dichotomous outcome)  Global improvement based on Clinical Global Impression Improvement (CGI-I) scale. Subjects with improvement of “very much” or “much” improved were considered responders e.g. GGI-I ≤ 2 (rating evaluations reported by clinician; Global improvement based on Clinical Global Impression Severy (CGI-S) scale e.g. CGI-S ≤ 3; Achievement of a 30% or greater decrease from baseline to endpoint in the ADHD-RS total score and a CGI-I of 1 or 2 (rater: clinician)  **All-cause treatment discontinuation** (as a dichotomous outcome)  Yes, reported as study discontinuation  **Tolerability** (as a dichotomous outcome)  Yes, reported as study discontinuation due tu AEs  **Serious adverse events** (as dichotomous outcome)  Yes, reported  **Specific adverse events** (as dichotomous outcome)  Loss of appetite/anorexia (as an adverse event)  Decreased weight (significant weight loss e.g. ≥ 7% of body weight loss)  Insomnia (as an adverse event)  Anxiety (as adverse event)  Syncope (as an adverse event)  Cardiovascular event –  blood pressure decrease (e.g. SBP decrease ≥ 25 mmHG, postural orthostatic blood pressure)  Tachycardia (as an adverse event) |
| Hirayama et al., 2014^228^ | **Eligibility criteria**  Children aged 4-14 years. Exclusion criteria not reported  **Diagnostic criteria**  DSM-IV-TR  **Number randomized**  40  **Age**  4-14 years (range)  **Co-morbidities/Co-medications**  Not reported/Not receiving stimulants  **ADHD subtypes**  Not reported | **Arm 1**  P-SER: phosphatidyl-serine 200 mg/day  **Arm 2**  Placebo: placebo pill | **Treatment response** (as a dichotomous outcome)  Not reported/data not abstractable  **All-cause treatment discontinuation** (as a dichotomous outcome)  Yes, reported as study drop-outs  **Tolerability** (as a dichotomous outcome)  Not repoted  **Serious adverse events** (as dichotomous outcome)  Not reported  **Specific adverse events** (as dichotomous outcome)  Not reported |
| Ko et al., 2014^229^ | **Eligibility criteria**  Children and adolescents aged 6-15; had symptoms for > 6 months and had morning salivary cortisol levels < 10 ng/mL, which is considered to indicate chronic stress. Exclusion criteria were: subjects were excluded if they were taking drugs that could affect psychological symptoms, including antipsychotic drugs; common cold drugs such as amphetamine, ephedrine, and ginseng products; or vitamin supplements. Subjects were also excluded if they had adrenal or thyroid disease, or if they had received a psychological diagnosis within the preceding 3 months  **Diagnostic criteria**  DSM-IV  **Number randomized**  72  **Age**  6-15 years (range)  **Co-morbidities/Co-medications**  No/Not reported  **ADHD subtypes**  Not reported | **Arm 1**  Herbal therapy: *Ginseng* (Korean red ginseng) 2 mg/day  **Arm 2**  Placebo | **Treatment response** (as a dichotomous outcome)  Not reported/data not abstractable  **All-cause treatment discontinuation** (as a dichotomous outcome)  Yes, reported as study drop-outs  **Tolerability** (as a dichotomous outcome)  Not reported  **Serious adverse events** (as dichotomous outcome)  Not reported  **Specific adverse events** (as dichotomous outcome)  Not reported |
| Lin et al., 2014^230,231^ | **Eligibility criteria**  Children and adolescents aged 6-17; ADHD-RS-IV-Parent:Inv total score ≥ 1.5 SD above the age and gender norms (at screening and week 0), and have a CGI-ADHD-S score ≥ 4 (at screening and  week 0). Exclusion criteria were: body weight < 18 kg or > 75 kg; history of bipolar I or II disorder, or psychosis; any seizure disorder or pervasive developmental disorder; presence of motor tics or a diagnosis of Tourette’s syndrome; marked anxiety, tension, or agitation sufficient to contraindicate treatment with OROS MPH; history of electroencephalographic abnormalities; clinically significant abnormal electrocardiogram; serious or unstable medical illness; any medical condition that would increase sympathetic nervous systemactivity markedly (e.g., catecholaminesecreting neural tumor); requiring the daily use of medications with sympathomimetic activity (e.g., albuterol, pseudoephedrine); any medical condition that would be exacerbated by an increase in norepinephrine tone; or current or past history of clinically significant hypertension  **Diagnostic criteria**  DSM-IV-TR  **Number randomized**  340  **Age**  6-17 years (range)  **Co-morbidities/Co-medications**  Yes, conduct disorders (19%), anxiety (2%), oppositional disorders (2%) – Not reported  **ADHD subtypes**  Combined (71%), inattentive (25%), hyperactive/impulsive (4%) | **Arm 1**  MPH-LA high dose: methylphenidate long acting (36-54 mg/day)  **Arm 2**  EDIVOX: edivoxetine 0.1-0.3 mg/kg/day  **Arm 3**  Placebo: placebo pill | **Treatment response** (as a dichotomous outcome)  Achievement of a 40% or greater decrease from baseline to endpoint in the ADHD-RS total score (rater: parents); Clinician-rated Clinical Global Impression Improvement (CGI-I) scale – e.g. responder considered very much improved or much improved (CGI-I ≤ 2)  **All-cause treatment discontinuation** (as a dichotomous outcome)  Yes, reported as study discontinuations  **Tolerability** (as a dichotomous outcome)  Yes, reported as study discontinuations due to AEs  **Serious adverse events** (as dichotomous outcome)  Not reported  **Specific adverse events** (as dichotomous outcome)  Loss of appetite/anorexia (as an adverse event)  Decreased weight (as an adverse event)  Insomnia (as an adverse event)  Sleep disturbances (as an adverse event)  Cardiovascular events – increased heart rated (as an adverse event) |
| Meisel et al., 2014^232^ | **Eligibility criteria**  Children and adolescents aged 7-14 years; IQ>80; scoring over 90th percentile in the ADHD-RS teache version and over 80th percentile in parent version; scoring over 7 points (from 24) in ODD scale; scoring under 5 (1–10 scale) in any academic area. Children were not receiving medication, at  least for two weeks before starting treatment, or concurring psychotherapy. Exclusion criteria were: children with comorbid disorders (other than  oppositional defiant disorder) evaluated through the Child Behavior Checklist (CBLC)  **Diagnostic criteria**  DSM-IV  **Number randomized**  130  **Age**  7-14 years (range)  **Co-morbidities/Co-medications**  None – No  **ADHD subtypes**  Combined (78%), inattentive (22%) | **Arm 1**  MPH-SA low/medium dose: methylphenidate < 1 mg/kg/day  **Arm 2**  Neurofeedback (theta-beta training): NF was conducted using Atlantis II 2x2 equipment from Brainmaster. This equipment uses an impedance check (below 5 k_) and controls artifacts automatically (>120 _V). The EEG was analyzed in two frequency bands (theta: 4–7 Hz, beta: 15–20 Hz). EEG recordings were obtained from a monopolar electrode site situated  on Cz for participants between 7 and 11 years old. For older participants it was calculated at FCz, based in the International 10/20 System, with ear references.  The 40 theta/beta training sessions were conducted by trained last-year psychology undergraduates supervised by the first author. Participants had two sessions per week. Each session consisted of six runs of 4 min each. Baseline values were determined at the beginning of each session (30 s). Participants had short pauses between runs that enabled them to relax. In sum, a session had a length of 35 min approximately.  The training was presented to the child as a computer game (puzzles, races, Pac-man, etc.) in which he/she had to concentrate to win. Specifically, children were instructed by the trainer to develop and prolong the strategy that best helped them to win points in the game. The child received visual and auditory reinforcement contingent on his/her success in controlling microvolts of theta and/or beta. The program calculated individual thresholds according to daily baseline values, and had the following reinforcement plan: 70% of the time below the threshold in theta, and up to 20% of the time below the threshold in beta (or 80% above the threshold) was rewarded. Throughout the session these reward thresholds were manually adjusted by the trainer when it was too difficult or too easy for the child to meet the criteria. Trainers verbally reinforced participants when concentration states were prolonged. Additionally, performance graphs were shown to participants during pauses. This enhanced motivation and engagement to the task. Another motivational factor proved to work very well for almost all participants: daily scores were written on a chalkboard. Participants were eager to compete with other members of the group and worked harder for getting the top scores. Independent of these scores, every week candies, picture cards or sketches of their favorite character/subject were given to all participants | **Treatment response** (as a dichotomous outcome)  Not reported/data not abstractable  **All-cause treatment discontinuation** (as a dichotomous outcome)  Yes, reported as study discontinuations  **Tolerability** (as a dichotomous outcome)  Yes, reported as study discontinuations due to AEs  **Serious adverse events** (as dichotomous outcome)  Not reported  **Specific adverse events** (as dichotomous outcome)  Not reported |
| Pfiffner et al., 2014^233^ | **Eligibility criteria**  Children aged 7-11 years; Inattentive subtype. Exclusion criteria not reported  **Diagnostic criteria**  DSM-IV  **Number randomized**  199  **Age**  7-11 years (range)  **Co-morbidities/Co-medications**  Not reported – Yes, 4%  **ADHD subtypes**  Inattentive (100%) | **Arm 1**  BT (child, parent and teacher training): Behavioral therapy as per CLAS program. CLAS included three manualized coordinated components: a) ten 90-minute parent group meetings, along with up to six 30-minute family meetings (parent, child, and therapist); b) ten 90-minute child group meetings (child training); and c) teacher consultation, which included one 30-minute orientation meeting involving the teacher and therapist and up to five subsequent 30-minute meetings with the parent, child, teacher, and therapist and booster sessions. Parent and child groups contained between five and eight families and were held in clinic offices. Individual meetings with families occurred in clinic offices, on the telephone, or in a private location on school grounds. Teacher consultation occurred on school grounds, or occasionally over the telephone  **Arm 2**  BT (parent training): BT parent training group included only the parent training group component described above which was adapted from existing parent training programs  **Arm 3**  Standard care (control): usual care | **Treatment response** (as a dichotomous outcome)  Clinically meaningful response rates as an improvement of ADHD symptoms using Child Symptom Inventory (CSI) teachers/parents rating with a mean of CSI symptom severity score within 1 SD of the normative mean  **All-cause treatment discontinuation** (as a dichotomous outcome)  Yes, reported as drop-outs/terminated early  **Tolerability** (as a dichotomous outcome)  Not reported  **Serious adverse events** (as dichotomous outcome)  Not reported  **Specific adverse events** (as dichotomous outcome)  Not reported |
| Steiner et al., 2014^234,235^ | **Eligibility criteria**  Children aged 7-11 years; child in second or fourth grade, and ability to speak and understand English sufficiently to follow the intervention protocol, although English need not be their first language. In order to increase external validity of running a school-based intervention, children were included regardless of medication status. Exclusion criteria were: children with a coexisting diagnosis of conduct disorder, autism spectrum disorder, or other serious mental illness (e.g., psychosis) or with an intelligence quotient <80 measured by the Kaufman Brief Intelligence Test  **Diagnostic criteria**  DSM-IV  **Number randomized**  104  **Age**  7-11 years (range)  **Co-morbidities/Co-medications**  No – Yes, 49% comedications  **ADHD subtypes**  Not reported | **Arm 1**  Cognitive training (attention training): the cognitive training intervention system used includes an array of  cognitive exercises. Authors used those that target areas of attention and working memory. For example, in 1 exercise, as participants match letter-number pairs correctly, a safe becomes unlocked, and children win a virtual prize. The tasks become more challenging as the participant progresses. Automatic progress from one exercise to the next makes it possible to deliver the intervention on a larger scale. The exercises are both auditory and visual, and users are able to design their own custom exercise protocols  **Arm 2**  Neurofeedback (theta-beta training): The NF intervention system used trains the child to increase beta waves and suppress theta waves. This system  uses EEG sensors that are embedded in a typical looking bicycle helmet, without requiring conductive gel, significantly easing delivery to children on a large scale. When the theta-to-beta ratio decreases, reflecting effective focusing, the participant progresses on the exercise. For example, in 1 specific exercise, as the theta-to-beta ratio decreases, a dolphin character swims down to the bottom of the ocean to collect coins from a treasure chest, and the child earns points. If the child becomes distracted, the dolphin swims back up to the surface of the ocean  **Arm 3**  Control: standard computer format | **Treatment response** (as a dichotomous outcome)  Not reported/data not abstractable  **All-cause treatment discontinuation** (as a dichotomous outcome)  Yes, reported as study discontinuations  **Tolerability** (as a dichotomous outcome)  Not reported  **Serious adverse events** (as dichotomous outcome)  Not reported  **Specific adverse events** (as dichotomous outcome)  Not reported |
| van Dongen-Boomsma et al., 2014^236^ | **Eligibility criteria**  Children aged 5-7 years; IQ of at least 80; medication-free; access to a computer with  Windows Vista/XP/7 and speakers, and access to internet. Exclusion criteria were: current intensive (i.e., weekly) psychotherapy; another comorbid psychiatric diagnosis except for Pervasive Developmental Disorder Not Otherwise Specified or Oppositional Defiant Disorder; any neurological and/or cardiovascular disease, currently or in the past; serious motor or perceptual handicap;  participation in another clinical trial  **Diagnostic criteria**  DSM-IV-TR  **Number randomized**  51  **Age**  5-7 years (range)  **Co-morbidities/Co-medications**  Yes, with comorbidities (19%), oppositional disorders (9%)– Yes, 29%  **ADHD subtypes**  Combined (70%), inattentive (9%), hyperactive/impulsive (21%) | **Arm 1**  WM training: Cognitive training – working memory training. The Cogmed training consisted of 25 sessions of 15 min, 5 days a week, included 7 visuospatial WM tasks. For all tasks, a number of visual stimuli were presented sequentially on the computer screen and the child had to remember both their location and order to subsequently respond by mouse clicking the targets in correct order. In the active condition, the software adjusted task difficulty based on the child’s performance. The parents were instructed to encourage the child during the training course, and give small rewards every five sessions and after training completion. A certified Cogmed coach contacted the parents every week to evaluate the performance and motivation of the child with a standardized questionnaire  **Arm2**  Placebo: WM placebo | **Treatment response** (as a dichotomous outcome)  Clinician-rated Clinical Global Impression Improvement (CGI-I) scale – e.g. responder considered very much improved or much improved (CGI-I ≤ 2)  **All-cause treatment discontinuation** (as a dichotomous outcome)  Yes, reported as study discontinuations  **Tolerability** (as a dichotomous outcome)  Not reported  **Serious adverse events** (as dichotomous outcome)  Not reported  **Specific adverse events** (as dichotomous outcome)  Not reported |
| Widenhorn-Müller et al., 2014^237^ | **Eligibility criteria**  Children aged 6-12; at least six out of nine symptoms of inattention (score 2 or 3) and/or six out of eleven symptoms of hyperactivity/impulsivity (score 2 or 3); onset of problems had to occur before age 7 and persisted for at least 6 months prior to diagnosis. Exclusion criteria were: IQ ≤ 70, use of stimulant medication and other psychoactive medication as wells as fatty acid supplements used within the previous 6 months; allergies against fish or fish products  **Diagnostic criteria**  DSM-IV  **Number randomized**  110  **Age**  6-12 years (range)  **Co-morbidities/Co-medications**  Not reported/No  **ADHD subtypes**  Combined (43%), inattentive (55%), hyperactive/impulsive (2%) | **Arm 1**  PUFA: polyunsaturated fatty acid (omega-3 fatty acid: eicosapentaenoic acid and docosahexaenoic acid)  **Arm 2**  Placebo: olive oil | **Treatment response** (as a dichotomous outcome)  Not reported/data not abstractable  **All-cause treatment discontinuation** (as a dichotomous outcome)  Yes, reported as study discontinuations/lost to follow-up  **Tolerability** (as a dichotomous outcome)  Not reported  **Serious adverse events** (as dichotomous outcome)  Not reported  **Specific adverse events** (as dichotomous outcome)  Not reported |
| Abikoff et al., 2015^238^ | **Eligibility criteria**  Children aged 3-4 years; the primary caretaker be fluent in English and that the child have  an IQ ≥ 70 on the Wechsler Preschool Primary Scale of Intelligence; elevated scores above age and gender norms on the DSM-IV Total, DSMIV  Hyperactive/Impulsive, or DSM-IV Inattentive subscales on both the Revised Conners Teacher (CTRS-R) (T-score ≥ 65) and Parent (CPRS-R) Rating Scales (T-score ≥ 60); standard score ≥ 7 on the  Concepts and Following Directions subscale of the Clinical Evaluation of Language Fundamentals (CELF-2). Exclusion criteria were: current medication or behavioral treatment for ADHD; a diagnosis of pervasive developmental disorder, psychosis, or post-traumatic stress disorder; history of sexual or physical abuse; or any other psychiatric or medical condition judged to contraindicate participation. Children with common mental health diagnoses were not excluded  **Diagnostic criteria**  DSM-IV  **Number randomized**  164  **Age**  3-4 years (range)  **Co-morbidities/Co-medications**  Yes, oppositional disorders (42%), anxiety (7%) – Not reported  **ADHD subtypes**  Combined (51%), inattentive (15%), hyperactive/impulsive (34%) | **Arm 1**  BT (parent training): New forest parenting package (NFPP) or Helping the noncompliant child (HNC). NFPP, a manualized intervention for preschoolers with ADHD, involves 8 weekly 1-to-1.5-hour sessions, delivered in the family home by trained clinicians.  NFPP focuses on key issues related to ADHD children’s functioning, and relies on the parent as the primary agent of change. While it shares a number of features with standard BT (i.e., management of problematic behavior using behavioral techniques; promotion of authoritative parenting; increasing the quality and quantity of positive and reciprocal parent–child interaction; reduction of parental negative reactivity; and between-session ‘homework tasks’ to facilitate  improvement in specific parenting techniques), it has a  number of distinctive features. First, its home-based nature enables the therapist to model play and behavioral strategies for the parent in the setting where the behaviors are problematic. It also enables the therapist to address naturally occurring instances of problematic child behaviors that call for the use of the parenting (and child) skills being taught. Sensitizing parents to the importance of these ‘teachable moments’ and of identifying and exposing their child to relevant real-world situations where skills can be used provides numerous opportunities for skills development and generalization. Second, NFPP directly aims to improve four elements of constructive parenting: (a) Scoping– learning how to observe their child’s current level of competencies so as to promote realistic expectations and performance goals for their child regarding self-control, attention, and memory, (b) Extending – establishing new goals based on their child’s performance and progress, (c) Scaffolding– using game-like activities to facilitate their child’s skills development and goal achievement, and (d) Consolidation—promoting their child’s skill use across settings and situations to facilitate generalization. Third, NFPP educates parents to alter their views of ADHD, avoid blaming their child for ADHD symptoms, and increase parental tolerance with the ultimate goal of improving the quality of the parent–child relationship.  HNC is a manualized BT intervention for treating  young children with noncompliance and oppositional problems. The individualized, clinic-based, treatment is delivered by therapists, with the parent and child jointly, in each session. The clinical provision of HNC typically averages 8–10 intervention sessions. To ensure that NFPP and HNC were equated for length and amount of therapist contact, HNC was delivered in 8 weekly sessions, lasting approximately one hour. HNC was provided according to the details specified in the McMahon and Forehand (2003) treatment manual, except that a fixed number of sessions was conducted and meeting behavioral criteria for advancement from one parenting skill to the next was not required.  HNC is based on social-learning theory and behavior modification principles and methods and incorporates characteristics of the BT model developed by Hanf (1969). Treatment focuses on reducing noncompliance using a variety of methods to teach parents how to change their maladaptive interaction patterns with their child. Specific program components include: (a) modeling and parent role play, along with didactic  instruction and discussion, to teach parents the skills of attending, rewarding, ignoring, clear instructions and time out, and (b) home practice, assignments and exercises, throughout the program.  **Arm 2**  Waiting list (WL): waitlist control | **Treatment response** (as a dichotomous outcome)  Not reported/data not abstractable  **All-cause treatment discontinuation** (as a dichotomous outcome)  Yes, reported as study drop-outs  **Tolerability** (as a dichotomous outcome)  Not reported  **Serious adverse events** (as dichotomous outcome)  Not reported  **Specific adverse events** (as dichotomous outcome)  Not reported |
| Bigorra et al., 2015^239^ | **Eligibility criteria**  Children aged 7-12 years; combined-type ADHD; *T* scores on the Conners ADHD index for parents and teachers >70 at the time of diagnosis; no previous psychological or pharmacological treatment  for ADHD; and access to a personal computer with Internet connection. Exclusion criteria were: IQ < 80; comorbidity with autism spectrum disorder, psychosis, affective or anxiety disorder, consumption of toxic substances, or learning disorder; history of traumatic brain injury in the last 2 years; and perceptual-motor alterations that would preclude the use of a computer. Participants whose educational or socio-economic context would make it unlikely for families to comply with the study requirements and follow the treatment procedure (subjects whose families did not speak Spanish or were monitored by social services due to suspected abuse/neglect) were also excluded from the study. Furthermore, children who participated in fewer than 20 training sessions were excluded from the posterior data analysis, as were those who initiated other pharmacological  or psychological treatments during study participation  **Diagnostic criteria**  DSM-IV-TR  **Number randomized**  66  **Age**  7-12 years (range)  **Co-morbidities/Co-medications**  Yes, oppositional disorders (27%), elimination disorder (5%)– Not reported  **ADHD subtypes**  Combined (100%) | **Arm 1**  WM training: Cognitive training – working memory training. The experimental group underwent WM training RoboMemo® (2005, Cogmed Cognitive Medical Systems AB, Stockholm, Sweden), which consisted of visuospatial, auditory, and location memory and tracking of moving visual objects  as WM tasks. Each training session included 90 trials and had a duration of 30–45 min. Participants attended 5 sessions per week over a 5-week period for a total of 25 sessions. The level of difficulty was automatically adjusted to the performance of each participant, thus generating a prolonged cognitive demand that exceeded existing capacity limits to keep the task challenging throughout the training phase and thereby maximize WM performance gains. This is based on the fact that cognitive plasticity is driven by a prolonged mismatch between functional organismic supplies and environmental demands  **Arm2**  Placebo: WM placebo | **Treatment response** (as a dichotomous outcome)  Not reported/data not abstractable  **All-cause treatment discontinuation** (as a dichotomous outcome)  Yes, reported as study drop-outs (including exclusions from analyses)  **Tolerability** (as a dichotomous outcome)  Not reported  **Serious adverse events** (as dichotomous outcome)  Not reported  **Specific adverse events** (as dichotomous outcome)  Not reported |
| Bédard et al., 2015^240^ | **Eligibility criteria**  Children aged 8-15; medically and psychiatrically healthy; rated ≥ 4 on the Clinical Global Impression-Severity (CGI-S). Exclusion criteria not reported  **Diagnostic criteria**  DSM-IV  **Number randomized**  25  **Age**  8-15 years (range)  **Co-morbidities/Co-medications**  Yes, oppositional disorders (44%)/Not reported  **ADHD subtypes**  Combined (68%), inattentive (32%) | **Arm 1**  GUAN-LA: guanfacine long acting 1-4 mg/day (mean dose: 2.8 mg/day)  **Arm 2**  Placebo: placebo pill | **Treatment response** (as a dichotomous outcome)  Global improvement based on Clinical Global Impression Improvement (CGI-I) scale. Subjects with improvement of “very much” or “much” improved were considered responders e.g. GGI-I ≤ 2 (rating evaluations reported by clinician  **All-cause treatment discontinuation** (as a dichotomous outcome)  Not reported  **Tolerability** (as a dichotomous outcome)  Not reported  **Serious adverse events** (as dichotomous outcome)  Not reported  **Specific adverse events** (as dichotomous outcome)  Not reported |
| Bos et al., 2015^241^ | **Eligibility criteria**  Children (only boys) aged 8-14 years; either medication naïve or using psychostimulant medication (MPH only). Children with ADHD who were on stimulant medication were instructed not to take their medication for 24 h before the fMRI scan. However, children were allowed to use their medication throughout the intervention period (16 weeks). Medication continued to be managed by the outside provider (eg, general practitioner, pediatrician, or psychiatrist). Any changes in medication status were recorded on a monthly basis by the research team. Exclusion criteria not reported  **Diagnostic criteria**  DSM-IV  **Number randomized**  40  **Age**  8-14 years (range)  **Co-morbidities/Co-medications**  Not reported/No  **ADHD subtypes**  Not reported | **Arm 1**  PUFA: polyunsaturated fatty acid (omega-3 fatty acid: eicosapentaenoic acid 650 mg and docosahexaenoic acid 650 mg per 10 g serving)  **Arm 2**  Placebo: plant oil | **Treatment response** (as a dichotomous outcome)  Not reported/data not abstractable  **All-cause treatment discontinuation** (as a dichotomous outcome)  Yes, reported as study discontinuations/lost to follow-up  **Tolerability** (as a dichotomous outcome)  Yes, reported as study discontinuations/lost to follow-up due to AEs  **Serious adverse events** (as dichotomous outcome)  Not reported  **Specific adverse events** (as dichotomous outcome)  Not reported |
| Choi et al., 2015^242^ | **Eligibility criteria**  Adolescents (boys) aged 13-18 years; drug naive or drug free during the previous 6 months, and 3) intelligence quotient (IQ) ≥ 80. Exclusion criteria were: other axis I disorders, including depression and tic disorder; history of head trauma with loss of consciousness, seizure disorder, multiple sclerosis, brain tumor, claustrophobia, metal implants, or cerebrovascular accident; IQ <  80; a history of substance abuse  **Diagnostic criteria**  DSM-IV  **Number randomized**  35  **Age**  13-18 years  **Co-morbidities/Co-medications**  None – Not reported  **ADHD subtypes**  Not reported | **Arm 1**  MPH-SA low/medium dose+BT (child training): methylphenidate (10-40 mg/day) in combination with behavioral therapy (education for behavior control). The BT intervention consisted of 12 sessions (S); S1: a  time for self-introduction, S2: a comparison of good behavior and bad behavior, S3: a review of self behavior, S4: a comparison of self and others, S5: doing well with family, S6: making friends I, S7: making friends II, S8: treatment methods for attention defi cit, S9: treatment methods for hyperactivity, S10: anger control, S11: guest speakers who have recovered from ADHD. S12: summary of ADHD education  **Arm 2**  MPH-SA low/medium dose+physical activity: methylphenidate (10-40 mg/day) in combination with physical activity or sports therapy. The research team for sports therapy consisted of 1 psychiatrist, 2 sports psychologist, and 4 teaching assistants majoring in sports psychology. Three times per week, 90-min sessions of sports therapy were scheduled as follows: 10 min for stretching and warming up, 60 min for aerobic exercise, and 10 min for feedback and cooling down. Aerobic exercises consisted of running (shuttle run, zigzag run), jumping rope (individual and group jumps), and basketball (dribble, pass, shoot, and game) to achieve a target HR (THR) of 60% HRmax intensity. Assessments of HR before and during exercise have been recommended as a method for establishing exercise intensity. HRrest and HRmax were measured once at the beginning of the study. HRmax was calculated using the ‘‘220 j age’’ formula. THR was calculated by the Karvonen formula, as follows: THR =  (HRmax j HRrest) x % intensity desired + HRrest. We  used 60% as the ‘‘intensity desired’’ in this formula on the basis of previous studies conducted in Thailand on preadolescents (9.5 ± 0.5 yr old) and youths (18–25 yr old). During the sports therapy period, if adolescents reported excessive exertion, they were allowed to rest until their HR went to down to HRrest level. The mean rest time was 7.1 T 2.7 min. | **Treatment response** (as a dichotomous outcome)  Not reported/data not abstractable  **All-cause treatment discontinuation** (as a dichotomous outcome)  Yes, reported as study discontinuations/exclusions  **Tolerability** (as a dichotomous outcome)  Yes, reported as study discontinuations due to AEs  **Serious adverse events** (as dichotomous outcome)  Not reported  **Specific adverse events** (as dichotomous outcome)  Not reported |
| Choi et al., 2015^243^ | **Eligibility criteria**  Children aged 9-13 years; IQ score above 90; Behavior Problem Scale score on the Child Behavior Checklist (CBCL) within the clinical range. All participants were under medication at the time of intervention. Exclusion criteria not reported  **Diagnostic criteria**  DSM-IV  **Number randomized**  80  **Age**  9-13 years  **Co-morbidities/Co-medications**  None – Yes (all, unspecified)  **ADHD subtypes**  Not reported | **Arm 1**  BT (child training): Emotion management training (EMT) or Social skills training (SST) program.  The EMT is an emotion identification and expression treatment, and consists of four major components: (a) identification and labeling of emotional words, (b) emotional recognition and expression, (c) emotional understanding, and (d) emotional regulation in social situations. Each session began by discussing any problems or issues related to homework from the previous one, followed by exercises, and ending with  an evaluation of the session. The SST program is  based on the studies conducted by Elliott and Gresham  (1991) and Pfiffner and McBurnett (1997). The SST is a  form of BT training focused on teaching various social skills to children with ADHD to improve their interaction with peers and teachers. The SST uses various behavioral techniques such as prompts, role-play, and reinforcement. Each session was focused on teaching a particular social skill such as listening skills, conversation skills, joining in, and reacting to rejection, negotiating, and reacting to being teased and criticized. Each session started with discussing homework, followed by exercises, and ending with a new homework assignment and an evaluation of the session  **Arm 2**  Waiting list (WL): waitlist control | **Treatment response** (as a dichotomous outcome)  Not reported/data not abstractable  **All-cause treatment discontinuation** (as a dichotomous outcome)  Yes, reported as study discontinuations/exclusions  **Tolerability** (as a dichotomous outcome)  Not reported  **Serious adverse events** (as dichotomous outcome)  Not reported  **Specific adverse events** (as dichotomous outcome)  Not reported |
| Chou et al., 2015^244^ | **Eligibility criteria**  Children and adolescents aged 7-17 years; drug-naïve. Exclusion criteria were: a full-scale IQ of less than 75 and a clinical diagnosis of systemic disease, neurological disorders or psychiatric disorders (except ADHD in the ADHD group)  **Diagnostic criteria**  DSM-IV  **Number randomized**  50  **Age**  7-17 years  **Co-morbidities/Co-medications**  Yes, oppostional disorders (33%) – Not reported  **ADHD subtypes**  Not reported | **Arm 1**  MPH-LA low/medium dose: methylphenidate long acting 28.8 mg/day (0.86 mg/kg/day)  **Arm 2**  ATX: atomoxetine 31.8 mg/day (0.75 mg/kg/day) | **Treatment response** (as a dichotomous outcome)  Not reported/data not abstractable  **All-cause treatment discontinuation** (as a dichotomous outcome)  Yes, reported as study discontinuations/exclusions  **Tolerability** (as a dichotomous outcome)  Not reported  **Serious adverse events** (as dichotomous outcome)  Not reported  **Specific adverse events** (as dichotomous outcome)  Not reported |
| Corkum et al., 2015^245^ | **Eligibility criteria**  Children aged 9-13 years; attending Grades 1 to 6 in a participating public school board; enrolled in an English classroom, or if French Immersion the teacher was able to complete the program in English; previously diagnosed with ADHD by a health care provider who was certified to make mental health diagnoses (i.e., physician, psychologist); on a stable dose of medication for ADHD or was taking no medication, with no plan to start or change medications for the duration of the study. Exclusion criteria were: child could  not (a) currently have an Individualized Program Plan (IPP) due to significant physical, behavioral, communication, or intellectual difficulties; (b) have significant co-occurring mental health problems aside from ADHD (e.g., no depression, anxiety, or severe conduct problems); (c) have a moderate or severe intellectual impairment; and that the teacher could not (d) have had previous involvement with the *Teacher Help for ADHD* program (e.g., provided feedback during the development stage, participated in the pilot study)  **Diagnostic criteria**  DSM-oriented (diagnosed with ADHD by a health care provider who was certified to make mental health diagnoses (i.e., physician, psychologist)  **Number randomized**  80  **Age**  6-12 years  **Co-morbidities/Co-medications**  None – Not reported  **ADHD subtypes**  Not reported | **Arm 1**  BT (teacher training): The intervention was accessed through a learning management system hosted on a secure server at Dalhousie University (i.e., Online Web Learning [OWL]/Blackboard [BBLearn]). Teachers reviewed the intervention content which included PowerPoint presentations and supporting documents, online through the OWL/BBLearn learning management system. Teachers were given access to one new session each Monday for 6 weeks along with a Discussion Board reminder to encourage them to access and work through the session. Introductory videos for each session featured coinvestigators describing the content of the session and encouraging active participation. Each week’s session ended with a brief questionnaire based on that week’s session, and  participants were contacted if weekly questionnaires were not completed within a few days of the end of the session. If the teachers encountered problems when implementing intervention strategies with their students, the ADHD coach was available on the Discussion Board and privately through the internal email system to answer questions and clarify information. The 6-week program included the following topics: • Session 1 targeted common myths about ADHD and provided information about impact, etiology, and effective treatments. • Session 2 addressed the teacher’s role in working with students with ADHD, focusing on the importance of home–school cooperation and using a team approach; target goals were set for the intervention, and the behavior program was introduced. • Session 3 supported teachers as they developed a structured behavior program specific to the goals developed in Session 2; teachers learned to use a reward-based behavior program that was specific to the student to decrease unwanted behavior and increase wanted behavior. • Session 4 guided teachers in structuring their physical  classrooms, providing schoolwork tasks, and  building positive relationships with their students  with ADHD. • Session 5 addressed instructional interventions for ADHD focused on academic and cognitive needs of students with ADHD and co-occurring learning disabilities. • Session 6 supported teachers in improving students’ study skills, meta-cognition, and self-monitoring; it concluded by helping teachers to evaluate progress, phase out the behavior program, and make plans for dealing with relapses of unwanted behaviors  **Arm 2**  Waiting list (WL): waitlist control | **Treatment response** (as a dichotomous outcome)  Not reported/data not abstractable  **All-cause treatment discontinuation** (as a dichotomous outcome)  Yes, reported as study discontinuations/exclusions  **Tolerability** (as a dichotomous outcome)  Not reported  **Serious adverse events** (as dichotomous outcome)  Not reported  **Specific adverse events** (as dichotomous outcome)  Not reported |
| Ghanizadeh et al., 2015^246^ | **Eligibility criteria**  Children aged 5-14 years. Exclusion criteria were: serious medical condition or lack willingness to enter this trial were not entered  **Diagnostic criteria**  DSM-IV  **Number randomized**  106  **Age**  5-14 years  **Co-morbidities/Co-medications**  Not reported – Not reported  **ADHD subtypes**  Not reported | **Arm 1**  MPH-SA low/medium dose+elimination diet: methylphenidate 11.9 mg/day and adjuvant dietary intervention (diary, homemade fruit juices, vegetables, and low-fat meat)  **Arm 2**  MPH-SA low/medium dose: methylphenidate 12.7 mg/day + control diet | **Treatment response** (as a dichotomous outcome)  Not reported/data not abstractable  **All-cause treatment discontinuation** (as a dichotomous outcome)  Yes, reported as study discontinuations/exclusions  **Tolerability** (as a dichotomous outcome)  Not reported  **Serious adverse events** (as dichotomous outcome)  Not reported  **Specific adverse events** (as dichotomous outcome)  Not reported |
| Hiscock et al., 2015^247^ | **Eligibility criteria**  Children aged 5-12 years; had parent reported moderate to severe sleep problems; and met the American Academy of Sleep Medicine diagnostic criteria for at least one sleep disorder (for example, sleep onset association disorder, limit setting disorder, delayed sleep phase, or idiopathic or psychophysiological insomnia) or anxiety leading to insomnia. Exclusion criteria were: families if the child was receiving specialised  sleep assistance from a psychologist or a sleep clinic, or had a serious medical condition (for example, severe cerebral palsy), intellectual disability (paediatrician record of IQ <70), or  suspected obstructive sleep apnoea assessed using the corresponding subscale from the children’s sleep habits questionnaire, and their parents had insufficient English to complete surveys  **Diagnostic criteria**  DSM-IV  **Number randomized**  244  **Age**  5-12 years  **Co-morbidities/Co-medications**  Yes, all comorbid moderate to severe sleep disorders (100%, rated by parentes), learning disability (34%), autism spectrum or Asperger disorder (25%) – Yes, 75% with stimulants (MPH)  **ADHD subtypes**  Not reported | **Arm 1**  BT (parent training): Families in the intervention group were offered two face to face, fortnightly consultations about sleep with a trained clinician (five psychologists; four with 1-4 years of clinical experience and one with 10 years, or a trainee consultant paediatrician with four years of paediatric clinical experience) at their paediatrician’s office, the hospital clinic, or home. Families were offered one follow-up telephone call two weeks later. The clinicians’ training consisted of two three hour sessions, and included information on normal sleep, sleep cycles, sleep cues, sleep hygiene (that is, set bed time, bedtime routines, keeping the bedroom media-free, and avoiding caffeine consumption after 3 pm), and standard management strategies for behaviour known to be effective in typically developing children  **Arm 2**  Standard care (control): usual care | **Treatment response** (as a dichotomous outcome)  ADHD-RS ≥ 30% reduction from baseline in total score (rater: parents and teachers) - unpublished outcome data provided by Prof. Hiscock, correspondence of April 6^th^ 2016  **All-cause treatment discontinuation** (as a dichotomous outcome)  Yes, reported as study discontinuations/exclusions  **Tolerability** (as a dichotomous outcome)  Not reported  **Serious adverse events** (as dichotomous outcome)  Not reported  **Specific adverse events** (as dichotomous outcome)  Not reported |
| Matsudaira et al., 2015^248^ | **Eligibility criteria**  Children and adolescents aged 12-17 years; a mean standardized score >65 (>95th percentile) on both CTRS-L and the CPRS; IQ>70. Exclusion criteria were: female, age <12 or >17; CTRS and CPRS < 65; IQ<70; omega-3 within 3 months; diabetes or other metabolic disorder influencing fatty acid metabolism; Non-English speaker  **Diagnostic criteria**  DSM-IV  **Number randomized**  76  **Age**  12-17 years (range)  **Co-morbidities/Co-medications**  None/Yes, 20% stimulants  **ADHD subtypes**  Combined (66%), inattentive (24%), hyperactive/impulsive (11%) | **Arm 1**  PUFA: polyunsaturated fatty acid (omega-3/6 fatty acid: 558 mg EPA, 174 mg DHA, 60 mg gamma linoleic acid [GLA])  **Arm 2**  Placebo: placebo pill | **Treatment response** (as a dichotomous outcome)  Not reported/data not abstractable  **All-cause treatment discontinuation** (as a dichotomous outcome)  Yes, reported as study drop-outs  **Tolerability** (as a dichotomous outcome)  Yes, reported as study drop-outs due to AEs  **Serious adverse events** (as dichotomous outcome)  Not reported  **Specific adverse events** (as dichotomous outcome)  Not reported |
| Shakibaei et al., 2015^249^ | **Eligibility criteria**  Children and adolescents aged 6-12; the Children's  Global Assessment Scale (CGAS) score of <80 indicating decreased general function. Exclusion criteria were any evidence of mental retardation (IQ ≤70), type I bipolar disorder, psychosis, pervasive developmental disorders, organic brain disease, seizure, or cardiovascular disease  **Diagnostic criteria**  DSM-IV-TR  **Number randomized**  66  **Age**  6-12 years (range)  **Co-morbidities/Co-medications**  No/Not reported  **ADHD subtypes**  Not reported | **Arm 1**  MPH-SA low/medium dose+herbal therapy: methylphenidate 20-30 mg/day with adjunctive *Ginkgo biloba* 80-120 mg/day  **Arm 2**  MPH-SA low/medium dose: methylphenidate 20-30 mg/day | **Treatment response** (as a dichotomous outcome)  Responders based on the achievement of at least a 27% decrease from baseline to endpoint in the ADHD-RS-IV (rater: parents and teachers)  **All-cause treatment discontinuation** (as a dichotomous outcome)  Yes, reported as study discontinuations  **Tolerability** (as a dichotomous outcome)  Yes, reported as study discontinuations due to AEs  **Serious adverse events** (as dichotomous outcome)  Not reported  **Specific adverse events** (as dichotomous outcome)  Loss of appetite/anorexia (as an adverse event)  Cardiovascular event – Palpitations (as an adverse event) |
| Shang et al., 2015^250^ | **Eligibility criteria**  Children and adolescents aged 7-16 years; drug-naïve; a Clinical Global Impressions-Severity Scale (CGI-S) score of at least 4 (moderately ill or worse). Exclusion criteria were: serious medical illness, such as a cardiovascular disease; IQ < 80; had a history of bipolar I or II disorder, psychosis, any substance abuse, or pervasive developmental disorder; had depression or anxiety disorders based on the DSM-IV criteria at study entry; had a history of any seizure disorder or prior electroencephalogram abnormalities related to epilepsy; or had ever used any psychotropic medications before the study  **Diagnostic criteria**  DSM-IV  **Number randomized**  160  **Age**  7-16 years  **Co-morbidities/Co-medications**  None – Not reported  **ADHD subtypes**  Not reported | **Arm 1**  MPH-LA low/medium dose: methylphenidate long acting 27.8 mg/day (0.82 mg/kg/day)  **Arm 2**  ATX: atomoxetine 31.7 mg/day (0.98 mg/kg/day) | **Treatment response** (as a dichotomous outcome)  Not reported/data not abstractable  **All-cause treatment discontinuation** (as a dichotomous outcome)  Yes, reported as study discontinuations  **Tolerability** (as a dichotomous outcome)  Yes, reported as study discontinuations due to AEs  **Serious adverse events** (as dichotomous outcome)  Not reported  **Specific adverse events** (as dichotomous outcome)  Loss of appetite/anorexia (as an adverse event)  Insomnia (as an adverse event)  Cardiovascular event – Palpitations (as an adverse event) |
| Storebø et al., 2015^251,252^ | **Eligibility criteria**  Children aged 8-12 years; and parents willing to take part in the trial and giving consent for medical treatment of the child as well as to participation of the child in the trial. Exclusion criteria were:  schizophrenia or all the autism diagnoses according to DSM IV, violent and criminal children, both verbal and nonverbal IQ<80, previously medicated for ADHD, and resistance against participating  **Diagnostic criteria**  DSM-IV-TR  **Number randomized**  56  **Age**  8-12 years  **Co-morbidities/Co-medications**  Yes, oppositional disorders (15%), anxiety (11%), enuresis (7%), depression (4%) – Not reported  **ADHD subtypes**  Combined (58%), inattentive (29%), hyperactive/impulsive (4%) | **Arm 1**  BT (child and parent training): weekly 90  minute child social-skills training sessions in a total of eight weeks. Each group included two therapists trained in social-skills training before the trial. Therapists from the Langager School in Aarhus  gave continuous supervision throughout the trial. Each session was video recorded, and the therapist completed forms confirming that he/she had followed the manual. These videos and forms were  used to ensure that the planned material in the intervention was being sufficiently implemented. The intervention manual, which may be obtained from the corresponding author, conforms to the  programme of several randomized trials. Different  methods of teaching the children social skills were used. Didactic instructions were used, including work with symbols, games, creative techniques, music, story reading, and movies. Each session had a theme, such as self-worth, nonverbal communication, feelings, impulse control, aggression management, conflict resolution, and problem solving. Many of these  themes are recommended as important topics in social skills training by the NICE guidelines. The treatment focused on strengthening the ability of the children to control them to start a self-help process. During the process where the children received training, the parents attended parental training. The themes from the children’s groups were discussed during the parental groups. The children’s homework was also discussed  **Arm 2**  Standard care (control): usual care | **Treatment response** (as a dichotomous outcome)  Not reported/data not abstractable  **All-cause treatment discontinuation** (as a dichotomous outcome)  Yes, reported as study discontinuations/los to follow-up  **Tolerability** (as a dichotomous outcome)  Not reported  **Serious adverse events** (as dichotomous outcome)  Not reported  **Specific adverse events** (as dichotomous outcome)  Not reported |
| Wilens et al., 2015^253-255^ | **Eligibility criteria**  Children aged 13-17 years; a minimum ADHD-RS-IV total score of 32 and a minimum CGI-S score of 4 at baseline (visit 2); supine and standing blood pressure measurements within the 95th percentile for age, sex, and height were also required. Exclusion criteria were: any current controlled or  uncontrolled comorbid psychiatric diagnosis (except oppositional defiant disorder), including severe comorbid Axis II disorders or severe Axis I disorders, such as anxiety disorder, posttraumatic  stress disorder, depression, bipolar illness, psychosis, pervasive developmental disorder, obsessive-compulsive disorder, substance abuse disorder within 6 months, or other symptomatic manifestations  or lifetime history of bipolar or unipolar illness (e.g., active suicidality), psychosis, or conduct disorder that, in the opinion of the investigator, contraindicated treatment with GXR or could confound efficacy or safety assessments. Other exclusion criteria included history/presence of structural cardiac abnormalities, serious heart rhythm abnormalities, syncope, cardiac conduction problems, exercise-related cardiac events, orthostatic hypotension, history of controlled or uncontrolled hypertension, or clinically significant bradycardia. Participants who used any medications that affect blood pressure or heart rate, have central nervous system effects, or affect cognitive performance (such as sedating antihistamines) were also excluded. Psychosocial treatment was permitted during the study if it had been ongoing for >1 month at the time of the baseline visit, and any changes/modifications to psychosocial treatment during the study had to be cleared by medical staff  **Diagnostic criteria**  DSM-IV-TR  **Number randomized**  314  **Age**  13-17 years (range)  **Co-morbidities/Co-medications**  Yes, oppositional disorders (12%)/Not reported  **ADHD subtypes**  Combined (68%), inattentive (29%), hyperactive/impulsive (3%) | **Arm 1**  GUAN-LA: guanfacine long acting 1-7 mg/day  **Arm 2**  Placebo: placebo pill | **Treatment response** (as a dichotomous outcome)  Global improvement based on Clinical Global Impression Improvement (CGI-I) scale. Subjects with improvement of “very much” or “much” improved were considered responders e.g. GGI-I ≤ 2 (rating evaluations reported by clinician); Subjects with an assessment of normal/borderline mentally ill on Clinical Global Impression-Severity of Illness (CGI-S); Response defined as a percentage reduction from baselines in the ADHD-RS total score of ≥ 30% and CGI-I=1 or 2  **All-cause treatment discontinuation** (as a dichotomous outcome)  Yes, reported as study discontinuations  **Tolerability** (as a dichotomous outcome)  Yes, reported as study discontinuations  **Serious adverse events** (as dichotomous outcome)  Yes, reported  **Specific adverse events** (as dichotomous outcome)  Loss of appetite/anorexia (as an adverse event)  Insomnia (as an adverse event)  Syncope (as an adverse event)  Cardiovascular event – blood pressure decrease (e.g. SBP decrease ≥ 15 mmHG, postural orthostatic blood pressure)  Bradycardia (as an adverse event) |
| Arabgol et al., 2015^256^ | **Eligibility criteria**  Children aged 3-4 years. Exclusion criteria were: the presence of any physical disease, mental retardation and any psychiatric co-morbid disorders except conduct disorder and oppositional defiant disorder  **Diagnostic criteria**  DSM-IV-TR  **Number randomized**  38  **Age**  3-4 years (range)  **Co-morbidities/Co-medications**  Not reported/None  **ADHD subtypes**  Combined (58%), inattentive (9%), hyperactive/impulsive (33%) | **Arm 1**  RISP: risperidone 0.1-1.5 mg/day  **Arm 2**  MPH-SA low/medium dose: methylphenidate 5-20 mg/day | **Treatment response** (as a dichotomous outcome)  Not reported/data not abstractable  **All-cause treatment discontinuation** (as a dichotomous outcome)  Yes, reported as study discontinuations  **Tolerability** (as a dichotomous outcome)  Yes, reported as study discontinuations  **Serious adverse events** (as dichotomous outcome)  Not reported  **Specific adverse events** (as dichotomous outcome)  Loss of appetite/anorexia (as an adverse event)  Sleep disturbances (as an adverse event)  (as an adverse event) |
| Correia-Filho et al., 2005^257^ | **Eligibility criteria**  Children aged 6-16 years. Exclusion criteria were: presence of pervasive developmental disorder, schizophrenia, or other psychotic disorders; seizure disorder requiring medication; history of head injury; previous treatment with MPH or risperidone; use of any psychiatric medication in the month before beginning of the trial  **Diagnostic criteria**  DSM-IV  **Number randomized**  38  **Age**  6-16 years (range)  **Co-morbidities/Co-medications**  Mental retardation/None  **ADHD subtypes**  Combined (69%), inattentive (31%) | **Arm 1**  RISP: risperidone 2.9 mg/day  **Arm 2**  MPH-SA low/medium dose: methylphenidate 5-20 mg/day | **Treatment response** (as a dichotomous outcome)  Not reported/data not abstractable  **All-cause treatment discontinuation** (as a dichotomous outcome)  Yes, reported as study discontinuations  **Tolerability** (as a dichotomous outcome)  Yes, reported as study discontinuations  **Serious adverse events** (as dichotomous outcome)  Not reported  **Specific adverse events** (as dichotomous outcome)  Not reported |
| Ferrin et al., 2016^258^ | **Eligibility criteria**  Children and adolescents aged 5-18; parents’ age greater than or equal to 18 years; responsibility and legal capacity in parents; participant on clinical ADHD symptoms stabilization for at least 1 month before entering the study. Exclusion criteria were: severe intellective disabilities (IQ<70); autistic spectrum disorders; subjects with any clinically significant or unstable medical or psychiatric condition; and children whose families had received any school-based individual and/or group psychosocial treatments at any point in time  **Diagnostic criteria**  DSM-IV  **Number randomized**  69  **Age**  5-18 years (range)  **Co-morbidities/Co-medications**  Yes, oppostional disorders or conduct disorders (91%), dyslexia (32%), anxiety (18%), obsessive compulsive disorders (15%), self-harm (11%), conduct disorders (9%)/Yes, 61%  **ADHD subtypes**  Combined (65%) | **Arm 1**  BT (parent training): The BT/psychoeducation programme was developed according to the basic principles and requirements for an educational programme; it was adapted and implemented from a previous evidence-based programme developed for patients with Bipolar Disorder.The BT group was composed of five successive groups of 10 families who received 6 sessions of 2hr at weekly intervals; families were educated on the disorder during the first nine sessions and finally very briefly introduced to a range of behavioural strategies for managing ADHD symptoms and reducing defiant behaviour during the last three. The integrity of the  BT sessions was guaranteed by a manual that explicitly outlined all the procedures to be used in the  intervention. Sessions were audiotaped and an independent person reviewed through a checklist that the different groups received an equivalent set of information. Parents received no further parental training or behavioural strategies as the aim of the programme was purely educational; they were given the opportunity to express their own experiences and feelings about their child and the impact that the child’s condition had had on them. Two experienced child and adolescent psychiatrists and one psychologist conducted all the sessions.  **Arm 2**  Standard care (control): usual care | **Treatment response** (as a dichotomous outcome)  Not reported/data not abstractable  **All-cause treatment discontinuation** (as a dichotomous outcome)  Yes, reported as study withrawals  **Tolerability** (as a dichotomous outcome)  Not reported  **Serious adverse events** (as dichotomous outcome)  Not reported  **Specific adverse events** (as dichotomous outcome)  Not reported |
| Janssen et al., 2016^259,260^ | **Eligibility criteria**  Children aged 7-13 years;  **Diagnostic criteria**  DSM-IV-TR  **Number randomized**  112  **Age**  7-13 years (range)  **Co-morbidities/Co-medications**  Not reported/Not reported  **ADHD subtypes**  Combined (70%), inattentive (28%), hyperactive/impulsive (1%) | **Arm 1**  MPH-SA low/medium dose: methylphenidate 5-20 mg/day  **Arm 2**  Neurofeedback (theta-beta training): Theta/beta training was applied unidirectionally, with the aim to inhibit theta (4–8 Hz) and reinforce beta (13–20 Hz) activity at Cz. The THERAPRAX_ EEG Biofeedback system (Neuroconn GmbH, Ilmenau, Germany) with a  DC-amplifier and a sampling rate of 128 Hz was used to transmit and analyse the EEG signal. Reference and ground electrodes were attached to right and left mastoids respectively. Electro-oculogram (EOG) was obtained with two electrodes at external canthi, and two electrodes at infra- and supraorbital sides. Subsequently, theta/beta index [theta(lV2/Hz)-beta(lV2/Hz)/ theta(lV2/Hz) + beta(lV2/Hz)] was computed with a shorttime-Fourier transformed moving average for direct feedback. The mean number of training sessions of participants who completed the assessments at postintervention (n = 38) was 29  (M = 28.53, SD = 2.63, range between 19 and 30). Each  training session started with a 1-min baseline theta/beta index measurement, followed by 10 runs of NF. Each run comprised four 30-s epochs.  **Arm 3**  Physical activity: Maximum heart rate (HRmax) was  determined before the start of the first training session. The mean number of completed training sessions was 27  with a minimum of 12 sessions. Each training session started with 5-min of warming up followed by five  2-min exercises at a level of 70–80% of HRmax. After a 5-min break, five 2-min exercises of 80–100% of HRmax were performed. The training finished with a 5 min cool down. Time and heart rate were monitored and registered using POLAR (model FTM4). | **Treatment response** (as a dichotomous outcome)  Not reported/data not abstractable  **All-cause treatment discontinuation** (as a dichotomous outcome)  Yes, reported as study drop-outs  **Tolerability** (as a dichotomous outcome)  Not reported  **Serious adverse events** (as dichotomous outcome)  Not reported  **Specific adverse events** (as dichotomous outcome)  Not reported |
| Steeger et al., 2016^261^ | **Eligibility criteria**  Children and adolescents aged 11-15; Grades 5-9. Exclusion criteria: Autism Spectrum Disorder diagnosis  **Diagnostic criteria**  DSM-IV-TR  **Number randomized**  104  **Age**  11-15 years (range)  **Co-morbidities/Co-medications**  Yes, oppositional disorders (40%), conduct disorders (2%), depression (2%), anxiety (2%), tic disorders (3%), Tourette’s disorder (2%) – Not reported  **ADHD subtypes**  Combined (59%), inattentive (39%), hyperactive/impulsive (2%) | **Arm 1**  BT (parent training): Mothers completed five consecutive, once-weekly, 90-minute parent education sessions. The treatment and active control BT groups met on Sundays (at different times) in the same university classroom. The faculty principal investigator of this study and an advanced doctoral student were the facilitator and cofacilitator, respectively, for both BT conditions. Based on their availability, mothers were alternated into the two meeting times such that the groups were approximately equal. Subsequently, a blinded researcher with no participant contact  randomly assigned treatment to meeting time by a coin flip. BT drew heavily from COPE (Cunningham, 2006), as well as therapy manuals  and parent self-help guides focused on defiant adolescents (Barkley, Edwards, & Robin, 1999; Barkley, Robin, & Benton, 2008). Content was aimed at increasing positive mother adolescent interactions, adolescent compliance, and maternal control, while reducing mother-adolescent conflict and adolescent oppositional and defiant behavior. Sessions were participatory and involved presentations, discussion, and roleplays of specific parenting skills. Weekly homework was assigned to mothers to practice content with their adolescents in between the group sessions  **Arm 2**  WM training: Cognitive training – working memory training. Over 5 weeks, adolescents completed a high- or low-dose version of Cogmed-RM, an at-home, 25-day, computerized WM training program (Cogmed-RM; Cogmed Cognitive Medical Systems AB, Stockholm, Sweden; Pearson Education, Upper Saddle River, NJ). We selected CWMT for the current study because the program is appropriate for use with adolescents, currently has the largest empirical evidence base among cognitive training programs for ADHD and appears to train the attentional control components of WM. Adolescents were randomly assigned to a CWMT condition using a random-numbers table. Each day of training, participants in both conditions completed a total of eight spatial and verbal WM exercises (e.g., remembering the sequence and locations of dots, backward digit span). Each participant completed the same 11 exercises; however, the length of the spatial or verbal list and adaptiveness or nonadaptiveness of the training program varied with version received. In the high-dose, treatment CWMT condition, the length of the list was automatically adjusted by the program on a trial-by-trial basis to match the WM span of the participant on that particular exercise (i.e., adaptive CWMT). In the low-dose, nonadaptive, active control condition, the length of the list was set to a maximum of three, with only three lights, letters, or numbers, maximum, appearing for the duration of the training. All participants completed their exercises on a computer with an Internet connection. The program provided positive feedback verbally after most successful trials. In addition, participants received “energy” on all successful trials that they could use in a video game involving racing robots at the end of each day of training  **Arm 3**  BT (parent training) + WM training: Combined cognitive training (working memory training) and parent traing.  **Arm 4**  Placebo: Placebo BT/WM | **Treatment response** (as a dichotomous outcome)  Not reported/data not abstractable  **All-cause treatment discontinuation** (as a dichotomous outcome)  Yes, reported as study discontinuations  **Tolerability** (as a dichotomous outcome)  Not reported  **Serious adverse events** (as dichotomous outcome)  Not reported  **Specific adverse events** (as dichotomous outcome)  Not reported |
| Su et al., 2016^262^ | **Eligibility criteria**  Children and adolescents aged 6-16 years. For ethical reasons, subjects were excluded if they  had a history of poor response with adequate treatment or intolerance to either MPH or ATX. Subjects who had medical contraindications  to stimulants (i.e., narrow-angle glaucoma, cardiovascular disease) or who had seizure disorder or an abnormal electroencephalogram  (EEG) associated with epilepsy, bipolar disorder,  psychosis, anxiety disorder, depression disorder, TD, pervasive developmental disorder, or an IQ <70 based on the Chinese-Wechsler Intelligence Scale for Children (C-WISC) were excluded from the study. In addition, subjects taking concomitant psychoactive medications including dietary supplements with central nervous system (CNS) activity in the past 30 days were also excluded. Subjects were not allowed to receive psychosocial treatment, such as behavior therapy, for ADHD symptoms during the short-term efficacy study. Behavioral treatments are not readily accessible in China  **Diagnostic criteria**  DSM-IV  **Number randomized**  262  **Age**  6-16 years  **Co-morbidities/Co-medications**  Yes, oppositional disorders (30%) – Not reported  **ADHD subtypes**  Combined (49%), inattentive (48%), hyperactive/impulsive (3%) | **Arm 1**  MPH-LA low/medium dose: methylphenidate long acting 18 mg/day (0.69 mg/kg/day)  **Arm 2**  ATX: atomoxetine 36.7 mg/day (1.01 mg/kg/day) | **Treatment response** (as a dichotomous outcome)  Responders based on the achievement of a 25% decrease from baseline to endpoint in the ADHD-RS total score (rater: investigator/clinician); Clinician-rated Clinical Global Impression Severy (CGI-S) scale – e.g. GGI-S ≤ 2  **All-cause treatment discontinuation** (as a dichotomous outcome)  Yes, reported as study drop-outs  **Tolerability** (as a dichotomous outcome)  Not reported  **Serious adverse events** (as dichotomous outcome)  Not reported  **Specific adverse events** (as dichotomous outcome)  Loss of appetite/anorexia (as an adverse event)  Sleep disturbances (as an adverse event)  Anxiety (as an adverse event) |
| Newcorn et al., 2016^263,264^ | **Eligibility criteria**  Children aged 6-17 years; baseline ADHD-RS-IV with a total score of 32 or higher and a minimum Clinical Global Impression-Severity (CGI-S) score of 4, were enrolled in the study. Those with age appropriate intellectual functioning; blood pressure measurements within the 95th percentile for age, sex and height; and the ability to swallow tablets or capsules were included. Girls of childbearing potential had to have a negative urine pregnancy test at screening and baseline and to comply with any protocol contraceptive requirements. Exclusion criteria were: clinically significant illness, including ac linically significant abnormal screening visit; current, comorbid psychiatric diagnosis (except oppositional defiant disorder); history/presence of cardiac abnormalities, cardiovascular or cerebrovascular disease, serious heart rhythm abnormalities, syncope, tachycardia, cardiac conduction problems, exercise-related cardiac events or syncope; clinically significant bradycardia; orthostatic hypotension or hypertension; seizures; and glaucoma; a patient history of alcohol or substance abuse and those patients with serious tic disorder, including Tourette's syndrome  **Diagnostic criteria**  DSM-IV-TR  **Number randomized**  316  **Age**  6-17 years (range)  **Co-morbidities/Co-medications**  Yes, oppositional disorders (27%)/Not reported  **ADHD subtypes**  Combined (84%), inattentive (12%), hyperactive/impulsive (4%) | **Arm 1**  GUAN-LA: guanfacine long acting 1-7 mg/day  **Arm 2**  Placebo: placebo pill | **Treatment response** (as a dichotomous outcome)  Global improvement based on Clinical Global Impression Severy (CGI-S) scale e.g. subjects with an assessment of normal, not at all ill, borderline mentally ill on Clinical Global Impression-Severity of Illness (CGI-S)  **All-cause treatment discontinuation** (as a dichotomous outcome)  Yes, reported as study early terminations  **Tolerability** (as a dichotomous outcome)  Yes, reported as study early terminations due tu AEs  **Serious adverse events** (as dichotomous outcome)  Yes, reported  **Specific adverse events** (as dichotomous outcome)  Syncope (as an adverse event)  Cardiovascular event – hypotension (as an adverse event) |
