# Supplementary material for: The pharmacological and non-pharmacological treatment of attention deficit hyperactivity disorder in children and adolescents: A systematic review with network meta-analyses of randomised trials
Source: PLoS One. 2017 Jul 12;12(7):e0180355. doi: 10.1371/journal.pone.0180355 (PMC5507500; doi:10.1371/journal.pone.0180355)
Supplement: S3 Table — (DOCX) [file pone.0180355.s008.docx]

**S3 Table. Risk of bias and sponsorship of included studies**

| **Trial name, year** | **Sequence generation**  **(selection bias)** | **Allocation concealment (selection bias)** | **Blinding of participants and personnel** | **Blinding of outcome assessors (detection bias)** | **Incomplete outcome data addressed (attrition bias)** | **Selective outcome reporting (reporting bias)** | **Other sources of bias** | **Overall risk of bias** | **Funding source** |
| --- | --- | --- | --- | --- | --- | --- | --- | --- | --- |
| Gittelman-Klein et al., 1976^1^ | Unclear risk | Unclear risk | Low risk | Unclear risk | Unclear risk | Unclear risk | Unclear risk | Unclear risk | Nonindustry |
| Firestone et al., 1986^2^ | Unclear risk | Unclear risk | Low risk | Low risk | High risk | High risk | Unclear risk | High risk | Nonindustry |
| Casat et al., 1987^3,4^ | Unclear risk | Unclear risk | Unclear risk | Unclear risk | High risk | High risk | Unclear risk | High risk | Industry |
| Kupietz et al., 1988^5^ | Unclear risk | Unclear risk | Unclear risk | Unclear risk | Unclear risk | High risk | Unclear risk | High risk | Nonindustry |
| Biederman et al., 1989^6,7^ | Low risk | Unclear risk | Low risk | Low risk | Unclear risk | Unclear risk | Unclear risk | Unclear risk | Mixed |
| Egger et al., 1992^8^ | Unclear risk | Unclear risk | Unclear risk | Unclear risk | High risk | High risk | Unclear risk | High risk | Nonindustry |
| Gunning 1992^9^ | Low risk | Low risk | Unclear risk | Unclear risk | Unclear risk | Unclear risk | Unclear risk | Unclear risk | Mixed |
| Pisterman 1992^10^ | High risk | High risk | Unclear risk | Unclear risk | High risk | High risk | Unclear risk | High risk | Nonindustry |
| Buitelaar et al., 1996^11^ | Unclear risk | Unclear risk | Unclear risk | Unclear risk | High risk | High risk | Unclear risk | High risk | Not reported |
| Conners et al., 1996^12^ | Unclear risk | Unclear risk | Unclear risk | Unclear risk | High risk | High risk | Unclear risk | High risk | Mixed |
| Schachar et al., 1997^13^ | Unclear risk | Unclear risk | Low risk | Low risk | High risk | High risk | Unclear risk | High risk | Nonindustry |
| Klein and Abikoff, 1997^14^ | Unclear risk | Unclear risk | High risk | High risk | Unclear risk | Unclear risk | Unclear risk | High risk | Not reported |
| Van der Meere et al., 1999^15^ | Unclear risk | Unclear risk | Low risk | Low risk | Unclear risk | Unclear risk | Unclear risk | Unclear risk | Mixed |
| MTA Cooperative, 1999^16-19^ | Low risk | Low risk | High risk | High risk | Low risk | Low risk | Low risk | High risk | Nonindustry |
| Connor et al., 2000^20^ | Unclear risk | High risk | Low risk | Low risk | Unclear risk | Unclear risk | Unclear risk | High risk | Nonindustry |
| Pliszka et al., 2000^21^ | Unclear risk | Unclear risk | Low risk | Low risk | High risk | Unclear risk | Unclear risk | Unclear risk | Industry |
| Prince et al., 2000^22^ | Unclear risk | Unclear risk | Unclear risk | Unclear risk | High risk | High risk | Unclear risk | High risk | Industry |
| Michelson et al., 2001^23-25^ | Unclear risk | Unclear risk | Unclear risk | Unclear risk | Unclear risk | Unclear risk | Unclear risk | Unclear risk | Industry |
| Scahill et al., 2001^26^ | Unclear risk | Unclear risk | Low risk | Low risk | Unclear risk | Unclear risk | Unclear risk | Unclear risk | Nonindustry |
| Sonuga-Barke et al., 2001^27^ | Unclear risk | Unclear risk | Unclear risk | Low risk | Unclear risk | Unclear risk | Unclear risk | Unclear risk | Nonindustry |
| Voigt et al., 2001^28^ | Low risk | Unclear risk | Unclear risk | Unclear risk | Unclear risk | Unclear risk | Unclear risk | Unclear risk | Mixed |
| Wolraich et al., 2001^29^ | Low risk | Low risk | Low risk | Low risk | Low risk | Unclear risk | Unclear risk | Unclear risk | Industry |
| Biederman et al., 2002^31,32^ | Unclear risk | Unclear risk | Unclear risk | Unclear risk | Unclear risk | Unclear risk | Unclear risk | Unclear risk | Industry |
| Bor et al., 2002^33^ | Unclear risk | Unclear risk | Unclear risk | Low risk | Unclear risk | Unclear risk | Unclear risk | Unclear risk | Nonindustry |
| Greenhill et al., 2002^34^ | Unclear risk | Unclear risk | Low risk | Unclear risk | Low risk | Unclear risk | Unclear risk | Unclear risk | Industry |
| Lehmkuhl et al., 2002^35-37^ | Low risk | Low risk | Low risk | Low risk | Low risk | Low risk | Low risk | Low risk | Industry |
| Kratochvil et al., 2002^38,39^ | Unclear risk | Unclear risk | High risk | High risk | Unclear risk | Unclear risk | Unclear risk | High risk | Industry |
| Michelson et al., 2002^40,41^ | Unclear risk | Unclear risk | Unclear risk | Unclear risk | Low risk | Unclear risk | Unclear risk | Unclear risk | Industry |
| Spencer et al., 2002a^42,30^ | Unclear risk | Unclear risk | Unclear risk | Unclear risk | High risk | High risk | Unclear risk | High risk | Industry |
| Spencer et al., 2002b^42,30^ | Unclear risk | Unclear risk | Unclear risk | Unclear risk | High risk | High risk | Unclear risk | High risk | Industry |
| Spencer et al., 2002c^43^ | Low risk | Low risk | Unclear risk | Unclear risk | Unclear risk | Unclea risk | Unclear risk | Unclear risk | Nonindustry |
| TSSG, 2002^44^ | Low risk | Low risk | Low risk | Low risk | Low risk | Low risk | Low risk | Low risk | Nonindustry |
| van Oudheusden et al., 2002^45^ | Unclear risk | Unclear risk | Unclear risk | Unclear risk | Unclear risk | Unclear risk | Unclear risk | Unclear risk | Nonindustry |
| Hazell et al., 2003^46^ | Low risk | Low risk | Low risk | Low risk | Unclear risk | Unclear risk | Unclear risk | Unclear risk | Nonindustry |
| Rugino et al., 2003^47^ | Unclear risk | Unclear risk | Unclear risk | Unclear risk | High risk | High risk | Unclear risk | High risk | Not reported |
| Abikoff et al., 2004^48-50^ | Unclear risk | Unclear risk | High risk | High risk | Unclear risk | Unclear risk | Unclea risk | High risk | Nonindustry |
| Akhondzadeh et al., 2004^51^ | Unclear risk | Unclear risk | Unclear risk | Unclear risk | Unclear risk | Unclear risk | Unclear risk | Unclear risk | Nonindustry |
| Bilici et al., 2004^52^ | Unclear risk | Unclear risk | Unclear risk | Unclear risk | Unclear risk | Unclear risk | Unclear risk | Unclear risk | Nonindustry |
| Döpfner et al., 2004^53^ | Unclear risk | Unclear risk | Unclear risk | Unclear risk | High risk | High risk | Unclear risk | High risk | Nonindustry |
| Kaplan et al., 2004^54^ | Unclear risk | Unclear risk | Unclear risk | Unclear risk | High risk | High risk | High risk | High risk | Industry |
| Kelsey et al., 2004^55,56^ | Unclear risk | Unclear risk | Unclear risk | Unclear risk | Unclear risk | Unclear risk | Unclear risk | Unclear risk | Industry |
| Michelson et al., 2004^57,58^ | Unclear risk | Low risk | Unclear risk | Unclear risk | High risk | High risk | Unclear risk | High risk | Industry |
| Wigal et al., 2004^59^ | Unclear risk | Unclear risk | Low risk | Unclear risk | Unclear risk | Unclear risk | Unclear risk | Unclear risk | Industry |
| Allen et al., 2005^60,61^ | Unclear risk | Low risk | Unclear risk | Unclear risk | Unclear risk | Unclear risk | Unclear risk | Unclear risk | Industry |
| Biederman et al., 2005^62^ | Unclear risk | Unclear risk | Unclear risk | Unclear risk | Unclear risk | Unclear risk | Unclear risk | Unclear risk | Industry |
| Jacobs et al., 2005^63^ | Low risk | Low risk | Low risk | Low risk | Unclear risk | Unclear risk | Unclear risk | Unclear risk | Nonindustry |
| Kemner et al., 2005^64^ | Unclear risk | Unclear risk | High risk | High risk | Unclea risk | Unclear risk | Unclear risk | High risk | Industry |
| Klingberg et al., 2005^65^ | Low risk | Low risk | Low risk | Low risk | Unclear risk | Unclear risk | Low risk | Unclear risk | Nonindustry |
| So, 2005^66,67^ | Low risk | Unclear risk | High risk | High risk | High risk | Unclear risk | Unclear risk | High risk | Nonindustry |
| Starr et al., 2005^68^ | Unclear risk | Unclear risk | High risk | High risk | Unclear risk | Unclear risk | Unclear risk | High risk | Industry |
| Weiss et al., 2005^69-71^ | Low risk | Low risk | Unclear risk | Unclea risk | Unclear risk | Unclear risk | Unclear risk | Unclear risk | Industry |
| Wigal et al., 2005^72^ | Low risk | Unclear risk | Low risk | Low risk | Low risk | Unclear risk | Unclear risk | Unclear risk | Industry |
| Bierderman et al., 2006^73^ | Unclear risk | Unclear risk | Low risk | Low risk | Low risk | Unclear risk | Unclear risk | Unclear risk | Industry |
| Findling et al., 2006^74^ | Unclear risk | Unclear risk | Low risk | Low risk | High risk | Unclear risk | Unclear risk | High risk | Industry |
| Gau et al., 2006^75^ | Unclear risk | Unclear risk | High risk | High risk | Unclear risk | Unclear risk | Unclear risk | High risk | Industry |
| Greenhill et al., 2006^76^ | Unclear risk | Unclea risk | Unclear risk | Unclear risk | Unclear risk | Unclear risk | Unclear risk | Unclear risk | Industry |
| Greenhill et al., 2006b^77^ | Unclear risk | Unclear risk | Unclear risk | Unclear risk | Unclear risk | Unclear risk | Unclear risk | Unclear risk | Industry |
| Greenhill et al., 2006c^78-80^ | Unclear risk | Unclear risk | Unclear risk | Unclear risk | Low risk risk | Unclear risk | Unclear risk | Unclear risk | Nonindustry |
| Sangal et al., 2006^81^ | Unclear risk | Unclear risk | Unclear risk | Unclear risk | Unclear risk | High risk | Unclear risk | High risk | Industry |
| Spencer et al., 2006^82^ | Unclear risk | Unclear risk | Unclear risk | Unclear risk | Unclear risk | Unclear risk | Unclear risk | Unclear risk | Industry |
| Spencer et al., 2006b^83^ | Unclear risk | Unclear risk | Unclear risk | Unclear risk | High risk | High risk | Unclear risk | High risk | Industry |
| Steele et al., 2006^84^ | Unclear risk | Unclear risk | High risk | High risk | High risk | High risk | Unclear risk | High risk | Industry |
| Trebatická et al., 2006^85,86^ | Unclear risk | Unclear risk | Unclear risk | Unclear risk | Unclear risk | High risk | High risk | High risk | Industry |
| Armenteros et al., 2007^87^ | Low risk | Low risk | Low risk | Low risk | Unclear risk | High risk | Unclear risk | High risk | Industry |
| Arnold et al., 2007^88^ | Unclear risk | Unclear risk | Unclear risk | Unclear risk | Unclear risk | Unclear risk | Unclear risk | Unclear risk | Industry |
| Bangs et al., 2007^89,90^ | Unclear risk | Unclear risk | Unclear risk | Unclear risk | Unclear risk | Unclear risk | Unclear risk | Unclear risk | Industry |
| Biederman et al., 2007^91,92^ | Low risk | Unclear risk | Unclear risk | Unclear risk | Unclear risk | High risk | Unclear risk | High risk | Industry |
| Buitelaar et al., 2007^93,58^ | Unclear risk | Low risk | Unclear risk | Unclear risk | High risk | High risk | Unclear risk | High risk | Industry |
| Carlson et al., 2007^94^ | Unclear risk | Unclear risk | Unclear risk | Unclear risk | Unclear risk | High risk | Unclear risk | High risk | Industry |
| Gau et al., 2007^95,96^ | Low risk | Low risk | Unclear risk | Unclear risk | Unclear risk | Unclear risk | Unclear risk | Unclear risk | Industry |
| Geller et al., 2007^97,98^ | Unclear risk | Unclear risk | Unclear risk | Unclear risk | Unclear risk | Unclear risk | Unclear risk | Unclear risk | Industry |
| Prasad et al., 2007^99,100^ | Unclear risk | Unclear risk | High risk | High risk | Unclear risk | High risk | Unclear risk | High risk | Industry |
| van den Hoofdakker et al., 2007^101^ | Unclear risk | Unclear risk | Unclear risk | High risk | Unclear risk | Unclear risk | Unclear risk | High risk | Nonindustry |
| van der Oord et al., 2007^102^ | Unclear risk | Unclear risk | High risk | High risk | Unclear risk | Unclear risk | Unclear risk | High risk | Not reported |
| Wang et al., 2007^103,104^ | Unclear risk | Unclear risk | Unclear risk | Unclear risk | Low risk | Low risk | Unclear risk | Unclear risk | Industry |
| Amiri et al., 2008^105^ | Low risk | Low risk | Low risk | Low risk | Low risk | Low risk | Unclear risk | Unclear risk | Non industry |
| Bangs et al., 2008^106,107^ | Unclear risk | Unclear risk | Low risk | Low risk | Unclear risk | Unclear risk | Unclear risk | Unclear risk | Industry |
| Bierdeman et al., 2008^108-110^ | Unclear risk | Unclear risk | Low risk | Low risk | High risk | High risk | Unclear risk | High risk | Industry |
| Palumbo et al., 2008^111-113^ | Low risk | Low risk | Low risk | Low risk | Unclear risk | Unclear risk | Unclear risk | Unclear risk | Nonindustry |
| Findling et al., 2008^114^ | Low risk | Low risk | Low risk | Low risk | High risk | Unclear risk | Unclear risk | High risk | Industry |
| Heriot et al., 2008^115^ | High risk | Unclear risk | Low risk | Low risk | High risk | Unclear risk | Unclear risk | High risk | Nonindustry |
| Konofal et al., 2008^116^ | Unclear risk | Unclear risk | Unclear risk | Unclear risk | Unclear risk | Unclear risk | Unclear risk | Unclear risk | Industry |
| Newcorn et al., 2008^117,118^ | Unclear risk | Unclear risk | Low risk | Low risk | Low risk | Low risk | Unclear risk | Unclear risk | Industry |
| Torrioli et al., 2008^119^ | Unclear risk | Unclear risk | Unclear risk | Unclear risk | High risk | High risk | Unclear risk | High risk | Industry |
| Vaisman et al., 2008^120^ | Low risk | Unclear risk | Unclear risk | Unclear risk | Low risk | Unclea risk | Unclear risk | Unclear risk | Industry |
| Weber et al., 2008^121^ | Low risk | Low risk | Low risk | Low risk | Low risk | Low risk | Low risk | Low risk | Nonindustry |
| Arabgol et al., 2009^122^ | Unclear risk | Low risk | Unclear risk | Unclear risk | High risk | Unclear risk | Unclear risk | High risk | Nonindustry |
| Block et al., 2009^123,124^ | Low risk | Low risk | Unclear risk | Unclear risk | Low risk | Low risk | Unclear risk | Unclear risk | Industry |
| Childress et al., 2009^125^ | Low risk | Low risk | Unclear risk | Unclear risk | Low risk | Low risk | Unclear risk | Unclear risk | Industry |
| Dell'Agnello et al., 2009^126^ | Unclear risk | Unclear risk | Low risk | Low risk risk | Unclear risk | Unclear risk | Unclear risk | Unclear risk | Industry |
| Johnson et al., 2009^127^ | Unclear risk | Low risk | Low risk | Unclear risk | Unclear risk | Unclear risk | Unclear risk | Unclear risk | Not reported |
| Kahbazi et al., 2009^128^ | Low risk | Low risk | Low risk | Low risk | Low risk | Low risk | Unclear risk | Unclear risk | Nonindustry |
| Montoya et al., 2009^129,130^ | Low risk | Low risk | Low risk | Unclear risk | Low risk | Unclear risk | Unclear risk | Unclear risk | Industry |
| Nair et al., 2009^131^ | Low risk | Unclear risk | Unclear risk | Unclear risk | High risk | High risk | Unclear risk | High risk | Not reported |
| Pelsser et al., 2009^132^ | Unclear risk | Unclear risk | High risk | High risk | Low risk | Unclear risk | Unclear risk | High risk | Nonindustry |
| Raz et al., 2009^133^ | Unclear risk | Unclear risk | Unclear risk | Low risk | High risk | High risk | Unclear risk | High risk | Nonindustry |
| Sallee et al., 2009^134-136^ | Unclear risk | Unclear risk | Low risk | Unclear risk | Low risk | Unclear risk | Unclear risk | Unclear risk | Industry |
| Svanborg et al., 2009^137,138^ | Unclear risk | Unclear risk | Unclear risk | Unclear risk | High risk | High risk | Unclear risk | High risk | Industry |
| Takahashi et al., 2009^139,140^ | Unclear risk | Unclear risk | Unclear risk | Unclear risk | High risk | High risk | Unclear risk | High risk | Industry |
| Perez-Alvarez et al., 2009^141^ | Unclear risk | Unclear risk | High risk | High risk | Low risk | Unclear risk | Unclear risk | High risk | Nonindustry |
| Thompson et al., 2009^142^ | Unclear risk | Unclear risk | Unclear risk | Low risk | Low risk | Unclear risk | Unclear risk | Unclear risk | Nonindustry |
| Tramontina et al., 2009^143^ | Unclear risk | Unclear risk | Low risk | Low risk | Low risk | High risk | Unclear risk | High risk | Mixed |
| Tucker et al., 2009^144^ | Unclear risk | Unclear risk | High risk | High risk | Unclear risk | Unclear risk | Unclear risk | High risk | Industry |
| Gevensleben et al., 2009^145-147^ | Low risk | Low risk | High risk | High risk | Low risk | Unclear risk | Unclear risk | High risk | Nonindustry |
| Connor et al., 2010^148,149^ | Low risk | Low risk | Low risk | Unclear risk | Low risk | Unclear risk | Unclear risk | Unclear risk | Industry |
| Fabiano et al., 2010^150^ | Unclear risk | Unclear risk | High risk | High risk | Low risk | Unclear risk | Unclear risk | High risk | Nonindustry |
| Findling et al., 2010^151^ | Low risk | Low risk | Low risk | Low risk | High risk | High risk | Unclear risk | High risk | Industry |
| Gustafsson et al., 2010^152^ | Low risk | Unclear risk | Unclear risk | Low risk | High risk | High risk | Unclear risk | High risk | Mixed |
| Martenyi et al., 2010^153,154^ | Unclear risk | Unclear risk | Unclear risk | Unclear risk | Low risk | Unclear risk | High risk | Unclear risk | Industry |
| Perreau-Linck et al., 2010^155^ | Unclear risk | Unclear risk | High risk | Unclear risk | Low risk | Unclear risk | Unclear risk | High risk | Nonindustry |
| Salehi et al., 2010^156^ | Unclear risk | Unclear risk | Low risk | Low risk | Low risk | Unclear risk | Unclear risk | Unclear risk | Nonindustry |
| Thurstone et al., 2010^157,158^ | Unclear risk | Unclear risk | Unclear risk | High risk | Low risk | Unclear risk | Unclear risk | High risk | Mixed |
| Waxmonsky et al., 2010^159^ | Unclear risk | Unclear risk | High risk | High risk | Unclear risk | High risk | Unclear risk | High risk | Industry |
| Zarinara et al., 2010^160^ | Unclear risk | Unclear risk | Low risk | Low risk | Low risk | Unclear risk | Unclear risk | Unclear risk | Nonindustry |
| Abbasi et al., 2011^161^ | Unclear risk | Unclear risk | Low risk | Low risk | Low risk | Unclear risk | Unclear risk | Unclear risk | Nonindustry |
| Arnold et al., 2011^162^ | Unclear risk | Unclear risk | Unclear risk | Unclear risk | Unclear risk | Unclear risk | Unclear risk | Unclear risk | Nonindustry |
| Bakhshayesh et al., 2011^163^ | Unclear risk | Unclear risk | Low risk | High risk | Unclear risk | Unclear risk | Unclear risk | High risk | Not reported |
| Dittmann et al., 2011^164^ | Unclear risk | Unclear risk | Unclear risk | Unclear risk | Unclear risk | High risk | Unclear risk | High risk | Industry |
| Findling et al., 2011^165,166^ | Low risk | Low risk | Unclear risk | Unclear risk | Low risk | Unclear risk | Unclear risk | Unclear risk | Industry |
| Jain et al., 2011^167^ | Unclear risk | Unclear risk | Unclear risk | Unclear risk | Unclear risk | High risk | Unclear risk | High risk | Industry |
| Giblin et al., 2011^168^ | Unclear risk | Unclear risk | Unclear risk | Unclear risk | High risk | High risk | High risk | High risk | Industry |
| Kang et al., 2011^169^ | Unclear risk | Unclear risk | High risk | High risk | Unclear risk | High risk | Unclear risk | High risk | Nonindustry |
| Kollins et al., 2011^170^ | Low risk | Low risk | Low risk | Low risk | Unclear risk | Unclear risk | Unclear risk | Unclear risk | Industry |
| Kollins et al., 2011^171^ | Unclear risk | Unclear risk | Low risk | Low risk | Low risk | Unclear risk | Unclear risk | Unclear risk | Industry |
| Kratochvil et al., 2011^172,173^ | Unclear risk | Unclear risk | Unclear risk | Unclear risk | Unclear risk | Unclear risk | Unclear risk | Unclear risk | Mixed |
| Lansbergen et al., 2011^174^ | Unclear risk | Unclear risk | Low risk | Low risk | Unclear risk | High risk | High risk | High risk | Nonindustry |
| Pelsser et al., 2011^175^ | Low risk | Low risk | Unclear risk | Unclear risk | Low risk | Unclear risk | Unclear risk | Unclear risk | Nonindustry |
| Riggs et al., 2011^176^ | Low risk | Low risk | Low risk | Low risk | Low risk | Low risk | Low risk | Low risk | Nonindustry |
| Steiner et al., 2011^177^ | Unclear risk | Unclear risk | Unclear risk | Unclear risk | Low risk | Unclear risk | Unclear risk | Unclear risk | Nonindustry |
| Wehmeier et al., 2011^178,179^ | Unclear risk | Unclear risk | Unclear risk | Unclear risk | Unclear risk | High risk | Unclear risk | High risk | Industry |
| Wilens et al., 2011^180^ | Unclear risk | Unclear risk | Unclear risk | Unclear risk | Unclear risk | High risk | Unclear risk | High risk | Industry |
| Yildiz et al., 2011^181^ | Unclear risk | Unclear risk | High risk | High risk | Unclear risk | Unclear risk | Unclear risk | High risk | Not reported |
| Zamora et al., 2011^182^ | High risk | Unclear risk | Unclear risk | Unclear risk | High risk | High risk | Unclear risk | High risk | Nonindustry |
| Assareh et al., 2012^183^ | Unclear risk | Unclear risk | Low risk | Low risk | Unclear risk | Unclear risk | Unclear risk | Unclear risk | Nonindustry |
| Duric et al., 2012^184^ | Unclear risk | Unclear risk | High risk | High risk | Unclear risk | Unclear risk | Unclear risk | High risk | Nonindustry |
| Fabiano et al., 2012^185^ | Unclear risk | Unclear risk | Unclear risk | High risk | Low risk | Unclear risk | Unclear risk | High risk | Nonindustry |
| Green et al., 2012^186^ | Unclear risk | Unclear risk | Unclear risk | Unclear risk | High risk | High risk | Unclear risk | High risk | Not reported |
| Jafarinia et al., 2012^187^ | Low risk | Low risk | Low risk | Low risk | Low risk | Low risk | Low risk | Low risk | Nonindustry |
| Manor et al., 2012^188,189^ | Low risk | Unclear risk | Unclear risk | Unclear risk | Low risk | High risk | Unclear risk | High risk | Industry |
| Perera et al., 2012^190^ | Unclear risk | Unclear risk | Low risk | Low risk | High risk | High risk | Unclear risk | High risk | Industry |
| Wilens et al., 2012^191-194^ | Unclear risk | Unclear risk | Unclear risk | Unclear risk | Low risk | Unclear risk | Unclear risk | Unclear risk | Industry |
| Abikoff et al., 2013^195^ | Unclear risk | Unclear risk | Unclear risk | Low risk | Low risk | Unclear risk | Unclear risk | Unclear risk | Nonindustry |
| Arnold et al., 2013^196^ | Unclear risk | Unclear risk | Unclear risk | Unclear risk | Low risk | Unclear risk | Unclear risk | Unclear risk | Nonindustry |
| Coghill et al., 2013^197-200^ | Unclear risk | Low risk | Low risk | Unclear risk | High risk | High risk | Unclear risk | High risk | Industry |
| Dittmann et al., 2013^201-204^ | Unclear risk | Low risk | Low risk | Unclear risk | Unclear risk | Unclear risk | Unclear risk | Unclear risk | Industry |
| Hovik et al., 2013^205,206^ | Unclear risk | Unclear risk | High risk | High risk | Low risk | Unclear risk | Unclear risk | High risk | Nonindustry |
| Li et al., 2013^207^ | Low risk | Low risk | Low risk | Low risk | Low risk | Unclear risk | Unclear risk | Unclear risk | Nonindustry |
| Newcorn et al., 2013^208-211^ | Unclear risk | Unclear risk | Low risk | Low risk | Unclear risk | High risk | Unclear risk | High risk | Industry |
| Ghanizadeh et al., 2013^212^ | Unclear risk | Unclear risk | Low risk | Low risk | High risk | Unclear risk | Unclear risk | High risk | Not reported |
| Oberai et al., 2013^213^ | Low risk | Low risk | High risk | Unclear risk | Unclear risk | High risk | Unclear risk | High risk | Nonindustry |
| Ogrim et al., 2013^213^ | Unclear risk | Unclear risk | High risk | High risk | High risk | High risk | Unclear risk | High risk | Nonindustry |
| Simonoff et al., 2013^214^ | Unclear risk | Unclear risk | Unclear risk | Unclear risk | Low risk | Unclear risk | Unclear risk | Unclear risk | Nonindustry |
| Tamm et al., 2013^215^ | Unclear risk | Unclear risk | High risk | High risk | Unclear risk | Unclear risk | Unclear risk | High risk | Nonindustry |
| van Dongen-Boomsma et al., 2013^217^ | Unclear risk | Unclear risk | Low risk | Low risk | Low risk | Unclear risk | Unclear risk | Unclear risk | Nonindustry |
| Aman et al., 2014^218-220^ | Low risk | Low risk | Unclear risk | Low risk | Low risk | Unclear risk | Unclear risk | Unclear risk | Nonindustry |
| Barragán et al., 2014^221^ | Unclear risk | Unclear risk | High risk | High risk | High risk | High risk | High risk | High risk | Industry |
| Chacko et al., 2014^222^ | Unclear risk | Unclear risk | Low risk | Low risk | Unclear risk | Unclear risk | Unclear risk | Unclear risk | Nonindustry |
| Ferrin et al., 2014^223^ | Low risk | Low risk | Low risk | Low risk | Low risk | Unclear risk | Unclear risk | Unclear risk | Nonindustry |
| Garg et al., 2014^224^ | Unclear risk | Unclear risk | High risk | High risk | High risk | High risk | Unclear risk | High risk | Nonindustry |
| Hervas et al., 2014^225-227^ | Unclear risk | Unclear risk | Low risk | Low risk | Unclear risk | Unclear risk | Unclear risk | Unclear risk | Industry |
| Hirayama et al., 2014^228^ | Unclear risk | Unclear risk | Unclear risk | Unclear risk | High risk | Unclear risk | Unclear risk | High risk | Industry |
| Ko et al., 2014^229^ | Unclear risk | Unclear risk | Low risk | Low risk | Low risk | Unclear risk | Unclear risk | Unclear risk | Industry |
| Lin et al., 2014^230,231^ | Unclear risk | Unclear risk | Unclear risk | Unclear risk | High risk | High risk | High risk | High risk | Industry |
| Meisel et al., 2014^232^ | Low risk | Unclear risk | High risk | High risk | High risk | High risk | Unclear risk | High risk | Nonindustry |
| Pfiffner et al., 2014^233^ | Unclear risk | Unclear risk | High risk | High risk | Low risk | Low risk | Unclear risk | High risk | Nonindustry |
| Steiner et al., 2014^234,235^ | Unclear risk | Unclear risk | Unclear risk | High risk | Low risk | Unclear risk | Unclear risk | High risk | Nonindustry |
| van Dongen-Boomsma et al., 2014^236^ | Unclear risk | Unclear risk | Low risk | Low risk | Unclear risk | Unclear risk | Unclear risk | Unclear risk | Nonindustry |
| Widenhorn-Müller et al., 2014^237^ | Low risk | Unclear risk | Low risk | Low risk | Unclear risk | Unclear risk | Unclear risk | Unclear risk | Nonindustry |
| Abikoff et al., 2015^238^ | Unclear risk | Unclear risk | Unclear risk | Unclear risk | Low risk | Unclear risk | Unclear risk | High risk | Nonindustry |
| Bigorra et al., 2015^239^ | Unclear risk | Unclear risk | Low risk | Low risk | Low risk | Unclear risk | Unclear risk | Unclear risk | Nonindustry |
| Bédard et al., 2015^240^ | Unclear risk | Unclear risk | Unclear risk | Unclear risk | Unclear risk | Unclear risk | Unclear risk | Unclear risk | Nonindustry |
| Bos et al., 2015^241^ | Unclear risk | Unclear risk | Unclear risk | Unclear risk | Unclear risk | Unclear risk | Unclear risk | Unclear risk | Industry |
| Choi et al., 2015^242^ | Unclear risk | Unclear risk | High risk | High risk | Unclear risk | Unclear risk | Unclear risk | High risk | Nonindustry |
| Choi et al., 2015^243^ | Unclear risk | Unclear risk | High risk | High risk | High risk | Unclear risk | Unclear risk | High risk | Nonindustry |
| Chou et al., 2015^244^ | Low risk | Unclear risk | High risk | High risk | High risk | High risk | Unclear risk | High risk | Nonindustry |
| Corkum et al., 2015^245^ | Low risk | Low risk | High risk | Low risk | Unclear risk | Unclear risk | Unclear risk | High risk | Nonindustry |
| Ghanizadeh et al., 2015^246^ | Unclear risk | Unclear risk | Unclear risk | Unclear risk | Unclear risk | Unclear risk | High risk | High risk | Nonindustry |
| Hiscock et al., 2015^247^ | Low risk | Low risk | High risk | Low risk | Low risk | Unclear risk | Unclear risk | High risk | Nonindustry |
| Matsudaira et al., 2015^248^ | Unclear risk | Low risk | Unclear risk | Unclear risk | High risk | High risk | High risk | High risk | Industry |
| Shakibaei et al., 2015^249^ | Low risk | Low risk | Unclear risk | Low risk | High risk | High risk | Unclear risk | High risk | Nonindustry |
| Shang et al., 2015^250^ | Unclear risk | Unclear risk | High risk | High risk | High risk | High risk | High risk | High risk | Nonindustry |
| Storebø et al., 2015^251,252^ | Low risk | Low risk | High risk | Low risk | Low risk | Unclear risk | Unclear risk | High risk | Nonindustry |
| Wilens et al., 2015^253-255^ | Unclear risk | Unclear risk | Unclear risk | Unclear risk | Low risk | Unclear risk | Unclear risk | Unclear risk | Industry |
| Arabgol et al., 2015^256^ | Unclear risk | Unclear risk | Unclear risk | Unclear risk | High risk | High risk | Unclear risk | High risk | Industry |
| Correia-Filho et al., 2005^257^ | Unclear risk | Unclear risk | High risk | High risk | High risk | High risk | Unclear risk | High risk | Industry |
| Ferrin et al., 2016^258^ | Low risk | Low risk | Unclear risk | High risk | Low risk | Unclear risk | Unclear risk | Unclear risk | Nonindustry |
| Janssen et al., 2016^259,260^ | Unclear risk | Unclear risk | Unclear risk | Unclear risk | Low risk | Unclear risk | Unclear risk | Unclear risk | Nonindustry |
| Steeger et al., 2016^261^ | Unclear risk | Unclear risk | Unclear risk | Unclear risk | Unclear risk | Unclear risk | Unclear risk | Unclear risk | Nonindustry |
| Su et al., 2016^262^ | Unclear risk | Unclear risk | High risk | High risk | High risk | High risk | High risk | High risk | Mixed |
| Newcorn et al., 2016^263,264^ | Low risk | Unclear risk | Unclear risk | Unclear risk | Unclear risk | Unclear risk | High risk | High risk | Industry |
