# Supplementary material for: The pharmacological and non-pharmacological treatment of attention deficit hyperactivity disorder in children and adolescents: A systematic review with network meta-analyses of randomised trials
Source: PLoS One. 2017 Jul 12;12(7):e0180355. doi: 10.1371/journal.pone.0180355 (PMC5507500; doi:10.1371/journal.pone.0180355)
Supplement: S4 Table — (DOCX) [file pone.0180355.s009.docx]

**S4 Table. Number of events per trial and treatment comparison for response, all-cause discontinuation, tolerability and serious AEs.**

| **Trial name, year** | **Treatment comparisons** | **Treatment response** | **All-cause discontinuation** | **Tolerability** | **Serious adverse events** |
| --- | --- | --- | --- | --- | --- |
| Gittelman-Klein et al., 1976^1^ | Stimulant (MPH-SA); Antipsychotic (THIO); Stimulant+antipsychotic (MPH-SA+THIO); Placebo | CGI-I Clinician: 26/41; 8/41; 24/42; 5/42  CGI-I Parents: 24/41; 16/41; 30/42; 9/42  CGI-I Teacher: 23/41; 17/41; 26/42; 3/42 | 2/41; 5/41; 3/42; 1/42 | 1/41; 3/41; 3/42; 0/42 | - |
| Firestone et al., 1986^2^ | Stimulant (MPH-SA); BT (parent training); Stimulant+BT (MPH-SA+parent training) | - | 12/30; 8/21; 10/22 | - | - |
| Casat et al., 1987^3,4^ | Antidepressant (BUP); Placebo | - | 1/20; 1/10 | 1/20; 0/10 | - |
| Kupietz et al., 1988^5^ | Stimulant (MPH-SA); Placebo | - | 7/42; 4/16 | - | - |
| Biederman et al., 1989^6,7^ | Antidepressant (DESIP); Placebo | CGI-I Clinician: 21/37; 3/36  CGI-S Clinician: 11/37; 3/36  Conners’ Parents: 13/37; 4/36 | 6/37; 5/36 | 1/37; 0/36 | - |
| Egger et al., 1992^8^ | Restricted elimination diet (oligoantigenic diet); Placebo | - | 2/20; 1/20 | - | - |
| Gunning 1992^9^ | Stimulant (MPH-SA); Non-stimulant α-2 agonist (CLON-SA); Placebo | CGI Clinician: 12/24; 16/42; 8/43 | 0/24; 0/42; 1/43 | - | - |
| Pisterman 1992^10^ | BT (parent training); Waiting list | CGI Parents: 15/28; 6/29 | 5/28; 7/29 | - | - |
| Buitelaar et al., 1996^11^ | Stimulant (MPH-SA); β-blocker (PINDO); Placebo | - | - | - | - |
| Conners et al., 1996^12^ | Antidepressant (BUP); Placebo | - | 8/72; 2/37 | 4/72; 0/37 | - |
| Schachar et al., 1997^13^ | Stimulant+BT (MPH-SA+parent training); BT (parent training) | - | 9/46; 16/45 | 5/46; 1/45 | - |
| Klein and Abikoff, 1997^14^ | Stimulant (MPH-SA); BT (parent and teacher training); Stimulant+BT (MPH-SA+parent and teacher training) | CGI-I Clinician: 23/30; 14/29; 28/30  CGI-I Parents: 22/30; 18/29; 27/30  CGI-I Teacher: 20/30; 18/29; 27/30 | 1/30; 1/29; 1/30 | 1/30; 0/29; 0/30 | - |
| Van der Meere et al., 1999^15^ | Stimulant (MPH-SA); Non-stimulant α-2 agonist (CLON-SA); Placebo | CGI Parents/teachers: 10/18; 8/17; 2/18 | - | - | - |
| MTA Cooperative, 1999^16-19^ | Stimulant (MPH-SA); BT (child, parent and teacher training); Stimulant+BT (MPH-SA+child, parent and teacher training); Standard care | SNAP Parents/teachers (36w):  77/144; 40/144; 91/145; 34/146  SNAP Parents/teachers (56w):  81/144; 49/144; 99/145; 37/146 | 8/144; 3/144; 3/145; 6/146 | - | - |
| Connor et al., 2000^20^ | Stimulant (MPH-SA); Non-stimulant α-2 agonist (CLON-SA); Stimulant (MPH-SA) + non-stimulant α-2 agonist (CLON-SA) | - | 1/8; 2/8; 0/8 | - | - |
| Pliszka et al., 2000^21^ | Stimulant (MPH-SA); Stimulant (MIX-AMPH); Placebo | CGI-I Clinician: 13/20; 18/20; 5/18 | 1/20; 2/20; 2/18 | 1/20; 2/20; 0/18 | - |
| Prince et al., 2000^22^ | Antidepressant (NT); Placebo | CGI-I Clinician: 8/12; 2/11 | - | - | - |
| Michelson et al., 2001^23-25^ | Non-stimulant (ATX); Placebo | - | 37/213; 12/84 | 7/213; 0/84 | 4/213; 1/84 |
| Scahill et al., 2001^26^ | Non-stimulant α-2 agonist (GUAN-SA); Placebo | CGI-I Clinician: 9/17; 0/17  ADHD-RS Teachers: 6/17; 1/17  Conners Parent Hyperactivity index: 5/17; 4/17 | 1/17; 0/17 | 1/17; 0/17 | - |
| Sonuga-Barke et al., 2001^27^ | BT (parent training); Control (parent counseling with support); Waiting list | PACS Parents: 16/30; 11/28; 5/20 | - | - | - |
| Voigt et al., 2011^28^ | Stimulant (MPH-SA)+PUFA (omega-3 fatty acid); Stimulant (MPH-SA)+Placebo | - | 5/32; 4/31 | - | - |
| Wolraich et al., 2001^29^ | Stimulant (MPH-SA); Stimulant (MPH-LA); Placebo | CGI-I Clinician: 44/97; 44/95; 15/90  Global assessment Parents: 44/97; 51/95; 18/90  Global assessment Teachers: 44/97, 40/95; 16/90 | 13/97; 15/95; 43/90 | 1/97; 1/95; 1/90 | - |
| Biederman et al., 2002^31,32^ | Stimulant (MIX-AMPH-LA); Placebo | CGI-I Clinician: 220/374; 37/210  Global assessment Parents: 219/374; 36/210 | 38/374; 37/210 | 9/374; 6/210 | - |
| Bor et al., 2002^33^ | BT (parent training); Waiting list | - | 19/55; 5/32 | - | - |
| Greenhill et al., 2002^34^ | Stimulant (MPH-INT); Placebo | CGI-I Clinician: 125/158; 78/163  CGI-S Clinician: 98/158; 41/163 | 17/158; 28/163 | 2/158; 0/163 | - |
| Lehmkuhl et al., 2002^35-37^ | Stimulant (MPH-LA); Placebo | ADHD-RS Teachers: 31/43; 11/42 | 3/43; 4/42 | 2/43; 0/42 | 1/43; 0/42 |
| Kratochvil et al., 2002^38,39^ | Stimulant (MPH-SA); Non-stimulant (ATX) | CGI-I Clinician: 26/44; 115/184  CGI-S Clinician: 18/44; 64/184 | 19/44; 66/184 | 5/44; 10/184 | - |
| Michelson et al., 2002^40,41^ | Non-stimulant (ATX); Placebo | CGI-S Clinician: 24/85; 8/86  ADHD-RS Parents: 50/85; 26/86 | 12/85; 11/86 | 2/85; 1/86 | - |
| Spencer et al., 2002a^42,30^ | Stimulant (MPH-SA); Non-stimulant (ATX); Placebo | ADHD-RS Parents: -; 33/56; 21/53 | 12/38; 13/56; 9/53 | -; 6/129; 3/124  (pooled Spencer 2002a and 2000b) | - |
| Spencer et al., 2002b^42,30^ | Non-stimulant (ATX); Placebo | ADHD-RS Parents: 47/73; 17/71 | 14/73; 23/71 | = | - |
| Spencer et al., 2002c^43^ | Antidepressant (DESIP); Placebo | CGI-I Clinician: 15/21; 0/20  ADHD-RS Parents: 16/21; 1/20 | 0/21; 2/20 | 0/21; 2/20 | - |
| TSSG, 2002^44^ | Stimulant (MPH-SA); Non-stimulant α-2 agonist (CLON-SA); Stimulant (MPH-SA) + non-stimulant α-2 agonist (CLON-SA); Placebo | CGI Clinician: 22/37; 21/34; 29/33; 10/32  CGI Parents: 25/37; 19/34; 29/33; 10/32  CGI Teacher: 28/37; 21/34; 28/33; 12/32 | 4/37; 4/34; 4/33; 7/32 | 0/37; 0/34; 1/33; 0/32 | - |
| van Oudheusden et al., 2002^45^ | Aminoacids (l-carnitine); Placebo | - | 1/13; 1/13 | 1/13; 0/13 | - |
| Hazell et al., 2003^46^ | Stimulant (MPH-SA or DEXAM); Stimulant (MPH-SA or DEXAM) + non-stimulant α-2 agonist (CLON-SA) | Conners Parents: 12/29; 26/38  Conners Parent Hyperactivity index: 11/29; 24/38 | 4/29; 3/38 | - | - |
| Rugino et al., 2003^47^ | Other unlicensed drug (MODAF); Placebo | - | 2/13; 0/11 | 1/13; 0/11 |  |
| Abikoff et al., 2004^48-50^ | Stimulant (MPH-SA); Stimulant+BT (MPH-SA+child and parent training); Stimulant+control (MPH-SA+attential control psychosocial therapy) | Clinician assessment (24w):  23/34; 25/34; 26/35  Clinician assessment (96w):  20/34; 28/34; 24/35 | 10/34; 6/34; 6/35 | - | - |
| Akhondzadeh et al., 2004^51^ | Stimulant (MPH-SA); Stimulant+minerals (MPH-SA+Zinc sulfate) | - | 2/22; 2/22 | - | - |
| Bilici et al., 2004^52^ | Minerals (Zinc sulfate); Placebo | ADHD-RS Clinician: 45/202; 21/198 | 107/202; 100/198 | 25/202; 17/202 | - |
| Döpfner et al., 2004^53^ | Stimulant+BT (MPH-SA+ MPH-SA+child, parent and teacher training); BT (child, parent and teacher training) | Global assessment Parents/teachers:  16/28; 9/45 | 7/28; 23/45 | - | - |
| Kaplan et al., 2004^54^ | Non-stimulant (ATX); Placebo | ADHD-RS Parents: 35/53; 16/45 | 12/53; 12/45 | 3/53; 2/45 | - |
| Kelsey et al., 2004^55,56^ | Non-stimulant (ATX); Placebo | ADHD-RS Clinician: 79/133; 20/64  CGI-I Clinician: 34/133; 3/64 | 26/133; 17/64 | 6/133; 1/64 | - |
| Michelson et al., 2004^57,58^ | Non-stimulant (ATX); Placebo | - | 132/292; 79/124 | 9/292; 1/124 | 5/292; 2/124 |
| Wigal et al., 2004^59^ | Stimulant (MPH-SA: low/medium dose; high dose); Placebo | CGI-I Clinician: 29/44; 23/46; 9/42 | 2/44; 6/46; 6/42 | 0/44; 2/46; 2/42 | - |
| Allen et al., 2005^60,61^ | Non-stimulant (ATX); Placebo | - | 50/76; 53/72 | 2/76; 1/72 | 1/76; 0/72 |
| Biederman et al., 2005^62^ | Other unlicensed drug (MODAF); Placebo | CGI-I Clinician: 78/164; 14/84 | 67/164; 51/84 | 5/164; 3/84 | 2/164; 0/84 |
| Jacobs et al., 2005^63^ | Homeopathy (HOMEO); Placebo | - | 2/22; 4/21 | - | - |
| Kemner et al., 2005^64^ | Stimulant (MPH-LA); Non-stimulant (ATX) | ADHD-RS Clinician: 646/850; 299/473  CGI-I Clinician: 583/850; 250/473 | - | 41/850; 26/473 | 7/850; 1/473 |
| Klingberg et al., 2005^65^ | Cognitive training (WM training); Control | - | 7/27; 2/26 | - | - |
| So, 2005^66,67^ | Stimulant (MPH-SA); Stimulant+BT (MPH-SA+child and parent training) | SWAN Parents/teachers: 2/41; 16/45 | 10/41; 3/45 | - | - |
| Starr et al., 2005^68^ | Stimulant (MPH-LA); Non-stimulant (ATX) | ADHD-RS Clinician: 97/125; 35/58  CGI-I Clinician: 86/125; 29/58 | - | 1/125; 1/58 | 0/125; 1/58 |
| Weiss et al., 2005^69-71^ | Non-stimulant (ATX); Placebo | ADHD-RS Teachers: 69/101; 22/52  CGI-S Clinician: 21/101; 5/52 | 17/101; 4/52 | 6/101; 0/52 | - |
| Wigal et al., 2005^72^ | Stimulant (MIX-AMPH-LA); Placebo | CGI-I Clinician: 75/107; 41/108  SKAMP Teachers: 76/107; 36/108 | 14/107; 11/108 | 7/107; 4/108 | 1/107; 1/108 |
| Bierderman et al., 2006^73^ | Other unlicensed drug (MODAF); Placebo | CGI-I Clinician: 60/197; 9/51 | 22/197; 3/51 | 9/197; 0/51 | 1/197; 0/51 |
| Findling et al., 2006^74^ | Stimulant (MPH-SA); Stimulant (MPH-INT); Placebo | CGI-I Clinician: 38/133; 33/139; 5/46  Global assessment Parents:48/133; 41/139; 4/46 | - | 4/133; 3/139; 14/46 | 0/133; 1/139; 0/46 |
| Gau et al., 2006^75^ | Stimulant (MPH-SA); Stimulant (MPH-LA) | CGI-I Clinician: 18/32; 27/32 | - | - | - |
| Greenhill et al., 2006^76^ | Stimulant (MPH-LA); Placebo | CGI-I Clinician: 35/53; 6/50  CGI-S Clinician: 33/53; 5/50 | 5/53; 13/50 | 0/53; 1/50 | - |
| Greenhill et al., 2006b^77^ | Other unlicensed drug (MODAF); Placebo | CGI-I Clinician: 67/133; 12/67 | 33/133; 26/67 | 6/133; 4/67 | - |
| Greenhill et al., 2006c^78-80^ | Stimulant (MPH-SA); Placebo | SNAP Parents/teachers: 13/61; 7/53 | 9/61; 24/53 | 1/61; 0/53 | - |
| Sangal et al., 2006^81^ | Stimulant (MPH-SA); Non-stimulant (ATX) | - | 4/41; 2/44 | 1/41; 0/44 | - |
| Spencer et al., 2006^82^ | Stimulant (MIX-AMPH-LA); Placebo | CGI-I Clinician: 143/233; 14/54 | 53/233; 8/54 | 5/233; 0/54 | - |
| Spencer et al., 2006b^83^ | Stimulant (MIX-AMPH-LA); Placebo | CGI-I Clinician: 105/195; 13/49 | - | 9/195; 0/49 | - |
| Steele et al., 2006^84^ | Stimulant (MPH-SA); Stimulant (MPH-LA) | CGI-I Clinician:45/74; 57/73  CGI-S Clinician: 36/74; 53/73  SNAP Parents/teachers: 25/74; 34/73 | 12/74; 14/73 | 2/74; 6/73 | - |
| Trebatická et al., 2006^85,86^ | Herbal therapy (pine bark extract); Placebo | - | 3/44; 1/17 | - | - |
| Armenteros et al., 2007^87^ | Stimulant+antipsychotic (MPH-SA or MIX-AMPH+RISP); Stimulant (MPH-SA or MIX-AMPH) | CGI-I Clinician: 9/12; 5/13 | 1/12; 1/13 | - | - |
| Arnold et al., 2007^88^ | Aminoacids (l-carnitine); Placebo | CGI-I Clinician (8w): 7/58; 4/60  CGI-I Clinician (16w): 9/58; 8/60 | 16/58; 21/60 | - | - |
| Bangs et al., 2007^89,90^ | Non-stimulant (ATX); Placebo | CGI-I Clinician: 33/72; 12/70  CGI-S Clinician: 13/72; 7/70 | 13/72; 9/70 | 1/72; 1/70 | 0/72; 1/70 |
| Biederman et al., 2007^91,92^ | Stimulant (LDX-LA); Placebo | ADHD-RS Clinician: 175/218; 20/72  CGI-I Clinician: 154/218; 13/72 | 42/218; 18/72 | 20/218; 1/72 | - |
| Buitelaar et al., 2007^93,58^ | Non-stimulant (ATX); Placebo | - | 16/81; 28/82 | 1/81; 1/82 | - |
| Carlson et al., 2007^94^ | Stimulant+non-stimulant (MPH-LA+ATX); Non-stimulant (ATX) | - | 1/9; 1/12 | 1/9; 1/12 | - |
| Gau et al., 2007^95,96^ | Non-stimulant (ATX); Placebo | ADHD-RS Parents: 53/72; 13/34 | 3/72; 5/34 | 1/72; 0/34 | - |
| Geller et al., 2007^97,98^ | Non-stimulant (ATX); Placebo | ADHD-RS Parents: 48/82; 10/89 | 21/82; 23/89 | 2/82; 1/89 | 1/82; 0/89 |
| Prasad et al., 2007^99,100^ | Non-stimulant (ATX); Control | ADHD-RS Clinician: 59/104; 43/97  CGI-I Clinician: 48/104; 25/97  CGI-S Clinician: 49/104; 28/97 | 26/104; 6/97 | 5/104; 1/97 | - |
| van den Hoofdakker et al., 2007^101^ | BT (parent training); Control | - | 7/48; 1/48 | - | - |
| van der Oord et al., 2007^102^ | Stimulant (MPH-SA); Stimulant+BT (MPH-SA+child, parent and teacher training) | - | 2/23; 3/27 | - | - |
| Wang et al., 2007^103,104^ | Stimulant (MPH-SA); Non-stimulant (ATX) | ADHD-RS Parents: 133/166; 123/164 | 14/166; 26/164 | 6/166; 18/164 | 0/166; 1/164 |
| Amiri et al., 2008^105^ | Stimulant (MPH-SA); Other unlicensed drug (MODAF) | ADHD-RS Parents: 21/30; 22/30  ADHD-RS Teachers: 22/30; 22/30 | 3/30; 22/30 | - | - |
| Bangs et al., 2008^106,107^ | Non-stimulant (ATX); Placebo | - | 24/156; 5/70 | 6/156; 0/70 | 3/156; 1/70 |
| Bierdeman et al., 2008^108-110^ | Non-stimulant α-2 agonist (GUAN-LA); Placebo | CGI-I Clinician: 133/259; 20/86  Global assessment Parents: 111/259; 15/86 | 97/259; 33/86 | 42/259; 1/86 | 2/259; 0/86 |
| Palumbo et al., 2008^111-113^ | Stimulant (MPH-SA); Non-stimulant α-2 agonist (CLON-SA); Stimulant (MPH-SA) + non-stimulant α-2 agonist (CLON-SA); Placebo | - | 11/29; 5/31; 8/32; 20/30 | 1/29; 1/31; 1/32; 0/30 | 1/29; 0/31; 0/32; 0/30 |
| Findling et al., 2008^114^ | Stimulant (MPH-LA); Stimulant (MPH-TS); Placebo | CGI-I Clinician: 59/94; 69/100; 20/88  Global assessment Parents: 54/94; 67/100; 21/88 | 25/94; 67/100; 53/88 | 2/94; 7/100; 1/88 | - |
| Heriot et al., 2008^115^ | Stimulant (MPH-SA); BT (parent training); Stimulant+BT (MPH-SA+parent training); Placebo | ADHD-RS Parent: 1/4; 1/4; 2/4; 0/4  ADHD-RS Teacher: 1/4; 1/4; 3/4; 0/4 | - | - | - |
| Konofal et al., 2008^116^ | Minerals (Iron); Placebo | CGI-S Clinician: 4/18; 0/5 | 2/18; 0/5 | 1/18; 0/5 | - |
| Newcorn et al., 2008^117,118^ | Stimulant (MPH-LA); Non-stimulant (ATX); Placebo | ADHD-RS Parents: 119/220; 95/222; 16/74 | 40/220; 36/222; 17/74 | 5/220; 5/222; 2/74 | 1/220; 4/222; 0/74 |
| Torrioli et al., 2008^119^ | Aminoacids (L-carnitine); Placebo | - | 3/30; 4/33 | - | - |
| Vaisman et al., 2008^120^ | PUFA (omega-3 fatty acid); Control (fish oil); Placebo | - | 11/29; 7/28; 5/26 | 2/29; 2/28; 0/26 | - |
| Weber et al., 2008^121^ | Herbal therapy (St John’s Wort); Placebo | CGI-I Clinician: 11/27; 11/27 | 1/27; 2/27 | 0/27; 1/27 | - |
| Arabgol et al., 2009^122^ | Stimulant (MPH-SA); Antidepressant (REBOX) | ADHD-RS Parents: 6/16; 5/17 | 4/16; 5/17 | 1/16; 2/17 | - |
| Block et al., 2009^123,124^ | Non-stimulant (ATX); Placebo | ADHD-RS Parents: 115/195; 33/93  CGI-S Clinician: 80/195; 20/93 | 55/195; 27/93 | 3/195; 2/93 | - |
| Childress et al., 2009^125^ | Stimulant (MPH-LA); Placebo | CGI-I Clinician: 131/188; 14/65  CGI-S Clinician: 106/188; 17/65 | 27/188; 8/65 | 3/188; 0/65 | - |
| Dell'Agnello et al., 2009^126^ | Non-stimulant (ATX); Placebo | SNAP Parents/teachers: 33/107; 2/32  CGI-S Clinician: 20/107; 2/32 | 5/107; 0/32 | 3/107; 0/32 | - |
| Johnson et al., 2009^127^ | PUFA (omega-3/6 fatty acid); Placebo | ADHD-RS Clinician: 9/37; 2/38 | 3/37; 8/38 | 2/37; 1/38 | - |
| Kahbazi et al., 2009^128^ | Other unlicensed drug (MODAF); Placebo | ADHD-RS Parents/teachers: 18/23; 0/23 | 1/23; 2/23 | - | - |
| Montoya et al., 2009^129,130^ | Non-stimulant (ATX); Placebo | ADHD-RS Parents: 63/100; 11/51  CGI-S Clinician: 40/100; 5/51 | 6/100; 3/51 | - | 0/100; 1/51 |
| Nair et al., 2009^131^ | Non-stimulant α-2 agonist (CLON-SA); Other unlicensed drug (CARBA) | ADHD-RS Clinician: 18/25; 3/25 | 4/25; 6/25 | - | - |
| Pelsser et al., 2009^132^ | Restricted elimination diet (elimination diet); Waiting list | ADHD-RS Parents: 11/15; 0/12  Conners’ Parents: 11/15; 0/12  Conners’ Teachers: 7/15; 0/12 | 2/15; 1/12 | 1/15; 0/12 | - |
| Raz et al., 2009^133^ | PUFA (omega-3/6 fatty acid); Placebo | - | 7/39; 8/39 | 1/39; 0/39 | - |
| Sallee et al., 2009^134-136^ | Non-stimulant α-2 agonist (GUAN-LA); Placebo | CGI-I Clinician: 126/258; 19/66  Global assessment Parents: 106/258; 16/66 | 88/258; 25/66 | 19/258; 5/66 | 1/258; 1/66 |
| Svanborg et al., 2009^137,138^ | Non-stimulant+BT (ATX+child and parent training); BT (child and parent training) + placebo | ADHD-RS Clinician: 35/49; 14/50 | - | - | - |
| Takahashi et al., 2009^139,140^ | Non-stimulant (ATX); Placebo | ADHD-RS Clinician: 114/183; 34/62 | 10/183; 1/62 | 2/183; 0/62 | 1/183; 0/62 |
| Perez-Alvarez et al., 2009^141^ | Stimulant (MPH-LA); HPT (humanistic psychotherapy); Stimulant+HPT (MPH-LA+humanistic psychotherapy) | SNAP Parents/teachers: 10/32; 20/59; 31/59 | - | - | - |
| Thompson et al., 2009^142^ | BT (parent training); Control | PACS Parents: 8/21; 1/20 | 6/21; 15/20 | - | - |
| Tramontina et al., 2009^143^ | Antipsychotic (ARIP); Placebo | - | 1/18; 1/25 | 1/18; 0/25 | - |
| Tucker et al., 2009^144^ | Stimulant+BT (MPH-LA+child and parent training); BT (child and parent training) | - | 15/53; 17/56 | - | - |
| Gevensleben et al., 2009^145-147^ | Neurofeedback (theta-beta and slow cortical potential training); Cognitive training (attention training) | ADHD-RS Parents (8w): 30/64; 10/38  ADHD-RS Parents (24w): 19/64; 6/38 | 11/64; 6/38 | - | - |
| Connor et al., 2010^148,149^ | Non-stimulant α-2 agonist (GUAN-LA); Placebo | CGI-I Clinician: 93/138; 24/79  CGI-S Clinician: 87/138; 24/79 | 29/138; 31/79 | 14/138; 1/79 | - |
| Fabiano et al., 2010^150^ | BT (child, parent and teacher training); Control | ADHD-RS Teachers: 28/33; 20/30 | 0/33; 3/30 | - | - |
| Findling et al., 2010^151^ | Stimulant (MPH-TS); Placebo | CGI-I Clinician: 93/145; 22/72  Global assessment Parents: 76/145; 15/72 | 53/145; 43/72 | 8/145; 2/72 | 1/145; 1/72 |
| Gustafsson et al., 2010^152^ | PUFA (omega-3 fatty acid); Placebo | Conners’ Parents: 15/57; 17/52  Conners’ Teachers: 14/57; 11/52 | 17/57; 10/52 | - | - |
| Martenyi et al., 2010^153,154^ | Non-stimulant (ATX); Placebo | ADHD-RS Parents: 52/72; 16/33 | 5/72; 1/33 | 1/72; 0/33 | - |
| Perreau-Linck et al., 2010^155^ | Neurofeedback (theta-beta training); Placebo | - | 1/5; 1/4 | 0/5; 1/4 | - |
| Salehi et al., 2010^156^ | Stimulant (MPH-SA); Herbal therapy (*Ginkgo biloba*) | ADHD-RS Parents: 22/25; 8/25  ADHD-RS Teachers: 16/25; 2/25 | 2/25; 2/25 | - | - |
| Thurstone et al., 2010^157,158^ | Non-stimulant+BT (ATX+child and parent training); BT (child and parent training) + placebo | CGI-I Clinician: 17/35; 20/35 | 3/35; 2/35 | 1/35; 0/35 | 1/35; 1/35 |
| Waxmonsky et al., 2010^159^ | Non-stimulant+BT (ATX+child, parent and teacher training); Non-stimulant (ATX) | CGI-I Clinician: 16/29; 14/27 | 5/29; 2/27 | 0/59; 1/27 | - |
| Zarinara et al., 2010^160^ | Stimulant (MPH-SA); Antidepressant (VENLAF) | ADHD-RS Parents: 14/19; 13/19  ADHD-RS Teachers: 13/19; 12/19 | 1/19; 1/19 | - | - |
| Abbasi et al., 2011^161^ | Stimulant (MPH-SA); Stimulant + aminoacids (MPH-SA+L-carnitine) | - | 1/20; 1/20 | - | - |
| Arnold et al., 2011^162^ | Minerals (Zinc supplementation); Placebo | - | 2/28; 2/24 | 1/28; 1/24 | - |
| Bakhshayesh et al., 2011^163^ | Neurofeedback (theta-beta training); Control | - | 1/18; 2/17 | - | - |
| Dittmann et al., 2011^164^ | Non-stimulant (ATX); Placebo | - | 29/121; 22/59 | 8/121; 1/59 | 2/121; 1/59 |
| Findling et al., 2011^165,166^ | Stimulant (LDX-LA); Placebo | CGI-Clinician: 154/235; 30/79  ADHD-RS Clinician: 182/235; 43/79 | 39/235; 10/79 | 10/235; 1/79 | - |
| Jain et al., 2011^167^ | Non-stimulant α-2 agonist (CLON-LA); Placebo | - | 56/158; 37/78 | 20/158; 1/78 | - |
| Giblin et al., 2011^168^ | Stimulant (LDX-LA); Placebo | - | - | - | - |
| Kang et al., 2011^169^ | Stimulant+BT (MPH-SA+child training); Stimulant+Exercise (MPH-SA+physical activity/sports) | - | 3/16; 1/16 | 1/16; 1/16 | - |
| Kollins et al., 2011^170^ | Stimulant+non-stimulant α-2 agonist (MPH-LA or LDX-LA+CLON-LA); Stimulant (MPH-LA or LDX) | CGI-Clinician/ADHD-RS Clinician: 43/102; 24/96 | 11/102; 22/96 | 1/102; 3/96 | - |
| Kollins et al., 2011^171^ | Non-stimulant α-2 agonist (GUAN-LA); Placebo | CGI-Clinician: 69/121; 20/57 | 7/121; 3/57 | 4/121; 1/57 | 2/121; 0/57 |
| Kratochvil et al., 2011^172,173^ | Non-stimulant+BT (ATX+parent training); BT (parent training) | CGI-I Clinician: 18/50; 11/51  CGI-S Clinician: 17/50; 11/51 | 14/50; 12/51 | 0/50; 3/51 | - |
| Lansbergen et al., 2011^174^ | Neurofeedback (IFBT); Placebo | CGI-I Clinician: 1/8; 0/6 | - | - | - |
| Pelsser et al., 2011^175^ | Restricted elimination diet (elimination diet); Control | ADHD-RS Clinician: 32/50; 0/50 | 9/50; 8/50 | 1/50; 0/50 | - |
| Riggs et al., 2011^176^ | Stimulant+BT (MPH-LA+child training); BT (child training) + placebo | CGI-I Clinician: 35/151; 29/152 | 33/151; 43/152 | - | 4/151; 7/152 |
| Steiner et al., 2011^177^ | Neurofeedback (theta-beta training); Control; Waiting list | - | 3/13; 2/13; 0/15 | - | - |
| Wehmeier et al., 2011^178,179^ | Non-stimulant (ATX); Placebo | - | 9/63; 11/62 | 2/63; 2/62 | - |
| Wilens et al., 2011^180^ | Non-stimulant (ATX); Placebo | - | 7/50; 5/47 | 2/50; 1/47 | - |
| Yildiz et al., 2011^181^ | Stimulant (MPH-LA); Non-stimulant (ATX) | CGI-I Clinician: 10/12; 7/17  ADHD-RS Parents: 7/12; 4/17 | 1/12; 3/17 | 1/12; 3/17 | - |
| Zamora et al., 2011^182^ | Stimulant (MPH-SA); Stimulant+minerals (MPH-SA+Zinc sulfate) | Conners’ Teachers: 6/20; 11/20 | 3/20; 1/20 | - | - |
| Assareh et al., 2012^183^ | Stimulant+PUFA (MPH-SA+omega-3/6 fatty acid); Stimulant (MPH-SA)+Placebo | ADHD-RS Parents: 19/20; 19/20 | - | - | - |
| Duric et al., 2012^184^ | Stimulant (MPH-SA); Neurofeedback (theta-beta training); Stimulant+neurofeedback (MPH-SA+theta-beta training) | - | 15/44; 13/42; 11/44 | - | - |
| Fabiano et al., 2012^185^ | BT (parent training); Waiting list | ECBI Parents: 17/28; 13/27 | 5/28; 4/27 | - | - |
| Green et al., 2012^186^ | Cognitive training (WM training); Placebo | - | 3/15; 1/15 | - | - |
| Jafarinia et al., 2012^187^ | Stimulant (MPH-SA); Antidepressant (BUP) | ADHD-RS Parents: 18/22; 18/22  ADHD-RS Teachers: 12/22; 8/22 | 1/22; 2/22 | - | - |
| Manor et al., 2012^188,189^ | PUFA (omega-3 fatty acid); Placebo | - | 27/137; 11/63 | 2/137; 1/63 | - |
| Perera et al., 2012^190^ | Stimulant+PUFA (MPH-SA+omega-3/6 fatty acid); Stimulant (MPH-SA)+Placebo | - | 1/49; 3/49 | - | - |
| Wilens et al., 2012^191-194^ | Stimulant+non-stimulant α-2 agonist (MPH-LA or LDX-LA+GUAN-LA); Stimulant (MPH-LA or LDX) | CGI-I Clinician: 215/307; 88/154  CGI-S Clinician: 229/307; 94/154  ADHD-RS Clinician: 212/307; 89/154  Global assessment Parents: 180/307; 67/154 | 58/307; 25/154 | 10/307; 1/154 | 3/307; 0/154 |
| Abikoff et al., 2013^195^ | BT (child, parent and teacher training), Waiting list | CGI-I Parents: 108/125; 0/33  CGI-I Teachers: 77/125; 1/33 | 7/125; 0/33 | 1/125; 0/33 | - |
| Arnold et al., 2013^196^ | Neurofeedback (theta-beta training); Placebo | - | 2/26; 3/13 | - | - |
| Coghill et al., 2013^197-200^ | Stimulant (LDX-LA); Stimulant (MPH-LA); Placebo | CGI-I Clinician: 78/113; 63/112; 15/111  ADHD-RS Clinician: 89/113; 76/112; 30/111 | 33/113; 38/112; 68/111 | 5/113; 2/112; 4/111 | 3/113; 2/112; 3/111 |
| Dittmann et al., 2013^201-204^ | Stimulant (LDX-LA); Non-stimulant (ATX) | CGI-I Clinician: 69/133; 53/134  CGI-S Clinician: 71/133; 57/134  ADHD-RS Clinician: 88/133; 68/134 | 34/133; 33/134 | 8/133; 10/134 | - |
| Hovik et al., 2013^205,206^ | Cognitive training (WM training); Control | - | 5/38; 3/37 | - | - |
| Li et al., 2013^207^ | Stimulant+neurofeedback (MPH-SA+theta-beta training); Stimulant (MPH-SA) | - | 1/32; 3/32 | - | - |
| Newcorn et al., 2013^208-211^ | Non-stimulant α-2 agonist (GUAN-LA); Placebo | CGI-I Clinician/ADHD-RS Clinician: 132/227; 34/113  CGI-I Clinician: 144/227; 35/113  CGI-S Clinician: 139/227; 31/113 | 60/227; 37/113 | 16/227; 1/113 | 3/227; 0/113 |
| Ghanizadeh et al., 2013^212^ | Stimulant+vitamins (MPH-SA+vitamin B9/folic acid); Stimulant (MPH-SA) | - | 15/23; 12/26 | 4/23; 3/26 | - |
| Oberai et al., 2013^213^ | Homeopathy (HOMEO); Placebo | - | 8/30; 11/31 | - | - |
| Ogrim et al., 2013^213^ | Stimulant (MPH-LA or DEXAM); Neurofeedback (theta-beta training) | Conners’ Parents/Teachers: 9/16; 2/16 | 1/16; 2/16 | - | - |
| Simonoff et al., 2013^214^ | Stimulant (MPH-SA); Placebo | CGI-I Clinician: 24/61; 4/61 | 10/61; 7/61 | 5/61; 0/61 | - |
| Tamm et al., 2013^215^ | Cognitive training (attention training); Waiting list | - | 9/54; 5/51 | - | - |
| van Dongen-Boomsma et al., 2013^217^ | Neurofeedback (theta-beta training); Placebo | CGI-I Clinician: 4/22; 0/22 | - | - | - |
| Aman et al., 2014^218-220^ | Stimulant+antipsychotic+BT (MPH-LA or MIX-AMPH+RISP+parent training); Stimulant+BT (MPH-LA or MIX-AMPH+parent training) | CGI-I Clinician: 63/84; 58/84  CGI-S Clinician: 56/84; 49/84 | 23/84; 15/84 | - | - |
| Barragán et al., 2014^221^ | Stimulant (MPH-SA)+PUFA (omega-3/6 fatty acid); Stimulant (MPH-SA); PUFA (omega-3/6 fatty acid) | ADHD-RS Parents: 28/30; 24/30; 18/30 | 3/30; 10/30, 8/30 | 1/30; 6/30; 1/30 | - |
| Chacko et al., 2014^222^ | Cognitive training (WM training); Placebo | - | 7/44; 6/41 | - | - |
| Ferrin et al., 2014^223^ | BT (parent training); Control (parent counseling with support) | - | 2/44; 1/37 | - | - |
| Garg et al., 2014^224^ | Stimulant (MPH-SA); Non-stimulant (ATX) | ADHD-RS Parents: 30/41; 31/43 | 14/41; 18/43 | 3/41; 3/43 | - |
| Hervas et al., 2014^225-227^ | Non-stimulant (ATX); Non-stimulant α-2 agonist (GUAN-LA); Placebo | CGI-I Clinician: 63/112; 76/115; 49/111  CGI-S Clinician: 65/112; 77/115; 51/111  ADHD-RS/CGI Clinician: 62/112; 72/112; 47/111 | 23/112; 24/115; 19/111 | 5/112; 9/115; 1/111 | 0/112; 1/115; 1/111 |
| Hirayama et al., 2014^228^ | Aminoacids (phosphatidyl-serine); Placebo | - | 1/20; 3/20 | - | - |
| Ko et al., 2014^229^ | Herbal therapy (*Ginseng*); Placebo | - | 0/35; 2/37 | - | - |
| Lin et al., 2014^230,231^ | Stimulant (MPH-LA); Non-stimulant (EDIVOX); Placebo | ADHD-RS Parents: 14/36; 81/226; 22/78  CGI-I Clinician: 13/36; 59/226; 20/78 | 7/36; 53/226; 16/78 | 3/36; 7/226; 3/78 | - |
| Meisel et al., 2014^232^ | Stimulant (MPH-SA); Neurofeedback (theta-beta training) | - | 2/13; 2/14 | 1/13; 0/14 | - |
| Pfiffner et al., 2014^233^ | BT (child, parent and teacher training); BT (parent training); Control (usual care) | CSI Parents: 41/74; 32/74; 15/51  CSI Teachers: 43/74; 33/74; 17/51 | 1/74; 0/74; 3/51 | - | - |
| Steiner et al., 2014^234,235^ | Cognitive training (attention training); Neurofeedback (theta-beta training); Control | - | 4/34; 0/34; 2/36 | - | - |
| van Dongen-Boomsma et al., 2014^236^ | Cognitive training (WM training); Placebo | CGI-I Clinician: 1/27; 2/24 | 1/27; 3/24 | - | - |
| Widenhorn-Müller et al., 2014^237^ | PUFA (omega-3 fatty acid); Placebo | - | 7/55; 6/55 | - | - |
| Abikoff et al., 2015^238^ | BT (parent training); Waiting list | - | 12/130; 1/34 | - | - |
| Bigorra et al., 2015^239^ | Cognitive training (WM training); Placebo | - | 4/36; 0/30 | - | - |
| Bédard et al., 2015^240^ | Non-stimulant α-2 agonist (GUAN-LA); Placebo | CGI-I Clinician: 8/12; 5/13 | - | - | - |
| Bos et al., 2015^241^ | PUFA (omega-3 fatty acid); Placebo | - | 1/20; 1/20 | 0/20; 1/20 | - |
| Choi et al., 2015^242^ | Stimulant+BT (MPH-SA+child training); Stimulant+Exercise (MPH-SA+physical activity/sports) | - | 1/18; 4/17 | 1/18; 1/17 | - |
| Choi et al., 2015^243^ | BT (child training); Waiting list | - | 5/53; 2/27 | - | - |
| Chou et al., 2015^244^ | Stimulant (MPH-LA); Non-stimulant (ATX) | - | 5/25; 3/25 | - | - |
| Corkum et al., 2015^245^ | BT (teacher training); Waiting list | - | 4/28; 2/30 | - | - |
| Ghanizadeh et al., 2015^246^ | Stimulant+restricted elimination diet (MPH-SA+elimination diet); Stimulant (MPH-SA) | - | 10/53; 11/53 | - | - |
| Hiscock et al., 2015^247^ | BT (parent training); Control (usual care) | ADHD-RS Parents (12w): 23/122; 12/122  ADHD-RS Parents (24w): 33/122; 12/122  ADHD-RS Teachers (12w): 14/122; 11/122  ADHD-RS Teachers (24w): 34/122; 30/122 | 16/122; 24/122 | - | - |
| Matsudaira et al., 2015^248^ | PUFA (omega-3/6 fatty acid); Placebo | - | 11/38; 8/38 | 3/38; 0/38 | - |
| Shakibaei et al., 2015^249^ | Stimulant+herbal therapy (MPH-SA+*Ginkgo biloba*); Stimulant (MPH-SA) | ADHD-RS Parents: 29/33; 17/33  ADHD-RS Teachers: 26/33; 23/33 | 2/33; 4/33 | 2/33; 1/33 | - |
| Shang et al., 2015^250^ | Stimulant (MPH-LA); Non-stimulant (ATX) | - | 14/80; 16/80 | 3/80; 3/80 | - |
| Storebø et al., 2015^251,252^ | BT (child and parent training); Control (usual care) | - | 0/28; 1/28 | - | - |
| Wilens et al., 2015^253-255^ | Non-stimulant α-2 agonist (GUAN-LA); Placebo | CGI-I Clinician: 104/157; 71/157  ADHD-RS Clinician: 103/157; 71/157  CGI-S Clinician: 78/157; 56/157 | 52/157; 55/157 | 9/157; 3/157 | 4/157; 2/157 |
| Arabgol et al., 2015^256^ | Antipsychotics (RISP); Stimulant (MPH-SA) | - | 2/20; 3/18 | 2/20; 1/18 | - |
| Correia-Filho et al., 2005^257^ | Antipsychotics (RISP); Stimulant (MPH-SA) | - | 3/22; 2/24 | 1/22; 1/24 | - |
| Ferrin et al., 2016^258^ | BT (parent training); Control (usual care) | - | 3/35; 4/34 | - | - |
| Janssen et al., 2016^259,260^ | Stimulant (MPH-SA); Neurofeedback (theta-beta training); Exercise (physical activity/sports) | - | 5/36; 1/39; 3/37 | - | - |
| Steeger et al., 2016^261^ | BT (parent training); Cognitive training (WM training); BT+cognitive training (parent training+WM training); Placebo | - | 1/26; 3/26; 4/26; 0/26 | - | - |
| Su et al., 2016^262^ | Stimulant (MPH-LA); Non-stimulant (ATX) | ADHD-RS Clinician: 78/130; 77/132  CGI-S Clinician: 50/130; 49/132 | 45/130; 75/132 | - | - |
| Newcorn et al., 2016^263,264^ | Non-stimulant α-2 agonist (GUAN-LA); Placebo | CGI-S: 75/157; 49/159 | 81/157; 106/159 | 3/157; 2/159 | 2/157; 4/159 |
