# Supplementary material for: The pharmacological and non-pharmacological treatment of attention deficit hyperactivity disorder in children and adolescents: A systematic review with network meta-analyses of randomised trials
Source: PLoS One. 2017 Jul 12;12(7):e0180355. doi: 10.1371/journal.pone.0180355 (PMC5507500; doi:10.1371/journal.pone.0180355)
Supplement: S5 Table — (DOCX) [file pone.0180355.s010.docx]

**S5 Table. Number of events per trial and treatment comparison for serious adverse events and specific adverse events.**

| **Trial name, year** | **Treatment comparisons** | **Anorexia**  **Decreased weight** | **Insomnia**  **Sleep disturbances** | **Anxiety** | **Syncope** | **Cardiovascular events** |
| --- | --- | --- | --- | --- | --- | --- |
| Gittelman-Klein et al., 1976^1^ | Stimulant (MPH-SA); Antipsychotic (THIO); Stimulant+antipsychotic (MPH-SA+THIO); Placebo | 22/41; 2/41; 7/42; 1/42  - | -  26/41; 2/41; 6/42; 7/42 | - | - | - |
| Firestone et al., 1986^2^ | Stimulant (MPH-SA); BT (parent training); Stimulant+BT (MPH-SA+parent training) | - | - | - | - | - |
| Casat et al., 1987^3,4^ | Antidepressant (BUP); Placebo | - | - | - | - | - |
| Kupietz et al., 1988^5^ | Stimulant (MPH-SA); Placebo | - | - | - | - | - |
| Biederman et al., 1989^6,7^ | Antidepressant (DESIP); Placebo | 10/37; 6/36  2/37; 0/36 | -  7/37; 2/36 | - | - | - |
| Egger et al., 1992^8^ | Restricted elimination diet (oligoantigenic diet); Placebo | - | - | - | - | - |
| Gunning 1992^9^ | Stimulant (MPH-SA); Non-stimulant α-2 agonist (CLON-SA); Placebo | 3/24; 4/42; 2/43  - | -  5/24; 0/42; 1/43 | - | - | - |
| Pisterman 1992^10^ | BT (parent training); Waiting list | - | - | - | - | - |
| Buitelaar et al., 1996^11^ | Stimulant (MPH-SA); Placebo | 2/10; 3/11  - | 4/11; 3/11  5/11; 4/11 | 2/10; 2/11 | - | - |
| Conners et al., 1996^12^ | Antidepressant (BUP); Placebo | - | - | - | - | - |
| Schachar et al., 1997^13^ | Stimulant+BT (MPH-SA+parent training); BT (parent training) | - | - | - | - | - |
| Klein and Abikoff, 1997^14^ | Stimulant (MPH-SA); BT (parent and teacher training); Stimulant+BT (MPH-SA+parent and teacher training) | - | - | - | - | Tachycardia: 1/30; 0/29; 0/30 |
| Van der Meere et al., 1999^15^ | Stimulant (MPH-SA); Non-stimulant α-2 agonist (CLON-SA); Placebo | - | - | - | - | - |
| MTA Cooperative, 1999^16-19^ | Stimulant (MPH-SA); BT (child, parent and teacher training); Stimulant+BT (MPH-SA+child, parent and teacher training); Standard care | - | - | - | - | - |
| Connor et al., 2000^20^ | Stimulant (MPH-SA); Non-stimulant α-2 agonist (CLON-SA); Stimulant (MPH-SA) + non-stimulant α-2 agonist (CLON-SA) | - | - | - | - | Bradycardia:  0/8; 2/8; 4/8 |
| Pliszka et al., 2000^21^ | Stimulant (MPH-SA); Stimulant (MIX-AMPH); Placebo | 3/20; 3/20; 2/18  - | - | 2/20; 1/20; 1/18 | - | - |
| Prince et al., 2000^22^ | Antidepressant (NT); Placebo | - | - | - | - | - |
| Michelson et al., 2001^23-25^ | Non-stimulant (ATX); Placebo | 23/213; 4/84  - | 13/213; 5/284  - | - | - | - |
| Scahill et al., 2001^26^ | Non-stimulant α-2 agonist (GUAN-SA); Placebo | 2/17; 0/17  - | -  3/17; 0/17 | - | - | Blood pressure decreased: 6/17; 2/17 |
| Sonuga-Barke et al., 2001^27^ | BT (parent training); Control (parent counseling with support); Waiting list | - | - | - | - | - |
| Voigt et al., 2011^28^ | Stimulant (MPH-SA)+PUFA (omega-3 acid); Stimulant (MPH-SA)+Placebo | - | - | - | - | - |
| Wolraich et al., 2001^29^ | Stimulant (MPH-SA); Stimulant (MPH-LA); Placebo | 18/97; 21/95; 11/90  - | - | - | - | - |
| Biederman et al., 2002^31,32^ | Stimulant (MIX-AMPH-LA); Placebo | 82/374; 4/210  - | 62/374; 4/210  - | - | - | Blood pressure increased: 4/374; 5/210 |
| Bor et al., 2002^33^ | BT (parent training); Waiting list | - | - | - | - | - |
| Greenhill et al., 2002^34^ | Stimulant (MPH-INT); Placebo | 15/158; 4/163  - | 11/158; 4/163  - | - | - | - |
| Lehmkuhl et al., 2002^35-37^ | Stimulant (MPH-LA); Placebo | - | - | - | - | - |
| Kratochvil et al., 2002^38,39^ | Stimulant (MPH-SA); Non-stimulant (ATX) | 6/44; 35/184  2/44; 5/184 | 7/44; 17/184  - | - | - | Tachycardia: 2/44; 11/184  Palpitations: 2/44; 3/184 |
| Michelson et al., 2002^40,41^ | Non-stimulant (ATX); Placebo | 17/85; 5/86  - | - | - | - | - |
| Spencer et al., 2002a^42,30^ | Stimulant (MPH-SA); Non-stimulant (ATX); Placebo | -; 28/129; 9/124  -; 5/129; 0/124  (pooled Spencer 2002a and 2000b) | -; 9/129; 11/124  - | - | - | - |
| Spencer et al., 2002b^42,30^ | Non-stimulant (ATX); Placebo | = | = | - | - | - |
| Spencer et al., 2002c^43^ | Antidepressant (DESIP); Placebo | 5/21; 0/20  - | -  4/21; 1/20 | - | - | - |
| TSSG, 2002^44^ | Stimulant (MPH-SA); Non-stimulant α-2 agonist (CLON-SA); Stimulant (MPH-SA) + non-stimulant α-2 agonist (CLON-SA); Placebo | - | - | - | - | - |
| van Oudheusden et al., 2002^45^ | Aminoacids (l-carnitine); Placebo | - | - | - | - | - |
| Hazell et al., 2003^46^ | Stimulant (MPH-SA or DEXAM); Stimulant (MPH-SA or DEXAM) + non-stimulant α-2 agonist (CLON-SA) | - | - | - | - | - |
| Rugino et al., 2003^47^ | Other unlicensed drug (MODAF); Placebo | 0/13; 1/11  - | -  4/13; 4/11 | - | - | - |
| Abikoff et al., 2004^48-50^ | Stimulant (MPH-SA); Stimulant+BT (MPH-SA+child and parent training); Stimulant+control (MPH-SA+attential control psychosocial therapy) | - | - | - | - | - |
| Akhondzadeh et al., 2004^51^ | Stimulant (MPH-SA); Stimulant+minerals (MPH-SA+Zinc sulfate) | 7/22; 8/22  - | -  6/22; 6/22 | 3/22; 3/22 | - | - |
| Bilici et al., 2004^52^ | Minerals (Zinc sulfate); Placebo | - | - | - | - | - |
| Döpfner et al., 2004^53^ | Stimulant+BT (MPH-SA+ MPH-SA+child, parent and teacher training); BT (child, parent and teacher training) | - | - | - | - | - |
| Kaplan et al., 2004^54^ | Non-stimulant (ATX); Placebo | 10/53; 1/45  - | - | - | - | - |
| Kelsey et al., 2004^55,56^ | Non-stimulant (ATX); Placebo | 23/133; 4/64  - | - | - | 3/133; 0/64 | - |
| Michelson et al., 2004^57,58^ | Non-stimulant (ATX); Placebo | - | - | - | - | - |
| Wigal et al., 2004^59^ | Stimulant (MPH-SA: low/medium dose; high dose); Placebo | 4/44; 5/46; 0/42  4/44; 6/46; 2/42 | - | 0/44; 1/46; 1/42 | - | Blood pressure decreased: 2/44; 6/46; 0/42  Increased heart rate: 1/44; 0/46; 0/42  Increased pulse values: 1/44; 2/46; 1/42 |
| Allen et al., 2005^60,61^ | Non-stimulant (ATX); Placebo | 12/76; 2/72  40/76; 9/72 | 2/76; 3/72  - |  |  | Increased heart rate: 10/76; 2/72 |
| Biederman et al., 2005^62^ | Other unlicensed drug (MODAF); Placebo | 26/164; 3/84  - | 48/164; 3/84  - | - | - | Tachycardia: 1/164; 0/84 |
| Jacobs et al., 2005^63^ | Homeopathy (HOMEO); Placebo | - | - | - | - | - |
| Kemner et al., 2005^64^ | Stimulant (MPH-LA); Non-stimulant (ATX) | 49/850; 14/473  - | 53/850; 11/473  - | - | - | - |
| Klingberg et al., 2005^65^ | Cognitive training (WM training); Control | - | - | - | - | - |
| So, 2005^66,67^ | Stimulant (MPH-SA); Stimulant+BT (MPH-SA+child and parent training) | - | - | - | - | - |
| Starr et al., 2005^68^ | Stimulant (MPH-LA); Non-stimulant (ATX) | 5/125; 1/58  - | 4/125; 0/58  - | - | - | - |
| Weiss et al., 2005^69-71^ | Non-stimulant (ATX); Placebo | 24/101; 2/52  35/101; 1/52 | 2/101; 0/52 | 1/101; 0/52 | - | Palpitations: 1/101; 0/52 |
| Wigal et al., 2005^72^ | Stimulant (MIX-AMPH-LA); Placebo | 30/107; 19/108  6/107; 4/108 | 30/107; 8/108  - | - | - | - |
| Bierderman et al., 2006^73^ | Other unlicensed drug (MODAF); Placebo | 14/197; 1/51  - | 23/197; 1/51  - | - | - | - |
| Findling et al., 2006^74^ | Stimulant (MPH-SA); Stimulant (MPH-INT); Placebo | 4/133; 9/139; 0/46  - | 5/133; 6/139; 0/46 | - | - | - |
| Gau et al., 2006^75^ | Stimulant (MPH-SA); Stimulant (MPH-LA) | 19/32; 15/32  - | 15/32; 13/32 | 10/32; 6/32 | - | - |
| Greenhill et al., 2006^76^ | Stimulant (MPH-LA); Placebo | 17/53; 5/50  6/53; 0/50 | 7/53; 5/50 | - | - | Blood pressure increased: 2/53; 2/50  Decreased heart rate: 1/53; 6/50 |
| Greenhill et al., 2006b^77^ | Other unlicensed drug (MODAF); Placebo | 23/133; 2/67  7/133; 0/67 | 37/133; 5/67  - | 0/133; 1/67 | - | Tachycardia: 0/133; 1/67 |
| Greenhill et al., 2006c^78-80^ | Stimulant (MPH-SA); Placebo | - | - | - | - | - |
| Sangal et al., 2006^81^ | Stimulant (MPH-SA); Non-stimulant (ATX) | - | - | - | - | - |
| Spencer et al., 2006^82^ | Stimulant (MIX-AMPH-LA); Placebo | 83/233; 1/54  10/233; 0/54 | 41/233; 2/54  - | - | - | - |
| Spencer et al., 2006b^83^ | Stimulant (MIX-AMPH-LA); Placebo | 59/195; 2/49  15/195; 0/49 | 22/195; 4/49  - | - | - | - |
| Steele et al., 2006^84^ | Stimulant (MPH-SA); Stimulant (MPH-LA) | 23/74; 17/73  - | 10/74; 12/73  7/74; 3/73 | - | - | - |
| Trebatická et al., 2006^85,86^ | Herbal therapy (pine bark extract); Placebo | - | - | - | - | - |
| Armenteros et al., 2007^87^ | Stimulant+antipsychotic (MPH-SA or MIX-AMPH+RISP); Stimulant (MPH-SA or MIX-AMPH) | - | - | - | - | - |
| Arnold et al., 2007^88^ | Aminoacids (l-carnitine); Placebo | - | - | - | - | - |
| Bangs et al., 2007^89,90^ | Non-stimulant (ATX); Placebo | 9/72; 0/70  20/72; 2/70 | - | - | - | Blood pressure increased: 4/72; 4/72  Decreased pulse values: 1/72; 1/70 |
| Biederman et al., 2007^91,92^ | Stimulant (LDX-LA); Placebo | 85/218; 3/72  20/218; 1/72 | 41/218; 2/72  - | - | - | - |
| Buitelaar et al., 2007^93,58^ | Non-stimulant (ATX); Placebo | - | - | - | - | - |
| Carlson et al., 2007^94^ | Stimulant+non-stimulant (MPH-LA+ATX); Non-stimulant (ATX) | - | 1/9; 3/12 | - | - | Blood pressure increased: 1/9; 0/12  Supraventricular extrasystole: 1/9; 0/12 |
| Gau et al., 2007^95,96^ | Non-stimulant (ATX); Placebo | 26/72; 5/34  4/72; 0/34 | 8/72; 1/34  - | - | - | - |
| Geller et al., 2007^97,98^ | Non-stimulant (ATX); Placebo | 11/82; 3/89  - | - | - | - | - |
| Prasad et al., 2007^99,100^ | Non-stimulant (ATX); Control | 8/104; 6/97  8/104; 8/97 | - | - | - | - |
| van den Hoofdakker et al., 2007^101^ | BT (parent training); Control | - | - | - | - | - |
| van der Oord et al., 2007^102^ | Stimulant (MPH-SA); Stimulant+BT (MPH-SA+child, parent and teacher training) | - | - | - | - | - |
| Wang et al., 2007^103,104^ | Stimulant (MPH-SA); Non-stimulant (ATX) | 42/166; 61/164  - | 9/166; 5/164 | - | - | Palpitations: 1/166; 0/164 |
| Amiri et al., 2008^105^ | Stimulant (MPH-SA); Other unlicensed drug (MODAF) | 26/30; 18/30  7/30; 3/30 | -  8/30; 2/30 | 4/30; 3/30 | - | - |
| Bangs et al., 2008^106,107^ | Non-stimulant (ATX); Placebo | 38/156; 1/70  60/156; 2/70 | - | - | - | Blood pressure increased: 14/156; 1/70 |
| Bierdeman et al., 2008^108-110^ | Non-stimulant α-2 agonist (GUAN-LA); Placebo | 18/259; 2/86  - | 17/259; 4/86  - | - | - | QT prolongation: 3/259; 1/86  Bradycardia: 1/259; 0/86 |
| Palumbo et al., 2008^111-113^ | Stimulant (MPH-SA); Non-stimulant α-2 agonist (CLON-SA); Stimulant (MPH-SA) + non-stimulant α-2 agonist (CLON-SA); Placebo | 4/29; 9/31; 3/32; 3/30  - | 1/29; 5/31; 4/32; 5/30  6/29; 5/31; 3/32; 3/30 | - | - | ECG abnormal: 0/29; 0/31; 1/32; 0/30  Tachycardia: 1/29; 0/31; 0/32; 0/30  Palpitations: 1/29; 0/31; 0/32; 0/30 |
| Findling et al., 2008^114^ | Stimulant (MPH-LA); Stimulant (MPH-TS); Placebo | 17/94; 25/100; 4/88  7/94; 9/100; 0/88 | 7/94; 13/100; 4/88 | - | - | - |
| Heriot et al., 2008^115^ | Stimulant (MPH-SA); BT (parent training); Stimulant+BT (MPH-SA+parent training); Placebo | - | - | - | - | - |
| Konofal et al., 2008^116^ | Minerals (Iron); Placebo | - | - | - | - | - |
| Newcorn et al., 2008^117,118^ | Stimulant (MPH-LA); Non-stimulant (ATX); Placebo | 37/220; 31/222; 2/74  90/220; 68/222; 1/74 | 29/220; 15/222; 1/74  - | - | - | Blood pressure increased: 8/220; 6/222; 1/74  Increased pulse values: 3/220; 4/222; 0/74 |
| Torrioli et al., 2008^119^ | Aminoacids (L-carnitine); Placebo | - | - | - | - | - |
| Vaisman et al., 2008^120^ | PUFA (omega-3 fatty acid); Control (fish oil); Placebo | - | - | - | - | - |
| Weber et al., 2008^121^ | Herbal therapy (St John’s Wort); Placebo | - | - | - | - | - |
| Arabgol et al., 2009^122^ | Stimulant (MPH-SA); Antidepressant (REBOX) | 5/16; 5/17  - | 3/16; 1/17  - | - | - | - |
| Block et al., 2009^123,124^ | Non-stimulant (ATX); Placebo | 21/195; 3/93  - | - | - | - | Increased heart rate: 13/195; 1/93  QT prolongation: 1/195; 0/93 |
| Childress et al., 2009^125^ | Stimulant (MPH-LA); Placebo | 22/188; 3/65  4/188; 0/65 | 18/188; 2/65  - | 3/188; 0/65 | - | Blood pressure increased: 2/188; 0/65  Increased pulse values:  10/188; 0/65  ECG abnormal: 7/188; 3/65  QT prolongation: 2/188; 1/65 |
| Dell'Agnello et al., 2009^126^ | Non-stimulant (ATX); Placebo | 36/107; 3/32  6/107; 1/32 | 5/107; 2/32 | - | - | - |
| Johnson et al., 2009^127^ | PUFA (omega-3/6 fatty acid); Placebo | - | - | - | - | - |
| Kahbazi et al., 2009^128^ | Other unlicensed drug (MODAF); Placebo | 7/23; 2/23  2/23; 1/23 | -  4/23; 2/23 | 2/23; 2/23 | - | - |
| Montoya et al., 2009^129,130^ | Non-stimulant (ATX); Placebo | 31/100; 4/51  - | - | - | - | Tachycardia: 4/100; 3/51 |
| Nair et al., 2009^131^ | Non-stimulant α-2 agonist (CLON-SA); Other unlicensed drug (CARBA) | - | - | - | - | - |
| Pelsser et al., 2009^132^ | Restricted elimination diet (elimination diet); Waiting list | - | - | - | - | - |
| Raz et al., 2009^133^ | PUFA (omega-3/6 fatty acid); Placebo | - | - | - | - | - |
| Sallee et al., 2009^134-136^ | Non-stimulant α-2 agonist (GUAN-LA); Placebo | 1/258; 0/66  - | - | 1/258; 0/66 | - | Hypotension: 10/258; 1/66  Increased pulse values; 12/258; 1/66  Decreased pulse values: 12/258; 0/66  ECG abnormal: 2/258; 0/66 |
| Svanborg et al., 2009^137,138^ | Non-stimulant+BT (ATX+child and parent training); BT (child and parent training) + placebo | 17/49; 0/50 | - | - | - | - |
| Takahashi et al., 2009^139,140^ | Non-stimulant (ATX); Placebo | 22/183; 2/62  40/183; 0/62 | - | - | - | Increased blood pressure: 16/183; 4/62  Increased pulse/heart rate values: 14/183; 2/62  QT prolongation: 15/183; 2/62 |
| Perez-Alvarez et al., 2009^141^ | Stimulant (MPH-LA); HPT (humanistic psychotherapy); Stimulant+HPT (MPH-LA+humanistic psychotherapy) | - | - | - | - | - |
| Thompson et al., 2009^142^ | BT (parent training); Control | - | - | - | - | - |
| Tramontina et al., 2009^143^ | Antipsychotic (ARIP); Placebo | - | - | - | - | - |
| Tucker et al., 2009^144^ | Stimulant+BT (MPH-LA+child and parent training); BT (child and parent training) | - | - | - | - | - |
| Gevensleben et al., 2009^145-147^ | Neurofeedback (theta-beta and slow cortical potential training); Cognitive training (attention training) | - | - | - | - | - |
| Connor et al., 2010^148,149^ | Non-stimulant α-2 agonist (GUAN-LA); Placebo | - | - | - | - | Decreased blood pressure: 8/138; 1/79  Decreased heart rate: 7/138; 1/79  Sinus bradycardia: 24/138; 4/79  Ectopic supraventricular rhythm: 1/138; 0/79  QT prolongation: 0/138; 1/79 |
| Fabiano et al., 2010^150^ | BT (child, parent and teacher training); Control | - | - | - | - | - |
| Findling et al., 2010^151^ | Stimulant (MPH-TS); Placebo | 37/145; 1/72  8/145; 1/72 | 9/145; 2/72  - | - | 1/145; 0/72 | Increased blood pressure: 1/145; 0/72  Tachycardia: 1/145; 0/72  Palpitations: 1/145; 0/72  Increased heart rate: 2/145; 0/72 |
| Gustafsson et al., 2010^152^ | PUFA (omega-3 fatty acid); Placebo | - | - | - | - | - |
| Martenyi et al., 2010^153,154^ | Non-stimulant (ATX); Placebo | 13/72; 2/33  7/72; 2/33 | 2/72; 0/33  0/72; 1/33 | - | - | - |
| Perreau-Linck et al., 2010^155^ | Neurofeedback (theta-beta training); Placebo | - | - | - | - | - |
| Salehi et al., 2010^156^ | Stimulant (MPH-SA); Herbal therapy (*Ginkgo biloba*) | 19/25; 5/25  12/15; 3/25 | 8/25; 3/25 | 9/25; 7/25 | - | - |
| Thurstone et al., 2010^157,158^ | Non-stimulant+BT (ATX+child and parent training); BT (child and parent training) + placebo | 21/35; 13/35  - | -  21/35; 25/35 | - | - | Tachycardia: 6/35; 4/35 |
| Waxmonsky et al., 2010^159^ | Non-stimulant+BT (ATX+child, parent and teacher training); Non-stimulant (ATX) | - | - | - | - | - |
| Zarinara et al., 2010^160^ | Stimulant (MPH-SA); Antidepressant (VENLAF) | 7/19; 2/19  - | 10/19; 2/19  - | - | - | - |
| Abbasi et al., 2011^161^ | Stimulant (MPH-SA); Stimulant + aminoacids (MPH-SA+L-carnitine) | 12/20; 14/20  6/20; 7/20 | -  10/20; 10/20 | 9/20; 9/20 | - | - |
| Arnold et al., 2011^162^ | Minerals (Zinc supplementation); Placebo | 7/28; 4/24  - | -  1/28; 4/24 | 8/28; 6/24 | - | - |
| Bakhshayesh et al., 2011^163^ | Neurofeedback (theta-beta training); Control | - | - | - | - | - |
| Dittmann et al., 2011^164^ | Non-stimulant (ATX); Placebo | 202/121; 1/59 | - | - | - | Tachycardia: 1/121; 0/59 |
| Findling et al., 2011^165,166^ | Stimulant (LDX-LA); Placebo | 79/235; 2/79  22/235; 0/79 | 26/235; 3/79  - | - | - | Increased blood pressure: 7/235; 2/79  Increased pulse/heart rate values: 7/235; 1/79  QT prolongation: 3/235; 0/79 |
| Jain et al., 2011^167^ | Non-stimulant α-2 agonist (CLON-LA); Placebo | - | 9/158; 1/78 | - | - | Bradycardia: 207158; 0/78  QT prolongation: 19/158; 11/78 |
| Giblin et al., 2011^168^ | Stimulant (LDX-LA); Placebo | - | - | - | - | Increased blood pressure: 2/16; 1/8  Increased pulse values: 5/16; 0/8 |
| Kang et al., 2011^169^ | Stimulant+BT (MPH-SA+child training); Stimulant+Exercise (MPH-SA+physical activity/sports) | - | - | - | - | - |
| Kollins et al., 2011^170^ | Stimulant+non-stimulant α-2 agonist (MPH-LA or LDX-LA+CLON-LA); Stimulant (MPH-LA or LDX) | - | 5/102; 3/96 | - | - | Increased heart rate: 0/102; 1/96 |
| Kollins et al., 2011^171^ | Non-stimulant α-2 agonist (GUAN-LA); Placebo | - | - | - | - | - |
| Kratochvil et al., 2011^172,173^ | Non-stimulant+BT (ATX+parent training); BT (parent training) | 13/50; 4/51  2/50; 2/51 | 1/50; 3/51  - | 1/50; 1/51 | - | - |
| Lansbergen et al., 2011^174^ | Neurofeedback (IFBT); Placebo | - | - | - | - | - |
| Pelsser et al., 2011^175^ | Restricted elimination diet (elimination diet); Control | - | - | - | - | - |
| Riggs et al., 2011^176^ | Stimulant+BT (MPH-LA+child training); BT (child training) + placebo | - | - | 0/151; 1/152 | - | - |
| Steiner et al., 2011^177^ | Neurofeedback (theta-beta training); Control; Waiting list | - | - | - | - | - |
| Wehmeier et al., 2011^178,179^ | Non-stimulant (ATX); Placebo | 3/63; 0/62  - | - | - | - | - |
| Wilens et al., 2011^180^ | Non-stimulant (ATX); Placebo | - | -  - | - | - | - |
| Yildiz et al., 2011^181^ | Stimulant (MPH-LA); Non-stimulant (ATX) | 9712; 12/17  5/12; 8/17 | 7/12; 5/17  - | - | - | - |
| Zamora et al., 2011^182^ | Stimulant (MPH-SA); Stimulant+minerals (MPH-SA+Zinc sulfate) | - | - | - | - | - |
| Assareh et al., 2012^183^ | Stimulant+PUFA (MPH-SA+omega-3/6 fatty acid); Stimulant (MPH-SA)+Placebo | - | - | - | - | - |
| Duric et al., 2012^184^ | Stimulant (MPH-SA); Neurofeedback (theta-beta training); Stimulant+neurofeedback (MPH-SA+theta-beta training) | - | - | - | - | - |
| Fabiano et al., 2012^185^ | BT (parent training); Waiting list | - | - | - | - | - |
| Green et al., 2012^186^ | Cognitive training (WM training); Placebo | - | - | - | - | - |
| Jafarinia et al., 2012^187^ | Stimulant (MPH-SA); Antidepressant (BUP) | 11/22; 9/22  - | 10/22; 7/22  - | 5/22; 3/22 | - | Tachycardia: 1/22; 2/22 |
| Manor et al., 2012^188,189^ | PUFA (omega-3 fatty acid); Placebo | 36/137; 17/63  - | 42/137; 28/63  - | 50/137; 33/63 | - | Tachycrdia: 2/137; 0/63 |
| Perera et al., 2012^190^ | Stimulant+PUFA (MPH-SA+omega-3/6 fatty acid); Stimulant (MPH-SA)+Placebo | - | - | - | - | - |
| Wilens et al., 2012^191-194^ | Stimulant+non-stimulant α-2 agonist (MPH-LA or LDX-LA+GUAN-LA); Stimulant (MPH-LA or LDX) | 26/307; 6/154  - | 20/307; 6/154  - | - | 1/307; 0/154 | - |
| Abikoff et al., 2013^195^ | BT (child, parent and teacher training), Waiting list | - | - | 1/125; 0/33 | - | - |
| Arnold et al., 2013^196^ | Neurofeedback (theta-beta training); Placebo | - | - | - | - | - |
| Coghill et al., 2013^197-200^ | Stimulant (LDX-LA); Stimulant (MPH-LA); Placebo | 28/113; 17/112; 3/111  35/113; 12/112; 0/111 | 16/113; 9/112; 0/111  6/113; 2/112; 1/111 | - | 1/113; 1/112; 0/111 | QT prolongation: 3/113; 1/112; 1/111 |
| Dittmann et al., 2013^201-204^ | Stimulant (LDX-LA); Non-stimulant (ATX) | 33/133; 14/134  34/133; 6/134 | 15/133; 8/134  - | - | - | Increased blood pressure: 32/133; 27/134  Increased pulse values: 19/133; 32/134  Decresed pulse values: 4/133; 0/134 |
| Hovik et al., 2013^205,206^ | Cognitive training (WM training); Control | - | - | - | - | - |
| Li et al., 2013^207^ | Stimulant+neurofeedback (MPH-SA+theta-beta training); Stimulant (MPH-SA) | - | - | - | - | - |
| Newcorn et al., 2013^208-211^ | Non-stimulant α-2 agonist (GUAN-LA); Placebo | 18/227; 3/113  - | 18/227; 4/113  - | - | 2/227; 0/113 | - |
| Ghanizadeh et al., 2013^212^ | Stimulant+vitamins (MPH-SA+vitamin B9/folic acid); Stimulant (MPH-SA) | 2/23; 1/23  - | - | - | - | - |
| Oberai et al., 2013^213^ | Homeopathy (HOMEO); Placebo | - | - | - | - | - |
| Ogrim et al., 2013^213^ | Stimulant (MPH-LA or DEXAM); Neurofeedback (theta-beta training) | - | - | - | - | - |
| Simonoff et al., 2013^214^ | Stimulant (MPH-SA); Placebo | 9/61; 1/61  - | -  13/61; 2/61 | 2/61; 1/61 | - | Increased blood pressure: 20/61; 17/61  Increased pulse values: 19/133; 23/61; 23/61 |
| Tamm et al., 2013^215^ | Cognitive training (attention training); Waiting list | - | - | - | - | - |
| van Dongen-Boomsma et al., 2013^217^ | Neurofeedback (theta-beta training); Placebo | - | - | - | - | - |
| Aman et al., 2014^218-220^ | Stimulant+antipsychotic+BT (MPH-LA or MIX-AMPH+RISP+parent training); Stimulant+BT (MPH-LA or MIX-AMPH+parent training) | 9/84; 19/84  - | -  14/84; 29/84 | - | - | - |
| Barragán et al., 2014^221^ | Stimulant (MPH-SA)+PUFA (omega-3/6 fatty acid); Stimulant (MPH-SA); PUFA (omega-3/6 fatty acid) | - | 0/30; 6/30; 0/30  - | - | - | Palpitations: 5/30; 7/30; 0/30 |
| Chacko et al., 2014^222^ | Cognitive training (WM training); Placebo | - | - | - | - | - |
| Ferrin et al., 2014^223^ | BT (parent training); Control (parent counseling with support) | - | - | - | - | - |
| Garg et al., 2014^224^ | Stimulant (MPH-SA); Non-stimulant (ATX) | 14/41; 12/43  - | 1/41; 0/43  - | - | - | - |
| Hervas et al., 2014^225-227^ | Non-stimulant (ATX); Non-stimulant α-2 agonist (GUAN-LA); Placebo | 31/112; 15/115; 12/111  8/112; 0/155; 2/111 | 8/112; 13/115; 7/111  - | 7/112; 9/115; 8/111 | 0/112; 1/115; 1/111 | Decreased blood pressure: 4/112; 5/115; 5/111  Tachycardia: 2/112; 2/115; 0/111 |
| Hirayama et al., 2014^228^ | Aminoacids (phosphatidyl-serine); Placebo | - | - | - | - | - |
| Ko et al., 2014^229^ | Herbal therapy (*Ginseng*); Placebo | - | - | - | - | - |
| Lin et al., 2014^230,231^ | Stimulant (MPH-LA); Non-stimulant (EDIVOX); Placebo | 17/36; 24/226; 3/78  4/36; 1/226; 0/78 | 7/36; 11/226; 5/78  4/36; 1/226; 0/78 | - | - | Increased heart rate: 1/36; 6/226; 0/78 |
| Meisel et al., 2014^232^ | Stimulant (MPH-SA); Neurofeedback (theta-beta training) | - | - | - | - | - |
| Pfiffner et al., 2014^233^ | BT (child, parent and teacher training); BT (parent training); Control (usual care) | - | - | - | - | - |
| Steiner et al., 2014^234,235^ | Cognitive training (attention training); Neurofeedback (theta-beta training); Control | - | - | - | - | - |
| van Dongen-Boomsma et al., 2014^236^ | Cognitive training (WM training); Placebo | - | - | - | - | - |
| Widenhorn-Müller et al., 2014^237^ | PUFA (omega-3 fatty acid); Placebo | - | - | - | - | - |
| Abikoff et al., 2015^238^ | BT (parent training); Waiting list | - | - | - | - | - |
| Bigorra et al., 2015^239^ | Cognitive training (WM training); Placebo | - | - | - | - | - |
| Bédard et al., 2015^240^ | Non-stimulant α-2 agonist (GUAN-LA); Placebo | - | - | - | - | - |
| Bos et al., 2015^241^ | PUFA (omega-3 fatty acid); Placebo | - | - | - | - | - |
| Choi et al., 2015^242^ | Stimulant+BT (MPH-SA+child training); Stimulant+Exercise (MPH-SA+physical activity/sports) | - | - | - | - | - |
| Choi et al., 2015^243^ | BT (child training); Waiting list | - | - | - | - | - |
| Chou et al., 2015^244^ | Stimulant (MPH-LA); Non-stimulant (ATX) | - | - | - | - | - |
| Corkum et al., 2015^245^ | BT (teacher training); Waiting list | - | - | - | - | - |
| Ghanizadeh et al., 2015^246^ | Stimulant+restricted elimination diet (MPH-SA+elimination diet); Stimulant (MPH-SA) | - | - | - | - | - |
| Hiscock et al., 2015^247^ | BT (parent training); Control (usual care) | - | - | - | - | - |
| Matsudaira et al., 2015^248^ | PUFA (omega-3/6 fatty acid); Placebo | - | - | - | - | - |
| Shakibaei et al., 2015^249^ | Stimulant+herbal therapy (MPH-SA+*Ginkgo biloba*); Stimulant (MPH-SA) | 2/33; 7/33  - | - | - | - | Palpitations: 0/33; 1/33 |
| Shang et al., 2015^250^ | Stimulant (MPH-LA); Non-stimulant (ATX) | 36/80; 38/80  - | 12/80; 4/80  - | - | - | Palpitations: 1/80; 0/80 |
| Storebø et al., 2015^251,252^ | BT (child and parent training); Control (usual care) | - | - | - | - | - |
| Wilens et al., 2015^253-255^ | Non-stimulant α-2 agonist (GUAN-LA); Placebo | 23/157; 21/157  - | 14/157; 6/157  - | - | 1/157; 0/157 | Decreased blood pressure: 17/157; 9/157  Bradycardia: 6/157; 0/157 |
| Arabgol et al., 2015^256^ | Antipsychotics (RISP); Stimulant (MPH-SA) | 4/20; 8/18  - | -  0/20; 4/18 | - | - | - |
| Correia-Filho et al., 2005^257^ | Antipsychotics (RISP); Stimulant (MPH-SA) | - | - | - | - | - |
| Ferrin et al., 2016^258^ | BT (parent training); Control (usual care) | - | - | - | - | - |
| Janssen et al., 2016^259,260^ | Stimulant (MPH-SA); Neurofeedback (theta-beta training); Exercise (physical activity/sports) | - | - | - | - | - |
| Steeger et al., 2016^261^ | BT (parent training); Cognitive training (WM training); BT+cognitive training (parent training+WM training); Placebo | - | - | - | - | - |
| Su et al., 2016^262^ | Stimulant (MPH-LA); Non-stimulant (ATX) | 96/130; 86/132  - | -  51/130; 46/132 | 34/130; 19/132 | - | - |
| Newcorn et al., 2016^263,264^ | Non-stimulant α-2 agonist (GUAN-LA); Placebo | - | - | - | 0/157; 1/59 | Hypotension: 1/157; 0/159 |
