# Supplementary material for: The pharmacological and non-pharmacological treatment of attention deficit hyperactivity disorder in children and adolescents: A systematic review with network meta-analyses of randomised trials
Source: PLoS One. 2017 Jul 12;12(7):e0180355. doi: 10.1371/journal.pone.0180355 (PMC5507500; doi:10.1371/journal.pone.0180355)
Supplement: S6 Table — (DOCX) [file pone.0180355.s011.docx]

**S6 Table. Network meta-analyses for efficacy and acceptability of classes of interventions**

| **PBO** | **0.55**  **(0.32-0.95)** | **0.33**  **(0.15-0.70)** | **0.58**  **(0.33-0.99)** | 1.32  (0.71-2.52) | 0.59  (0.31-1.14) | **0.67**  **(0.54-0.83)** | **0.81**  **(0.67-0.97)** | 0.99  (0.44-2.28) | 1.25  (0.43-3.63) | 0.73  (0.42-1.29) | 0.79  (0.23-2.76) | 1.09  (0.68-1.74) | 0.59  (0.24-1.44) | 1.14  (0.49-2.72) | 0.53  (0.15-1.88) | 0.56  (0.18-1.73) | 0.44  (0.07-2.19) | **0.37**  **(0.21-0.67)** | 1.00  (0.37-2.84) | **0.47**  **(0.27-0.81)** |
| --- | --- | --- | --- | --- | --- | --- | --- | --- | --- | --- | --- | --- | --- | --- | --- | --- | --- | --- | --- | --- |
| 1.99  (0.98-4.15) | **CONT** | 0.61  (0.29-1.22) | 1.05  (0.64-1.71) | **2.40**  **(1.24-4.71)** | 1.08  (0.51-2.26) | 1.22  (0.71-2.07) | 1.47  (0.85-2.53) | 1.80  (0.69-4.76) | 2.28  (0.69-7.46) | 1.33  (0.61-2.88) | 1.44  (0.46-4.70) | 1.98  (0.99-3.89) | 1.09  (0.37-3.01) | 2.09  (0.76-5.68) | 0.96  (0.24-3.91) | 1.02  (0.29-3.62) | 0.80  (0.13-4.28) | 0.69  (0.37-1.25) | 1.83  (0.66-5.11) | 0.86  (0.40-1.80) |
| 0.57  (0.20-1.62) | 0.29  (0.11-0.72) | **WL** | 1.73  (0.97-3.19) | **4.00**  **(1.76-9.07)** | 1.78  (0.73-4.43) | 2.01  (0.96-4.32) | **2.44**  **(1.14-5.28)** | 3.00  (0.99-9.31) | **3.76**  **(1.04-14.14)** | 2.19  (0.87-5.72) | 2.38  (0.67-8.78) | **3.28**  **(1.37-7.92)** | 1.81  (0.55-5.87) | 3.46  (1.14-10.75) | 1.61  (0.36-7.06) | 1.70  (0.43-6.54) | 1.32  (0.19-7.54) | 1.13  (0.55-2.39) | **3.04**  **(1.02-9.23)** | 1.42  (0.57-3.54) |
| **2.97**  **(1.53-5.88)** | 1.49  (0.86-2.56) | **5.24**  **(2.35-12.07)** | **BEHAV** | **2.29**  **(1.15-4.62)** | 1.03  (0.48-2.20) | 1.16  (0.68-1.96) | 1.40  (0.80-2.42) | 1.73  (0.66-4.62) | 2.17  (0.66-7.10) | 1.27  (0.59-2.76) | 1.37  (0.41-4.71) | 1.89  (0.93-3.76) | 1.04  (0.35-2.92) | 1.98  (0.74-5.44) | 0.92  (0.23-3.66) | 0.98  (0.27-3.43) | 0.76  (0.12-4.05) | 0.65  (0.40-1.04) | 1.75  (0.68-4.47) | 0.81  (0.38-1.72) |
| 0.70  (0.12-3.87) | 0.35  (0.05-2.21) | 1.23  (0.16-8.99) | 0.23  (0.04-1.46) | **COG** | **0.45**  **(0.21-0.95)** | **0.51**  **(0.26-0.95)** | 0.61  (0.32-1.15) | 0.75  (0.27-2.11) | 0.95  (0.27-3.22) | 0.55  (0.24-1.28) | 0.60  (0.17-2.20) | 0.82  (0.37-1.76) | 0.45  (0.15-1.33) | 0.87  (0.30-2.47) | 0.40  (0.09-1.66) | 0.43  (0.11-1.55) | 0.33  (0.05-1.80) | **0.28**  **(0.13-0.60)** | 0.76  (0.25-2.35) | **0.36**  **(0.15-0.80)** |
| 1.96  (0.52-8.26) | 0.99  (0.22-4.83) | 3.45  (0.66-20.34) | 0.66  (0.15-3.17) | 2.85  (0.71-11.97) | **NF** | 1.13  (0.59-2.13) | 1.37  (0.70-2.63) | 1.68  (0.59-4.79) | 2.11  (0.61-7.13) | 1.23  (0.53-2.90) | 1.33  (0.36-5.16) | 1.83  (0.84-4.03) | 1.01  (0.33-3.00) | 1.93  (0.67-5.70) | 0.89  (0.21-3.64) | 0.95  (0.25-3.49) | 0.74  (0.13-3.70) | 0.63  (0.28-1.41) | 1.70  (0.53-5.50) | 0.79  (0.34-1.82) |
| **6.21**  **(4.89-7.96)** | **3.12**  **(1.54-6.28)** | **10.94**  **(3.96-31.49)** | **2.09**  **(1.10-3.99)** | **8.89**  **(1.59-50.19)** | 3.16  (0.76-12.05) | **STI** | 1.21  (0.96-1.52) | 1.49  (0.65-3.44) | 1.87  (0.64-5.39) | 1.09  (0.61-1.99) | 1.18  (0.35-4.09) | 1.63  (0.98-2.67) | 0.90  (0.35-2.22) | 1.72  (0.72-4.16) | 0.79  (0.22-2.86) | 0.84  (0.26-2.65) | 0.66  (0.11-3.25) | **0.56**  **(0.32-0.98)** | 1.51  (0.56-4.26) | 0.70  (0.41-1.19) |
| **3.95**  **(3.13-5.07)** | 1.99  (0.97-4.05) | **6.95**  **(2.49-20.34)** | 1.33  (0.68-2.62) | **5.68**  **(1.00-32.28)** | 2.02  (0.48-7.81) | **0.64**  **(0.49-0.84)** | **N-STI** | 1.23  (0.54-2.86) | 1.55  (0.53-4.52) | 0.90  (0.61-1.99) | 0.98  (0.29-3.40) | 1.34  (0.81-2.21) | 0.74  (0.29-1.82) | 1.42  (0.60-3.34) | 0.66  (0.18-2.35) | 0.70  (0.22-2.19) | 0.54  (0.09-2.74) | **0.46**  **(0.26-0.83)** | 1.24  (0.45-3.50) | 0.58  (0.33-1.01) |
| **8.52**  **(3.95-18.96)** | **4.29**  **(1.51-12.16)** | **15.07**  **(4.19-55.40)** | **2.88**  **(1.06-8.00)** | **12.33**  **(1.85-82.43)** | 4.33  (0.87-20.33) | 1.37  (0.64-3.01) | 2.16  (0.97-4.86) | **AD** | 1.25  (0.33-4.80) | 0.73  (0.27-1.97) | 0.79  (0.18-3.46) | 1.09  (0.42-2.77) | 0.60  (0.17-1.99) | 1.15  (0.35-3.74) | 0.53  (0.12-2.46) | 0.56  (0.14-2.27) | 0.44  (0.06-2.66) | 0.38  (0.14-1.00) | 1.01  (0.28-3.71) | 0.47  (0.18-1.23) |
| 1.36  (0.34-5.38) | 0.68  (0.15-3.14) | 2.40  (0.44-13.25) | 0.46  (0.10-2.07) | 1.95  (0.22-17.03) | 0.69  (0.10-4.59) | **0.22**  **(0.05-0.85)** | 0.34  (0.09-1.37) | **0.16**  **(0.03-0.77)** | **A-PSY** | 0.58  (0.17-1.95) | 0.63  (0.13-3.29) | 0.87  (0.27-2.77) | 0.48  (0.12-1.92) | 0.92  (0.24-3.62) | 0.43  (0.08-2.17) | 0.45  (0.09-2.11) | 0.35  (0.04-2.43) | **0.30**  **(0.09-0.98)** | 0.80  (0.18-3.50) | 0.37  (0.11-1.23) |
| **3.80**  **(2.04-7.14)** | 1.90  (0.74-4.86) | **6.72**  **(2.02-22.91)** | 1.28  (0.52-3.19) | 5.48  (0.88-34.01) | 1.94  (0.41-8.52) | 0.61  (0.32-1.18) | 0.96  (0.50-1.85) | 0.44  (0.16-1.20) | 2.81  (0.62-12.57) | **O-DRU** | 1.08  (0.29-4.21) | 1.49  (0.71-3.07) | 0.82  (0.28-2.32) | 1.57  (0.57-4.40) | 0.73  (0.18-2.90) | 0.77  (0.21-2.70) | 0.60  (0.09-3.26) | 0.51  (0.23-1.13) | 1.37  (0.43-4.38) | 0.64  (0.29-1.40) |
| 2.06E+7  (459.2-1.31E+22) | 1.03E+7  (234.2-6.32E+21) | 3.71E+7  (806.7-2.06E+22) | 6.92E+6  (159.7-4.00E+21) | 3.01E+7  (575.6-1.91E+22) | 9.99E+6  (216.6-6.71E+21) | 3.30E+6  (74.34-2.05E+21) | 5.21E+6  (115.4-3.19E+21) | 2.40E+6  (52.03-1.54E+21) | 1.50E+7  (309.6-9.96E+21) | 4.42E+6  (122.8-3.48E+21) | **DIET** | 1.37  (0.37-4.96) | 0.76  (0.16-3.39) | 1.46  (0.32-6.40) | 0.67  (0.11-3.90) | 0.71  (0.13-3.77) | 0.55  (0.06-4.01) | 0.47  (0.13-1.67) | 1.27  (0.28-5.77) | 0.59  (0.15-2.20) |
| 2.14  (0.83-5.57) | 1.07  (0.33-3.49) | 3.78  (0.93-15.75) | 0.72  (0.23-2.29) | 3.08  (0.43-22.22) | 1.08  (0.20-5.73) | **0.34**  **(0.13-0.90)** | 0.54  (0.21-1.43) | **0.25**  **(0.07-0.85)** | 1.58  (0.30-8.43) | 0.56  (0.18-1.74) | 1.08E-7  (1.74E-22-0.00) | **PUFA** | 0.55  (0.20-1.49) | 1.06  (0.41-2.81) | 0.49  (0.12-1.91) | 0.52  (0.15-1.77) | 0.40  (0.06-2.15) | **0.35**  **(0.17-0.72)** | 0.93  (0.31-2.84) | **0.43**  **(0.21-0.89)** |
| 1.19  (0.25-5.71) | 0.60  (0.11-3.33) | 2.12  (0.32-14.06) | 0.40  (0.07-2.18) | 1.71  (0.17-17.71) | 0.61  (0.07-4.79) | **0.19**  **(0.04-0.93)** | 0.30  (0.06-1.46) | **0.14**  **(0.02-0.80)** | 0.88  (0.11-7.11) | 0.31  (0.06-1.68) | 5.92E-8  (8.37E-23-0.00) | 0.56  (0.09-3.39) | **AMIN** | 1.92  (0.56-6.67) | 0.89  (0.19-4.31) | 0.94  (0.22-4.06) | 0.73  (0.10-4.72) | 0.63  (0.22-1.86) | 1.67  (0.43-6.88) | 0.78  (0.28-2.27) |
| 2.93  (0.90-10.15) | 1.47  (0.36-6.21) | **5.18**  **(1.08-26.91)** | 0.99  (0.25-4.05) | 4.22  (0.53-34.66) | 1.50  (0.23-8.99) | 0.47  (0.14-1.67) | 0.74  (0.22-2.61) | 0.34  (0.08-1.48) | 2.19  (0.35-13.58) | 0.77  (0.20-3.13) | 1.46E-7  (2.20E-22-0.01) | 1.37  (0.31-6.49) | 2.46  (0.35-18.13) | **MIN** | 0.46  (0.10-2.10) | 0.49  (0.12-2.00) | 0.38  (0.05-2.38) | **0.33**  **(0.12-0.91)** | 0.87  (0.24-3.36) | 0.41  (0.14-1.13) |
| 0.59  (0.17-1.99) | 0.30  (0.07-1.20) | 1.04  (0.20-5.16) | 0.20  (0.05-0.78) | 0.84  (0.10-6.83) | 0.30  (0.05-1.80) | **0.10**  **(0.03-0.32)** | **0.15**  **(0.04-0.51)** | **0.07**  **(0.02-0.29)** | 0.43  (0.07-2.71) | **0.16**  **(0.04-0.61)** | 2.88E-8  (4.22E-23-0.00) | 0.28  (0.06-1.26) | 0.49  (0.07-3.58) | 0.20  (0.03-1.07) | **HERB** | 1.06  (0.19-5.79) | 0.82  (0.09-6.56) | 0.71  (0.17-2.88) | 1.90  (0.37-10.06) | 0.88  (0.22-3.51) |
| - | - | - | - | - | - | - | - | - | - | - | - | - | - | - | - | **HOMEO** | 0.78  (0.09-5.83) | 0.67  (0.19-2.42) | 1.79  (0.40-8.45) | 0.83  (0.24-3.01) |
| - | - | - | - | - | - | - | - | - | - | - | - | - | - | - | - | - | **PHYS** | 0.86  (0.16-5.49) | 2.31  (0.35-17.58) | 1.07  (0.19-6.93) |
| **13.62**  **(6.83-27.93)** | **6.83**  **(3.30-14.40)** | **23.96**  **(8.83-68.98)** | **4.58**  **(2.49-8.75)** | **19.76**  **(3.14-123.90)** | **6.94**  **(1.45-30.69)** | **2.19**  **(1.14-4.34)** | **3.44**  **(1.73-7.05)** | 1.60  (0.58-4.48) | **10.03**  **(2.25-46.71)** | **3.59**  **(1.44-9.17)** | 6.62E-7  (1.11E-21-0.03) | **6.41**  **(2.02-20.94)** | **11.39**  **(2.11-64.41)** | **4.63**  **(1.14-18.91)** | **23.25**  **(5.77-98.61)** | - | - | **STI+BEHAV** | 2.68  (0.96-7.63) | 1.25  (0.57-2.68) |
| **6.05**  **(2.39-15.27)** | **3.03**  **(1.21-7.53)** | **10.71**  **(3.53-32.77)** | 2.04  (0.95-4.38) | **8.70**  **(1.26-59.30)** | 3.07  (0.57-15.26) | 0.97  (0.39-2.43) | 1.53  (0.61-3.83) | 0.71  (0.21-2.31) | 4.45  (0.86-22.94) | 1.60  (0.52-4.74) | 2.98E-7  (5.15E-22-0.01) | 2.83  (0.77-10.48) | 5.05  (0.83-31.13) | 5.05  (0.83-31.13) | **10.30 (2.26-48.55)** | - | - | 0.44  (0.17-1.13) | **N-STI+ BEHAV** | 0.47  (0.15-1.45) |
| **15.18**  **(7.50-31.46)** | **7.62**  **(2.88-20.25)** | **26.72**  **(7.96-93.71)** | **5.09**  **(2.01-13.18)** | **21.85**  **(3.47-140.0)** | **7.76**  **(1.60-34.62)** | **2.45**  **(1.24-4.87)** | **3.84**  **(1.88-7.93)** | 1.78  (0.64-4.93) | **11.18**  **(2.47-51.76)** | **3.99**  **(1.58-10.16)** | 7.44E-7  (1.22E-21-0.03) | 7**.09**  **(2.21-23.0)** | **12.71**  **(2.30-70.72)** | **12.71**  **(1.30-70.72)** | **25.78**  **(6.42-109.10)** | - | - | 1.11  (0.43-2.88) | 2.50  (0.82-7.86) | **STI+N-STI** |

Data in blue represents efficacy (treatment response). Data in red represents acceptability (all-cause discontinuation). Results are the ORs in the column-defining treatment compared with the ORs in the row-defining treatment. For efficacy (acceptability), ORs higher than 1 favour the row-defining treatment. For acceptability, ORs lower than 1 favour the row-defining treatment. Significant results are in bold and underscored. PBO=placebo. CONT=control. WL=waiting list. BEHAV=behavioural therapy. COGN=cognitive training. NF=neurofeedback. STI=stimulants. N-STI=non-stimulants. AD=antidepressants. A-PSY=antipsychotics. O-DRU=other unlicensed drugs. DIET=dietary therapy. PUFA= polyunsaturated fatty acids. AMIN=amino acids. MIN=minerals. HERB=herbal therapy. HOMEO=homeopathy. PHYS=physical activity. STI+BEHAV=stimulants+behavioural therapy. STI+N-STI=stimulants+non-stimulants. N-STI+BEHAV=non-stimulants+behavioural therapy. OR=Odds ratio. CI=credibility interval
