# Supplementary material for: The pharmacological and non-pharmacological treatment of attention deficit hyperactivity disorder in children and adolescents: A systematic review with network meta-analyses of randomised trials
Source: PLoS One. 2017 Jul 12;12(7):e0180355. doi: 10.1371/journal.pone.0180355 (PMC5507500; doi:10.1371/journal.pone.0180355)
Supplement: S7 Table — (DOCX) [file pone.0180355.s012.docx]

**S7 Table. Quality of evidence (GRADE) for primary outcomes**

**Network meta-analysis for efficacy. Class effect.**

|  | **Trials** | **Events and participants** | **Class effect,**  **OR (95% CrI)** | **Quality of evidence**  **(GRADE)** |
| --- | --- | --- | --- | --- |
| **Controls** |  |  |  |  |
| **Placebo** | 65 | 929/4070 | Reference | Reference |
| **Control (e.g., usual care)** | 8 | 148/545 | 1.99 (0.98-4.15) | + ○ ○ ○ VERY LOW^2,4^ |
| **Waiting list** | 5 | 25/121 | 0.57 (0.20-1.62) | + ○ ○ ○ VERY LOW^2,4^ |
| **Psychological interventions** |  |  |  |  |
| **Behavioural therapy** | 15 | 396/1016 | 2.97 (1.53-5.88)* | + + ○ ○ LOW^2^ |
| **Cognitive training** | 2 | 7/65 | 0.70 (0.12-3.87) | + ○ ○ ○ VERY LOW^2,4^ |
| **Other psychotherapies** | 1 | 10/32 | 7.02 (1.56-32.59)* | + ○ ○ ○ VERY LOW^2,4^ |
| **Neurofeedback** | 4 | 26/110 | 1.96 (0.52-8.26) | + ○ ○ ○ VERY LOW^2,4^ |
| **Pharmacological interventions** |  |  |  |  |
| **Stimulants** | 53 | 3464/5831 | 6.21 (4.89-7.96)* | + + ○ ○ LOW^2^ |
| **Non-stimulants** | 40 | 2447/4741 | 3.95 (3.13-5.07)* | + + ○ ○ LOW^2^ |
| **Antidepressants** | 6 | 69/128 | 8.52 (3.95-18.96)* | + ○ ○ ○ VERY LOW^2,4^ |
| **Antipsychotics** | 1 | 8/41 | 1.36 (0.34-5.38) | + ○ ○ ○ VERY LOW^2,4^ |
| **Other unlicensed drugs** | 6 | 248/572 | 3.80 (2.04-7.14)* | + ○ ○ ○ VERY LOW^2,4^ |
| **Complementary and alternative medicine interventions** |  |  |  |  |
| **Dietary therapy** | 2 | 43/65 | 2.07x10^7^ (0.46x10^3^-13.01x10^21^)† | + ○ ○ ○ VERY LOW^2,4^ |
| **Polyunsaturated fatty acids (or PUFAs)** | 3 | 41/124 | 2.14 (0.83-5.57) | + ○ ○ ○ VERY LOW^2,4^ |
| **Amino acids** | 1 | 9/58 | 1.19 (0.25-5.71) | + ○ ○ ○ VERY LOW^2,4^ |
| **Minerals** | 2 | 49/220 | 2.93 (0.90-10.15) | + ○ ○ ○ VERY LOW^2,4^ |
| **Herbal therapy** | 2 | 13/52 | 0.59 (0.17-1.99) | + ○ ○ ○ VERY LOW^2,4^ |
| **Combined interventions** |  |  |  |  |
| **Stimulants plus behavioural therapy** | 8 | 283/521 | 13.62 (6.83-27.93)* | + ○ ○ ○ VERY LOW^2,4^ |
| **Stimulants plus non-stimulants** | 4 | 313/480 | 15.18 (7.50-31.46)* | + ○ ○ ○ VERY LOW^2,4^ |
| **Non-stimulants plus behavioural therapy** | 4 | 86/163 | 6.05 (2.39-15.27)* | + ○ ○ ○ VERY LOW^2,4^ |
| **Stimulants plus antipsychotics plus behavioural therapy** | 1 | 63/84 | 18.19 (4.10-83.33)* | + ○ ○ ○ VERY LOW^2,4^ |
| **Stimulants plus antipsychotics** | 2 | 33/54 | 10.32 (3.49-32.11)* | + ○ ○ ○ VERY LOW^2,4^ |
| **Stimulants plus other psychotherapies** | 1 | 31/59 | 15.42 (3.38-70.31)* | + ○ ○ ○ VERY LOW^2,4^ |
| **Stimulants plus polyunsaturated fatty acids (or PUFAs)** | 2 | 47/50 | 16.81 (3.45-101.6)* | + ○ ○ ○ VERY LOW^2,4^ |
| **Stimulants plus minerals** | 1 | 11/20 | 18.67 (3.15-116.0)* | + ○ ○ ○ VERY LOW^2,4^ |
| **Stimulants plus herbal therapy** | 1 | 26/33 | 10.15 (1.97-54.8)* | + ○ ○ ○ VERY LOW^2,4^ |

Results are odds ratios (OR) with 95% credible Intervals and quality of evidence. An OR < 1 favours placebo (that is, more response events occur with placebo that with other intervention). *p < 0.05. †Extremely wide credible intervals owing to small patient and trial numbers and frequent events among treatment arm.

Reasons for downgrading the quality of evidence for a specific outcome:

1. Rated down 1 level for risk of bias (for example, the majority of the comparisons are at low/unclear risk of bias but the proportion of information from studies at high risk of bias is sufficient to affect the interpretation of results)
2. Rated down 2 levels for risk of bias (for example, very serious limitations for one or more risk of bias criteria and/or the majority of information from studies at high risk of bias sufficient to affect the interpretation of results).
3. Rated down 1 level for imprecision of results (for example, 95% credible intervals are wide and include or are close to null effect, or based on small number of studies/outcome events).
4. Rated down 2 level for imprecision of results (for example, 95% credible intervals include the null effect and/or are extremely wide).
5. Rated down 1 level for unexplained inconsistency of results (such as intransitivity or lack of consistency as per the DIC).

**Table: Network meta-analysis for efficacy. Individual effect.**

|  | **Trials** | **Events and participants** | **Individual effect,**  **OR (95% CrI)** | **Quality of evidence**  **(GRADE)** |
| --- | --- | --- | --- | --- |
| **Controls** |  |  |  |  |
| **Placebo** | 65 | 929/4070 | Reference | Reference |
| **Control (e.g., usual care)** | 8 | 148/545 | 1.29 (0.65-2.59) | + ○ ○ ○ VERY LOW^2,4^ |
| **Waiting list** | 5 | 25/121 | 0.29 (0.10-0.85)* | + ○ ○ ○ VERY LOW^2,3^ |
| **Psychological interventions** |  |  |  |  |
| **Behavioural therapy** | 15 | 396/1016 | - | - |
| Parent training | 8 | 128/357 | 1.19 (0.50-2.77) | + ○ ○ ○ VERY LOW^2,4^ |
| Child, parent and/or teacher training | 8 | 254/535 | 2.73 (1.41-5.39)* | + + ○ ○ LOW^2^ |
| **Cognitive training** | 2 | 7/65 | - | - |
| Working memory training | 1 | 1/27 | 0.34 (0.01-5.82) | + ○ ○ ○ VERY LOW^2,4^ |
| Attention training | 1 | 6/38 | 4.17x10^12^ (1.63-12.44x10^28^)† | + ○ ○ ○ VERY LOW^2,4^ |
| **Neurofeedback** | 4 | 26/110 | - | - |
| Neurofeedback/theta-beta training | 2 | 23/86 | 1.61x10^13^ (4.57-23.91x10^28^)† | + ○ ○ ○ VERY LOW^2,4^ |
| **Pharmacological interventions** |  |  |  |  |
| **Stimulants** | 53 | 3464/5831 | - | - |
| Methylphenidate | 40 | 2279/3836 | 5.26 (4.09-6.82)* | + + ○ ○ LOW^2^ |
| Amphetamine | 9 | 1017/1628 | 7.45 (5.10-11.09)* | + + ○ ○ LOW^2^ |
| **Non-stimulants** | 40 | 2447/4741 | - | - |
| Atomoxetine | 27 | 1547/3162 | 3.63 (2.81-4.73)* | + + ○ ○ LOW^2^ |
| Guanfacine | 10 | 837/1461 | 3.29 (2.27-4.82)* | + + ○ ○ LOW^2^ |
| Clonidine | 4 | 63/118 | 3.96 (1.89-8.41)* | + ○ ○ ○ VERY LOW^2,4^ |
| **Antidepressants** | 6 | 69/128 | - | - |
| Bupropion | 1 | 8/22 | 2.41 (0.48-11.63) | + ○ ○ ○ VERY LOW^2,4^ |
| Desipramine | 2 | 36/58 | 36.76 (9.17-214.0)* | + ○ ○ ○ VERY LOW^2,4^ |
| Venlafaxine | 1 | 12/19 | 4.07 (0.73-22.36) | + ○ ○ ○ VERY LOW^2,4^ |
| Reboxetine | 1 | 5/17 | 3.58 (0.57-22.11) | + ○ ○ ○ VERY LOW^2,4^ |
| **Antipsychotics** | 1 | 8/41 | - | - |
| Thioridazine | 1 | 8/41 | 1.04 (0.28-3.78) | + ○ ○ ○ VERY LOW^2,4^ |
| **Other unlicensed drugs** | 6 | 248/572 | - | - |
| Modafinil | 5 | 245/547 | 5.51 (3.04-10.32)* | + ○ ○ ○ VERY LOW^2,4^ |
| Carbamazepine | 1 | 3/25 | 0.18 (0.02-1.20) | + ○ ○ ○ VERY LOW^2,4^ |
| **Complementary and alternative medicine interventions** |  |  |  |  |
| **Polyunsaturated fatty acids (or PUFAs)** | 3 | 41/124 | - | - |
| Omega-3 and -3/6 fatty acids | 3 | 41/124 | 1.99 (0.85-4.82) | + ○ ○ ○ VERY LOW^2,4^ |
| **Amino acids** | 1 | 9/58 | - | - |
| L-carnitine | 1 | 9/58 | 1.20 (0.29-5.22) | + ○ ○ ○ VERY LOW^2,4^ |
| **Minerals** | 2 | 49/220 | - | - |
| Zinc | 1 | 45/202 | 2.42 (0.80-7.67) | + ○ ○ ○ VERY LOW^2,4^ |
| Iron | 1 | 4/18 | 2.71x10^10^ (3.34-9.19x10^23^)† | + ○ ○ ○ VERY LOW^2,4^ |
| **Herbal therapy** | 2 | 13/52 | - | - |
| St. John’s wort (*Hypericum perforatum*) | 1 | 11/27 | 1.00 (0.23-4.26) | + ○ ○ ○ VERY LOW^2,4^ |
| Ginkgo biloba | 1 | 2/25 | 0.21 (0.02-1.35) | + ○ ○ ○ VERY LOW^2,4^ |
| **Combined interventions** |  |  |  |  |
| **Stimulants plus behavioural therapy** | 8 | 283/521 | - | - |
| Methylphenidate plus parent training | 1 | 1/4 | 55.63 (3.18-29.52x10^2^)* | + ○ ○ ○ VERY LOW^2,4^ |
| Methylphenidate plus child, parent and/or teacher training | 5 | 187/282 | 15.82 (8.06-32.80)* | + ○ ○ ○ VERY LOW^2,4^ |
| **Stimulants plus non-stimulants** | 4 | 313/480 | - | - |
| Methylphenidate plus clonidine | 1 | 29/33 | 21.91 (5.52-105.4)* | + ○ ○ ○ VERY LOW^2,4^ |
| **Non-stimulants plus behavioural therapy** | 4 | 86/163 | - | - |
| Atomoxetine plus parent training | 1 | 18/50 | 2.48 (0.51-11.79) | + ○ ○ ○ VERY LOW^2,4^ |
| Atomoxetine plus child, parent and/or teacher training | 3 | 68/113 | 5.53 (2.19-14.06)* | + ○ ○ ○ VERY LOW^2,4^ |
| **Stimulants plus antipsychotics** | 2 | 33/54 | - | - |
| Methylphenidate plus thioridazine | 1 | 8/41 | 5.97 (1.90-19.59)* | + ○ ○ ○ VERY LOW^2,4^ |
| **Stimulants plus polyunsaturated fatty acids (or PUFAs)** | 2 | 47/50 | - | - |
| Methylphenidate plus omega-3/6 fatty acids | 2 | 47/50 | 15.68 (3.43-87.0)* | + ○ ○ ○ VERY LOW^2,4^ |
| **Stimulants plus minerals** | 1 | 11/20 | - | - |
| Methylphenidate plus zinc | 1 | 11/20 | 15.73 (2.94-85.29)* | + ○ ○ ○ VERY LOW^2,4^ |
| **Stimulants plus herbal therapy** | 1 | 26/33 | - | - |
| Methylphenidate plus ginkgo biloba | 1 | 26/33 | 8.65 (1.94-40.79)* | + ○ ○ ○ VERY LOW^2,4^ |

Results are odds ratios (OR) with 95% credible Intervals and quality of evidence. An OR < 1 favours placebo (that is, more response events occur with placebo that with other intervention). *p < 0.05. †Extremely wide credible intervals owing to small patient and trial numbers and frequent events among treatment arm.

Reasons for downgrading the quality of evidence for a specific outcome:

1. Rated down 1 level for risk of bias (for example, the majority of the comparisons are at low/unclear risk of bias but the proportion of information from studies at high risk of bias is sufficient to affect the interpretation of results)
2. Rated down 2 levels for risk of bias (for example, very serious limitations for one or more risk of bias criteria and/or the majority of information from studies at high risk of bias sufficient to affect the interpretation of results).
3. Rated down 1 level for imprecision of results (for example, 95% credible intervals are wide and include or are close to null effect, or based on small number of studies/outcome events).
4. Rated down 2 level for imprecision of results (for example, 95% credible intervals include the null effect and/or are extremely wide).
5. Rated down 1 level for unexplained inconsistency of results (such as intransitivity or lack of consistency as per the DIC).

**Network meta-analysis for acceptability. Class effect.**

|  | **Trials** | **Events and participants** | **Class effect,**  **OR (95% CrI)** | **Quality of evidence (GRADE)** |
| --- | --- | --- | --- | --- |
| **Controls** |  |  |  |  |
| **Placebo** | 92 | 1406/5202 | Reference | Reference |
| **Control (e.g., usual care)** | 17 | 79/819 | 0.55 (0.32-0.95)* | + ○ ○ ○ VERY LOW^2,5^ |
| **Waiting list** | 10 | 28/290 | 0.33 (0.15-0.70)* | + ○ ○ ○ VERY LOW^2,5^ |
| **Psychological interventions** |  |  |  |  |
| **Behavioural therapy** | 25 | 224/1385 | 0.58 (0.33-0.99)* | + ○ ○ ○ VERY LOW^1,3,5^ |
| **Cognitive training** | 10 | 49/339 | 1.32 (0.71-2.52) | + ○ ○ ○ VERY LOW^2,4,5^ |
| **Behavioural therapy with cognitive training** | 1 | 3/26 | 3.39 (0.60-19.58) | + ○ ○ ○ VERY LOW^2,4,5^ |
| **Neurofeedback** | 10 | 38/271 | 0.59 (0.31-1.14); | + ○ ○ ○ VERY LOW^2,4,5^ |
| **Pharmacological interventions** |  |  |  |  |
| **Stimulants** | 65 | 847/4778 | 0.67 (0.54-0.83)* | + + ○ ○ LOW^1,5^ |
| **Non-stimulants** | 52 | 1420/5817 | 0.81 (0.67-0.97)* | + + ○ ○ LOW^1,5^ |
| **Antidepressants** | 7 | 23/208 | 0.99 (0.44-2.28) | + ○ ○ ○ VERY LOW^2,4,5^ |
| **Antipsychotics** | 4 | 11/108 | 1.25 (0.43-3.63) | + ○ ○ ○ VERY LOW^2,4,5^ |
| **Other unlicensed drugs** | 7 | 133/585 | 0.73 (0.42-1.29) | + ○ ○ ○ VERY LOW^2,4,5^ |
| **Complementary and alternative medicine interventions** |  |  |  |  |
| **Dietary therapy** | 3 | 13/85 | 0.79 (0.23-2.76) | + ○ ○ ○ VERY LOW^2,4,5^ |
| **Polyunsaturated fatty acids (or PUFAs)** | 9 | 92/442 | 1.09 (0.68-1.74) | + ○ ○ ○ VERY LOW^2,4,5^ |
| **Amino acids** | 4 | 20/121 | 0.59 (0.24-1.44) | + ○ ○ ○ VERY LOW^2,4,5^ |
| **Minerals** | 3 | 111/248 | 1.14 (0.49-2.72) | + ○ ○ ○ VERY LOW^2,4,5^ |
| **Herbal therapy** | 4 | 6/131 | 0.53 (0.15-1.88) | + ○ ○ ○ VERY LOW^2,4,5^ |
| **Homeopathy** | 2 | 10/52 | 0.56 (0.18-1.73) | + ○ ○ ○ VERY LOW^2,4,5^ |
| **Physical activity (exercise)** | 1 | 3/37 | 0.44 (0.07-2.19) | + ○ ○ ○ VERY LOW^2,4,5^ |
| **Combined interventions** |  |  |  |  |
| **Stimulants plus behavioural therapy** | 13 | 109/699 | 0.37 (0.21-0.67)* | + ○ ○ ○ VERY LOW^2,5^ |
| **Stimulants plus non-stimulants** | 7 | 85/529 | 0.47 (0.27-0.81)* | + ○ ○ ○ VERY LOW^2,5^ |
| **Non-stimulants plus behavioural therapy** | 3 | 22/114 | 1.00 (0.37-2.84) | + ○ ○ ○ VERY LOW^2,4,5^ |
| **Stimulants plus antipsychotics plus behavioural therapy** | 1 | 23/84 | 0.65 (0.19-2.37) | + ○ ○ ○ VERY LOW^2,4,5^ |
| **Stimulants plus antipsychotics** | 2 | 4/54 | 0.99 (0.21-4.19) | + ○ ○ ○ VERY LOW^2,4,5^ |
| **Stimulants plus neurofeedback** | 2 | 12/76 | 0.37 (0.13-1.05) | + ○ ○ ○ VERY LOW^2,4,5^ |
| **Stimulants plus dietary therapy** | 1 | 10/53 | 0.59 (0.16-2.21) | + ○ ○ ○ VERY LOW^2,4,5^ |
| **Stimulants plus polyunsaturated fatty acids (or PUFAs)** | 3 | 9/111 | 0.34 (0.12-0.94)* | + ○ ○ ○ VERY LOW^2,4,5^ |
| **Stimulants plus amino acids** | 1 | 1/20 | 0.65 (0.02-34.56) | + ○ ○ ○ VERY LOW^2,4,5^ |
| **Stimulants plus minerals** | 2 | 3/42 | 0.36 (0.05-1.97) | + ○ ○ ○ VERY LOW^2,4,5^ |
| **Stimulants plus herbal therapy** | 1 | 2/33 | 0.28 (0.03-2.08) | + ○ ○ ○ VERY LOW^2,4,5^ |
| **Stimulants plus vitamins** | 1 | 15/23 | 1.49 (0.35-6.67) | + ○ ○ ○ VERY LOW^2,4,5^ |
| **Stimulants plus physical activity** | 2 | 5/33 | 0.50 (0.09-2.76) | + ○ ○ ○ VERY LOW^2,4,5^ |

Results are odds ratios (OR) with 95% credible Intervals and quality of evidence. An OR < 1 favours placebo (that is, more response events occur with placebo that with other intervention). *p < 0.05. †Extremely wide credible intervals owing to small patient and trial numbers and frequent events among treatment arm.

Reasons for downgrading the quality of evidence for a specific outcome:

1. Rated down 1 level for risk of bias (for example, the majority of the comparisons are at low/unclear risk of bias but the proportion of information from studies at high risk of bias is sufficient to affect the interpretation of results)
2. Rated down 2 levels for risk of bias (for example, very serious limitations for one or more risk of bias criteria and/or the majority of information from studies at high risk of bias sufficient to affect the interpretation of results).
3. Rated down 1 level for imprecision of results (for example, 95% credible intervals are wide and include or are close to null effect, or based on small number of studies/outcome events).
4. Rated down 2 level for imprecision of results (for example, 95% credible intervals include the null effect and/or are extremely wide).
5. Rated down 1 level for unexplained inconsistency of results (such as intransitivity or lack of consistency as per the DIC).

**Network meta-analysis for acceptability. Individual effect.**

|  | **Trials** | **Events and participants** | **Individual effect,**  **OR (95% CrI)** | **Quality of evidence (GRADE)** |
| --- | --- | --- | --- | --- |
| **Controls** |  |  |  |  |
| **Placebo** | 92 | 1406/5202 | Reference | Reference |
| **Control (e.g., usual care)** | 17 | 79/819 | 0.55 (0.32-0.98)* | + ○ ○ ○ VERY LOW^2,5^ |
| **Waiting list** | 10 | 28/290 | 0.30 (0.13-0.69)* | + ○ ○ ○ VERY LOW^2,5^ |
| **Psychological interventions** |  |  |  |  |
| **Behavioural therapy** | 25 | 224/1385 | - | - |
| Parent training | 16 | 124/742 | 0.69 (0.35-1.36) | + ○ ○ ○ VERY LOW^1,4,5^ |
| Child, parent and/or teacher training | 9 | 54/569 | 0.37 (0.17-0.80)* | + + ○ ○ LOW^1,5^ |
| Child training | 2 | 48/205 | 0.25 (0.04-1.98) | + ○ ○ ○ VERY LOW^1,4,5^ |
| Teacher training | 1 | 4/28 | 0.76 (0.08-8.49) | + ○ ○ ○ VERY LOW^1,4,5^ |
| **Cognitive training** | 10 | 49/339 | - | - |
| Working memory training | 6 | 27/198 | 1.75 (0.75-4.14); | + ○ ○ ○ VERY LOW^2,4,5^ |
| Attention training | 3 | 19/126 | 0.65 (0.22-1.92) | + ○ ○ ○ VERY LOW^2,4,5^ |
| **Neurofeedback** | 10 | 38/271 | - | - |
| Neurofeedback/theta-beta training | 9 | 34/225 | 0.40 (0.19-0.82)* | + ○ ○ ○ VERY LOW^2,5^ |
| **Pharmacological interventions** |  |  |  |  |
| **Stimulants** | 65 | 847/4778 | - | - |
| Methylphenidate | 55 | 585/3196 | 0.59 (0.46-0.75)* | + + ○ ○ LOW^1,5^ |
| Amphetamine | 8 | 255/1433 | 0.78 (0.52-1.18) | + ○ ○ ○ VERY LOW^1,3,5^ |
| **Non-stimulants** | 52 | 1420/5817 | - | - |
| Atomoxetine | 37 | 857/3844 | 0.85 (0.68-1.07) | + ○ ○ ○ VERY LOW^1,3,5^ |
| Guanfacine | 9 | 439/1449 | 0.79 (0.54-1.14) | + ○ ○ ○ VERY LOW^1,3,5^ |
| Clonidine | 6 | 71/298 | 0.40 (0.20-0.78)* | + ○ ○ ○ VERY LOW^1,3,5^ |
| **Antidepressants** | 7 | 23/208 | - | - |
| Bupropion | 3 | 11/114 | 1.54 (0.39-6.76) | + ○ ○ ○ VERY LOW^2,4,5^ |
| Desipramine | 2 | 6/58 | 0.70 (0.17-2.89) | + ○ ○ ○ VERY LOW^2,4,5^ |
| Venlafaxine | 1 | 1/19 | 0.64 (0.02-19.78) | + ○ ○ ○ VERY LOW^2,4,5^ |
| Reboxetine | 1 | 5/17 | 0.73 (0.12-4.44) | + ○ ○ ○ VERY LOW^2,4,5^ |
| **Antipsychotics** | 4 | 11/108 | - | - |
| Risperidone | 2 | 5/42 | 0.56 (0.13-2.77) | + ○ ○ ○ VERY LOW^2,4,5^ |
| Thioridazine | 1 | 5/41 | 3.01 (0.57-17.81) | + ○ ○ ○ VERY LOW^2,4,5^ |
| Aripiprazole | 1 | 1/25 | 0.61 (0.02-25.34) | + ○ ○ ○ VERY LOW^2,4,5^ |
| **Other unlicensed drugs** | 7 | 133/585 | - | - |
| Modafinil | 6 | 127/560 | 0.67 (0.37-1.24) | + ○ ○ ○ VERY LOW^2,4,5^ |
| Carbamazepine | 1 | 6/25 | 0.69 (0.11-4.27) | + ○ ○ ○ VERY LOW^2,4,5^ |
| **Complementary and alternative medicine interventions** |  |  |  |  |
| **Dietary therapy** | 3 | 13/85 | - | - |
| Elimination diet | 3 | 13/85 | 0.77 (0.23-2.64) | + ○ ○ ○ VERY LOW^2,4,5^ |
| **Polyunsaturated fatty acids (or PUFAs)** | 9 | 92/442 | - | - |
| Omega-3 and -3/6 fatty acids | 9 | 92/442 | 1.06 (0.66-1.71) | + ○ ○ ○ VERY LOW^2,4,5^ |
| **Amino acids** | 4 | 20/121 | - | - |
| L-carnitine | 3 | 19/101 | 0.66 (0.25-1.75) | + ○ ○ ○ VERY LOW^2,4,5^ |
| **Minerals** | 3 | 111/248 | - | - |
| Zinc | 2 | 109/230 | 1.05 (0.42-2.62) | + ○ ○ ○ VERY LOW^2,4,5^ |
| Iron | 1 | 2/18 | 26.79 (0.19-1.03x10^6^)† | + ○ ○ ○ VERY LOW^2,4,5^ |
| **Herbal therapy** | 4 | 6/131 | - | - |
| St. John's wort (*Hypericum perforatum*) | 1 | 1/27 | 0.41 (0.01-6.11) | + ○ ○ ○ VERY LOW^2,4,5^ |
| Ginkgo biloba | 1 | 2/25 | 0.57 (0.05-6.67) | + ○ ○ ○ VERY LOW^2,4,5^ |
| Ginseng | 1 | 0/35 | 5.31x10^-4^ (5.70x10^-12^-0.69)† | + ○ ○ ○ VERY LOW^2,4,5^ |
| Pine bark (extract) | 1 | 3/44 | 1.33 (0.12-44.24) | + ○ ○ ○ VERY LOW^2,4,5^ |
| **Homeopathy** | 2 | 10/52 | 0.56 (0.17-1.77) | + ○ ○ ○ VERY LOW^2,4,5^ |
| **Physical activity (exercise)** | 1 | 3/37 | 0.48 (0.07-2.75) | + ○ ○ ○ VERY LOW^2,4,5^ |
| **Combined interventions** |  |  |  |  |
| **Stimulants plus behavioural therapy** | 13 | 109/699 | - | - |
| Methylphenidate plus parent training | 2 | 19/68 | 0.50 (0.18-1.44) | + ○ ○ ○ VERY LOW^2,4,5^ |
| Methylphenidate plus child training | 3 | 37/185 | 0.18 (0.02-1.74) | + ○ ○ ○ VERY LOW^2,4,5^ |
| Methylphenidate plus child, parent and/or teacher training | 7 | 38/362 | 0.24 (0.12-0.50)* | + ○ ○ ○ VERY LOW^2,5^ |
| **Stimulants plus non-stimulants** | 7 | 85/529 | - | - |
| Methylphenidate plus atomoxetine | 1 | 1/9 | 1.00 (0.03-48.87) | + ○ ○ ○ VERY LOW^2,4,5^ |
| Methylphenidate plus clonidine | 3 | 12/73 | 0.32 (0.13-0.77)* | + ○ ○ ○ VERY LOW^2,3,5^ |
| **Non-stimulants plus behavioural therapy** | 3 | 22/114 | - | - |
| Atomoxetine plus parent training | 1 | 14/50 | 0.87 (0.21-3.74) | + ○ ○ ○ VERY LOW^2,4,5^ |
| Atomoxetine plus child, parent and/or teacher training | 2 | 8/64 | 1.23 (0.29-6.03) | + ○ ○ ○ VERY LOW^2,4,5^ |
| **Stimulants plus antipsychotics** | 2 | 4/54 | - | - |
| Methylphenidate plus thioridazine | 1 | 3/42 | 1.61 (0.25-10.33) | + ○ ○ ○ VERY LOW^2,4,5^ |
| **Stimulants plus neurofeedback** | 2 | 12/76 | - | - |
| Methylphenidate plus neurofeedback | 2 | 12/76 | 0.30 (0.10-0.89)* | + ○ ○ ○ VERY LOW^2,3,5^ |
| **Stimulants plus dietary therapy** | 1 | 10/53 | - | - |
| Methylphenidate plus elimination diet | 1 | 10/53 | 0.52 (0.13-2.00) | + ○ ○ ○ VERY LOW^2,4,5^ |
| **Stimulants plus polyunsaturated fatty acids (or PUFAs)** | 3 | 9/111 | - | - |
| Methylphenidate plus omega-3/6 fatty acids | 3 | 9/111 | 0.31 (0.11-0.84)* | + ○ ○ ○ VERY LOW^2,3,5^ |
| **Stimulants plus aminoacids** | 1 | 1/20 | - | - |
| Methylphenidate plus l-carnitine | 1 | 1/20 | 0.50 (0.01-17.26) | + ○ ○ ○ VERY LOW^2,4,5^ |
| **Stimulants plus minerals** | 2 | 3/42 | - | - |
| Methylphenidate plus zinc | 2 | 3/42 | 0.31 (0.05-1.68) | + ○ ○ ○ VERY LOW^2,4,5^ |
| **Stimulants plus herbal therapy** | 1 | 2/33 | - | - |
| Methylphenidate plus ginkgo biloba | 1 | 2/33 | 0.23 (0.02-1.75) | + ○ ○ ○ VERY LOW^2,4,5^ |
| **Stimulants plus vitamins** | 1 | 15/23 | - | - |
| Methylphenidate plus vitamin B9 | 1 | 15/23 | 1.32 (0.30-5.94) | + ○ ○ ○ VERY LOW^2,4,5^ |
| **Stimulants plus physical activity** | 2 | 5/33 | - | - |
| Methylphenidate plus exercise | 2 | 5/33 | 0.25 (0.02-3.93) | + ○ ○ ○ VERY LOW^2,4,5^ |

Results are odds ratios (OR) with 95% credible Intervals and quality of evidence. An OR < 1 favours placebo (that is, more response events occur with placebo that with other intervention). *p < 0.05. †Extremely wide credible intervals owing to small patient and trial numbers and frequent events among treatment arm.

Reasons for downgrading the quality of evidence for a specific outcome:

1. Rated down 1 level for risk of bias (for example, the majority of the comparisons are at low/unclear risk of bias but the proportion of information from studies at high risk of bias is sufficient to affect the interpretation of results)
2. Rated down 2 levels for risk of bias (for example, very serious limitations for one or more risk of bias criteria and/or the majority of information from studies at high risk of bias sufficient to affect the interpretation of results).
3. Rated down 1 level for imprecision of results (for example, 95% credible intervals are wide and include or are close to null effect, or based on small number of studies/outcome events).
4. Rated down 2 level for imprecision of results (for example, 95% credible intervals include the null effect and/or are extremely wide).
5. Rated down 1 level for unexplained inconsistency of results (such as intransitivity or lack of consistency as per the DIC).
