# Supplementary material for: The pharmacological and non-pharmacological treatment of attention deficit hyperactivity disorder in children and adolescents: A systematic review with network meta-analyses of randomised trials
Source: PLoS One. 2017 Jul 12;12(7):e0180355. doi: 10.1371/journal.pone.0180355 (PMC5507500; doi:10.1371/journal.pone.0180355)
Supplement: S8 Table — (DOCX) [file pone.0180355.s013.docx]

**S8 Table. Additional analyses: Sensitivity analysis for efficacy (treatment response)**

|  | **Base case model** | **Adjusted for publication year** | **Adjusted for study duration (follow-up)** | **Adjusted for mean age** | **Adjusted for % males** | **Adjusted for baseline risk/ placebo response** | **Exclusion of high RoB studies** | **Exclusion of small studies (<100 patients)** | **Exclusion of ‘unblinded’ trials** |
| --- | --- | --- | --- | --- | --- | --- | --- | --- | --- |
| **PBO** | reference | reference | reference | reference | reference | reference | reference | reference | reference |
| **CONT** | 1.99 (0.98-4.15) | 1.95 (0.97-4.02) | 1.99 (0.94-4.32) | 2.08 (1.01-4.40) | 2.04 (0.99-4.32) | 2.04 (0.96-4.35) | - | 1.37 (0.71-2.59) | 56.21 (5.53-708) |
| **WL** | 0.57 (0.20-1.62) | 0.56 (0.20-1.56) | 0.57 (0.19-1.68) | 0.60 (0.21-1.71) | 0.58 (0.20-1.64) | 0.56 (0.19-1.65) | - | 0.06 (0.01-0.36) | 15.32 (1.14-249) |
| **BEHAV** | 2.97 (1.53-5.88) | 2.91 (1.50-5.66) | 2.96 (1.46-6.13) | 3.10 (1.57-6.25) | 3.05 (1.53-6.05) | 2.92 (1.45-5.83) | - | 2.42 (1.21-4.75) | 33.64 (4.97-310) |
| **COG** | 0.70 (0.12-3.87) | 0.76 (0.14-4.17) | 0.69 (0.12-3.94) | 0.68 (0.12-3.65) | 0.70 (0.12-4.09) | 0.20 (0.04-0.88) | 0.33 (0.01-5.24) | 0.61 (0.01-45.54) | 0.35 (0.01-6.52) |
| **NF** | 1.96 (0.52-8.26) | 2.08 (0.54-8.35) | 1.96 (0.49-8.44) | 2.01 (0.51-7.89) | 1.98 (0.49-8.25) | 0.58 (0.16-1.88) | - | 1.38 (0.02-98.00) | - |
| **STI** | 6.21 (4.89-7.96) | 5.99 (4.73-7.64) | 6.18 (4.47-8.81) | 6.49 (5.06-8.38) | 6.34 (4.97-8.17) | 6.51 (5.16-8.22) | 6.28 (4.83-8.41) | 5.35 (4.34-6.61) | 6.15 (4.73-8.06) |
| **N-STI** | 3.95 (3.13-5.07) | 3.98 (3.16-5.07) | 3.97 (3.01-5.33) | 4.17 (3.26-5.42) | 4.06 (3.19-5.23) | 4.60 (3.67-5.79) | 3.59 (2.79-4.75) | 3.36 (2.75-4.13) | 3.91 (3.04-5.12) |
| **AD** | 8.52 (3.95-18.96) | 7.17 (3.28-15.91) | 8.55 (3.73-19.97) | 9.16 (4.22-20.54) | 8.22 (3.76-18.52) | 5.89 (2.86-12.06) | 9.38 (4.11-22.71) | - | 8.42 (3.87-19.04) |
| **A-PSY** | 1.36 (0.34-5.38) | 0.95 (0.23-3.84) | 1.35 (0.33-5.47) | 1.37 (0.34-5.31) | 1.31 (0.31-5.16) | 1.10 (0.28-4.27) | 1.13 (0.32-4.00) | 1.00 (0.00-501) | 1.35 (0.33-5.34) |
| **O-DRU** | 3.80 (2.04-7.14) | 3.72 (2.03-6.90) | 3.82 (1.99-7.48) | 3.85 (2.07-7.27) | 3.97 (2.12-7.58) | 5.96 (2.86-12.06) | 5.59 (3.06-10.55) | 2.69 (1.54-4.64) | 3.81 (2.02-7.23) |
| **STI+BEHAV** | 13.62 (6.83-27.93) | 13.15 (6.76-26.83) | 13.62 (6.67-29.51) | 14.19 (7.13-29.74) | 13.94 (6.94-28.97) | 13.49 (6.63-27.94) | - | 6.06 (2.98-12.20) | 61.30 (12.46-407) |
| **N-STI+BEHAV** | 6.05 (2.39-15.27) | 5.92 (2.41-14.67) | 6.05 (2.35-15.84) | 6.26 (2.51-16.00) | 6.18 (2.45-15.74) | 6.10 (2.29-15.90) | - | 4.67 (1.18-18.32) | 74.27 (8.90-785) |
| **STI+N-STI** | 15.18 (7.50-31.46) | 14.56 (7.34-29.87) | 15.13 (7.22-32.09) | 15.89 (7.82-33.03) | 15.29 (7.54-32.04) | 17.08 (8.17-35.95) | 14.70 (7.88-29.03) | 11.66 (6.29-22.09) | 14.99 (7.27-32.05) |
|  |  |  |  |  |  |  |  |  |  |
| **Residual deviance** | 261.4 | 263.7 | 261.2 | 261.7 | 260.1 | 238.4 | 154.1 | 152.5 | 204.3 |
| **DIC** | 1426.27 | 1427.70 | 1427.18 | 1426.80 | 1425.75 | 1411.97 | 811.10 | 959.27 | 1119.05 |
| **# data points** | 241 | 241 | 241 | 241 | 241 | 241 | 136 | 147 | 186 |

Values represent odds ratios with 95% credible intervals. RoB = risk of bias. DIC = deviance information criteria. SD = standard deviation.

PBO=placebo. CONT=control. WL=waiting list. BEHAV=behavioural therapy. COGN=cognitive training. NF=neurofeedback. STI=stimulants. N-STI=non-stimulants. AD=antidepressants. A-PSY=antipsychotics. O-DRU=other unlicensed drugs. STI+BEHAV=stimulants+behavioural therapy. N-STI+BEHAV=non-stimulants+behavioural therapy. STI+N-STI=stimulants+non-stimulants.

**S8 Table. Additional analyses: Subgroup analyses for treatment response**

|  |  | **Type of rating scale^†^** | | **Type of rater** | | | **Treatment duration** | | |
| --- | --- | --- | --- | --- | --- | --- | --- | --- | --- |
|  | **Base case model** | **ADHD symptoms-based** | **Global functioning-based** | **Clinician** | **Teacher** | **Parents** | **Short-term/acute treatment** | **Mid-term treatment** | **Long-term treatment^††^** |
| **PBO** | reference | reference | reference | reference | reference | reference | reference | reference | - |
| **CONT** | 1.99 (0.98-4.15) | 2.36 (0.97-6.08) | 1.29 (0.47-3.62) | 1.31 (0.40-4.51) | 2.57 (1.06-7.67) | 1.93 (0.66-6.10) | 1.45 (0.53-4.04) | 1.89 (0.37-13.05) | reference |
| **WL** | 0.57 (0.20-1.62) | 1.33 (0.30-6.01) | 0.23 (0.05-0.91) | - | 0.06 (0.00-0.59) | 0.40 (0.10-1.57) | 0.33 (0.07-1.45) | 0.64 (0.08-6.41) | - |
| **BEHAV** | 2.97 (1.53-5.88) | 3.09 (1.21-8.09) | 2.99 (1.21-7.31) | 2.41 (0.90-6.46) | 4.40 (1.95-12.57) | 3.19 (1.18-9.04) | 2.37 (0.92-6.05) | 2.36 (0.48-14.54) | 1.50 (0.02-140.2) |
| **COG** | 0.70 (0.12-3.87) | 0.23 (0.01-3.73) | 0.39 (0.01-5.80) | 0.33 (0.01-5.64) | - | 0.21 (0.01-3.33) | 1.40 (0.17-15.38) | 0.34 (0.02-9.15) | - |
| **NF** | 1.96 (0.52-8.26) | 0.56 (0.04-5.16) | - | - | 0.68 (0.05-5.37) | 0.49 (0.04-4.65) | 5.06 (0.62-64.39) | 0.81 (0.07-10.97) | - |
| **STI** | 6.21 (4.89-7.96) | 6.20 (4.22-9.34) | 5.72 (4.63-7.10) | 5.72 (4.48-7.40) | 7.12 (4.54-12.03) | 5.26 (3.49-8.25) | 6.25 (4.88-8.13) | 5.07 (1.66-18.04) | 3.92 (0.07-263.2) |
| **N-STI** | 3.95 (3.13-5.07) | 3.96 (2.90-5.57) | 3.22 (2.60-4.06) | 3.30 (2.57-4.32) | 3.45 (1.95-6.91) | 4.10 (2.80-6.05) | 4.06 (3.17-5.30) | 2.80 (1.06-8.17) | - |
| **AD** | 8.52 (3.95-18.96) | 6.16 (2.58-15.38) | 25.80 (8.46-96.25) | 26.5 (8.28-102.3) | 4.18 (1.20-14.64) | 6.56 (2.64-17.2) | 8.56 (3.95-19.10) | - | - |
| **A-PSY** | 1.36 (0.34-5.38) | - | 1.24 (0.38-3.85) | 1.26 (0.34-4.49) | 5.28 (1.55-18.73) | 2.32 (0.56-10.24) | 1.35 (0.34-5.29) | - | - |
| **O-DRU** | 3.80 (2.04-7.14) | 4.11 (1.39-12.59) | 3.70 (2.00-6.92) | 2.28 (1.17-4.32) | 20.97 (6.11-88.36) | 21.13 (5.24-99.61) | 3.83 (2.08-7.24) | - | - |
| **STI+BEHAV** | 13.62 (6.83-27.93) | 17.64 (7.31-45.54) | 7.47 (3.08-18.87) | 7.03 (2.70-19.84) | 23.95 (10.63-72.3) | 17.52 (6.87-51.28) | 41.96 (8.42-286.5) | 8.10 (1.89-46.00) | 6.12 (0.10-416.7) |
| **N-STI+BEHAV** | 6.05 (2.39-15.27) | 20.87 (3.42-131.5) | 3.72 (1.39-9.76) | 4.97 (1.75-14.06) | - | - | 5.09 (1.79-14.44) | - | - |
| **STI+N-STI** | 15.18 (7.50-31.46) | 13.33 (5.23-34.95) | 12.42 (6.06-26.73) | 12.88 (6.42-27.07) | 21.55 (5.47-104.9) | 11.72 (4.61-31.18) | 13.70 (6.15-31.36) | 19.20 (3.09-140.9) | - |
|  |  |  |  |  |  |  |  |  |  |
| **Residual deviance** | 261.4 | 157.3 | 152.8 | 158.4 | 75.04 | 136.5 | 216.1 | 52.87 | 5.78 |
| **DIC** | 1426.27 | 875.27 | 875.74 | 915.45 | 343.45 | 723.72 | 1179.95 | 290.56 | 40.47 |
| **# data points** | 241 | 146 | 145 | 150 | 73 | 126 | 191 | 49 | 6 |

Values represent odds ratios with 95% credible intervals.

RoB = risk of bias. DIC = deviance information criteria. SD = standard deviation.

PBO=placebo. CONT=control. WL=waiting list. BEHAV=behavioural therapy. COGN=cognitive training. NF=neurofeedback. STI=stimulants. N-STI=non-stimulants. AD=antidepressants. A-PSY=antipsychotics. O-DRU=other unlicensed drugs. STI+BEHAV=stimulants+behavioural therapy. N-STI+BEHAV=non-stimulants+behavioural therapy. STI+N-STI=stimulants+non-stimulants.

**^†^**Type of rating scale: ADHD symptoms-based rating scale such as the ADHD Rating Scale (ADHD-RS), the Swanson, Nolan and Pelham (SNAP) Rating Scale and the Conner’s Rating Scale; Global-functioning-based rating scale such as the Clinical Global Impression (GGI) improvement scale and severity scale.^††^For long-term effects, reference comparator was control. No placebo controlled trial were available for this subgroup.

**S8 Table. Additional analyses: Sensitivity analysis for acceptability (all-cause discontinuation)**

|  | **Base case model** | **Adjusted for publication year** | **Adjusted for study duration (follow-up)** | **Adjusted for mean age** | **Adjusted for % males** | **Adjusted for baseline risk/ discontinuations** | **Exclusion of high RoB studies** | **Exclusion of small studies (<100 patients)** | **Exclusion of ‘unblinded’ trials** |
| --- | --- | --- | --- | --- | --- | --- | --- | --- | --- |
| **PBO** | reference | reference | reference | reference | reference | reference | reference | reference | reference |
| **CONT** | 0.55 (0.32-0.95) | 0.55 (0.32-0.93) | 0.52 (0.31-0.89) | 0.53 (0.32-0.92) | 0.53 (0.31-0.91) | 0.55 (0.35-0.87) | 0.47 (0.16-1.38) | 0.43 (0.19-0.99) | 0.41 (0.15-1.06) |
| **WL** | 0.33 (0.15-0.70) | 0.33 (0.16-0.71) | 0.32 (0.15-0.67) | 0.33 (0.15-0.68) | 0.33 (0.16-0.68) | 0.33 (0.17-0.64) | 0.20 (0.03-1.15) | 0.16 (0.04-0.60) | 1.18 (0.01-99.92) |
| **BEHAV** | 0.58 (0.33-0.99) | 0.58 (0.34-1.01) | 0.55 (0.32-0.94) | 0.56 (0.33-0.97) | 0.56 (0.33-0.97) | 0.59 (0.36-0.93) | 1.37 (0.32-5.98) | 0.31 (0.13-0.72) | 0.91 (0.39-2.18) |
| **COG** | 1.32 (0.71-2.52) | 1.33 (0.70-2.58) | 1.24 (0.65-2.39) | 1.33 (0.71-2.53) | 1.26 (0.68-2.44) | 1.08 (0.63-1.85) | 2.15 (0.80-6.05) | 0.70 (0.24-2.18) | 2.15 (0.91-5.37) |
| **NF** | 0.59 (0.31-1.14) | 0.59 (0.31-1.13) | 0.55 (0.29-1.07) | 0.57 (0.30-1.09) | 0.58 (0.30-1.12) | 0.59 (0.33-1.04) | 0.48 (0.13-1.70) | 0.42 (0.17-1.06) | 0.30 (0.07-1.16) |
| **STI** | 0.67 (0.54-0.83) | 0.67 (0.54-0.83) | 0.63 (0.50-0.79) | 0.64 (0.51-0.79) | 0.66 (0.53-0.81) | 0.72 (0.62-0.85) | 0.68 (0.50-0.92) | 0.63 (0.49-0.80) | 0.67 (0.53-0.84) |
| **N-STI** | 0.81 (0.67-0.97) | 0.81 (0.67-0.97) | 0.78 (0.65-0.94) | 0.76 (0.63-0.93) | 0.80 (0.68-0.97) | 0.95 (0.82-1.08) | 0.85 (0.65-1.13) | 0.80 (0.66-0.98) | 0.78 (0.65-0.95) |
| **AD** | 0.99 (0.44-2.28) | 0.98 (0.42-2.33) | 0.93 (0.41-2.09) | 0.95 (0.43-2.17) | 1.30 (0.50-2.58) | 0.76 (0.40-1.45) | 0.80 (0.24-2.65) | 2.43 (0.40-21.21) | 1.00 (0.44-2.29) |
| **A-PSY** | 1.25 (0.43-3.63) | 1.23 (0.42-3.64) | 1.15 (0.39-3.39) | 1.19 (0.40-3.46) | 1.22 (0.43-3.57) | 0.98 (0.38-2.46) | 3.22 (0.57-22.61) | 3.21 (0.55-20.91) | 1.25 (0.37-4.27) |
| **O-DRU** | 0.73 (0.42-1.29) | 0.73 (0.42-1.28) | 0.68 (0.39-1.21) | 0.72 (0.42-1.26) | 0.70 (0.40-1.23) | 0.93 (0.61-1.41) | 0.62 (0.33-1.22) | 0.66 (0.34-1.31) | 0.73 (0.42-1.28) |
| **STI+BEHAV** | 0.37 (0.21-0.67) | 0.38 (0.21-0.68) | 0.36 (0.20-0.64) | 0.36 (0.21-0.65) | 0.37 (0.21-0.65) | 0.39 (0.23-0.64) | 0.98 (0.15-6.17) | 0.27 (0.11-0.65) | 0.51 (0.21-1.21) |
| **N-STI+BEHAV** | 1.00 (0.37-2.84) | 0.99 (0.36-2.85) | 0.95 (0.35-2.69) | 0.96 (0.35-2.69) | 0.98 (0.36-2.67) | 1.03 (0.41-2.58) | 1.78 (0.24-13.10) | 0.39 (0.09-1.89) | 1.24 (0.32-4.87) |
| **STI+N-STI** | 0.47 (0.27-0.81) | 0.47 (0.27-0.82) | 0.45 (0.26-0.79) | 0.45 (0.25-0.79) | 0.47 (0.27-0.81) | 0.54 (0.34-0.86) | 0.49 (0.25-0.93) | 0.48 (0.25-0.89) | 0.47 (0.27-0.81) |
|  |  |  |  |  |  |  |  |  |  |
| **Residual deviance** | 371.5 | 371.7 | 372.5 | 372.2 | 374.1 | 381.5 | 138.31 | 196.0 | 272.6 |
| **DIC** | 1855.73 | 1856.75 | 1855.99 | 1855.55 | 1856.56 | 1855.82 | 941.88 | 1097.20 | 1403.01 |
| **# data points** | 365 | 365 | 365 | 365 | 365 | 365 | 181 | 185 | 270 |

Values represent odds ratios with 95% credible intervals. RoB = risk of bias. DIC = deviance information criteria. SD = standard deviation.

PBO=placebo. CONT=control. WL=waiting list. BEHAV=behavioural therapy. COGN=cognitive training. NF=neurofeedback. STI=stimulants. N-STI=non-stimulants. AD=antidepressants. A-PSY=antipsychotics. O-DRU=other unlicensed drugs. STI+BEHAV=stimulants+behavioural therapy. N-STI+BEHAV=non-stimulants+behavioural therapy. STI+N-STI=stimulants+non-stimulants.

**S8 Table. Additional analyses: Network meta-analyses for secondary outcome of tolerability**

| **PBO** |  |  |  |  |  |  |  |  |  |  |  |  |  |  |  |  |  |
| --- | --- | --- | --- | --- | --- | --- | --- | --- | --- | --- | --- | --- | --- | --- | --- | --- | --- |
| 1.26  (0.17-8.31) | **CONT** |  |  |  |  |  |  |  |  |  |  |  |  |  |  |  |  |
| 15.20 (0.02-100.2E+3) | 12.97 (0.02-96.54E+3) | **WL** |  |  |  |  |  |  |  |  |  |  |  |  |  |  |  |
| 0.43  (0.02-7.87) | 0.35  (0.01-11.76) | 0.03 (2.53E-6-11.46) | **BEHAV** |  |  |  |  |  |  |  |  |  |  |  |  |  |  |
| 0.00  (1.27E-7-1.28) | 0.00  (7.18E-8-1.58) | 1.80E-4 (1.32E-11-1.78) | 0.01 (1.65E-7-7.19) | **NF** |  |  |  |  |  |  |  |  |  |  |  |  |  |
| **2.38**  **(1.45-3.99)** | 1.91  (0.28-14.48) | 0.16 (2.22E-5-102.8) | 5.55  (0.30-105.9) | 500.4 (1.83-1.91E+7) | **STI** |  |  |  |  |  |  |  |  |  |  |  |  |
| **3.11**  **(1.99-5.08)** | 2.46  (0.38-19.31) | 0.20 (2.83E-5-129.2) | 7.27  (0.40-145.4) | 647.5 (2.39-2.40E+7) | 1.31  (0.77-2.21) | **N-STI** |  |  |  |  |  |  |  |  |  |  |  |
| 2.86  (0.51-19.36) | 2.33  (0.18-34.43) | 0.19 (2.17E-5-136.3) | 6.74  (0.22-196.9) | 643.4 (1.47-2.97E+7) | 1.20  (0.21-8.22) | 0.92  (0.16-6.31) | **AD** |  |  |  |  |  |  |  |  |  |  |
| 3.48  (0.63-20.12) | 2.82  (0.22-35.66) | 0.22 (2.42E-5-189.3) | 8.28  (0.28-223.5) | 792.4 (1.76-2.99E+7) | 1.46  (0.26-8.29) | 1.11  (0.19-6.55) | 1.20  (0.09-14.27) | **A-PSY** |  |  |  |  |  |  |  |  |  |
| 1.72  (0.45-7.43) | 1.38  (0.13-16.14) | 0.11  (1.44E-5-86.9) | 4.00  (0.16-104.0) | 374.8 (1.10-1.39E+7) | 0.72  (0.17-3.40) | 0.55  (0.13-2.47) | 0.60  (0.06-5.55) | 0.50  (0.05-4.62) | **O-DRU** |  |  |  |  |  |  |  |  |
| 609.8  (0.81-9.95E+6) | 465.2  (0.72-7.68+6) | 25.6 (0.11-84.65E+3) | 1376 (1.52-4.40E+7) | 199.10E+3 (16.41-2.27E+11) | 254.6 (0.34-4.12E+6) | 195.3 (0.26-3.04E+6) | 199.6 (0.15-62.4E+4) | 164.6 (0.18-3.19E+6) | 355.0 (0.41-6.19E+6) | **DIET** |  |  |  |  |  |  |  |
| 1.30  (0.38-4.85) | 1.04  (0.14-9.22) | 0.09  (1.21E-5-55.32) | 3.08  (0.13-72.95) | 282.1  (0.85-1.02E+7) | 0.55  (0.15-2.09) | 0.42  (0.11-1.60) | 0.45  (0.05-3.78) | 0.38  (0.05-3.19) | 0.76 (0.11-4.75) | 0.00 (1.23E-7-1.73) | **PUFA** |  |  |  |  |  |  |
| 146.2  (0.20-1.69E+7) | 125.1  (0.12-1.32E+7) | 8.09 (1.40E-4-1.01E+7) | 341.3 (0.22-5.75E+7) | 582.1E+2  (4.68-2.18E+11) | 60.97 (0.08-6.84E+6) | 46.66 (0.06-5.22E+4) | 23.34  (0.05-47.32E+4) | 43.09  (0.04-5.24E+6) | 84.62 (0.09-1.08E+7) | 0.26 (1.9E-6-252.1E+3) | 111.3  (0.13-1.140E+7) | **AMIN** |  |  |  |  |  |
| 1.46  (0.30-7.60) | 1.18  (0.10-15.46) | 0.10  (1.063E-5-74.18) | 3.40  (0.12-96.31) | 322.9  (0.87-1.45E+7) | 0.62  (0.11-3.36) | 0.47  (0.09-2.51) | 0.51  (0.04-5.33) | 0.42  (0.04-4.27) | 0.86  (0.10-7.13) | 0.00 (1.18E-7-2.27) | 1.13  (0.14-8.51) | 0.01 (7.24E-8-9.47) | **MIN** |  |  |  |  |
| 7.70E-4 (1.19E-10-3.28) | 6.01E-4 (8.76E-11-3.33) | 1.75E-5 (3.37E-12-2.66) | 0.002 (2.34E-10-13.81) | 0.23 (1.02E-8-266.90E+3) | 3.26E-4 (4.83E-11-1.33) | 2.46E-4 (3.66E-10-1.57) | 2.61E-4  (4.19E-11-1.37) | 2.15E-4  (3.18E-11-1.27) | 4.11E-4 (6.80E-10-2.03) | 6.35E-7(1.73E-14-0.13) | 5.94E-4 (8.29E-11-2.77) | 2.16E-6 (6.09E-15-0.46) | 4.89E-4 (7.42E-11-2.82) | **HERB** |  |  |  |
| 3.10  (0.16-72.06) | 2.51  (0.08-113.1) | 0.20  (1.67E-5-143.1) | 6.88  (0.69-102.8) | 754.4  (1.01-4.37E+7) | 1.30  (0.07-29.42) | 0.95  (0.05-20.4) | 1.04  (0.03-41.92) | 0.88  (0.03-32.3) | 1.80  (0.07-53.02) | 0.00 (1.61E-7-6.53) | 2.33  (0.09-69.67) | 0.02 (1.30E-7-32.34) | 2.09  (0.07-72.63) | 42.8E+2 (0.52-3.76E+10) | **STI+**  **BEHAV** |  |  |
| 0.12  (0.00-3.59) | 0.09  (9.91E-4-4.75) | 0.01  (4.17E-7-4.57) | 0.27  (0.01-3.57) | 25.41  (0.02-6.07E+7) | 0.05  (7.52E-4-1.54) | 0.03 (4.20E-4-1.05) | 0.04 (4.40E-4-1.89) | 0.03 (4.01E-4-1.59) | 0.07 (8.92E-4-2.68) | 1.77E-4 (2.94E-9-0.29) | 0.09  (0.00-3.19) | 7.40E-4 (3.72E-9-1.79) | 0.08 (9.45E-4-3.47) | 145.9 (0.01-1.03E+9) | 0.04 (7.36E-4-1.13) | **N-STI+ BEHAV** |  |
| **4.54**  **(1.10-20.41)** | 3.66  (0.34-42.78) | 0.28 (3.74E-5-219.8) | 10.48  (0.41-282.5) | 972.8  (2.79-3.83E+7) | 1.90  (0.48-7.93) | 1.47  (0.38-6.25) | 1.57  (0.16-15.02) | 1.31  (0.14-11.88) | 2.61  (0.37-18.88) | 0.01 (4.85E-7-6.95) | 3.50  (0.51-23.12) | 0.03 (2.53E-7-27.15) | 3.13  (0.36-27.59) | 60.4E+2 (1.25-4.03E+10) | 1.49  (0.05-37.47) | 39.22  (1.00-3351) | **STI+N-STI** |

AEs = adverse events. PBO=placebo. CONT=control. WL=waiting list. BEHAV=behavioural therapy. NF=neurofeedback. STI=stimulants. N-STI=non-stimulants. AD=antidepressants. A-PSY=antipsychotics. O-DRU=other unlicensed drugs. STI+BEHAV=stimulants+behavioural therapy. N-STI+BEHAV=non-stimulants+behavioural therapy. STI+N-STI=stimulants+non-stimulants.

**S8 Table. Additional analyses: Network meta-analyses for secondary outcome of serious adverse events**

| **PBO** |  |  |  |  |  |  |  |
| --- | --- | --- | --- | --- | --- | --- | --- |
| 0.09  (2.36E-11-1.30E+10) | **BEHAV** |  |  |  |  |  |  |
| 1.15  (0.41-3.31) | 13.92  (1.04E-10-5.85E+10) | **STI** |  |  |  |  |  |
| 1.38  (0.74-2.98) | 15.72  (1.22E-10-9.10E+10) | 1.29  (0.46-3.32) | **N-STI** |  |  |  |  |
| 1.41E+9  (17.56-1.14E+16) | 5.83E+12  (3.52E-8-1.51E+12) | 1.31E+9  (16.54-7.35E+15) | 1.11E+9  (13.16-6.99E+15) | **O-DRU** |  |  |  |
| 0.04  (6.96E-12-6.94E+9) | 0.55  (0.08-3.38) | 0.03  (5.86E-12-2.29E+9) | 0.03  (4.42E-12-4.85E+9) | 9.59E-14  (4.15E-21-1.52E+7) | **STI+**  **BEHAV** |  |  |
| 0.05  (1.38E-11-2.24E+10) | 1.11  (0.03-41.56) | 0.04  (1.17E-11-2.45E+10) | 0.03  (9.91E-12-1.33E+10) | 2.65E-13  (8.91E-21-4.01E+7) | 1.81  (0.03-105) | **N-STI+ BEHAV** |  |
| 5.82  (0.26-177) | 58.16  (7.26E-10-8.41E+11) | 4.42  (0.24-274) | 3.88  (0.18-142) | 2.38E-9  (1.03E-15-0.19) | 117  (1.26E-9-2.79E+12) | 113  (1.45E-10-9.50E+11) | **STI+N-STI** |

PBO=placebo. BEHAV=behavioural therapy. STI=stimulants. N-STI=non-stimulants. O-DRU=other unlicensed drugs. STI+BEHAV=stimulants+behavioural therapy. N-STI+BEHAV=non-stimulants+behavioural therapy. STI+N-STI=stimulants+non-stimulants.

**S8 Table. Additional analyses: Network meta-analyses for secondary outcome of anorexia**

| **PBO** |  |  |  |  |  |  |  |  |  |  |  |  |  |
| --- | --- | --- | --- | --- | --- | --- | --- | --- | --- | --- | --- | --- | --- |
| 3.70  (0.66-21.20) | **CONT** |  |  |  |  |  |  |  |  |  |  |  |  |
| 0.44  (3.64E-7-43.41E+4) | 0.11  (8.71E-8-12.88E+4) | **BEHAV** |  |  |  |  |  |  |  |  |  |  |  |
| **8.01**  **(5.75-11.34)** | 2.17  (0.38-12.45) | 18.50  (1.82E-5-2.24E+7) | **STI** |  |  |  |  |  |  |  |  |  |  |
| **4.70**  **(3.41-6.62)** | 1.27  (0.23-7.06) | 10.86  (1.08E-5-1.27E+7) | **0.59**  **(0.41-0.83)** | **N-STI** |  |  |  |  |  |  |  |  |  |
| **4.01**  **(1.63-10.17)** | 1.09  (0.16-7.65) | 9.44  (9.10E-6-1.15E+7) | 0.50  (0.20-1.23) | 0.85  (0.34-2.20) | **AD** |  |  |  |  |  |  |  |  |
| 1.06  (0.26-4.08) | 0.28  (0.03-2.58) | 2.37  (2.31E-6-3.05E+6) | **0.13**  **(0.03-0.48)** | **0.22**  **(0.05-0.87)** | 0.26  (0.05-1.27) | **A-PSY** |  |  |  |  |  |  |  |
| **3.64**  **(1.59-8.67)** | 0.99  (0.14-6.79) | 8.44  (8.17E-6-1.07E-7) | 0.45  (0.19-1.11) | 0.77  (0.32-1.92) | 0.91  (0.26-3.11) | 3.47  (0.73-17.40) | **O-DRU** |  |  |  |  |  |  |
| 0.98  (0.23-4.16) | 0.26  (0.03-2.52) | 2.30  (2.06E-6-2.64E+6) | **0.12**  **(0.03-0.54)** | **0.21**  **(0.05-0.92)** | 0.25  (0.04-1.34) | 0.94  (0.13-6.88) | 0.27  (0.05-1.44) | **PUFA** |  |  |  |  |  |
| 1.70  (0.26-12.30) | 0.46  (0.03-6.35) | 3.92  (3.44E-5.42E+6) | 0.21  (0.03-1.58) | 0.36  (0.05-2.63) | 0.42  (0.05-3.67) | 1.64  (0.16-17.80) | 0.46  (0.06-3.98) | 1.74  (0.16-19.75) | **MIN** |  |  |  |  |
| 0.56  (0.08-3.66) | 0.15  (0.01-1.92) | 1.26  (1.15E-6-1.68E+6) | **0.07**  **(0.01-0.43)** | 0.12  (0.02-0.78) | 0.14  (0.02-1.08) | 0.53  (0.05-5.30) | 0.15  (0.02-1.19) | 0.57  (0.05-6.11) | 0.33  (0.02-4.65) | **HERB** |  |  |  |
| 1.08  (4.11E-7-6.03E+5) | 0.28  (1.13E-7-1.8E+5) | 2.19  (5.31E-9-6.21E+8) | 0.13  (5.09E-8-7.46E+4) | 0.22  (8.70E-7-12.82E+4) | 0.27  (1.05E-7-14.91E+4) | 1.01  (3.83E-7-6.54E+5) | 0.29  (1.07E-7-1.69E+5) | 1.10  (4.06E-7-6.55E+5) | 0.63  (2.20E-7-4.32E+5) | 1.95  (6.87E-7-1.24E+6) | **STI+**  **BEHAV** |  |  |
| 2.75  (2.32E-6-2.78E+6) | 0.71  (5.93E-7-8.3E+5) | **6.30**  **(2.25-18.54)** | 0.34  (2.93E-7-3.50E+5) | 0.58  (5.14E-7-70.21E+4) | 0.68  (5.61E-7-70.09E+4) | 2.69  (2.01E-6-2.75+6) | 0.75  (6.18E-7-7.74E+5) | 2.82  (2.26E-6-2.95E+6) | 1.60  (1.21E-6-1.87E+6) | 5.00  (3.76E-6-5.68E+6) | 2.84 (1.07E-8-1.19E+9) | **N-STI+ BEHAV** |  |
| **6.25**  **(1.97-19.11)** | 1.70  (0.21-12.69) | 14.40  (1.40E-5-1.80E+7) | 0.78  (0.25-2.29) | 1.33  (0.41-4.02) | 1.55  (0.37-6.26) | **5.91**  **(1.05-33.91)** | 1.71  (0.42-6.85) | 6.39  (0.99-39.79) | 3.66  (0.37-33.71) | **11.20**  **(1.26-100.4)** | 5.92  (9.5E-6-1.54E+7) | 2.24  (2.22E-6-2.73E+6) | **STI+N-STI** |

PBO=placebo. BEHAV=behavioural therapy. STI=stimulants. N-STI=non-stimulants. O-DRU=other unlicensed drugs. STI+BEHAV=stimulants+behavioural therapy. N-STI+BEHAV=non-stimulants+behavioural therapy. STI+N-STI=stimulants+non-stimulants.

**S8 Table. Additional analyses: Network meta-analyses for secondary outcome of decreased weight**

| **PBO** |  |  |  |  |  |  |  |  |  |
| --- | --- | --- | --- | --- | --- | --- | --- | --- | --- |
| **11.44**  **(2.24-59.61)** | **CONT** |  |  |  |  |  |  |  |  |
| 1.22  (1.17E-6-73.16E+4) | 0.11  (8.69E-8-71.96E+4) | **BEHAV** |  |  |  |  |  |  |  |
| **22.52**  **(12.07-43.08)** | 1.97  (0.38-10.10) | 18.32  (3.13E-5-2.04E+7) | **STI** |  |  |  |  |  |  |
| **10.50**  **(6.15-18.72)** | 0.92  (0.20-4.26) | 8.74  (1.39E-5-9.64E+6) | **0.47**  **(0.26-0.84)** | **N-STI** |  |  |  |  |  |
| 139.4  (0.78-1.19E+7) | 12.45  (0.05-1.18E+6) | 196.9  (5.88E-5-4.33E+9) | 5.98  (0.03-5.46E+5) | 13.09  (0.08-1.14E+6) | **AD** |  |  |  |  |
| **7.10**  **(1.75-36.58)** | 0.63  (0.08-5.70) | 6.09  (9.58E-6-6.76E+6) | 0.32  (0.08-1.49) | 0.68  (0.16-3.44) | 0.05  (5.96E-7-13.26) | **O-DRU** |  |  |  |
| 2.93  (0.34-19.50) | 0.26  (0.02-2.93) | 2.31  (3.42E-6-3.14E+6) | **0.13**  **(0.02-0.76)** | 0.28  (0.03-1.80) | 0.02  (2.04E-7-5.33) | 0.40  (0.03-4.07) | **HERB** |  |  |
| **28.35**  **(4.35-186.7)** | 0.11  (8.37E-8-82.18E+4) | 1.03  (0.08-12.86) | 0.05  (5.01E-8-38.77E+4) | 0.11  (1.04-81.59E+4) | 0.01  (2.41E-10-17.06E+4) | 0.16  (1.45E-7-11.87E+4) | 0.42  (3.19E-7-33.12E+4) | **N-STI+ BEHAV** |  |

PBO=placebo. CONT=control. BEHAV=behavioural therapy. STI=stimulants. N-STI=non-stimulants. O-DRU=other unlicensed drugs. HERB=herbal therapy. N-STI+BEHAV=non-stimulants+behavioural therapy.

**S8 Table. Additional analyses: Network meta-analyses for secondary outcome of insomnia**

| **PBO** |  |  |  |  |  |  |  |  |  |  |
| --- | --- | --- | --- | --- | --- | --- | --- | --- | --- | --- |
| 1.14  (2.94E-6-3.44E+6) | **BEHAV** |  |  |  |  |  |  |  |  |  |
| **3.99**  **(2.90-5.55)** | 3.51  (1.12E-6-1.33E+6) | **STI** |  |  |  |  |  |  |  |  |
| **1.55**  **(1.12-2.19)** | 1.35  (4.47E-7-5.13E+5) | **0.39**  **(0.28-0.54)** | **N-STI** |  |  |  |  |  |  |  |
| 1.04  (0.37-3.04) | 0.93  (2.87E-7-2.97E+5) | **0.26**  **(0.10-0.71)** | 0.68  (0.24-1.90) | **AD** |  |  |  |  |  |  |
| **7.18**  **(3.20-18.27)** | 6.16  (1.83E-6-2.83E+6) | 1.81  (0.75-4.88) | **4.67**  **(1.87-12.41)** | **7.05**  **(1.85-27.70)** | **O-DRU** |  |  |  |  |  |
| 0.47  (0.18-1.13) | 0.41  (1.18E-7-1.60E+5) | 0.12  (0.04-0.29) | **0.30**  **(0.11-0.75)** | 0.44  (0.11-1.73) | **0.06**  **(0.02-0.22)** | **PUFA** |  |  |  |  |
| 1.15  (0.18-5.79) | 0.92  (1.93E-7-4.15E+5) | 0.29  (0.04-1.38) | 0.74  (0.11-3.66) | 1.08  (0.13-7.13) | **0.16**  **(0.02-0.93)** | 2.47  (0.32-16.34) | **HERB** |  |  |  |
| 0.26  (5.50E-7-1.85E+6) | 0.25  (0.01-2.84) | 0.06  (1.96E-7-4.57E+5) | 0.17  (4.97E-7-1.14E+6) | 0.26  (7.92E-7-1.65E+6) | 0.04  (1.02E-7-25.49E+4) | 0.57  (1.46E-6-3.59E+6) | 0.26  (5.78E-7-1.60E+6) | **N-STI+ BEHAV** |  |  |
| **3.81**  **(1.70-8.23)** | 3.40  (9.72E-7-1.23E+6) | 0.95  (0.44-1.93) | **2.44**  **(1.09-5.18)** | 3.68  (1.03-11.69) | 0.52  (0.15-1.58) | **8.21**  **(2.50-27.0)** | 3.35  (0.57-23.72) | 14.53  (1.87E-6-4.74E+6) | **STI+N-STI** |  |

PBO=placebo. BEHAV=behavioural therapy. STI=stimulants. N-STI=non-stimulants. AD=antidepressants. O-DRU=other unlicensed drugs. PUFA=polyunsaturated fatty acids. HERB=herbal therapy. N-STI+BEHAV=non-stimulants+behavioural therapy. STI+N-STI=stimulants+non-stimulants.

**S8 Table. Additional analyses: Network meta-analyses for secondary outcome of sleep disturbances**

| **PBO** |  |  |  |  |  |  |  |  |  |  |  |
| --- | --- | --- | --- | --- | --- | --- | --- | --- | --- | --- | --- |
| 0.85  (2.96E-6-1.13E+6) | **BEHAV** |  |  |  |  |  |  |  |  |  |  |
| **6.02**  **(2.81-14.45)** | 7.12  (5.88E-6-2.15E+6) | **STI** |  |  |  |  |  |  |  |  |  |
| 2.81  (0.67-7.78) | 2.90  (2.57E-6-86.22E+4) | 0.49  (0.11-1.07) | **N-STI** |  |  |  |  |  |  |  |  |
| 4.50  (0.85-31.99) | 5.65  (3.61E-6-1.88E+6) | 0.75  (0.11-6.20) | 1.61  (0.24-19.24) | **AD** |  |  |  |  |  |  |  |
| **0.15**  **(0.02-0.89)** | 0.16  (8.33E-8-58.97E+3) | **0.02**  **(0.00-0.13)** | **0.05**  **(0.00-0.47)** | **0.03**  **(0.00-0.38)** | **A-PSY** |  |  |  |  |  |  |
| 1.23  (0.31-5.05) | 1.33  (1.11E-6-43.36E+4) | **0.20**  **(0.04-0.85)** | 0.44  (0.09-3.28) | 0.27  (0.02-2.36) | 8.74  (0.86-114.3) | **O-DRU** |  |  |  |  |  |
| 42.49  (0.21-6.56E+6) | 118  (1.67E-5-2.90E+9) | 6.93  (0.03-1.19E+6) | 16.35  (0.05-2.91E+6) | 9.31  (0.02-1.33E+6) | 337.2  (1.10-5.30E+7) | 35.17  (0.13-8.43E+6) | **PUFA** |  |  |  |  |
| 0.16  (0.00-2.17) | 0.16  (1.04E-7-66.20E+3) | 003  (6.69E-4-1.19E+6) | 0.06  (0.00-1.16) | 0.04  (6.03E-4-0.75) | 1.13  (0.02-33.4) | 0.13  (0.00-2.54) | 0.00  (1.44E-8-2.29) | **MIN** |  |  |  |
| 0.78  (3.57E-7-10.64E+4) | 1.60  (1.31E-6-53.48E+4) | 0.13  (5.92E-8-17.63E+3) | 0.27  (1.19E-7-47.06E+3) | 0.16  (6.94E-8-29.41E+3) | 6.10  (2.08E-6-96.55E+4) | 0.65  (2.49E-7-98.37E+3) | 0.01  (1.31E-10-82.62E+2) | 5.30  (1.57E-6-1.02E+6) | **STI+**  **BEHAV** |  |  |
| 0.52 (1.70E-6-72.46E+4) | 0.58  (0.09-4.10) | 0.08  (2.88E-7-11.92E+4) | 0.20  (6.74E-7-24.23E+4) | 0.10  (2.97E-7-18.59E+4) | 3.67  (1.06E-5-38.02E+4) | 0.44  (1.39E-6-61.31E+4) | 0.01  (2.39E-10-41.05E+3) | 3.68  (1.06-5-6.27E+6) | 1.06  (1.73E-8-1.39E+9) | **N-STI+ BEHAV** |  |
| 1.52  (0.18-10.47) | 0.20  (1.56E-10-1.39E+7) | 0.25  (0.03-1.59) | 0.54  (0.07-4.86) | 0.33  (0.02-4.23) | 10.64  (0.71-177.9) | 1.26  (0.10-12.96) | 0.03  (1.92E-7-14.87) | 9.60  (0.32-514.5) | 1.95  (1.23E-5-5.79E+6) | 2.70  (1.93E-6-90.83E+4) | **STI+N-STI** |

PBO=placebo. BEHAV=behavioural therapy. STI=stimulants. N-STI=non-stimulants. AD=antidepressants. O-DRU=other unlicensed drugs. PUFA=polyunsaturated fatty acids. HERB=herbal therapy. N-STI+BEHAV=non-stimulants+behavioural therapy. STI+N-STI=stimulants+non-stimulants.

**S8 Table. Additional analyses: Network meta-analyses for secondary outcome of anxiety**

| **PBO** |  |  |  |  |  |  |  |  |  |  |  |
| --- | --- | --- | --- | --- | --- | --- | --- | --- | --- | --- | --- |
| 0.12  (7.17E-7-17.76E+3) | **BEHAV** |  |  |  |  |  |  |  |  |  |  |
| 1.63  (0.61-4.51) | 13.71  (8.32E-5-3.36E+6) | **STI** |  |  |  |  |  |  |  |  |  |
| 0.92  (0.34-3.40) | 7.89  (5.01E-5-1.79E+6) | 0.14  (1.57E-6-4634) | **N-STI** |  |  |  |  |  |  |  |  |
| 0.87  (0.08-8.02) | 7.38  (2.88E-5-2.73E+6) | 0.08  (9.91E-7-2662) | 0.92  (0.07-8.52) | **AD** |  |  |  |  |  |  |  |
| 0.78  (0.13-3.49) | 5.99  (2.85E-5-1.65E+6) | 0.06  (7.07E-7-3477) | 0.84  (0.10-4.43) | 0.93  (0.06-12.17) | **O-DRU** |  |  |  |  |  |  |
| 0.52  (0.13-2.22) | 4.33  (2.54E-e-85.51E+4) | 0.07  (6.40E-7-2300) | 0.56  (0.08-2.91) | 0.59  (0.04-9.72) | 0.66  (0.09-6.93) | **PUFA** |  |  |  |  |  |
| 1.21  (0.21-7.67) | 9.77  (5.83E-5-2.14E+6) | 0.05  (5.28E-7-1529) | 1.32  (0.15-10.13) | 1.41  (0.08-30.18) | 1.58  (0.17-21.56) | 2.39  (0.24-23.0) | **MIN** |  |  |  |  |
| 1.14  (0.15-8.22) | 9.34  (5.11E-e-2.50E+6) | 0.11  (1.24E-6-4042) | 1.23  (0.13-8.26) | 1.33  (0.09-21.25) | 1.44  (0.15-18.53) | 2.21  (0.18-25.05) | 0.93  (0.06-12.54) | **HERB** |  |  |  |
| 0.00  (5.30E-11-717) | 0.02  (1.17E-11-97.83E+3) | 0.09  (9.77E-7-3488) | 0.00  (5.16E-11-770) | 0.00  (3.99E-11-1066) | 0.00  (3.96E-11-1216) | 0.00  (1.13E-10-1559) | 0.00  (4.73E-11-660) | 0.00  (2.30E-11-847) | **STI+**  **BEHAV** |  |  |
| 9.98  (2.49E-4-72.68E+4) | 48.61  (0.03-8.22E+6) | 5.30E-4  (1.55E-13-2.96) | 10.67  (2.57E-4-83.78E+4) | 12.03  (2.76E-4-1.26E+6) | 12.56  (2.60E-4-1.15E+6) | 18.64  (4.27E-4-1.66E+6) | 8.06  (1.73E-4-6.61E+5) | 8.37  (1.83-4-87.22E+4) | 2136  (0.15-8.31E+12) | **N-STI+ BEHAV** |  |

PBO=placebo. BEHAV=behavioural therapy. STI=stimulants. N-STI=non-stimulants. AD=antidepressants. O-DRU=other unlicensed drugs. PUFA=polyunsaturated fatty acids. HERB=herbal therapy. N-STI+BEHAV=non-stimulants+behavioural therapy.

**S8 Table. Additional analyses: Sensitivity analysis - Network meta-analyses for efficacy and acceptability of commonly prescribed medications including different dosage of stimulants**

| **PBO** | **0.54**  **(0.40-0.74)** | 0.76  (0.52-1.14) | 0.59  (0.23-1.51) | 0.81  (0.53-1.26) | 0.87  (0.69-1.10) | **0.38**  **(0.17-0.82)** | 0.79  (0.54-1.16) | 0.67  (0.37-1.25) | 1.45  (0.38-5.97) |
| --- | --- | --- | --- | --- | --- | --- | --- | --- | --- |
| **5.06**  **(3.79-6.92)** | **MPH-LM** | 1.40  (0.90-2.19) | 1.08  (0.41-2.85) | 1.49  (0.89-2.45) | **1.59**  **(1.14-2.22)** | 0.69  (0.31-1.54) | 1.44  (0.89-2.34) | 1.23  (0.63-2.42) | 2.66  (0.68-11.18) |
| **5.92**  **(3.87-9.08)** | 1.17  (0.74-1.86) | **MPH-H** | 0.77  (0.28-2.12) | 1.06  (0.60-1.81) | 1.14  (0.76-1.69) | 0.50  (0.21-1.15) | 1.03  (0.59-1.75) | 0.88  (0.42-1.80) | 1.92  (0.47-8.15) |
| **9.88**  **(4.03-25.63)** | 1.94  (0.78-5.21) | 1.65  (0.63-4.73) | **AMPH-LM** | 1.37  (0.49-3.80) | 1.47  (0.56-3.84) | 0.65  (0.19-2.15) | 1.33  (0.48-3.64) | 1.13  (0.37-3.43) | 2.46  (0.47-13.41) |
| **7.02**  **(4.50-11.09)** | 1.39  (0.82-2.35) | 1.19  (0.66-2.11) | 0.71  (0.25-1.93) | **AMPH-H** | 1.07  (0.68-1.69) | 0.47  (0.19-1.14) | 0.97  (0.54-1.73) | 0.82  (0.39-1.77) | 1.79  (0.44-7.82) |
| **3.58**  **(2.74-4.75)** | **0.71**  **(0.51-0.98)** | **0.61**  **(0.38-0.97)** | 0.36  (0.14-0.92) | **0.51**  **(0.31-0.83)** | **ATX** | **0.44**  **(0.19-0.96)** | 0.90  (0.58-1.40) | 0.77  (0.40-1.48) | 1.67  (0.43-7.02) |
| **3.86**  **(1.77-8.16)** | 0.76  (0.35-1.62) | 0.65  (0.27-1.53) | 0.39  (0.11-1.25) | 0.55  (0.22-1.31) | 1.07  (0.49-2.37) | **CLON** | 2.06  (0.88-5.03) | 1.77  (0.66-4.78) | 3.80  (0.81-19.29) |
| **3.31**  **(2.26-4.90)** | 0.65  (0.40-1.06) | **0.56**  **(0.32-0.99)** | **0.33**  **(0.12-0.88)** | **0.47**  **(0.26-0.85)** | 0.92  (0.59-1.46) | 0.86  (0.37-2.07) | **GUAN** | 0.85  (0.41-1.78) | 1.84  (0.46-8.04) |
| **5.40**  **(2.96-10.25)** | 1.07  (0.55-2.11) | 0.92  (0.44-1.95) | 0.55  (0.18-1.65) | 0.77  (0.36-1.68) | 1.51  (0.78-3.03) | 1.41  (0.53-3.78) | 1.64  (0.80-3.44) | **MODAF** | 2.18  (0.50-10.28) |
| 2.36  (0.45-12.02) | 0.47  (0.09-3.21) | 0.40  (0.07-2.07) | 0.24  (0.04-1.50) | 0.33  (0.06-1.81) | 0.66  (0.13-3.35) | 0.61  (0.10-3.66) | 0.71  (0.13-3.75) | 0.43  (0.07-2.36) | **BUP** |

Data in blue represents efficacy (treatment response). Data in red represents acceptability (all-cause discontinuation). Results are the ORs in the column-defining treatment compared with the ORs in the row-defining treatment. For efficacy (acceptability), ORs higher than 1 favour the row-defining treatment. For acceptability, ORs lower than 1 favour the row-defining treatment. Significant results are in bold and underscored. PBO=placebo. MPH-LM=methylphenidate low to moderate dose. MPH-H=methylphenidate high dose. AMPH-LM=amphetamine low to moderate dose. AMPH-H=amphetamine high dose. ATX=atomoxetine. CLON=clonidine. GUAN=guanfacine. MODAF=modafinil. BUP=bupropion. OR=Odds ratio. CI=credibility interval. Dosage definitions: amphetamine low to moderate dose: ≤ 20 mg/day; amphetamine high dose: >20 mg/day; methylphenidate (short-acting) low to moderate dose: ≤ 30 mg/day; methylphenidate (short-acting) high dose >30 mg/day; methylphenidate (long-acting) low to moderate dose: ≤ 40 mg/day; methylphenidate (long-acting) low to moderate dose: > 40 mg/day.

**S8 Table. Additional analyses: Sensitivity analysis - Network meta-analyses for efficacy and acceptability of commonly prescribed medications including different type of formulations (short-acting and long-acting formulations)**

| **PBO** | **0.58**  **(0.42-0.83)** | **0.59**  **(0.42-0.85)** | 0.99  (0.09-7.36) | 0.74  (0.50-1.12) | 0.85  (0.68-1.07) | **0.22**  **(0.07-0.72)** | 0.60  (0.21-1.79) | 0.77  (0.52-1.13) | 0.67  (0.36-1.25) | 1.47  (0.38-5.98) |
| --- | --- | --- | --- | --- | --- | --- | --- | --- | --- | --- |
| **4.37**  **(3.20-6.02)** | **MPH-SA** | 1.00  (0.65-1.56) | 1.67  (0.15-12.88) | 1.27  (0.76-2.06) | **1.45**  **(1.01-2.05)** | 0.37  (0.11-1.21) | 1.03  (0.34-3.21) | 1.31  (0.78-2.16) | 1.14  (0.57-2.25) | 2.49  (0.65-10.37) |
| **6.39**  **(4.68-8.85)** | 1.46  (1.00-2.16) | **MPH-LA** | 1.67  (0.15-13.02) | 1.26  (0.76-2.09) | 1.44  (1.00-2.08) | 0.37  (0.11-1.26) | 1.02  (0.34-3.15) | 1.30  (0.77-2.15) | 1.13  (0.56-2.27) | 2.51  (0.62-10.41) |
| **24.89**  **(4.49-193.3)** | **5.65**  **(1.05-44.17)** | 3.90  (0.69-30.21) | **AMPH-SA** | 0.75  (0.10-8.64) | 0.86  (0.11-9.60) | 0.22  (0.02-3.32) | 0.62  (0.06-8.49) | 0.78  (0.10-8.86) | 0.68  (0.08-7.96) | 1.52  (0.13-23.78) |
| **7.03**  **(4.80-10.44)** | 1.61  (0.99-2.60) | 1.10  (0.66-1.75) | 0.28  (0.04-1.64) | **AMPH-LA** | 1.15  (0.74-1.77) | 0.29  (0.08-1.04) | 0.81  (0.26-2.60) | 1.04  (0.59-1.81) | 0.90  (0.44-1.89) | 1.99  (0.50-8.43) |
| **3.63**  **(2.83-4.70)** | 0.83  (0.59-1.17) | **0.57**  **(0.40-0.79**) | **0.15**  **(0.02-0.80)** | 0.52  (0.34-0.79) | **ATX** | **0.26**  **(0.08-0.85)** | 0.71  (0.24-2.16) | 0.90  (0.58-1.40) | 0.78  (0.41-1.50) | 1.73  (0.44-7.11) |
| **3.57**  **(1.71-7.49)** | 0.82  (0.39-1.71) | 0.56  (0.25-1.23) | **0.14**  **(0.02-0.91)** | 0.51  (0.22-1.17) | 0.98  (0.46-2.13) | **CLON-SA** | 2.75  (0.56-13.60) | **3.51**  **(1.00-12.26)** | 3.06  (0.82-11.76) | **6.77**  **(1.17-39.67)** |
| - | - | - | - | - | - | - | **CLON-LA** | 1.27  (0.40-3.89) | 1.10  (0.33-3.83) | 2.44  (0.44-14.55) |
| **3.05**  **(2.12-4.40)** | 0.70  (0.43-1.12) | **0.48**  **(0.29-0.77)** | **0.12**  **(0.02-0.69)** | **0.43**  **(0.25-0.74)** | 0.84  (0.54-1.29) | 0.86  (0.37-1.94) | - | **GUAN-LA** | 0.87  (0.42-1.83) | 1.90  (0.49-8.20) |
| **5.23**  **(2.90-9.61)** | 1.21  (0.63-2.31) | 0.83  (0.42-1.59) | 0.21  (0.03-1.29) | 0.75  (0.37-1.52) | 1.45  (0.76-2.72) | 1.48  (0.57-3.81) | - | 1.73  (0.87-3.50) | **MODAF** | 2.21  (0.50-10.10) |
| 2.06  (0.43-9.52) | 0.47  (0.10-2.15) | 0.32  (0.07-1.52) | **0.08**  **(0.01-0.81)** | 0.29  (0.06-1.42) | 0.57  (0.12-2.65) | 0.57  (0.11-3.04) | - | 0.67  (0.14-3.30) | 0.39  (0.08-2.01) | **BUP** |

Data in blue represents efficacy (treatment response). Data in red represents acceptability (all-cause discontinuation). Results are the ORs in the column-defining treatment compared with the ORs in the row-defining treatment. For efficacy (acceptability), ORs higher than 1 favour the row-defining treatment. For acceptability, ORs lower than 1 favour the row-defining treatment. Significant results are in bold and underscored. PBO=placebo. MPH-SA= methylphenidate short-acting. MPH-LA=methylphenidate long-acting. AMPH-SA=amphetamine short-acting. AMPH-LA=amphetamine long-acting. ATX=atomoxetine. CLON-SA= clonidine short-acting. CLON-LA=clonidine long-acting. GUAN-LA=guanfacine long-acting. MODAF=modafinil. BUP=bupropion. OR=Odds ratio. CI=credibility interval. No data or very imprecise for guanfacine short-acting.
