# Supplementary material for: The pharmacological and non-pharmacological treatment of attention deficit hyperactivity disorder in children and adolescents: A systematic review with network meta-analyses of randomised trials
Source: PLoS One. 2017 Jul 12;12(7):e0180355. doi: 10.1371/journal.pone.0180355 (PMC5507500; doi:10.1371/journal.pone.0180355)
Supplement: S9 Table — (DOCX) [file pone.0180355.s014.docx]

**S9 Table. Summary of model fit statistics from network meta-analysis by outcome**

|  | **Efficacy (treatment response)** | **Acceptability (all-cause discontinuation)** | **Tolerability (discontinuation due to AEs)** | **Serious AEs** | **Decreased weight** | **Anorexia** | **Insomnia** | **Sleep disturbances** | **Anxiety** |
| --- | --- | --- | --- | --- | --- | --- | --- | --- | --- |
| **Consistency model** | | | | | | | | | |
| **Number of data points** | 241 | 365 | 220 | 67 | 70 | 168 | 102 | 49 | 42 |
| **Residual deviance (posterior mean)** | 261.4 | 371.5 | 214.1 | 68.9 | 73.3 | 170.8 | 111.0 | 53.7 | 38.9 |
| **DIC** | 1426.27 | 1855.73 | 842.87 | 220.83 | 318.85 | 890.67 | 522.78 | 229.84 | 177.27 |
| **Inconsistency model** | | | | | | | | | |
| **Number of data points** | 241 | 365 | 220 | 67 | 70 | 168 | 102 | 49 | 42 |
| **Residual deviance (posterior mean)** | 258.0 | 379.3 | 217.0 | 65.8 | 74.39 | 172.1 | 107.2 | 51.5 | 40.2 |
| **DIC** | 1429.43 | 1881.88 | 851.00 | 217.90 | 321.30 | 893.63 | 520.21 | 230.00 | 180.95 |

AEs = adverse events. DIC = deviance information criteria.
