# Supplementary material for: The pharmacological and non-pharmacological treatment of attention deficit hyperactivity disorder in children and adolescents: A systematic review with network meta-analyses of randomised trials
Source: PLoS One. 2017 Jul 12;12(7):e0180355. doi: 10.1371/journal.pone.0180355 (PMC5507500; doi:10.1371/journal.pone.0180355)
Supplement: S10 Table — (DOCX) [file pone.0180355.s015.docx]

**S10 Table. Pairwise meta-analysis and heterogeneity for the primary outcomes**

**Class effect**

| **Comparisons vs PBO** | **Number of participants per comparison** | **Number of events**  **per comparison** | **Number of trials** | **Pairwise meta-analysis** | **Heterogeneity:**  **Cochran Q**  **P-value;**  ***I^2^* index (%)** | **Publication bias:**  **P-value Begg test*** | **Network meta-analysis**  **(base case)** |
| --- | --- | --- | --- | --- | --- | --- | --- |
| **Efficacy (response)** |  |  |  |  |  |  |  |
| **CONT** | - | - | - | - | - | - | 1.99 (0.98-4.15) |
| **WL** | - | - | - | - | - | - | 0.57 (0.20-1.62) |
| **BEHAV** | 8 | 1 | 1 | 3.86 (0.12-126.7) | - | - | 2.97 (1.53-5.88) |
| **COG** | 51 | 3 | 1 | 0.42 (0.04-4.99) | - | - | 0.70 (0.12-3.87) |
| **NF** | 55 | 5 | 2 | 5.36 (0.57-50.18) | 0.57; 0.0% | - | 1.96 (0.52-8.26) |
| **STI** | 5013 | 2321 | 25 | 5.21 (4.22-6.44) | 0.01; 46.6% | 0.58 | 6.21 (4.89-7.96) |
| **N-STI** | 5132 | 2165 | 28 | 3.37 (2.75-4.12) | 0.00; 48.7% | 0.01 | 3.95 (3.13-5.07) |
| **AD** | 137 | 49 | 3 | 15.99 (5.66-45.15) | 0.41; 0.0% | - | 8.52 (3.95-18.96) |
| **A-PSY** | 83 | 13 | 1 | 1.79 (0.53-6.03) | - | - | 1.36 (0.34-5.38) |
| **O-DRU** | 742 | 258 | 4 | 4.48 (2.03-9.87) | 0.03; 67.0% | - | 3.80 (2.04-7.14) |
| **STI+BEHAV** | 8 | 3 | 1 | 7.00 (0.28-178.5) | - | - | 13.62 (6.83-27.93) |
| **N-STI+BEHAV** | - | - | - | - | - | - | 6.05 (2.39-15.27) |
| **STI+N-STI** | 65 | 39 | 1 | 15.95 (4.41-57.66) | - | - | 15.18 (7.50-31.46) |
| **Acceptabibity** |  |  |  |  |  |  |  |
| **CONT** | 54 | 12 | 1 | 1.40 (0.38-5.12) | - | - | 0.55 (0.32-0.95) |
| **WL** | - | - | - | - | - | - | 0.33 (0.15-0.70) |
| **BEHAV** | 52 | 1 | 1 | 3.12 (0.12-80.12) | - | - | 0.58 (0.33-0.99) |
| **COG** | 284 | 27 | 5 | 1.67 (0.66-4.24) | 0.38; 4.6% | - | 1.32 (0.71-2.52) |
| **NF** | 48 | 7 | 2 | 0.37 (0.07-1.90) | 0.60; 0.0% | - | 0.59 (0.31-1.14) |
| **STI** | 4333 | 883 | 23 | 0.61 (0.45-0.83) | 0.00; 63.7% | 0.36 | 0.67 (0.54-0.83) |
| **N-STI** | 7325 | 1899 | 37 | 0.79 (0.67-0.94) | 0.01; 39.2% | 0.17 | 0.81 (0.67-0.97) |
| **AD** | 253 | 25 | 4 | 1.12 (0.45-2.78) | 0.49; 0.0% | - | 0.99 (0.44-2.28) |
| **A-PSY** | 126 | 8 | 2 | 2.46 (0.33-18.25) | 0.26; 22.8% | - | 1.25 (0.43-3.63) |
| **O-DRU** | 766 | 207 | 5 | 0.66 (0.36-1.21) | 0.16; 39.8% | - | 0.73 (0.42-1.29) |
| **STI+BEHAV** | - | - | - | - | - | - | 0.37 (0.21-0.67) |
| **N-STI+BEHAV** | - | - | - | - | - | - | 1.00 (0.37-2.84) |
| **STI+N-STI** | 127 | 39 | 2 | 0.27 (0.09-0.77) | 0.22; 33.2% | - | 0.47 (0.27-0.81) |

Values of meta-analyses represent odds ratios (compared to placebo) with 95% confidence or credible intervals. PBO=placebo. CONT=control. WL=waiting list. BEHAV=behavioural therapy. COGN=cognitive training. NF=neurofeedback. STI=stimulants. N-STI=non-stimulants. AD=antidepressants. A-PSY=antipsychotics. O-DRU=other unlicensed drugs. STI+BEHAV=stimulants+behavioural therapy. N-STI+BEHAV=non-stimulants+behavioural therapy. STI+N-STI=stimulants+non-stimulants. *Begg’s test for publication bias if at least 10 studies (observations).

**(cont.) Pairwise meta-analysis and heterogeneity for the primary outcomes. Individual effect**

| **Comparisons vs PBO** | **Number of participants per comparison** | **Number of events**  **per comparison** | **Number of trials** | **Pairwise meta-analysis** | **Heterogeneity:**  **Cochran Q**  **P-value;**  ***I^2^* index (%)** | **Publication bias:**  **P-value Begg test*** | **Network meta-analysis**  **(base case)** |
| --- | --- | --- | --- | --- | --- | --- | --- |
| **Efficacy (response)** |  |  |  |  |  |  |  |
| **CONT** | - | - | - | - | - | - | 1.29 (0.65-2.59) |
| **WL** | - | - | - | - | - | - | 0.29 (0.10-0.85) |
| **P training** | 8 | 1 | 1 | 3.86 (0.12-126.7) | - | - | 1.19 (0.50-2.77) |
| **C, P, T training** | - | - | - | - | - | - | 2.73 (1.41-5.39) |
| **WM training** | 51 | 3 | 1 | 0.42 (0.04-4.98) | - | - | 0.34 (0.01-5.82) |
| **Attention training** | - | - | - | - | - | - | 4.17x10^12^ (1.63-12.44x10^28^) |
| **NF** | 37 | 4 | 1 | 9.49 (0.48-188.68) | - | - | 1.61x10^13^ (4.57-23.91x10^28^) |
| **MPH** | 3161 | 1342 | 20 | 5.04 (3.99-6.37) | 0.07; 33.7% | 0.40 | 5.26 (4.09-6.82) |
| **AMPH** | 1981 | 999 | 7 | 6.41 (4.00-10.28) | 0.001; 72.3% | - | 7.45 (5.10-11.09) |
| **ATX** | 2738 | 1020 | 16 | 3.75 (2.66-5.28) | 0.001; 64.5% | 0.04 | 3.63 (2.81-4.73) |
| **GUAN** | 2319 | 1129 | 10 | 2.87 (2.31-3.55) | 0.257; 20.2% | 0.09 | 3.29 (2.27-4.82) |
| **CLON** | 186 | 65 | 3 | 3.47 (1.80-6.69) | 0.64; 0.0% | - | 3.96 (1.89-8.41) |
| **BUP** | - | - | - | - | - | - | 2.41 (0.48-11.63) |
| **DESIP** | 114 | 39 | 2 | 23.51 (4.59-120.46) | 0.25; 25.1% | - | 36.76 (9.17-214.0) |
| **VENLAF** | - | - | - | - | - | - | 4.07 (0.73-22.36) |
| **REBOX** | - | - | - | - | - | - | 3.58 (0.57-22.11) |
| **MODAF** | 742 | 258 | 4 | 4.48 (2.03-9.87) | 0.03; 67.0% | - | 5.51 (3.04-10.32) |
| **THIO** | 83 | 13 | 1 | 1.79 (0.53-6.03) | - | - | 1.04 (0.28-3.78) |
| **MPH + P training** | 8 | 3 | 1 | 21.00 (0.64-690.0) | - | - | 55.63 (3.18-29.52x10^2^) |
| **MPH + C,P,T training** | - | - | - | - | - | - | 15.82 (8.06-32.80) |
| **MPH+CLON** | 65 | 39 | 1 | 15.95 (4.41-57.66) | - | - | 21.91 (5.52-105.4) |
| **MPH+THIO** | 84 | 29 | 1 | 9.87 (3.23-30.12) | - | - | 5.97 (1.90-19.59) |
| **ATX + P training** | - | - | - | - | - | - | 2.48 (0.51-11.79) |
| **ATX + C,P,T training** | - | - | - | - | - | - | 5.53 (2.19-14.06) |
| **Acceptabibity** |  |  |  |  |  |  |  |
| **CONT** | 54 | 12 | 1 | 1.40 (0.38-5.12) | - | - | 0.55 (0.32-0.98) |
| **WL** | - | - | - | - | - | - | 0.30 (0.13-0.69) |
| **P training** | 82 | 5 | 2 | 3.36 (0.49-23.04) | 0.95; 0.0% | - | 0.69 (0.35-1.36) |
| **C, P, T training** | - | - | - | - | - | - | 0.37 (0.17-0.80) |
| **C training** | - | - | - | - | - | - | 0.25 (0.04-1.98) |
| **T training** | - | - | - | - | - | - | 0.76 (0.08-8.49) |
| **WM training** | 254 | 23 | 4 | 1.60 (0.49-5.22) | 0.29; 19.7% | - | 1.75 (0.75-4.14) |
| **Attention training** | - | - | - | - | - | - | 0.65 (0.22-1.92) |
| **NF** | 48 | 7 | 2 | 0.37 (0.07-1.90) | 0.60; 0.0% | - | 0.40 (0.19-0.82) |
| **MPH** | 3007 | 708 | 20 | 0.51 (0.37-0.71) | 0.00; 60.0% | 0.33 | 0.59 (0.46-0.75) |
| **AMPH** | 1737 | 350 | 6 | 0.72 (0.40-1.30) | 0.00; 75.3% | - | 0.78 (0.52-1.18) |
| **ATX** | 4390 | 971 | 24 | 0.87 (0.70-1.07) | 0.06; 33.7% | 0.05 | 0.85 (0.68-1.07) |
| **GUAN** | 2294 | 748 | 9 | 0.77 (0.61-0.98) | 0.19; 28.5% | - | 0.79 (0.54-1.14) |
| **CLON** | 448 | 130 | 4 | 0.33 (0.13-0.88) | 0.06; 59.0% | - | 0.40 (0.20-0.78) |
| **BUP** | 139 | 12 | 2 | 1.52 (0.38-6.19) | 0.36; 0.0% | - | 1.54 (0.39-6.76) |
| **DESIP** | 114 | 13 | 2 | 0.77 (0.16-3.80) | 0.26; 22.3% | - | 0.70 (0.17-2.89) |
| **VENLAF** | - | - | - | - | - | - | 0.64 (0.02-19.78) |
| **REBOX** | - | - | - | - | - | - | 0.73 (0.12-4.44) |
| **MODAF** | 766 | 207 | 5 | 0.66 (0.36-1.21) | 0.16; 39.8% | - | 0.67 (0.37-1.24) |
| **RISP** | - | - | - | - | - | - | 0.56 (0.13-2.77) |
| **THIO** | 83 | 6 | 1 | 5.69 (0.64-51.04) | - | - | 3.01 (0.57-17.81) |
| **ARIP** | 43 | 2 | 1 | 0.71 (0.04-12.13) | - | - | 0.61 (0.02-25.34) |
| **MPH + P training** | - | - | - | - | - | - | 0.50 (0.18-1.44) |
| **MPH + C training** | - | - | - | - | - | - | 0.18 (0.02-1.74) |
| **MPH + C,P,T training** | - | - | - | - | - | - | 0.24 (0.12-0.50) |
| **MPH+ATX** | - | - | - | - | - | - | 1.00 (0.03-48.87) |
| **MPH+CLON** | 127 | 39 | 2 | 0.27 (0.09-0.77) | 0.22; 33.2% | - | 0.32 (0.13-0.77) |
| **MPH+THIO** | 84 | 4 | 1 | 3.15 (0.31-31.62) | - | - | 1.61 (0.25-10.33) |
| **ATX + P training** | - | - | - | - | - | - | 0.87 (0.21-3.74) |
| **ATX + C,P,T training** | - | - | - | - | - | - | 1.23 (0.29-6.03) |

Values of meta-analyses represent odds ratios (compared to placebo) with 95% confidence or credible intervals.

**(cont.) Forest plots. Pairwise meta-analysis and heterogeneity for the primary outcomes.**

**Efficacy (response)**

Forest plots were generated when 5 or more studies (observations) available.

**Forest plots. Pairwise meta-analysis and heterogeneity for the primary outcomes.**

**Acceptability**

Forest plots were generated when 5 or more studies (observations) available.
