# Supplementary material for: The pharmacological and non-pharmacological treatment of attention deficit hyperactivity disorder in children and adolescents: A systematic review with network meta-analyses of randomised trials
Source: PLoS One. 2017 Jul 12;12(7):e0180355. doi: 10.1371/journal.pone.0180355 (PMC5507500; doi:10.1371/journal.pone.0180355)
Supplement: S1 Fig — (DOCX) [file pone.0180355.s016.docx]

**S1 Figure. PRISMA Flow Diagram for Study Selection Process**

Records identified through database searching:

PubMed (Jan 1, 2005-Apr 7, 2016): n =1772 Cochrane Library/Cochrane Database of Systematic Reviews: n = 30

Additional records identified through other sources:

n = 8

## Identification

Citations screened from PubMed ‘related search’: n = 4535*

Records screened after duplicates removed:
n = 1785

Records excluded:
n = 1669

Additional citations screened/ identified by complementary searches: n = 88

Records of systematic reviews included: n = 116

## Screening

*Related search based on 215 included articles as of 25^th^ April 2016 (limits: 1/1/2014-6/5/2016)

Full-text articles excluded: n = 540

- Crossover design with ineligible follow-up and/or not reported outcome: n = 207
- Irrelevant population, intervention or outcome: n= 194
- Not randomised controlled trial: n = 95
- Data not abstractable/not retrievable: n = 25
- Follow-up < 3 weeks: 19

## Eligibility

## Included

Full-text articles and citations of clinical trials assessed for eligibility:
n = 804

Trial citations from included systematic reviews: n = 2153

Potential trial citations screened from included systematic reviews: n = 983

Records excluded
n = 4802

Duplicates excluded:
n = 1170

Articles included in both qualitative and quantitative synthesis/meta-analysis: n = 264 (studies included n = 190)
