# Supplementary material for: The pharmacological and non-pharmacological treatment of attention deficit hyperactivity disorder in children and adolescents: A systematic review with network meta-analyses of randomised trials
Source: PLoS One. 2017 Jul 12;12(7):e0180355. doi: 10.1371/journal.pone.0180355 (PMC5507500; doi:10.1371/journal.pone.0180355)
Supplement: S2 Fig — (DOCX) [file pone.0180355.s017.docx]

**S2 Figure. Network geometry.**

1. **Primary outcome: efficacy among therapeutic classes**

**(26 treatment nodes, 113 studies, 8916 outcome events, and 19398 participants)**

1. **Primary outcome: efficacy among individual therapies**

**(35 treatment nodes, 101 studies, 8097 outcome events, and 17715 participants)**

1. **Primary outcome: acceptability among therapeutic classes**

**(32 treatment nodes, 171 studies, 4822 outcome events, and 22961 participants)**

1. **Primary outcome: acceptability among individual therapies**

**(52 treatment nodes, 165 studies, 4696 outcome events, and 21991 participants)**

1. **Secondary outcome: tolerability among therapeutic classes**

**(23 treatment nodes, 105 studies, 740 outcome events, and 18863 participants)**

1. **Secondary outcome: tolerability among individual therapies**

**(36 treatment nodes, 103 studies, 725 outcome events, and 18204 participants)**

1. **Secondary outcome: serious adverse events among therapeutic classes**

**(8 treatment nodes, 32 studies, 96 outcome events and 9212 participants)**

1. **Secondary outcome: serious adverse events among individual therapies**

**(12 treatment nodes, 31 studies, 93 outcome events, and 8751 participants)**

1. **Secondary outcome: decreased weight among therapeutic classes**

**(10 treatment nodes, 34 studies, 710 outcome events and 6453 participants)**

1. **Secondary outcome: decreased weight among individual therapies**

**(12 treatment nodes, 34 studies, 710 outcome events, and 6453 participants)**

1. **Secondary outcome: anorexia among therapeutic classes**

**(20 treatment nodes, 81 studies, 2440 outcome events and 15856 participants)**

1. **Secondary outcome: anorexia among individual therapies**

**(27 treatment nodes, 79 studies, 2380 outcome events, and 15227 participants)**

1. **Secondary outcome: insomnia among therapeutic classes**

**(11 treatment nodes, 49 studies, 1068 outcome events and 11722 participants)**

1. **Secondary outcome: insomnia among individual therapies**

**(17 treatment nodes, 47 studies, 1034 outcome events, and 11033 participants)**

1. **Secondary outcome: sleep disturbances among therapeutic classes**

**(16 treatment nodes, 22 studies, 371 outcome events and 2125 participants)**

1. **Secondary outcome: sleep disturbances among individual therapies**

**(18 treatment nodes, 21 studies, 328 outcome events, and 1957 participants)**

1. **Secondary outcome: anxiety among therapeutic classes**

**(14 treatment nodes, 21 studies, 256 outcome events and 2961 participants)**

1. **Secondary outcome: anxiety among individual therapies**

**(18 treatment nodes, 21 studies, 256 outcome events, and 2961 participants)**

1. **Secondary outcome: syncope among therapeutic classes**

**(4 treatment nodes, 8 studies, 13 outcome events and 2519 participants)**

1. **Secondary outcome: syncope among individual therapies**

**(5 treatment nodes, 7 studies, 12 outcome events, and 2058 participants)**

Solid lines represent direct comparisons within randomised trials. Nodes in blue represent controls. Nodes in green represent psychological interventions. Nodes in grey represent pharmacological interventions. Nodes in purple represent complementary and alternative medicine interventions. Nodes in red represent combined interventions. Size of node is proportional to number of randomised trials, and thickness of line connecting nodes is proportional to number of randomised trials directly comparing the two treatments. AMPH: amphetamine. ARIP: aripiprazole. ATX: atomoxetine. CLON=clonidine. GUAN=guanfacine. MODAF=modafinil. CARBA=carbamazepine. BUP=bupropion. DESIP=desipramine. REBOX=reboxetine. VENLAF=venlafaxine. RISP=risperidone. THIO=thioridazine. L-CARN=L-carnitine. HYP=hypericum. HOMEO=homeopathy. C, P, T training: child, parent and/or teacher training. P training=parent training. C training=child training. T training=teacher training. WM training=working memory training
