# Supplementary material for: The pharmacological and non-pharmacological treatment of attention deficit hyperactivity disorder in children and adolescents: A systematic review with network meta-analyses of randomised trials
Source: PLoS One. 2017 Jul 12;12(7):e0180355. doi: 10.1371/journal.pone.0180355 (PMC5507500; doi:10.1371/journal.pone.0180355)
Supplement: S3 Fig — (DOCX) [file pone.0180355.s018.docx]

**S3 Figure. Publication bias for the primary outcomes**

1. **Funnel plot for primary outcome of efficacy: STI vs PBO**

1. **Funnel plot for primary outcome of efficacy: N-STI vs PBO**

1. **Funnel plot for primary outcome of efficacy: MPH vs PBO**

1. **Funnel plot for primary outcome of efficacy: ATX vs PBO**

1. **Funnel plot for primary outcome of efficacy: GUAN vs PBO**

1. **Funnel plot for primary outcome of acceptability: STI vs PBO**

1. **Funnel plot for primary outcome of acceptability: N-STI vs PBO**

1. **Funnel plot for primary outcome of acceptability: MPH vs PBO**

1. **Funnel plot for primary outcome of acceptability: ATX vs PBO**

PBO=placebo. STI=stimulants. N-STI=non-stimulants. MPH= methylphenidate. TX=atomoxetine. GUAN=guanfacine. Funnel plots were generated when 10 or more studies (observations) available.
